# Atroposelective Suzuki–Miyaura Coupling to Form 2‐Amino‐2′‐Hydroxybiphenyls Enabled by sRuPhos

**DOI:** 10.1002/anie.202520698

**Published:** 2025-12-24

**Authors:** Hamzah Sharif, Luke A. McCall, Matthew G. Sanders, Robert J. Phipps

**Affiliations:** ^1^ Yusuf Hamied Department of Chemistry University of Cambridge Lensfield Road Cambridge CB2 1EW UK; ^2^ Oncology Targeted Discovery, Oncology R&D AstraZeneca The Discovery Centre, Cambridge Biomedical Campus, 1 Francis Crick Avenue Cambridge CB2 0AA UK

**Keywords:** Atropisomers, Biaryls, Cross‐coupling, Palladium, Phosphine

## Abstract

Atropisomeric biaryls are increasingly important in molecules of pharmaceutical interest. Asymmetric versions of the Suzuki–Miyaura coupling arguably constitute the most direct and attractive route to these without resorting to wasteful preparative separations or resolution steps. Herein, we report a new chiral phosphine ligand sRuPhos, a sulfonated form of RuPhos, which permits the coupling of unprotected *ortho*‐bromoanilines and *ortho*‐phenolic boronate esters with extremely high ee (up to 99%) to form 2‐amino‐2′‐hydroxybiphenyls. *N*‐alkylation can be accommodated on the aniline nitrogen and sRuPhos is also able to form highly hindered 2,2′‐biphenols, which could not be achieved in our previous studies using the related chiral ligand sSPhos. Given the great importance of biaryl atropisomers in medicinal chemistry, as well as the potential utility of the 2‐amino‐2′‐hydroxybiphenyl motif in chiral catalyst design, we anticipate that this reaction will soon find utility and that sRuPhos may be a useful ligand in the development of other asymmetric palladium‐catalysed processes.

## Graphical abstract

We report a new chiral phosphine ligand, sRuPhos, which permits the highly enantioselective Suzuki–Miyaura coupling of *ortho*‐aniline and *ortho*‐phenol building blocks. This results in 2‐amino‐2′‐hydroxybiphenyls with very high enantiomeric excesses, products that will have application in pharmaceutical synthesis and ligand design for catalysis. Attractive non‐covalent interactions between ligand and substrate are proposed to play a crucial role in the reaction outcome.

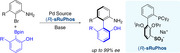

The Suzuki–Miyaura coupling has huge importance in contemporary organic chemistry and has enabled extensive exploration of previously limited chemical space around the biaryl motif.^[^ ^1^ ^]^ Continuous ligand development has enabled very hindered biaryl bonds to be formed, in many cases leading to atropoisomeric compounds.^[^ ^2^ ^]^ It is now increasingly common to encounter atropisomeric biaryl compounds in medicinal chemistry campaigns, meaning that the development of enantioselective variants of the Suzuki–Miyaura coupling is of paramount importance.^[^ ^3^, ^4^, ^5^, ^6^ ^]^ Since seminal reports in 2000,^[^ ^7^, ^8^ ^]^ much effort has focused on identifying new chiral ligands able to combine the required high reactivity with ability to induce enantioselectivity.^[^ ^9^, ^10^, ^11^, ^12^, ^13^, ^14^, ^15^, ^16^, ^17^, ^18^, ^19^, ^20^, ^21^, ^22^, ^23^, ^24^, ^25^, ^26^, ^27^, ^28^, ^29^, ^30^, ^31^, ^32^, ^33^, ^34^, ^35^, ^36^, ^37^, ^38^, ^39^, ^40^ ^]^ Despite this attention, a survey of the literature reveals sizable limitations amongst current methods. First, it is very rare for a nitrogen substituent to be included at the biaryl axis, with examples possessing carbon, oxygen, or phosphorous‐based groups much more common (Figure 1a, left). Second, there are very few protocols able to form biphenyl‐type products with high enantiomeric excesses; most require at least one coupling partner to be an extended π‐system such as a naphthalene, forming phenyl–naphthyl or binaphthyl‐type products (Figure 1a, right).^[^ ^41^, ^42^ ^]^ Tang and coworkers have recently made significant advances on both counts using the ligand **BaryPhos** (Figure 1b).^[^ ^43^, ^44^, ^45^ ^]^ In 2020 they demonstrated that *ortho*‐formyl and/or methoxy‐substituted phenyl coupling partners could form biphenyl‐type products in high ee.^[^ ^44^ ^]^ In 2022 they showed that *ortho*‐nitro substituted aryl bromides could be coupled with high ee, a limitation being that the partner aryl boronic acid should be naphthalene‐based or possess an *ortho*‐vinyl substituent.^[^ ^45^, ^46^ ^]^ A recent report from Zhu and coworkers featured four examples involving highly enantioselective coupling of aryl bromides featuring an *ortho*‐trifluoroacetamide group.^[^ ^47^ ^]^ Shi and coworkers reported a single scope example with an *ortho*‐aniline partner.^[^ ^32^ ^]^ Despite these notable examples, it is evident that the lack of asymmetric Suzuki–Miyaura methods able to couple partners bearing amine‐based *ortho* substituents is a significant limitation for this powerful methodology, particularly to form challenging biphenyl‐type products, which constitute the most generally‐applicable building blocks.

**Figure 1:**
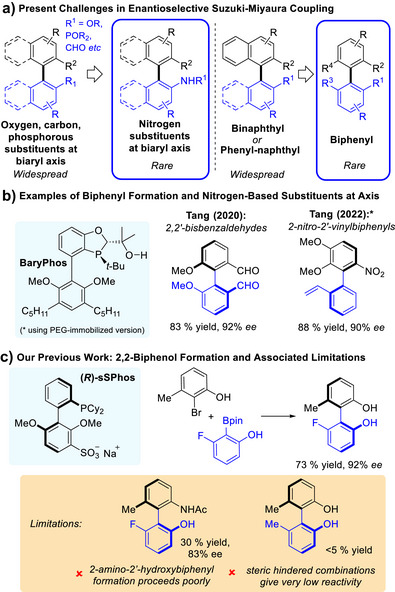
Present challenges in enantioselective Suzuki–Miyaura coupling and selected prior examples.

We previously reported the coupling of *ortho*‐hydroxyphenyl partners to form 2,2′‐biphenols with high ee (Figure 1c).^[^ ^48^ ^]^ This used **sSPhos**, a sulfonated version of SPhos,^[^ ^49^, ^50^ ^]^ which we could resolve into enantiomerically pure form.^[^ ^51^, ^52^ ^]^ We hypothesised that the ligand sulfonate group engages in hydrogen bonding interactions with the phenolic partners, providing a high degree of organisation during C─C bond formation, leading to high levels of enantioenrichement in the product. However, our protocol suffered from several significant limitations (Figure 1c, inset box). First, the introduction of an *N*‐acetyl amino substituent in place of one hydroxyl resulted in low yield (30%), albeit with promising enantiomeric excess (83%). Second, large substituents around the biaryl axis resulted in only trace conversion for 2,2′‐biphenol formation, revealing the limit of **sSPhos**’ ability to couple two highly hindered partners. In this work we disclose a new chiral ligand **sRuPhos** which can overcome these limitations to deliver synthetically valuable unprotected 2‐amino‐2′‐hydroxybiphenyl products with extremely high enantioselectivity. In addition to being highly relevant biaryl building blocks in pharmaceutical synthesis, the 2‐amino‐2′‐hydroxybiphenyl motif is closely related to the privileged and versatile chiral building block NOBIN (2‐amino‐2′‐hydroxy‐1,1′‐binaphthyl), a starting point for numerous chiral catalysts.^[^ ^53^, ^54^, ^55^ ^]^ Furthermore, **sRuPhos** can form the hindered 2,2′‐biphenols that were previously inaccessible and these are important motifs in natural product synthesis^[^ ^56^ ^]^ and ligand design.^[^ ^57^ ^]^ The new ligand is able to significantly expand, beyond the present state‐of‐the art, products accessible through asymmetric Suzuki–Miyaura coupling and we anticipate it will be of utility in medicinal chemistry applications and beyond.

Although we had carried out preliminary experiments related to aniline coupling previously using *N*‐acetylated 2‐bromoaniline, we surmised that an ideal protocol for the synthesis of chiral 2‐amino‐2′‐hydroxybiphenyls would dispense with a protecting group on the nitrogen atom, allowing direct Suzuki–Miyaura coupling of *ortho*‐aniline and *ortho*‐phenol partners. We began with evaluation of the coupling of bromoaniline **1a** and phenol boronate ester **2a** under previously optimised conditions using (*R*)‐**sSPhos** as ligand (Table 1). The yield of the desired biphenyl **3a** was very low but we were excited to note that it was obtained with a remarkable 99% ee, suggesting that the unprotected aniline has outstanding compatibility with our ligand, in terms of stereoselectivity (entry 1).^[^ ^58^ ^]^ We attempted to improve the yield by increasing the temperature to 60 °C (entry 2) or removing water from the solvent and increasing temperature further, but these changes resulted in a maximum of 29% yield achieved at 80 °C, accompanied by a drop in ee to 91% (entries 3 and 4). We noticed the formation of significant amounts of *C*_2_‐symmetric **4a**, resulting from boronate ester homocoupling. This was formed in 93% ee and was accompanied by formation of a byproduct arising from protodebromination of **1a**. Based on insightful precedent from Sieber and coworkers, we hypothesised that in our system the reductive elimination step to form **3a** is so challenging that protonolysis of the preceding diarylpalladium(II) intermediate becomes a competitive, in this case major, pathway.^[^ ^59^, ^60^, ^61^ ^]^ After protonolysis, a second transmetalation can occur, followed by a less challenging reductive elimination to give **4a**.^[^ ^62^ ^]^ The ability of **sSPhos** to promote reductive elimination to give **4a** was established in our previous work where it had been obtained intentionally.^[^ ^48^ ^]^ We anticipated that the solution to this problem may be to synthesise a bulkier variant of **sSPhos** which may better promote reductive elimination, but still retain the essential components of the ligand design that lead to high enantioselectivity. We first evaluated **s(*t*BuSPhos)**, which we had previously reported in racemic form, but this gave very low conversion to **3a**, suggesting that increased bulk on the phosphine is not productive (entry 5).^[^ ^63^ ^]^ To provide a more subtle steric increase at a different site of the ligand scaffold, we targeted the novel sulfonated phosphine **sRuPhos**, in which the two methoxy groups of **sSPhos** are replaced by *iso*propoxy.^[^ ^64^ ^]^ Sulfonation of **RuPhos** required very mild conditions in order to avoid acid‐promoted elimination of propene and the resulting enantiomers were separated by preparative chiral SFC to obtain (*R*)‐**sRuPhos** (see Supporting Information for details).^[^ ^65^ ^]^ Use of this new ligand produced immediate improvement; at 60 °C with water as an additive, **3a** was formed in 55% yield and 99% ee (entry 6, compared with entry 2 for **sSPhos**). This could be improved to 70% yield in anhydrous toluene, retaining 99% ee (entry 7, compared with entry 3 for **sSPhos**).^[^ ^66^ ^]^

**Table 1:** Reaction optimisation^a)^ ^,^ ^b)^

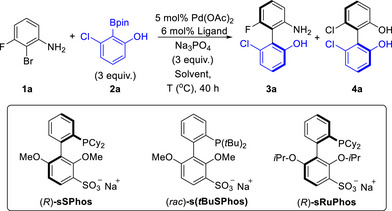						
Entry	Ligand	*T* °C	Solvent	% Yield **3a**	%ee **3a**	% Yield **4a**
1	*(R)*‐**sSPhos**	40	PhMe:H_2_O (19:1)	10	99	34
2	*(R)*‐**sSPhos**	60	PhMe:H_2_O (19:1)	23	94	56
3	*(R)*‐**sSPhos**	60	PhMe	19	93	54
4	*(R)*‐**sSPhos**	80	PhMe	29	91	66^c)^
5	*rac‐* **s(*t*BuSPhos)**	60	PhMe:H_2_O (19:1)	<5	–	<5
6	*(R)*‐**sRuPhos**	60	PhMe:H_2_O (19:1)	55	99	42
7	*(R)*‐**sRuPhos**	60	PhMe	(70)	(99)	30

^a)^ Yields determined by ^1^H NMR with reference to internal standard. Values in parentheses refer to isolated yield.

^b)^ ee determined by SFC analysis of the crude reaction mixture.

^c)^ ee of **4a** determined to be 93%.

We proceeded to evaluate the scope of the transformation (Scheme 1). At first, we retained the chloro‐substituted aryl boronate **2a** and added substitution to **1a** in the form of additional fluorines (**3b**, **3c**), a chlorine (**3d**) and a nitro group (**3e**) and in all cases enantioselectivity remained outstanding. We then evaluated a fluoro‐substituted aryl boronate in combination with variously substituted *ortho*‐bromoanilines. These included examples bearing chlorine atom(s) (**3f**‐**3h**), a methyl at the biaryl axis (**3i**), a fused cyclohexane ring (**3j**), and a methyl and a chlorine (**3k**). The absolute stereochemistry of **3f** was determined by X‐ray crystallography (CCDC deposition number 2418029 for **3f**). We evaluated a difluorinated boronate ester in combination with a selection of aryl bromides (**3l**‐**3o**). It is notable that of the 15 substrates illustrated so far, all but two were obtained in the 96%–99% ee range, emphasising the extremely high level of stereocontrol imparted by the new **sRuPhos** ligand. We also evaluated several alkyl‐substituted, phenolic boronate esters, including a methyl‐substituted example (**3p**) and one incorporating a fused cyclohexane ring (**3q**). Interestingly, an aryl bromide that gives rise to trisubstitution at the biaryl axis gave slightly reduced ee at 80% (**3r**). This reaction was run at 40 °C after the usual reaction temperature of 60 °C was found to give 50% ee. We subsequently determined the racemisation half‐life of **3r** to be 25.9 h at 60 °C. This equates to a rotation barrier of 27.8 kcalmol^−1^ placing this at the boundary between a Class 2 and Class 3 atropisomer.^[^ ^67^, ^68^ ^]^ It is notable that the mild conditions under which sRuPhos promotes coupling under means that enantioselective couplings to form atropisomers of only moderate stability can still be achieved. We evaluated whether *N*‐alkyl substitution could be tolerated on the aniline and were pleased to find that *N*‐Me (**3s, 3t**), *N*‐Et (**3u, 3v**) and *N*‐Bn (**3w**) partners all gave excellent enantioselectivity. Moving to the corresponding *N*,*N*‐dimethylaniline completely shut down the coupling, presumably due to excessive steric hindrance (**3x**). Interestingly, an aryl bromide bearing a nitro group was accommodated – although the yield was modest, the ee was promising at 86% (**3y**).

**Scheme 1:**
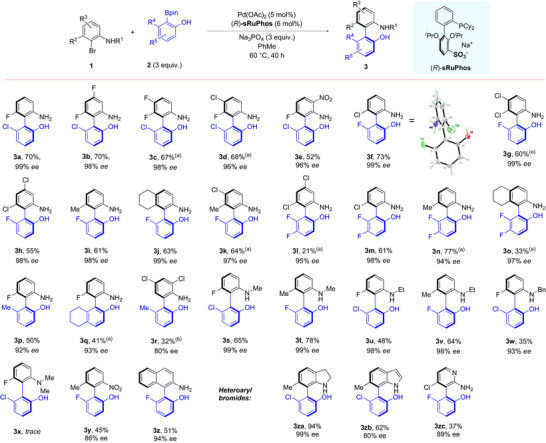
Scope of enantioselective Suzuki–Miyaura coupling to form axially chiral 2‐amino‐2′‐hydroxybiphenyls. Yield and ee values correspond to isolated material. ^(a)^ Reaction temperature 80 °C. ^(b)^ Reaction temperature 40 °C.

Although one of the key advantages of our atroposelective cross‐coupling is that we can accommodate phenyl‐type partners, we were able to show that a naphthyl bromide could engage in high ee, to form a half‐NOBIN‐like product with a single extended aromatic system (**3z**). We next examined heteroaryl bromides and found that an indolinyl bromide gave an excellent result (**3za**). Furthermore, an analogous indole gave a slightly reduced but still synthetically useful enantioselectivity (**3zb**). Finally, we could include a pyridine nitrogen into the upper ring with high selectivity (**3zc**). Although the yield of this compound is moderate, we found that at higher temperatures of 80 and 100 °C the ee was very much reduced suggesting the barrier to racemisation of this compound is lower than its non‐pyridinyl analogue **3f** (see Supporting Information for details). Generally, we experienced the main scope limitation not to be enantioselectivity but rather reactivity when steric crowding around the biaryl axis becomes prohibitively high (see Supporting Information for unsuccessful examples).

We next sought to determine whether **sRuPhos** may be able to address limitations of our previously reported Suzuki‐Miyaura coupling to form 2,2′‐biphenols.^[^ ^48^ ^]^ Using **sSPhos**, reactivity had been low or negligible for more hindered partner combinations; **4a** could be obtained in low to moderate yield and **4b** in only trace amounts (Scheme 2a).^[^ ^69^ ^]^ In contrast, using **sRuPhos**, **4a** could be obtained in 96% yield, 98% ee and **4b** in 40% yield, 90% ee. Although the latter yield is still moderate, given the highly hindered nature of the bond being formed combined with the excellent stereocontrol, we believe this to be a significant advancement. We further demonstrate the superiority of **sRuPhos** for accessing challenging 2,2′‐biphenols in the synthesis of **4c** and **4d**, which both gave only trace conversion when evaluated using **sSPhos**. An observation in our previous work was that in order to obtain > 90% ee, both arene partners should possess an *ortho* hydroxy group. If one was a methoxy, lower but still significant enantioselectivity could be obtained, as in **4e** and **4f** (Scheme 2b, 85% and 78% ee). Based on the observation that **sRuPhos** provides generally improved enantioselectivity we reevaluated these and were pleased to observe both increase to 94% and 91% ee respectively. Two further substrates gave good yields and high ee with **sRuPhos** where **sSPhos** had given poorer outcomes (**4** **g**, **4** **h**). This coupling type could be strategically useful in situations requiring one oxygen of a non‐symmetrical 2,2′‐biphenol to be selectively protected.

**Scheme 2:**
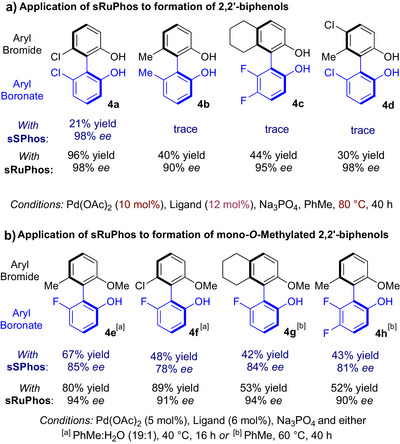
Application of **sRuPhos** to improve outcomes with 2,2′‐biphenols a) and mono‐*O*‐methylated variants b).

Based on the results presented, it could be speculated that so long as one coupling partner is phenolic, there are few limitations for the second. However, this is not the case, as evidenced by outcomes using partners which have no oxygen or nitrogen atoms flanking the biaryl axis: **5a** gives only 44% ee and **5b** only 8% (Scheme 3a). We evaluated a substrate in which methoxy potentially competes with aniline on the same aryl bromide and found that a reduced 71% ee was obtained (Scheme 3b, **5c**). An analogous competition between phenol and aniline reduced the ee further, reflecting a situation where two effective “directing groups” in this chemistry may be directly competing in a detrimental fashion (**5d**). We consider the use of unprotected anilines a distinct advantage in this methodology but were intrigued as to the effect that electron‐withdrawing groups on nitrogen might have on ee. We thus evaluated four typical protecting groups and found that all produced inferior results in terms of both reactivity and selectivity, emphasising the outstanding performance of the unprotected aniline (Scheme 3c). We next performed a series of experiments where we accessed three different biaryl products, each through both possible combinations of bromide/Bpin partners (Scheme 3d). Only very minor differences in ee outcome were observed within each pair, suggesting that reductive elimination is most likely the major contributor to the final product stereoselectivity.^[^ ^70^ ^]^ This ability to swap partners constitutes a practical advantage as it allows flexibility when determining the most synthetically accessible partner combination for a given biaryl. Finally, we were able to obtain a closely related, enantiopure analogue of our ligand without having to employ preparative SFC separation by utilising the elegant synthesis of enantiopure **Me‐RuPhos** developed by Zhu and coworkers using a chiral auxiliary approach (Scheme 3e).^[^ ^71^ ^]^ After sulfonation, (*R*)‐**Me‐sRuPhos** could deliver similarly outstanding ee values to (*R*)‐**sRuPhos** in formation of **3a**. The extra methyl group proved detrimental to yield but still superior to **sSPhos** (see later discussion). To gain support for the importance of attractive interactions, we evaluated (*S*)**‐sRuPhos‐Np**, in which the sulfonate group has been transformed into a sulfonate ester (Scheme 3f). This sulfonate ester can still act as a hydrogen bond acceptor, but it is far poorer in this regard and the group will engage primarily in repulsive steric interactions.^[^ ^72^, ^73^ ^]^ When evaluated in coupling to form **3a**, (*S*)**‐sRuPhos‐Np** gave only trace product formation, providing support that attractive interactions are crucial to the effectiveness of our sulfonated ligand. To further probe the difference between ligands we compared the reaction profiles using (*R*)**‐sRuPhos, RuPhos,** (*S*)**‐sRuPhos‐Np, sSPhos** and (*R*)‐**Me‐sRuPhos** (Scheme 3g). (*R*)‐**sRuPhos** and **RuPhos** promoted the coupling at a very similar rate (Graph, 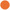 and 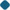), both being far greater than (*S*)**‐sRuPhos‐Np** (Graph, 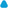), which only afforded trace yield. We interpret this as follows. The Suzuki–Miyaura coupling to form **3a** is fundamentally a challenging one due to the significant hindrance and the presence of the aniline nitrogen. **RuPhos** can accomplish this coupling but if a sterically bulky group (i.e., the sulfonate ester of (*S*)**‐sRuPhos‐Np**) is placed on the lower ring of the ligand this is detrimental and greatly reduces reactivity. If, however, a bulky group is introduced that is proficient at engaging in attractive non‐covalent interactions during the challenging step then this may facilitate the desired process, the stabilising effects of the attractive interactions with an intermediate counteracting any negative impact of extra steric bulk, possibly through a fundamentally different transition state. (*R*)‐**Me‐sRuPhos** possesses a methyl group on the lower ring compared with (*R*)‐**sRuPhos** and proves less reactive, in line with the hypothesis that extra groups on the lower ring reduce reactivity unless attractive interactions can compensate (Graph, 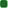). The subtleties of counterbalancing steric effects in these ligands are admittedly complex, since the bulkier *iso*propoxy groups of (*R*)‐**sRuPhos** confer clear acceleration compared with the methoxy groups of (*R*)‐**sSPhos**, the latter among the most poorly reactive ligands (Graph, 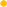). Our present working hypothesis is that the ligand sulfonate group interacts with the phenolic hydroxyl through hydrogen bonding at the transition state for reductive elimination. For the second component, we believe there are two plausible possibilities – one that hydrogen bond donation occurs in a network to the phenolic hydroxyl (Scheme 3h, left) and one in which the associated sodium cation of the ligand interacts with the aniline in a cation–dipole interaction to provide organisation (right). Previous work with **sSPhos** has shown the importance of the associated cation in other contexts and it is feasible it is playing a role here, although further studies will be required to distinguish between the possibilities. Extended screening of bases under the optimised conditions showed no difference in ee between analogous Na and K salts and no reactivity was observed with tetra‐*n*‐butylammonium bases (see Supporting Information).^[^ ^51^, ^52^, ^63^, ^74^ ^]^

**Scheme 3:**
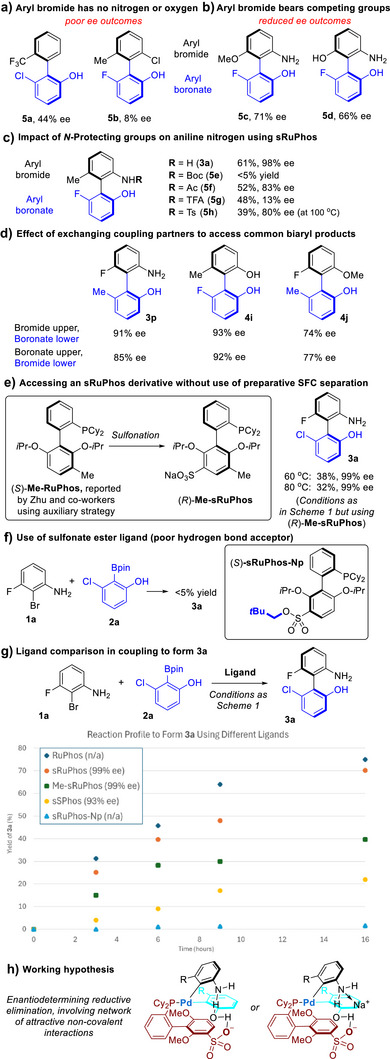
Miscellaneous experiments to probe structural–selectivity relationship of substrates and ligands and working hypothesis relating to interactions at the transition state for reductive elimination.

In summary, we report a new chiral sulfonated phosphine, **sRuPhos**, which can enable Suzuki–Miyaura coupling of unprotected 2‐bromoanilines and *ortho*‐phenolic boronate esters to give axially chiral 2‐amino‐2′‐hydroxybiphenyls with very high enantioselectivity. Furthermore, this new ligand addresses limitations in related couplings to form 2,2′‐biphenols. Evidence is provided for attractive non‐covalent interactions being critical to the reaction outcome. We anticipate that ready access to these motifs will be of importance to medicinal chemists and researchers designing new chiral catalyst scaffolds, and that **sRuPhos** will be of interest more broadly to practitioners of asymmetric palladium catalysis.

## Supporting Information

Further reaction optimisation, procedures, characterisation data are provided in the associated pdf file. CCDC 2418029 contains the supplementary crystallographic data for this paper. This can be obtained free of charge via www.ccdc.cam.ac.uk/data_request/cif or by contacting The Cambridge Crystallographic Data Centre, 12 Union Road, Cambridge, CB2 1EW.

## Conflict of Interests

The authors declare no conflict of interest.

## Supporting information



Supporting Information

## Data Availability

The data that support the findings of this study are available in the supplementary material of this article.

## References

[1] A. Suzuki , Angew. Chem. Int. Ed. 2011, 50, 6722–6737, 10.1002/anie.201101379. 21618370

[2] R. Martin , S. L. Buchwald , Acc. Chem. Res. 2008, 41, 1461–1473, 10.1021/ar800036s. 18620434 PMC2645945

[3] J. Clayden , W. J. Moran , P. J. Edwards , S. R. LaPlante , Angew. Chem. Int. Ed. 2009, 48, 6398–6401, 10.1002/anie.200901719. 19637174

[4] S. R. LaPlante , L. D. Fader , K. R. Fandrick , D. R. Fandrick , O. Hucke , R. Kemper , S. P. F. Miller , P. J. Edwards , J. Med. Chem. 2011, 54, 7005–7022, 10.1021/jm200584g. 21848318

[5] J. E. Smyth , N. M. Butler , P. A. Keller , Nat. Prod. Rep. 2015, 32, 1562–1583, 10.1039/C4NP00121D. 26282828

[6] S. T. Toenjes , J. L. Gustafson , Future Med. Chem. 2018, 10, 409–422, 10.4155/fmc-2017-0152. 29380622 PMC5967358

[7] J. Yin , S. L. Buchwald , J. Am. Chem. Soc. 2000, 122, 12051–12052, 10.1021/ja005622z.

[8] A. N. Cammidge , K. V. L. Crépy , Chem. Commun. 2000, 1723–1724, 10.1039/b004513f.

[9] A. H. Cherney , N. T. Kadunce , S. E. Reisman , Chem. Rev. 2015, 115, 9587–9652, 10.1021/acs.chemrev.5b00162. 26268813 PMC4566132

[10] D. Zhang , Q. Wang , Coord. Chem. Rev. 2015, 286, 1–16, 10.1016/j.ccr.2014.11.011.

[11] P. Loxq , E. Manoury , R. Poli , E. Deydier , A. Labande , Coord. Chem. Rev. 2016, 308, 131–190, 10.1016/j.ccr.2015.07.006.

[12] J. K. Cheng , S.‐H. Xiang , S. Li , L. Ye , B. Tan , Chem. Rev. 2021, 121, 4805–4902, 10.1021/acs.chemrev.0c01306. 33775097

[13] G. Hedouin , S. Hazra , F. Gallou , S. Handa , ACS Catal. 2022, 12, 4918–4937, 10.1021/acscatal.2c00933.

[14] T. A. Schmidt , V. Hutskalova , C. Sparr , Nat. Rev. Chem. 2024, 8, 497–517, 10.1038/s41570-024-00618-x. 38890539

[15] A. N. Cammidge , K. V. L. Crépy , Tetrahedron 2004, 60, 4377–4386, 10.1016/j.tet.2003.11.095.

[16] A. Joncour , A. Décor , J.‐M. Liu , M.‐E. Tran Huu Dau , O. Baudoin , Chem. Eur J. 2007, 13, 5450–5465, 10.1002/chem.200601764. 17352448

[17] A. Bermejo , A. Ros , R. Fernández , J. M. Lassaletta , J. Am. Chem. Soc. 2008, 130, 15798–15799, 10.1021/ja8074693. 18980320

[18] K. Sawai , R. Tatumi , T. Nakahodo , H. Fujihara , Angew. Chem. Int. Ed. 2008, 47, 6917–6919, 10.1002/anie.200802174. 18671314

[19] Y. Uozumi , Y. Matsuura , T. Arakawa , Y. M. A. Yamada , Angew. Chem. Int. Ed. 2009, 48, 2708–2710, 10.1002/anie.200900469. 19283811

[20] X. Shen , G. O. Jones , D. A. Watson , B. Bhayana , S. L. Buchwald , J. Am. Chem. Soc. 2010, 132, 11278–11287, 10.1021/ja104297g. 20698695 PMC2924746

[21] S.‐S. Zhang , Z.‐Q. Wang , M.‐H. Xu , G.‐Q. Lin , Org. Lett. 2010, 12, 5546–5549, 10.1021/ol102521q. 21069984

[22] T. Yamamoto , Y. Akai , Y. Nagata , M. Suginome , Angew. Chem. Int. Ed. 2011, 50, 8844–8847, 10.1002/anie.201103792. 21818832

[23] W. Tang , N. D. Patel , G. Xu , X. Xu , J. Savoie , S. Ma , M.‐H. Hao , S. Keshipeddy , A. G. Capacci , X. Wei , Y. Zhang , J. J. Gao , W. Li , S. Rodriguez , B. Z. Lu , N. K. Yee , C. H. Senanayake , Org. Lett. 2012, 14, 2258–2261, 10.1021/ol300659d. 22497425

[24] S. Wang , J. Li , T. Miao , W. Wu , Q. Li , Y. Zhuang , Z. Zhou , L. Qiu , Org. Lett. 2012, 14, 1966–1969. 22482994 10.1021/ol300721p

[25] L. Benhamou , C. Besnard , E. P. Kündig , Organometallics 2014, 33, 260–266, 10.1021/om4009982.

[26] Z. Wu , C. Wang , L. N. Zakharov , P. R. Blakemore , Synthesis 2014, 46, 1362–1366, 10.1055/s-0033-1341102.

[27] G. Xu , W. Fu , G. Liu , C. H. Senanayake , W. Tang , J. Am. Chem. Soc. 2014, 136, 570–573, 10.1021/ja409669r. 24147559

[28] Y. Zhou , X. Zhang , H. Liang , Z. Cao , X. Zhao , Y. He , S. Wang , J. Pang , Z. Zhou , Z. Ke , L. Qiu , ACS Catal. 2014, 4, 1390–1397, 10.1021/cs500208n.

[29] R. Haraguchi , S. Hoshino , T. Yamazaki , S.‐i. Fukuzawa , Chem. Commun. 2018, 54, 2110–2113, 10.1039/C7CC09960F. 29404546

[30] N. D. Patel , J. D. Sieber , S. Tcyrulnikov , B. J. Simmons , D. Rivalti , K. Duvvuri , Y. Zhang , D. A. Gao , K. R. Fandrick , N. Haddad , K. S. Lao , H. P. R. Mangunuru , S. Biswas , B. Qu , N. Grinberg , S. Pennino , H. Lee , J. J. Song , B. F. Gupton , N. K. Garg , M. C. Kozlowski , C. H. Senanayake , ACS Catal. 2018, 8, 10190–10209, 10.1021/acscatal.8b02509. 30450265 PMC6234982

[31] G. Beutner , R. Carrasquillo , P. Geng , Y. Hsiao , E. C. Huang , J. Janey , K. Katipally , S. Kolotuchin , T. La Porte , A. Lee , P. Lobben , F. Lora‐Gonzalez , B. Mack , B. Mudryk , Y. Qiu , X. Qian , A. Ramirez , T. M. Razler , T. Rosner , Z. Shi , E. Simmons , J. Stevens , J. Wang , C. Wei , S. R. Wisniewski , Y. Zhu , Org. Lett. 2018, 20, 3736–3740, 10.1021/acs.orglett.8b01218. 29909639

[32] D. Shen , Y. Xu , S.‐L. Shi , J. Am. Chem. Soc. 2019, 141, 14938–14945, 10.1021/jacs.9b08578. 31460761

[33] W. Ji , H.‐H. Wu , J. Zhang , ACS Catal. 2020, 10, 1548–1554, 10.1021/acscatal.9b04354.

[34] L. Byrne , C. Sköld , P.‐O. Norrby , R. H. Munday , A. R. Turner , P. D. Smith , Adv. Synth. Catal. 2021, 363, 259–267, 10.1002/adsc.202001211.

[35] K. B. Gan , R.‐L. Zhong , Z.‐W. Zhang , F. Y. Kwong , J. Am. Chem. Soc. 2022, 144, 14864–14873, 10.1021/jacs.2c06240. 35921609

[36] I. K. W. On , W. Hong , Y. Zhu , Tetrahedron Lett. 2023, 119, 154408, 10.1016/j.tetlet.2023.154408.

[37] H.‐Y. Qu , W.‐H. Zheng , Org. Lett. 2023, 25, 9119–9123, 10.1021/acs.orglett.3c03487. 38112557

[38] Y. Yang , C. Wu , J. Xing , X. Dou , J. Am. Chem. Soc. 2024, 146, 6283–6293, 10.1021/jacs.3c14450. 38381856

[39] N. Kamiya , T. Kuroda , Y. Nagata , T. Yamamoto , M. Suginome , J. Am. Chem. Soc. 2025, 147, 8534–8547, 10.1021/jacs.4c16952. 40019936

[40] L. Wu , C. Ge , M. Wang , Z. Shi , J. Am. Chem. Soc. 2025, 147, 20657–20666, 10.1021/jacs.5c03816. 40481785

[41] Z. Zuo , R. S. Kim , D. A. Watson , J. Am. Chem. Soc. 2021, 143, 1328–1333, 10.1021/jacs.0c12843. 33439640 PMC8173523

[42] R. S. Kim , L. O. Kgoadi , J. C. Hayes , D. P. Rainboth , C. M. Mudd , G. P. A. Yap , D. A. Watson , J. Am. Chem. Soc. 2024, 146, 17606–17612, 10.1021/jacs.4c04608. 38780663 PMC11222061

[43] G. Xu , C. H. Senanayake , W. Tang , Acc. Chem. Res. 2019, 52, 1101–1112, 10.1021/acs.accounts.9b00029. 30848882

[44] H. Yang , J. Sun , W. Gu , W. Tang , J. Am. Chem. Soc. 2020, 142, 8036–8043, 10.1021/jacs.0c02686. 32240585

[45] H. Yang , W. Tang , Nat. Commun. 2022, 13, 4577, 10.1038/s41467-022-32360-7. 35931694 PMC9355965

[46] Y. Zhou , S. Wang , W. Wu , Q. Li , Y. He , Y. Zhuang , L. Li , J. Pang , Z. Zhou , L. Qiu , Org. Lett. 2013, 15, 5508–5511, 10.1021/ol402666p. 24138017

[47] I. K. W. On , W. Hong , Y. Zhu , Chem. Catal. 2023, 3, 100523.

[48] R. Pearce‐Higgins , L. N. Hogenhout , P. J. Docherty , D. M. Whalley , P. Chuentragool , N. Lee , N. Y. S. Lam , T. M. McGuire , D. Valette , R. J. Phipps , J. Am. Chem. Soc. 2022, 144, 15026–15032, 10.1021/jacs.2c06529. 35969692 PMC9434994

[49] K. W. Anderson , S. L. Buchwald , Angew. Chem. Int. Ed. 2005, 44, 6173–6177, 10.1002/anie.200502017. 16097019

[50] J. Rodriguez , H. H. Dhanjee , S. L. Buchwald , Org. Lett. 2021, 23, 777–780, 10.1021/acs.orglett.0c04001. 33475382 PMC8057820

[51] M. Kadarauch , D. M. Whalley , R. J. Phipps , J. Am. Chem. Soc. 2023, 145, 25553–25558, 10.1021/jacs.3c10663. 37972383 PMC10690801

[52] M. Kadarauch , T. A. Moss , R. J. Phipps , J. Am. Chem. Soc. 2024, 146, 34970–34978, 10.1021/jacs.4c14754. 39631941 PMC11664591

[53] M. Smrčina , M. Lorenc , V. Hanuš , P. Kočovský , Synlett 1991, 1991, 231–232.

[54] P. Kočovský , Š. Vyskočil , M. Smrčina , Chem. Rev. 2003, 103, 3213–3246. 12914496 10.1021/cr9900230

[55] K. Ding , X. Li , B. Ji , H. Guo , M. Kitamura , Curr. Org. Synth. 2005, 2, 499–545, 10.2174/157017905774322631.

[56] W. Hüttel , M. Müller , Nat. Prod. Rep. 2021, 38, 1011–1043. 33196733 10.1039/d0np00010h

[57] M. Grzybowski , B. Sadowski , H. Butenschön , D. T. Gryko , Angew. Chem. Int. Ed. 2020, 59, 2998–3027, 10.1002/anie.201904934. PMC7027897 31342599

[58] Compound **3a** appears to be a very stable atropisomer ‐ heating a 99% ee sample of **3a** in toluene at 110 °C for 24h gave no loss of ee.

[59] M. L. O'Duill , K. M. Engle , Synthesis 2018, 50, 4699–4714. 31105348 10.1055/s-0037-1611064 PMC6521976

[60] S. L. Gargaro , B. S. Dunson , J. D. Sieber , Synlett 2020, 32, 511–516.

[61] It seems plausible that the presence of two protic functionalities on the reductive elimination precursor complex in the form of the aniline and phenol may exacerbate this undesired process.

[62] Although the steric demand of the substituents on **3a** and **4a** are cumulatively similar (Bott, G.; Field, L. D.; Sternhell, S., Steric effects. A study of a rationally designed system. *J. Am. Chem. Soc*. **1980**, *102*, 5618–5626.), it is possible that presence of the basic amino functionality in **3a** impacts reductive elimination through non‐productive binding.

[63] W. A. Golding , R. Pearce‐Higgins , R. J. Phipps , J. Am. Chem. Soc. 2018, 140, 13570–13574, 10.1021/jacs.8b08686. 30295472

[64] J. E. Milne , S. L. Buchwald , J. Am. Chem. Soc. 2004, 126, 13028–13032, 10.1021/ja0474493. 15469301

[65] The absolute stereochemistry of (*R*)‐sRuPhos was assigned by tenative analogy with (*R*)‐sSPhos, whose absolute configuration is established, with respect to the major enantiomer of biaryl product **3a** obtained.

[66] For extended optimisation including solvent, concentration, equivalents of boronate ester and use of Pd_2_dba_3_ (which gave no conversion under final optimised conditions) see Supporting Information.

[67] S. R. LaPlante , P. J. Edwards , L. D. Fader , A. Jakalian , O. Hucke , ChemMedChem. 2011, 6, 505–513, 10.1002/cmdc.201000485. 21360821

[68] J.‐P. Heeb , J. Clayden , M. D. Smith , R. J. Armstrong , Nat. Protoc. 2023, 18, 2745–2771, 10.1038/s41596-023-00859-y. 37542183

[69] In the original study, slighly modified conditions using sSPhos could give **4a** in 44% yield.

[70] In other systems it has been suggested that oxidative addition and transmetalation can contribute substantially to final stereochemical outcome ‐ see ref. 30.

[71] Y. Lou , J. Wei , M. Li , Y. Zhu , J. Am. Chem. Soc. 2022, 144, 123–129, 10.1021/jacs.1c12345. 34979078 PMC9549467

[72] C. A. Hunter , Angew. Chem. Int. Ed. 2004, 43, 5310–5324, 10.1002/anie.200301739. 15468180

[73] M. C. Storer , C. A. Hunter , Chem. Soc. Rev. 2022, 51, 10064–10082, 10.1039/D2CS00701K. 36412990

[74] W. A. Golding , H. L. Schmitt , R. J. Phipps , J. Am. Chem. Soc. 2020, 142, 21891–21898, 10.1021/jacs.0c11056. 33332114

