# Supplementary material for: Atroposelective Suzuki–Miyaura Coupling to Form 2‐Amino‐2′‐Hydroxybiphenyls Enabled by sRuPhos
Source: Angew Chem Int Ed Engl. 2025 Dec 24;65(8):e20698. doi: 10.1002/anie.202520698 (PMC12910156; doi:10.1002/anie.202520698)

Atroposelective Suzuki-Miyaura Coupling to form 2-Amino-2'-Hydroxybiphenyls Enabled by sRuPhos

Hamzah Sharif,[a] Luke A. McCall,[a] Matthew G. Sanders[b] and Robert J. Phipps*[a]

[a] Hamzah Sharif, Luke A. McCall, and Prof. R. J. Phipps
Yusuf Hamied Department of Chemistry
University of Cambridge
Lensfield Road, Cambridge, CB2 1EW, United Kingdom.
*E-mail: rjp71@cam.ac.uk

[b] Dr. Matthew G. Sanders
Oncology Targeted Discovery, Oncology R&D
The Discovery Centre, Cambridge Biomedical Campus
1 Francis Crick Avenue, Cambridge, CB2 0AA, United Kingdom

Table of Contents

General Information 3

Synthesis of sRuPhos and Me-sRuPhos 4

Resolution of sRuPhos by Preparative SFC and Assignment of Stereochemistry 6

Synthesis of Alkylated Ligand (*S*)-sRuPhos-Np 7

Extended Optimization Details: 9

Synthesis of Starting Materials 11

Synthesis of Products (Scheme 1) 22

Application of *(R)*-sRuPhos to formation of 2,2’-biphenols and comparisons with *(R)*-sSPhos (Scheme 2A) 35

Application of *(R)*-sRuPhos to formation of mono-O-methylated-2,2’-biphenols and comparisons with *(R)*-sSPhos (Scheme 2B) 38

Evaluation of Aryl Bromides with no directing group (Scheme 3A) 41

Aryl Bromide containing a competing directing group (Scheme 3B) 42

Impact of Substitution on aniline nitrogen (Scheme 3C) 44

Effect of swapping coupling partners to access common biaryl products (Scheme 3D) 46

Direct Comparison of Ligands –(Scheme 3G) 49

Effect of Cation on Enantioselectivity 50

Racemization Experiment: (S)-2'-amino-6'-chloro-6-fluoro-[1,1'-biphenyl]-2-ol (3f) 51

Racemization Experiments: (S)-2'-amino-3',5'-dichloro-6-methyl-[1,1'-biphenyl]-2-ol (**3r**) 52

X-Ray Structure of *(S)*- 2'-amino-6'-chloro-6-fluoro-[1,1'-biphenyl]-2-ol (**3f**) 54

Unsuccessful Substrates 55

References 56

General Information

Reagents: All reagents, unless otherwise stated, were used as supplied from commercial sources without further purification. CH_2Cl_2, THF, Et_2O and MeCN was purified by distillation on site under inert atmosphere via the following processes THF and Et_2O were pre-dried over sodium wire then distilled from calcium hydride and lithium aluminium hydride. CH_2Cl_2, MeCN, and toluene were distilled from calcium hydride.

Reaction setup: Reactions were carried out in 4 mL, 15x45mm crimp-top vials, which were purged with nitrogen. In cases where the reactions were heated, the vials were heated in deep welled heating blocks (IKA DB 5.2).

NMR spectra: ^1H NMR spectra were recorded on a 700 MHz TXO Cryoprobe, 600 MHz Bruker Avance DRX-600 spectrometer, 500 MHz Bruker DCH Cryoprobe, 400 MHz Bruker QNP Cryoprobe or 400 MHz Bruker Avance NEO Prodigy N_2 Cryoprobe. Chemical shifts are reported in parts per million (ppm) and the spectra are calibrated to the resonance resulting from incomplete deuteration of the solvent ($CDCl_3$: 7.26 ppm; DMSO-d^6: 2.50 ppm, qn; MeOD-d^4: 3.31 ppm, qn). ^{13}C NMR spectra were recorded on the same spectrometers with complete proton decoupling. Chemical shifts are reported in ppm with the solvent resonance as the internal standard ($CDCl_3$: 77.16 ppm, t; DMSO-d^6: 39.52 ppm, sept; MeOD-d^4: 49.00 ppm, sept). Data are reported as follows: chemical shift δ/ppm, multiplicity (s = singlet, d = doublet, t = triplet, q = quartet, qn = quintet, sept = septet, br = broad, m = multiplet or combinations thereof; ^{13}C, ^{19}F and ^{31}P signals are singlets unless otherwise stated), coupling constants J in Hz, integration (1H only). ^1H-COSY, DEPT-135, HMQC and HMBC were used where appropriate to facilitate structural determination. The carbon atom attached to boron was generally not observed by ^{13}C spectroscopy due to quadrupolar relaxation. ^{19}F and ^{31}P NMR spectra were recorded on a 400 MHz Bruker Avance III HD and 400 MHz Bruker Avance NEO Prodigy N_2 Cryoprobe Spectrometer with complete proton decoupling.

High Resolution Mass Spectrometry (HRMS): Recorded on a Waters Vion IMS QTOF, Q-Exactive Orbitrap, or AGILENT 6230 LC/TOF at the Department of Chemistry at the University of Cambridge. The ionization method is noted as either positive or negative electrospray ionization (+/−ESI). Measured values are reported to 4 decimal places and are within ± 5 ppm of the calculated value. The calculated values are based on the most abundant isotope unless otherwise stated in the chemical formula.

Chromatography: Analytical thin layer chromatography was performed using precoated Merck glass backed silica gel plates (Silicagel 60 F254). Visualisation was by ultraviolet fluorescence (λ = 254 or 365 nm) and/or staining with potassium permanganate ($KMnO_4$). Flash column chromatography was performed using silica gel 60 (0.040-0.063 μm) from Fluorochem or Material Harvest Ltd.

Optical rotation: Measured in $CHCl_3$ or MeOH on a Perkin Elmer 343 Polarimeter using a sodium lamp (λ 589 nm, D-line). Values are reported at a given temperature (°C) in degrees.$cm^2.g^{-1}$ with concentration in g/100mL.

Chiral SFC analysis: Performed on a Waters ACQUITY Performed on a Waters ACQUITY UPC2 System with DAICEL CHIRALPAK IA, IC, IE, IG, IH, IJ or IK columns (4.6 × 250 mm, 3.0 μm) in a mixed solvent system of supercritical CO_2 and MeOH, or a YMC CHIRALART SB column (4.6 x 250 mm, 3 μm) in a mixed system of CO_2 and MeOH. A system backpressure of 138 bar was used.

Chiral HPLC analysis: Performed on a Waters ARC system with CHIRALPAK YMC IG or CHIRALART SC or SB columns (4.6 x 250 mm, 3 μm) in a mixed solvent system of n-hexane and iPrOH.

X-ray crystallography: Data collection and analysis was performed by Dr. Andrew Bond (University of Chemistry).

Racemic Reactions: Reactions to obtain racemic SFC or HPLC traces were run with RuPhos.

Synthesis of sRuPhos and Me-sRuPhos

Under the sulfonation conditions previously used for sSPhos, we observed that the *iso*-propyl groups of RuPhos were removed competitively with the desired arene sulfonation. Therefore, a milder sulfonation method using lower temperature and short reaction time was required. For this purpose of combination of TFAA and sulfuric acid was utilised, referred to below as "activated sulfuric acid". This combination that has been previously used for other sulfonations.[1]

Activated sulfuric acid: Trifluoroacetic anhydride (1.84 mL) and sulfuric acid (0.36 mL) were combined and stirred for 16 h, to form a solution, which was used for the following ligands below.

Sodium (*rac*)-2'-(dicyclohexylphosphaneyl)-2,6-diisopropoxy-[1,1'-biphenyl]-3-sulfonate (sRuPhos)

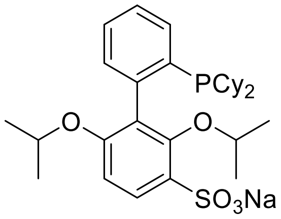

Activated sulfuric acid solution (1.9 mL, as prepared above) was added dropwise to a solution of RuPhos (2.33 g, 5 mmol) in trifluoroacetic acid (3 mL) at 0 °C . The solution was stirred at 0 °C for 3 h, then quenched with ice cold water followed by NaOH until the solution was basic, pH 9-11. The mixture was further diluted by water (20 mL) and extracted with DCM (30 mL x 3). The product was purified by column chromatography (1-10% MeOH:DCM) to yield the product as a white solid (1.79 g, 3.15 mmol, 63%). **^{1}H NMR** (500 MHz, MeOD) δ 7.91 (d, *J* = 8.8 Hz, 1H), 7.79 – 7.50 (m, 1H), 7.35 (dd, *J* = 5.7, 3.3 Hz, 2H), 7.25 – 7.13 (m, 1H), 6.73 (d, *J* = 8.9 Hz, 1H), 4.55 (p, *J* = 6.0 Hz, 1H), 4.06 – 3.80 (m, 1H), 2.06 (s, 1H), 1.83 (dd, *J* = 30.3, 11.8 Hz, 2H), 1.74 – 1.48 (m, 9H), 1.39 – 0.73 (m, 22H). **^{31}P NMR** (203 MHz, MeOD) δ -9.54. **^{13}C NMR** (126 MHz, MeOD) δ 158.0, 153.6, 143.2 (d, *J* = 31.7 Hz), 136.4 (d, *J* = 18.6 Hz), 132.0, 131.9, 130.2, 128.8, 127.3, 126.8 (d, *J* = 6.7 Hz), 126.0, 105.7, 73.4, 69.8, 35.2, 35.1, 33.1, 33.0, 30.1 (d, *J* = 14.3 Hz), 29.7 (t, *J* = 16.6 Hz), 29.3 (d, *J* = 8.7 Hz), 27.1 (d, *J* = 8.1 Hz), 27.1 (d, *J* = 4.6 Hz), 26.9 (d, *J* = 4.6 Hz), 26.8 (d, *J* = 4.4 Hz), 26.2, 21.7, 21.3, 20.8, 20.7. **HRMS** $[M\text{-}Na]^-$ calcd for $[C_{30}H_{42}O_5PS]^-$ 545.2496, found 545.2490, Δ= -1.1 ppm.

Sodium (R)-2'-(dicyclohexylphosphaneyl)-2,6-diisopropoxy-5-methyl-[1,1'-biphenyl]-3-sulfonate ((*R*)-Me-sRuPhos)

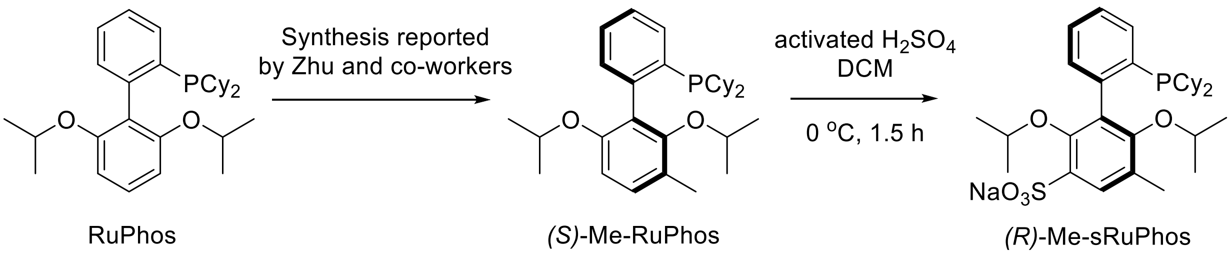

Both enantiomers of dicyclohexyl(2',6'-diisopropoxy-3'-methyl-[1,1'-biphenyl]-2-yl)phosphane), Me-RuPhos, were prepared following the reported procedure by Zhu et al.[2] The *S* enantiomer of Me-RuPhos was then taken forward and converted to *(R)*-Me-sRuPhos shown above. *Please note that the priority of the lower ring changes upon sulfonation, for the purposes of assigning stereochemical descriptor.*

Activated sulfuric acid (prepared as above, 0.15 mL) was added dropwise to a solution of *(S)*-Me-RuPhos (220 mg, 0.46 mmol), in DCM (0.28 mL) at 0 °C. The solution was stirred at 0 °C for 1.5 h, then quenched with ice cold water followed by NaOH until the solution was basic, pH 9-11. The mixture was further diluted by water (20 mL) and extracted with DCM (30 mL x 3). The product was purified by column chromatography (1-10% MeOH:DCM) to yield the product, (*R*)-Me-sRuPhos, as a white solid (40.2 mg, 0.069 mmol, 15%). ^{1}**H NMR** (500 MHz, MeOD) δ 7.81 (s, 1H), 7.68 (m, 1H), 7.40 (d, 3H), 4.19 – 3.98 (m, 1H), 3.88 – 3.72 (m, 1H), 2.28 (s, 3H), 2.23 – 2.10 (m, 2H), 2.08-1,96 (m, 2H), 1.85 – 1.75 (m, 2H), 1.75 – 1.50 (m, 8H), 1.39 – 1.06 (m, 8H), 1.03 (d, *J* = 6.1 Hz, 3H), 0.91 (t, *J* = 6.2, 6H), 0.82 (d, *J* = 6.1 Hz, 3H). ^{13}**C NMR** (126 MHz, MeOD) δ 156.4 (d, *J* = 1.7 Hz), 151.6, 143.0, 142.8, 133.1, 132.3, 132.2, 131.8 (d, *J* = 3.9 Hz), 129.9, 127.5, 126.6, 125.0, 74.6, 74.4, 33.6 (dd, *J* = 32.3, 14.4 Hz), 32.1 (d, *J* = 20.3 Hz), 31.2 (d, *J* = 18.0 Hz), 29.2 (d, *J* = 12.4 Hz), 28.4 (d, *J* = 8.6 Hz), 27.5 – 27.2 (m), 27.1, 27.1, 27.0, 26.9 (d, *J* = 8.8 Hz), 26.2, 21.9, 21.6, 21.5, 21.4, 17.00, 15.9. ^{31}**P NMR** (203 MHz, MeOD) δ -9.04. **HRMS** $[M\text{-}Na]^-$ calcd for $[C_{31}H_{44}O_5PS]^-$ 559.2653, found 559.2646, Δ= -1.3 ppm. $[\alpha]_D^{25}$ = -26.5 (c 1.10, $CHCl_3$).

Note – the low yield here was due to competing loss of iPr groups, which predominated if high conversion to the sulfonated product was attempted by longer reaction times or higher temperatures.

Resolution of sRuPhos by Preparative SFC and Assignment of Stereochemistry

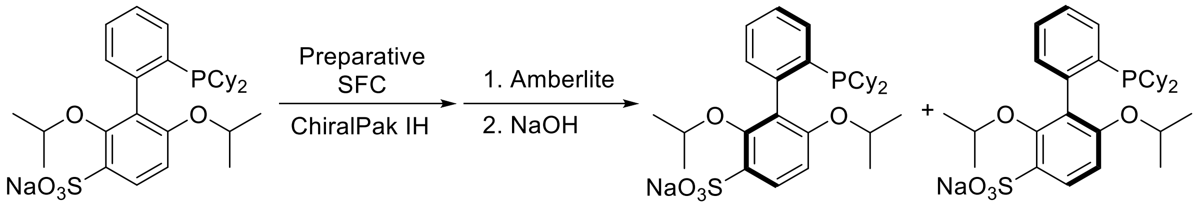

(rac)-sRuPhos *(S)*-sRuPhos *(R)*-sRuPhos

(rac)-sRuPhos was separated at AstraZeneca, Cambridge, UK by preparatory SFC (Waters SFC Prep 100 System). *(rac)*-sRuPhos (7 g) was dissolved in CH_2Cl_2 (30 mL) and MeOH (50 mL), but due to poor solubility the solution was centrifuged. The resulting particulate free solution was then purified by preparative SFC (Column: CHIRAL PAK IH, 20 x 250 mm, 5 micron, Mobile phase: 25 % [MeOH + 0.1% NH_3]: 75% CO_2, Flow rate: 60 mL/min, Back pressure regulator (BPR): 120 bar, Column temperature: 40 °C). Two fractions were isolated: 1) An earlier fraction which was later determined (mentioned below) to be *(R)*-sRuPhos (2.2 - 3.6 minutes) and 2) a later fraction that was subsequently determined to be *(S)*-sRuPhos (10.6 - 15.8 minutes). The solvent was removed to yield off-white solids. A portion of the material (500 mg) corresponding to *(R)*-sRuPhos (2.2 - 3.6 minutes fraction) was dissolved in MeOH (100 mL) and passed through a column of Amberlite IRC120 H, hydrogen form (washed first with MeOH until run clear) five times, then the solvent was removed to yield the protonated, zwitterionic *(R)*-sRuPhos. This residue was dissolved in CH_2Cl_2 (500 mL) and cooled in ice. NaOH (2.5 M, 200 mL) and H_2O (300 mL) were added, and the layers separated. The aqueous layer was washed with CH_2Cl_2 (400 mL), the organic layers combined, dried ($MgSO_4$) and solvent removed (keeping the flask below 25 °C to reduce potential phosphine oxidation) to give *(R)*-sRuPhos as an off-white solid (475 mg, 0.83 mmol). **^{1}H NMR** (500 MHz, MeOD) δ 7.91 (d, *J* = 8.8 Hz, 1H), 7.79 – 7.50 (m, 1H), 7.35 (dd, *J* = 5.7, 3.3 Hz, 2H), 7.25 – 7.13 (m, 1H), 6.73 (d, *J* = 8.9 Hz, 1H), 4.55 (p, *J* = 6.0 Hz, 1H), 4.06 – 3.80 (m, 1H), 2.06 (s, 1H), 1.83 (dd, *J* = 30.3, 11.8 Hz, 2H), 1.74 – 1.48 (m, 9H), 1.39 – 0.73 (m, 22H). **^{31}P NMR** (203 MHz, MeOD) δ -9.54. **^{13}C NMR** (126 MHz, MeOD) δ 158.0, 153.6, 143.2 (d, *J* = 31.7 Hz), 136.4 (d, *J* = 18.6 Hz), 132.0, 131.9, 130.2, 128.8, 127.3, 126.8 (d, *J* = 6.7 Hz), 126.0, 105.7, 73.4, 69.8, 35.2, 35.1, 33.1, 33.0, 30.1 (d, *J* = 14.3 Hz), 29.7 (t, *J* = 16.6 Hz), 29.3 (d, *J* = 8.7 Hz), 27.1 (d, *J* = 8.1 Hz), 27.1 (d, *J* = 4.6 Hz), 26.9 (d, *J* = 4.6 Hz), 26.8 (d, *J* = 4.4 Hz), 26.2, 21.7, 21.3, 20.8, 20.7. **HRMS** $[M-Na]^-$ calcd for $[C_{30}H_{42}O_5PS]^-$ 545.2496, found 545.2490, Δ= -1.1 ppm. $[\alpha]_D^{25}$ = - 61.0 (c 1.20, MeOH).

The ligand batch corresponding to the first fraction (2.2 - 3.6 minutes), was assigned as being *(R)*-sRuPhos, by comparing the major enantiomer obtained of the Suzuki-Miyaura product **3a** with that using ligands of similar structure and known stereochemistry, *(R)*-sSPhos and *(R)*-Me-sRuPhos. All three ligands gave the same major enantiomer of **3a**, shown by SFC analysis, supporting the assignment that the first fraction separated by preparative SFC to be *(R)*-sRuPhos.

The same method was repeated but with the later fraction to yield *(S)*-sRuPhos (460 mg, 0.80 mmol). All characterisation data matched that for *(R)*-sRuPhos, except optical rotation. $[\alpha]_D^{25}$ = + 54.9 (c 0.25, MeOH). The enantiopurity of *(S)*-sRuPhos was later verified by SFC to be >99% ee, when the ligand was converted to *(S)*-sRuPhos-Np (see later).

Synthesis of Alkylated Ligand (*S*)-sRuPhos-Np

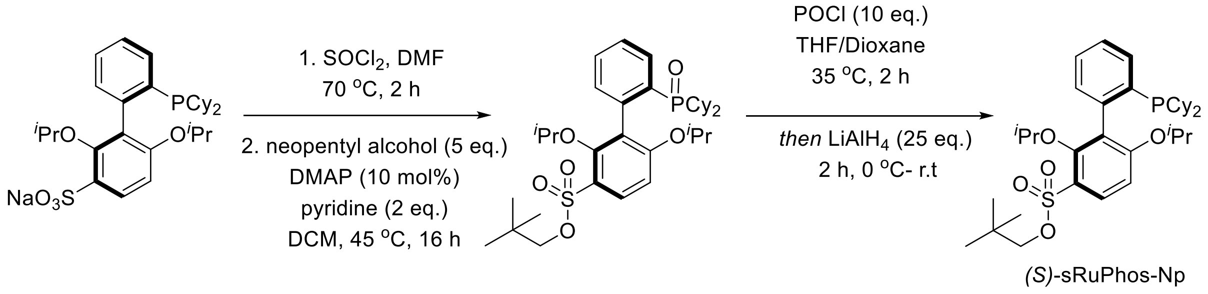

Neopentyl (S)-2'-(dicyclohexylphosphoryl)-2,6-diisopropoxy-[1,1'-biphenyl]-3-sulfonate

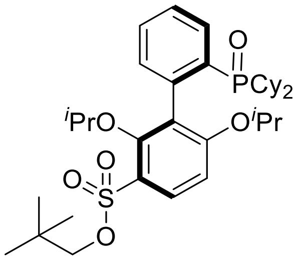

Step 1: A 20 mL crimped top microwave vial was charged with (S)-Na-sRuPhos (284 mg, 0.500 mmol, 1.00 equiv.) and subjected to three nitrogen/vacuum cycles. Thionyl chloride (5 mL) was added at room temperature under a nitrogen atmosphere, followed by anhydrous DMF (0.5 mL). The solution was stirred at 70 °C for 2 h. Upon completion, the excess thionyl chloride was removed under a stream of air. The crude reaction mixture was cooled to 0 °C and diluted with EtOAc (5 mL), and excess thionyl chloride was quenched by the dropwise addition of water (5 mL). The mixture was extracted with EtOAc (3×). The combined organic extracts were dried over MgSO4, filtered, and the solvent removed under reduced pressure. The crude sulfonyl chloride product was dried under high vacuum for 1 hour and subjected to the next step without further purification.

Step 2: A 5 mL crimped top microwave vial was charged with neopentyl alcohol (220 mg, 2.50 mmol, 5.00 equiv.) and DMAP (6 mg, 0.05 mmol, 10 mol%), and was subjected to three nitrogen/vacuum cycles. Crude sulfonyl chloride was dissolved in DCM (2.5 mL) and added under a nitrogen atmosphere, followed by anhydrous pyridine (AcroSeal Extra Dry) (79 mg, 81 µL, 1.0 mmol, 2.0 equiv.). The reaction was heated to 45 °C and stirred for 16 hours. The solvent was removed under a stream of air and the crude reaction mixture purified by flash column chromatography (EtOAc:Pet 20%-100%), to afford the title compound (164 mg, 0.265 mmol, 53%).

^{1}H NMR (700 MHz, $CDCl_3$) δ 7.92 (d, *J* = 9.0 Hz, 1H), 7.56 – 7.43 (m, 3H), 7.34 – 7.29 (m, 1H), 6.71 (d, *J* = 9.1 Hz, 1H), 4.58 (p, *J* = 6.0 Hz, 1H), 4.01 (d, *J* = 9.3 Hz, 1H), 3.88 (d, *J* = 9.2 Hz, 1H), 3.73 (p, *J* = 6.1 Hz, 1H), 2.14 – 1.47 (m, 18H), 1.34 – 0.97 (m, 25H). **^{13}C NMR** (176 MHz, $CDCl_3$) δ 171.2, 159.8, 155.1, 140.3, 133.6 (d, *J* = 9.2 Hz), 131.8, 130.4 (d, *J* = 12.4 Hz), 130.0 (d, *J* = 3.3 Hz), 126.8 (d, *J* = 11.7 Hz), 125.9 (d, *J* = 3.3 Hz), 121.9, 106.4, 79.7, 74.3, 70.4, 60.4, 38.5 (d, *J* = 65.9 Hz), 36.4 (d, *J* = 67.1 Hz), 31.8, 26.7, 26.6 (d, *J* = 1.8 Hz), 26.5

(d, J= 4.2 Hz), 26.5, 26.4, 26.3 (d, J= 6.2 Hz), 26.3 (d, J= 3.6 Hz), 25.9 (d, J= 1.8 Hz), 25.8, 25.4 (d, J= 3.8 Hz), 25.2 (d, J= 3.4 Hz), 22.6, 22.0, 21.7, 21.2, 14.2. ^{31}**P NMR** (162 MHz, $CDCl_3$) δ 46.94 (br s). **HRMS** $[M+H]^+$ calcd for $[C_{35}H_{54}O_6PS]^+$; 633.3373, found 633.3379, Δ= +0.9 ppm. $[\alpha]_D^{25}$ = - 14.7 (c 0.26, $CHCl_3$).

Neopentyl (S)-2'-(dicyclohexylphosphaneyl)-2,6-diisopropoxy-[1,1'-biphenyl]-3-sulfonate (*(S)*-sRuPhos-Np)

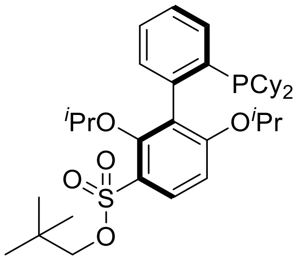

A 20 mL crimped top microwave vial was charged with Neopentyl (S)-2'-(dicyclohexylphosphoryl)-2,6-diisopropoxy-[1,1'-biphenyl]-3-sulfonate (164 mg, 0.265 mmol) and subjected to three nitrogen/vacuum cycles. THF (3 mL) and 1,4-dioxane (0.70 mL) were added under nitrogen, followed the dropwise addition of phosphoryl chloride (383 mg, 234 µL, 2.50 mmol, 10.0 equiv.). The reaction was stirred at 35 ℃ for 2 hours. The reaction mixture was then cooled to 0 ℃ and $LiAlH_4$ (2.00 M in THF, 3.12 mL, 6.25 mmol, 25.0 equiv.) was added dropwise (caution: exotherm). The reaction was stirred at room temperature for 2 hours. Upon completion, the reaction was quenched with the slow addition of EtOAc (2 mL), methanol (2 mL) and water (2 mL). Additional water (20 mL) was added to the quenched reaction mixture. The aqueous phase was extracted with chloroform (3×). The combined organic extracts were dried over $MgSO_4$, filtered, and the solvent removed under reduced pressure on a rotary evaporator (keeping the water bath at room temperature). The crude product was purified by flash column chromatography (50% EtOAc in petroleum ether) to afford the title compound as a solid (94.1 mg, 0.153 mmol, 58%, > 99% ee). ^{1}**H NMR** (400 MHz, $CDCl_3$) δ 7.96 (d, J= 8.9 Hz, 1H), 7.43 (br s, 2H), 7.28 (br s, 2H), 6.71 (d, J= 9.0 Hz, 1H), 4.57 (q, J= 6.0 Hz, 1H), 3.93 (d, J= 9.0 Hz, 1H), 3.85 (d, J= 9.0 Hz, 1H), 3.59 (br s, 1H), 1.99 – 1.48 (m, 13H), 1.37 – 0.65 (m, 30H). ^{31}**P NMR** (162 MHz, $CDCl_3$) δ -9.51 (br s). ^{13}**C NMR** (176 MHz, C_6D_6) δ 160.3, 154.8 (d, J= 1.8 Hz), 142.1 (d, J= 31.9 Hz), 136.4 (d, J= 22.0 Hz), 132.4 (d, J= 3.5 Hz), 131.8, 131.8 (d, J= 5.9 Hz), 126.8, 126.3 (d, J= 6.4 Hz), 123.4, 105.9, 79.4 (d, J= 4.5 Hz), 74.2, 69.8, 35.4 (d, J= 16.9 Hz), 32.5 (d, J= 14.6 Hz), 31.7, 31.6, 30.0 (d, J= 3.9 Hz), 30.0, 30.0, 30.0, 28.7 (d, J= 6.1 Hz), 27.6 (d, J= 12.1 Hz), 27.5 (d, J= 6.3 Hz), 27.1 (d, J= 11.1 Hz), 27.0 (d, J= 10.1 Hz), 26.5 (d, J= 28.8 Hz), 26.3, 22.7, 22.4, 21.6, 21.3, 21.0. **Chiral HPLC Analysis** (SC-3, 80:20 Hexane : ^{i}PrOH, 1.25 mL/min, 4.26 min [major], 6.69 min [minor]). **HRMS** $[M+H]^+$ calcd for $[C_{35}H_{54}O_5PS]^+$; 617.3424, found 617.3430, Δ= +1.0 ppm. $[\alpha]_D^{25}$ = + 11.3 (c 0.14, $CHCl_3$).

Extended Optimization Details:

Concentration and Equivalence screen of **3a**:

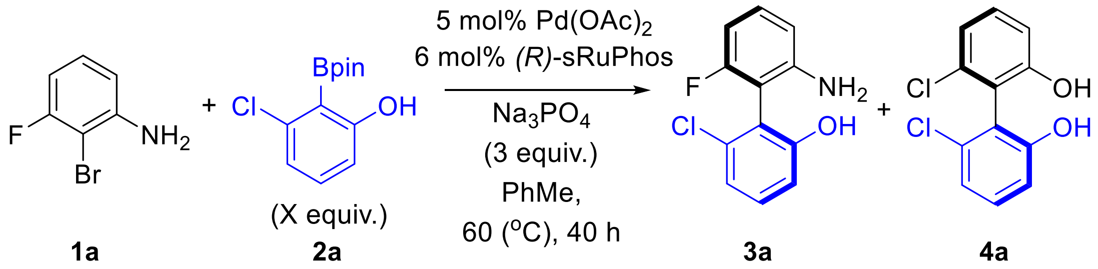

Entry	**2a** (X equiv.)	PhMe Volume	Yield **3a**[a]	Yield **4a**[a]
1	3	0.5 mL	70%	30%
2	3	0.25 mL	56%	43%
3	2	0.5 mL	60%	35%
4	2	0.25 mL	40%	56%

[a]Yields determined by ^{1}H NMR with reference to internal standard. [b]ee determined by SFC analysis of the crude reaction mixture

Solvent screen of **3f**:

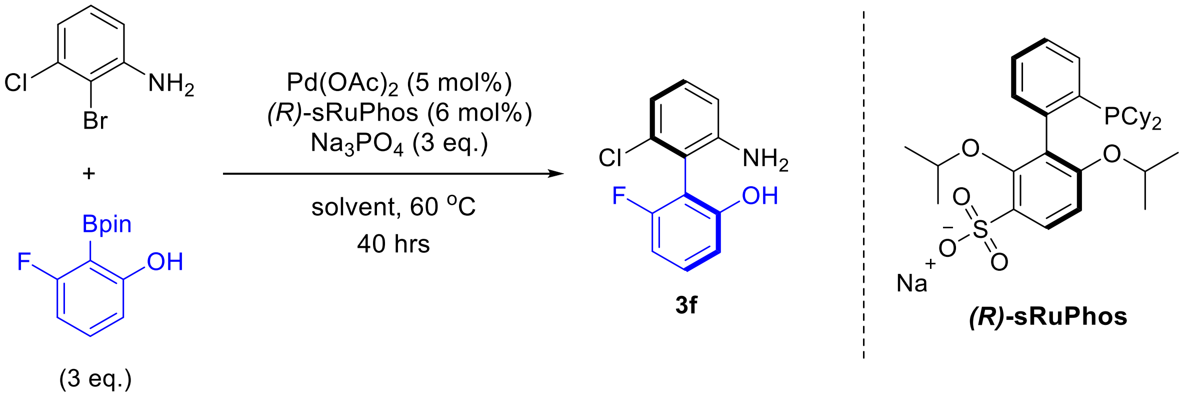

Entry	Solvent	Yield **3f**[a] /%	%ee **3f**[b] /%
1	PhMe:H_2O (19:1)	55	99
2	PhMe	(70)	(99)
3	$PhCF_3$	67	99
4	DCE	76	97
5	THF	79	97
6	MeOH	9	89

[a]Yields determined by ^{1}H NMR with reference to internal standard. Values in parentheses refer to isolated yield and ee. [b]ee determined by SFC analysis of the crude reaction mixture

Evaluation of $Pd_2(dba)_3$ in place of $Pd(OAc)_2$:

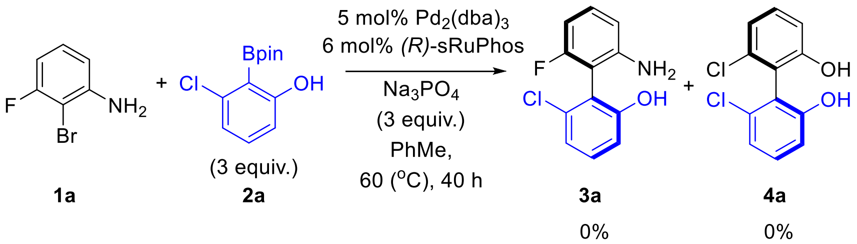

1a 2a 3a 4a

0% 0%

Evaluation of reaction in absence of aryl bromide to assess whether homocoupling to form **4a** occurs:

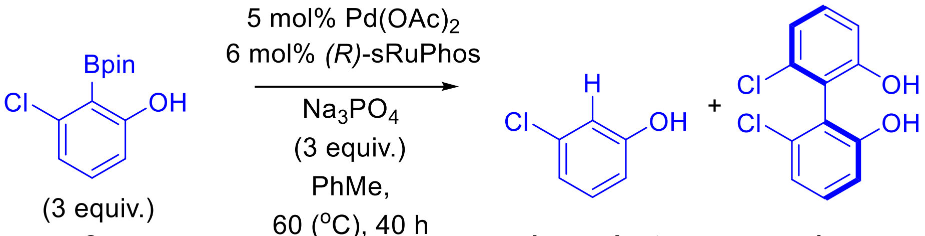

2a **byproduct** **4a**

100% 0%

Synthesis of Starting Materials

General procedure A: demethylation and boron esterification of methoxyphenylboronic acids

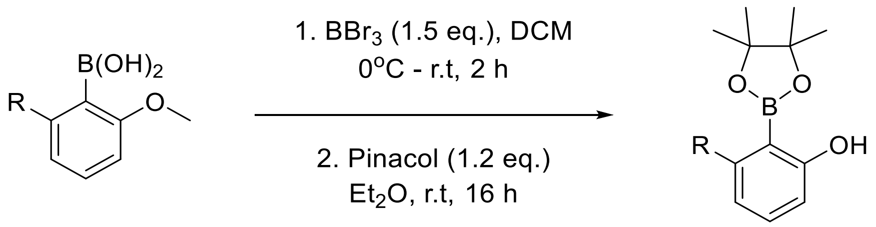

Boronic acid (5 mmol) was dissolved in CH_2Cl_2 (20 mL), then cooled to 0 °C. BBr_3 (1M in CH_2Cl_2, 7.5 mL, 1.5 eq.) was added dropwise, and allowed to warm to rt for 2 h. Ice-cold water (5 mL) was added, then EtOAc (30 mL) was added and the organic layer extracted. The aqueous layer was extracted a further two times with EtOAc (20 mL), the organic layers combined, dried ($MgSO_4$), and solvent removed under reduced pressure. To the crude residue, pinacol (710 mg, 6 mmol, 1.2 eq.) was added, then Et_2O (20 mL) was added and the resulting solution stirred at room temperature for 16 h. After this time, the solvent was removed under reduced pressure and the product purified by flash column chromatography (5-25% EtOAc:petrol) to yield the product.

General procedure B: Borylation and Pinacol Protection of o-bromophenols[3]

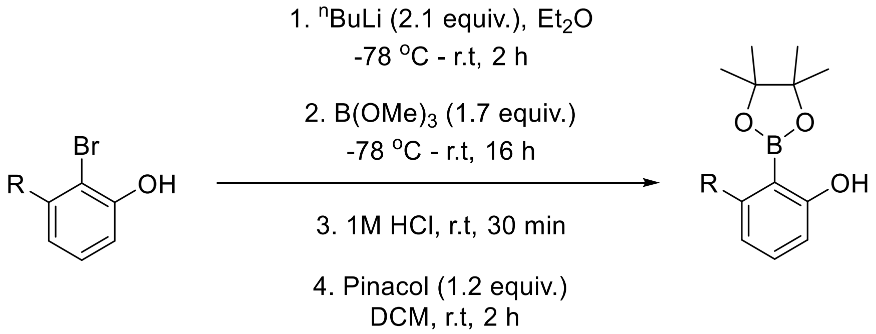

Bromophenol (1 equiv.) and dry ether (0.15 M) were charged to an oven-dried Schlenk flask which was placed under nitrogen and cooled to -78 °C. A solution of n-BuLi in hexanes (2.1 equiv.) was added dropwise. The mixture was then allowed to warm to 25 °C over 2 hours. The flask was again cooled to -78 °C and trimethyl borate (1.7 equiv.) was rapidly added. The mixture was allowed to warm to 25 °C and stirred for 15 hours. Aqueous 1 M HCl (0.15 M) was then slowly added into the reaction mixture and stirred for 30 mins. The layers were separated, and the aqueous layer was extracted with ether (3 x 40 mL). The combined organic layers were concentrated under reduced pressure to give the boronic acid product as a white to yellow powder, which was taken immediately to the esterification step to avoid degradation. A mixture of boronic acid and pinacol (1.2 equiv.) in DCM (0.24 M) was stirred at 25 °C temperature for 2 hours. The resulting solution was diluted with water (20 mL) and then extracted with DCM (3 x 20 mL). The combined organic layers were dried over $MgSO_4$ and concentrated under reduced pressure and the product purified by flash column chromatography (5-25% EtOAc:petrol) to yield the product.

General Procedure C: Chlorination of 2-bromoanilines

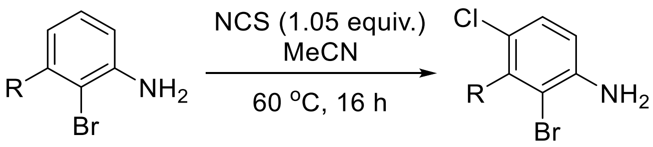

A mixture of 3-substituted-2-bromoaniline (1 equiv.) and NCS (1.05 equiv.) were dissolved in CH_3CN (0.125M) and the mixture heated to 60°C for 16 h. After this time the solution was allowed to cool to room temperature and the solvent removed *in vacuo*. The crude mixture was then purified by column chromatography (0-10% EtOAc:petrol) to give the title compound.

General procedure D: alkylation of Anilines

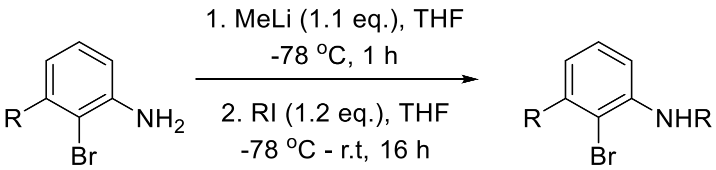

Following a procedure by Probst N. et al.[4] To a stirred solution of aniline derivative in THF (0.5 M) was added MeLi (1.5 M in Et_2O, 1.1 equiv) at –78 °C under nitrogen. After stirring for 1 h at this temperature, MeI or EtI (1.2 equiv.) in THF was added dropwise to the mixture. The mixture was gradually warmed to room temperature and stirred overnight at this temperature. The reaction mixture was quenched with aqueous saturated NH_4Cl and extracted with EtOAc twice. The combined organic layer was washed with brine, dried over $MgSO_4$, and concentrated in vacuum. The residue was purified by flash column chromatography (0-10% EtOAc:petrol) .

General procedure E: benzylation of Anilines

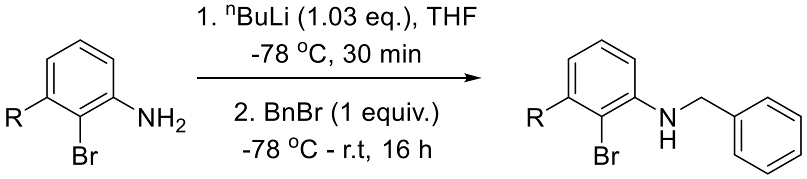

Following a procedure by cat[5] A solution of 2-bromoaniline in tetrahydrofuran (150 ml) was cooled to -78°C. n-Butyllithium in hexane (1.6 M, 1.03 equiv.) was slowly added dropwise and the solution was stirred at -78 °C for 30 minutes. Benzylbromide (1 equiv.) was added slowly via a syringe and the reaction mixture was allowed to warm slowly to room temperature overnight. Water (10 ml) was added to quench the reaction and the aqueous phase was extracted with ethyl acetate (5 x 30 ml). The combined organic extracts were washed with a saturated solution of sodium carbonate (4 x 100 ml) and water (100 ml). The solution was dried over magnesium sulphate, filtered through celite, and the solvents were removed on a rotary evaporator. The product was purified by flash chromatography (0-5% EtOAc:petrol).

<u>3-fluoro-2-(4,4,5,5-tetramethyl-1,3,2-dioxaborolan-2-yl)phenol</u>

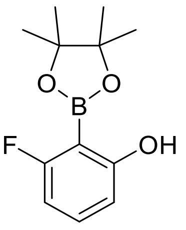

Synthesised according to general procedure **A** with 2-fluoro-6-methoxyphenylboronic acid (849 mg, 5 mmol) to afford the title compound as a white/grey solid (952 mg, 4.00 mmol, 80%). ^{1}**H NMR** (400 MHz, $CDCl_3$) δ 8.38 (s, 1H), 7.33 (q, J = 7.1 Hz, 1H), 6.70 (d, J = 8.2 Hz, 1H), 6.58 (t, J = 8.5 Hz, 1H), 1.41 (s, 12H). ^{13}**C NMR** (101 MHz, $CDCl_3$) δ 167.8 (d, *J* = 251.5 Hz), 164.9 (d, *J* = 10.4 Hz), 134.5 (d, *J* = 11.5 Hz), 111.6 (d, *J* = 3.2 Hz), 106.4 (d, *J* = 23.9 Hz), 84.5, 24.8. ^{19}**F NMR** (376 MHz, $CDCl_3$) δ -100.87.

Data in accordance with literature.[6]

3-chloro-2-(4,4,5,5-tetramethyl-1,3,2-dioxaborolan-2-yl)phenol

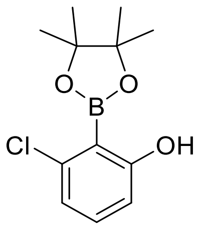

Synthesised according to general procedure **A** with 2-chloro-6-methoxyphenylboronic acid (930 mg, 5 mmol) to afford the title compound as a pale-yellow oil (979 mg, 3.85 mmol, 77%). ^{1}**H NMR** (400 MHz, $CDCl_3$) δ 8.64 (s, 1H), 7.25 (t, J = 8.1 Hz, 1H), 6.92 (d, J = 7.9 Hz, 1H), 6.80 (d, J = 8.3 Hz, 1H), 1.42 (s, 12H). ^{13}**C NMR** (101 MHz, $CDCl_3$) δ 165.2, 141.0, 133.5, 121.5, 114.5, 84.5, 24.8.

Data accordance with literature.[6]

3,4-difluoro-2-(4,4,5,5-tetramethyl-1,3,2-dioxaborolan-2-yl)phenol

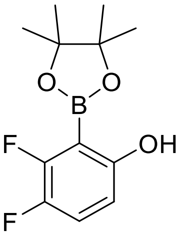

Synthesised according to general procedure **A** with 2,3-difluoro-6-methoxyphenylboronic acid (939 mg, 5 mmol) to afford the title compound as a white solid (768 mg, 3.00 mmol, 60%). ^{1}**H NMR** (700 MHz, $CDCl_3$) δ 8.09 (s, 1H), 7.14 (q, J = 9.1 Hz, 1H), 6.59 (ddd, J = 9.1 Hz, J= 3.2 Hz , J= 1.7 Hz ,1H), 1.39 (s, 12H). ^{13}**C NMR** (176 MHz, $CDCl_3$) δ. 159.5 (d, *J* = 10.6 Hz), 154.0 (dd, *J* = 253.0, 12.8 Hz), 144.1 (dd, *J* = 239.1, 14.3 Hz), 121.6 (dd, *J* = 18.7, 2.8 Hz), 111.2 (dd, *J* = 5.5, 3.9 Hz), 84.8, 24.8. ^{19}**F NMR** (376 MHz, $CDCl_3$) δ -126.24 (d, J = 21.9 Hz), -150.18 (d, J = 21.9 Hz). **HRMS** $[M-H]^-$ calcd for $[C_{12}H_{14}BF_2O_3]^-$ 255.1012, found 255.1011, Δ= -0.2 ppm.

3-methyl-2-(4,4,5,5-tetramethyl-1,3,2-dioxaborolan-2-yl)phenol

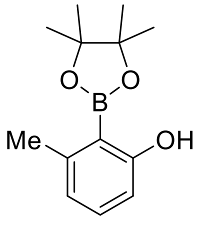

Synthesised according to general procedure **B** with 2-bromo-3-methylphenol (935 mg, 5 mmol) to afford the title compound as a white solid (468 mg, 2.00 mmol, 40%). ^{1}**H NMR** (700 MHz, $CDCl_3$) δ 8.55 (s, 1H), 7.24 (dd, *J* = 8.2, 7.4 Hz, 1H), 6.73 (dt, *J* = 8.3, 0.9 Hz, 1H), 6.71 (dt, *J* = 7.4, 0.8 Hz, 1H), 2.52 (s, 3H), 1.39 (s, 12H). ^{13}**C NMR** (176 MHz, $CDCl_3$) δ 164.9, 147.1, 132.9, 121.6, 113.1, 83.8, 24.8, 22.8.

Data in accordance with literature.[6]

1-(4,4,5,5-tetramethyl-1,3,2-dioxaborolan-2-yl)-5,6,7,8-tetrahydronaphthalen-2-ol

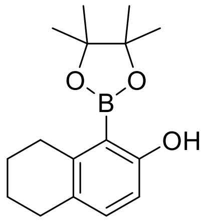

Synthesised according to general procedure **B** with 1-bromo-5,6,7,8-tetrahydronaphthalen-2-ol (1.12 g, 5 mmol) to afford the title compound as a white solid (466 mg, 1.70 mmol, 34%). ^{1}**H NMR** (700 MHz, $CDCl_3$) δ 8.56 (s, 1H), 7.08 (d, *J* = 8.4 Hz, 1H), 6.70 (d, *J* = 8.4 Hz, 1H), 3.03 (t, *J* = 6.3 Hz, 2H), 2.72 (t, *J* = 6.3 Hz, 2H), 1.94 – 1.70 (m, 4H), 1.40 (s, 12H). ^{13}**C NMR** (176 MHz, $CDCl_3$) δ 163.1, 145.0, 134.5, 128.0, 113.4, 83.6, 29.8, 29.6, 24.8, 23.5, 22.9. **HRMS** $[M-H]^-$ calcd for $[C_{16}H_{22}BO_3]^-$ 273.1670, found 273.1666, Δ= -1.6 ppm.

2-(2-fluoro-6-methoxyphenyl)-4,4,5,5-tetramethyl-1,3,2-dioxaborolane

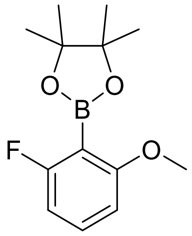

Pinacol (710 mg, 6 mmol, 1.2 eq.) was added to a solution of (2-fluoro-6-methoxyphenyl)boronic acid (850 mg, 5.00 mmol) and Et_2O (20 mL) was added and the resulting solution stirred at room temperature for 16 h. After this time, the solvent was removed under reduced pressure and the product purified by flash column chromatography (0-5% EtOAc:petrol) to yield the product as an off-white solid (1.26 g, 5.00 mmol, 100%). ^{1}**H NMR** (400 MHz, $CDCl_3$) δ 7.29 (q, *J* = 8.1 Hz, 1H), 6.70 – 6.44 (m, 1H), 3.82 (s, 3H), 1.41 (s, 12H). ^{19}**F NMR** (376 MHz, $CDCl_3$) δ -104.17. ^{13}**C NMR** (126 MHz, $CDCl_3$) δ 166.3 (d, *J* = 243.6 Hz), 164.0 (d, *J* = 13.4 Hz), 132.1 (d, *J* = 10.6 Hz), 107.7 (d, *J* = 24.2 Hz), 105.8 (d, *J* = 2.9 Hz), 84.1, 56.0, 24.7.

Data in accordance with literature.[6]

2-bromo-3,4-dichloroaniline

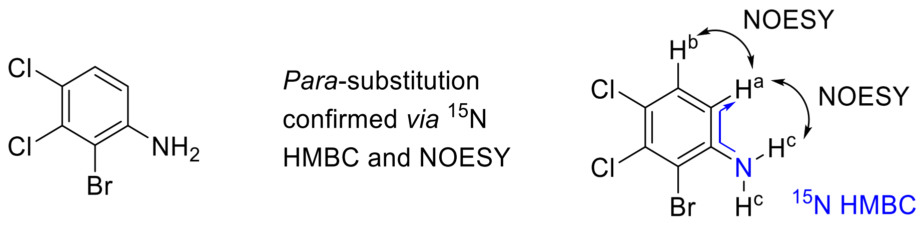

Preparation according to General Procedure **C** with 2-bromo-3-chloroaniline (1.03 g, 5 mmol), NCS (700 mg, 5.25 mmol), and CH_3CN (40 ml). The crude product was purified by flash column chromatography (0–4% EtOAc in petroleum ether) to afford the title compound as an off white solid (681 mg, 2.85 mmol, 57%). The regioselectivity of the chlorination as being *para* relative to the aniline nitrogen was confirmed by ^{15}N HMBC and NOESY experiments. In the ^{15}N HMBC a correlation was observed between the ^{15}N and the aromatic proton labelled H^a. In 1D NOESY when we select this aromatic proton, H^a, we see equal NOE intensity to the other aromatic proton, labelled H^b, as to the NH proton, labelled H^c. In 1D NOESY when we select the NH proton labelled H^c we observe an NOE peak for the aromatic proton labelled H^a. **^{1}H NMR** (700 MHz, $CDCl_3$) δ 7.19 (d, *J* = 8.7 Hz, 1H), 6.62 (d, *J* = 8.7 Hz, 1H), 4.25 (br s, 2H). **^{13}C NMR** (176 MHz, $CDCl_3$) δ 144.7, 133.3, 129.1, 121.8, 113.9, 110.2. **HRMS**: $[M+H]^+$ calculated for $[C_6H_5BrCl_2N]^+$ 239.8977, found 239.8977. Δ = 0.1 ppm.

2-bromo-4-chloro-3-fluoroaniline

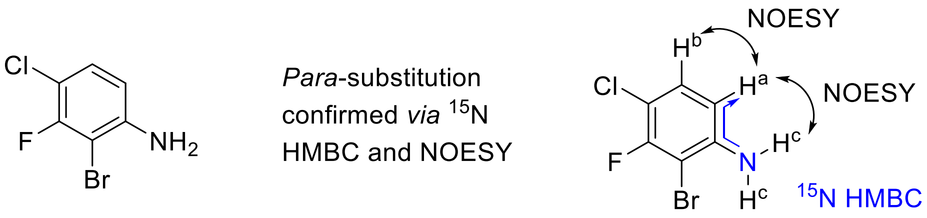

Preparation according to General Procedure **C** with 2-bromo-3-fluoroaniline (950 mg, 5 mmol), NCS (700 mg, 5.25 mmol), and CH_3CN (40 ml). The crude product was purified by flash column chromatography (0–5% EtOAc in petroleum ether) to afford the title compound as an off white solid (553 mg, 2.45 mmol, 49%). The *para*-substitution of the chlorine relative to the amino group was confirmed by ^{15}N HMBC and NOESY. In the ^{15}N HMBC a correlation was observed between the ^{15}N and the aromatic proton labelled H^a. In 1D NOESY when we select this aromatic proton, H^a, we see equal NOE intensity to the other aromatic proton, labelled H^b, as to the NH proton, labelled H^c. In 1D NOESY when we select the NH proton labelled H^c we observe an NOE peak for the aromatic proton labelled H^a. **^{1}H NMR** (700 MHz, $CDCl_3$) δ 7.10 (dd, *J* = 8.8, 7.7 Hz, 1H), 6.49 (dd, *J* = 8.8, 1.8 Hz, 1H), 4.22 (br s, 2H). **^{13}C NMR** (176 MHz, $CDCl_3$) δ 155.4 (d, *J* = 245.3 Hz), 144.8 (d, *J* = 2.5 Hz), 129.2 (d, *J* = 1.3 Hz), 110.7 (d, *J* = 3.4 Hz), 109.7 (d, *J* = 19.3 Hz), 97.3 (d, *J* = 23.5 Hz). **^{19}F NMR** (376 MHz, $CDCl_3$) δ -106.1. **HRMS**: $[M+H]^+$ calculated for $[C_6H_5BrClFN]^+$ 223.9272, found 223.9269. Δ = −1.4 ppm.

2-bromo-4-chloro-3-methylaniline

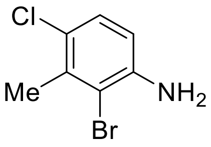

Preparation according to General Procedure **C** with 2-bromo-3-methylaniline (930 mg, 5 mmol), NCS (700 mg, 5.25 mmol), and CH_3CN (40 ml). The crude product was purified by flash column chromatography (0–5% EtOAc in petroleum ether) to afford the title compound as an off white solid (169 mg, 0.75 mmol, 15%). **^{1}H NMR** (700 MHz, $CDCl_3$) δ 7.10 (d, *J* = 8.6 Hz, 1H), 6.58 (d, *J* = 8.6, 1H), 4.12 (br s, 2H), 2.49 (s, 3H). **^{13}C NMR** (176 MHz, $CDCl_3$) δ 143.3, 136.2, 128.4, 123.4, 113.7, 112.8, 21.2. **HRMS**: $[M+H]^+$ calculated for $[C_7H_8BrClN]^+$ 219.9523, found 219.9524. Δ = 0.3 ppm.

The *para*-substitution of the chlorine relative to the amino group was further confirmed by adding ^{n}BuLi (0.25 mL, 0.4 mmol, 4 equiv.) to a solution of the product (22.0 mg, 0.1 mmol) in THF (0.5 mL) at -78 °C, and letting the reaction stir at this temperature for 2 h before being quenched with MeOH (1 mL), to give 4-chloro-3-methylaniline. **^{1}H NMR** (400 MHz, $CDCl_3$) δ 7.09 (d, J = 8.4 Hz, 1H), 6.55 (d, J = 2.4 Hz, 1H), 6.46 (dd, J = 8.4,2.6 Hz, 1H), 3.56 (s, 2H), 2.29 (s, 3H). **^{13}C NMR** (176 MHz, $CDCl_3$) δ 145.0, 136.6, 129.5, 123.5, 117.5, 113.9, 20.2.

Data in accordance with literature.[7]

N-(1-bromo-5,6,7,8-tetrahydronaphthalen-2-yl)pivalamide

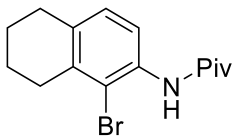

Following a modified patent.[8] To tetralin-6-amine (530 mg, 3.6 mmol) in MeCN (7 mL), cooled to 0 °C under nitrogen, was added a solution of 1-bromopyrrolidine-2,5-dione (641 mg, 3.6 mmol, 1 eq.) in MeCN (7 mL) dropwise. The reaction mixture was allowed to stir at 0 °C for 2 hours. The reaction mixture was poured into water and extracted with EtOAc (3 x 10 mL). The organic layer was washed with brine (10 mL), dried over magnesium sulfate, and filtered. The filtrate was concentrated to give an inseparable mixture of regioisomers. The crude brominated mixture was then dissolved in DCM (7 mL) and cooled to 0 °C. Pivaloyl chloride (0.53 mL, 4.3 mmol, 1.2 eq.) and Triethylamine (0.56 mL, 4.0 mmol, 1.2 eq.) were then added, the ice bath removed, and the mixture allowed to stir for 16 h. The reaction mixture was poured into water and extracted with EtOAc (3 x 10 mL). The organic layer was washed with brine (10 mL), dried over magnesium sulfate, and filtered. The crude material was then purified by flash column chromatography (0–1% EtOAc: petrol) to yield the title compound as a pink powder (519 mg, 1.67 mmol, 47% yield). **^{1}H NMR** (700 MHz, $CDCl_3$) δ 8.10 (d, *J* = 8.5 Hz, 1H), 8.04 (br s, 1H), 7.03 (d, *J* = 8.5 Hz, 1H), 2.74 (q, *J* = 6.1 Hz, 4H), 1.85 – 1.80 (m, 2H), 1.75 – 1.71 (m, 2H), 1.35 (s, 9H). **^{13}C NMR** (176 MHz, $CDCl_3$) δ 176.7, 136.6, 135.1, 133.6, 128.7, 119.1, 117.5, 40.3, 31.1, 29.8, 27.8, 23.4, 22.7. **HRMS**: $[M+H]^+$ calculated for $[C_{15}H_{21}BrNO]^+$ 310.0801, found 310.0803. Δ = 0.5 ppm.

1-bromo-5,6,7,8-tetrahydronaphthalen-2-amine

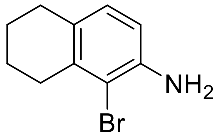

N-(1-bromo-5,6,7,8-tetrahydronaphthalen-2-yl)pivalamide (452 mg, 2 mmol) and HCl (36% w/w, 2 mL) were added to a 6 mL crimp top microwave vial. The vial was then closed with an unpunctured suba seal, taped shut with electrical tape, and heated to 100 °C for 16 h. The vial was then cooled to room temperature, and the contents transferred to ice cold water. The mixture was then extracted with EtOAc (3

x 10 mL) and dried with $MgSO_4$. The crude material was purified by flash column chromatography (0–5% EtOAc: petrol) to yield the title compound as an off-white powder (169 mg, 0.74 mmol, 37% yield). **^{1}H NMR** (700 MHz, $CDCl_3$) δ 6.84 (d, *J* = 8.1 Hz, 1H), 6.60 (d, *J* = 8.1 Hz, 1H), 4.06 (br s, 2H), 2.71 (t, *J* = 6.5 Hz, 2H), 2.68 (t, *J* = 6.2 Hz, 2H), 1.84 – 1.77 (m, 2H), 1.74 – 1.68 (m, 2H). **^{13}C NMR** (176 MHz, $CDCl_3$) δ 142.0, 136.9, 129.0, 128.7, 113.3, 113.0, 30.9, 29.5, 23.6, 23.0. **HRMS**: $[M+H]^+$ calculated for $[C_{10}H_{13}BrN]^+$ 226.0226, found 226.0230. Δ = 1.9 ppm.

2-bromo-3-fluoro-*N*-methylaniline

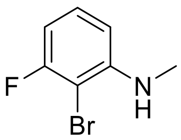

Synthesised according to general procedure **D** with 2-bromo-3-fluoroaniline (950 mg, 5 mmol) and methyl iodide ((851mg, 6 mmol) to afford the title compound as a brown oil (715 mg, 3.20 mmol, 64%).**^{1}H NMR** (700 MHz, $CDCl_3$) δ 7.17 (td, *J* = 8.2, 6.3 Hz, 1H), 6.50 (td, *J* = 8.3, 1.3 Hz, 1H), 6.41 (dt, *J* = 8.3, 1.2 Hz, 1H), 4.48 (br s, 1H), 2.94 (s, 3H). **^{19}F NMR** (376 MHz, $CDCl_3$) δ -106.31. **^{13}C NMR** (176 MHz, $CDCl_3$) δ 159.7 (d, *J* = 243.6 Hz), 147.6 (d, *J* = 3.4 Hz), 128.8 (d, *J* = 10.2 Hz), 105.7 (d, *J* = 2.4 Hz), 104.0 (d, *J* = 22.8 Hz), 96.0 (d, *J* = 23.1 Hz), 30.7. **HRMS** $[M+H]^+$ calcd for $[C_7H_8BrFN]^+$ 203.9819, found 203.9820, Δ= 0.4 ppm.

2-bromo-3-methyl-*N*-methylaniline

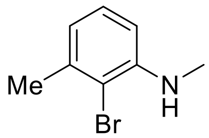

Synthesised according to general procedure **D** with 2-bromo-3-methylaniline (930 mg, 5 mmol) and methyl iodide (851 mg, 6 mmol) to afford the title compound as a brown oil (700 mg, 3.50 mmol, 70%). **^{1}H NMR** (700 MHz, $CDCl_3$) δ 7.13 (t, *J* = 7.7 Hz, 1H), 6.74 – 6.59 (m, 1H), 6.52 (d, *J* = 8.1 Hz, 1H), 4.57 (br s, 1H), 2.92 (s, 3H), 2.40 (s, 3H). **^{13}C NMR** (176 MHz, $CDCl_3$) δ 146.0, 138.3, 127.6, 118.9, 112.3, 108.2, 30.9, 23.7. **HRMS** $[M+H]^+$ calcd for $[C_8H_{11}BrN]^+$ 200.0069, found 200.0070, Δ= 0.3 ppm.

2-bromo-*N*-ethyl-3-fluoroaniline

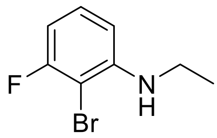

Synthesised according to general procedure **D** with 2-bromo-3-fluoroaniline (950 mg, 5 mmol) and ethyl iodide (935 mg, 6 mmol) to afford the title compound as a brown oil (370 mg, 1.70 mmol, 34%). **^{1}H NMR** (700 MHz, $CDCl_3$) δ 7.14 (td, *J* = 8.2, 6.3 Hz, 1H), 6.49 (td, *J* = 8.3, 1.3 Hz, 1H), 6.42 (dt, *J* = 8.3, 1.2 Hz, 1H), 4.35 (br s, 1H), 3.24 (qd, *J* = 7.2, 4.4 Hz, 2H), 1.34 (t, *J* = 7.2 Hz, 3H). **^{19}F NMR** (376 MHz, $CDCl_3$) δ -105.93. **^{13}C NMR** (176 MHz, $CDCl_3$) δ 159.7 (d, *J* = 243.7 Hz), 146.7 (d, *J* = 3.5 Hz), 128.7 (d, *J* = 10.1 Hz), 106 (d, *J* = 2.6 Hz), 103.8 (d, *J* = 22.8 Hz), 95.9 (d, *J* = 23.1 Hz), 43.2 (m), 14.6. **HRMS** $[M]^-$ calcd for $[C_8H_9BrFN]^-$ 216.9902, found 216.9901, Δ= -3.2 ppm.

2-bromo-*N*-ethyl-3-methylaniline

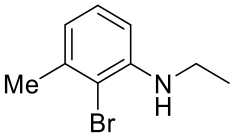

Synthesised according to general procedure **D** with 2-bromo-3-methylaniline (930 mg, 5 mmol) and ethyl iodide (935 mg, 6 mmol) to afford the title compound as a brown oil (321 mg, 1.50 mmol, 30%). **^{1}H NMR** (700 MHz, $CDCl_3$) δ 7.10 (t, *J* = 7.8 Hz, 1H), 6.76 – 6.54 (m, 1H), 6.54 – 6.43 (m, 1H), 4.34 (br s, 1H), 3.22 (q, *J* = 7.2 Hz, 2H), 2.40 (s, 3H), 1.34 (t, *J* = 7.2 Hz, 3H). **^{13}C NMR** (176 MHz, $CDCl_3$) δ 145.2, 138.3, 127.6, 118.7, 112.2, 108.6, 38.6, 23.8, 14.7. **HRMS** [M+H]+ calcd for $[C_9H_{13}BrN]^+$ 214.0226, found 214.0219, Δ= -3.1 ppm.

N-benzyl-2-bromo-3-fluoroaniline

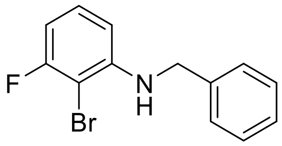

Synthesised according to general procedure **E** with 2-bromo-3-fluoroaniline (950 mg, 5 mmol) to afford the title compound as a brown oil (560 mg, 1.70 mmol, 34%). **^{1}H NMR** (700 MHz, $CDCl_3$) δ 7.55 – 7.35 (m, 4H), 7.35 – 7.30 (m, 1H), 7.10 (td, *J* = 8.2, 6.3 Hz, 1H), 6.51 (td, *J* = 8.3, 1.3 Hz, 1H), 6.41 (dt, *J* = 8.3, 1.2 Hz, 1H), 4.90 (br s, 1H), 4.44 (s, 2H). **^{19}F NMR** (376 MHz, $CDCl_3$) δ -105.79. **^{13}C NMR** (176 MHz, $CDCl_3$) δ 159.7 (d, *J* = 244.0 Hz), 146.4 (d, *J* = 3.2 Hz), 138.2, 128.8, 128.7, 128.7, 127.4, 127.1, 106.7 (d, *J* = 2.7 Hz), 104.4 (d, *J* = 22.7 Hz), 96.2 (d, *J* = 23.2 Hz), 48.1. **HRMS** $[M+H]^+$ calcd for $[C_{13}H_{12}BrFN]^+$ 280.0132, found 280.0132, Δ= 0.1 ppm.

N-(2-bromo-3-methylphenyl)-4-methylbenzenesulfonamide

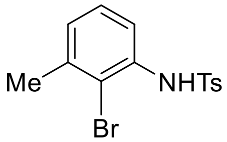

To a stirred solution of 2-bromo-3-methylaniline (1.86 g, 10 mmol) in Pyr/ THF (18 mL/ 6 mL) was added TsCl (1.91 g, 12 mmol, 1.2 eq.) portion wise at room temperature. The reaction mixture was allowed to stir at 40 °C for 16 h. After the solvent was removed under reduced pressure, ethyl acetate (100 mL) was added, and the mixture was washed with water (50 mL × 2) and dried over anhydrous Na_2SO_4. The solvent was then removed under reduced pressure, and the residue was purified by column chromatography (2-10% EtOAc: petrol) to yield the title compound (2.27 g, 6.7 mmol, 67%). **^{1}H NMR** (700 MHz, $CDCl_3$) δ 7.66 (d, J = 8.3 Hz, 2H), 7.49 (dd, *J* = 8.2, 1.5 Hz, 1H), 7.21 (d, J = 8.0 Hz, 2H), 7.14 (t, *J* = 7.9 Hz, 1H), 7.11 (br s, 1H), 6.96 (ddd, *J* = 7.5, 1.6, 0.8 Hz, 1H), 2.37 (s, 3H), 2.31 (s, 3H). **^{13}C NMR** (176 MHz, $CDCl_3$) δ 144.1, 139.0, 136.0, 134.9, 129.6, 127.7, 127.4, 127.0, 119.3, 118.1, 23.8, 21.6.

Data in accordance with literature.[9]

N-(2-bromo-3-methylphenyl)-2,2,2-trifluoroacetamide

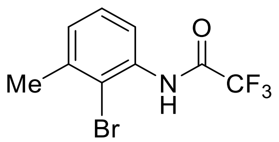

2-bromo-3-methylaniline (930 mg, 5 mmol) and Et_3N (0.84 mL, 6 mmol) were dissolved in DCM (5mL). The mixture was cooled to 0°C and then TFAA (0.83 mL, 6 mmol) was slowly added. The reaction was allowed to reach rt. and stirred for 16 h. The CH_2Cl_2 was then removed *in* vacuo. The crude residue was dissolved in EtOAc (10 ml) washed with 1M HCl (2 x 10 ml), and then purified by silica plug (10% EtOAc: petrol) to give the title compound (1090 mg, 3.87 mmol, 77%). **^{1}H NMR** (700 MHz, $CDCl_3$) δ 8.57 (br s, 1H), 8.14 (dd, *J* = 8.2, 1.6 Hz, 1H), 7.28 (t, *J* = 7.9 Hz, 1H), 7.14 (ddd, *J* = 7.6, 1.6, 0.8 Hz, 1H), 2.46 (s, 3H). **^{19}F NMR** (376 MHz, $CDCl_3$) δ -76.8. **^{13}C NMR** (176 MHz, $CDCl_3$) δ 154.8 (q, *J* = 37.6 Hz), 139.3, 133.4, 128.3, 128.1, 119.6, 117.0, 115.8 (q, *J* = 288.7 Hz), 23.9. **HRMS** $[M+Na]^+$ calcd for $[C_9H_7BrF_3NNaO]^+$; 303.9555, found 303.9550, Δ= -1.7 ppm.

1-bromo-5,6,7,8-tetrahydronaphthalen-2-ol

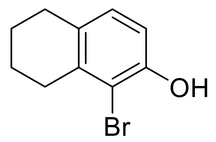

To a solution of 5,6,7,8-tetrahydro-2-naphol (741 mg, 5 mmol) in DMF (5 mL) was added a solution of NBS (890 mg, 5 mmol 1 equiv.) in DMF (5 mL) at 0 °C. The reaction mixture was left to stir for 16 h, at which point the solvent was removed under reduced pressure. The crude material was purified via column chromatography (0-1% EtOAc:petrol) to afford the desired product (908 mg, 4.00 mmol, 80 %) as well as trace (<10%) amounts of the other regioisomer as an inseparable mix. **^{1}H NMR** (500 MHz, $CDCl_3$) δ 6.97 (d, J = 8.3 Hz, 1H), 6.84 (d, J = 8.3 Hz, 1H), 5.49 (s, 1H), 2.77 – 2.70 (m, 4H), 1.89 – 1.79 (m, 2H), 1.79 – 1.71 (m, 2H). **^{13}C NMR** (126 MHz, $CDCl_3$) δ 150.0, 136.6, 131.0, 129.1, 113.5, 112.8, 30.5, 29.3, 23.2, 22.7.

Data in accordance with literature.[6]

5-bromo-6-methoxy-1,2,3,4-tetrahydronaphthalene

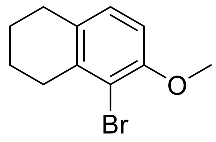

1-bromo-5,6,7,8-tetrahydronaphthalen-2-ol (201 mg, 0.88 mmol), K_2CO_3 (367 mg, 2.66 mmol, 3 equiv.) and methyl iodide (150 mg, 1.06 mmol, 1.2 equiv.), were added to MeCN (3.54 mL). The solution was refluxed for 16 h at which point the solvent was removed under reduced pressure. The crude material was purified via column chromatography (0-1% EtOAc:petrol) to afford the desired product as a white solid (867 mg, 3.60 mmol, 90%). **^{1}H NMR** (500 MHz, $CDCl_3$) δ 7.04 – 7.00 (d, J= 8.4, 1H), 6.73 (d, *J* = 8.4 Hz, 1H), 3.90 (s, 3H), 3.14 – 2.47 (m, 4H), 1.90 – 1.67 (m, 4H). **^{13}C NMR** (126 MHz, $CDCl_3$) δ 153.8, 137.9, 131.5, 128.3, 114.7, 109.2, 56.3, 30.6, 29.4, 23.3, 22.7. **HRMS** $[M]^+$ calcd for $[C_{11}H_{13}BrO]^+$ 240.0144, found 240.0143 Δ= -0.5 ppm.

2-bromo-4-chloro-3-methylphenol

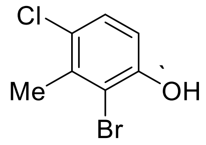

A mixture of 2-bromo-3-methylphenol (935 mg, 5 mmol, 1 equiv.) and NCS (700 mg, 5.25 mmol, 1.05 equiv.) was dissolved in CH_3CN (40 mL, 0.125M) and the mixture heated to 60°C for 16 h. After this time the solution was allowed to cool to room temperature and the solvent removed *in vacuo*. The crude mixture was then purified by column chromatography (0-5% EtOAc:petrol) to give the title compound as a white solid (332 mg, 1.50 mmol, 30 %). **^{1}H NMR** (700 MHz, $CDCl_3$) δ 7.25 (d, *J* = 8.7 Hz, 1H), 6.87 (dd, *J* = 8.7, 0.7 Hz, 1H), 5.57 (s, 1H), 2.53 (s, 3H).**^{13}C NMR** (176 MHz, $CDCl_3$) δ 151.1, 135.9, 128.9, 125.8, 113.9, 113.8, 20.9. **HRMS** $[M-H]^-$ calcd for $[C_7H_5BrClO]^-$ 218.9218, found 218.9212 Δ= -2.8 ppm.

Data in accordance with literature.[10]

The *para*-substitution of the chlorine relative to the hydroxy group was further confirmed by adding ^{n}BuLi (0.25 mL, 0.4 mmol, 4 equiv.) to a solution of the product (22.1 mg, 0.1 mmol) in THF (0.5 mL) at -78 °C, and letting the reaction stir at this temperature for 2 h before being quenched with MeOH (1 mL), to give 4-chloro-3-methylphenol. **^{1}H NMR** (400 MHz, $CDCl_3$) δ 7.18 (d, J = 8.5 Hz, 1H), 6.71 (d, J = 2.8 Hz, 1H), 6.61 (dd, J = 8.6, 3.0 Hz, 1H), 5.14 (br, 1H), 2.31 (s, 3H); **^{13}C NMR** (101 MHz, $CDCl_3$) δ 153.9, 137.4, 129.8, 126.0, 117.8, 114.1, 20.1.

Data in accordance with literature. [11]

7-bromo-6-methylindole

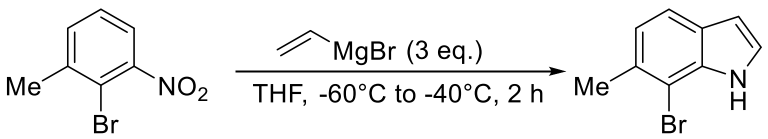

Following a modified procedure by Luo et al.[12] 2-bromo-1-methyl-3-nitrobenzene (2.16 g, 10.0 mmol, 1.0 equiv.) was dissolved in THF (60 mL). The resulting solution was cooled to -60 °C, and vinyl magnesium bromide (1 M in THF, 30 mL, 30.0 mmol, 3.0 equiv.) was added dropwise. The temperature was then raised, and the resulting mixture was stirred at -40 °C for 2 hours and then poured into saturated NH_4Cl. The product was then extracted with EtOAc (×3), the combined organic layers were washed with brine, dried over $MgSO_4$, filtered, and concentrated *in vacuo*. The crude product was purified by flash column chromatography on silica (0.2% EtOAc in petrol) to afford the corresponding indole (1.05 g, 5 mmol, 50%). **^{1}H NMR** (700 MHz, $CDCl_3$) δ 8.27 (br s, 1H), 7.48 (d, *J* = 7.9 Hz, 1H), 7.20 (dd, *J* = 3.0, 2.4 Hz, 1H), 7.02 (d, *J* = 7.9 Hz, 1H), 6.59 (dd, *J* = 3.2, 2.2 Hz, 1H), 2.53 (s, 3H). **^{13}C NMR** (176 MHz, $CDCl_3$) δ 135.3, 130.6, 126.8, 124.1, 122.8, 119.4, 106.6, 103.7, 21.9.

Data in accordance with literature.[13]

7-bromo-6-methylindoline

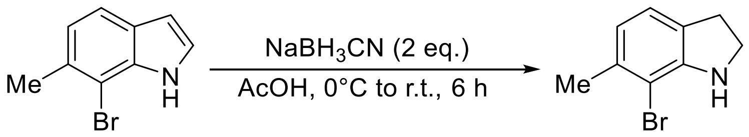

Following a modified procedure by Luo et al.[12] 7-bromo-6-methyl-1H-indole (210 mg, 1.0 mmol, 1.0 equiv.) was dissolved in glacial acetic acid (1.0 mL) and then cooled to 0°C. $NaBH_3CN$ (126 mg, 2.0 mmol, 2.0 equiv.) was added in a single portion, the cooling bath was subsequently removed, and the resulting mixture was stirred vigorously for 6 h. The resulting mixture was then cooled to 0°C and NaOH (10% sol.) was added dropwise until a pH > 11 was reached. EtOAc was added, the organic layer was separated, and the aqueous layer was extracted (×3). The combined organic layers were washed with brine, dried over $MgSO_4$, filtered, and concentrated in vacuo. This crude material was then purified by flash column chromatography (5% EtOAc in petrol) to provide the 7-bromo-6-methylindoline (120 mg, 0.57 mmol, 57%). **^{1}H NMR** (700 MHz, $CDCl_3$) δ 6.92 (d, *J* = 7.3 Hz, 1H), 6.57 (dd, *J* = 7.3, 0.9 Hz, 1H), 3.98 (br s, 1H), 3.62 (t, *J* = 8.4 Hz, 2H), 3.13 (t, *J* = 8.4 Hz, 2H), 2.33 (s, 3H). **^{13}C NMR** (176 MHz, $CDCl_3$) δ 150.6, 136.0, 127.6, 122.8, 120.3, 106.1, 47.2, 30.9, 22.3.

Data in accordance with literature.[12]

Synthesis of Products (Scheme 1)

General procedure F: asymmetric Suzuki cross-coupling to form 2-Amino-2'-hydroxy-1,1'-biphenyls

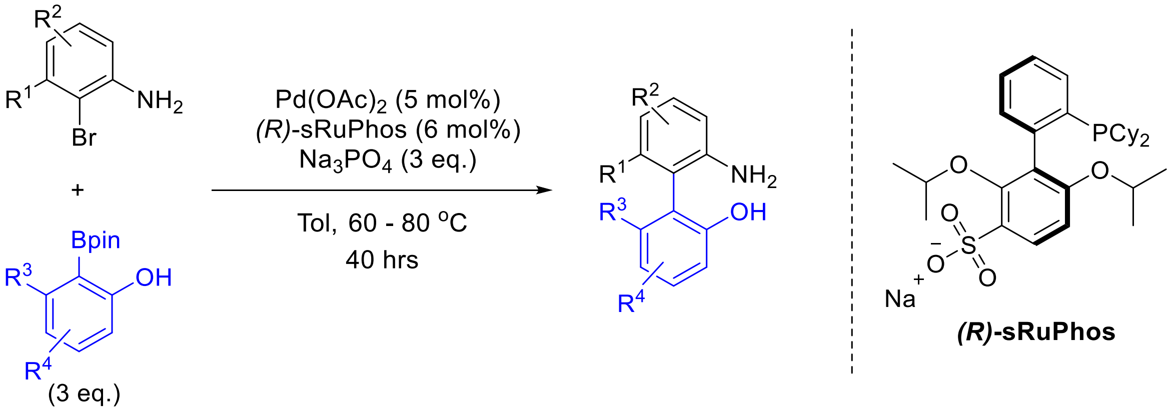

Aryl bromide (0.10 mmol), aryl boronic ester (0.30 mmol, 3 eq.), Na_3PO_4 (49.2 mg, 0.30 mmol), *(R)*- sRuPhos (3.28 mg, 6 mol%) and $Pd(OAc)_2$ (1.12 mg, 5 mol%) were added to a 4 mL crimp vial. After 3 evacuation-backfill cycles with nitrogen, toluene (0.5 mL) was added. The reaction mixture was stirred at the specified temperature for 40 h and then the solvent was removed under a stream of air. The product was purified via column chromatography (petrol/EtOAc).

(S)-2'-amino-6-chloro-6'-fluoro-[1,1'-biphenyl]-2-ol (**3a**)

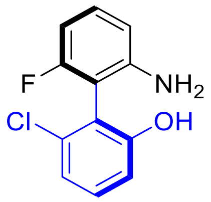

General procedure **F** was performed with 2-bromo-3-fluoroaniline (19.0 mg, 0.1 mmol) and 2- hydroxy-6-chlorophenylboronic acid pinacol ester (76.4 mg, 0.3 mmol), at 60 °C. The product was purified by column chromatography (5-20% EtOAc:petrol) to yield the product as a white solid (16.6 mg, 0.070 mmol, 70%, 99% ee), with trace impurities of the homocoupled byproduct. **^{1}H NMR** (400 MHz, MeOD) δ 7.22 (t, *J* = 8.1 Hz, 1H), 7.12 (td, *J* = 8.2, 6.4 Hz, 1H), 7.03 (dd, *J* = 8.1, 1.1 Hz, 1H), 6.89 (dd, *J* = 8.2, 1.1 Hz, 1H), 6.64 (dt, *J* = 8.1, 0.9 Hz, 1H), 6.47 (ddd, *J* = 9.2, 8.1, 1.1 Hz, 1H). **^{19}F NMR** (376 MHz, MeOD) δ -116.08. **^{13}C NMR** (176 MHz, MeOD) δ 160.9 (d, *J* = 241.5 Hz), 156.6, 147.1 (d, *J* = 6.2 Hz), 135.5 (d, *J* = 1.1 Hz), 129.6, 129.1 (d, *J* = 10.7 Hz), 120.1, 118.8 (d, *J* = 1.6 Hz), 113.7, 110.7 (d, *J* = 2.9 Hz), 109.4 (d, *J* = 20.5 Hz), 103.6 (d, *J* = 23.0 Hz). **HRMS** $[M+H]^+$ calcd for $[C_{12}H_{10}ClFNO]^+$; 238.0429, found 238.0427, Δ= -1.2 ppm. **Chiral SFC Analysis** (IC-3, 96:04 CO_2:MeOH, 2.5 mL/min, 8.33 min [major], 9.31 min [minor]). $[\alpha]_D^{25}$ = +31.4 (c 0.07, $CHCl_3$).

(S)-2'-amino-6-chloro-4',6'-difluoro-[1,1'-biphenyl]-2-ol (**3b**)

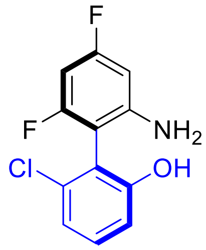

General procedure **F** was performed with 2-bromo-3,5-difluoroaniline (20.8 mg, 0.1 mmol) and 2- hydroxy-6-chlorophenylboronic acid pinacol ester (76.4 mg, 0.3 mmol), at 60 °C. The product was purified by column chromatography (5-20% EtOAc:petrol) to yield the product as a white solid (17.9 mg, 0.070 mmol, 70%, 98% ee). **^{1}H NMR** (400 MHz, $CDCl_3$) δ 7.30 (t, *J* = 8.1 Hz, 1H), 7.15 (dd, *J* = 8.0, 1.1 Hz, 1H), 6.98 (dd, *J* = 8.2, 1.2 Hz, 1H), 6.43-6.36 (m, 2H), 5.15 (br s, 1H), 3.88 (br s, 2H). **^{19}F NMR** (376 MHz, $CDCl_3$) δ -107.98 (d, *J* = 9.5 Hz), -108.49 (d, *J* = 9.5 Hz). **^{13}C NMR** (101 MHz, $CDCl_3$) δ 164.3 (d, *J* = 231.3 Hz), 162.9 (d, *J* = 15.3 Hz), 161.5 (d, *J* = 246.1 Hz), 155.0, 147.2 (dd, *J* = 13.3, 7.8 Hz), 135.7, 130.9, 122.1, 116.8, 114.6, 98.1 (dd, *J* = 25.0, 3.3 Hz), 94.3 (t, *J* = 26.5 Hz). **HRMS** $[M+H]^+$ calcd for $[C_{12}H_9ClF_2NO]^+$; 256.0335, found 256.0337, Δ= +0.7 ppm. **Chiral SFC Analysis** (IG-3, 98:02 CO_2:MeOH, 2.5 mL/min, 23.35 min [major], 29.99 min [minor]). $[\alpha]_D^{25}$ = +4.0 (c 0.4, $CHCl_3$).

(S)-6'-amino-6-chloro-2',3'-difluoro-[1,1'-biphenyl]-2-ol (**3c**)

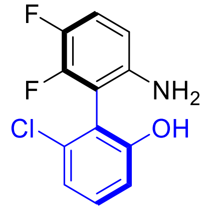

General procedure **F** was performed with 2-bromo-3,4-difluoroaniline (20.8 mg, 0. 1 mmol) and 2- hydroxy-6-chlorophenylboronic acid pinacol ester (76.4 mg, 0.3 mmol), at 80 °C. The product was purified by column chromatography (10–20% EtOAc: petrol) to yield the title compound, (17.1 mg, 0.067 mmol, 67%, 98% ee). **^{1}H NMR** (700 MHz, $CDCl_3$) δ 7.28 (t, *J* = 8.1 Hz, 1H), 7.13 (dd, *J* = 8.1, 1.1 Hz, 1H), 7.09 (dt, *J* = 9.8, 8.7 Hz, 1H), 6.95 (dd, *J* = 8.3, 1.1 Hz, 1H), 6.55 (ddd, *J* = 9.0, 3.9, 1.8 Hz, 1H). **^{13}C NMR** (176 MHz, $CDCl_3$) δ 154.9, 148.6 (dd, *J* = 247.4, 13.4 Hz), 144.3 (dd, *J* = 239.6, 13.4 Hz), 141.0 (dd, *J* = 3.6, 2.2 Hz), 135.3, 131.0, 122.2, 118.3 (dd, *J* = 18.2, 2.0 Hz), 117.2 (d, *J* = 2.2 Hz), 115.3, 110.5 (dd, *J* = 6.1, 3.9 Hz), 109.5 (d, *J* = 16.5 Hz). **^{19}F NMR** (376 MHz, $CDCl_3$) δ -135.0 (d, *J* = 22.4 Hz), -149.3 (d, *J* = 22.8 Hz). **HRMS** $[M+H]^+$ calcd for $[C_{12}H_9ClF_2NO]^+$ 256.0335, found 256.0335. Δ = −0.2 ppm. **Chiral SFC Analysis** (IC-3, CO_2:MeOH 96:04, 2.5 mL min^{-1} , 40 °C) tR = 7.66 (major), 9.29 (minor) minutes. $[\alpha]_D^{25}$ = +54.0 (c 0.07, $CHCl_3$).

(S)- 6'-amino-3',6-dichloro-2'-fluoro-[1,1'-biphenyl]-2-ol (**3d**)

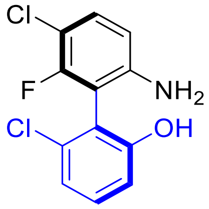

General procedure **F** was performed with 2-bromo-4-chloro-3-fluoroaniline (22.4 mg, 0.1 mmol) and 2-hydroxy-6-chlorophenylboronic acid pinacol ester (76.4 mg, 0.3 mmol), at 80 °C. The product was purified by column chromatography (5-20% EtOAc:petrol) to yield the product as a white solid (18.4 mg, 0.068 mmol, 68%, 96% ee). **^{1}H NMR** (400 MHz, MeOD) δ 7.37 – 7.23 (m, 2H), 6.84 – 6.63 (m, 3H). **^{19}F NMR** (376 MHz, MeOD) δ -115.04. **^{13}C NMR** (176 MHz, MeOD) δ 156.6, 155.7 (d, *J* = 242.8 Hz), 146.3 (d, *J* = 5.4 Hz), 135.3 (d, *J* = 0.8 Hz), 130.0, 129.3, 120.1, 118.0, 113.8, 110.9 (d, *J* = 3.3 Hz), 110.5 (d, *J* = 20.3 Hz), 107.5 (d, *J* = 19.3 Hz). **HRMS** $[M+H]^+$ calcd for $C_{12}H_9FCl_2NO$; 272.0040, found 272.0045, Δ= +1.8 ppm. **Chiral SFC Analysis** (IC-3, 94:06 CO_2:MeOH, 2.5 mL/min, 6.26 min [major], 8.92 min [minor]). $[\alpha]_D^{25}$ = +7.2 (c 0.29, $CHCl_3$).

(S)- 2'-amino-6-chloro-6'-fluoro-3'-nitro-[1,1'-biphenyl]-2-ol (**3e**)

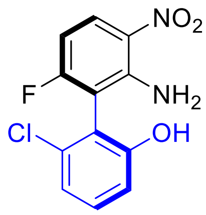

General procedure **F** was performed with 2-bromo-3-fluoro-6-nitroaniline (23.5 mg, 0.1 mmol) and 2-hydroxy-6-chlorophenylboronic acid pinacol ester (76.4 mg, 0.3 mmol), at 60 °C. The product was purified by column chromatography (5-25% EtOAc:petrol) to yield the product as a yellow solid (14.7 mg, 0.052 mmol, 52%, 96% ee). **^{1}H NMR** (400 MHz, $CDCl_3$) δ 8.36 (dd, *J* = 9.6, 5.8 Hz, 1H), 7.37 (t, *J* = 8.2 Hz, 1H), 7.21 (dd, *J* = 8.1, 1.1 Hz, 1H), 7.00 (dd, *J* = 8.2, 1.1 Hz, 1H), 6.63 (dd, *J* = 9.6, 7.6 Hz, 1H), 6.23 (br s, 2H), 4.96 (br s, 1H). **^{19}F NMR** (376 MHz, $CDCl_3$) δ -97.91. **^{13}C NMR** (101 MHz, $CDCl_3$) δ 171.2, 164.4 (d, *J* = 256.7 Hz), 154.9, 145.1 (d, *J* = 8.3 Hz), 135.7, 131.7, 129.8 (d, *J* = 12.7 Hz), 122.5, 115.2, 114.9, 108.5 (d, *J* = 21.7 Hz), 105.2 (d, *J* = 25.4 Hz). **HRMS** $[M+H]^+$ calcd for $C_{12}H_9ClFN_2O_3$; 283.0280, found 283.0291, Δ= +3.7 ppm. **Chiral SFC Analysis** (IC-3, 94:06 CO_2:MeOH, 2.5 mL/min, 7.95 min [major], 9.44 min [minor]). $[\alpha]_D^{25}$ = -39.8 (c 0.15, $CHCl_3$).

(S)- 2'-amino-6'-chloro-6-fluoro-[1,1'-biphenyl]-2-ol (**3f**)

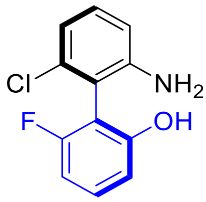

General procedure **F** was performed with 2-bromo-3-chloroaniline (20.6 mg, 0.1 mmol) and 2- hydroxy-6-fluorophenylboronic acid pinacol ester (71.4 mg, 0.3 mmol), at 60 °C. The product was purified by column chromatography (5-20% EtOAc:petrol) to yield the product as a white solid (17.3 mg, 0.073 mmol, 73%, 99% ee). **^{1}H NMR** (400 MHz, MeOD) δ 7.26 (td, *J* = 8.3, 6.6 Hz, 1H), 7.10 (t, *J* = 8.0 Hz, 1H), 6.82 (dd, *J* = 7.9, 0.9 Hz, 1H), 6.81 – 6.73 (m, 2H), 6.70 (t, *J* = 9.1 Hz, 1H). **^{19}F NMR** (376 MHz, MeOD) δ -114.65. **^{13}C NMR** (126 MHz, MeOD) δ 161.0 (d, *J* = 243.6 Hz), 156.3 (d, *J* = 6.9 Hz), 147.4, 135.0, 129.6 (d, *J* = 10.9 Hz), 129.0, 117.9, 116.7, 113.4, 111.2 (d, *J* = 20.0 Hz), 111.0 (d, *J* = 3.3 Hz), 106.0 (d, *J* = 22.9 Hz). **HRMS** $[M+H]^+$ calcd for $C_{12}H_{10}ClFNO$; 238.0429, found 238.0427, Δ= -0.9 ppm. **Chiral SFC Analysis** (IC-3, 94:06 CO_2:MeOH, 2.5 mL/min, 5.50 min [major], 6.12 min [minor]). $[\alpha]_D^{25}$ = + 4.8 (c 0.08, $CHCl_3$).

(S)-6'-amino-2',3'-dichloro-6-fluoro-[1,1'-biphenyl]-2-ol (**3g**)

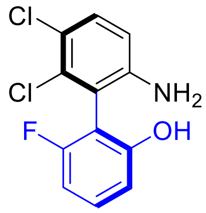

General procedure **F** was performed with 2-bromo-3,4-dichloroaniline (24.0 mg, 0.1 mmol) and 2-hydroxy-6-fluorophenylboronic acid pinacol ester (71.4 mg, 0.3 mmol), at 80 °C. The product was purified by column chromatography (5-20% EtOAc:petrol) to yield the product as a white solid (16.4 mg, 0.060 mmol, 60%, 99% ee), with trace impurity of the homocoupled by product. **^{1}H NMR** (400 MHz, MeOD) δ 7.25 (t, *J* = 8.1 Hz, 1H), 7.20 – 7.13 (m, 1H), 7.05 (dd, *J* = 8.1, 1.1 Hz, 1H), 6.90 (dd, *J* = 8.2, 1.1 Hz, 1H), 6.61 (dd, *J* = 8.7, 1.4 Hz, 1H). **^{19}F NMR** (376 MHz, MeOD) δ -117.71. **^{13}C NMR** (176 MHz, MeOD) δ 160.8 (d, J = 244.0 Hz), 156.3 (d, J = 6.9 Hz), 146.3, 132.7 (d, J = 1.0 Hz), 130.0 (d, J = 10.8 Hz), 129.5, 119.9, 118.2 (d, J = 1.2 Hz), 114.1, 111.2 (d, J = 24.6 Hz), 111.2 (d, J = 3.1 Hz), 106.1 (d, J = 22.6 Hz). **HRMS** $[M+H]^+$ calcd for $C_{12}H_9FCl_2NO$; 272.040, found 272.0042, Δ= +0.9 ppm. **Chiral SFC Analysis** (IC-3, 94:06 CO_2:MeOH, 2.5 mL/min, 6.56 min [major], 8.56 min [minor]). $[\alpha]_D^{25}$ = +20.6 (c 0.21, $CHCl_3$).

(S)- 2'-amino-4',6'-dichloro-6-fluoro-[1,1'-biphenyl]-2-ol (**3h**)

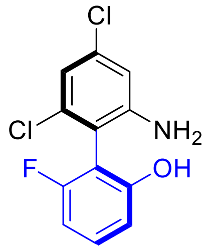

General procedure **F** was performed with 2-bromo-3,5-dichloroaniline (24.0 mg, 0.1 mmol) and 2- hydroxy-6-fluorophenylboronic acid pinacol ester (71.4 mg, 0.3 mmol), at 60 °C. The product was purified by column chromatography (5-20% EtOAc:petrol) to yield the product (15.0 mg, 0.055 mmol, 55%, 98% ee). **^{1}H NMR** (400 MHz, $CDCl_3$) δ 7.34 (td, *J* = 8.3, 6.5 Hz, 1H), 6.99 (d, *J* = 1.9 Hz, 1H), 6.88 (dt, *J* = 8.3, 1.0 Hz, 1H), 6.82 (td, *J* = 8.5, 1.0 Hz, 1H), 6.77 (d, *J* = 2.0 Hz, 1H), 3.79 (br s, 3H). **^{19}F NMR** (376 MHz, $CDCl_3$) δ -111.83. **^{13}C NMR** (126 MHz, $CDCl_3$) δ 160.4 (d, *J* = 247.4 Hz), 154.5 (d, *J* = 5.6 Hz), 147.0, 136.9 (d, *J* = 0.8 Hz), 136.1, 131.0 (d, *J* = 10.5 Hz), 119.6, 113.8, 112.4, 112.0 (d, *J* = 3.4 Hz), 109.5 (d, *J* = 20.2 Hz), 108.1 (d, *J* = 22.2 Hz). **HRMS** $[M+H]^+$ calcd for $C_{12}H_9Cl_2FNO$; 272.0040, found 272.0038, Δ= -0.8 ppm. **Chiral SFC Analysis** (IG-3, 90:10 CO_2:MeOH, 2.5 mL/min, 8.89 min [major], 11.46 min [minor]). $[\alpha]_D^{25}$ = +26.7 (c 0.03, $CHCl_3$).

(R)-2'-amino-6-fluoro-6'-methyl-[1,1'-biphenyl]-2-ol (**3i**)

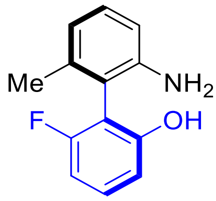

General procedure **F** was performed with 2-bromo-3-methylaniline (18.6 mg, 0.1 mmol) and 2- hydroxy-6-fluorophenylboronic acid pinacol ester (71.4 mg, 0.3 mmol), at 60 °C. The product was purified by column chromatography (5-20% EtOAc:petrol) to yield the product as a grey solid (13.3 mg, 0.061 mmol, 61%, 98% ee). ^{1}**H NMR** (500 MHz, $CDCl_3$) δ 7.40 – 7.24 (m, 1H), 7.19 (t, *J* = 7.8 Hz, 1H), 6.89 (dd, *J* = 8.3, 1.1 Hz, 1H), 6.80 (dt, *J* = 7.5, 4.8 Hz, 2H), 6.71 (d, *J* = 8.0 Hz, 1H), 5.26 (br s, 1H), 3.64 (br s, 2H), 2.07 (s, 3H). ^{19}**F NMR** (471 MHz, $CDCl_3$) δ -112.78. ^{13}**C NMR** (176 MHz, $CDCl_3$) δ 160.4 (d, *J* = 245.3 Hz), 154.6 (d, *J* = 6.1 Hz), 145.0, 139.6, 130.1 (d, *J* = 10.4 Hz), 130.0, 120.9, 114.3, 113.4, 111.9 (d, *J* = 24.6 Hz), 111.9 (d, *J* = 3.2 Hz), 107.9 (d, *J* = 22.8 Hz), 20.0 (d, *J* = 1.76 Hz). **HRMS** $[M+H]^+$ calcd for $C_{13}H_{13}FNO$; 218.0976, found 218.0974, Δ= -0.6 ppm. **Chiral HPLC Analysis** (SB-3, 95:05 Hexane : ^{i}PrOH, 1.25 mL/min, 11.34 min [major], 16.11 min [minor]). $[\alpha]_D^{25}$ = + 75.0 (c 0.19, $CHCl_3$).

(R)-2-(2-amino-5,6,7,8-tetrahydronaphthalen-1-yl)-3-fluorophenol (**3j**)

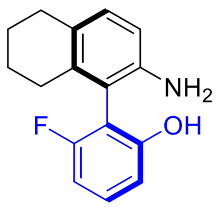

General procedure **F** was performed with 1-bromo-5,6,7,8-tetrahydronaphthalen-2-amine (22.6 mg, 0. 1 mmol) and 2- hydroxy-6-fluorophenylboronic acid pinacol ester (71.4 mg, 0.3 mmol), at 60 °C. The product was purified by column chromatography (5–15% EtOAc: petrol) to yield the title compound, (16.3 mg, 0.063 mmol, 63%, 99% ee). ^{1}**H NMR** (700 MHz, MeOD) δ 7.18 (td, *J* = 8.3, 6.6 Hz, 1H), 6.85 (d, *J* = 8.2 Hz, 1H), 6.72 (dt, *J* = 8.3, 0.9 Hz, 1H), 6.66 – 6.61 (m, 2H), 2.67 (t, *J* = 6.4 Hz, 2H), 2.26 (t, *J* = 6.0 Hz, 2H), 1.72 – 1.60 (m, 4H). ^{13}**C NMR** (176 MHz, MeOD) δ 160.8 (d, *J* = 241.8 Hz), 156.1 (d, *J* = 7.2 Hz), 142.0, 136.4, 129.2, 129.2 (d, *J* = 10.7 Hz), 127.5, 117.7, 113.8, 112.5 (d, *J* = 20.8 Hz), 111.2 (d, *J* = 3.1 Hz), 106.3 (d, *J* = 23.2 Hz), 29.0, 27.0, 23.1, 22.9. ^{19}**F NMR** (376 MHz, $CDCl_3$) δ -113.7. **HRMS** $[M+H]^+$ calculated for $[C_{16}H_{17}FNO]^+$ 258.1289, found 258.1287. Δ = −0.7 ppm. **Chiral SFC Analysis** (SB-3, 90:10, CO_2 : ^{i}PrOH, 2.5 mL min^{-1} , 40 °C) tR = 5.50 (major), 6.44 (minor). $[\alpha]_D^{25}$ = + 33.9 (c 0.56, $CHCl_3$).

(R)-6'-amino-3'-chloro-6-fluoro-2'-methyl-[1,1'-biphenyl]-2-ol (**3k**)

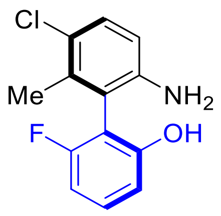

General procedure **F** was performed with 2-bromo-4-chloro-3-methylaniline (22.0 mg, 0. 1 mmol) and 2-hydroxy-6-fluorophenylboronic acid pinacol ester (71.4 mg, 0.3 mmol), at 80 °C. The product was purified by column chromatography (0–5% EtOAc: $CHCl_3$) to yield the title compound (16.1 mg, 0.064 mmol, 64%, 97% *ee*). ^{1}**H NMR** (700 MHz, MeOD) δ 7.25 (td, *J* = 8.3, 6.7 Hz, 1H), 7.12 (d, *J* = 8.6 Hz, 1H), 6.78 (dt, *J* = 8.3, 0.9 Hz, 1H), 6.70 (ddd, *J* = 9.1, 8.3, 1.0 Hz, 1H), 6.67 (dd, *J* = 8.7, 0.6 Hz, 1H), 2.01 (s, 3H). ^{13}**C NMR** (176 MHz, MeOD) δ 160.8 (d, *J* = 242.7 Hz), 156.2 (d, *J* = 7.0 Hz), 144.4, 135.3, 129.6 (d, *J* = 10.7 Hz), 128.5, 122.9, 119.2 (d, *J* = 1.1 Hz), 114.0, 112.4 (d, *J* = 20.3 Hz), 111.3 (d, *J* = 3.0 Hz), 106.2 (d, *J* = 23.0 Hz), 16.2. ^{19}**F NMR** (376 MHz, $CDCl_3$) δ -113.47. **HRMS** $[M+H]^+$ calculated for $[C_{13}H_{12}ClFNO]^+$ 252.0586, found 252.0587. Δ = 0.4

ppm. **Chiral SFC Analysis** (SB-3, 90:10, CO_2 : ^{i}PrOH, 1.25 mL min^{-1} , 40 °C) tR = 6.99 (major), 7.81 (minor). $[\alpha]_D^{25}$ = + 42.5 (c 0.04, $CHCl_3$).

(S)-2'-amino-4',6'-dichloro-5,6-difluoro-[1,1'-biphenyl]-2-ol (**3l**)

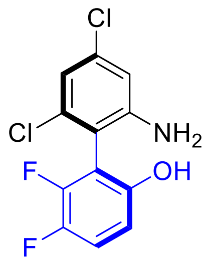

General procedure **F** was performed with 2-bromo-3,5-dichloroaniline (24.0 mg, 0. 1 mmol) and 2- hydroxy-5,6-difluorophenylboronic acid pinacol ester (76.8 mg, 0.3 mmol), at 80 °C. The product was purified by column chromatography (10–25% EtOAc: petrol) to yield the title compound, (6.1 mg, 0.021 mmol, 21%, 95% ee). **^{1}H NMR** (700 MHz, $CDCl_3$) δ 7.17 (q, *J* = 9.2 Hz, 1H), 6.98 (d, *J* = 2.0 Hz, 1H), 6.78 (ddd, *J* = 9.2, 3.9, 2.0 Hz, 1H), 6.76 (d, *J* = 2.0 Hz, 1H). **^{13}C NMR** (176 MHz, $CDCl_3$) δ 149.7 (t, *J* = 3.1 Hz), 148.0 (dd, *J* = 249.0, 13.8 Hz), 147.0, 145.5 (dd, *J* = 241.7, 13.2 Hz), 136.9, 136.7, 119.9, 118.3 (d, *J* = 18.6 Hz), 114.2, 111.8, 111.6 (dd, *J* = 6.3, 3.7 Hz), 111.5 (d, *J* = 16.4 Hz). **^{19}F NMR** (376 MHz, $CDCl_3$) δ -134.6 (d, *J* = 22.2 Hz), -146.4 (d, *J* = 22.4 Hz). **HRMS**: $[M+H]^+$ calculated for $[C_{12}H_8Cl_2F_2NO]^+$ 289.9946, found 289.9947. Δ = 0.5 ppm. **Chiral SFC Analysis** (IC-3, 98:02, CO_2:MeOH, 2.5 mL min^{-1} , 40 °C) tR = 16.42 (major), 18.94 (minor) minutes. $[\alpha]_D^{25}$ = + 23.3 (c 0.06, $CHCl_3$).

(S)-2'-amino-6'-chloro-5,6-difluoro-[1,1'-biphenyl]-2-ol (**3m**)

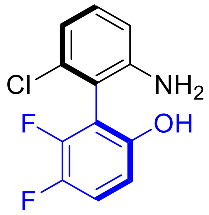

General procedure **F** was performed with 2-bromo-3-chloroaniline (20.7 mg, 0. 1 mmol) and 2- hydroxy-5,6-difluorophenylboronic acid pinacol ester (76.8 mg, 0.3 mmol), at 60 °C. The product was purified by column chromatography (10–25% EtOAc: petrol) to yield the title compound, (15.6 mg, 0.061 mmol, 61%, 98% ee). **^{1}H NMR** (700 MHz, Acetone) δ 7.21 (dt, *J* = 10.4, 9.1 Hz, 1H), 7.10 (t, *J* = 8.1 Hz, 1H), 6.82 – 6.75 (m, 3H), 4.62 (br s, 2H). **^{13}C NMR** (176 MHz, Acetone) δ 152.0 (d, *J* = 4.8 Hz), 148.5, 148.2 (dd, *J* = 243.6, 13.4 Hz), 144.5 (dd, *J* = 236.6, 13.8 Hz), 134.9, 130.0, 117.0, 116.7 (d, *J* = 17.4 Hz), 114.3, 113.4, 113.3 (d, *J* = 16.2 Hz), 110.9 (dd, *J* = 6.4, 3.5 Hz). **^{19}F NMR** (376 MHz, Acetone) δ -139.0 (d, *J* = 22.6 Hz), -152.2 (d, *J* = 22.6 Hz). **HRMS**: $[M+H]^+$ calculated for $[C_{12}H_9ClF_2NO]^+$ 256.0335, found 256.0335. Δ = −0.1 ppm. **Chiral SFC Analysis** (IC-3, 96:04, CO_2:MeOH, 2.5 mL min^{-1} , 40 °C) tR = 6.51 (major). $[\alpha]_D^{25}$ = + 83.9 (c 0.15, $CHCl_3$).

(R)-2'-amino-5,6-difluoro-6'-methyl-[1,1'-biphenyl]-2-ol (**3n**)

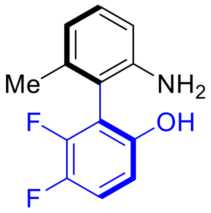

General procedure **F** was performed with 2-bromo-3-methylaniline (18.6 mg, 0. 1 mmol) and 2- hydroxy-5,6-difluorophenylboronic acid pinacol ester (76.8 mg, 0.3 mmol), at 80 °C. The product was purified by column chromatography (10–20% EtOAc: petrol) to yield the title compound, (18.1 mg, 0.077 mmol, 77%, 94% ee). **^{1}H NMR** (700 MHz, $CDCl_3$) δ 7.18 (t, *J* = 7.8 Hz, 1H), 7.13 (q, *J* = 9.2 Hz, 1H), 6.81 – 6.77 (m, 2H), 6.70 (d, *J* = 8.1 Hz, 1H), 2.06 (s, 3H). **^{13}C NMR** (176 MHz, $CDCl_3$) δ 149.7 (t, *J* = 3.1 Hz), 147.7 (dd, *J* = 246.9, 13.5 Hz), 145.3 (dd, *J* = 241.0, 13.6 Hz), 144.5, 139.5, 130.3, 121.2, 117.1 (d, *J* = 18.7 Hz), 114.0, 113.8 (d, *J* = 17.0 Hz), 113.7, 111.3 (dd, *J* = 6.4, 3.5 Hz), 19.9. **^{19}F NMR** (376 MHz, $CDCl_3$) δ -135.8 (d, *J* = 22.6 Hz), -147.0 (d, *J* = 22.6 Hz). **HRMS**: $[M+H]^+$ calculated for $[C_{13}H_{12}F_2NO]^+$ 236.0881, found 236.0881. Δ = −0.2 ppm. **Chiral HPLC Analysis** (SB-3, 95:05, Hexane : ^{i}PrOH, 1.25 mL min^{-1} , 40 °C, tR = 13.28 (major), 20.52(minor). $[\alpha]_D^{25}$ = + 76.5 (c 0.13, $CHCl_3$).

(R)-2-(2-amino-5,6,7,8-tetrahydronaphthalen-1-yl)-3,4-difluorophenol (**3o**)

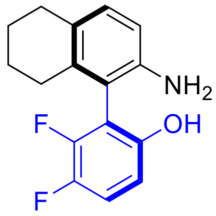

General procedure **F** was performed with 1-bromo-5,6,7,8-tetrahydronaphthalen-2-amine (22.6 mg, 0.1 mmol) and 2-hydroxy-5,6-difluorophenylboronic acid pinacol ester (76.8 mg, 0.3 mmol), at 80 °C. The product was purified by column chromatography (5-20% EtOAc:petrol) to yield the product as a light brown solid (9.1 mg, 0.033 mmol, 33%, 97% ee). NMR contained solvent impurity. **^{1}H NMR** (500 MHz, MeOD) δ 7.13 (dt, *J* = 10.2, 9.0 Hz, 1H), 6.92 (d, *J* = 8.2 Hz, 1H), 6.86 – 6.51 (m, 2H), 2.73 (t, *J* = 6.1 Hz, 2H), 2.31 (t, *J* = 6.1 Hz, 2H), 1.93 – 1.58 (m, 4H). **^{19}F NMR** (376 MHz, $CDCl_3$) δ -135.81 (d, *J* = 23.0 Hz), -147.12 (d, *J* = 23.0 Hz). **^{13}C NMR** (126 MHz, $CDCl_3$) δ 149.6 (dd, *J* = 4.4, 2.5 Hz), 147.7 (dd, *J* = 246.3, 13.5 Hz), 145.3 (d, *J* = 227.2 Hz), 142.4, 137.8, 131.5, 128.6, 116.9 (dd, *J* = 18.6, 2.2 Hz), 114.0 (dd, *J* = 16.38 Hz), 113.8, 113.1 (d, *J* = 2.5 Hz), 111.0 (dd, *J* = 6.7, 3.9 Hz), 29.2, 27.2 (d, *J* = 1.25), 23.0, 22.9 . **HRMS** $[M+H]^+$ calcd for $C_{16}H_{16}F_2NO$; 276.1194, found 276.1196, Δ= +0.6 ppm. **Chiral SFC Analysis** (IC-3, 95:05 CO_2:MeOH, 2.5 mL/min, 11.09 min [major], 13.28 min [minor]). $[\alpha]_D^{25}$ = +36.9 (c 0.33, $CHCl_3$).

(R)-2'-amino-6'-fluoro-6-methyl-[1,1'-biphenyl]-2-ol (**3p**)

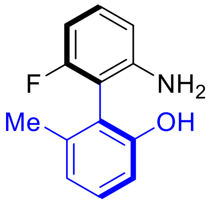

General procedure **F** was performed with 2-bromo-3-fluoroaniline (19.0 mg, 0.1 mmol) and 2- hydroxy-6-methylphenylboronic acid pinacol ester (70.2 mg, 0.3 mmol), at 60 °C. The product was purified by column chromatography (5-20% EtOAc:petrol) to yield the product as a white solid (10.9 mg, 0.050 mmol, 50%, 92% ee). **^{1}H NMR** (500 MHz, $CDCl_3$) δ 7.32 – 7.13 (m, 2H), 6.99 – 6.83 (m, 2H), 6.70 – 6.56 (m, 2H), 4.95 (br s, 1H), 3.70 (br s, 2H), 2.13 (s, 3H). **^{19}F NMR** (376 MHz, $CDCl_3$) δ -112.49. **^{13}C NMR** (126 MHz, $CDCl_3$) δ 161.2 (d, *J* = 244.1 Hz), 153.5, 146.4 (d, *J* = 6.1 Hz), 139.3, 130.6 (d, *J* = 10.5 Hz), 129.8, 122.4, 117.6 (d, *J* = 1.5 Hz), 113.2, 110.8 (d, *J* = 3.1 Hz), 107.4 (d, *J* = 21.3 Hz), 105.3 (d, *J* = 22.9 Hz), 19.7 (d, *J* = 1.2 Hz). **HRMS** $[M+H]^+$ calcd for $C_{12}H_{10}ClFNO$; 218.0976, found 218.0974, Δ= -0.8 ppm **Chiral HPLC Analysis** (SB-3, 95:05 Hexane : ^{i}PrOH, 1.25 mL/min, 13.48 min [major], 17.03 min [minor]). $[\alpha]_D^{25}$ = +2.3 (c 0.24, $CHCl_3$).

(R)-1-(2-amino-6-fluorophenyl)-5,6,7,8-tetrahydronaphthalen-2-ol (**3q**)

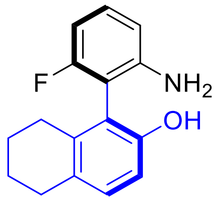

General procedure **F** was performed with 2-bromo-3-fluoroaniline (19.0 mg, 0.1 mmol) and 1-(4,4,5,5-tetramethyl-1,3,2-dioxaborolan-2-yl)-5,6,7,8-tetrahydronaphthalen-2-ol (82.2 mg, 0.3 mmol), at 80 °C. The product was purified by column chromatography (5-20% EtOAc:petrol) to yield the product as a light brown solid (10.1 mg, 0.041 mmol, 41%, 93% ee). *After isolation the product decomposes in solution at r.t. which gives rise to extra peaks visible in the NMR spectra.* **^{1}H NMR** (700 MHz, $CDCl_3$) δ 7.20 (td, *J* = 8.2, 6.3 Hz, 1H), 7.08 (d, *J* = 8.3 Hz, 1H), 6.85 (d, *J* = 8.3 Hz, 1H), 6.63 – 6.58 (m, 2H), 4.62 (br s, 1H), 3.65 (br s, 2H), 2.84 – 2.71 (m, 2H), 2.46 (ddd, *J* = 19.9, 8.1, 5.1 Hz, 1H), 2.34 (dt, *J* = 16.8, 5.7 Hz, 1H), 1.80 – 1.75 (m, 3H), 1.70 – 1.65 (m, 1H). **^{19}F NMR** (376 MHz, $CDCl_3$) δ -112.30. **^{13}C NMR** (176 MHz, $CDCl_3$) δ 161.2 (d, *J* = 243.7 Hz), 151.2, 146.4 (d, *J* = 6.2 Hz), 137.5, 130.8, 130.4 (d, *J* = 10.5 Hz), 129.8, 116.8, 113.0, 110.7 (d, *J* = 3.0 Hz), 107.4 (d, *J* = 21.4 Hz), 105.3 (d, *J* = 22.9 Hz), 29.2, 27.0, 23.0, 22.9. **HRMS** $[M+H]^+$ calcd for $C_{16}H_{17}FNO$; 258.1289, found 258.1287, Δ= -0.8 ppm. **Chiral HPLC Analysis** (IG-3, 95:05 Hexane : ^{i}PrOH, 1.25 mL/min, 17.15 min [major], 23.31 min [minor]). $[\alpha]_D^{25}$ = -2.3 (c 0.26, $CHCl_3$).

(S)-2'-amino-3',5'-dichloro-6-methyl-[1,1'-biphenyl]-2-ol (**3r**)

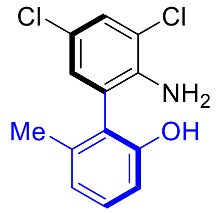

General procedure **F** was performed with 2-bromo-4,6-dichloroaniline (24.0 mg, 0.1 mmol) and 2- hydroxy-6-methylphenylboronic acid pinacol ester (70.2 mg, 0.3 mmol), at 40°C. The product was purified by column chromatography (5-20% EtOAc:petrol) to yield the product as a white solid (8.6 mg, 0.032 mmol, 32%, 80% ee). **^{1}H NMR** (500 MHz, $CDCl_3$) δ 7.35 (d, *J* = 2.4 Hz, 1H), 7.22 (t, *J* = 7.9 Hz, 1H), 6.99 (d, *J* = 2.4 Hz, 1H), 6.91 – 6.86 (m, 2H), 2.08 (s, 3H).**^{13}C NMR** (126 MHz, $CDCl_3$) δ 153.3, 140.5, 138.4, 130.0, 129.5, 129.4, 122.9, 122.8, 122.5, 122.1, 120.4, 113.7, 20.0. **HRMS**: $[M-H]^+$ calculated for $[C_{13}H_{10}Cl_2NO]^-$ 266.0145, found

266.0137. Δ = −2.9 ppm. **Chiral SFC Analysis** (IA-3, 96:04, CO_2:MeOH, 2.5 mL min^{-1}, 40 °C) tR = 12.01 (minor), 12.60 (major) minutes. $[\alpha]_D^{25}$ = -22.2 (c 0.5, $CHCl_3$).

(S)- 6-chloro-2'-fluoro-6'-(methylamino)-[1,1'-biphenyl]-2-ol (**3s**)

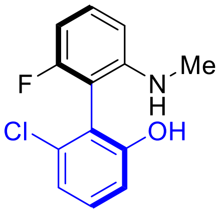

General procedure **F** was performed with 2-bromo-3-fluoro-N-methylaniline (20.4 mg, 0.1 mmol) and 2-hydroxy-6-chlorophenylboronic acid pinacol ester (76.4 mg, 0.3 mmol), at 60 °C. The product was purified by column chromatography (5-15% EtOAc:petrol) to yield the product as an oil (16.3 mg, 0.065 mmol, 65%, 99% ee). **^{1}H NMR** (400 MHz, Chloroform-*d*) δ 7.36 (td, *J* = 8.3, 6.5 Hz, 1H), 7.32 – 7.26 (m, 1H), 7.15 (dd, *J* = 8.0, 1.1 Hz, 1H), 6.99 (dd, *J* = 8.2, 1.2 Hz, 1H), 6.72 – 6.52 (m, 2H), 5.11 (br s, 1H), 3.62 (br s, 1H), 2.84 (s, 3H). **^{19}F NMR** (376 MHz, $CDCl_3$) δ -111.93. **^{13}C NMR** (126 MHz, $CDCl_3$) 161.0 (d, *J* = 243.8 Hz), 155.00, 148.5 (d, *J* = 5.8 Hz), 135.6, 131.7 (d, *J* = 10.6 Hz), 130.7, 122.0, 117.5, 114.5, 105.0 (d, *J* = 20.1 Hz), 104.3 (d, *J* = 22.5 Hz), 30.7. **HRMS** [M+H]$^+$ calcd for $C_{13}H_{11}ClFNO$; 251.0513, found 251.0511, Δ= -1.5 ppm. **Chiral SFC Analysis** (IC-3, 96:04 CO_2:MeOH, 2.5 mL/min, 4.97 min [major], 5.83 min [minor]). $[\alpha]_D^{25}$ = +25.7 (c 0.09, $CHCl_3$).

(R)-6-fluoro-2'-methyl-6'-(methylamino)-[1,1'-biphenyl]-2-ol (**3t**)

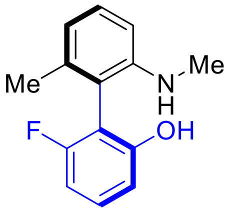

General procedure **F** was performed with 2-bromo-N,3-dimethylaniline (20.0 mg, 0.1 mmol) and 2-hydroxy-6-fluorophenylboronic acid pinacol ester (71.4 mg, 0.3 mmol), at 60 °C. The product was purified by column chromatography (5-15% EtOAc:petrol) to yield the product as an oil (18.0 mg, 0.078 mmol, 78%, 99% ee). **^{1}H NMR** (400 MHz, $CDCl_3$) δ 7.37 – 7.24 (m, 2H), 6.89 (dt, *J* = 8.3, 1.0 Hz, 1H), 6.84 – 6.72 (m, 2H), 6.64 (d, *J* = 8.2 Hz, 1H), 5.21 (br s, 1H), 3.53 (br s, 1H), 2.80 (s, 3H), 2.05 (s, 3H). **^{19}F NMR** (376 MHz, $CDCl_3$) δ -112.65. **^{13}C NMR** (176 MHz, $CDCl_3$) δ 160.6 (d, *J* = 245.5 Hz), 154.7 (d, *J* = 6.2 Hz), 147.5, 139.2, 130.3, 130.1 (d, *J* = 10.4 Hz), 119.4, 113.3, 111.6 (d, *J* = 21.4 Hz), 111.6 (d, *J* = 3.3 Hz), 107.9 (d, *J* = 22.7 Hz), 107.9, 30.8, 20.0. **HRMS** [M+H]$^+$ calcd for $[C_{14}H_{15}FNO]^+$ 232.1132, found 232.1132 Δ= -0.1 ppm. **Chiral HPLC Analysis** (IG-3, 98:02, Hexane : ^{i}PrOH, 1.25 mL/min, 5.33 min [major], 6.41 min [minor]). $[\alpha]_D^{25}$ = +6.0 (c 0.07, $CHCl_3$).

(S)- 6-chloro-2'-(ethylamino)-6'-fluoro-[1,1'-biphenyl]-2-ol (**3u**)

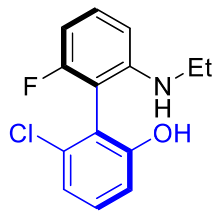

General procedure **F** was performed with 2-bromo-N-ethyl-3-fluoroaniline (21.8 mg, 0.1 mmol) and 2-hydroxy-6-chlorophenylboronic acid pinacol ester (76.4 mg, 0.3 mmol), at 60 °C. The product was purified by column chromatography (5-15% EtOAc:petrol) to yield the product as an oil (12.8 mg, 0.048 mmol, 48%, 98% ee). **^{1}H NMR** (400 MHz, $CDCl_3$) δ 7.37 – 7.26 (m, 2H), 7.15 (dd, *J* = 8.0, 1.1 Hz, 1H), 7.00 (dd, *J* = 8.3, 1.2 Hz, 1H), 6.58 (m, 2H), 5.19 (br s, 1H), 3.54 (br s, 1H), 3.19 (q, *J* = 7.2 Hz, 2H), 1.19 (t, *J* = 7.1 Hz, 3H). **^{19}F NMR** (376 MHz, $CDCl_3$) δ -111.61. **^{13}C NMR** (126 MHz, $CDCl_3$) δ 161.07 (d, *J* = 245.2 Hz), 155.0, 147.6, 135.6, 131.6 (d, *J* = 10.6 Hz), 130.7, 122.0, 117.5, 114.6, 106.6, 105.2 (d, *J* = 19.9 Hz), 104.2 (d, *J* = 22.5 Hz), 38.5, 14.6. **HRMS** $[M+H]^+$ calcd for $C_{14}H_{14}ClFNO$; 266.0742, found 266.0742, Δ= -0.2 ppm. **Chiral SFC Analysis** (IG-3, 94:06 CO_2:MeOH, 2.5 mL/min, 4.05 min [major], 4.76 min [minor]). $[\alpha]_D^{25}$ = +5.9 (c 0.2, $CHCl_3$).

R)-2'-(ethylamino)-6-fluoro-6'-methyl-[1,1'-biphenyl]-2-ol (**3v**)

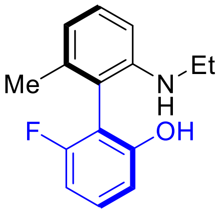

General procedure **F** was performed with 2-bromo-N-ethyl-3-methylaniline (21.4 mg, 0.1 mmol) and 2-hydroxy-6-fluorophenylboronic acid pinacol ester (71.4 mg, 0.3 mmol), at 60 °C. The product was purified by column chromatography (5-15% EtOAc:petrol) to yield the product as an oil (15.7 mg, 0.064 mmol, 64%, 98% ee). *After isolation the product decomposes in solution at r.t. which gives rise to extra peaks visible in the NMR spectra.* **^{1}H NMR** (400 MHz, $CDCl_3$) δ 7.38 – 7.29 (m, 1H), 7.26 (d, *J* = 7.9 Hz, 1H), 6.90 (dt, *J* = 8.3, 1.0 Hz, 1H), 6.80 (td, *J* = 8.5, 1.1 Hz, 1H), 6.76 (d, *J* = 7.7 Hz, 1H), 6.68 (d, *J* = 8.3 Hz, 1H), 3.15 (q, *J* = 7.1 Hz, 2H), 2.05 (s, 3H), 1.16 (t, *J* = 7.1 Hz, 3H). **^{19}F NMR** (376 MHz, $CDCl_3$) δ -112.67. **^{13}C NMR** (176 MHz, $CDCl_3$) δ 160.5 (d, *J* = 245.5 Hz), 154.7 (d, *J* = 6.0 Hz), 146.3, 139.4, 130.2, 130.1 (d, *J* = 10.3 Hz), 119.6, 111.7 (d, *J* = 3.3 Hz), 111.7 (d, *J* = 20.8 Hz), 113.8, 109.0, 107.9 (d, *J* = 22.6 Hz), 38.6, 20.0, 14.6. **HRMS** $[M+H]^+$ calcd for $[C_{15}H_{17}FNO]^+$ 246.1289, found 246.1286 Δ= -1.3 ppm. **Chiral HPLC Analysis** (IG-3, 98:02 Hexane : ^{i}PrOH, 1.25 mL/min, 4.06 min [major], 4.99 min [minor]). $[\alpha]_D^{25}$ = +30.0 (c 0.02, $CHCl_3$).

(S)- 2'-(benzylamino)-6-chloro-6'-fluoro-[1,1'-biphenyl]-2-ol (**3w**)

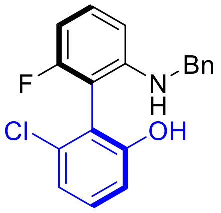

General procedure **F** was performed with N-benzyl-2-bromo-3-fluoroaniline (28.0 mg, 0.1 mmol) and 2-hydroxy-6-chlorophenylboronic acid pinacol ester (76.4 mg, 0.3 mmol), at 60 °C. The product was purified

by column chromatography (5-15% EtOAc:petrol) to yield the product as an oil (12.8 mg, 0.035 mmol, 35%, 93% ee). ^{1}**H NMR** (400 MHz, $CDCl_3$) δ 7.41 – 7.22 (m, 7H), 7.16 (dd, *J* = 8.1, 1.1 Hz, 1H), 7.01 (dd, *J* = 8.2, 1.1 Hz, 1H), 6.64 – 6.56 (m, 1H), 6.51 (d, *J* = 8.3 Hz, 1H) 5.20 (br s, 1H), 4.38 (s, 2H), 4.11 (br s, 1H). ^{19}**F NMR** (376 MHz, $CDCl_3$) δ -111.72. ^{13}**C NMR** (101 MHz, $CDCl_3$) δ 161.06 (d, *J* = 245.2 Hz), 155.0, 147.3 (d, *J* = 5.7 Hz), 138.5, 135.6, 131.6 (d, *J* = 10.8 Hz), 130.8, 128.7, 127.3, 126.9, 122.0, 117.3, 116.5, 114.5, 108.5 (d, *J* = 22.2 Hz), 106.9 (d, *J* = 2.7 Hz), 105.2, 104.5 (d, *J* = 22.5 Hz), 47.8. **HRMS** $[M+H]^+$ calcd for $C_{19}H_{16}ClFNO$; 328.0899, found 328.0900, Δ= +0.4 ppm. **Chiral SFC Analysis** (IC-3, 94:06, CO_2:MeOH, 2.5 mL/min, 5.66 min [major], 6.88 min [minor]). $[\alpha]_D^{25}$ = -19.7 (c 0.1, $CHCl_3$).

(R)-6-fluoro-2'-methyl-6'-nitro-[1,1'-biphenyl]-2-ol (**3y**)

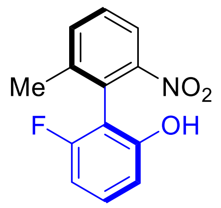

2-bromo-1-methyl-3-nitrobenzene (21.2 mg, 0.1 mmol), 2- hydroxy-6-fluorophenylboronic acid pinacol ester (71.4 mg, 0.3 mmol), Na_3PO_4 (49.2 mg, 0.30 mmol), *(R)*- sRuPhos (3.3 mg, 6 mol%) and $Pd(OAc)_2$ (1.1 mg, 5 mol%) were added to a 4 mL crimp vial. After 3 evacuation-backfill cycles with nitrogen, toluene (0.475 mL) and water (o.o25 mL) were added. The reaction mixture was stirred at 60 °C for 16 h and then the solvent was removed under a stream of air. The product was purified by column chromatography (0-20% EtOAc:petrol) to yield the product (mg, 0.045 mmol, 45%, 86% ee). ^{1}**H NMR** (400 MHz, $CDCl_3$) δ 7.88 (d, *J* = 8.1 Hz, 1H), 7.62 (d, *J* = 7.7 Hz, 1H), 7.51 (t, *J* = 7.9 Hz, 1H), 7.36 – 7.24 (m, 1H), 6.79 (dd, *J* = 8.2, 2.2 Hz, 2H). ^{19}**F NMR** (376 MHz, $CDCl_3$) δ -113.58. ^{13}**C NMR** (176 MHz, $CDCl_3$) δ 159.7 (d, *J* = 245.1 Hz), 153.7 (d, *J* = 6.3 Hz), 150.8, 141.1, 134.6, 130.3 (d, *J* = 10.3 Hz), 129.3, 124.6 (d, *J* = 1.5 Hz), 122.0, 111.8 (d, *J* = 20.1 Hz), 111.7 (d, *J* = 3.3 Hz), 108.1 (d, *J* = 22.1 Hz), 20.0. **HRMS** $[M+Na]^+$ calcd for $C_{13}H_9FNO_3^-$; 246.0566, found 246.0560, Δ = -2.4 ppm. **Chiral SFC Analysis** (SC-3, 95:05 CO_2:MeOH, 2.5 mL/min, 8.12 min [minor], 9.92 min [major]). $[\alpha]_D^{25}$ = +3.2 (c. 0.13 $CHCl_3$).

(R)-2-(2-aminonaphthalen-1-yl)-3-fluorophenol (**3z**)

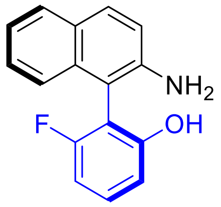

1-bromonaphthalen-2-amine (22.2 mg, 0.1 mmol), 2-hydroxy-6-fluorophenylboronic acid pinacol ester (71.4 mg, 0.3 mmol), Na_3PO_4 (49.2 mg, 0.30 mmol), *(R)*- sRuPhos (3.3 mg, 6 mol%) and $Pd(OAc)_2$ (1.1 mg, 5 mol%) were added to a 4 mL crimp vial. After 3 evacuation-backfill cycles with nitrogen, toluene (0.5 mL) was added. The reaction mixture was stirred at 80 °C for 40 h and then the solvent was removed under a stream of air. The product was purified by column chromatography (0-20% EtOAc:petrol) to yield the product (12.9 mg, 0.051 mmol, 51%, 94% ee). ^{1}**H NMR** (700 MHz, $CDCl_3$) δ 7.80 (dd, *J* = 17.0, 8.4 Hz, 2H), 7.43 – 7.35 (m, 2H), 7.31 (ddd, *J* = 8.0, 6.8, 1.2 Hz, 1H), 7.28 (d, *J* = 8.9 Hz, 1H), 7.10 (d, *J* = 8.8 Hz, 1H), 6.97 (d, *J* = 8.4 Hz, 1H), 6.88 (td, *J* = 8.5, 1.0 Hz, 1H). ^{19}**F NMR** (471 MHz, $CDCl_3$) δ -111.72. ^{13}**C NMR** (176 MHz,

$CDCl_3$) δ 161.1 (d, *J* = 246.4 Hz), 155.3 (d, *J* = 6.0 Hz), 143.1, 133.6, 131.0, 130.5 (d, *J* = 10.4 Hz), 128.3, 128.3, 127.5, 123.3, 123.0, 118.1, 111.8 (d, *J* = 3.3 Hz), 110.5 (d, *J* = 20.5 Hz), 108.0 (d, *J* = 22.4 Hz), 105.5. **HRMS** $[M+H]^+$ calcd for $C_{16}H_{13}FNO$; 254.0976, found 254.0976, Δ= +0.0 ppm. **Chiral SFC Analysis** (IJ-3, 87:13 CO_2:MeOH, 2.5 mL/min, 10.01 min [minor], 12.22 min [major]). $[\alpha]_D^{25}$ = +46.3 (c. 0.42 $CHCl_3$).

(R)-3-chloro-2-(6-methylindolin-7-yl)phenol (**3za**)

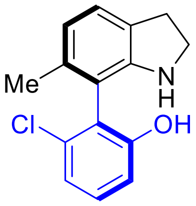

7-bromo-6-methylindoline (21.2 mg, 0.1 mmol), 2- hydroxy-6-chlorophenylboronic acid pinacol ester (76.4 mg, 0.3 mmol), Na_3PO_4 (49.2 mg, 0.30 mmol), *(R)*- sRuPhos (3.3 mg, 6 mol%) and $Pd(OAc)_2$ (1.1 mg, 5 mol%) were added to a 4 mL crimp vial. After 3 evacuation-backfill cycles with nitrogen, toluene (0.5 mL) was added. The reaction mixture was stirred at 60 °C for 40 h and then the solvent was removed under a stream of air. The product was purified by column chromatography (0-20% EtOAc:petrol) to yield the product (24.4 mg, 0.045 mmol, 94%, 99% ee). ^{1}**H NMR** (700 MHz, $CDCl_3$) δ 7.24 (t, *J* = 8.1 Hz, 1H), 7.11 (ddd, *J* = 16.8, 7.7, 1.1 Hz, 2H), 6.96 (dd, *J* = 8.2, 1.1 Hz, 1H), 6.72 (dd, *J* = 7.4, 0.8 Hz, 1H), 3.57 (dd, *J* = 9.2, 7.7 Hz, 2H), 3.12 (qt, *J* = 15.5, 8.4 Hz, 2H), 2.05 (s, 3H). ^{13}**C NMR** (176 MHz, $CDCl_3$) δ 154.4, 150.8, 136.7, 134.4, 129.7, 127.3, 125.2, 123.1, 121.6, 120.5, 114.2, 113.2, 47.4, 29.9, 19.1. **HRMS** $[M+H]^+$ calcd for $C_{15}H_{15}ClO$; 260.0837, found 260.0838, Δ= +0.6 ppm. **Chiral SFC Analysis** (IJ-3, 96:04 CO_2:MeOH, 2.5 mL/min, 7.12 min [minor], 9.03 min [major]). $[\alpha]_D^{25}$ = -15.8 (c. 0.63 $CHCl_3$).

(R)-3-chloro-2-(6-methyl-1H-indol-7-yl)phenol (**3zb**)

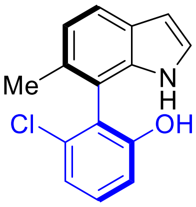

7-bromo-6-methyl-1H-indole (21.0 mg, 0.1 mmol), 2-hydroxy-6-chlorophenylboronic acid pinacol ester (76.4 mg, 0.3 mmol), Na_3PO_4 (49.2 mg, 0.30 mmol), *(R)*- sRuPhos (3.3 mg, 6 mol%) and $Pd(OAc)_2$ (1.1 mg, 5 mol%) were added to a 4 mL crimp vial. After 3 evacuation-backfill cycles with nitrogen, toluene (0.5 mL) was added. The reaction mixture was stirred at 60 °C for 40 h and then the solvent was removed under a stream of air. The product was purified by column chromatography (0-20% EtOAc:petrol) to yield the product (15.9 mg, 0.062 mmol, 62%, 80% ee). ^{1}**H NMR** (700 MHz, MeOD) δ 7.49 (d, *J* = 8.0 Hz, 1H), 7.25 (t, *J* = 8.1 Hz, 1H), 7.11 – 7.04 (m, 2H), 6.99 (d, *J* = 8.2 Hz, 1H), 6.93 (d, *J* = 9.3 Hz, 1H), 6.43 (d, *J* = 3.1 Hz, 1H), 2.16 (s, 3H). ^{13}**C NMR** (176 MHz, MeOD) δ 156.3, 135.3, 135.1, 135.1, 129.2, 129.0, 126.3, 123.8, 121.1, 120.3, 119.3, 117.9, 113.8, 100.8, 17.9. **HRMS** $[M+H]^+$ calcd for $C_{15}H_{14}ClNO^+$; 259.0557, found 259.0557, Δ= +0.0 ppm. **Chiral HPLC Analysis** (IG-3, 98:02 CO_2:MeOH, 2.5 mL/min, 7.29 min [minor], 8.45 min [major]). $[\alpha]_D^{25}$ = -35.3 (c. 0.13 MeOH).

(S)-2-(2-amino-4-chloropyridin-3-yl)-3-fluorophenol (**3zc**)

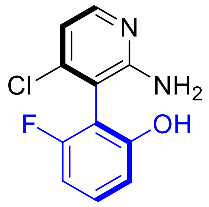

3-bromo-4-chloropyridin-2-amine (20.7 mg, 0.1 mmol), 2-hydroxy-6-fluorophenylboronic acid pinacol ester (71.4 mg, 0.3 mmol), Na_3PO_4 (49.2 mg, 0.30 mmol), *(R)*- sRuPhos (3.3 mg, 6 mol%) and $Pd(OAc)_2$ (1.1 mg, 5 mol%) were added to a 4 mL crimp vial. After 3 evacuation-backfill cycles with nitrogen, toluene (0.5 mL) was added. The reaction mixture was stirred at 60 °C for 40 h and then the solvent was removed under a stream of air. The product was purified by column chromatography (30-100% EtOAc:petrol) to yield the product (8.8 mg, 0.037 mmol, 37%, 89% ee). **^{1}H NMR** (400 MHz, MeOD) δ 7.89 (d, *J* = 5.6 Hz, 1H), 7.30 (td, *J* = 8.3, 6.7 Hz, 1H), 6.81 (d, *J* = 5.6 Hz, 1H), 6.80 – 6.76 (m, 1H), 6.72 (ddd, *J* = 9.2, 8.3, 1.0 Hz, 1H). **^{19}F NMR** (376 MHz, MeOD) δ -115.16. **^{13}C NMR** (176 MHz, MeOD) δ 161.0 (d, *J* = 244.3 Hz), 158.7, 156.7 (d, *J* = 6.8 Hz), 147.0, 145.5, 130.5 (d, *J* = 10.9 Hz), 113.7, 111.8 (d, *J* = 1.3 Hz), 111.3 (d, *J* = 3.2 Hz), 109.2 (d, *J* = 19.5 Hz), 106.0 (d, *J* = 22.5 Hz). **HRMS** $[M+H]^+$ calcd for $C_{11}H_9ClFN_2O+$; 239.0382, found 239.0382, Δ= +0.0 ppm. **Chiral SFC Analysis** (IC-3, 90:10 CO_2:MeOH, 2.5 mL/min, 4.09 min [major], 5.97. min [minor]). $[\alpha]_D^{25}$ = +37.1 (c. 0.53 MeOH).

Performing this reaction at elevated temperatures caused an increase in yield but at a cost of %ee.

Temperature	Yield	Ee
60 °C	37%	89%
80 °C	60%	71%
100 °C	74%	11%

Application of *(R)*-sRuPhos to formation of 2,2'-biphenols and comparisons with *(R)*-sSPhos (Scheme 2A)

General Procedure G: asymmetric Suzuki cross-coupling to form biphenols using *(R)*-sRuPhos

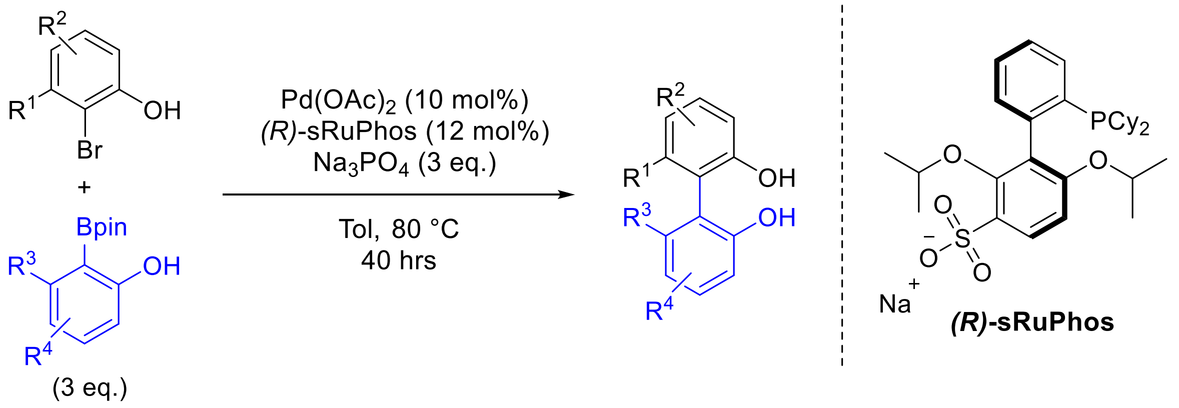

Aryl bromide (0.10 mmol), aryl boronic ester (0.30 mmol, 3 eq.), Na_3PO_4 (49.2 mg, 0.30 mmol), (*R*)- sRuPhos (6.6 mg, 12 mol%) and $Pd(OAc)_2$ (2.2 mg, 10 mol%) were added to a 4 mL crimp vial. After 3 evacuation-backfill cycles with nitrogen, toluene (0.5 mL) was added. The reaction mixture was stirred at 80 °C for 40 h and then the solvent was removed under a stream of air. The product was purified via column chromatography (petrol/EtOAc).

General Procedure H: asymmetric Suzuki cross-coupling to form biphenols using *(R)*-sSPhos

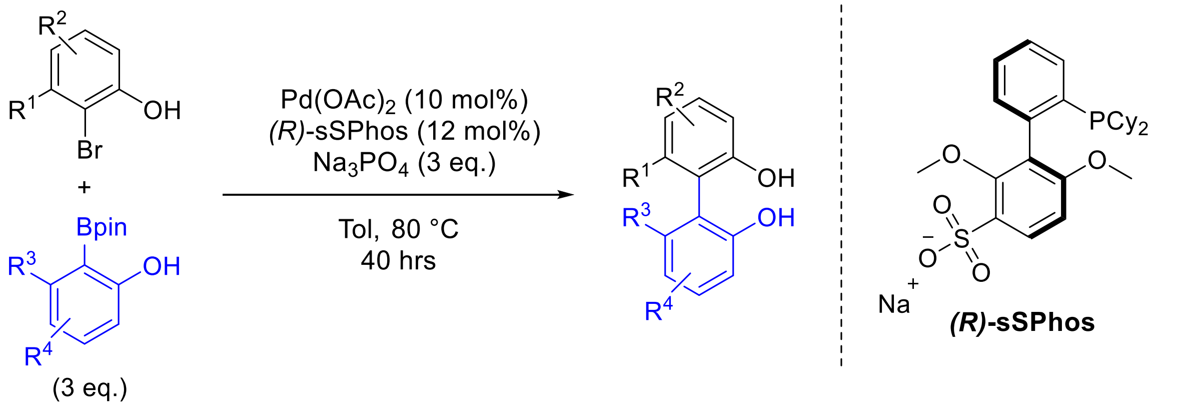

Aryl bromide (0.10 mmol), aryl boronic ester (0.30 mmol, 3 eq.), Na_3PO_4 (49.2 mg, 0.30 mmol), (*R*)- sSPhos (6.2 mg, 12 mol%) and $Pd(OAc)_2$ (2.2 mg, 10 mol%) were added to a 4 mL crimp vial. After 3 evacuation-backfill cycles with nitrogen, toluene (0.5 mL) was added. The reaction mixture was stirred at 80 °C for 40 h and then the solvent was removed under a stream of air. The product was purified via column chromatography (petrol/EtOAc).

(S)-6,6'-dichloro-[1,1'-biphenyl]-2,2'-diol (**4a**)

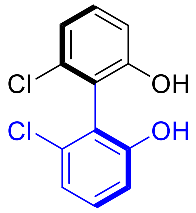

General procedure **G** was performed with 2-bromo-3-chlorophenol (20.7 mg, 0.1 mmol) and 2- hydroxy-6-chlorophenylboronic acid pinacol ester (76.4 mg, 0.3 mmol). The product was purified by column chromatography (10–20% EtOAc: petrol) to yield the title compound (24.5 mg, 0.096 mmol, 96%, 98% ee). **^{1}H NMR** (700 MHz, $CDCl_3$) δ 7.33 (t, *J* = 8.2 Hz, 2H), 7.16 (dd, *J* = 8.1, 1.1 Hz, 2H), 6.99 (dd, *J* = 8.3, 1.1 Hz, 2H), 4.87 (s, 2H). **^{13}C NMR** (176 MHz, $CDCl_3$) δ 155.1, 135.5, 131.6, 122.3, 118.8, 114.8. **Chiral SFC Analysis** (IG-3, CO_2:MeOH 90:10, 2.5 mL min^{-1} , 40 °C) tR = 8.90 (major), 10.16 (minor) minutes. $[\alpha]_D^{25}$ = -9.7 (c 1.15, $CHCl_3$).

General procedure **H** was performed with 2-bromo-3-chlorophenol (20.7 mg, 0. 1 mmol) and 2- hydroxy-6-chlorophenylboronic acid pinacol ester (76.4 mg, 0.3 mmol), to yield the title compound (5.3 mg, 0.021 mmol, 21%, 98% ee).

Data in accordance with literature.[6]

(S)-6,6'-dimethyl-[1,1'-biphenyl]-2,2'-diol (**4b**)

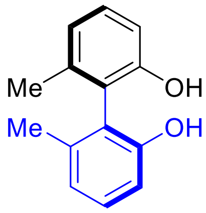

General procedure **G** was performed with 2-bromo-3-methylphenol (18.7 mg, 0.1 mmol) 2- hydroxy-6-methylphenylboronic acid pinacol ester (70.2 mg, 0.3 mmol), at 80 °C. The product was purified by column chromatography (5-20% EtOAc:petrol) to yield the product as a white solid (8.56 mg, 0.040 mmol, 40%, 90% ee). **^{1}H NMR** (500 MHz, $CDCl_3$) δ 7.31 – 7.27 (m, 2H), 6.96 (dt, *J* = 7.6, 1.0 Hz, 2H), 6.93 (d, *J* = 8.2 Hz, 2H), 4.70 (s, 2H), 2.04 (s, 6H). **^{13}C NMR** (176 MHz, $CDCl_3$) δ 153.8, 138.9, 130.1, 122.6, 119.5, 113.1, 19.5. **Chiral HPLC Analysis** (SB-3, 96:04 Hexane : ^{i}PrOH, 1.25 mL/min, 10.54 min [major], 14.51 min [minor]). $[\alpha]_D^{25}$ = -47.9 (c 0.21, $CHCl_3$).

General procedure **H** was performed with 2-bromo-3-methylphenol (18.7 mg, 0.1 mmol) and 2- hydroxy-6-methylphenylboronic acid pinacol ester (70.2 mg, 0.3 mmol). This gave only traces (<5%) of product, as determined by comparison of the crude NMR.

Data in accordance with literature value of $[\alpha]_D$ = -60.5 (c 1.00, $CHCl_3$), for enantiomerically pure product.[14]

(R)-1-(2,3-difluoro-6-hydroxyphenyl)-5,6,7,8-tetrahydronaphthalen-2-ol (**4c**)

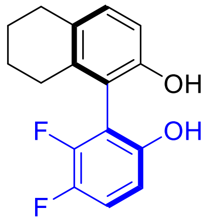

General procedure **G** was performed 1-bromo-5,6,7,8-tetrahydronaphthalen-2-ol (22.7 mg, 0. 1 mmol) and 2- hydroxy-5,6-difluorophenylboronic acid pinacol ester (76.8 mg, 0.3 mmol). The product was purified by column chromatography (10–20% EtOAc: petrol) to yield the title compound, (12.1 mg, 0.044 mmol, 44%, 95% ee). **^{1}H NMR** (400 MHz, MeOD) δ 7.05 (dt, *J* = 10.3, 9.0 Hz, 1H), 6.95 (d, *J* = 8.3 Hz, 1H), 6.69 (d, *J* = 8.3 Hz, 1H), 6.64 (ddd, *J* = 9.1, 4.0, 2.0 Hz, 1H), 2.75 (t, *J* = 6.2 Hz, 2H), 2.37 (ddt, *J* = 56.6, 16.9, 6.2 Hz, 2H), 1.73 (dddd, *J* = 11.7, 6.5, 4.4, 2.0 Hz, 4H). **^{13}C NMR** (176 MHz, $CDCl_3$) δ 151.7, 149.9 – 149.6 (m), 148.0 (dd, *J* = 247.2, 13.6 Hz), 145.4 (dd, *J* = 241.4, 13.4 Hz), 138.3, 132.2, 130.8, 117.7 (d, *J* = 18.7 Hz), 113.8 (d, *J* = 2.1 Hz), 113.7, 111.8 (d, *J* = 17.5 Hz), 110.8 (dd, *J* = 6.4, 3.8 Hz), 29.3, 27.3, 23.0, 22.9. **^{19}F NMR** (376 MHz, $CDCl_3$) δ -136.2 (d, *J* = 22.6 Hz), -147.9 (d, *J* = 22.6 Hz). **HRMS**: $[M-H]^-$ calculated for $[C_{16}H_{13}F_2O_2]^-$ 275.0889, found 275.0893. Δ = 1.3 ppm. **Chiral SFC Analysis** (IC-3, CO_2:MeOH 94:06, 2.5 mL min^{-1} , 40 °C) tR = 5.25 (minor), 7.99 (major) minutes. $[\alpha]_D^{25}$ = +1.0 (c 0.61, $CHCl_3$).

General procedure **H** was performed with 1-bromo-5,6,7,8-tetrahydronaphthalen-2-ol (22.7 mg, 0. 1 mmol) and 2- hydroxy-5,6-difluorophenylboronic acid pinacol ester (76.8 mg, 0.3 mmol). This gave only traces (<5%) of product, as determined by comparison of the crude NMR.

(R)-5,6'-dichloro-6-methyl-[1,1'-biphenyl]-2,2'-diol (**4d**)

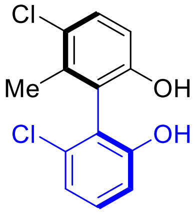

General procedure **G** was performed with 2-bromo-4-chloro-3-methylphenol (22.1 mg, 0. 1 mmol) and 2-hydroxy-6-chlorophenylboronic acid pinacol ester (76.4 mg, 0.3 mmol). The product was purified by column chromatography (10–20% EtOAc: petrol) to yield the title compound, (8.1 mg, 0.030 mmol, 30%, 98% ee). SFC analysis revealed coelution with trace amounts of the homocoupled byproduct **4a**. **^{1}H NMR** (700 MHz, $CDCl_3$) δ 7.41 (d, *J* = 8.7 Hz, 1H), 7.34 (t, *J* = 8.2 Hz, 1H), 7.17 (dd, *J* = 8.1, 1.1 Hz, 1H), 7.01 (dd, *J* = 8.3, 1.1 Hz, 1H), 6.90 (dd, *J* = 8.8, 0.7 Hz, 1H), 4.82 (s, 1H), 4.66 (s, 1H), 2.11 (s, 3H). **^{13}C NMR** (176 MHz, $CDCl_3$) δ 154.7, 152.4, 137.0, 135.2, 131.3, 131.3, 126.8, 122.2, 120.0, 119.7, 114.8, 114.6, 17.2. **HRMS**: $[M-H]^-$ calculated for $[C_{13}H_9Cl_2O_2]^-$ 266.9985, found 266.9974. Δ = -4.3 ppm. **Chiral SFC Analysis** (IG-3, CO_2:MeOH 90:10, 2.5 mL min^{-1} , 40 °C) tR = 7.58 (major), 9.27 (minor) minutes. $[\alpha]_D^{25}$ = +36.0 (c 0.33, $CHCl_3$).

General procedure **H** was performed with 2-bromo-4-chloro-3-methylphenol (22.1 mg, 0. 1 mmol) and 2-hydroxy-6-chlorophenylboronic acid pinacol ester (76.4 mg, 0.3 mmol). This gave only traces (<5%) of product, as determined by comparison of the crude NMR.

Application of *(R)*-sRuPhos to formation of mono-O-methylated-2,2'-biphenols and comparisons with *(R)*-sSPhos (Scheme 2B)

(R)-6-fluoro-2'-methoxy-6'-methyl-[1,1'-biphenyl]-2-ol (**4e**)

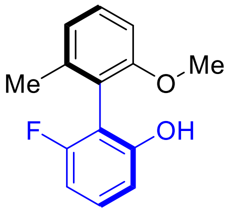

2-bromo-1-methoxy-3-methylbenzene (20.1 mg, 0.1 mmol), 2-hydroxy-6-fluorophenylboronic acid pinacol ester (71.4 mg, 0.3 mmol, 3 equiv.), Na_3PO_4 (49.2 mg, 0.30 mmol, 3 equiv.), (*R*)- sRuPhos (3.3 mg, 6 mol%) and $Pd(OAc)_2$ (1.1 mg, 5 mol%) were added to a 4 mL crimp vial. After 3 evacuation-backfill cycles with nitrogen, toluene (0.475 mL) and water (0.025 mL) were added. The reaction mixture was stirred at 40 °C for 16 h and then the solvent was removed under a stream of air. The product was purified by column chromatography (5-15% EtOAc:petrol) to yield the product as a white solid (18.6 mg, 0.080 mmol, 80%, 94% ee). **^{1}H NMR** (500 MHz, $CDCl_3$) δ 7.36 (t, *J* = 8.0 Hz, 1H), 7.32 – 7.19 (m, 1H), 7.00 (d, *J* = 7.6 Hz, 1H), 6.90 (d, *J* = 8.3 Hz, 1H), 6.84 (dt, *J* = 8.2, 1.0 Hz, 1H), 6.76 (td, *J* = 8.6, 1.0 Hz, 1H), 4.90 (s, 1H), 3.78 (s, 3H), 2.13 (s, 3H). **^{13}C NMR** (126 MHz, $CDCl_3$) δ 160.2 (d, *J* = 244.4 Hz), 157.7, 154.4 (d, *J* = 6.3 Hz), 140.3, 130.0, 129.3 (d, *J* = 10.4 Hz), 122.9, 117.7, 112.1 (d, *J* = 20.7 Hz), 111.1 (d, *J* = 3.0 Hz), 108.6, 107.3 (d, *J* = 22.8 Hz), 55.9, 19.6. **Chiral SFC Analysis** (IG-3, 98:02 CO_2:MeOH, 2.5 mL/min, 5.68 min (major), 6.42 min (minor)). $[\alpha]_D^{25}$ = +42.0 (c 0.02, $CHCl_3$).

As reported in our prior work,[6] the same procedure was repeated but with (R)-sSPhos to yield the product (18.6 mg, 0.08 mmol, 67%, 85% ee).

Data in accordance with literature.[6]

(S)-2'-chloro-6-fluoro-6'-methoxy-[1,1'-biphenyl]-2-ol (**4f**)

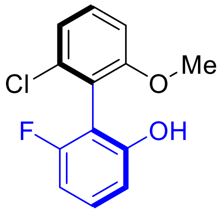

2-bromo-1-chloro-3-methoxybenzene (22.1 mg, 0. 1 mmol), 2- hydroxy-6-fluorophenylboronic acid pinacol ester (71.4 mg, 0.3 mmol), Na_3PO_4 (49.2 mg, 0.30 mmol, 3 equiv.), (*R*)- sRuPhos (3.3 mg, 6 mol%) and $Pd(OAc)_2$ (1.1 mg, 5 mol%) were added to a 4 mL crimp vial. After 3 evacuation-backfill cycles with nitrogen, toluene (0.475 mL) and water (0.025 mL) were added. The reaction mixture was stirred at 40 °C for 16 h and then the solvent was removed under a stream of air. The product was purified by column chromatography (5–10% EtOAc: petrol) to yield the title compound, (22.5 mg, 0.089 mmol, 89%, 91% ee). **^{1}H NMR** (500 MHz, $CDCl_3$) δ 7.37 (t, *J* = 8.3 Hz, 1H), 7.28 (td, *J* = 8.3, 6.5 Hz, 1H), 7.18 (dd, *J* = 8.1, 1.0 Hz, 1H), 6.94 (dd, *J* = 8.4, 1.0 Hz, 1H), 6.82 (dt, *J* = 8.3, 1.0 Hz, 1H), 6.79 – 6.73 (m, 1H), 4.87 (s, 1H), 3.79 (s, 3H). **^{13}C NMR** (126 MHz, $CDCl_3$) δ 160.5 (d, *J* = 246.0 Hz), 158.9, 154.5 (d, *J* = 5.9 Hz), 136.5 (d, *J* = 0.8 Hz), 131.0, 130.2 (d, *J* = 10.5 Hz), 122.4, 118.3 (d, *J* = 0.7 Hz), 111.5 (d, *J* = 3.1 Hz), 111.1 (d, *J* = 20.1 Hz), 109.8, 107.7 (d, *J* = 22.5 Hz), 56.4. **^{19}F NMR** (471 MHz, $CDCl_3$) δ -112.6. **HRMS** $[M-H]^-$ calculated for $[C_{13}H_9ClFO_2]^-$ 251.0281, found 251.0290. Δ

= 3.8 ppm. **Chiral SFC Analysis** (IC-3, CO_2:MeOH 96:04, 2.5 mL min^{-1}, 40 °C) tR = 5.68 (major), 6.25 (minor) minutes. $[\alpha]_D^{25}$ = +15.0 (c 0.1, $CHCl_3$).

The same procedure was repeated but with *(R)*-sSPhos to yield the product (12.1 mg, 0.048 mmol, 48%, 78% ee).

(R)-3-fluoro-2-(2-methoxy-5,6,7,8-tetrahydronaphthalen-1-yl)phenol (**4g**)

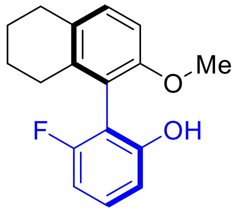

5-bromo-6-methoxy-1,2,3,4-tetrahydronaphthalene (22.7 mg, 0. 1 mmol), 2- hydroxy-6-fluorophenylboronic acid pinacol ester (71.4 mg, 0.3 mmol), Na_3PO_4 (49.2 mg, 0.30 mmol), (*R*)- sRuPhos (3.3 mg, 6 mol%) and $Pd(OAc)_2$ (1.1 mg, 5 mol%) were added to a 4 mL crimp vial. After 3 evacuation-backfill cycles with nitrogen, toluene (0.5 mL) was added. The reaction mixture was stirred at 60 °C for 40 h and then the solvent was removed under a stream of air. The product was purified by column chromatography ($CHCl_3$) to yield the title compound, (14.5 mg, 0.053 mmol, 53%, 94% ee). **^{1}H NMR** (700 MHz, $CDCl_3$) δ 7.23 (td, *J* = 8.2, 6.5 Hz, 1H), 7.16 (d, *J* = 8.5 Hz, 1H),), 6.85 – 6.80 (m, 2H), 6.73 (td, *J* = 8.5, 1.0 Hz, 1H), 4.86 (s, 1H), 3.73 (s, 3H), 2.84 – 2.72 (m, 2H), 2.47 – 2.39 (m, 1H), 2.32 (dt, *J* = 16.9, 5.8 Hz, 1H), 1.79 – 1.69 (m, 3H), 1.66 (tdd, *J* = 9.3, 6.3, 4.3 Hz, 1H). **^{13}C NMR** (176 MHz, $CDCl_3$) δ 160.2 (d, *J* = 244.0 Hz), 155.6, 154.3 (d, *J* = 6.4 Hz), 138.9, 131.2, 130.5, 129.3 (d, *J* = 10.3 Hz), 116.9, 112.2 (d, *J* = 20.9 Hz), 110.9 (d, *J* = 3.2 Hz), 109.0, 107.4 (d, *J* = 22.7 Hz), 56.0, 29.2, 27.1, 22.9, 22.9. **^{19}F NMR** (376 MHz, $CDCl_3$) δ -114.4. **HRMS M:Z:** $[M]^+$ calculated for $[C_{17}H_{17}FO_2]^+$ 272.1207, found 272.1202. Δ = -2.0 ppm. **Chiral SFC Analysis** (IK-3, CO_2:MeOH 99:01, 2.5 mL min^{-1} , 40 °C) tR = 10.30 (minor), 11.12 (major) minutes. $[\alpha]_D^{25}$ = = -25.4 (c 0.31, $CHCl_3$).

The same procedure was repeated but with *(R)*-sSPhos to yield the product (11.5 mg, 0.042 mmol, 42%, 84% ee).

(R)-5,6-difluoro-2'-methoxy-6'-methyl-[1,1'-biphenyl]-2-ol (**4h**)

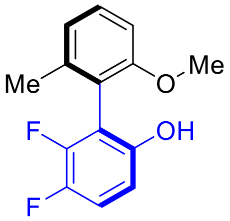

2-bromo-1-methoxy-3-methylbenzene (20.1 mg, 0. 1 mmol), 2- hydroxy-5,6-difluorophenylboronic acid pinacol ester (76.8 mg, 0.3 mmol), Na_3PO_4 (49.2 mg, 0.30 mmol), (*R*)- sRuPhos (3.3 mg, 6 mol%) and $Pd(OAc)_2$ (1.1 mg, 5 mol%) were added to a 4 mL crimp vial. After 3 evacuation-backfill cycles with nitrogen, toluene (0.5 mL)was added. The reaction mixture was stirred at 60 °C for 40 h and then the solvent was removed under a stream of air. The product was purified by column chromatography (5–15% EtOAc: petrol) to yield the title compound, (13.0 mg, 0.052 mmol, 52%, 90% ee). **^{1}H NMR** (700 MHz, $CDCl_3$) δ 7.38 (t, *J* =

8.0 Hz, 1H), 7.11 (dt, *J* = 10.0, 8.9 Hz, 1H), 7.01 (dt, *J* = 7.7, 0.9 Hz, 1H), 6.90 (d, *J* = 8.3 Hz, 1H), 6.75 (ddd, *J* = 9.1, 4.0, 2.0 Hz, 1H), 4.75 (s, 1H), 3.79 (s, 3H), 2.14 (s, 3H). **^{13}C NMR** (176 MHz, $CDCl_3$) δ 157.6, 149.5 – 149.3 (m), 147.7 (dd, *J* = 245.8, 13.7 Hz), 145.2 (dd, *J* = 239.7, 13.7 Hz), 140.2, 130.5, 123.1, 116.9 (d, *J* = 2.1 Hz), 116.3 (d, *J* = 18.7 Hz), 114.1 (d, *J* = 17.0 Hz), 110.2 (dd, *J* = 6.4, 3.5 Hz), 108.8, 55.9, 19.6. **^{19}F NMR** (376 MHz, $CDCl_3$) δ -137.5 (d, *J* = 22.6 Hz), -149.1 (d, *J* = 22.5 Hz). **HRMS**: $[M-H]^-$ calculated for $[C_{14}H_{11}F_2O_2]^-$ 249.0733, found 249.0720. Δ = -5.1 ppm. **Chiral SFC Analysis** (IC-3, CO_2:MeOH 96:04, 2.5 mL min^{-1} , 40 °C) tR = 2.38 (minor), 2.56 (major) minutes. $[\alpha]_D^{25}$ = +20.6 (c 0.95, $CHCl_3$).

The same procedure was repeated but with *(R)*-sSPhos to yield the product (10.8 mg, 0.043 mmol, 43%, 81% ee).

Evaluation of Aryl Bromides with no directing group (Scheme 3A)

6-chloro-2'-(trifluoromethyl)-[1,1'-biphenyl]-2-ol (**5a**)

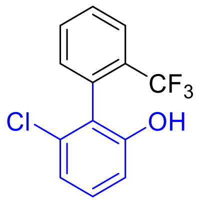

1-bromo-2-(trifluoromethyl)benzene (22.5 mg, 0.1 mmol), 2- hydroxy-6-chlorophenylboronic acid pinacol ester (76.4 mg, 0.3 mmol), Na_3PO_4 (49.2 mg, 0.30 mmol), (*R*)- sRuPhos (3.3 mg, 6 mol%) and $Pd(OAc)_2$ (1.1 mg, 5 mol%) were added to a 4 mL crimp vial. After 3 evacuation-backfill cycles with nitrogen, toluene (0.5 mL) was added. The reaction mixture was stirred at 60 °C for 40 h and then the solvent was removed under a stream of air. The product was purified by column chromatography (5-20% EtOAc:petrol) to yield the product as a white solid (16.4 mg, 0.060 mmol, 60%, 44% ee). **^{1}H NMR** (400 MHz, $CDCl_3$) δ 7.88 (dd, *J* = 7.8, 1.3 Hz, 1H), 7.78 – 7.68 (m, 1H), 7.63 (t, *J* = 7.7 Hz, 1H), 7.37 (d, *J* = 7.5 Hz, 1H), 7.34 – 7.20 (m, 1H), 7.09 (dd, *J* = 8.1, 1.1 Hz, 1H), 6.92 (dd, *J* = 8.2, 1.1 Hz, 1H), 4.53 (s, 1H). **^{19}F NMR** (376 MHz, $CDCl_3$) δ -62.42. **^{13}C NMR** (176 MHz, $CDCl_3$) δ 154.0, 134.4, 132.7, 132.5 (q, *J* = 3.1 Hz), 132.3 (q, *J* = 3.3 Hz), 130.3 (q, *J* = 30.4 Hz), 130.1, 129.3, 126.9 (q, *J* = 5.2 Hz), 124.9, 123.5 (q, *J* = 274.1 Hz), 121.4, 114.0. **HRMS** $[M-H]^-$ calcd for $[C_{13}H_7ClF_3O]^-$ 271.0143, found 271.0130, Δ= -4.7 ppm. **Chiral SFC Analysis** (IG-3, 98:02 CO_2:MeOH, 2.5 mL/min, 5.80 min [major], 6.20 min [minor]).

2'-chloro-6-fluoro-6'-methyl-[1,1'-biphenyl]-2-ol (**5b**)

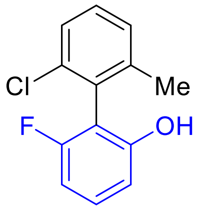

2-bromo-1-chloro-3-methylbenzene (20.5 mg, 0.1 mmol), 2-hydroxy-6-fluorophenylboronic acid pinacol ester (71.4 mg, 0.3 mmol), Na_3PO_4 (49.2 mg, 0.30 mmol), *(R)-* sRuPhos (3.3 mg, 6 mol%) and $Pd(OAc)_2$ (1.1 mg, 5 mol%) were added to a 4 mL crimp vial. After 3 evacuation-backfill cycles with nitrogen, toluene (0.5 mL) was added. The reaction mixture was stirred at 60 °C for 40 h and then the solvent was removed under a stream of air. The product was purified by column chromatography (5-15% EtOAc:petrol) to yield the product as a white solid (4.3 mg, 0.018 mmol, 18%, 8% ee). **^{1}H NMR** (700 MHz, $CDCl_3$) δ 7.42 (ddd, *J* = 8.0, 1.3, 0.7 Hz, 1H), 7.37 – 7.30 (m, 2H), 7.30 – 7.29 (m, 1H), 6.85 (dt, *J* = 8.3, 1.0 Hz, 1H), 6.80 (td, *J* = 8.5, 1.0 Hz, 1H), 4.75 (s, 1H), 2.16 (s, 3H). **^{13}C NMR** (176 MHz, $CDCl_3$) δ 159.9 (d, *J* = 245.3 Hz), 153.7 (d, *J* = 6.1 Hz), 140.9, 135.5, 130.1, 130.1 (d, *J* = 10.6 Hz), 128.8, 128.5, 127.4, 113.4 (d, *J* = 20.8 Hz), 111.3 (d, *J* = 3.2 Hz), 107.7 (d, *J* = 22.3 Hz), 25.4. **HRMS** $[M+Na]^+$ calcd for $C_{13}H_{10}ClFONa$; 259.0296, found 259.0283, Δ= -5 ppm. **Chiral SFC Analysis** (IC-3, 98:02 CO_2:MeOH, 2.5 mL/min, 9.10 min [minor], 11.14 min [major]).

Aryl Bromide containing a competing directing group (Scheme 3B)

(S)-2'-amino-6-fluoro-6'-methoxy-[1,1'-biphenyl]-2-ol (**5c**)

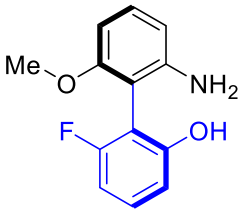

2-bromo-3-methoxyaniline (20.2 mg, 0.1 mmol), 2-hydroxy-6-fluorophenylboronic acid pinacol ester (71.4 mg, 0.3 mmol), Na_3PO_4 (49.2 mg, 0.30 mmol), (*R*)- sRuPhos (3.3 mg, 6 mol%) and $Pd(OAc)_2$ (1.1 mg, 5 mol%) were added to a 4 mL crimp vial. After 3 evacuation-backfill cycles with nitrogen, toluene (0.5 mL) was added. The reaction mixture was stirred at 60 °C for 40 h and then the solvent was removed under a stream of air. The product was purified by column chromatography (5-20% EtOAc:petrol) to yield the product as a light brown solid (10.1 mg, 0.040 mmol, 40%, 71% ee). **^{1}H NMR** (400 MHz, $CDCl_3$) δ 7.42 – 7.10 (m, 2H), 6.88 (dt, *J* = 8.2, 1.0 Hz, 1H), 6.78 (ddd, *J* = 9.2, 8.3, 1.1 Hz, 1H), 6.61 – 6.29 (m, 2H), 5.57 (br s, 1H), 3.77 (s, 3H), 3.70 (br s, 1H). **^{19}F NMR** (376 MHz, $CDCl_3$) δ -111.68. **^{13}C NMR** (176 MHz, $CDCl_3$) δ 160.9 (d, *J* = 246.4 Hz), 158.6, 155.0 (d, *J* = 6.0 Hz), 145.9, 130.8, 129.8 (d, *J* = 10.6 Hz), 112.0 (d, *J* = 3.3 Hz), 109.4 (d, *J* = 20.1 Hz), 108.9, 107.7 (d, *J* = 22.7 Hz), 103.6, 101.5, 55.8. **HRMS** $[M\text{-}H]^-$ calcd for $[C_{13}H_{11}FNO_2]^-$ 232.0779, found 232.0778, Δ= -0.7 ppm. **Chiral SFC Analysis** (IC-3, 97:03 CO_2:MeOH, 2.5 mL/min, 13.27 min [major], 16.16 min [minor]). $[\alpha]_D^{25}$ = +23.7 (c 0.14, $CHCl_3$).

(S)-6-amino-6'-fluoro-[1,1'-biphenyl]-2,2'-diol (**5d**)

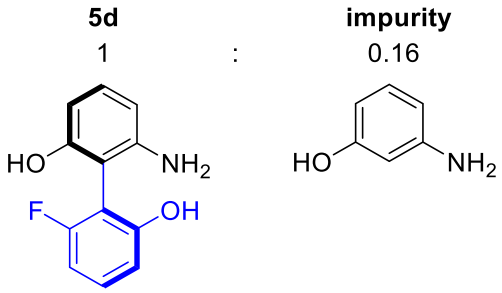

3-amino-2-bromophenol (18.8 mg, 0.1 mmol), 2-hydroxy-6-fluorophenylboronic acid pinacol ester (71.4 mg, 0.3 mmol), Na_3PO_4 (49.2 mg, 0.30 mmol), (*R*)- sRuPhos (3.3 mg, 6 mol%) and $Pd(OAc)_2$ (1.1 mg, 5 mol%) were added to a 4 mL crimp vial. After 3 evacuation-backfill cycles with nitrogen, toluene (0.5 mL) was added. The reaction mixture was stirred at 60 °C for 40 h and then the solvent was removed under a stream of air. The product was purified by column chromatography (5-30% EtOAc:petrol)) to yield a mix of the product as a light brown solid (10.1 mg, 0.040 mmol, 40%, 66% ee) contaminated with an inseparable impurity of debrominated starting material (1:0.16 molar ratio of **5d**:impurity). **^{1}H NMR** (700 MHz, $CDCl_3$) δ 7.36 (td, *J* = 8.3, 6.5 Hz, 1H), 7.17 (t, *J* = 8.1 Hz, 1H), 6.94 (dt, *J* = 8.4, 1.0 Hz, 1H), 6.85 (td, *J* = 8.4, 1.1 Hz, 1H), 6.48 (ddd, *J* = 11.7, 8.1, 1.0 Hz, 2H), 5.77 (br s, 1H), 4.84 (br s, 1H), 3.68 (br s, 2H). **^{19}F NMR** (376 MHz, $CDCl_3$) δ -111.00. **^{13}C NMR** (176 MHz, $CDCl_3$) δ 160.9 (d, *J* = 247.3 Hz), 155.6 (d, *J* = 5.5 Hz), 154.7, 145.7, 131.2, 131.1 (d, *J* = 10.4 Hz), 112.6 (d, *J* = 3.2 Hz), 108.3, 108.2 (d, *J* = 22.3 Hz), 107.2 (d, *J* = 20.1 Hz), 106.3, 102.1.

HRMS $[M+H]^+$ calcd for $[C_{12}H_{11}FNO_2]^+$ 220.0768, found 220.0765, Δ= -1.4 ppm. **Chiral SFC Analysis** (IC-3, 70:30 CO_2:MeOH, 2.5 mL/min, 1.80 min [major], 2.35 min [minor]).

Impact of Substitution on aniline nitrogen (Scheme 3C)

(R)-tert-butyl (2'-fluoro-6'-hydroxy-6-methyl-[1,1'-biphenyl]-2-yl)carbamate (**5e**)

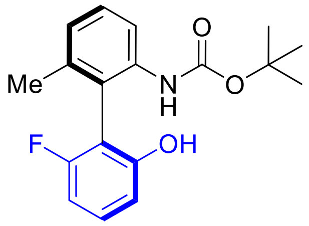

General procedure **F** was performed with tert-butyl (2-bromo-3-methylphenyl)carbamate (28.6 mg, 0.1 mmol) and 2-hydroxy-6-flourophenylboronic acid pinacol ester (71.4 mg, 0.3 mmol), at 60 °C. Only trace amounts (<5%) of the product was observed in the crude NMR.

(R)-*N*-(2'-fluoro-6'-hydroxy-6-methyl-[1,1'-biphenyl]-2-yl)acetamide (**5f**)

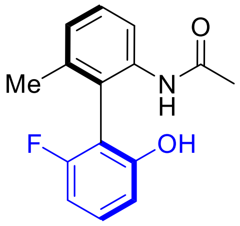

General procedure **F** was performed with *N*-(2-bromo-3-methylphenyl)acetamide (22.8 mg, 0.1 mmol) and 2-hydroxy-6-flourophenylboronic acid pinacol ester (71.4 mg, 0.3 mmol), at 60 °C. The product was purified by column chromatography (5–25% EtOAc: Pet) to yield the title compound (13.5 mg, 0.052 mmol, 52%, 83% *ee*). **^{1}H NMR** (400 MHz, $CDCl_3$) δ 7.89 (d, J = 8.2 Hz, 1H), 7.39 (t, J = 8.2 Hz, 1H), 7.33 (m, 1 H), 7.21 (d, J = 7.7 Hz, 1H), 6.93 – 6.85 (d and br s, 2H), 6.80 (t, J = 8.4 Hz, 1H), 5.59 (br s, 1H), 2.10 (s, 3H), 1.98 (s, 3H). **^{13}C NMR** (126 MHz, $CDCl_3$) δ 169.3, 159.9 (d, J = 245.0 Hz), 154.8 (d, J = 5.7 Hz), 139.2, 136.5, 130. 5 (d, J = 10.5 Hz), 129.8, 127.4, 122.0, 121.2, 112.4, 111.6 (d, J = 19.8 Hz), 107.8 (d, J = 21.6 Hz), 24.2, 20.0. **^{19}F NMR** (376 MHz, $CDCl_3$) δ -113.03. **Chiral SFC Analysis** (IE-3, 95:05, CO_2 : MeOH, 1.25 mL min^{-1} , 40 °C) tR = 10.97 (major), 12.11 (minor). $[\alpha]_D^{25}$ = - 11.3 (c 0.45, $CHCl_3$).

Data in accordance with literature.[6]

(R)-2,2,2-trifluoro-N-(2'-fluoro-6'-hydroxy-6-methyl-[1,1'-biphenyl]-2-yl)acetamide (**5g**)

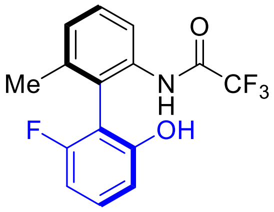

General procedure **F** was performed with N-(2-bromo-3-methylphenyl)-2,2,2-trifluoroacetamide (28.2 mg, 0.1 mmol) and 2- hydroxy-6-fluorophenylboronic acid pinacol ester (71.4 mg, 0.3 mmol) at 60 °C. The product was purified by column chromatography (5-15% EtOAc: petrol) to yield the title compound (15.1

mg, 0.048 mmol, 48%, 13% ee). **^{1}H NMR** (700 MHz, $CDCl_3$) δ 7.97 (d, *J* = 8.2 Hz, 1H), 7.66 (s, 1H), 7.43 (t, *J* = 7.9 Hz, 1H), 7.35 (td, *J* = 8.3, 6.5 Hz, 1H), 7.28 (d, *J* = 7.7 Hz, 1H), 6.87 (dt, *J* = 8.3, 1.0 Hz, 1H), 6.83 (td, *J* = 8.6, 1.0 Hz, 1H), 5.21 (s, 1H), 2.14 (s, 3H). **^{19}F NMR** (376 MHz, $CDCl_3$) δ -77.2, -113.5. **^{13}C NMR** (176 MHz, $CDCl_3$) δ 159.9 (d, *J* = 245.8 Hz), 155.1 (q, *J* = 37.4, 36.8 Hz), 154.2 (d, *J* = 6.4 Hz), 139.8, 133.9, 131.4 (d, *J* = 10.5 Hz), 130.0, 128.7, 121.9, 120.3, 115.6 (q, *J* = 288.3 Hz), 112.2 (d, *J* = 4.1 Hz), 109.9 (d, *J* = 20.5 Hz), 108.2 (d, *J* = 22.4 Hz), 19.9. **Chiral SFC Analysis** (IC-3, 98:02 CO_2:MeOH, 2.5 mL/min, 4.22 min [minor], 4.68 min [major]). **HRMS** $[M-H]^-$ calcd for $[C_{15}H_{10}F_4NO_2]^-$; 312.0653, found 312.0642, Δ= -3.5 ppm.

(R)-N-(2'-fluoro-6'-hydroxy-6-methyl-[1,1'-biphenyl]-2-yl)-4-methylbenzenesulfonamide (**5h**)

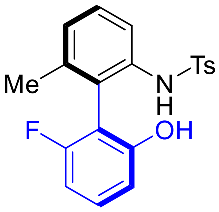

General procedure **F** was performed with N-(2-bromo-3-methylphenyl)-4-methylbenzenesulfonamide (34.0 mg, 0.1 mmol) and 2- hydroxy-6-fluorophenylboronic acid pinacol ester (71.4 mg, 0.3 mmol) at 100 °C. The product was purified by column chromatography (15-25% EtOAc: petrol) to yield the title compound (14.6 mg, 0.039 mmol, 39%, 80% ee). **^{1}H NMR** (700 MHz, $CDCl_3$) δ 7.55 (d, *J* = 8.3 Hz, 2H), 7.51 (d, *J* = 8.3 Hz, 1H), 7.32 – 7.27 (m, 2H), 7.20 (d, *J* = 8.0 Hz, 2H), 7.08 (d, *J* = 7.7 Hz, 1H), 6.80 (d, *J* = 8.3 Hz, 1H), 6.71 (td, *J* = 8.4, 1.0 Hz, 1H), 6.31 (br s, 1H), 2.39 (s, 3H), 1.98 (s, 3H). **^{19}F NMR** (376 MHz, $CDCl_3$) δ -112.9. **^{13}C NMR** (176 MHz, $CDCl_3$) δ 159.9 (d, *J* = 246.4 Hz), 154.2 (d, *J* = 5.7 Hz), 144.0, 139.7, 135.9 (d, *J* = 33.8 Hz), 131.0 (d, *J* = 10.0 Hz), 130.0, 129.7, 127.3, 127.0, 120.6, 118.6, 112.0 (d, *J* = 3.2 Hz), 110.3 (d, *J* = 20.7 Hz), 108.1 (d, *J* = 22.1 Hz), 21.6, 20.0. **Chiral SFC Analysis** (IC-3, 90:10 CO_2:MeOH, 2.5 mL/min, 9.79 min [minor], 11.52 min [major]). **HRMS** $[M+H]^+$ calcd for $[C_{20}H_{19}FNO_3S]^+$; 372.1065, found 372.1066, Δ= 0.6 ppm. $[\alpha]_D^{25}$ = + 41.5 (c 0.43, MeOH).

Effect of swapping coupling partners to access common biaryl products (Scheme 3D)

(R)-6-fluoro-6'-methyl-[1,1'-biphenyl]-2,2'-diol (**4i**)

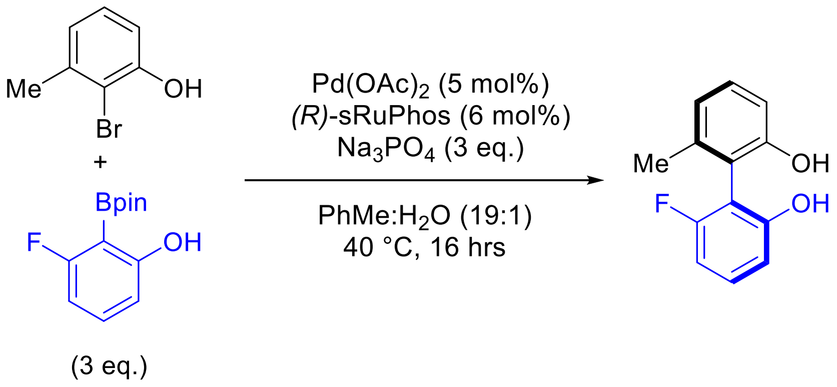

3-methyl-2-bromophenol (18.7 mg, 0.1 mmol), 2-hydroxy 6-fluorophenylboronic acid pinacol ester (71 mg, 0.3 mmol), $Pd(OAc)_2$ (1.1 mg, 0.005 mmol), (R)-sRuPhos (3.3 mg, 6 mol%) and Na_3PO_4 (49 mg, 0.3 mmol) were added to a 4 mL crimp vial. After 3 evacuation-backfill cycles with nitrogen, toluene (0.475 mL) and water (0.025 mL) were added. The reaction mixture was stirred at 40 °C for 16 h and then the solvent was removed under a stream of air. The product was purified by column chromatography (10–20% EtOAc: petrol) to yield the title compound, (13.1 mg, 0.060 mmol, 60%, 93% ee). **^{1}H NMR** (700 MHz, $CDCl_3$) δ 7.32 (td, *J* = 8.3, 6.5 Hz, 1H), 7.26 (t, *J* = 7.9 Hz, 1H)*, 6.94 (dt, *J* = 7.6, 0.9 Hz, 1H), 6.90 – 6.86 (m, 2H), 6.80 (td, *J* = 8.5, 1.0 Hz, 1H), 5.02 (s, 1H), 4.79 (s, 1H), 2.09 (s, 3H). **^{13}C NMR** (176 MHz, $CDCl_3$) δ 160.6 (d, *J* = 246.5 Hz), 154.8 (d, *J* = 5.8 Hz), 154.0, 140.0, 130.9 (d, *J* = 10.3 Hz), 130.6, 122.8, 115.0, 113.4, 111.6 (d, *J* = 3.3 Hz), 109.3 (d, *J* = 20.9 Hz), 108.0 (d, *J* = 22.2 Hz), 19.8. **^{19}F NMR** (376 MHz, $CDCl_3$) δ -112.9. **Chiral HPLC Analysis** (SC-3, Hexane: IPA 97:03, 1.25 mL min^{-1} , 40 °C) tR = 14.72 (major), 18.63 (minor) minutes.

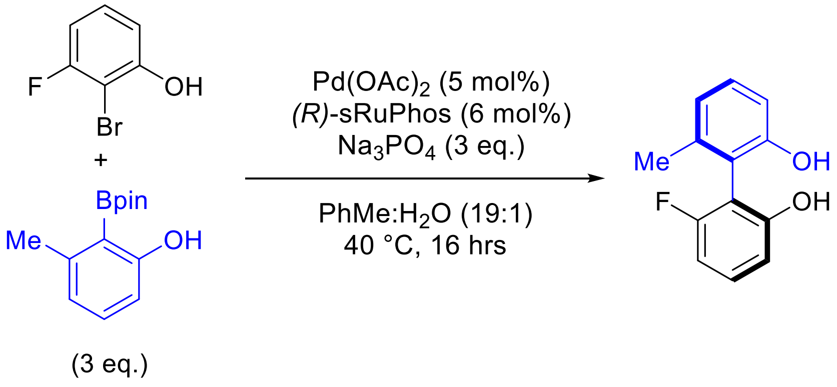

The same procedure was repeated but with 2-bromo-3-fluorophenol (19 mg, 0.1 mmol) and 2-hydroxy 6-methylphenylboronic acid pinacol ester (70 mg, 0.3 mmol), to yield the product (1.1 mg, 0.005 mmol, 5%, 92% ee).

Data in accordance with literature.[6]

(R)-2'-fluoro-6'-methoxy-6-methyl-[1,1'-biphenyl]-2-ol (**4j**)

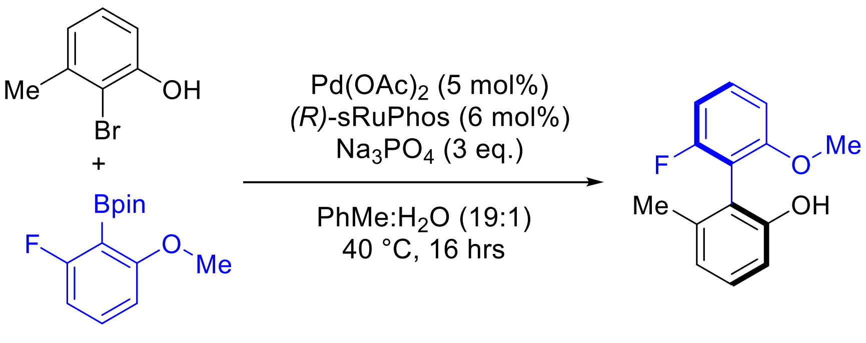

(3 eq.)

3-methyl-2-bromophenol (18.7 mg, 0.1 mmol), 2-methoxy 6-fluorophenylboronic acid pinacol ester (76 mg, 0.3 mmol), $Pd(OAc)_2$ (1.1 mg, 0.005 mmol), (R)-sRuPhos (3.3 mg, 6 mol%) and Na_3PO_4 (49 mg, 0.3 mmol) were added to a 4 mL crimp vial. After 3 evacuation-backfill cycles with nitrogen, toluene (0.475 mL) and water (0.025 mL) were added. The reaction mixture was stirred at 40 °C for 16 h and then the solvent was removed under a stream of air. The product was purified by column chromatography (0-15% EtOAc:petrol) to yield the product as a colourless oil (18.5 mg, 0.080 mmol, 80%, 77% ee). **^{1}H NMR** (400 MHz, CDCl3) δ 7.39 (td, J = 8.4, 6.6 Hz, 1H), 7.21 (t, J = 7.8 Hz, 1H), 6.88 (d, J = 8.3 Hz, 1H), 6.87 – 6.80 (m, 3H), 4.68 (s, 1H), 3.79 (s, 3H), 2.06 (s, 3H). **^{13}C NMR** (101 MHz, CDCl3) δ 160.91 (d, J = 245.6 Hz), 158.4 (d, J = 7.1 Hz), 153.3, 138.8, 130.4 (d, J = 10.5 Hz), 129.2, 122.0, 118.0, 112.8, S41 111.7 (d, J = 20.2 Hz), 108.7 (d, J = 23.0 Hz), 106.9 (d, J = 3.0 Hz), 56.2, 19.9. **^{19}F NMR** (376 MHz, CDCl3) δ -111.22. **Chiral HPLC Analysis** (SC-3, Hexane: IPA, 97:03, 1.25 mL min^{-1} , 40 °C) tR = 12.01 (minor), 13.13 (major) minutes.

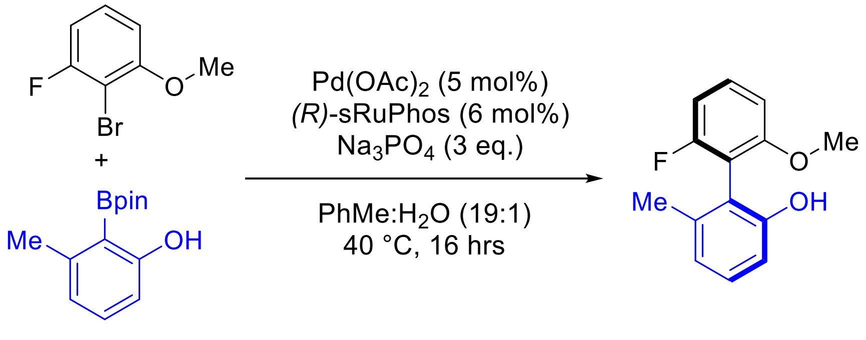

(3 eq.)

The same procedure was repeated but with 2-bromo-1-fluoro-3-methoxybenzene (20.1 mg, 0.1 mmol) and 2-hydroxy 6-methylphenylboronic acid pinacol ester (70 mg, 0.3 mmol), to yield the product (17.1 mg, 0.074 mmol, 77%, 74% ee).

Data in accordance with literature.[6]

(R)-2'-amino-6'-fluoro-6-methyl-[1,1'-biphenyl]-2-ol (**3p**)

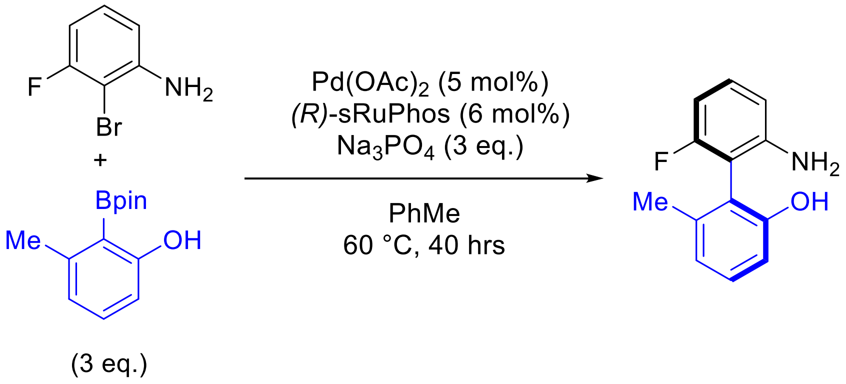

General procedure **F** was performed with 2-bromo-3-fluoroaniline (19.0 mg, 0.1 mmol) and 2- hydroxy-6-methylphenylboronic acid pinacol ester (70.2 mg, 0.3 mmol), at 60 °C. The product was purified by column chromatography (5-20% EtOAc:petrol) to yield the product as a white solid (10.9 mg, 0.050 mmol, 50%, 91% ee), as previously mentioned.

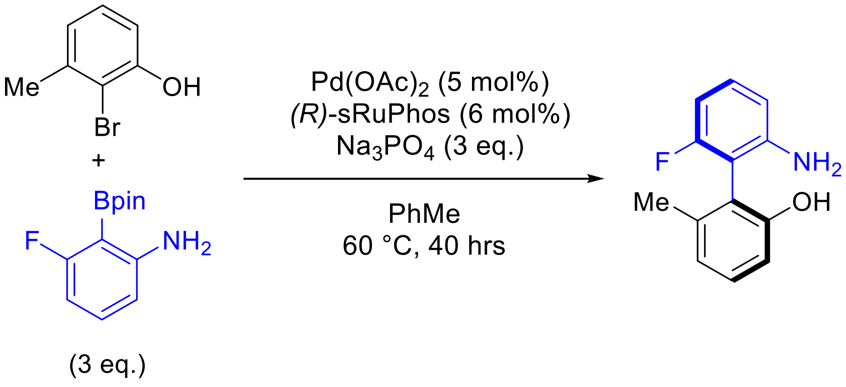

The same procedure was repeated but with 2-bromo-3-methylphenol (18.7 mg, 0.1 mmol) and 2-amino-6-fluorophenylboronic acid pinacol ester (71 mg, 0.3 mmol), to yield the product (19.6 mg, 0.090 mmol, 90%, 85% ee).

Direct Comparison of Ligands –(Scheme 3G)

General procedure M: asymmetric Suzuki cross-coupling to form **3a**

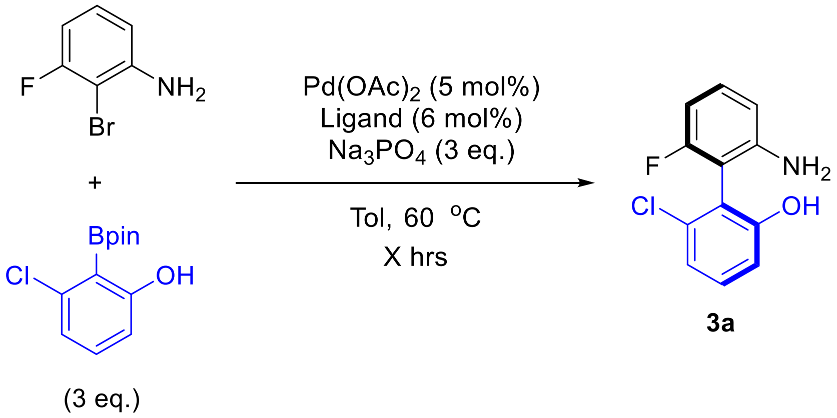

2-bromo-3-fluoroaniline (19.0 mg, 0.1 mmol) and 2- hydroxy-6-chlorophenylboronic acid pinacol ester (76.4 mg, 0.3 mmol), Na_3PO_4 (49.2 mg, 0.30 mmol), Ligand (6 mol%) and $Pd(OAc)_2$ (1.12 mg, 5 mol%) were added to a 4 mL crimp vial. After 3 evacuation-backfill cycles with nitrogen, toluene (0.5 mL) was added. The reaction mixture was stirred at 60 °C for 16 h, with aliquots being taken at 3, 6, 9 and 16 hours.

time	RuPhos	sRuPhos	Me-sRuPhos	sRuPhos-Np	sSPhos
0 h	0 %	0 %	0 %	0 %	0 %
3 h	31 %	25 %	15 %	0 %	4 %
6 h	45 %	39 %	28 %	1 %	9 %
9 h	64 %	48 %	30 %	1 %	17 %
16 h	75 %	70 %	39 %	2 %	22 %

Effect of Cation on Enantioselectivity

2-bromo-3-fluoroaniline (19.0 mg, 0.1 mmol), 2-hydroxy-6-chlorophenylboronic acid pinacol ester (76.4 mg, 0.3 mmol), base (0.30 mmol), *(R)*- sRuPhos (3.3 mg, 6 mol%) and $Pd(OAc)_2$ (1.1 mg, 5 mol%) were added to a 4 mL crimp vial. After 3 evacuation-backfill cycles with nitrogen, toluene (0.5 mL) was added. The reaction mixture was stirred at 60 °C for 40 h and then the solvent was removed under a stream of air.

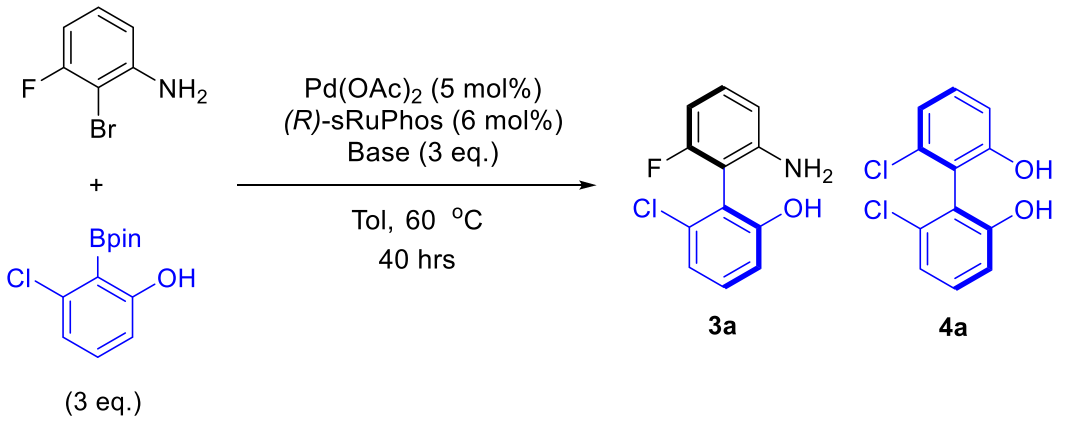

Base	Yield	Ee
Na_3PO_4	(70)	(99)
Na_2HPO_4	50%	99
NaH_2PO_4	<5%	-
K_3PO_4	31%	99
K_2HPO_4	22%	99
KH_2PO_4	<5%	-
$NBu_4H_2PO_4$	<5%	-
Na_3PO_4 + 15-crown-5	<5%	-
Na_3PO_4 + 12-crown-4	<5%	-
NaOH	50%	99
KOH	35%	98
NBu_4OH	<5%	-

Racemization Experiment: (S)-2'-amino-6'-chloro-6-fluoro-[1,1'-biphenyl]-2-ol (3f)

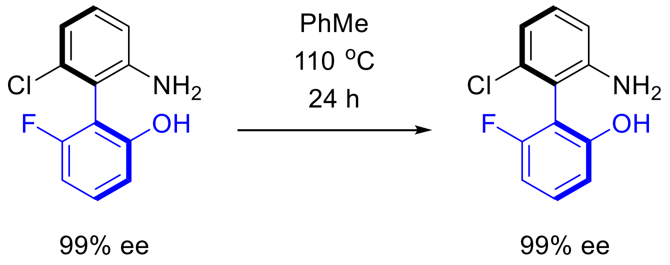

(S)-2'-amino-6'-chloro-6-fluoro-[1,1'-biphenyl]-2-ol (**3f**) (2.0 mg, 0.01 mmol) was added to a 4 mL crimp vial. After 3 evacuation-backfill cycles with nitrogen, toluene (0.25 mL) was added. The reaction mixture was stirred at 110 °C for 24 h and then the solvent was removed under a stream of air. The %ee of product remained the same (99% ee). **Chiral SFC Analysis** (IC-3, 98:02 CO_2:MeOH, 2.5 mL/min, 9.10 min [minor], 11.14 min [major]).

Racemization Experiments: (S)-2'-amino-3',5'-dichloro-6-methyl-[1,1'-biphenyl]-2-ol (**3r**)

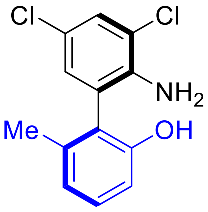

3r

32%, 80% *ee*

69% ee after 10 months storage at RT

Chiral SFC analysis of **3r** revealed that the *ee* had deteriorated from 80% *ee* to 69% *ee* over ten months at room temperature. The rate of racemisation was then monitored by the following procedure developed by Clayden, Smith, and Armstrong et al.[15] In an oven dried 4 ml crimp top vial 8.4 mg of **3r** was dissolved in 1.5 ml of toluene. The resulting mixture was then heated to 60°C in a preheated heating block. Aliquots were taken and the *ee* determined *via* chiral SFC analysis (Table 1, Figure 1). **Chiral SFC Analysis** (IA-3, 96:04, CO_2:MeOH, 2.5 mL min^{-1}, 40 °C) tR = 12.01 (minor), 12.60 (major) minutes.

Time (min)	Time (seconds)	ee (%)	ln(1/ee)
0	0	69	0.371064
60	3600	66.6	0.406466
180	10800	64.8	0.433865
300	18000	61.4	0.48776
420	25200	58.4	0.537854
540	32400	57.2	0.558616
1440	86400	33.8	1.084709
1740	104400	31.8	1.145704
1920	115200	31.2	1.164752

Table 1: ee of **3r** for racemisation of a toluene solution of **3r** at 60 °C. ln(1/ee) is calculated as ee being a maximum of 1 at optical purity. I.e., 69% ee would give ln(1/0.69).

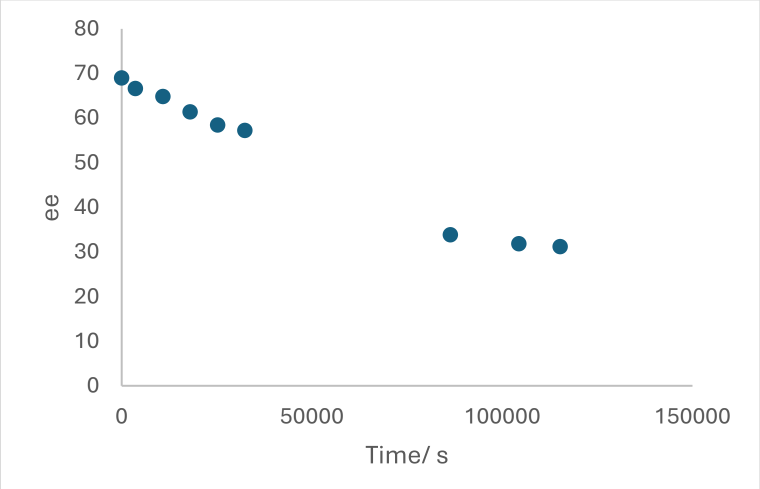

Figure 1: A plot of time (in seconds) versus ee.

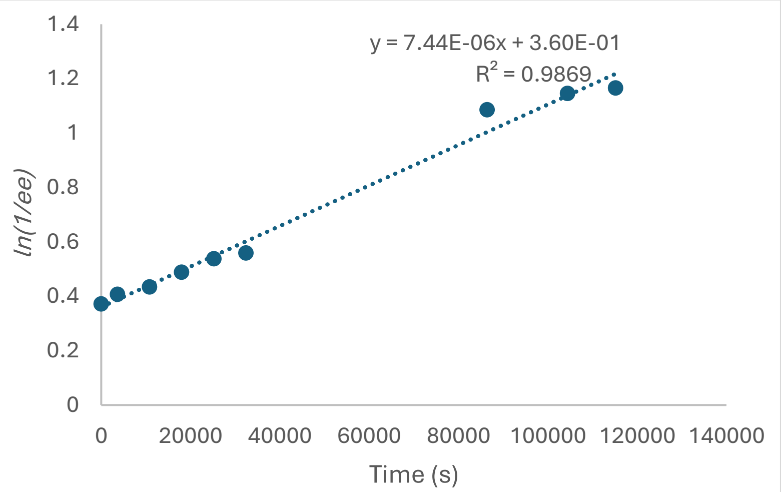

Figure 2: A plot of time (in seconds) versus ln(1/ee) gives k_{rac} = 7.44 ×10^{-6} s^{-1}.

The gradient of this line graph (Figure 2) corresponds to k_{rac}.

k_{rac} = 7.44 × 10^{-6} s^{-1} (at 60°C)

Substituting this value into eq. ii gives the corresponding racemization half-life

$$t_{rac}^{1/2} = \frac{ln2}{k_{rac}} = \frac{0.693}{7.44 \times 10^{-6}\, s^{-1}} = 9.31 \times 10^{4} s = 25.9\text{h (at 60°C)}$$

The rate constant for enantiomerisation was subsequently calculated

$$k_{ent} = \frac{k_{rac}}{2} = \frac{7.44 \times 10^{-6}\, s^{-1}}{2} = 3.72 \times 10^{-6}\, \text{s}^{-1}$$

Substituting this value of k_{ent} along with the temperature (333.15 K) into the Eyring equation (eq. iii) gives $\Delta G^{\ddagger}$. For compound **3r**.

$$\Delta G^{\ddagger} = RTln(\frac{k_B T}{k_{ent} h}) = 8.314 \times 333.15 \times ln(\frac{1.381 \times 10^{-23} \times 333.15}{3.72 \times 10^{-6} \times 6.626 \times 10^{-34}}) = 116.5\, kJmol^{-1}$$
$$= 27.8\ \text{kcalmol}^{-1}$$

X-Ray Structure of *(S)*- 2'-amino-6'-chloro-6-fluoro-[1,1'-biphenyl]-2-ol (**3f**)

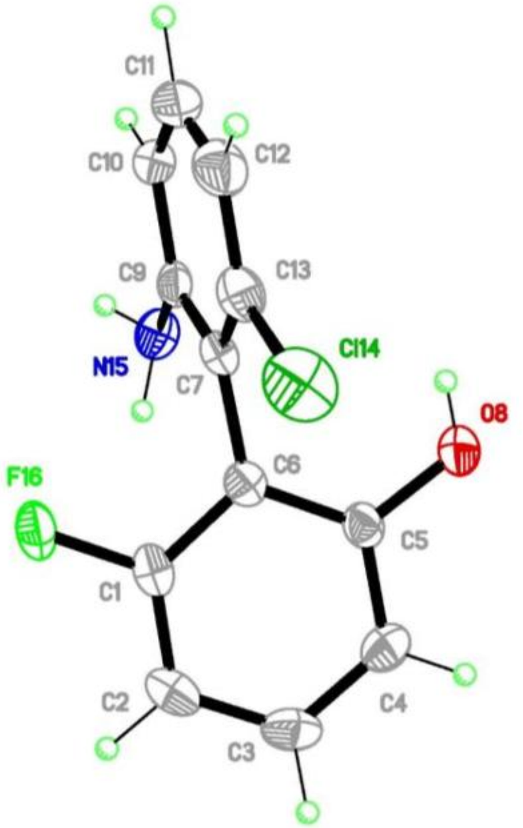

Crystals of the purified sample were grown via slow diffusion of hexane into an ethyl acetate solution of **3f**. These were analysed by Dr Andrew Bond (University of Cambridge) by x-ray diffraction from which the absolute configuration was determined to be (*S*). The structure was deposited in the Cambridge Crystallographic Data Centre (deposition no. 2418029). The absolute stereochemistry of the other atropisomeric products in the scope have been assigned in analogy to this compound.

Unsuccessful Substrates

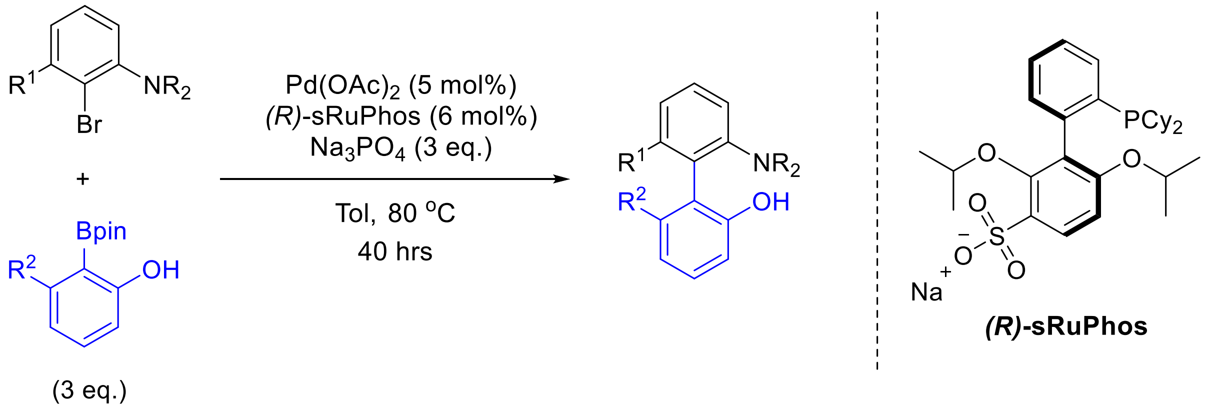

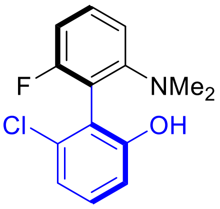

0%

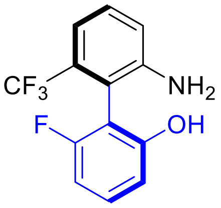

0%

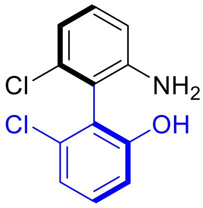

0%

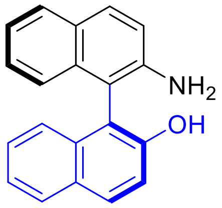

0%

References

1. (a) Bakker, B. H.; Cerfontain, H., Sulfonation of Alkenes by Chlorosulfuric Acid, Acetyl Sulfate, and Trifluoroacetyl Sulfate. *Eur. J. Org. Chem.* **1999,** *1999*, 91-96; (b) Corby, B. W.; Gray, A. D.; Meaney, P. J.; Falvey, M. J.; Lawrence, G. P.; Smyth, T. P., Clean-Chemistry Sulfonation of Aromatics †. *J. Chem. Res.* **2002,** *2002*, 326 - 327; (c) Anderson, L. J.; Yuan, X.; Fahs, G. B.; Moore, R. B., Blocky Ionomers via Sulfonation of Poly(ether ether ketone) in the Semicrystalline Gel State. *Macromolecules* **2018,** *51*, 6226-6237; (d) Wang, J.-C.; Kim, S.; Lee, J. H.; Ascencio, C.; Chung, L.; Cashman, J.; Jung, K. W., Mechanistic Investigations on the Direct Regioselective Sulfonation of Gaseous Light Hydrocarbons by Sulfur Dioxide. *ChemCatChem*, e202401970.
2. Lou, Y.; Wei, J.; Li, M.; Zhu, Y., Distal Ionic Substrate–Catalyst Interactions Enable Long-Range Stereocontrol: Access to Remote Quaternary Stereocenters through a Desymmetrizing Suzuki–Miyaura Reaction. *J. Am. Chem. Soc.* **2022,** *144*, 123-129.
3. Denman, B. N.; Plasek, E. E.; Roberts, C. C., Ligand-Induced Regioselectivity in Metal-Catalyzed Aryne Reactions Using Borylaryl Triflates as Aryne Precursors. *Organometallics* **2023,** *42*, 859-864.
4. Probst, N.; Grelier, G.; Ghermani, N.; Gandon, V.; Alami, M.; Messaoudi, S., Intramolecular Pd-Catalyzed Anomeric C(sp3)–H Activation of Glycosyl Carboxamides. *Org. Lett.* **2017,** *19*, 5038-5041.
5. Bedford, R. B.; Cazin, C. S. J., A novel catalytic one-pot synthesis of carbazoles via consecutive amination and C–H activation. *Chem. Commun.* **2002**, 2310-2311.
6. Pearce-Higgins, R.; Hogenhout, L. N.; Docherty, P. J.; Whalley, D. M.; Chuentragool, P.; Lee, N.; Lam, N. Y. S.; McGuire, T. M.; Valette, D.; Phipps, R. J., An Enantioselective Suzuki–Miyaura Coupling To Form Axially Chiral Biphenols. *J. Am. Chem. Soc.* **2022,** *144*, 15026-15032.
7. Yang, X.-Y.; Zhao, H.-Y.; Mao, S.; Zhang, S.-Q., Copper-Mediated monochlorination of anilines and nitrogen-containing heterocycles. *Synth. Commun.* **2018,** *48*, 2708-2714.
8. Zhang, J.; POWERS, H.; ALBERS, A.; Pham, P.; Wu, G.; BUELL, J.; Spevak, W.; GUO, Z.; Walleshauser, J.; Zhang, Y. COMPOUNDS AND METHODS FOR IDO AND TDO MODULATION, AND INDICATIONS THEREFOR. PCT/US2019/023036, 2018.
9. Chen, D.; Chen, Y.; Ma, Z.; Zou, L.; Li, J.; Liu, Y., One-Pot Synthesis of Indole-3-acetic Acid Derivatives through the Cascade Tsuji–Trost Reaction and Heck Coupling. *J. Org. Chem.* **2018,** *83*, 6805-6814.
10. NARASIMHULU;, N. B.; MANOJ, P.; STANLEY, D. A.; BARBARA, Z. Z.; P, C. T.; R;, L. D.; KEVIN, P.; ZHONGYU, W.; A;, W. M.; F, K. J. INHIBITORS OF HUMAN IMMUNODEFICIENCY VIRUS REPLICATION. WO2014028384A1, 2014.
11. Lee, B.; Mihai, M. T.; Stojalnikova, V.; Phipps, R. J., Ion-Pair-Directed Borylation of Aromatic Phosphonium Salts. *J. Org. Chem.* **2019,** *84*, 13124-13134.
12. Guo, H.; Wang, H.; Tan, J.-P.; Luo, W.-K.; Liu, C.; Zhou, J.; Luo, W., Divergent Synthesis of Atropisomeric Diarylamines Enabled by Bromine-Masked Indolines. *European Journal of Organic Chemistry* **2025,** *28*, e202500159.
13. K. Ohba, Y. N., T. Matsudaira, M. Hamada, R. Yamazaki, T. Ibuki SULFONAMIDE OR SULFINAMIDE COMPOUND HAVING EFFECT OF INDUCING BRD4 PROTEIN DEGRADATION AND PHARMACEUTICAL USE THEREOF. 2021.
14. Meyers, A. I.; Nelson, T. D.; Moorlag, H.; Rawson, D. J.; Meier, A., Chiral oxazoline route to enantiomerically pure biphenyls: magnesio and copper mediated asymmetric hetero- and homo-coupling reactions. *Tetrahedron* **2004,** *60*, 4459-4473.

15. Heeb, J.-P.; Clayden, J.; Smith, M. D.; Armstrong, R. J., Interrogating the configurational stability of atropisomers. *Nature Protocols* **2023,** *18*, 2745-2771.

Neopentyl (S)-2'-(dicyclohexylphosphaneyl)-2,6-diisopropoxy-[1,1'-biphenyl]-3-sulfonate (**(S)-sRuPhos-Np)**

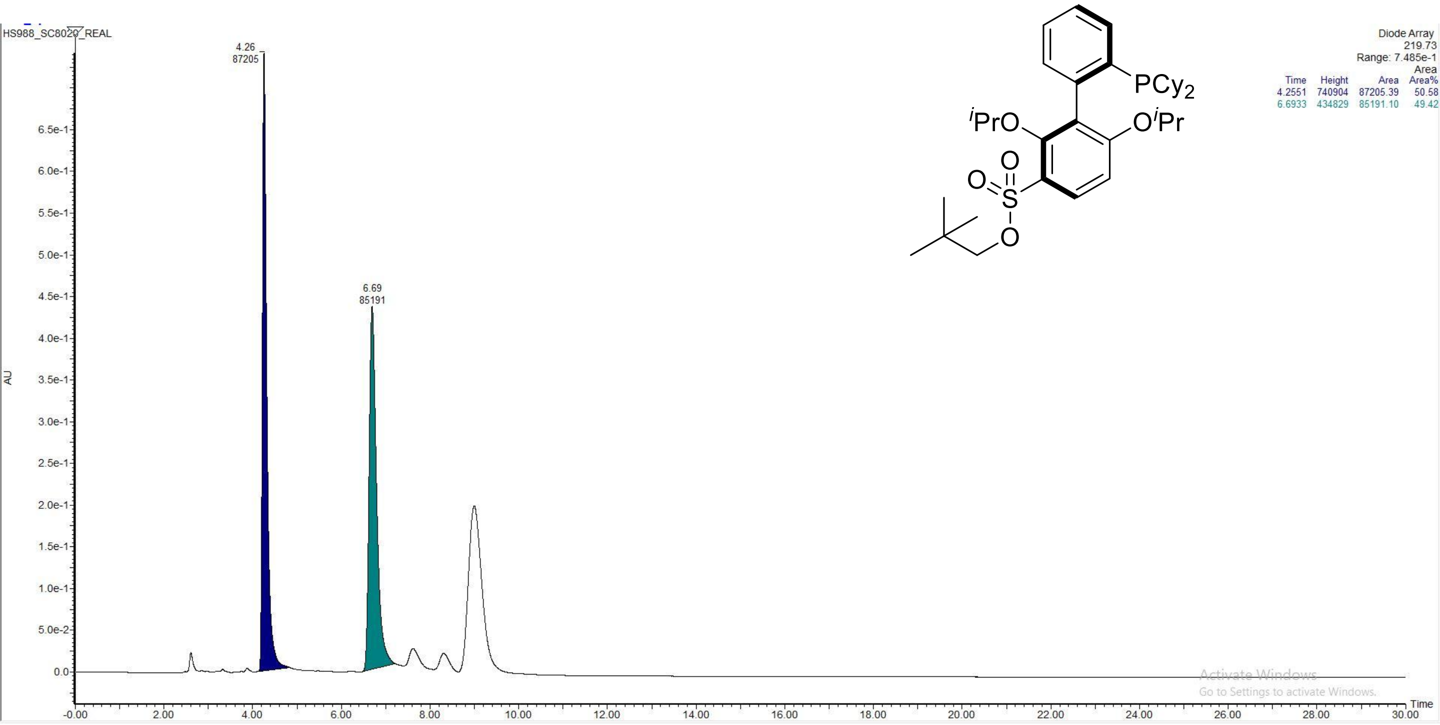

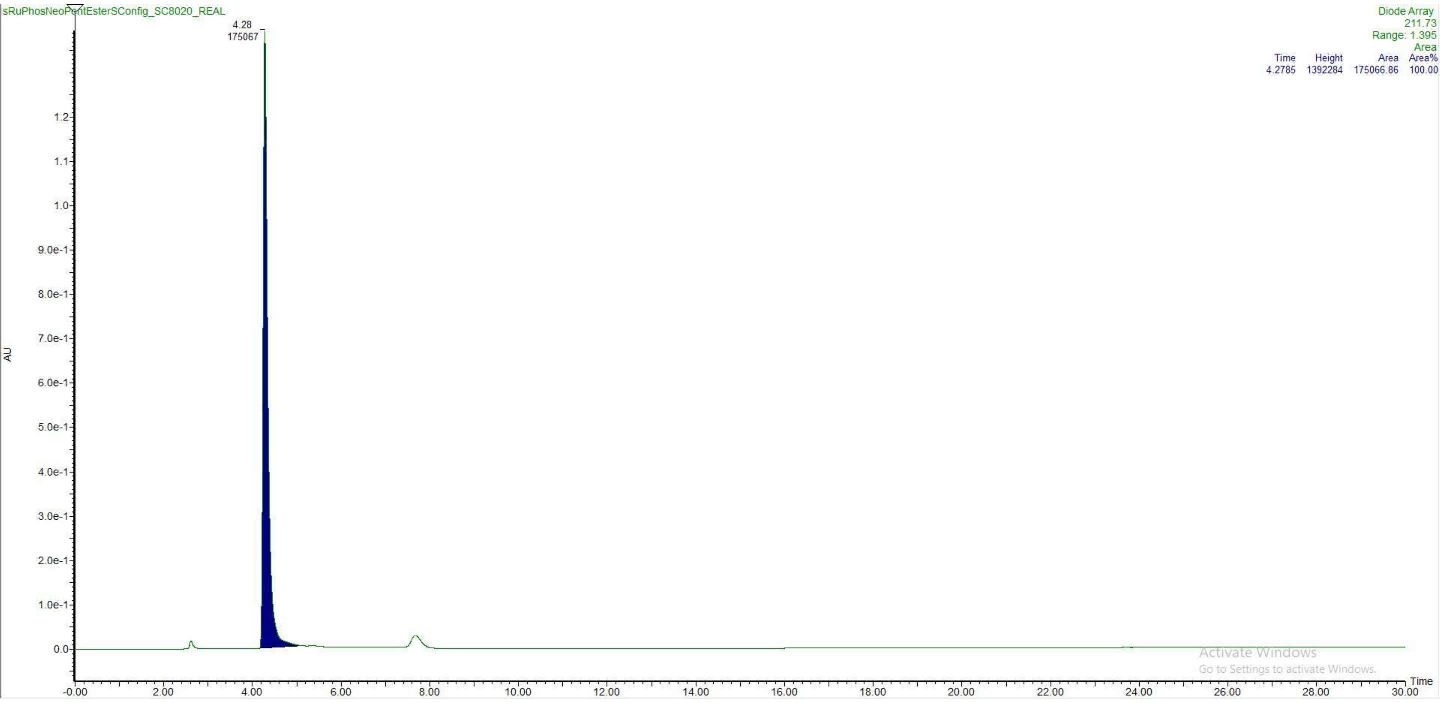

(*S*)-2'-amino-6'-chloro-6-fluoro-[1,1'-biphenyl]-2-ol (**3a**)

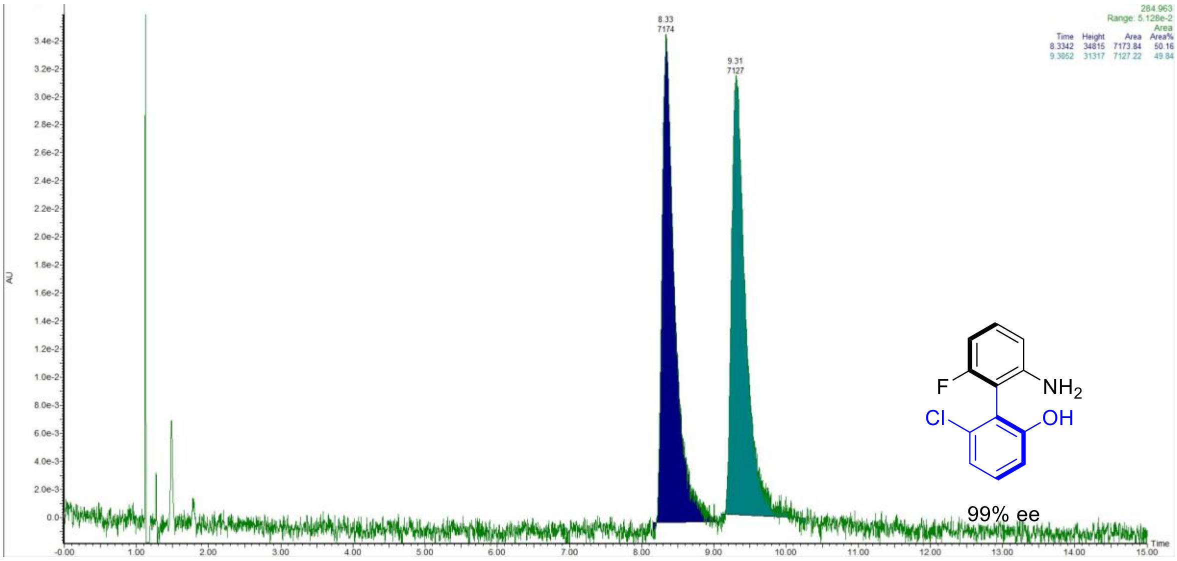

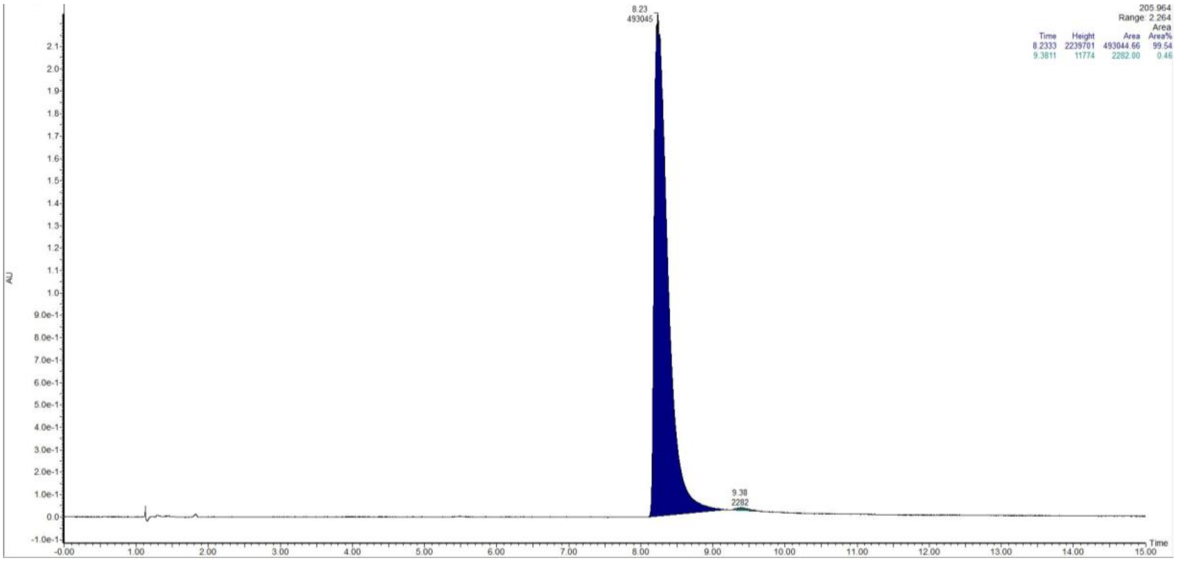

(S)-2'-amino-6-chloro-4',6'-difluoro-[1,1'-biphenyl]-2-ol (**3b**)

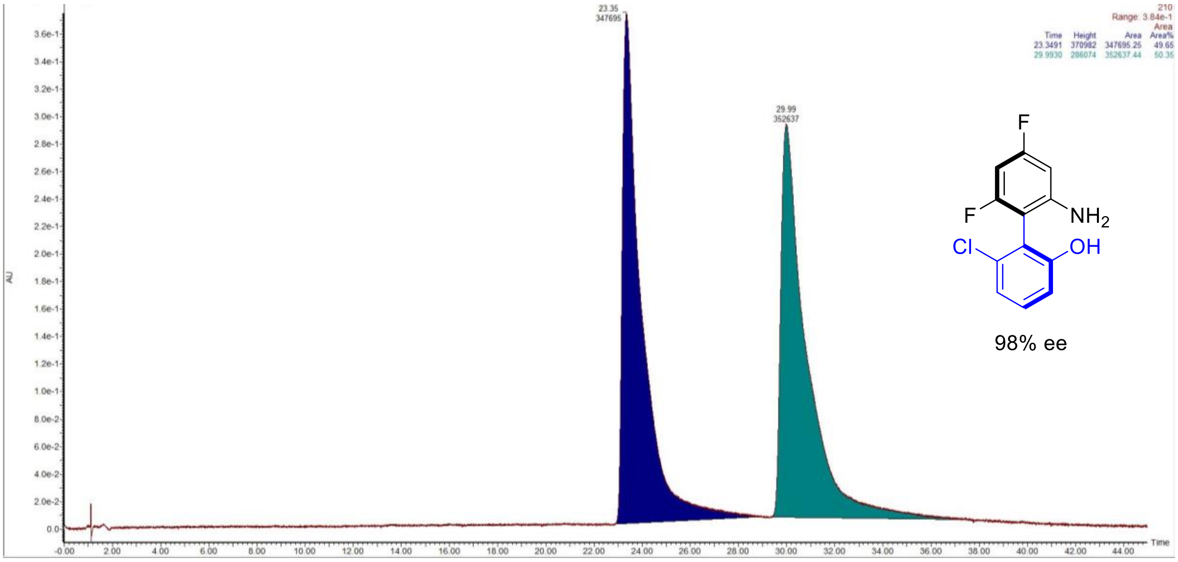

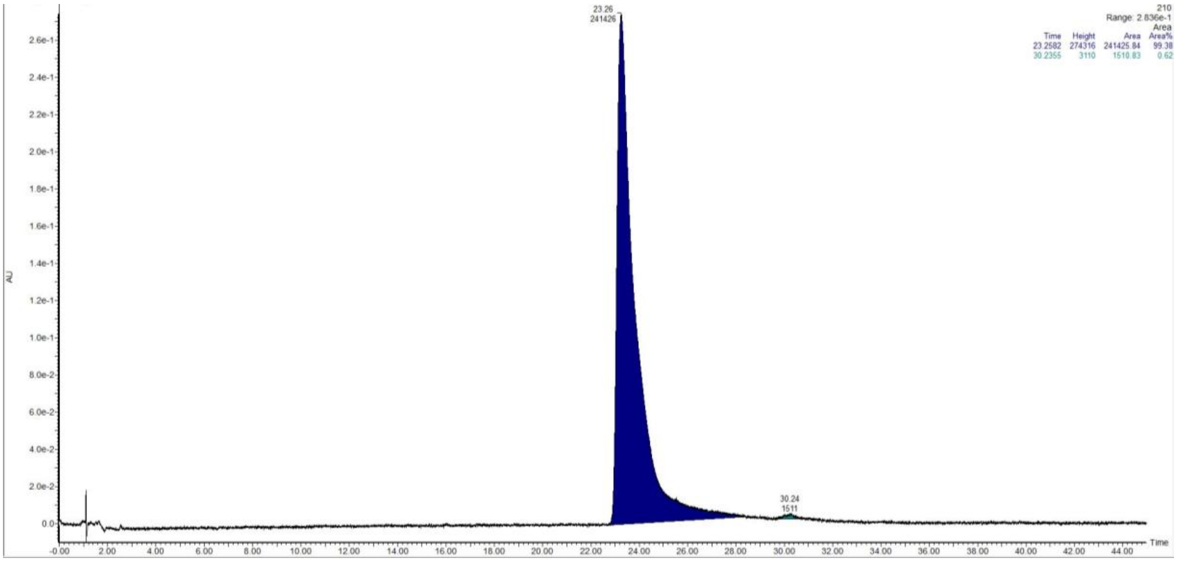

(S)-6'-amino-6-chloro-2',3'-difluoro-[1,1'-biphenyl]-2-ol (**3c**)

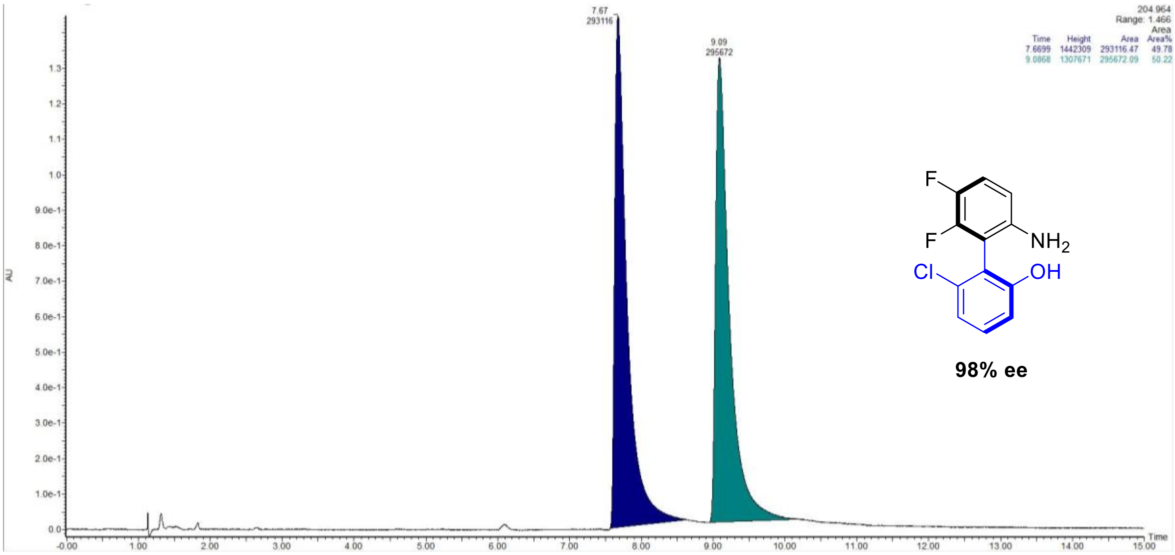

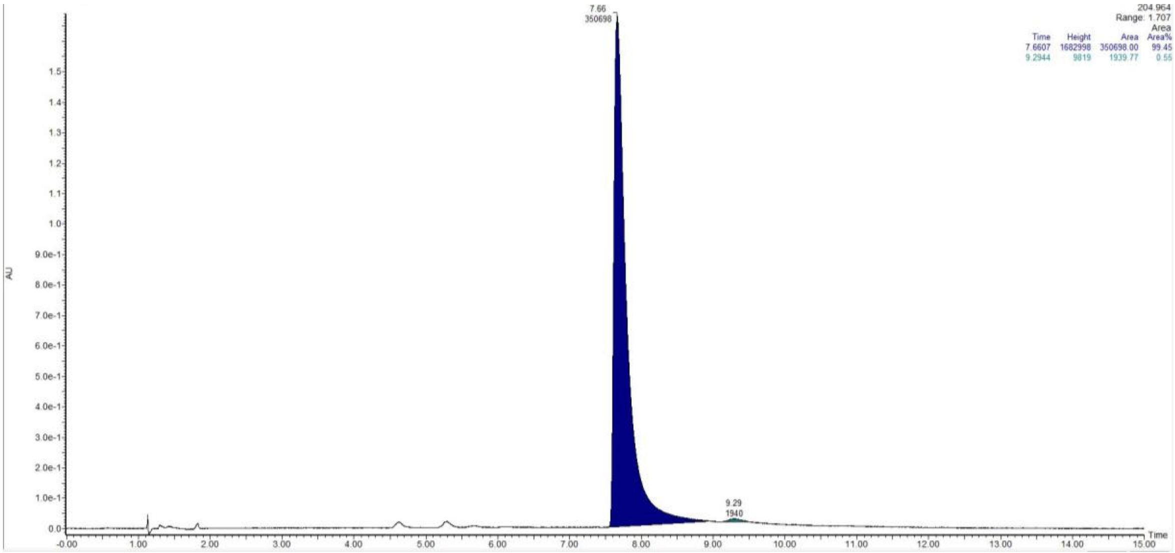

(S)- 6'-amino-3',6-dichloro-2'-fluoro-[1,1'-biphenyl]-2-ol (**3d**)

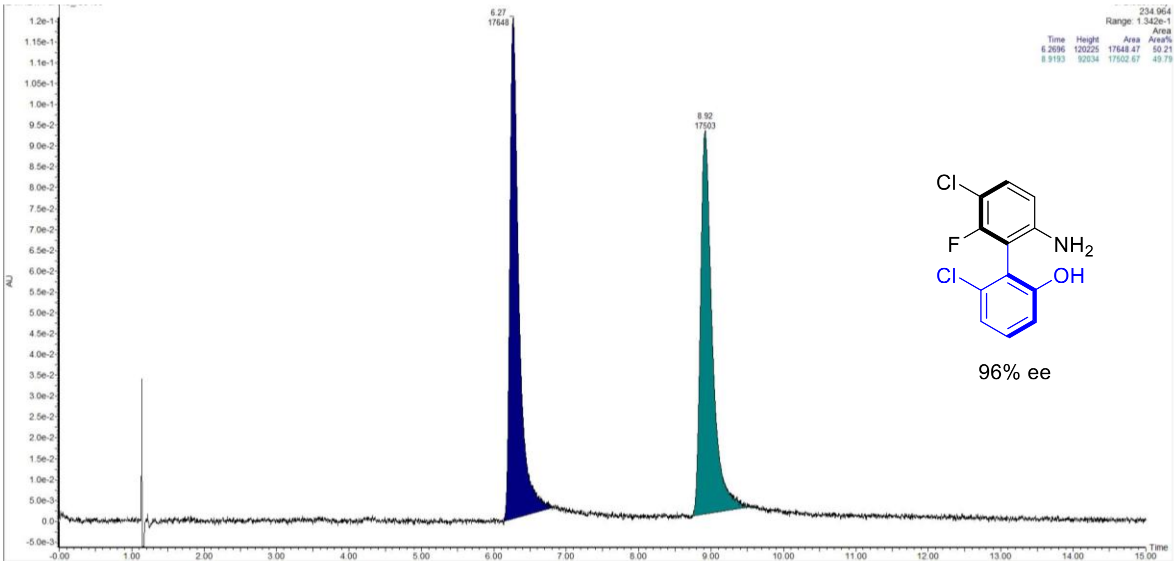

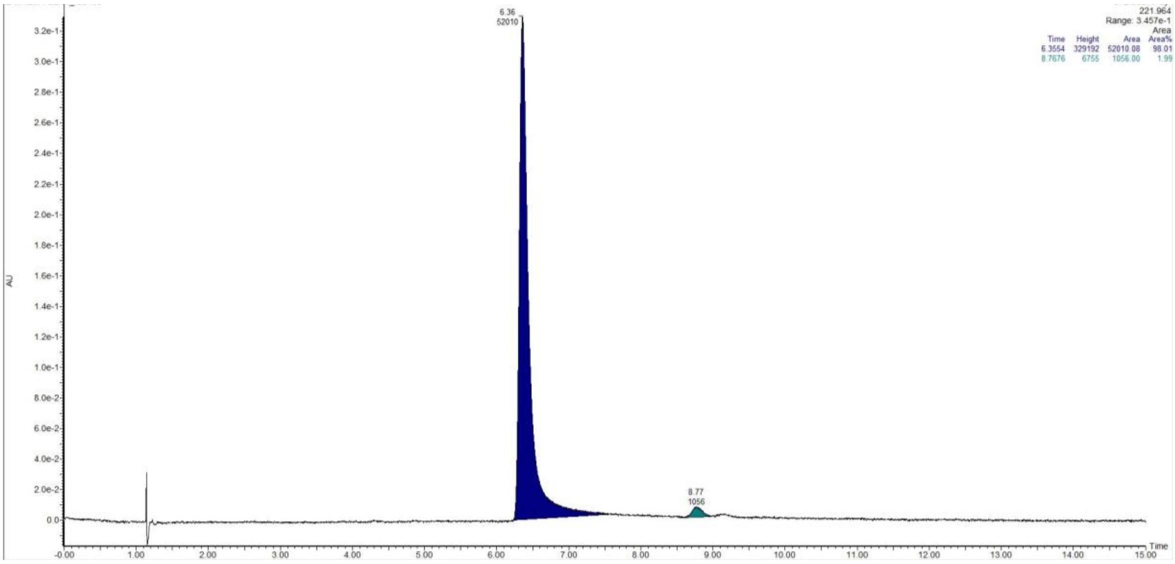

SFC Rac: *(S)*-2'-amino-6-chloro-6'-fluoro-3'-nitro-[1,1'-biphenyl]-2-ol (**3e**)

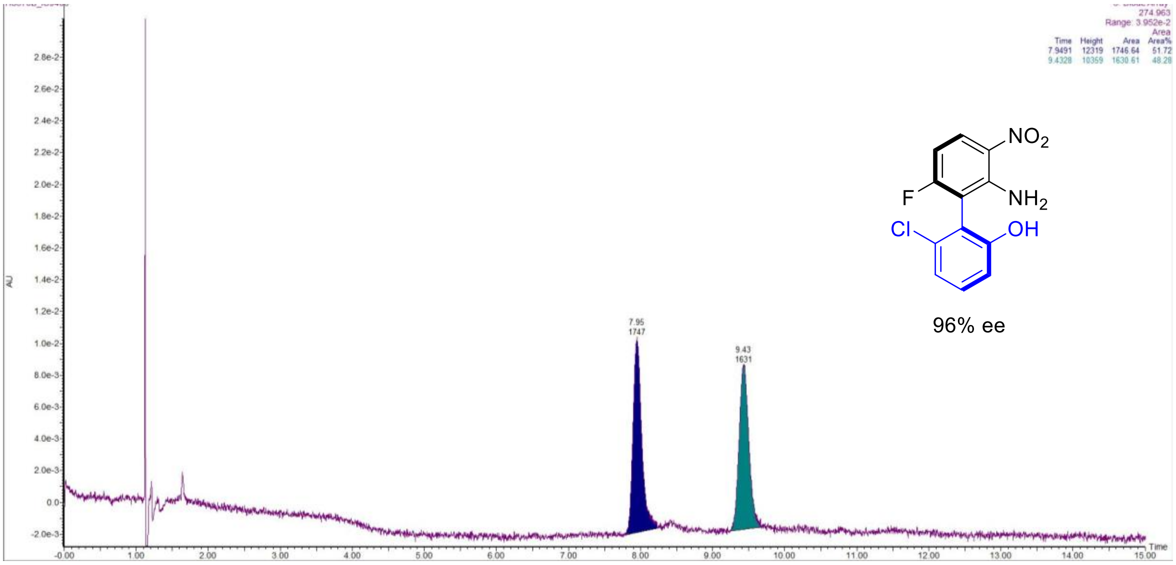

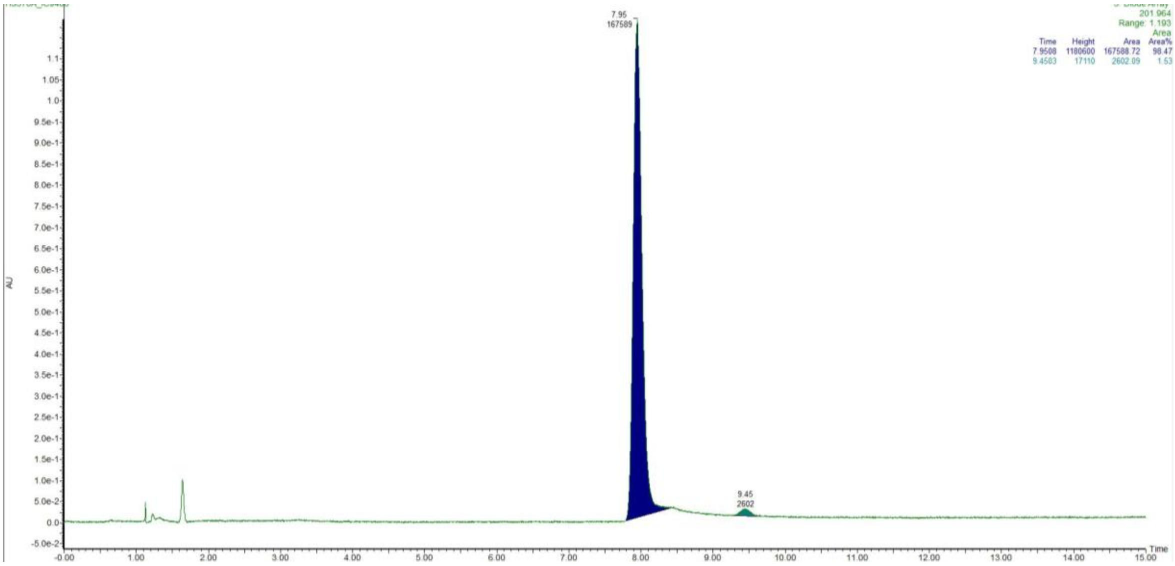

(S)-2'-amino-6'-chloro-6-fluoro-[1,1'-biphenyl]-2-ol (**3f**)

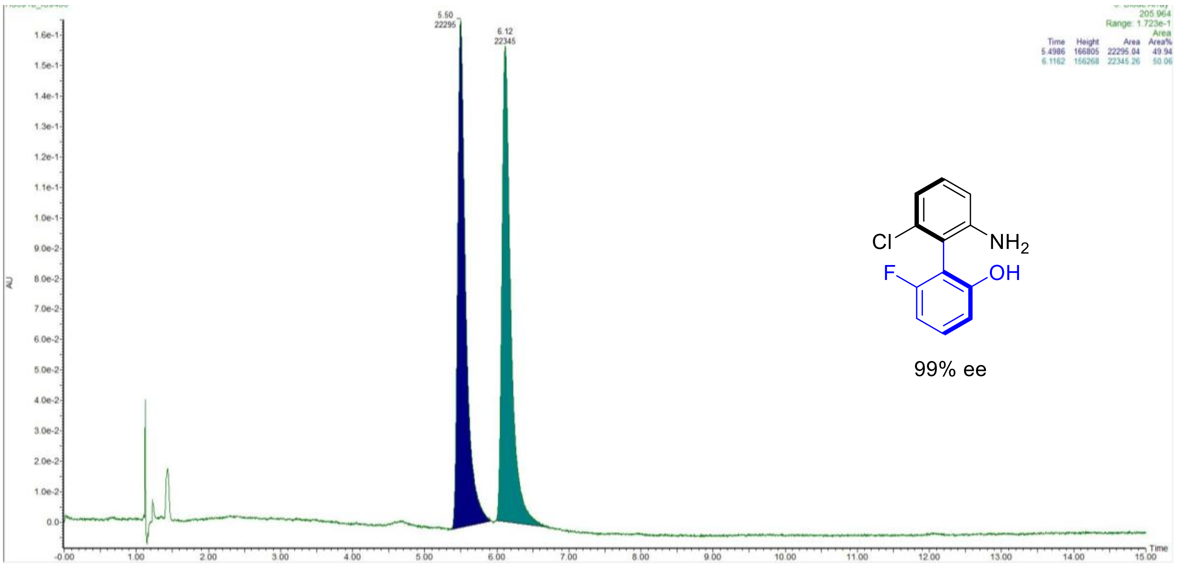

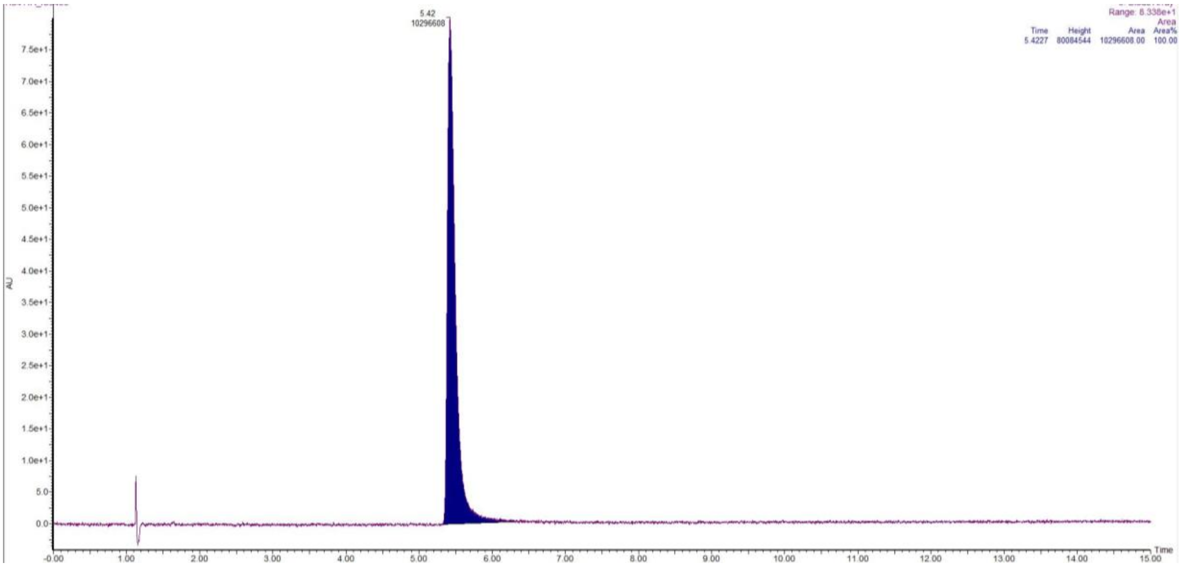

(*S*)-6'-amino-2',3'-dichloro-6-fluoro-[1,1'-biphenyl]-2-ol (**3g**)

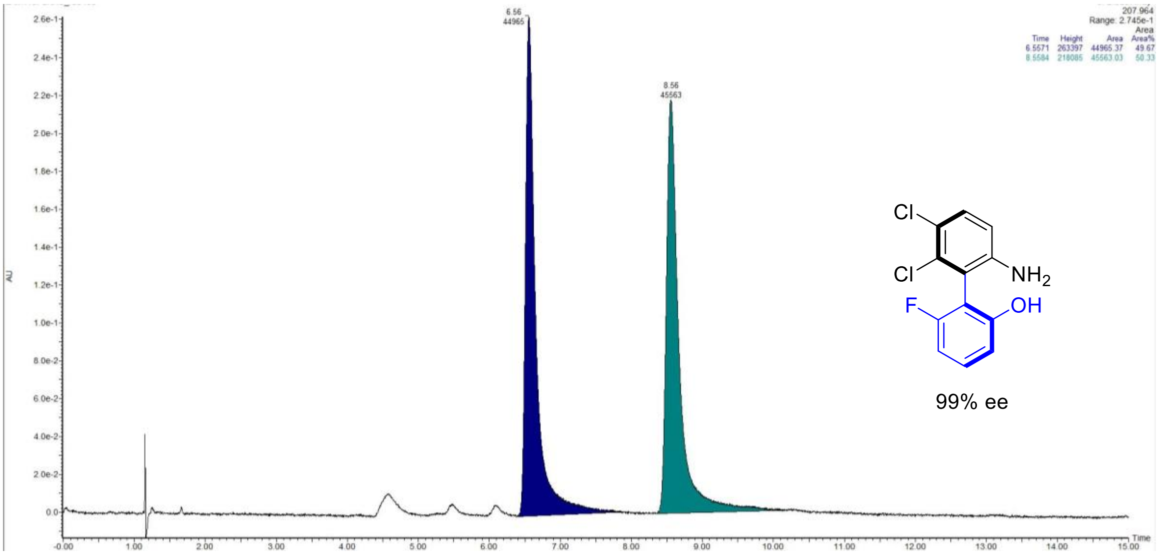

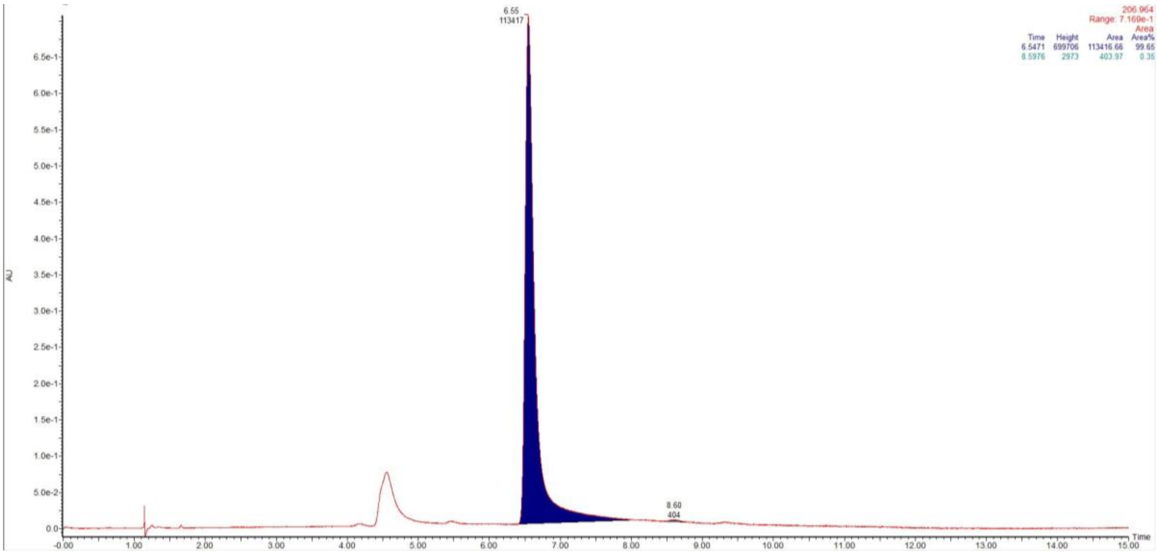

(S)-2'-amino-4',6'-dichloro-6-fluoro-[1,1'-biphenyl]-2-ol (**3h**)

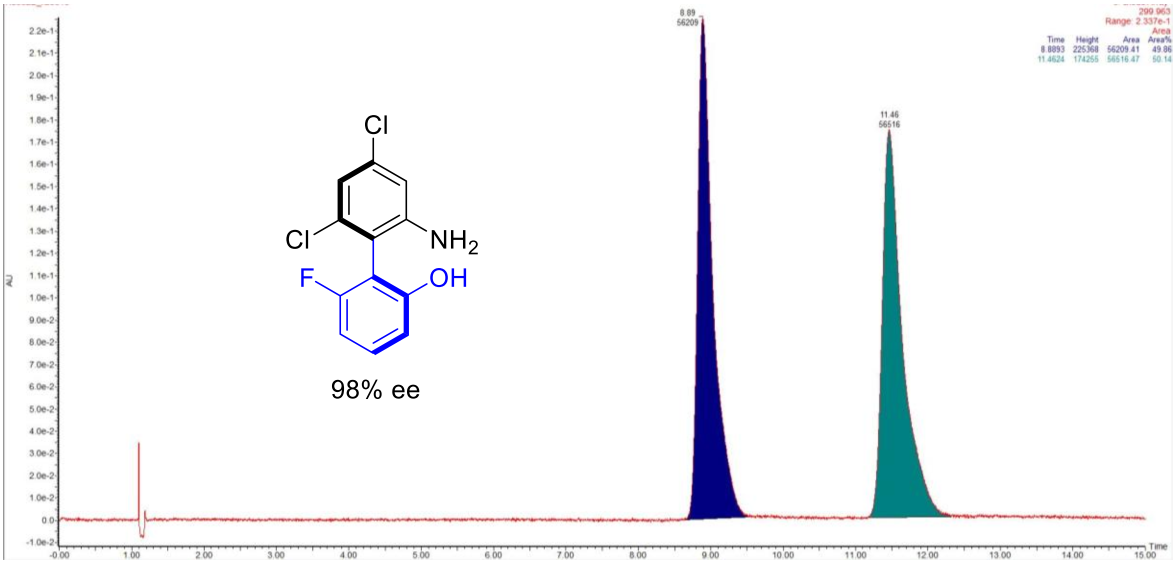

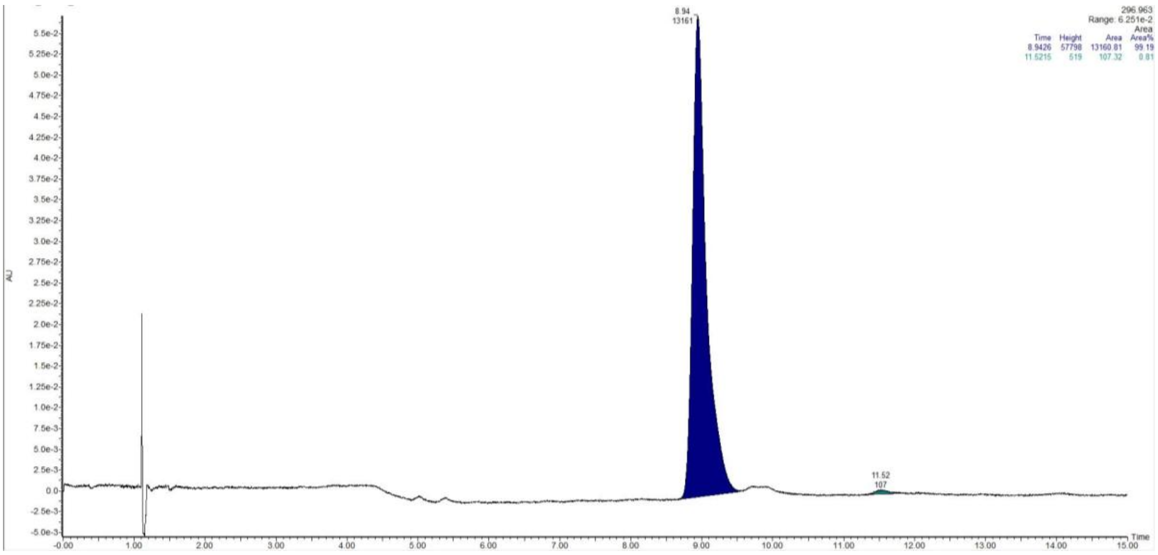

(R)-2'-amino-6-fluoro-6'-methyl-[1,1'-biphenyl]-2-ol (**3i**)

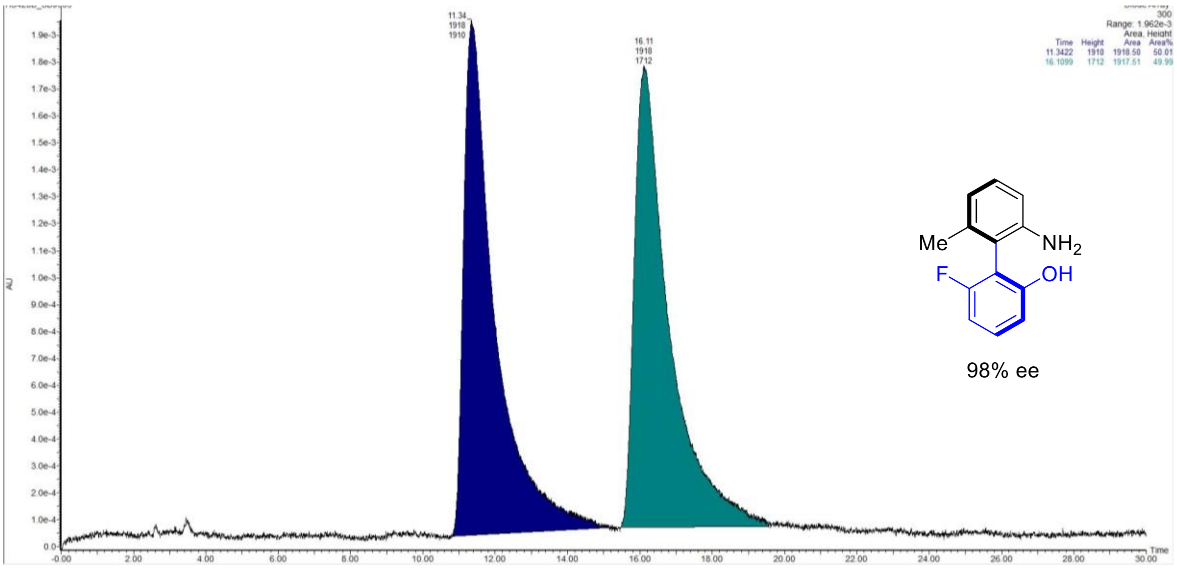

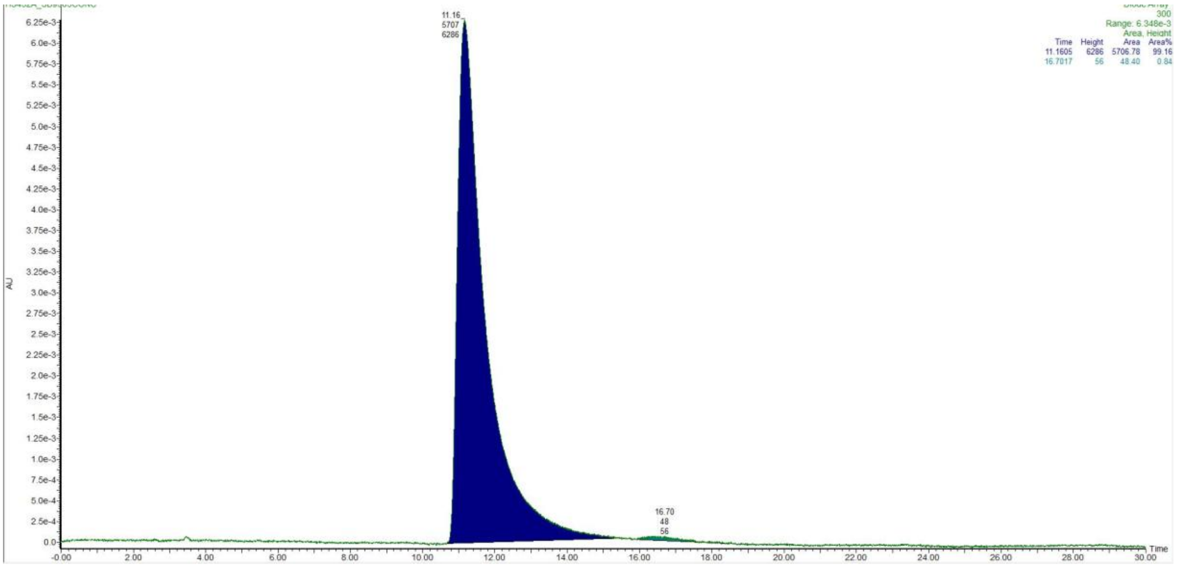

(R)-2-(2-amino-5,6,7,8-tetrahydronaphthalen-1-yl)-3-fluorophenol (**3j**)

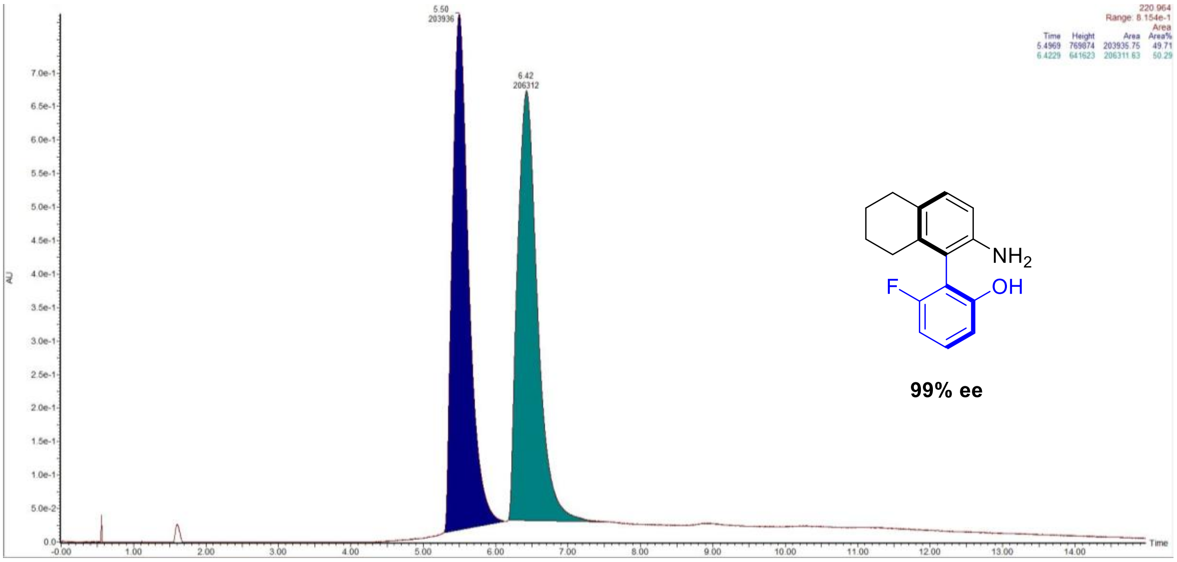

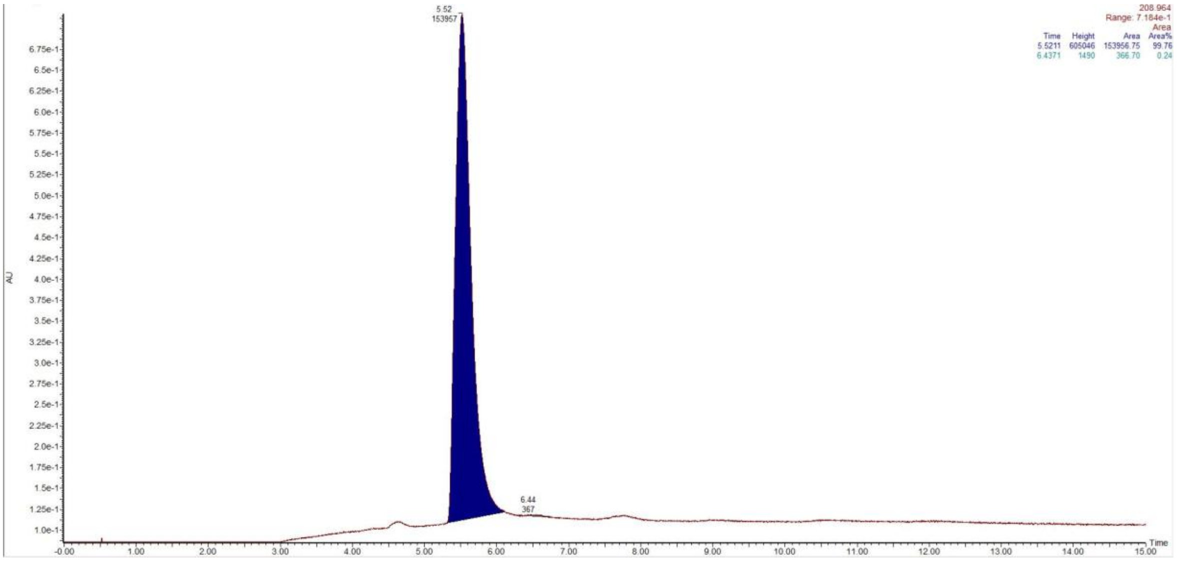

(R)-6'-amino-3'-chloro-6-fluoro-2'-methyl-[1,1'-biphenyl]-2-ol (**3k**)

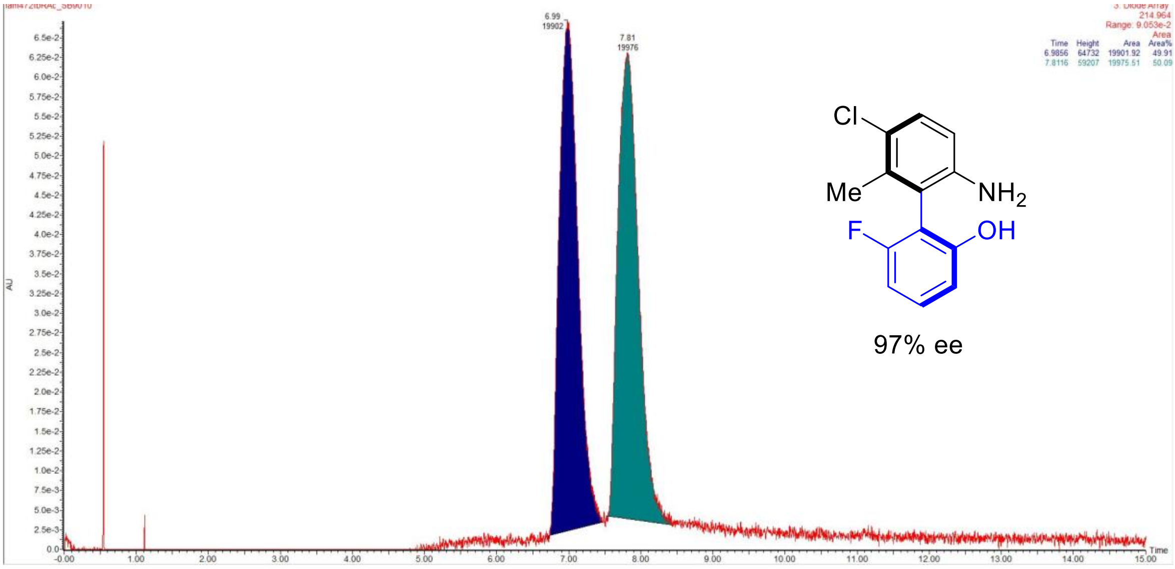

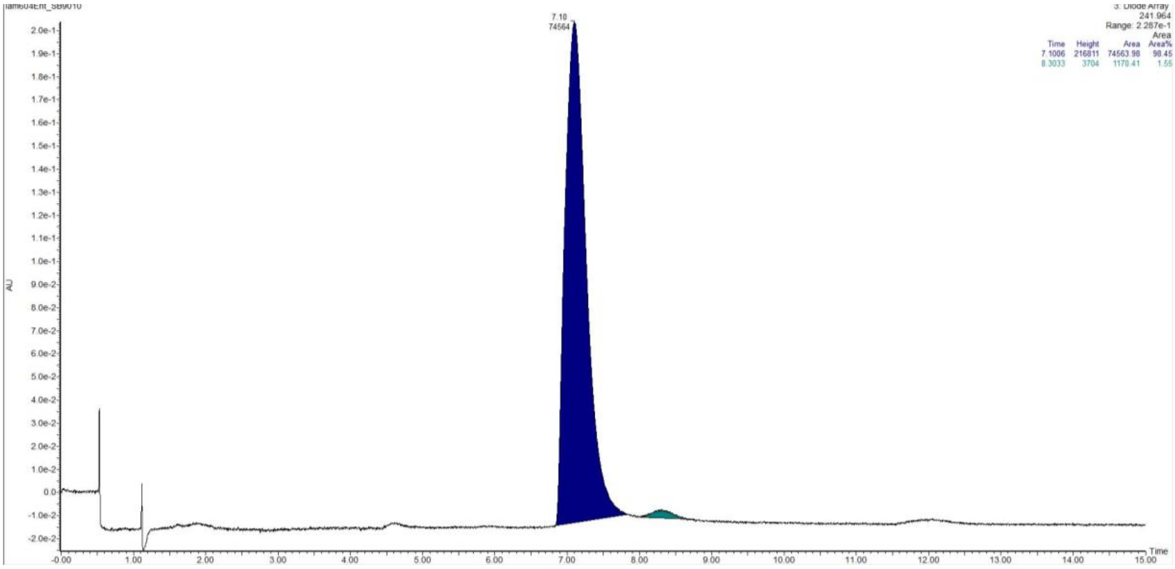

(S)-2'-amino-4',6'-dichloro-5,6-difluoro-[1,1'-biphenyl]-2-ol (**3l**)

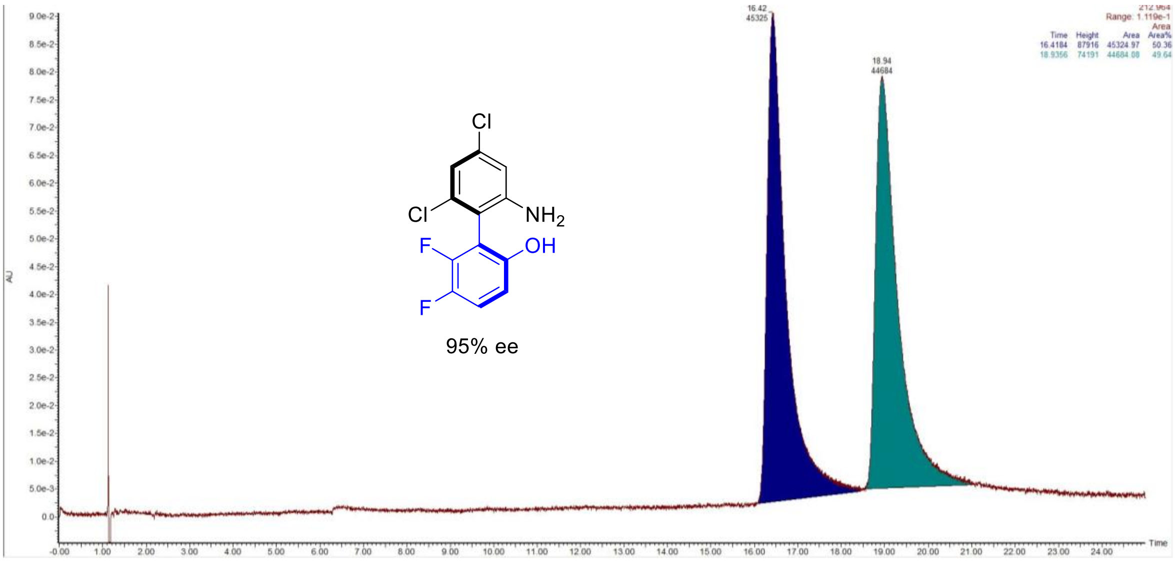

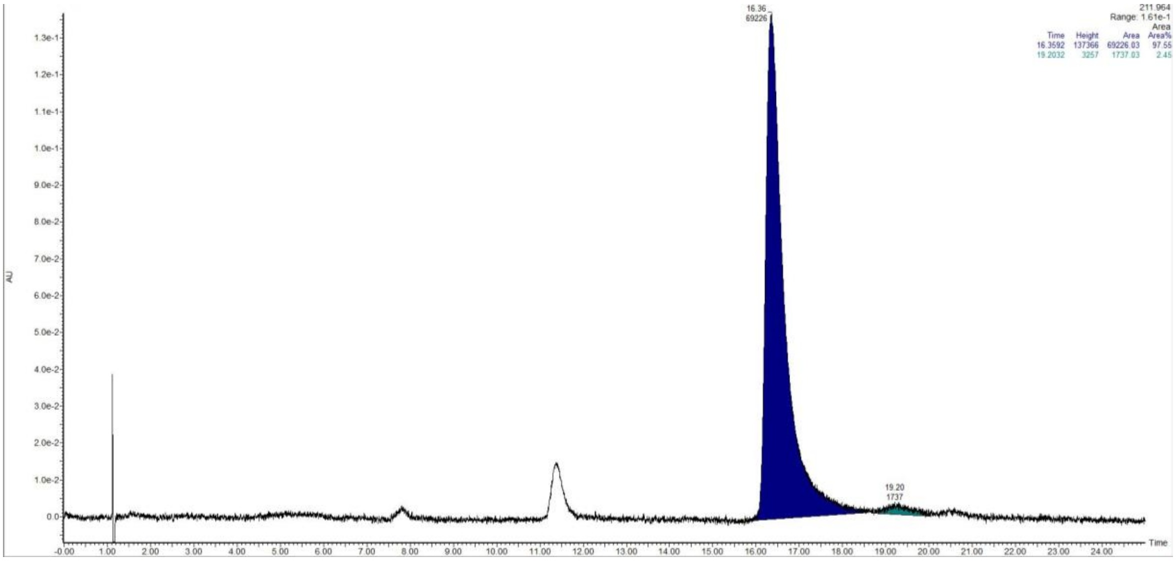

(S)-2'-amino-6'-chloro-5,6-difluoro-[1,1'-biphenyl]-2-ol (**3m**)

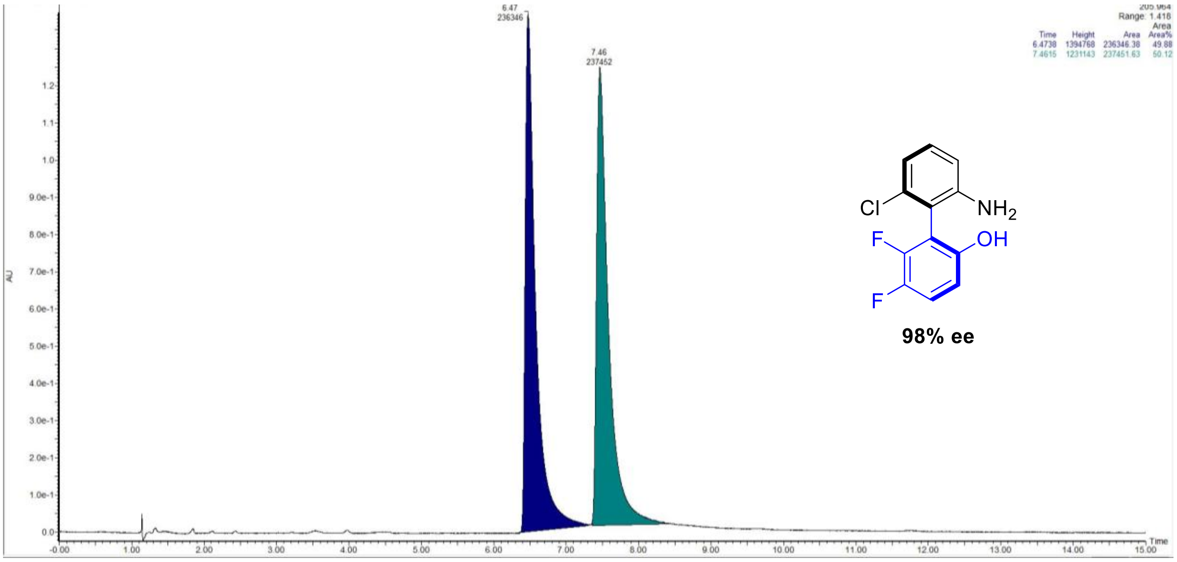

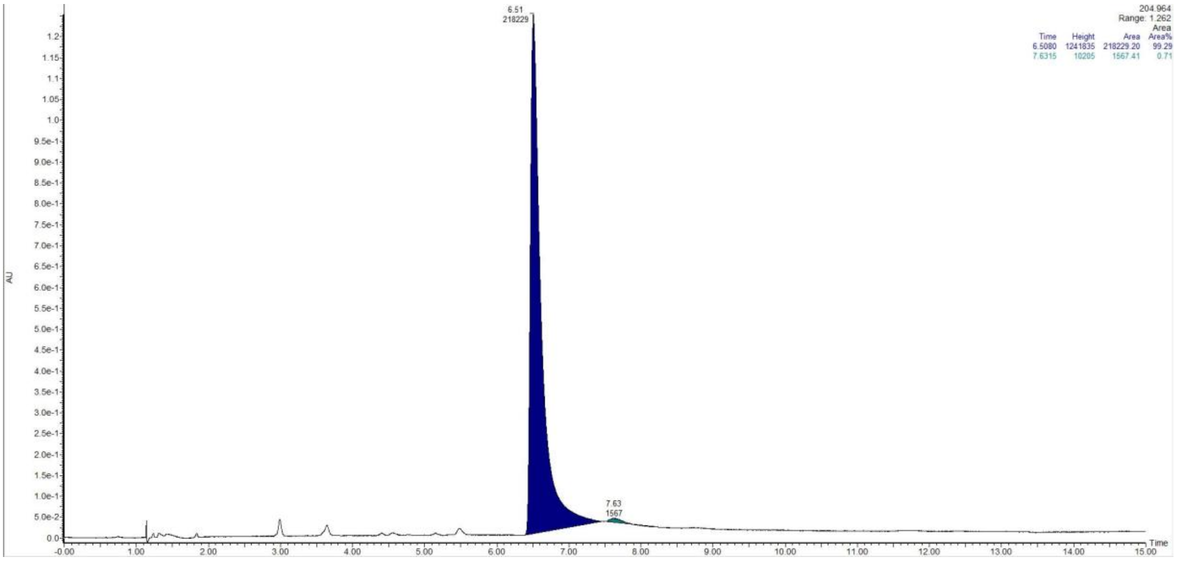

(R)-2'-amino-5,6-difluoro-6'-methyl-[1,1'-biphenyl]-2-ol (**3n**)

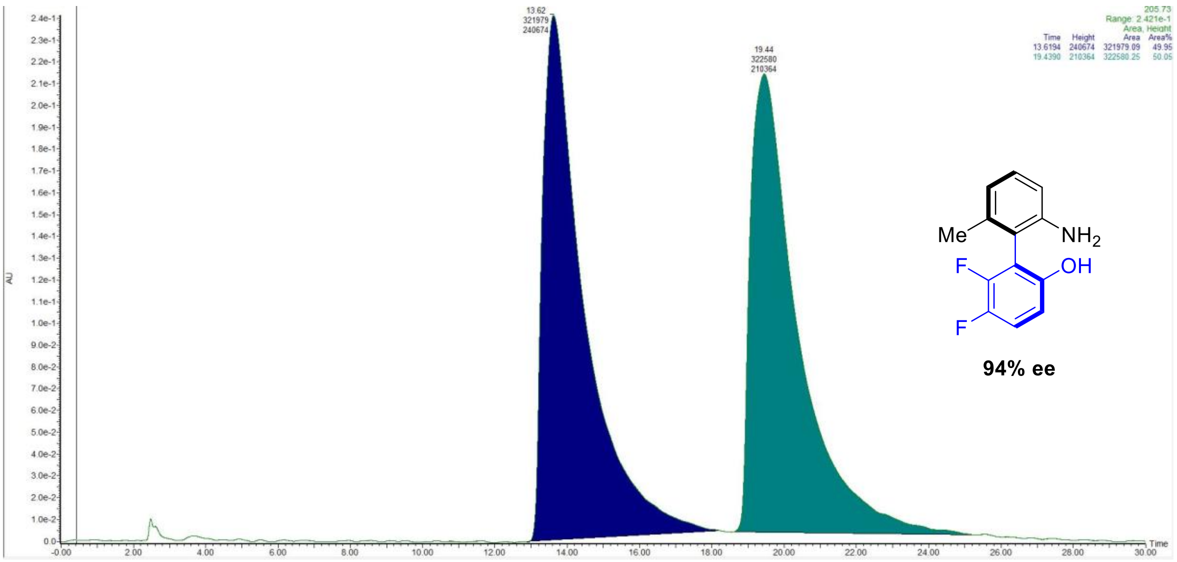

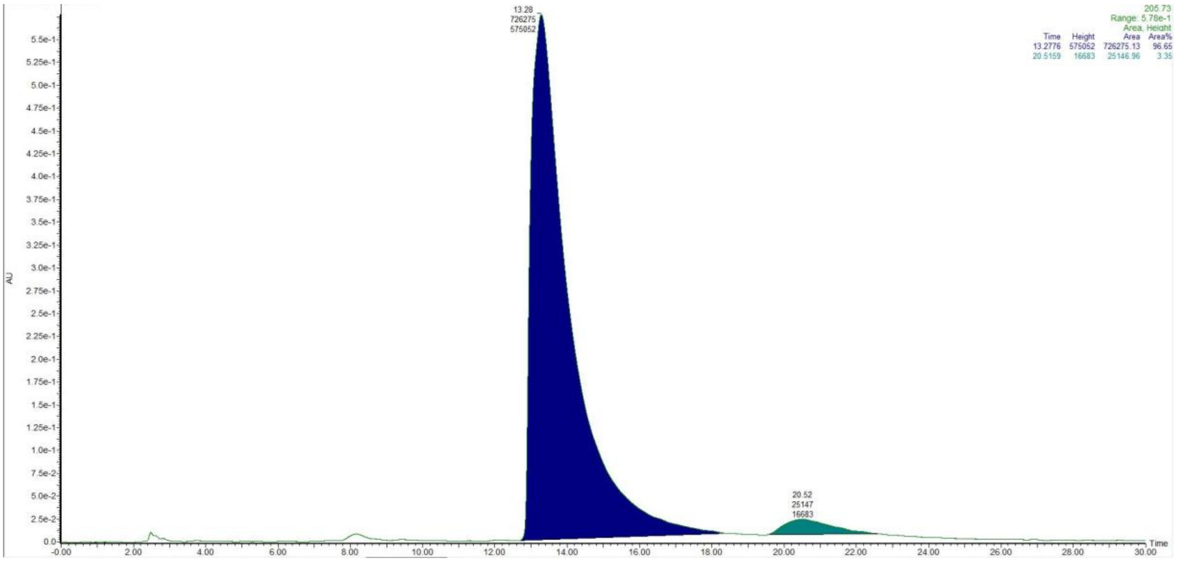

(R)-2-(2-amino-5,6,7,8-tetrahydronaphthalen-1-yl)-3,4-difluorophenol (**3o**)

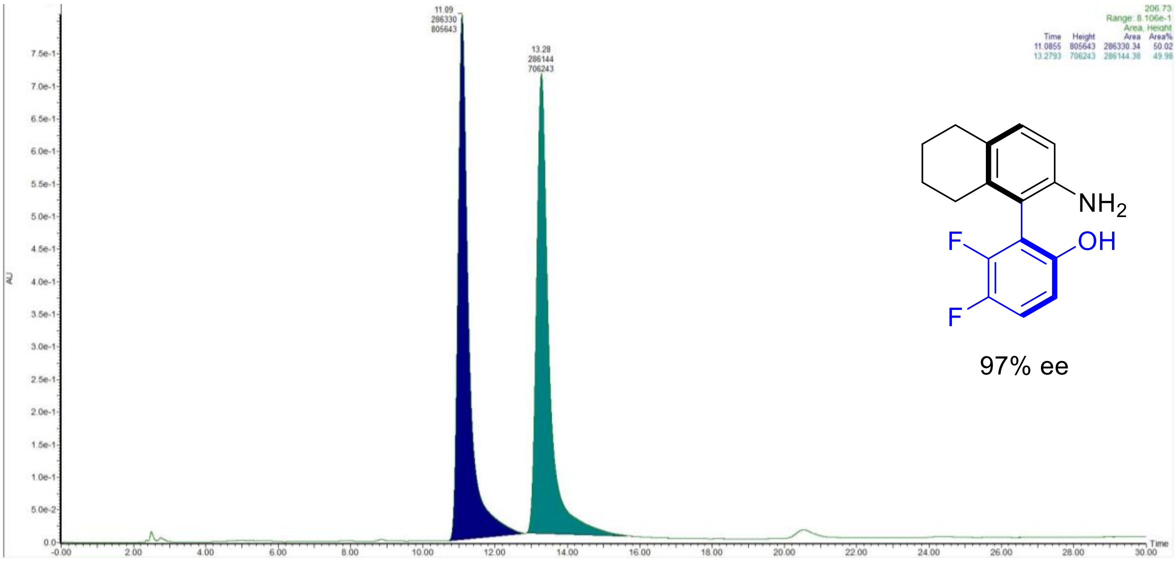

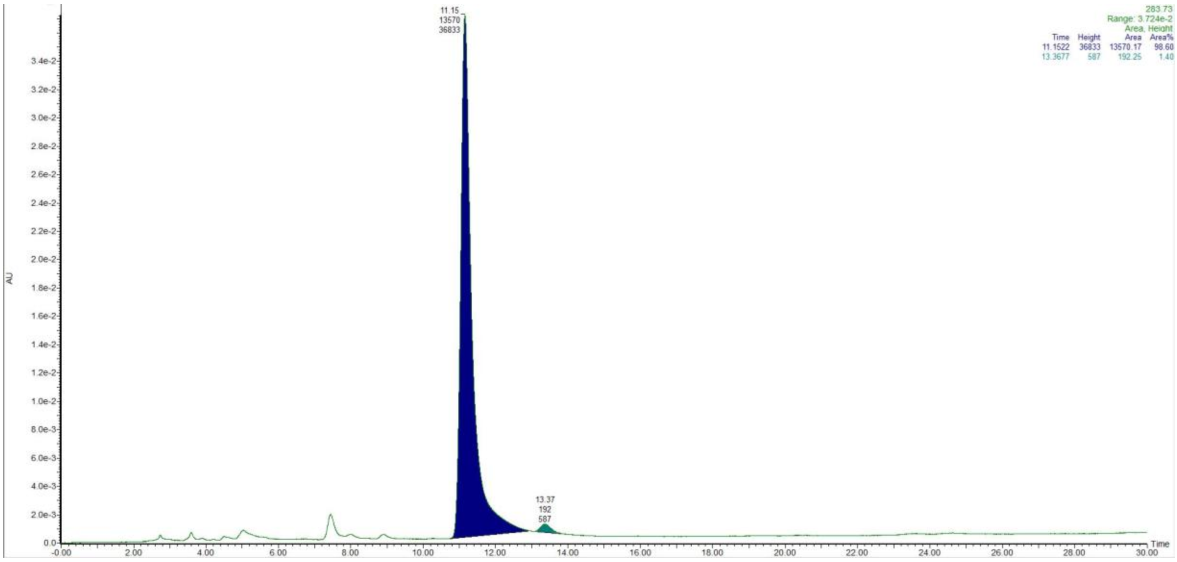

(*R*)-2'-amino-6'-fluoro-6-methyl-[1,1'-biphenyl]-2-ol (**3p**)

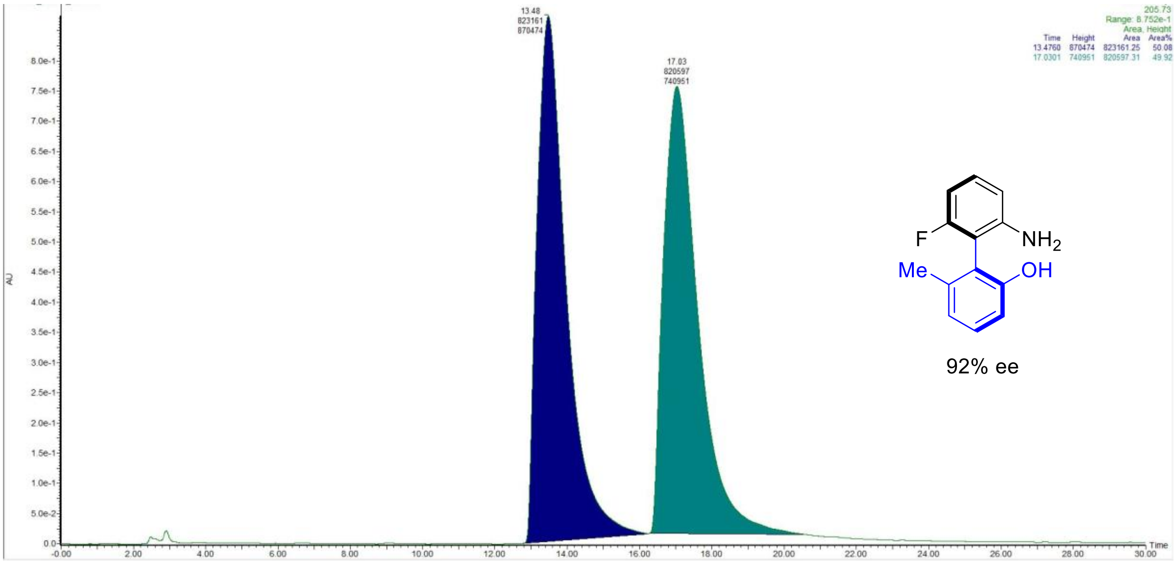

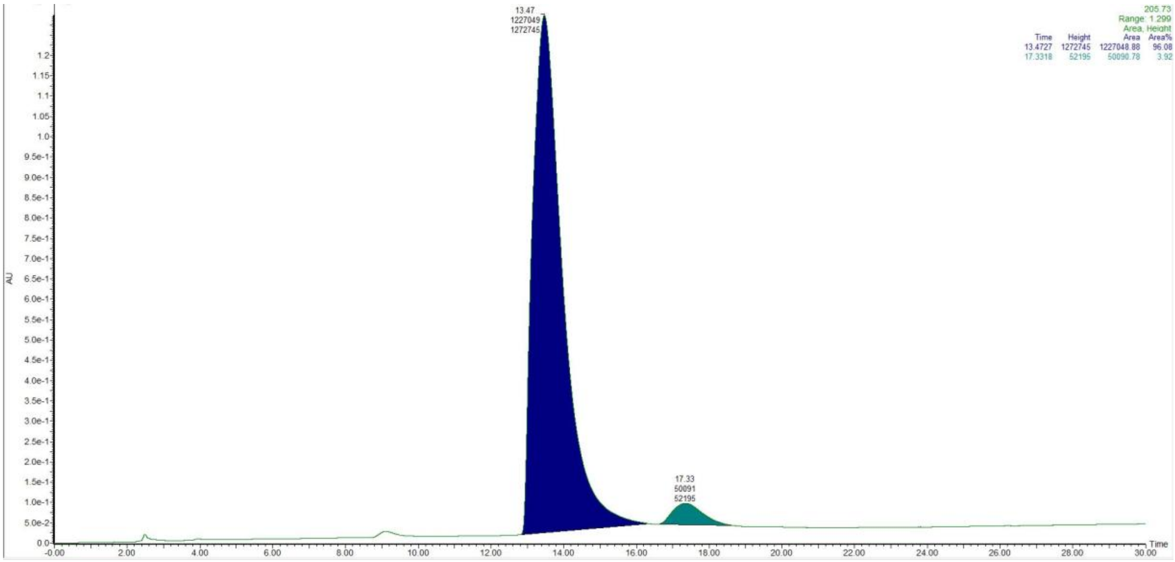

(*R*)-1-(2-amino-6-fluorophenyl)-5,6,7,8-tetrahydronaphthalen-2-ol (**3q**)

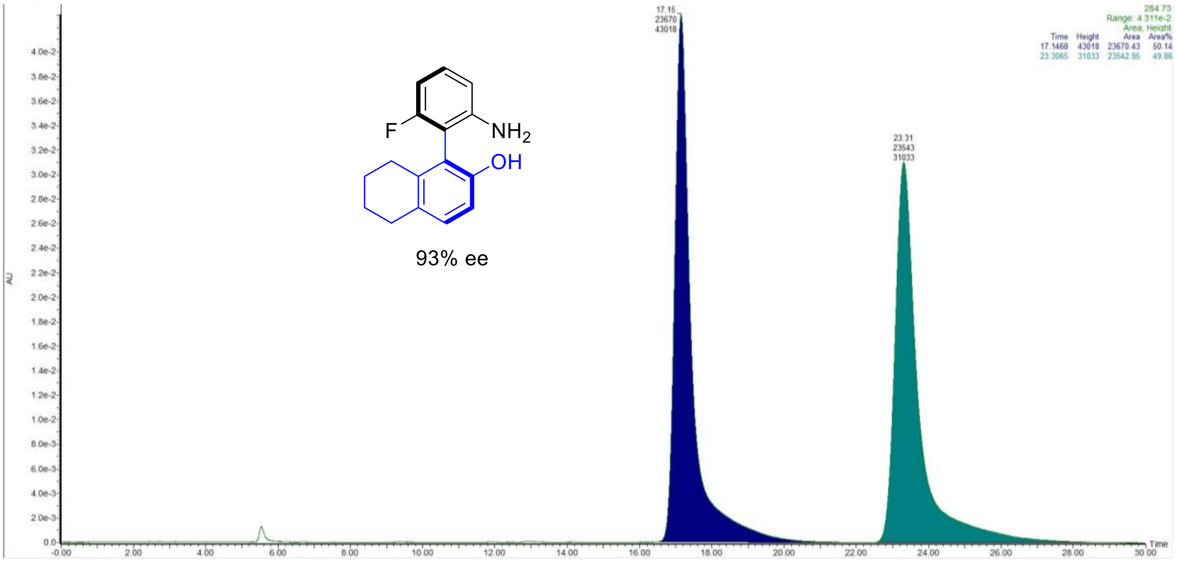

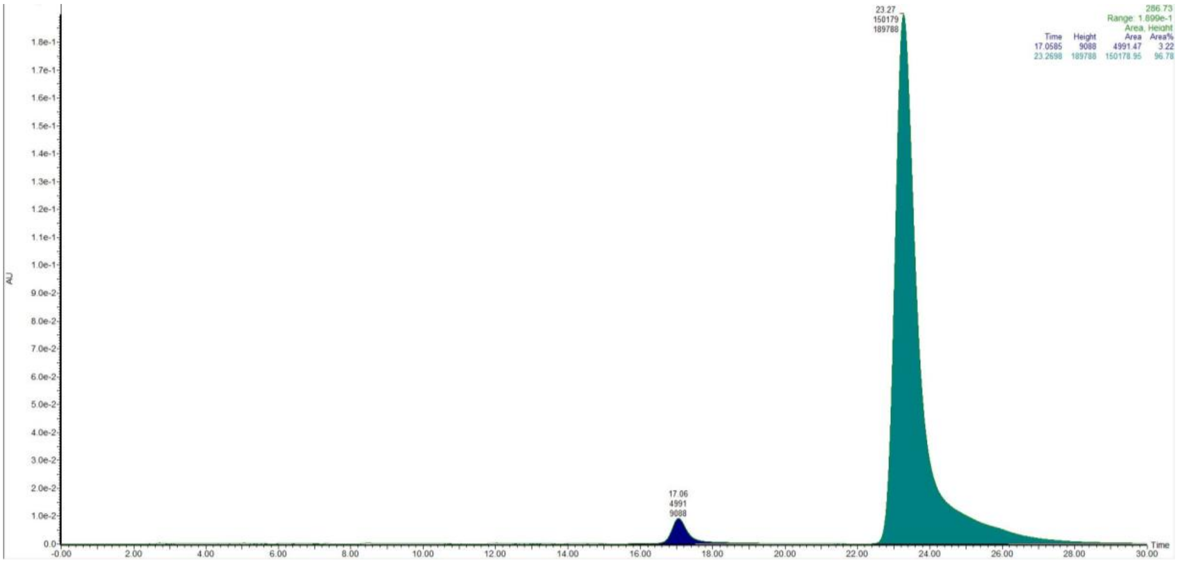

(S)-2'-amino-3',5'-dichloro-6-methyl-[1,1'-biphenyl]-2-ol (**3r**)

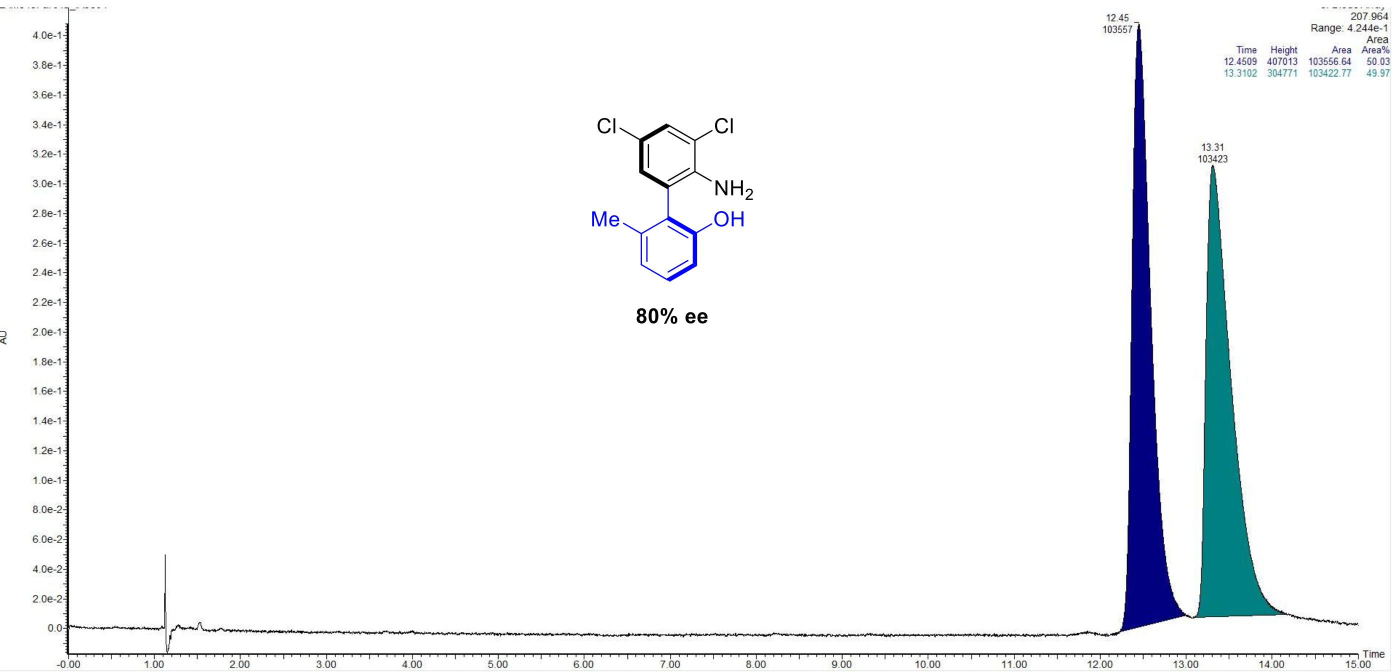

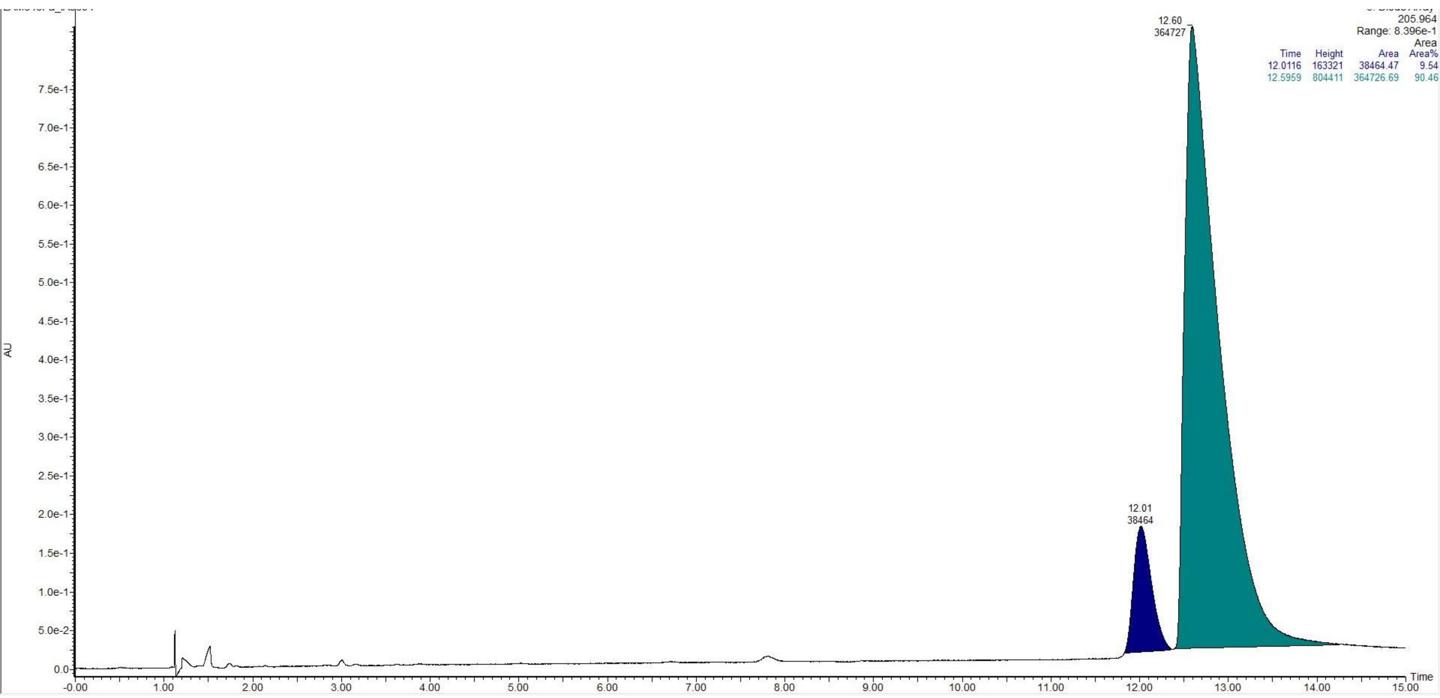

(S)-6-chloro-2'-fluoro-6'-(methylamino)-[1,1'-biphenyl]-2-ol (**3s**)

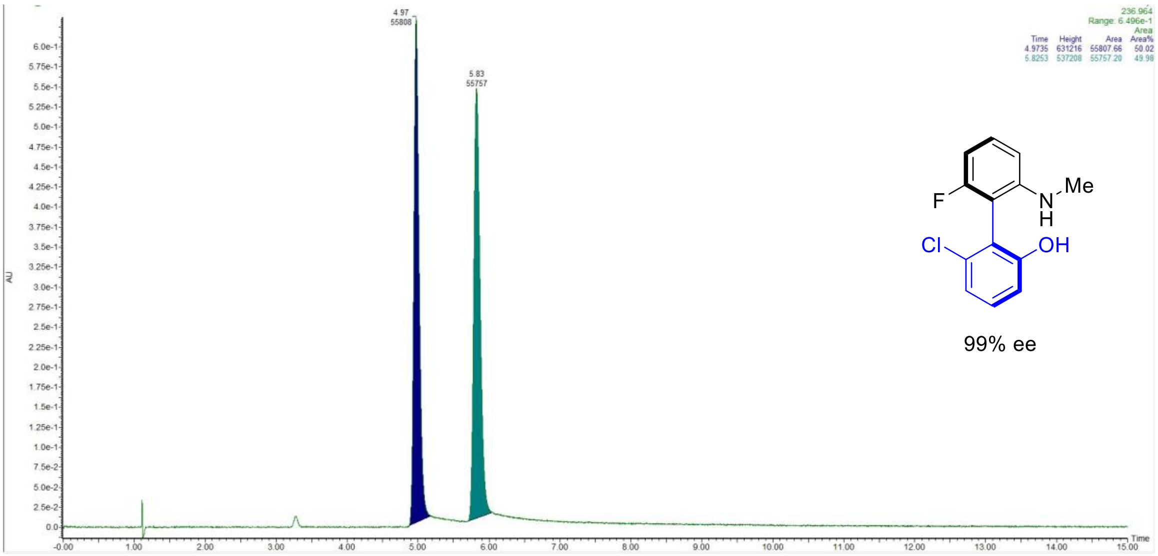

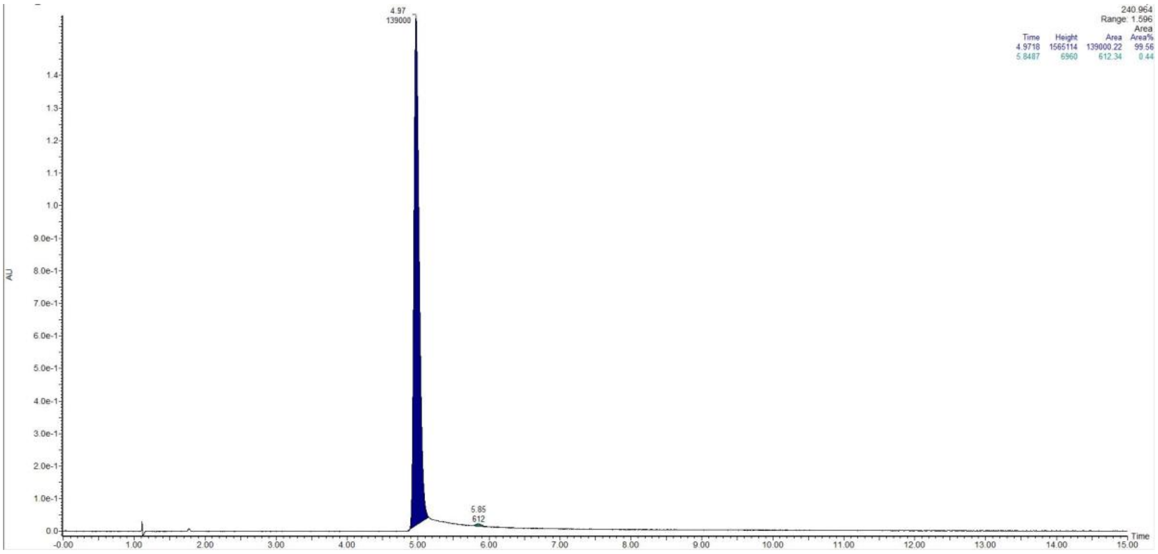

(R)-6-fluoro-2'-methyl-6'-(methylamino)-[1,1'-biphenyl]-2-ol (**3t**)

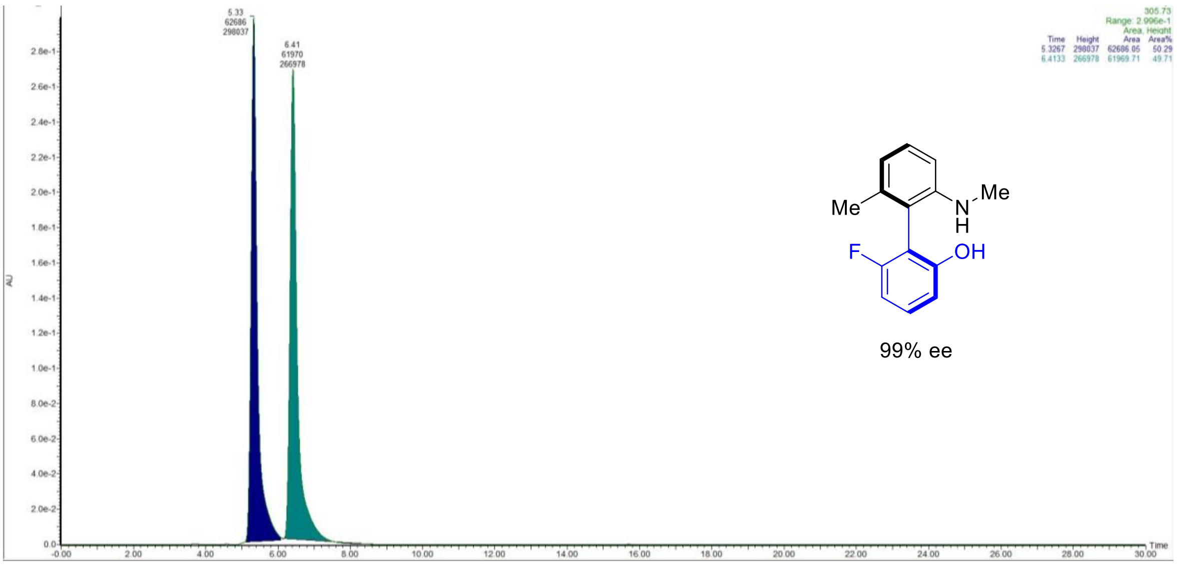

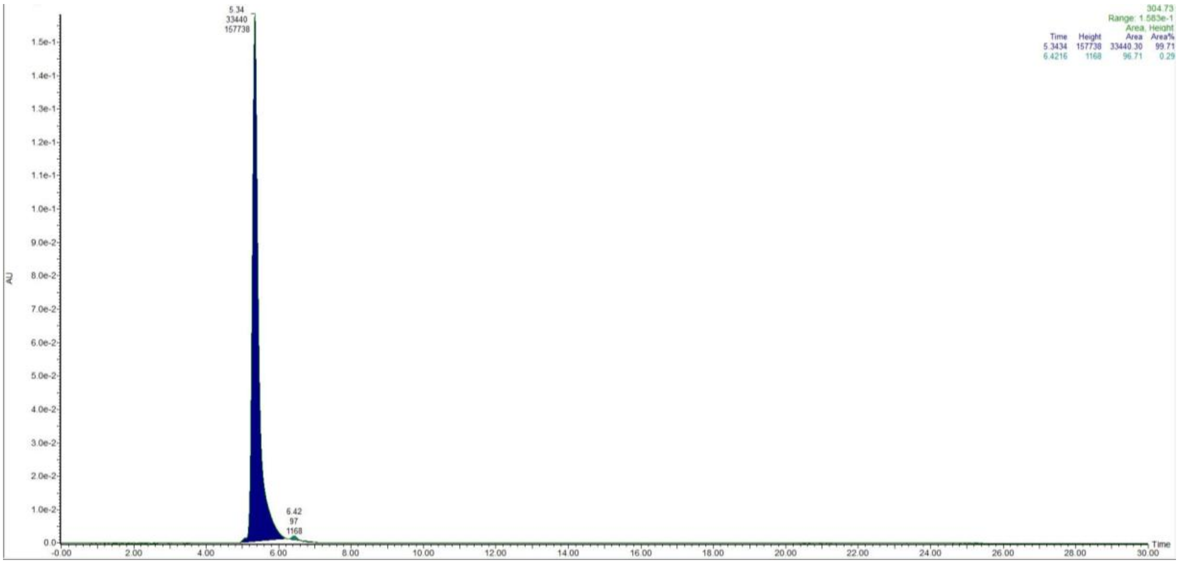

(S)-6-chloro-2'-(ethylamino)-6'-fluoro-[1,1'-biphenyl]-2-ol (**3u**)

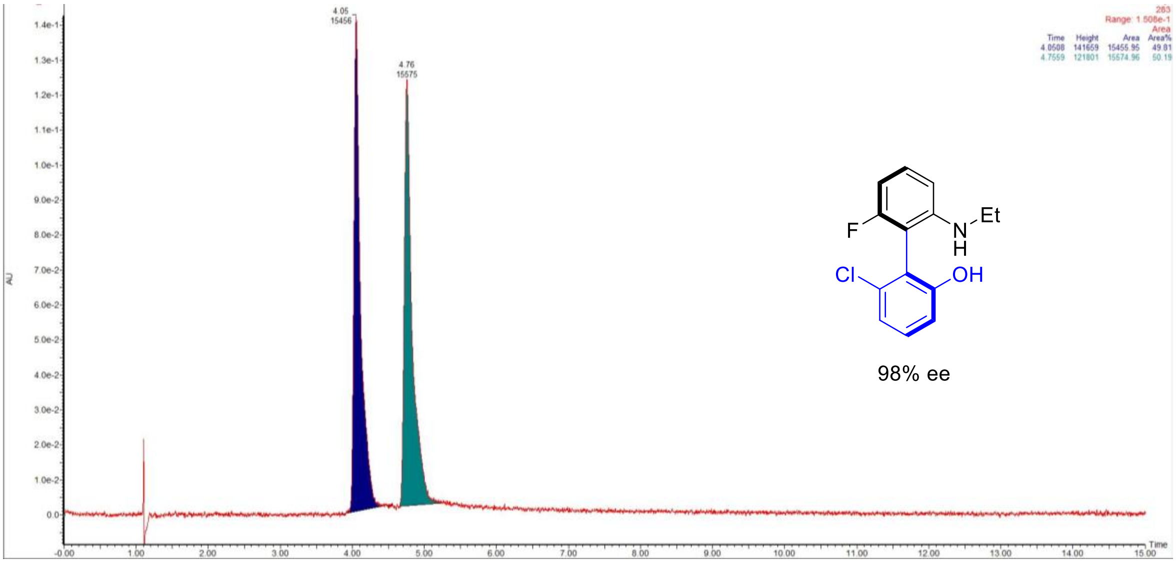

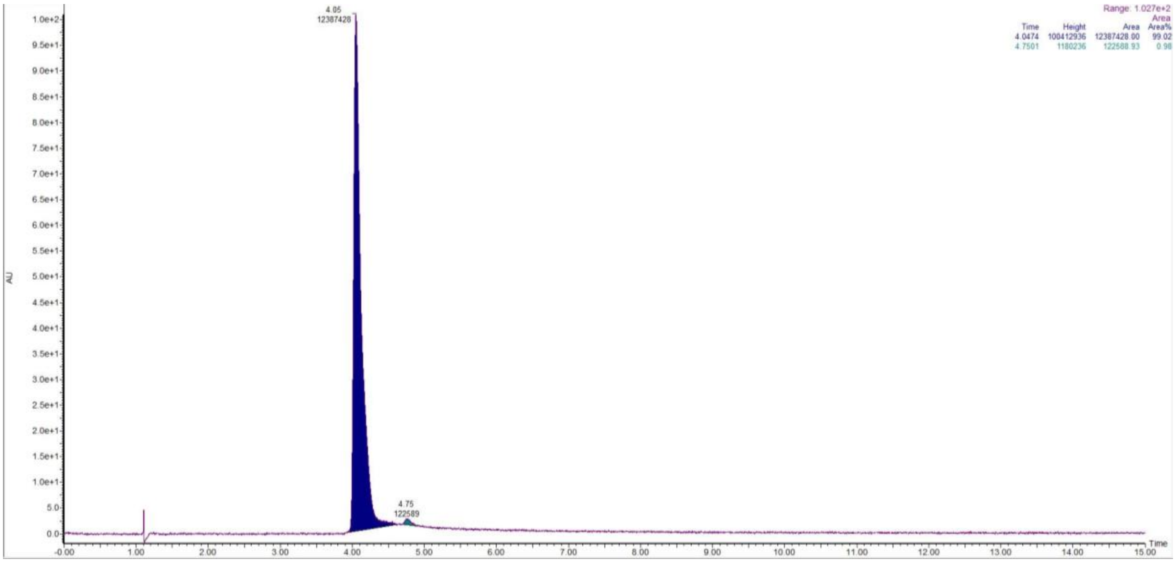

(R)-2'-(ethylamino)-6-fluoro-6'-methyl-[1,1'-biphenyl]-2-ol (**3v**)

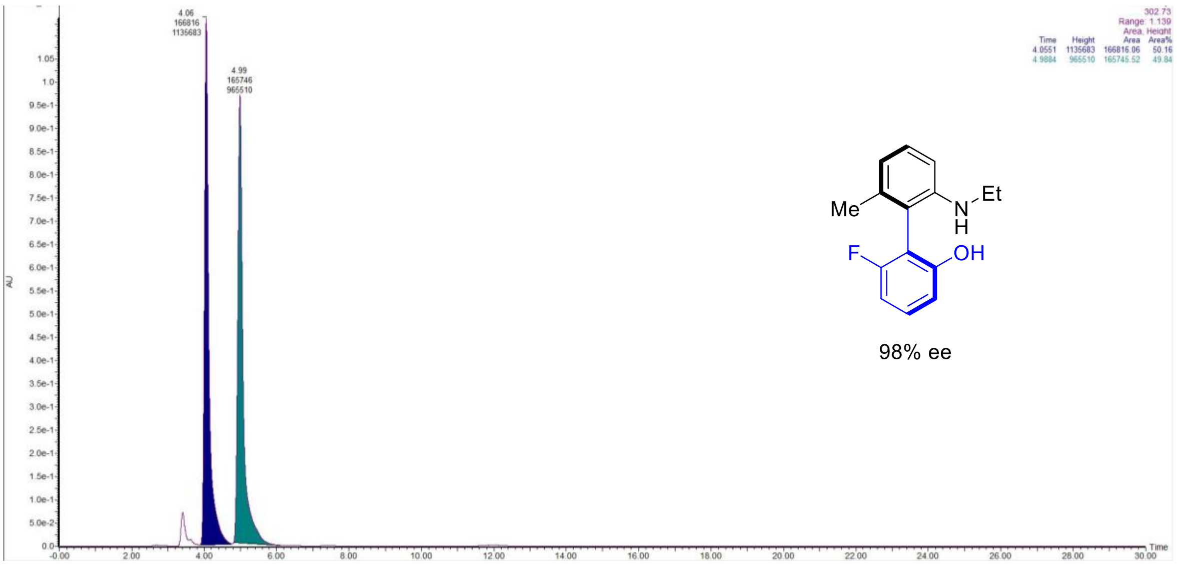

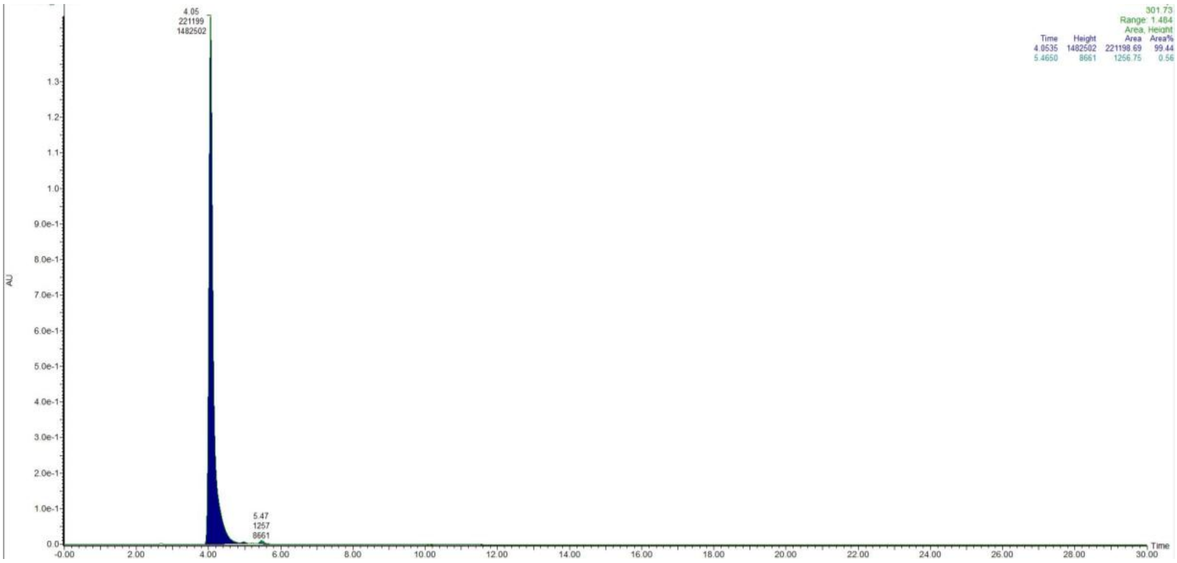

(S)-2'-(benzylamino)-6-chloro-6'-fluoro-[1,1'-biphenyl]-2-ol (**3w**)

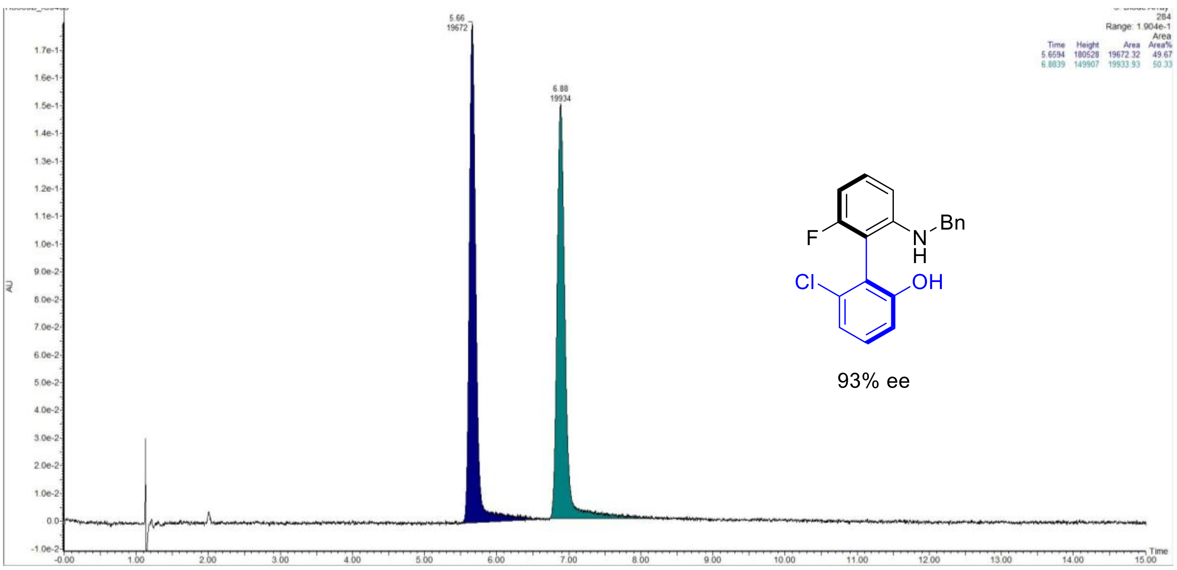

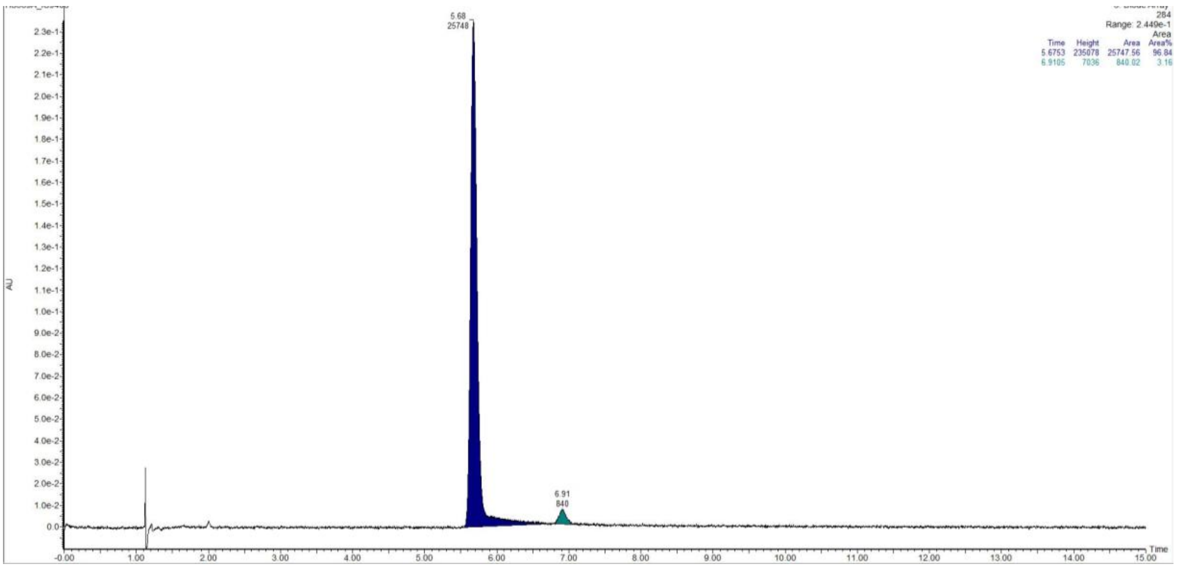

(*R*)-6-fluoro-2'-methyl-6'-nitro-[1,1'-biphenyl]-2-ol (**3y**)

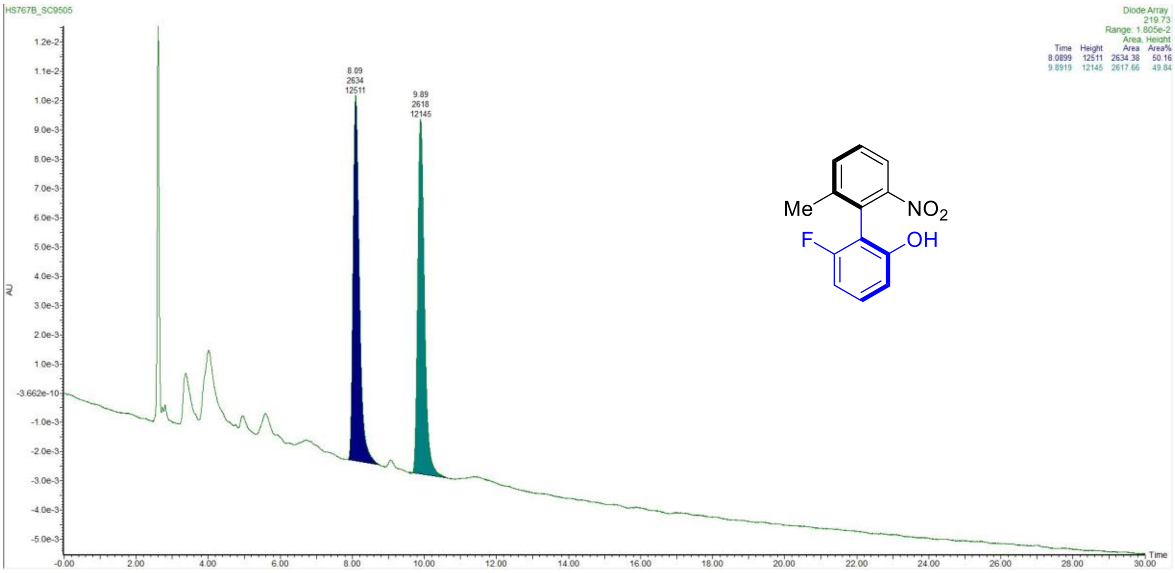

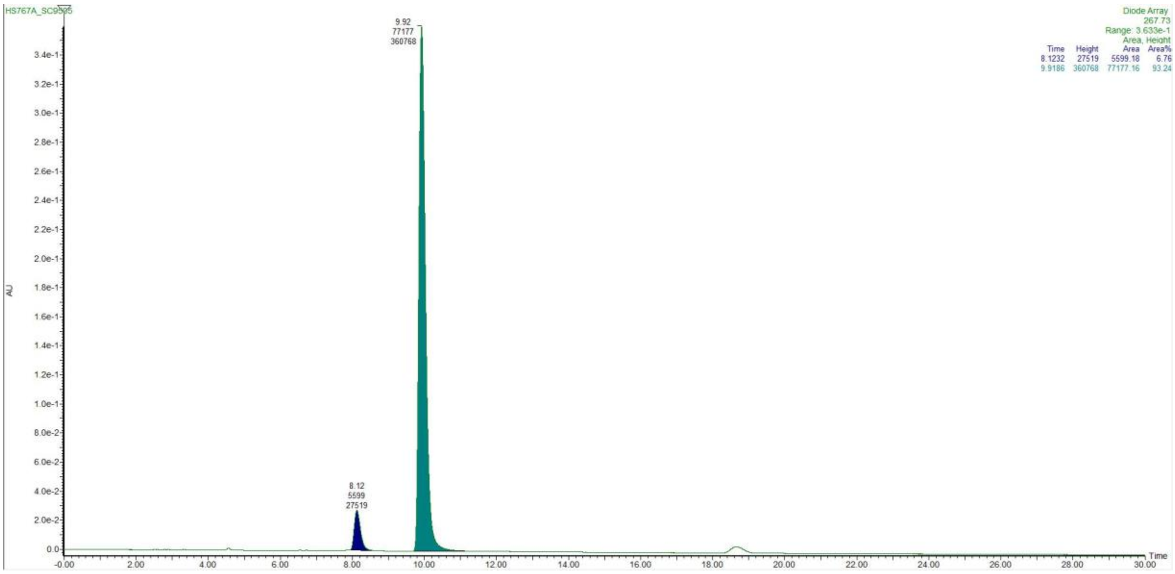

(R)-2-(2-aminonaphthalen-1-yl)-3-fluorophenol (**3z**)

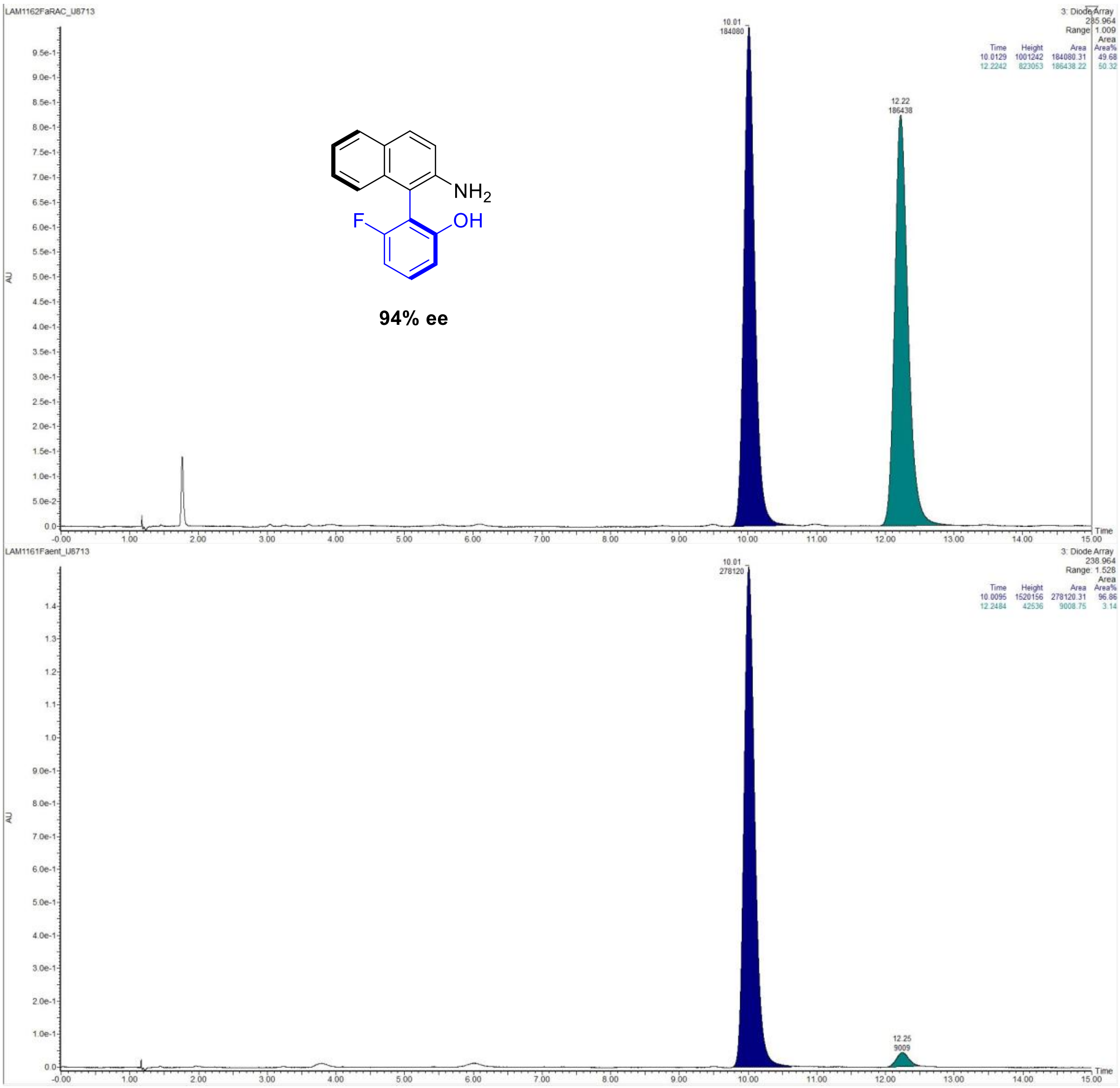

<u>*(R)*-3-chloro-2-(6-methylindolin-7-yl)phenol (**3za**)</u>

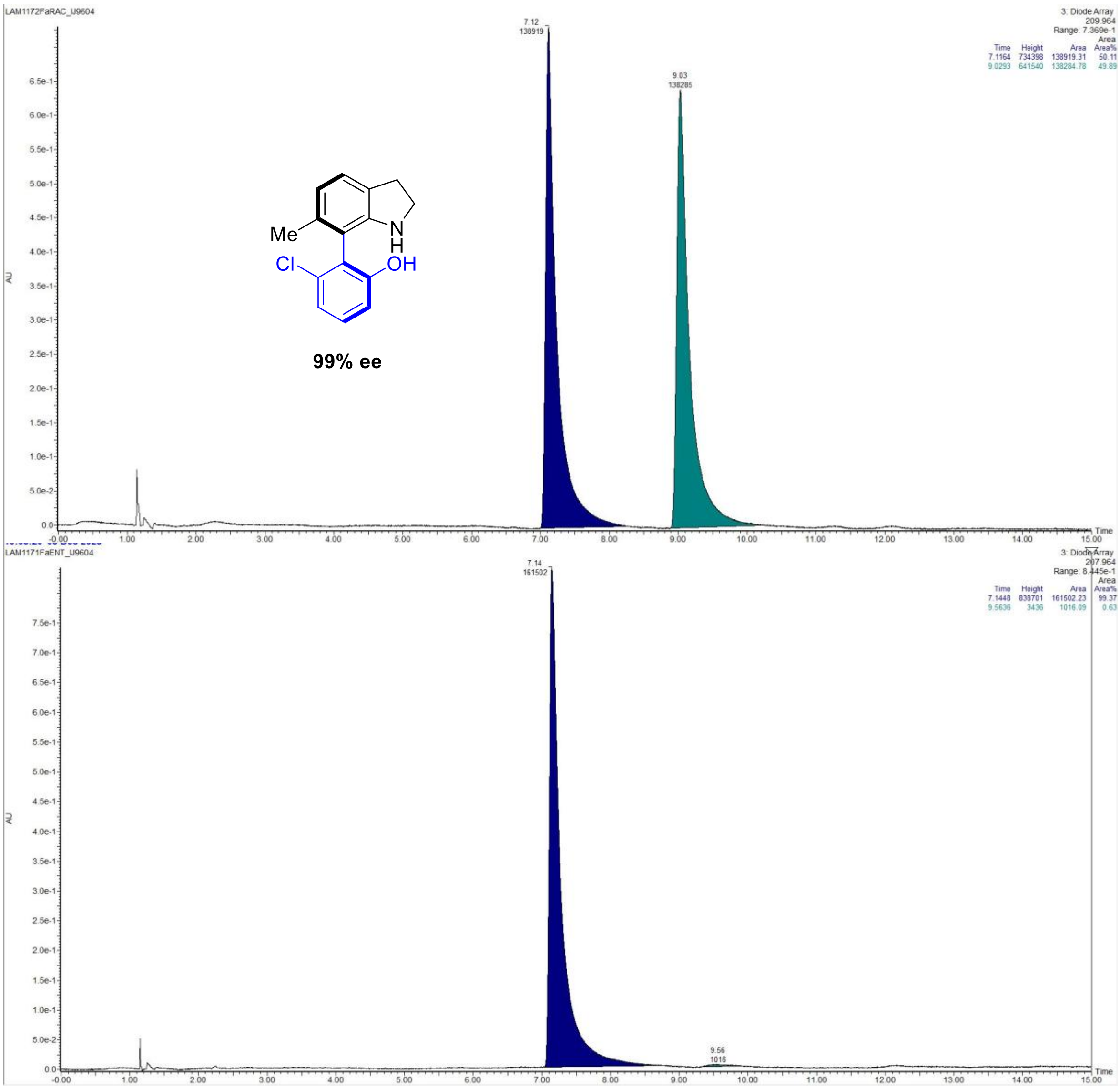

<u>*(R)*-3-chloro-2-(6-methyl-1H-indol-7-yl)phenol (**3zb**)</u>

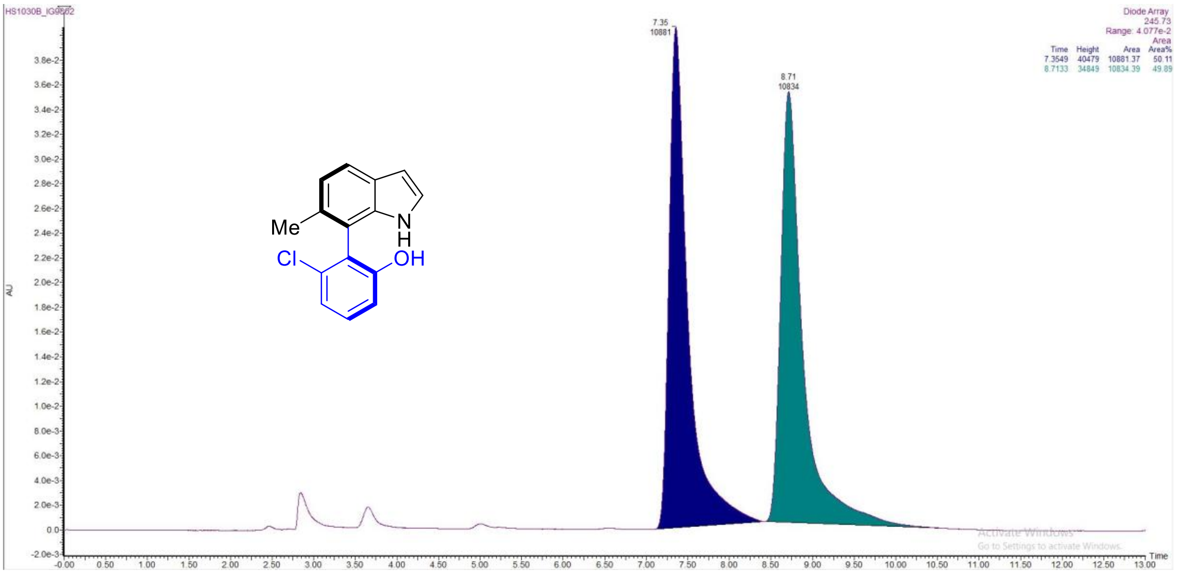

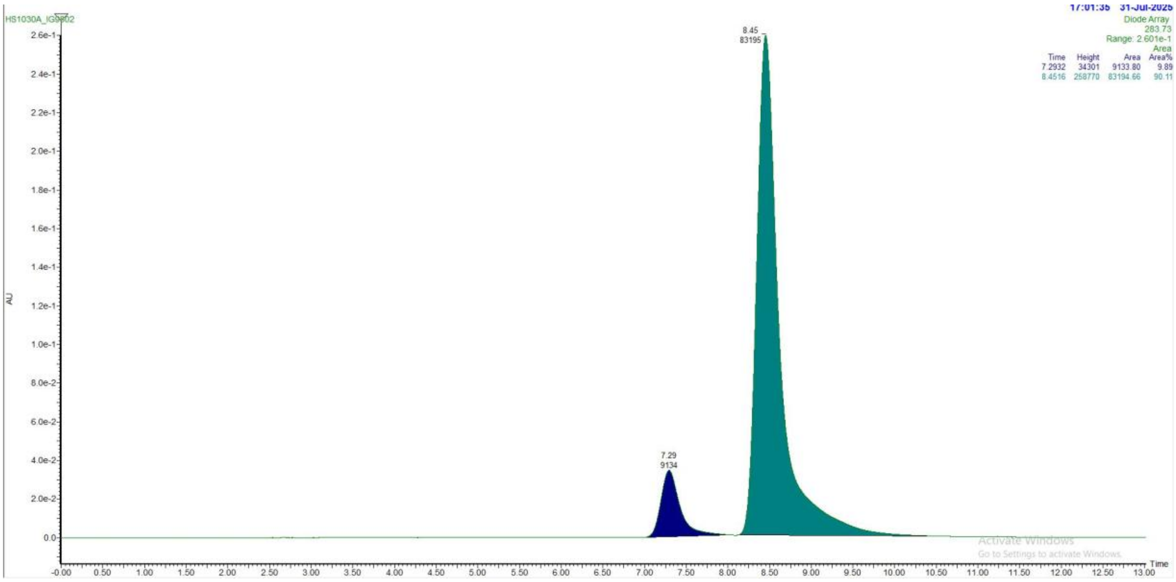

<u>*(S)*-2-(2-amino-4-chloropyridin-3-yl)-3-fluorophenol (**3zc**)</u>

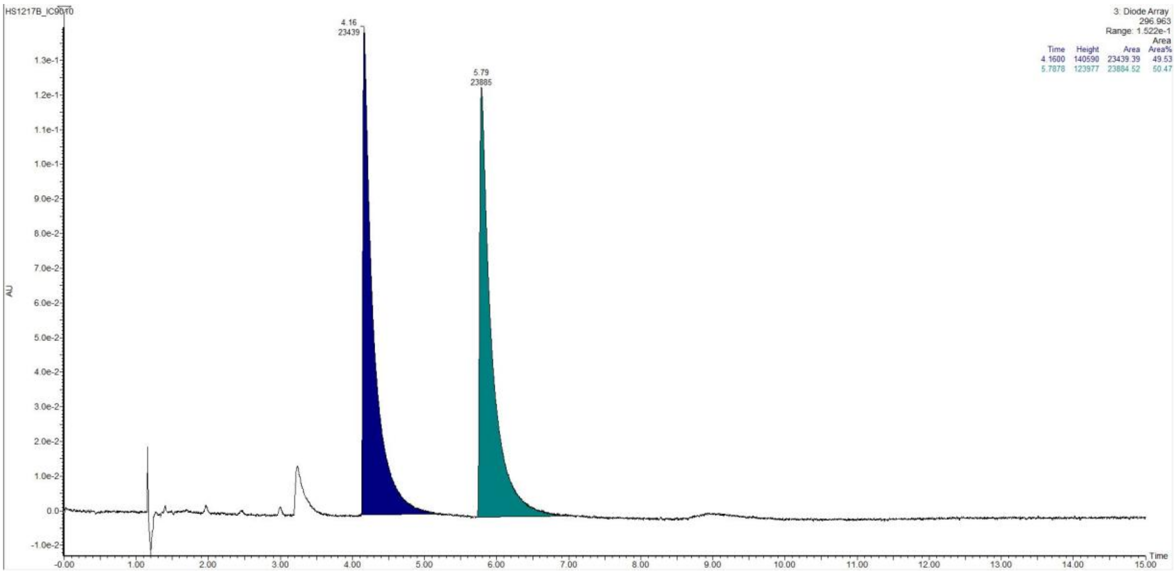

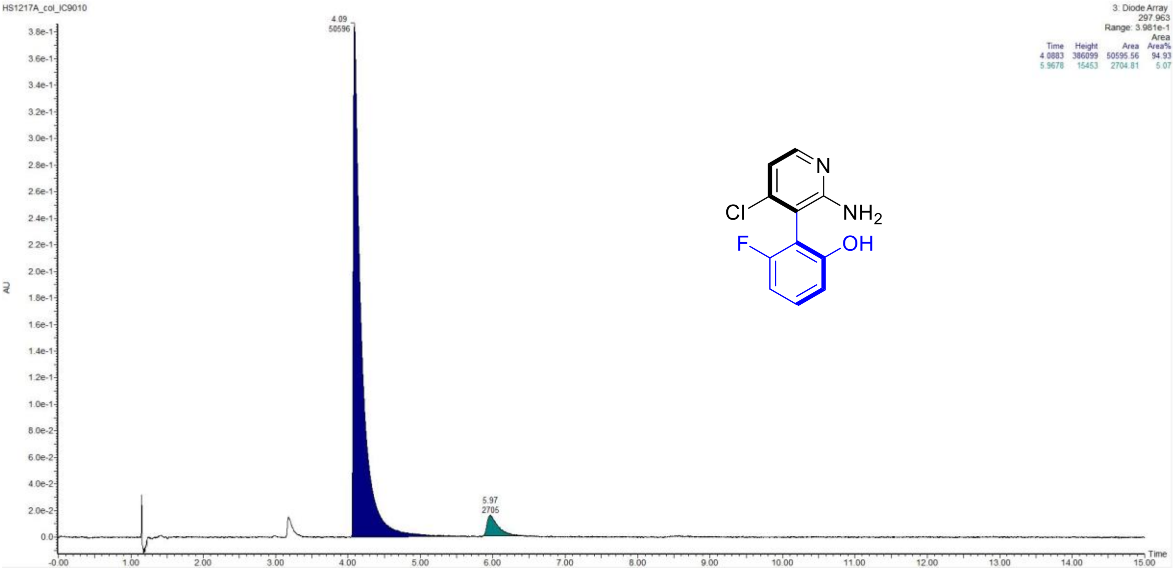

(S)-6,6'-dichloro-[1,1'-biphenyl]-2,2'-diol (**4a**)

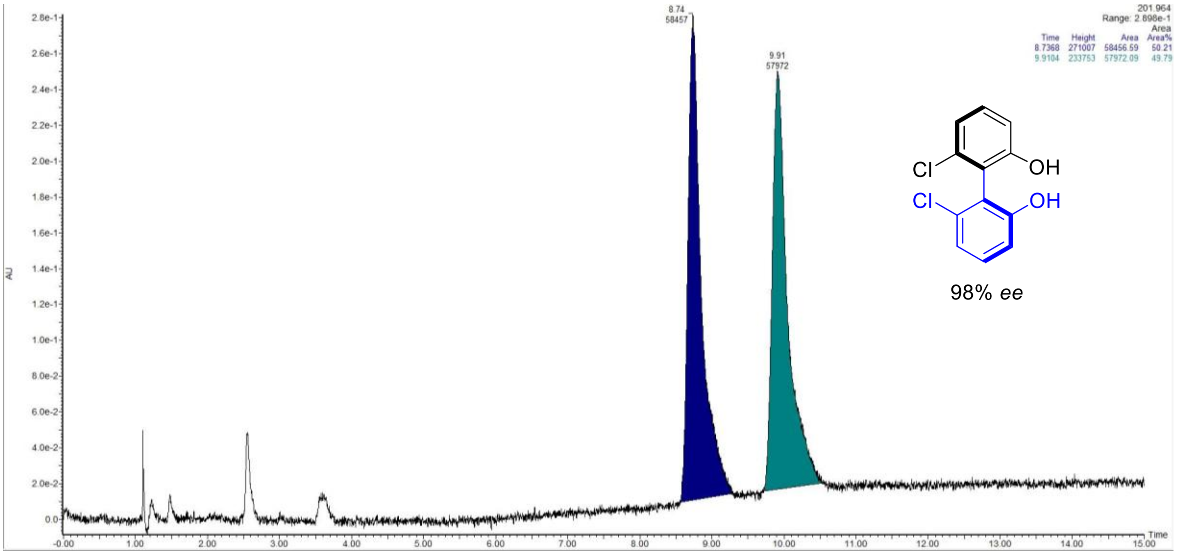

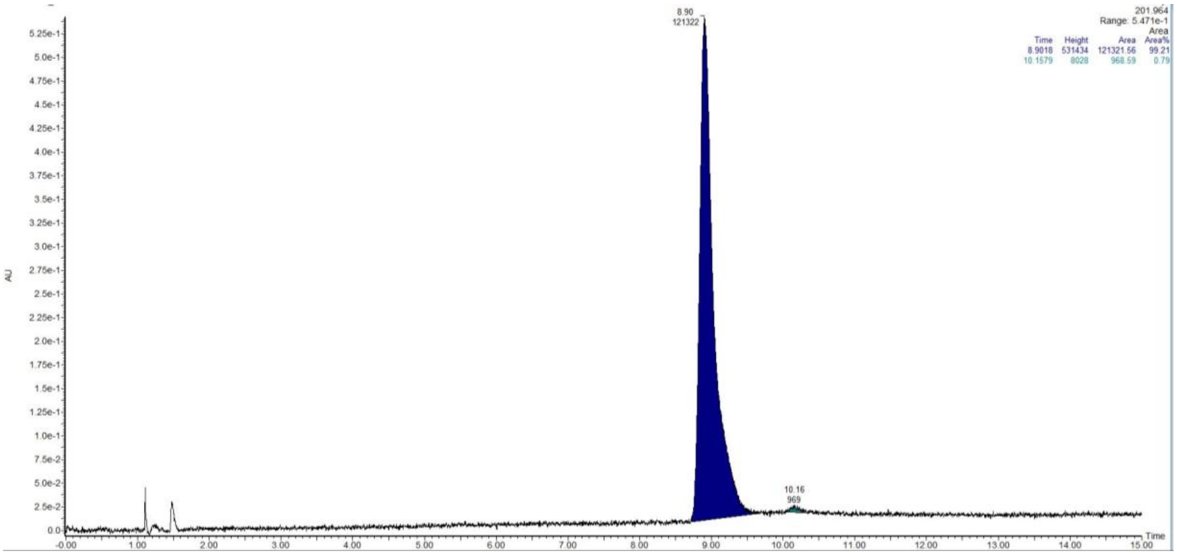

(S)-6,6'-dimethyl-[1,1'-biphenyl]-2,2'-diol (**4b**)

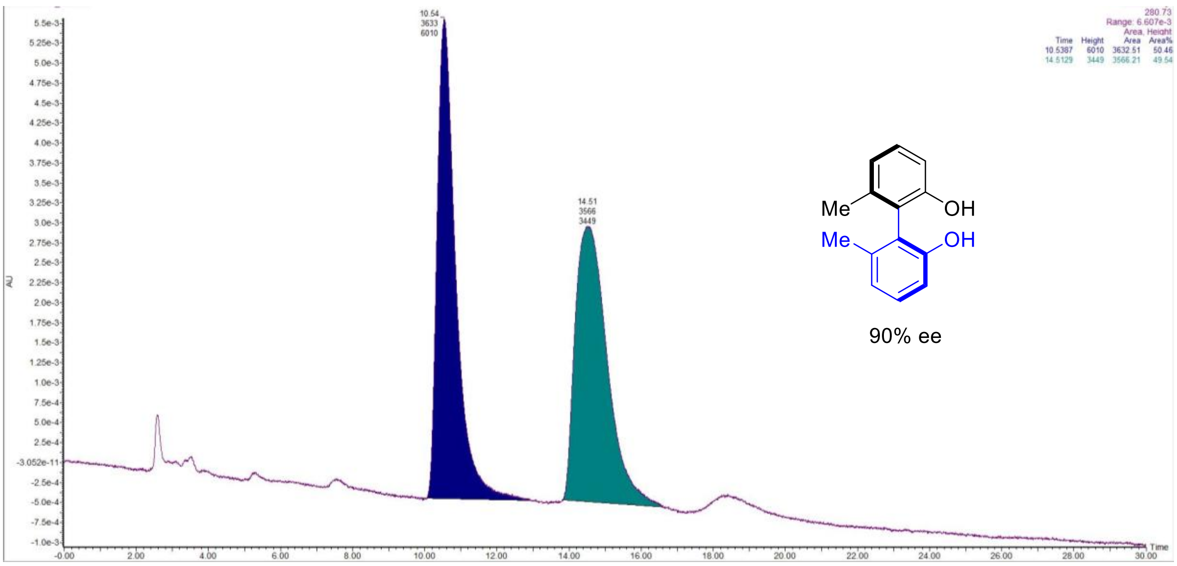

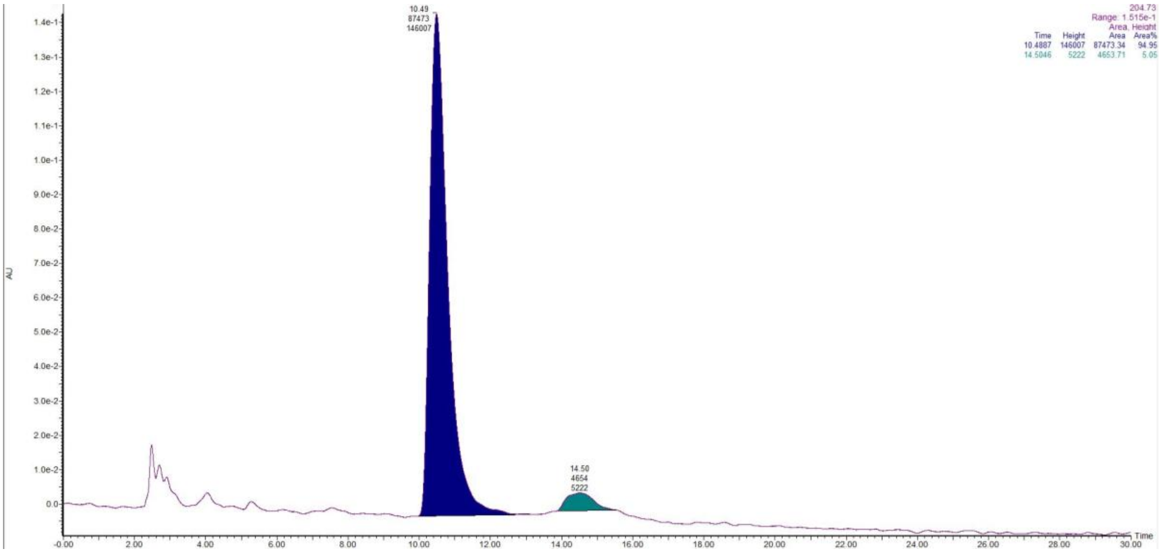

(R)-1-(2,3-difluoro-6-hydroxyphenyl)-5,6,7,8-tetrahydronaphthalen-2-ol (**4c)**

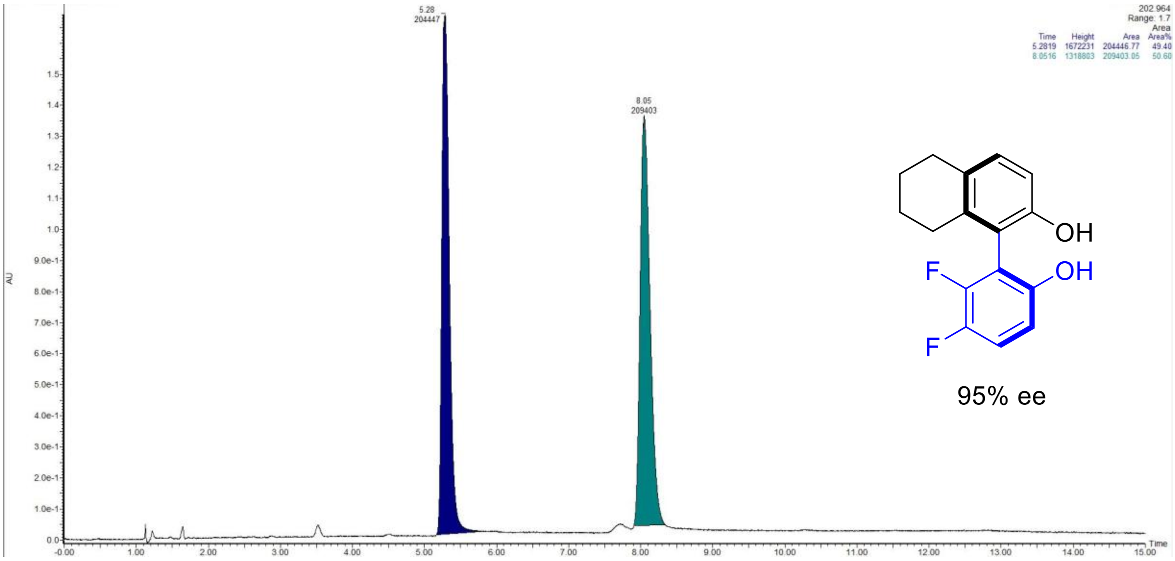

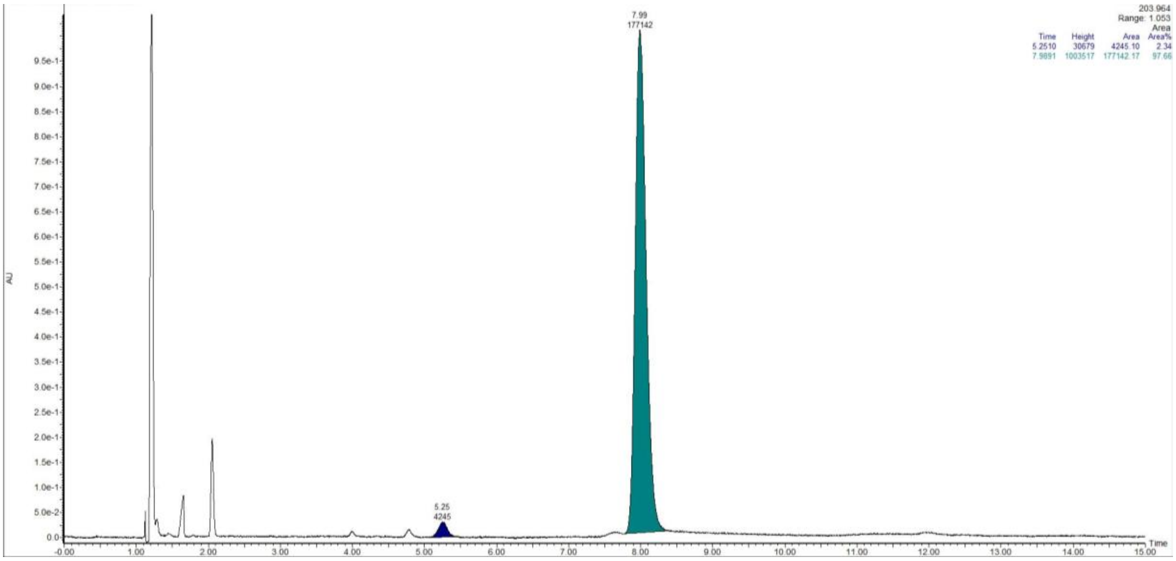

(R)-5,6'-dichloro-6-methyl-[1,1'-biphenyl]-2,2'-diol **(4d)**

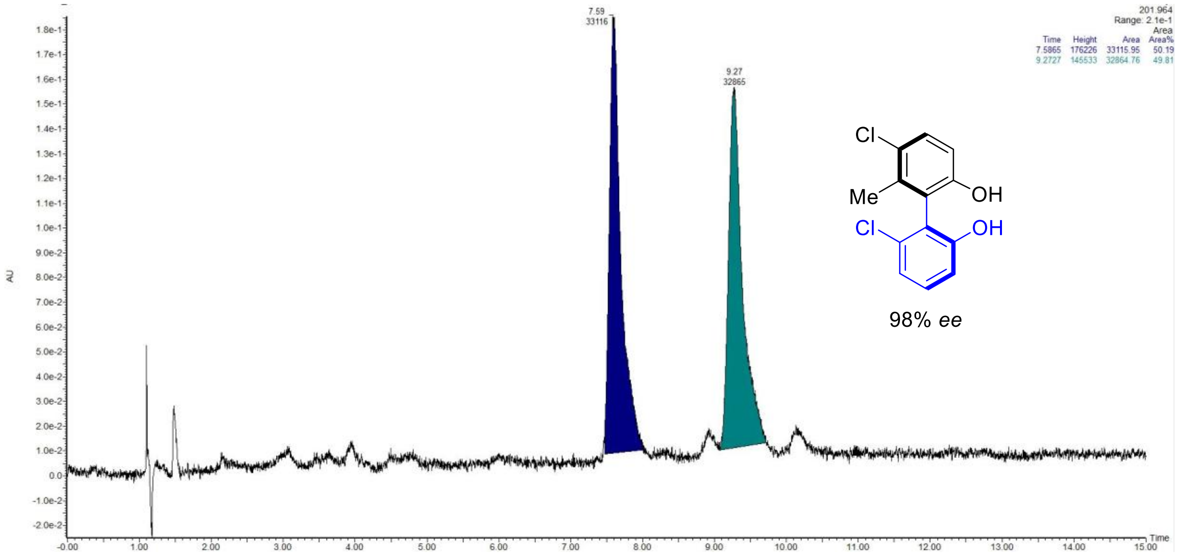

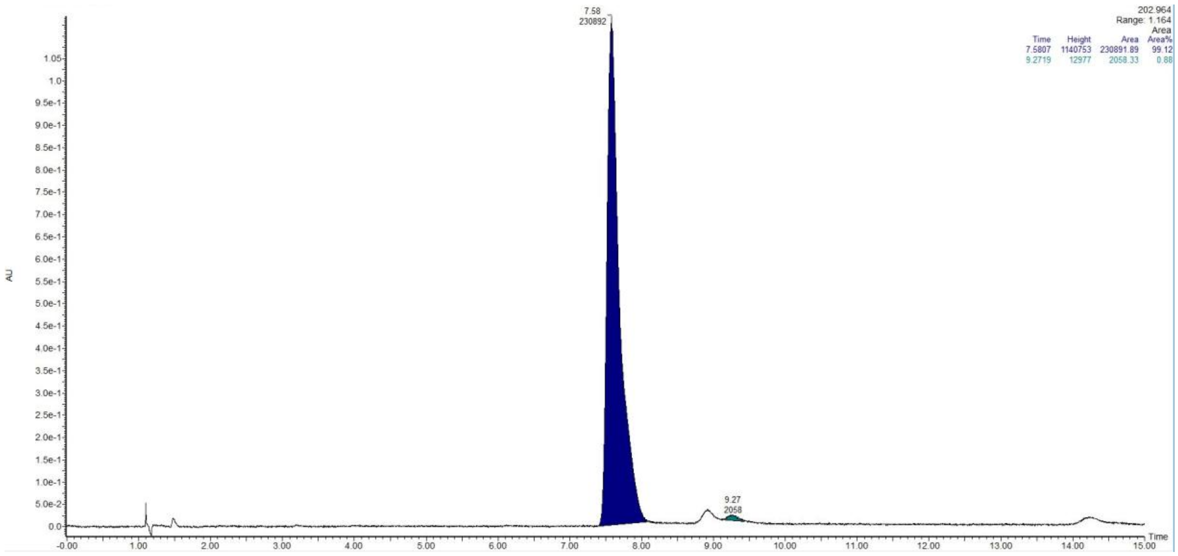

6-fluoro-2'-methoxy-6'-methyl-[1,1'-biphenyl]-2-ol **(4e)**

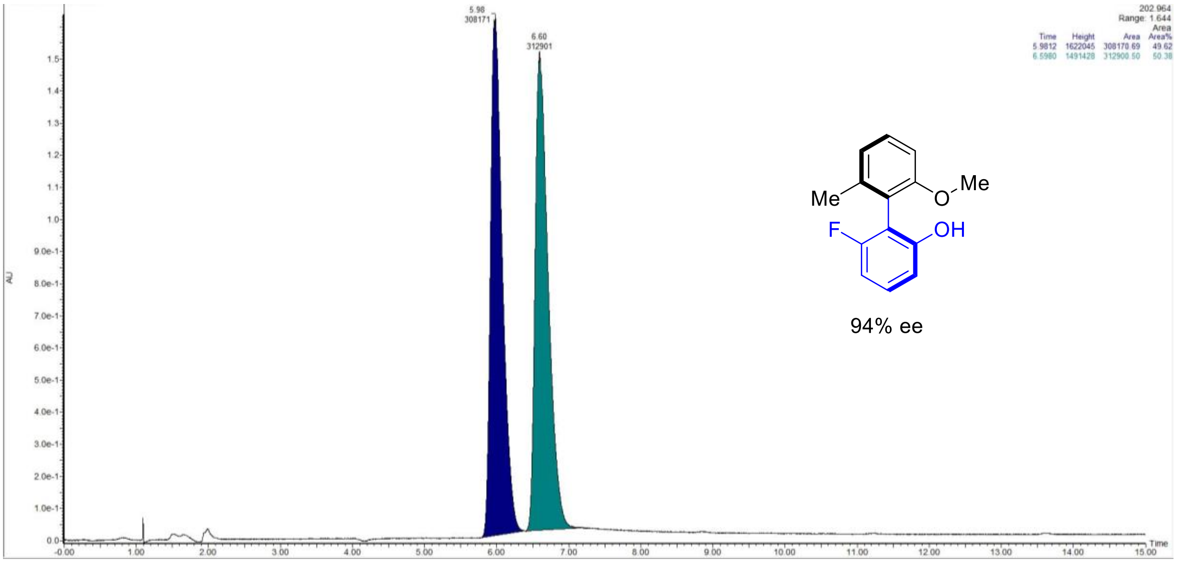

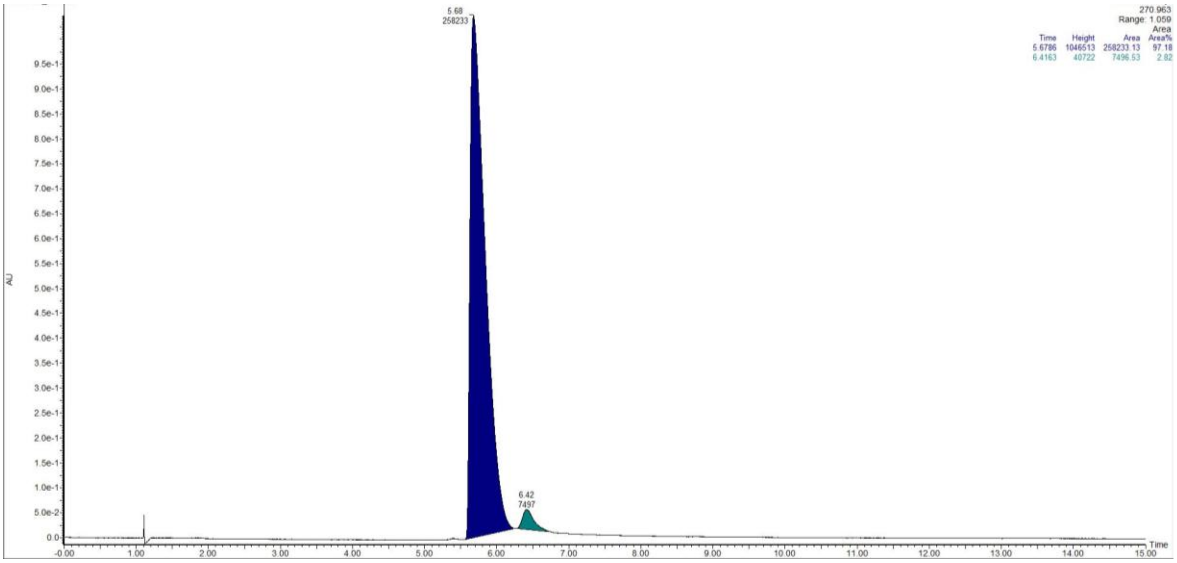

(S)-2'-chloro-6-fluoro-6'-methoxy-[1,1'-biphenyl]-2-ol (**4f**)

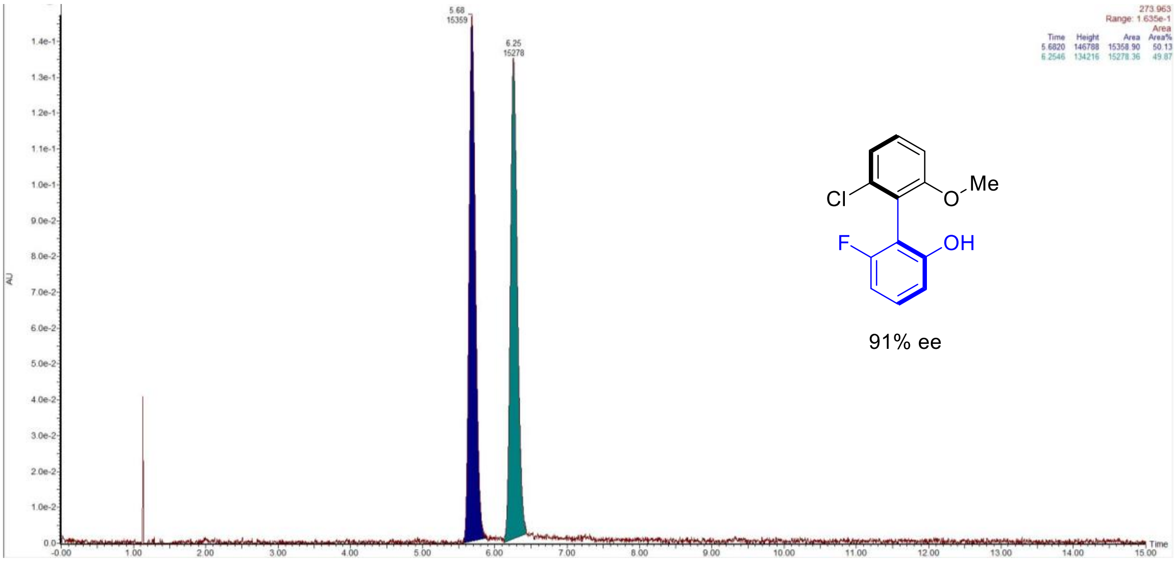

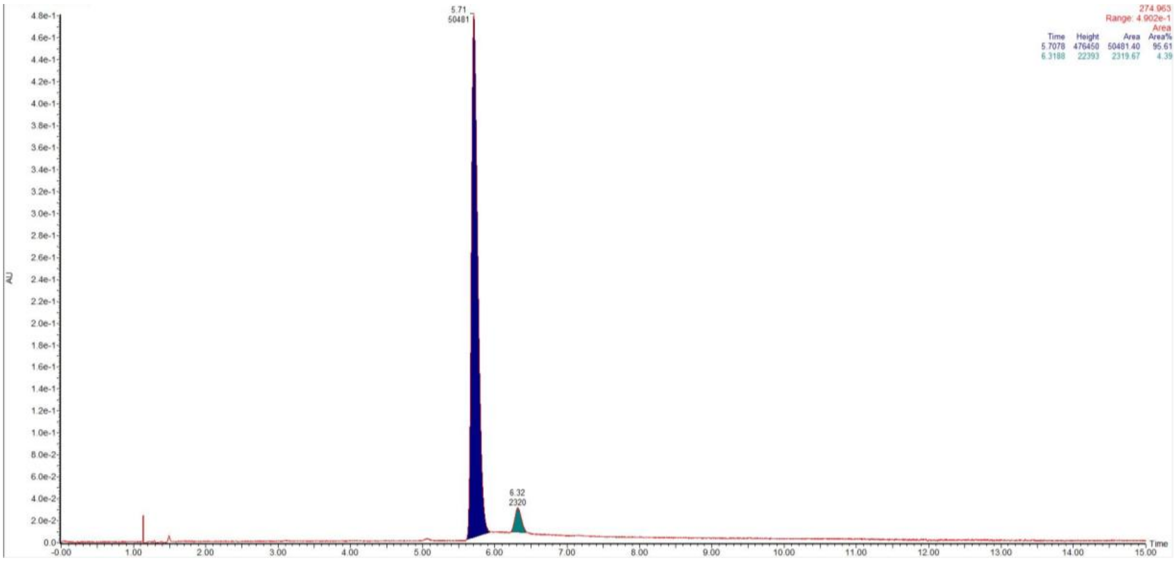

(R)-3-fluoro-2-(2-methoxy-5,6,7,8-tetrahydronaphthalen-1-yl)phenol (**4g)**

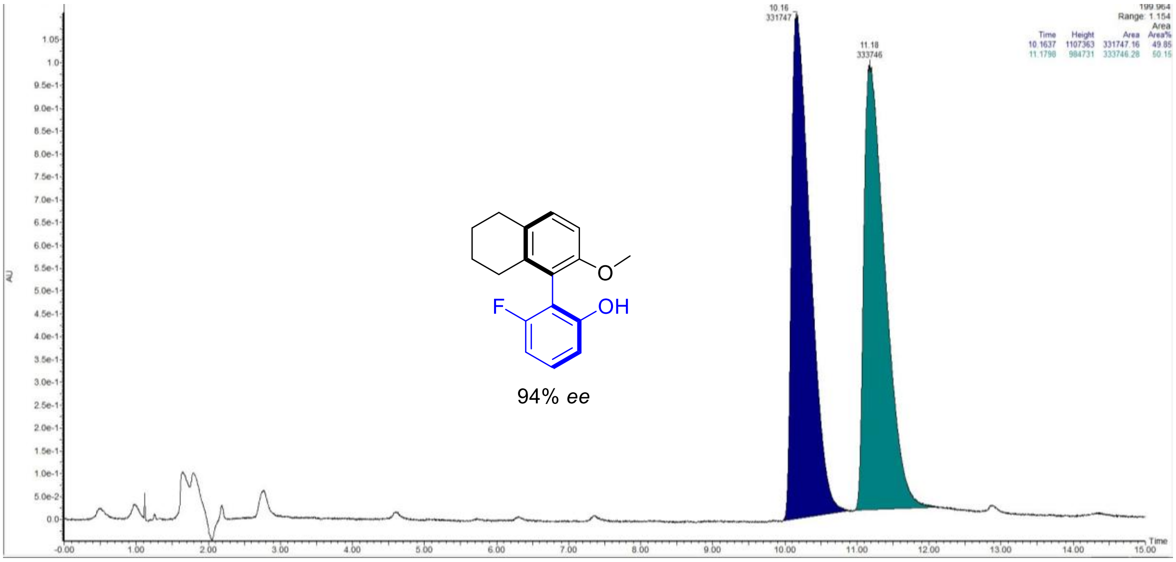

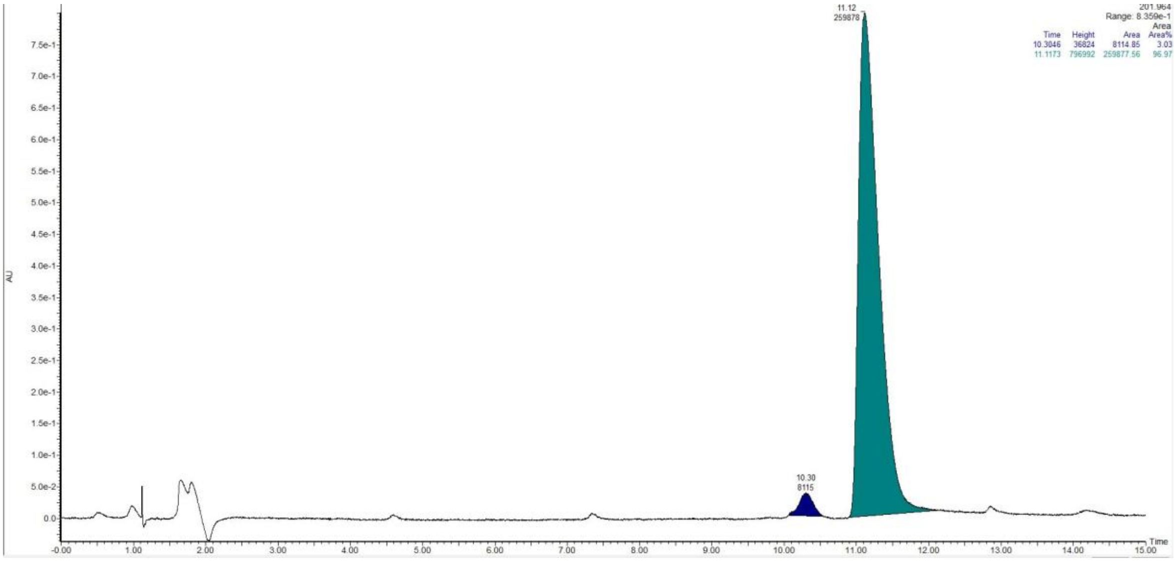

(R)-5,6-difluoro-2'-methoxy-6'-methyl-[1,1'-biphenyl]-2-ol (**4h**)

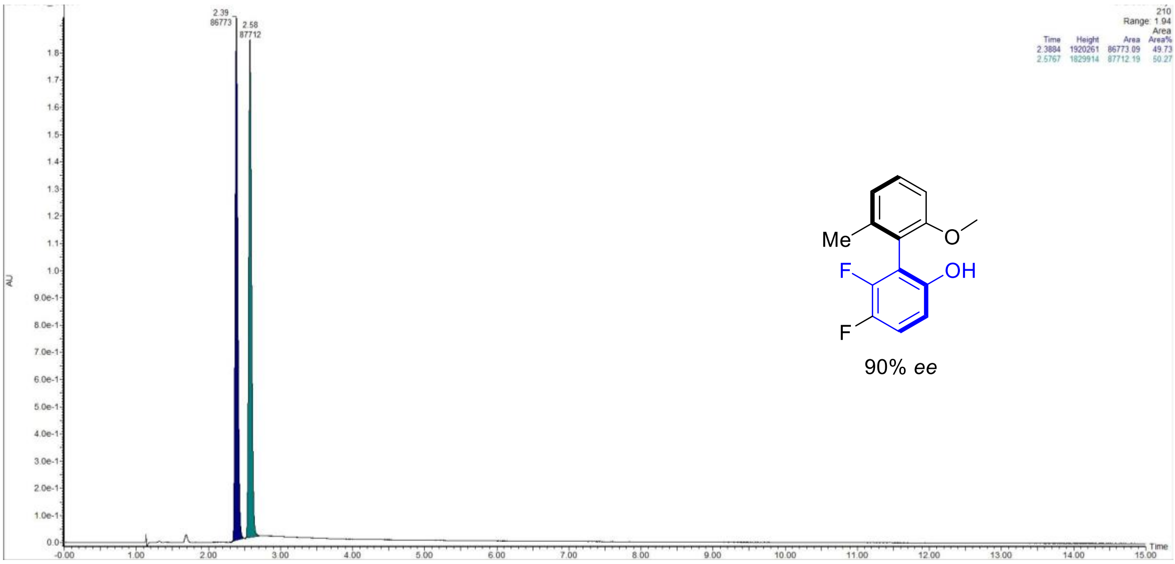

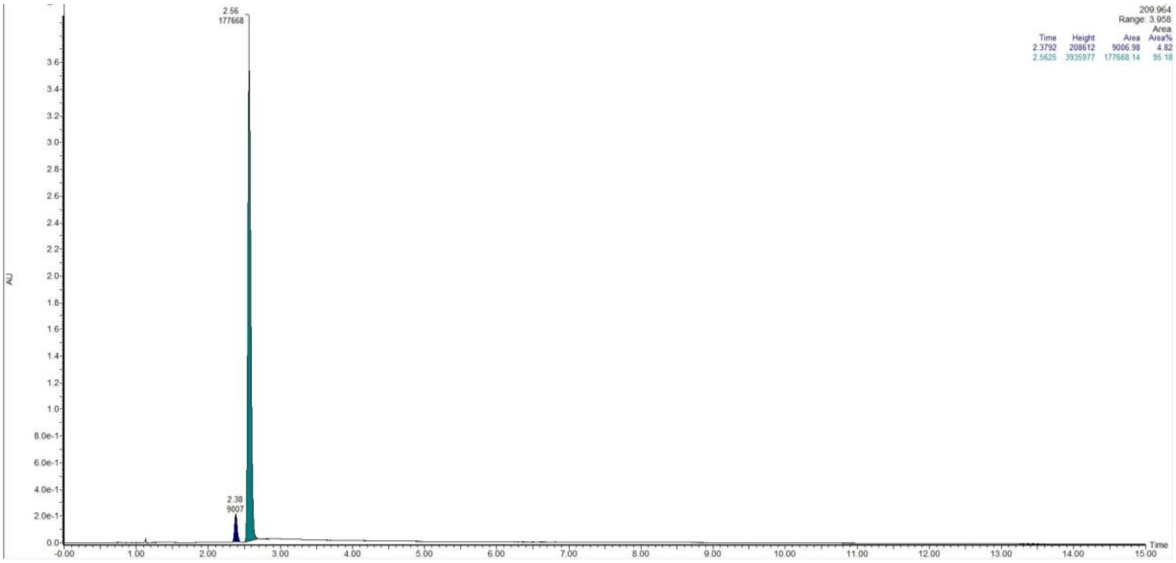

(R)-6-fluoro-6'-methyl-[1,1'-biphenyl]-2,2'-diol (**4i**)

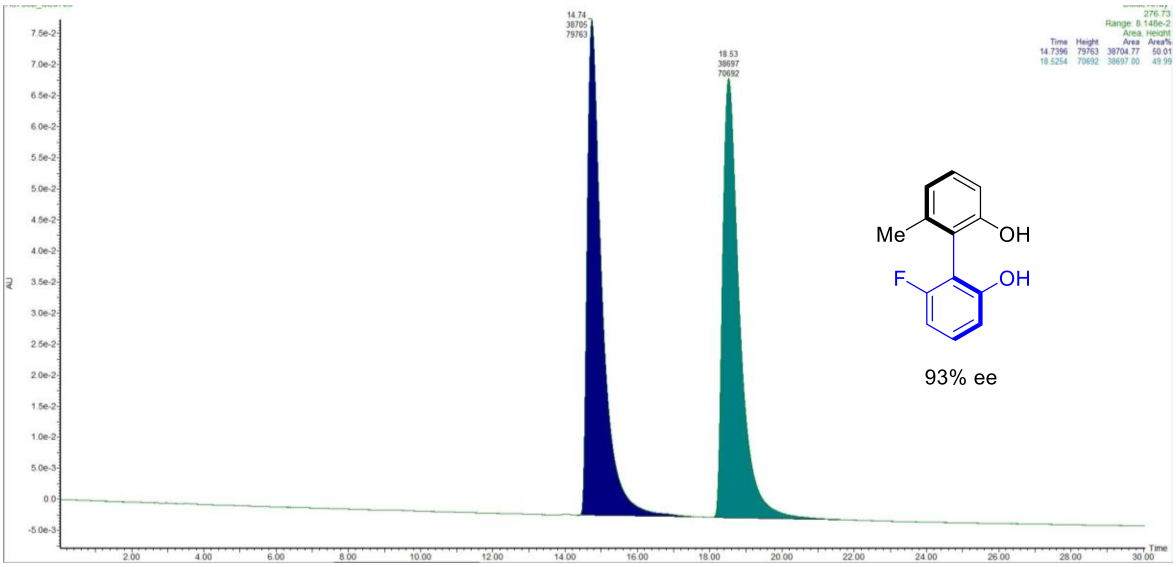

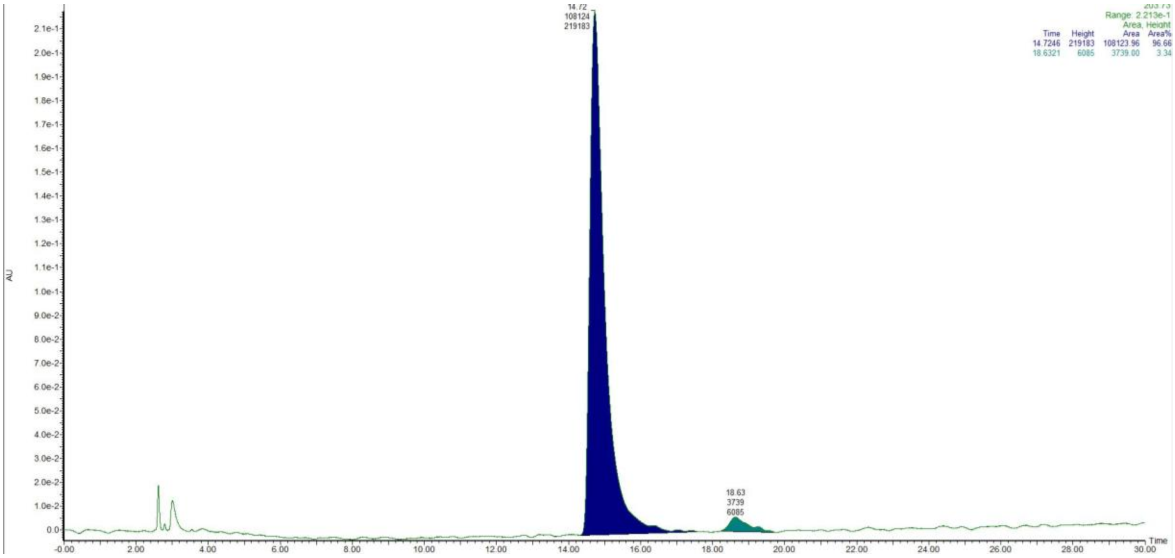

(R)-2'-fluoro-6'-methoxy-6-methyl-[1,1'-biphenyl]-2-ol (4j)

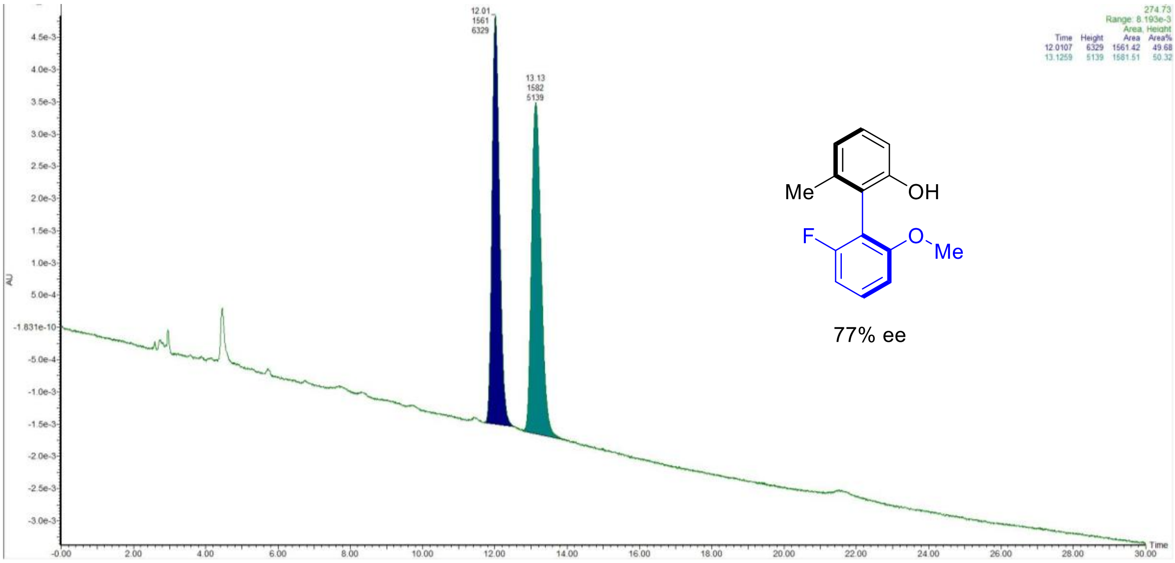

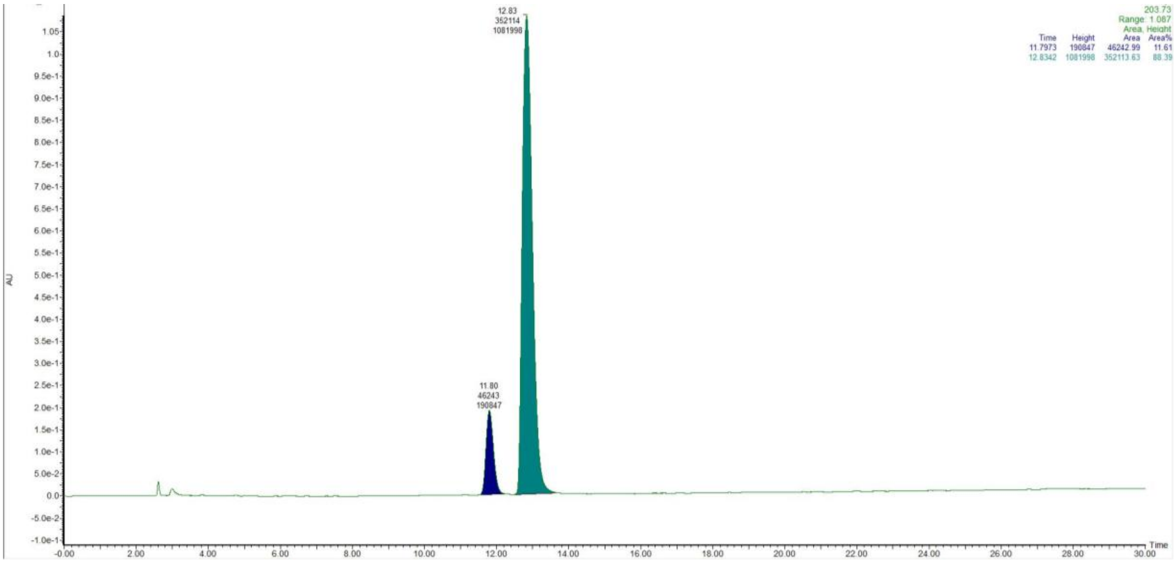

6-chloro-2'-(trifluoromethyl)-[1,1'-biphenyl]-2-ol (**5a**)

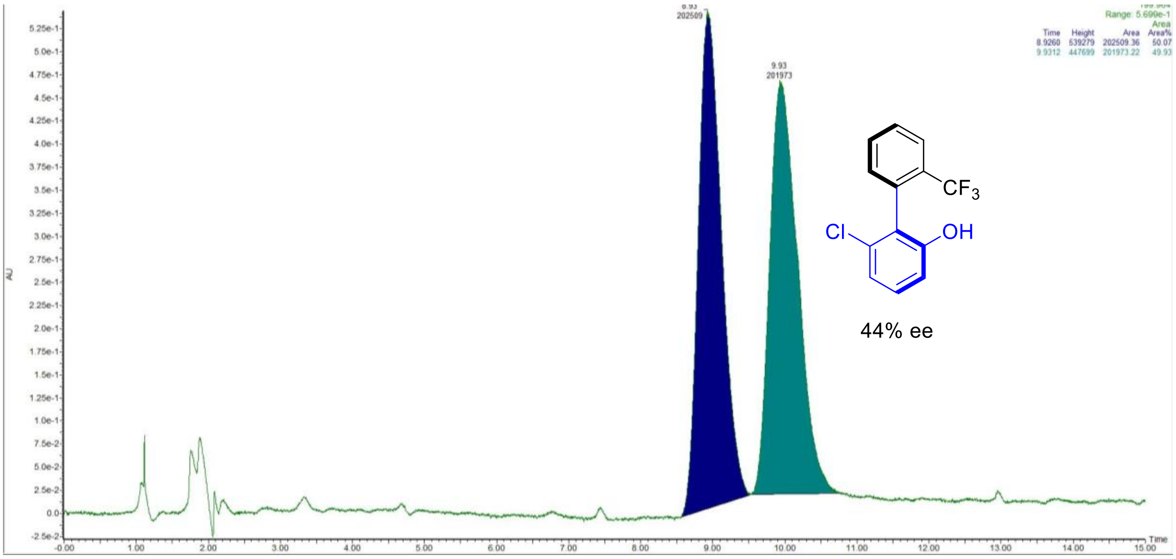

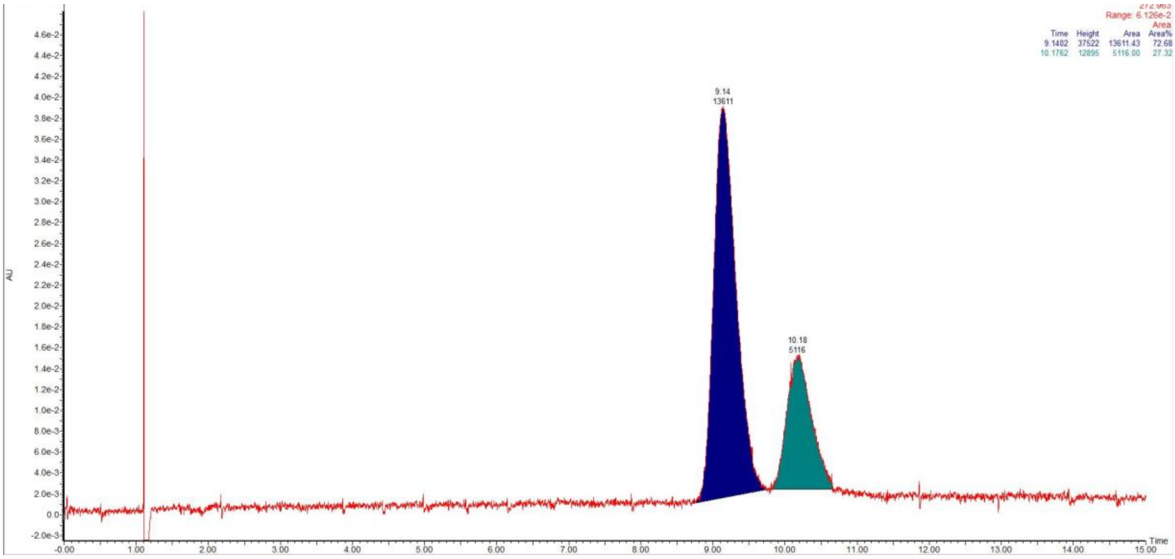

(*S*)-2'-chloro-6-fluoro-6'-methyl-[1,1'-biphenyl]-2-ol (**5b**)

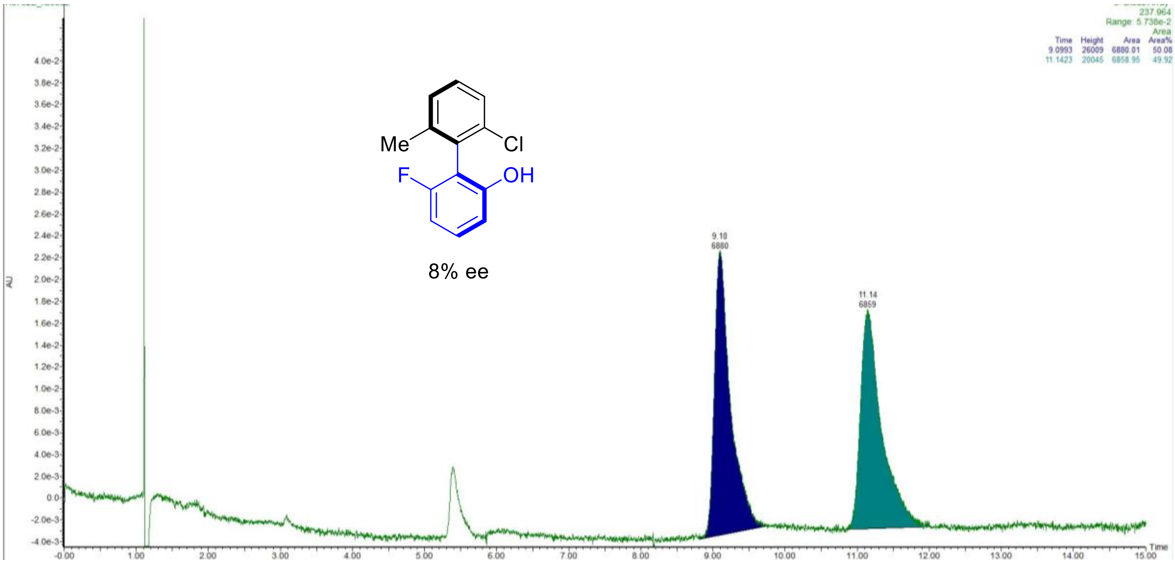

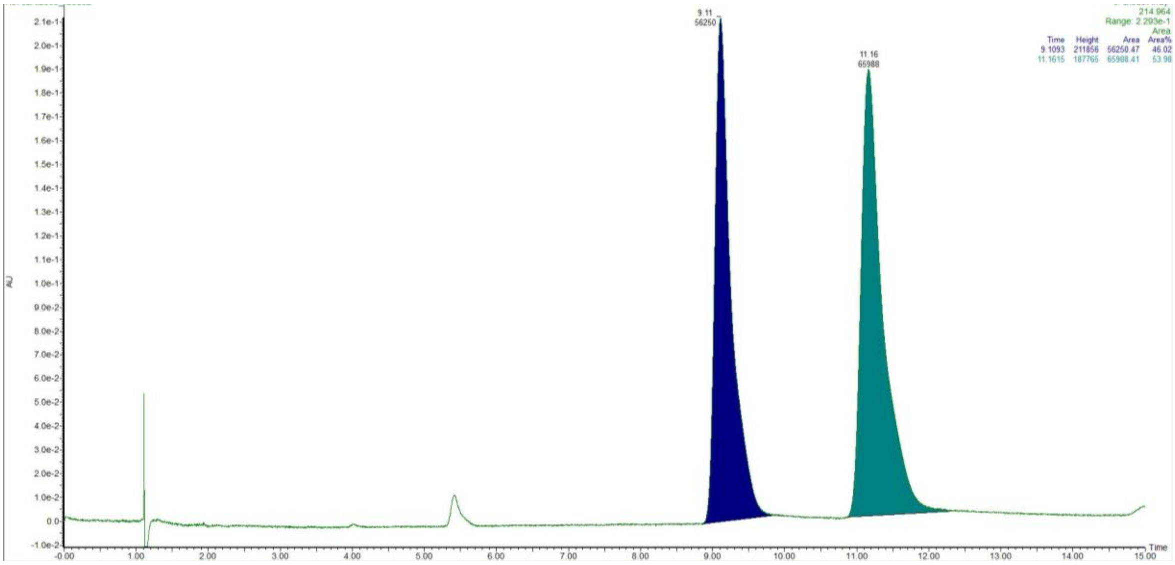

(S)-2'-amino-6-fluoro-6'-methoxy-[1,1'-biphenyl]-2-ol (**5c**)

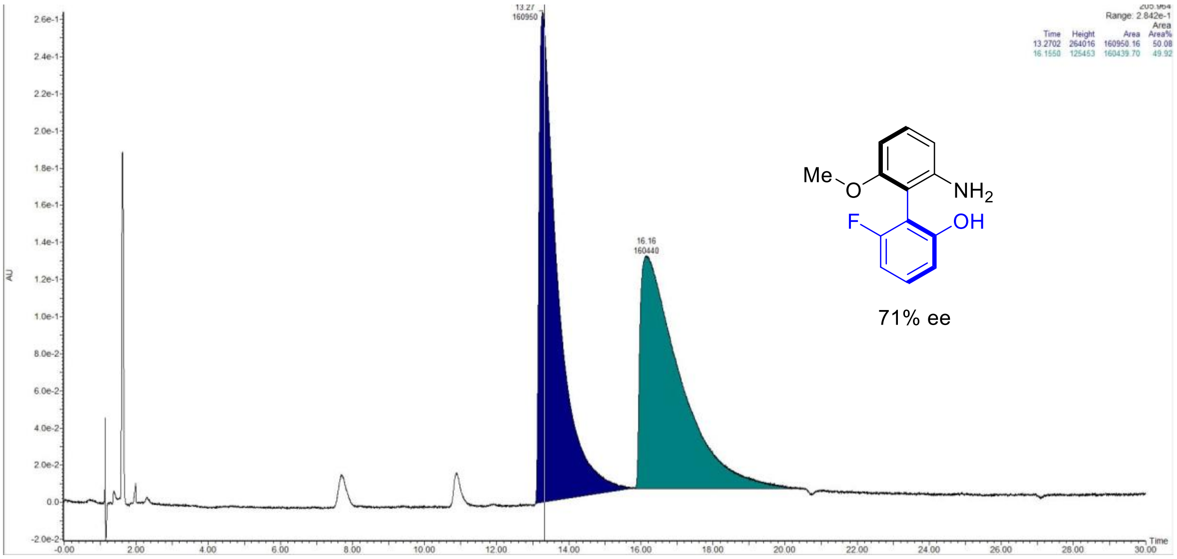

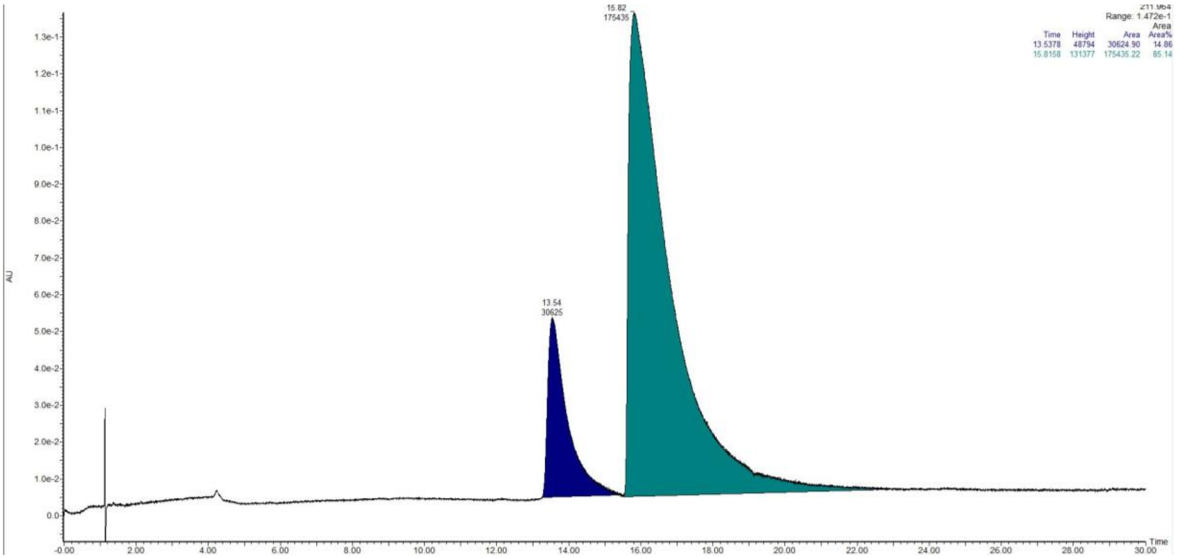

(R)-6-amino-6'-fluoro-[1,1'-biphenyl]-2,2'-diol (**5d**)

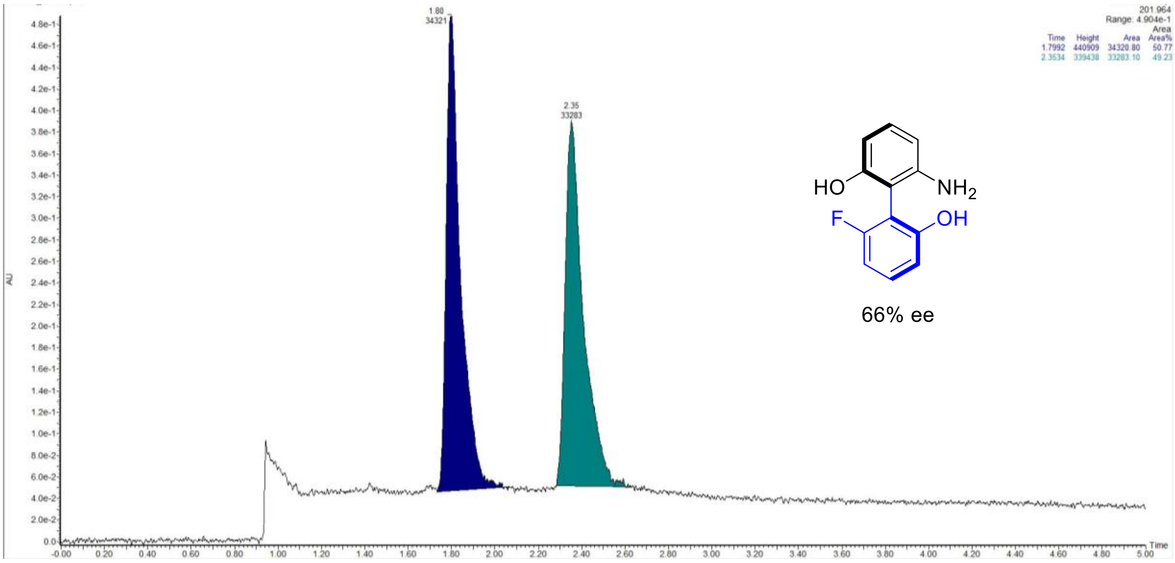

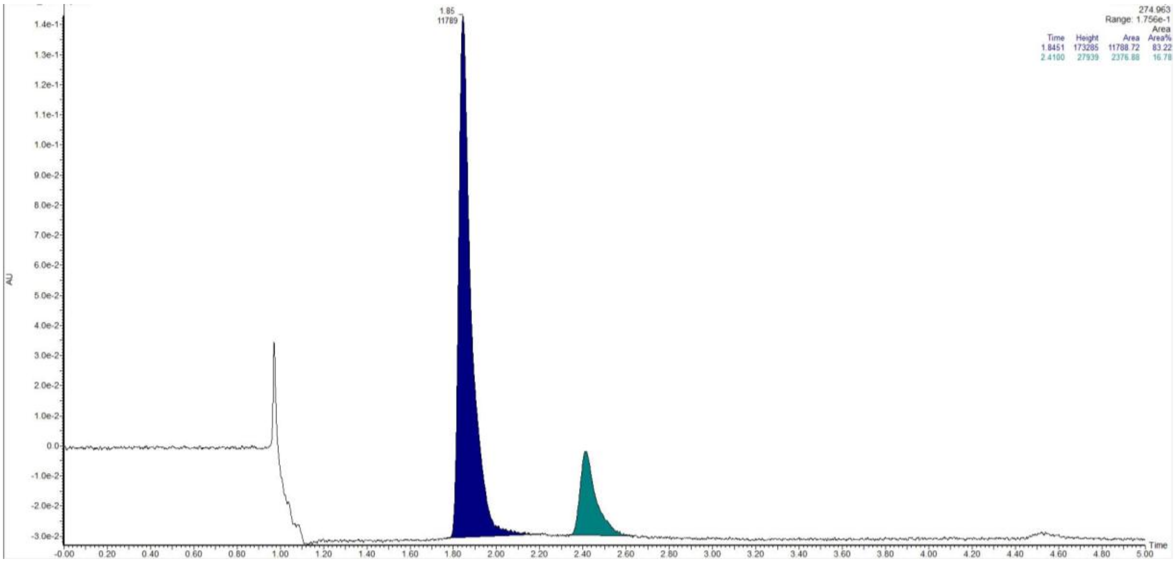

(R)-*N*-(2'-fluoro-6'-hydroxy-6-methyl-[1,1'-biphenyl]-2-yl)acetamide (**5f**)

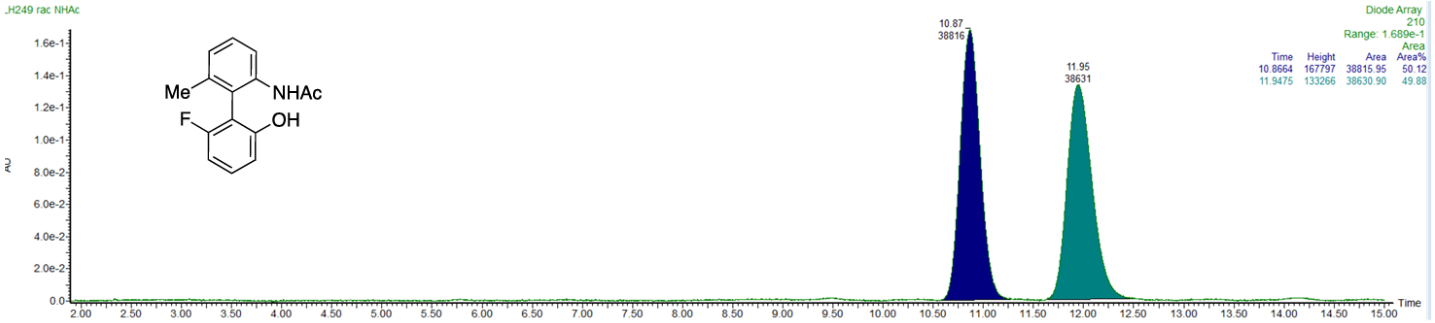

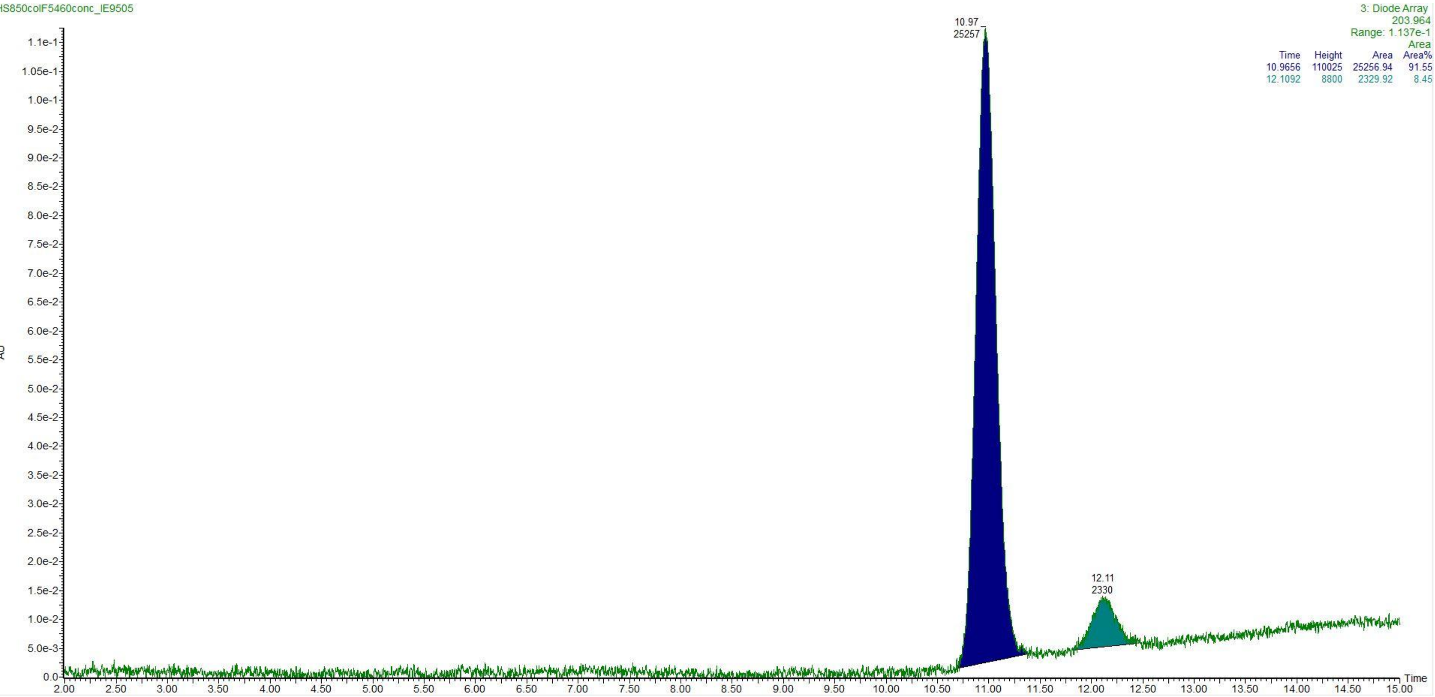

(R)-2,2,2-trifluoro-N-(2'-fluoro-6'-hydroxy-6-methyl-[1,1'-biphenyl]-2-yl)acetamide (**5g**)

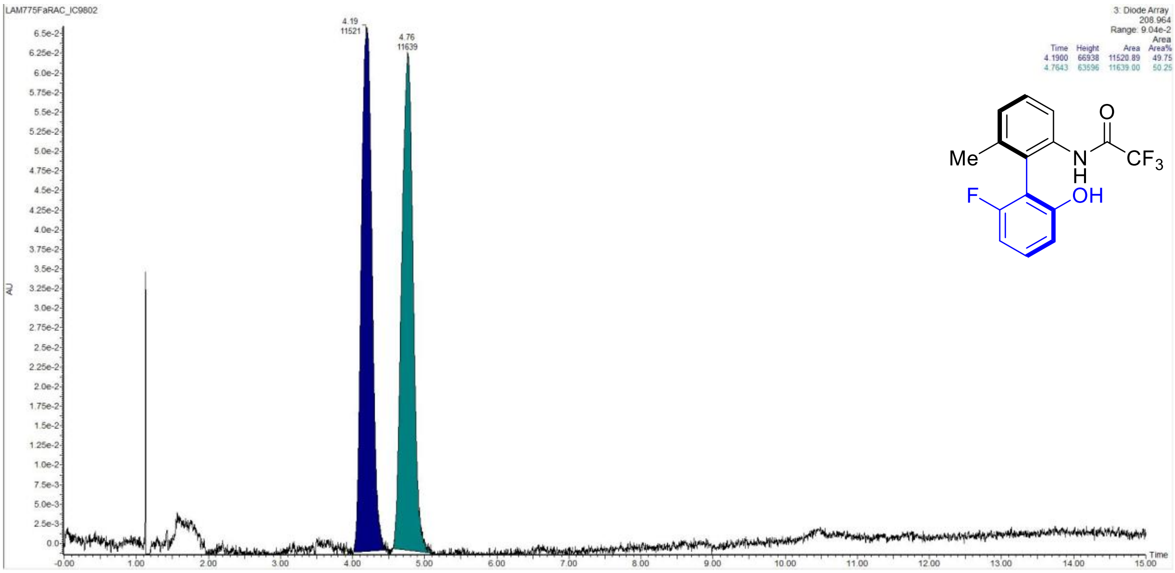

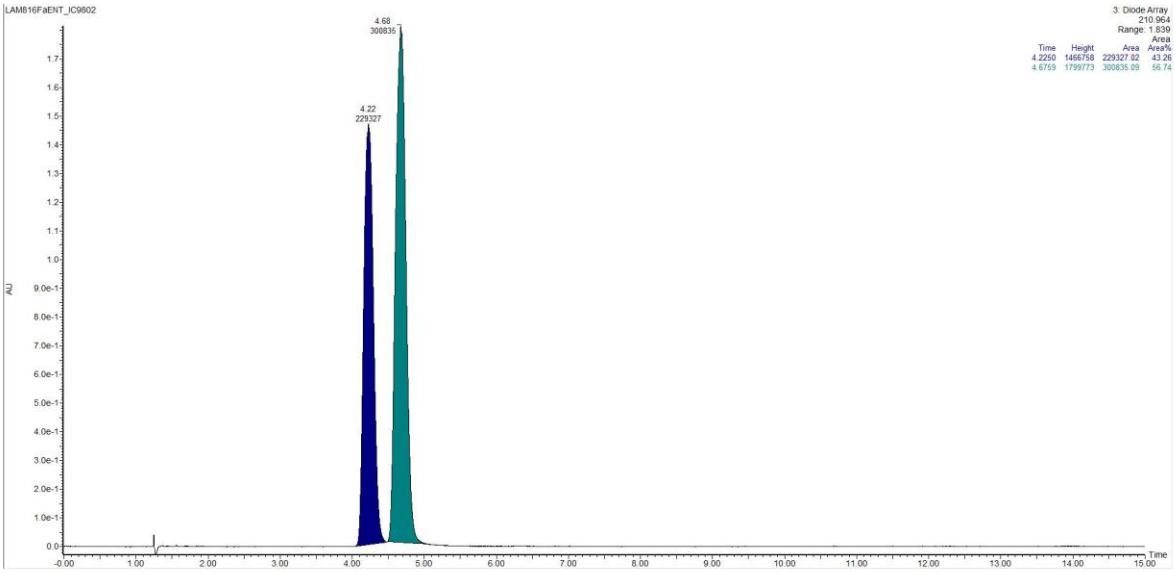

(R)-N-(2'-fluoro-6'-hydroxy-6-methyl-[1,1'-biphenyl]-2-yl)-4-methylbenzenesulfonamide (**5h**)

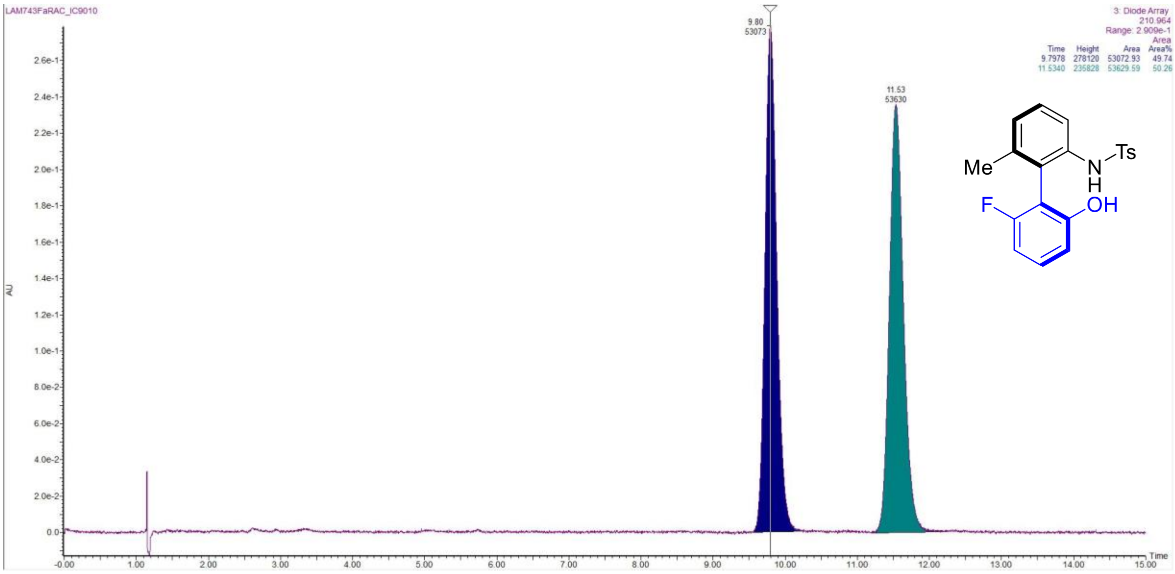

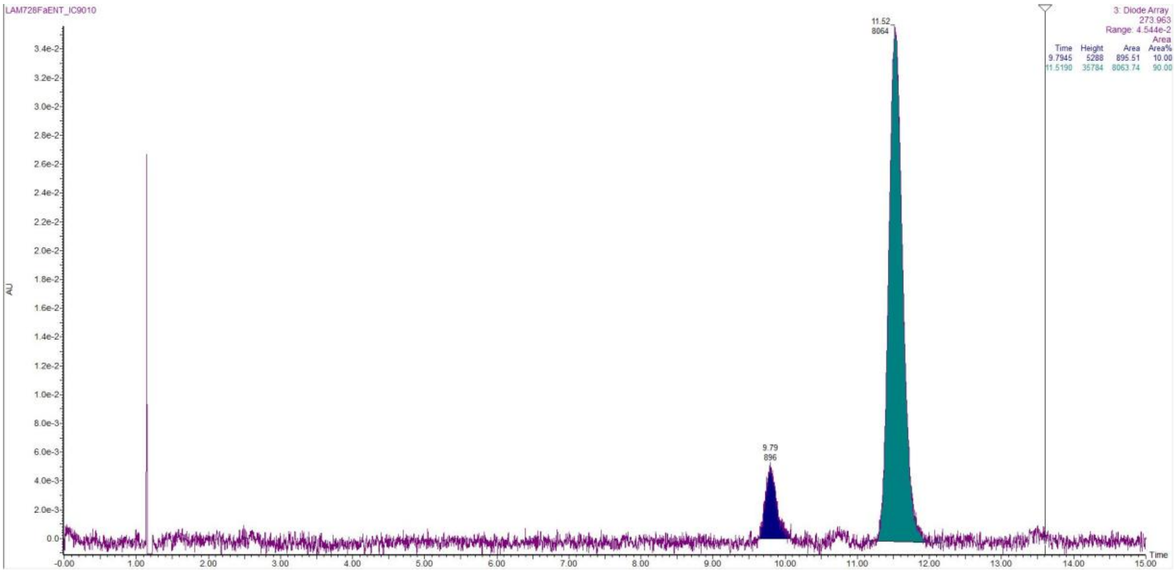

¹H-NMR (MeOD): Sodium-2'-(dicyclohexylphosphaneyl)-2,6-diisopropoxy-[1,1'-biphenyl]-3-sulfonate (rac)-sRuPhos

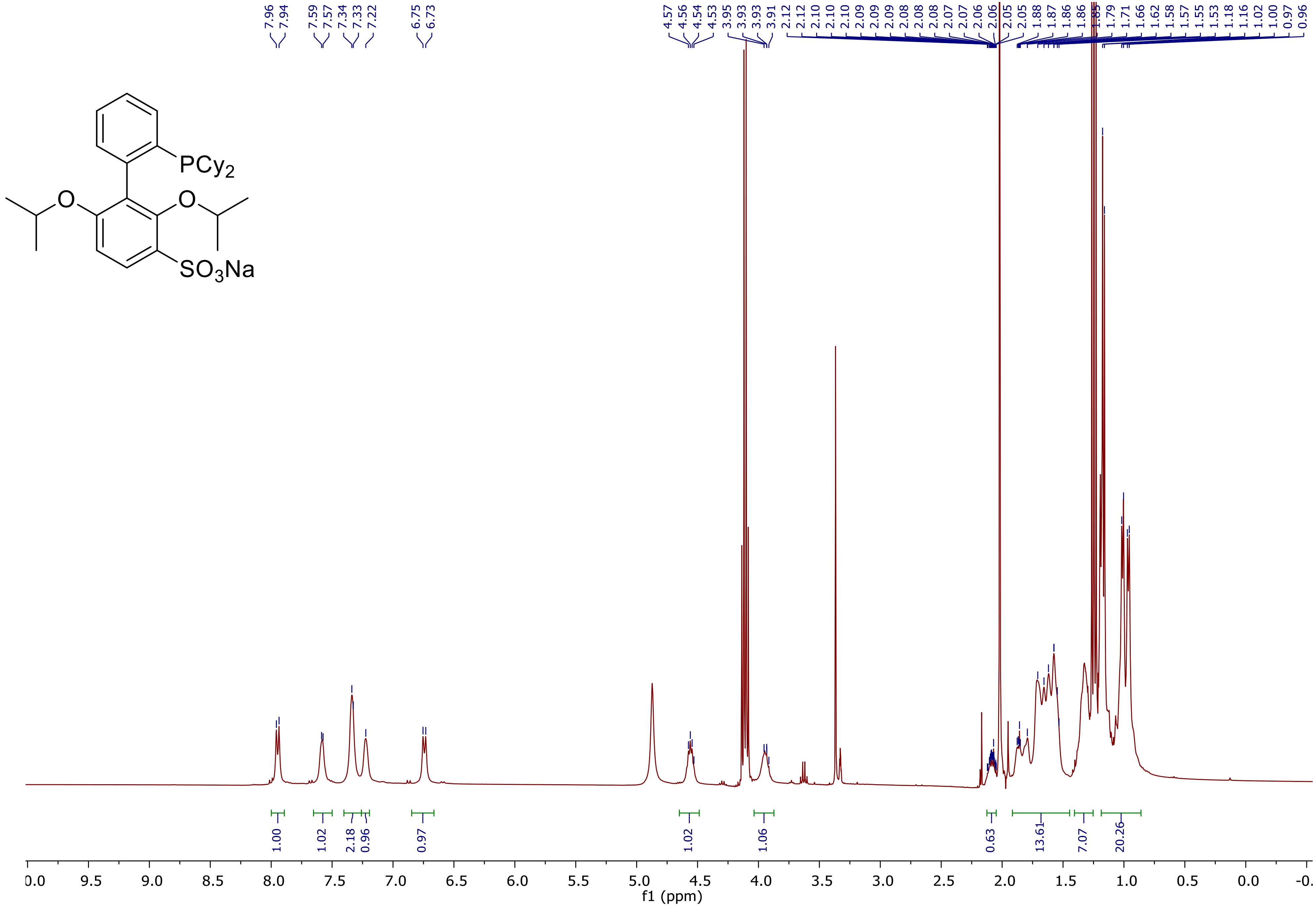

^{31}P-NMR (MeOD): Sodium-2'-(dicyclohexylphosphaneyl)-2,6-diisopropoxy-[1,1'-biphenyl]-3-sulfonate (rac)- sRuPhos

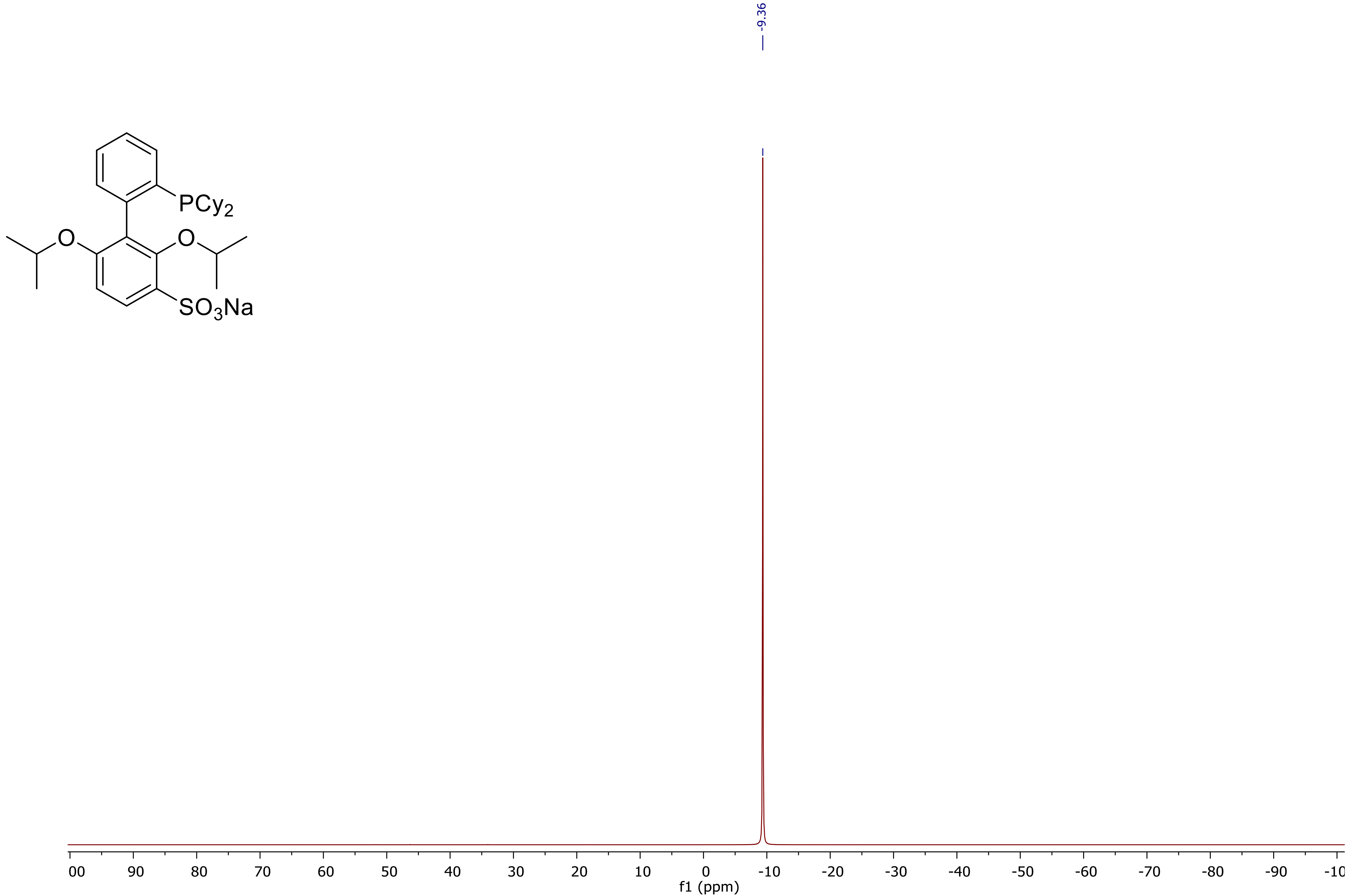

^{13}C-NMR (MeOD): Sodium-2'-(dicyclohexylphosphaneyl)-2,6-diisopropoxy-[1,1'-biphenyl]-3-sulfonate (rac)-sRuPhos

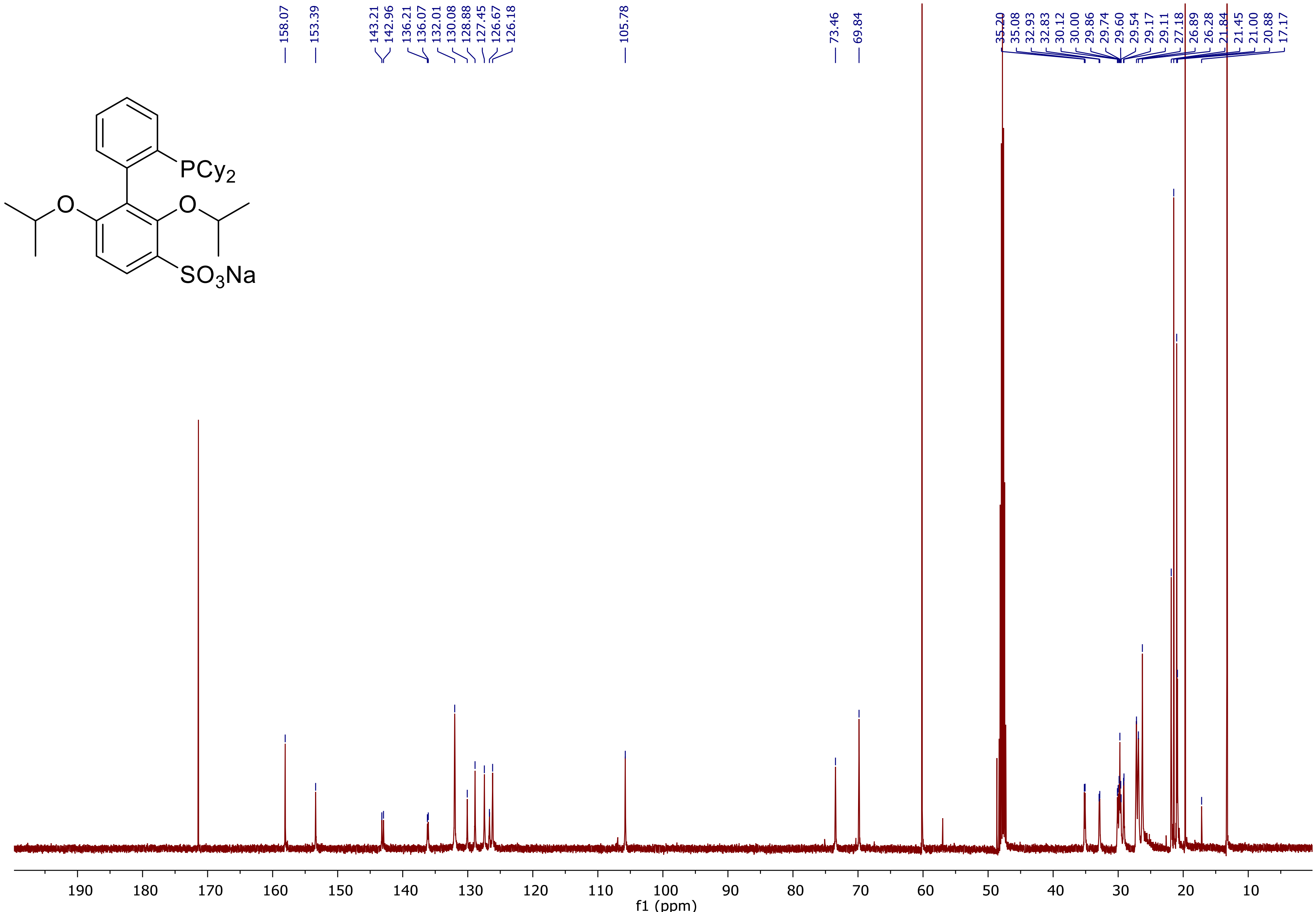

¹H-NMR (MeOD): Sodium (R)-2'-(dicyclohexylphosphaneyl)-2,6-diisopropoxy-5-methyl-[1,1'-biphenyl]-3-sulfonate (R)-Me-sRuPhos

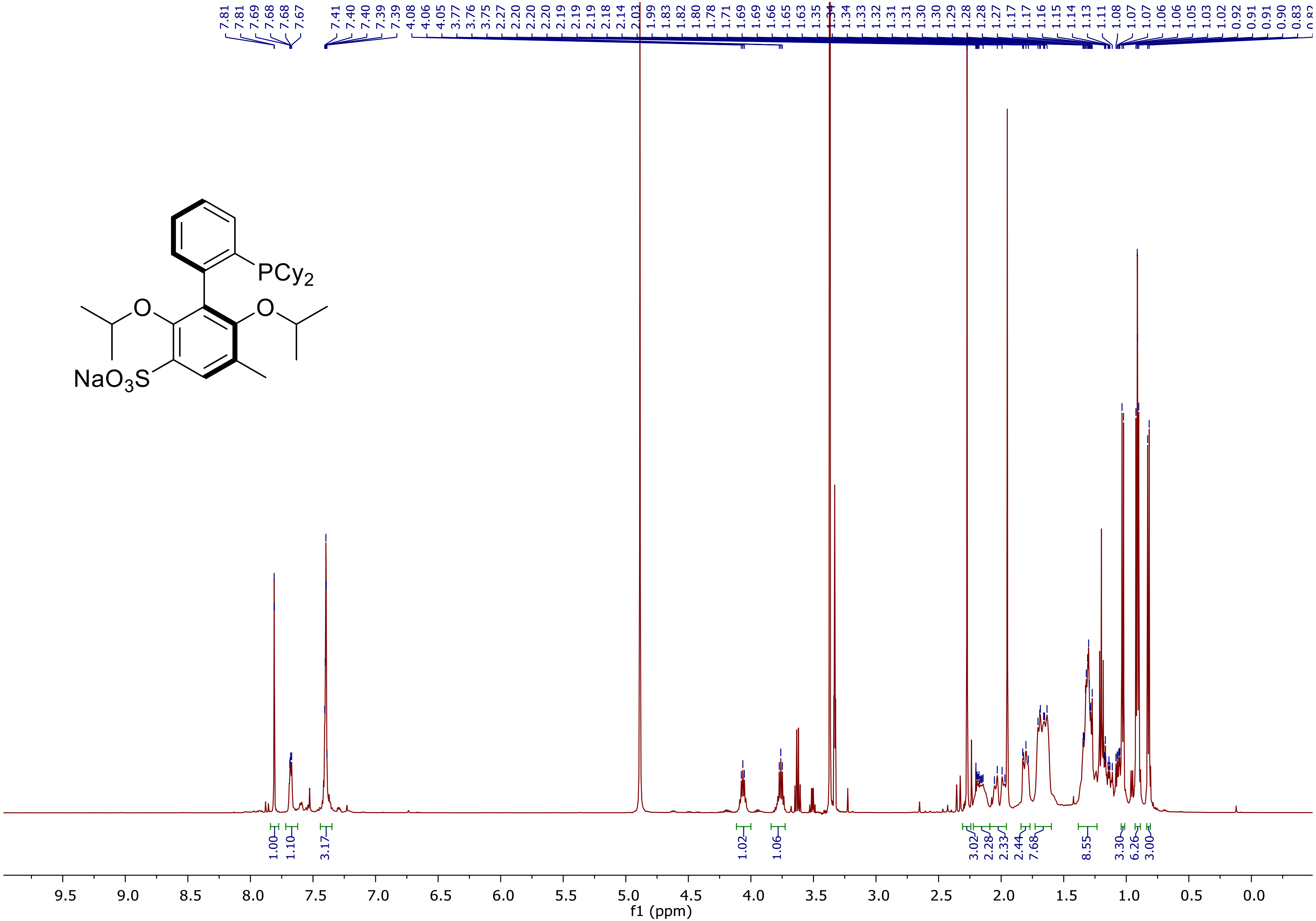

^{31}P-NMR (MeOD): Sodium (R)-2'-(dicyclohexylphosphaneyl)-2,6-diisopropoxy-5-methyl-[1,1'-biphenyl]-3-sulfonate (R-Me-sRuPhos

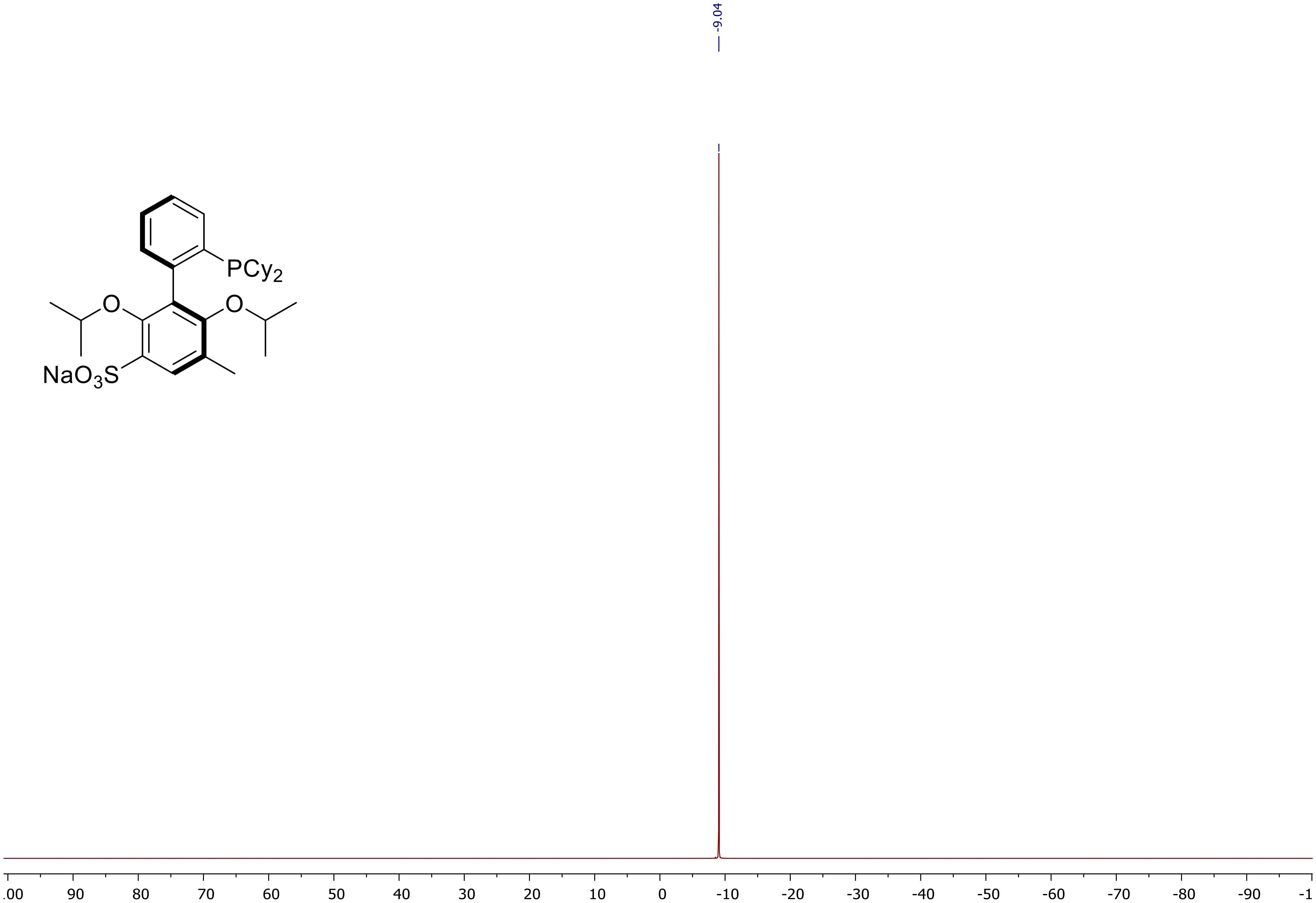

^{13}C-NMR (MeOD): Sodium (R)-2'-(dicyclohexylphosphaneyl)-2,6-diisopropoxy-5-methyl-[1,1'-biphenyl]-3-sulfonate (R)-Me-sRuPhos

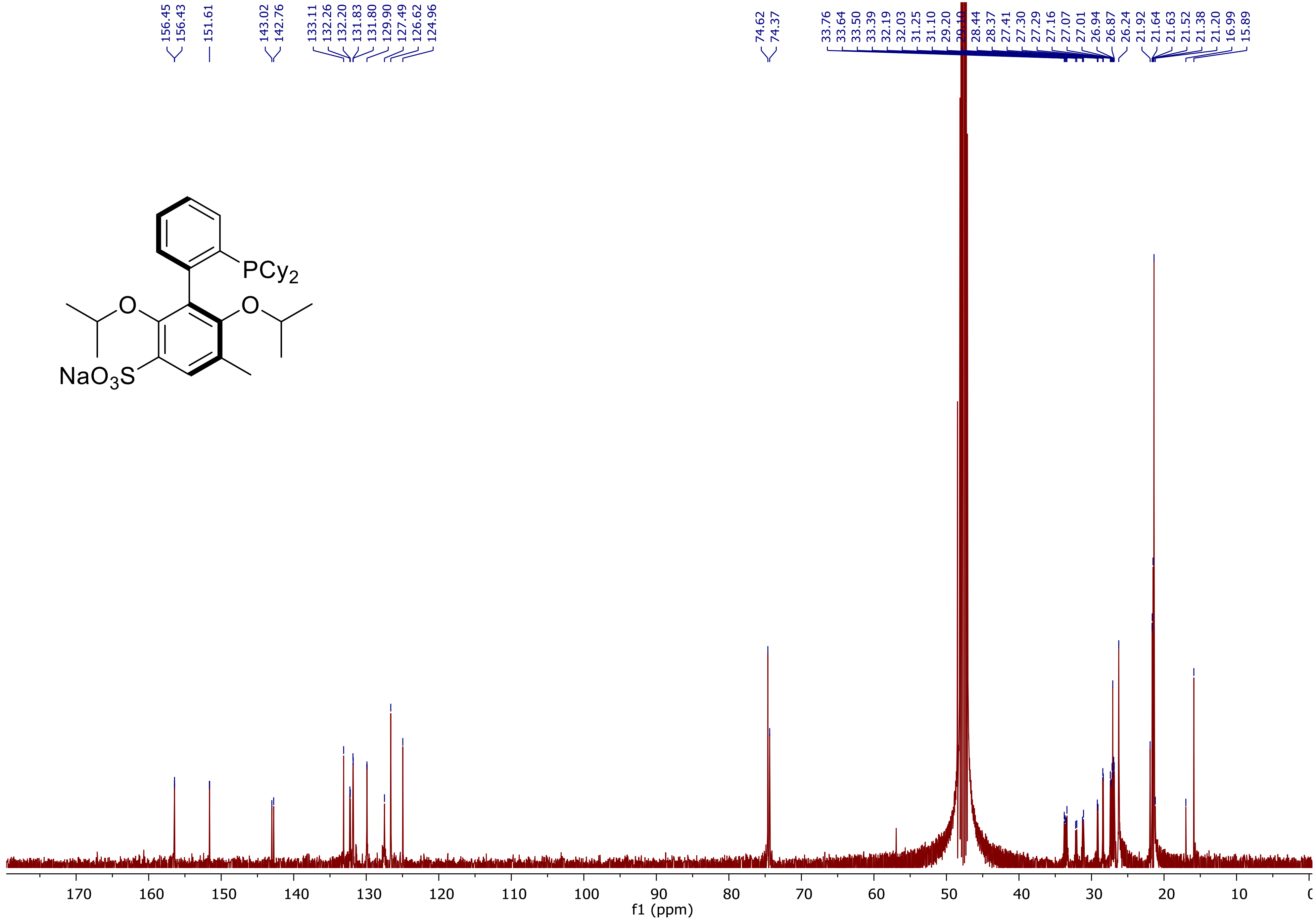

¹H-NMR (MeOD): Sodium-2'-(dicyclohexylphosphaneyl)-2,6-diisopropoxy-[1,1'-biphenyl]-3-sulfonate (R)-sRuPhos

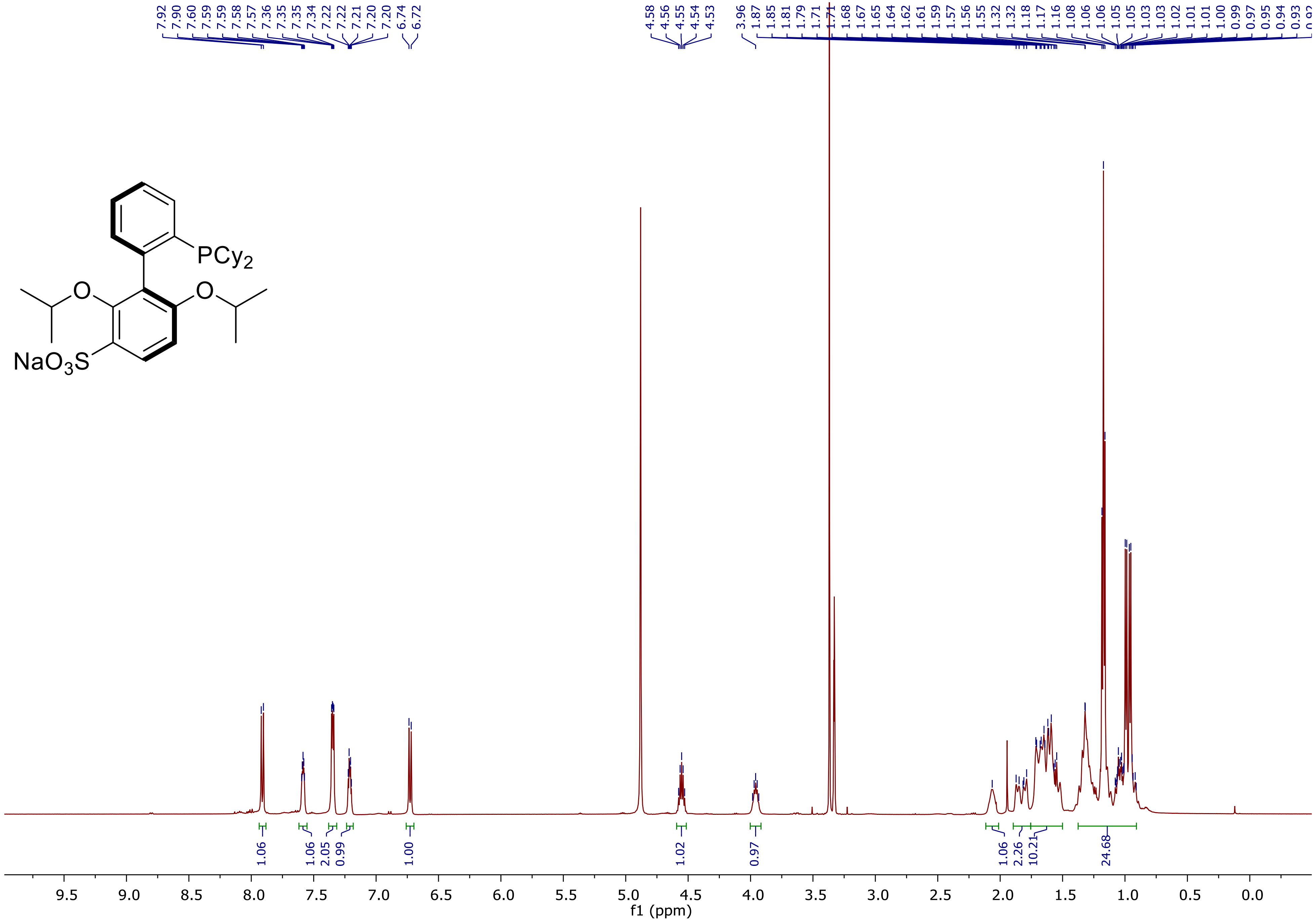

^{31}P-NMR (MeOD): Sodium-2'-(dicyclohexylphosphaneyl)-2,6-diisopropoxy-[1,1'-biphenyl]-3-sulfonate (R)- sRuPhos

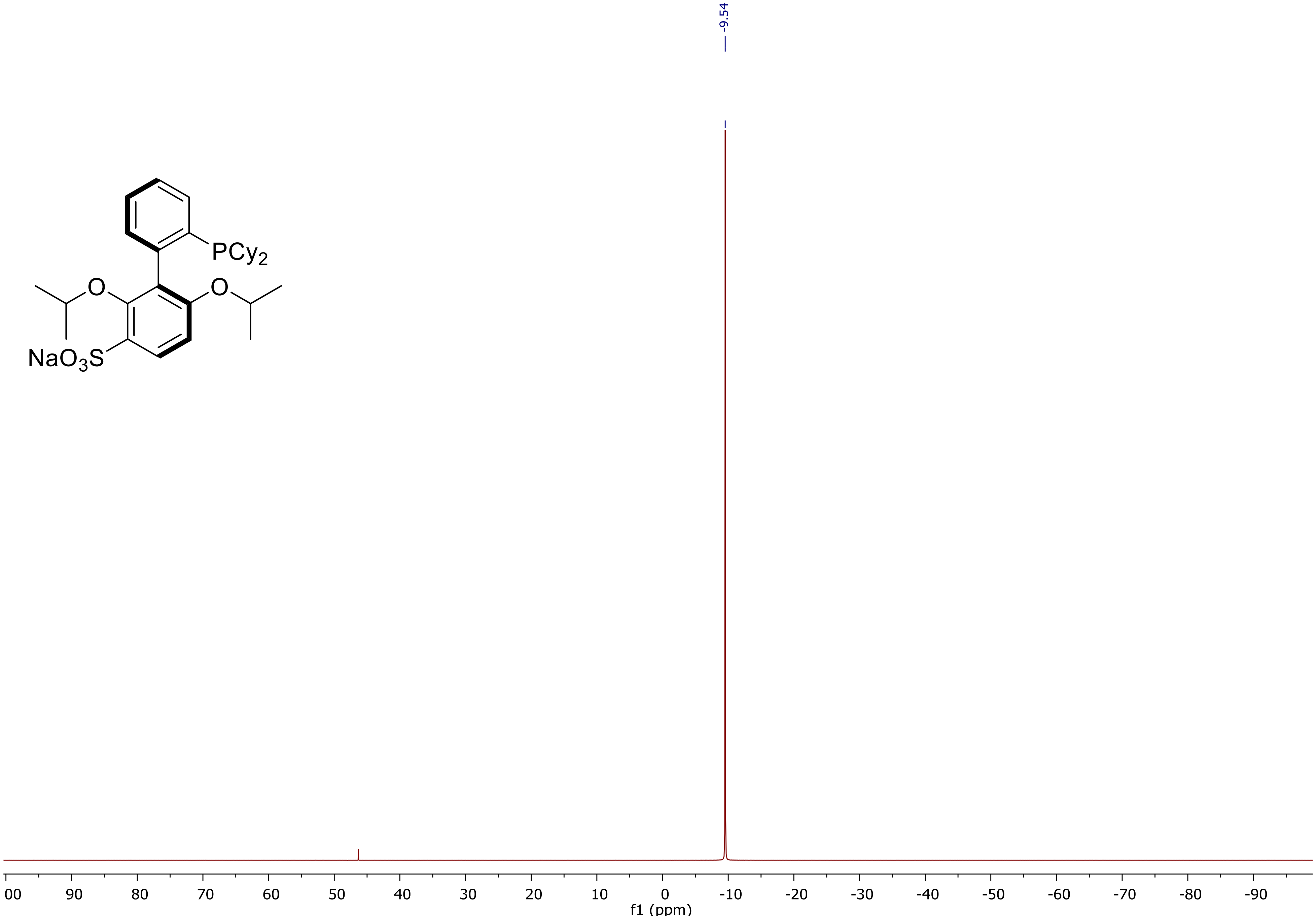

^{13}C-NMR (MeOD): Sodium-2'-(dicyclohexylphosphaneyl)-2,6-diisopropoxy-[1,1'-biphenyl]-3-sulfonate (R)-sRuPhos

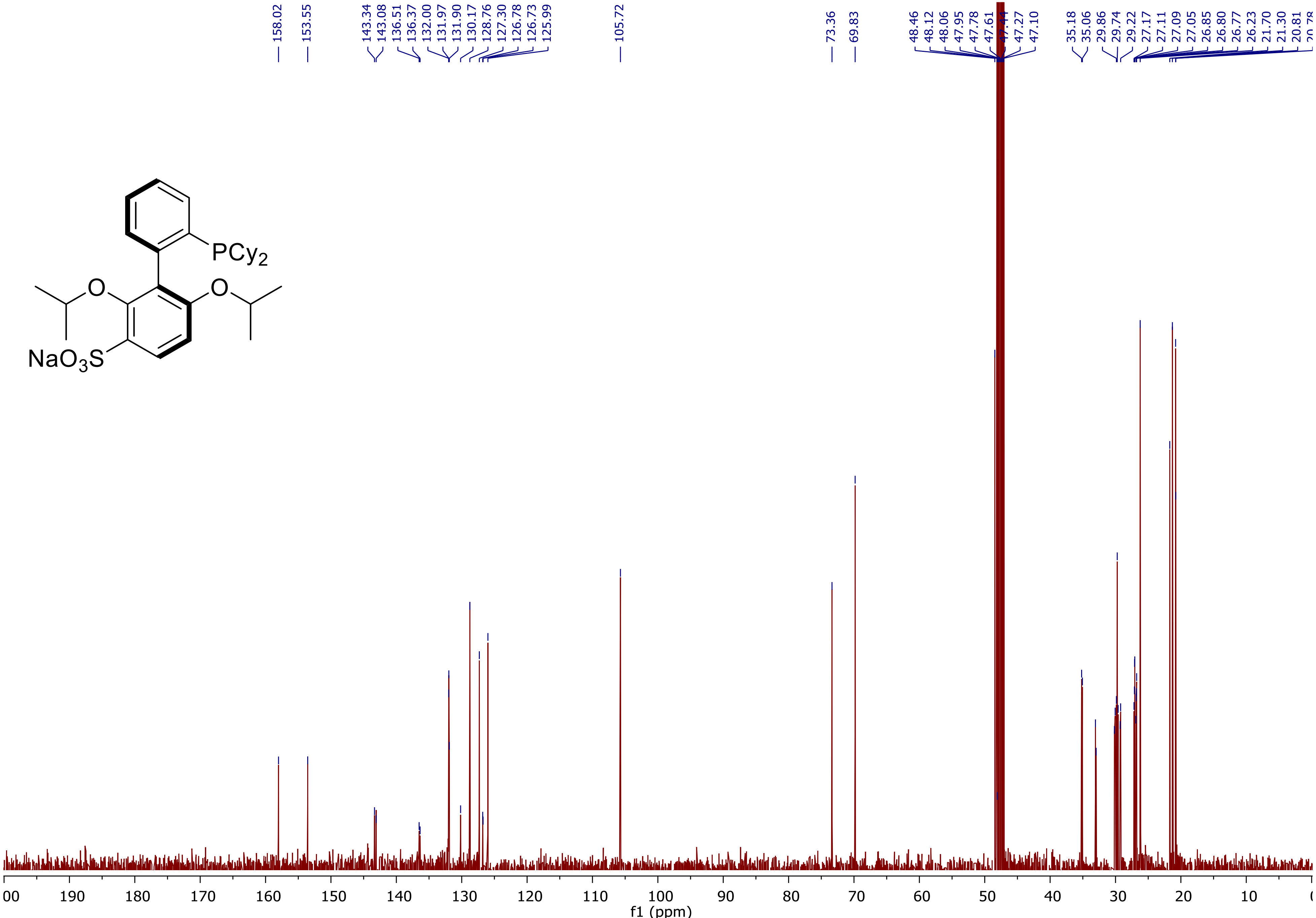

¹H-NMR ($CDCl_3$): Neopentyl (S)-2'-(dicyclohexylphosphoryl)-2,6-diisopropoxy-[1,1'-biphenyl]-3-sulfonate

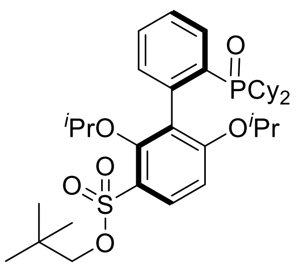

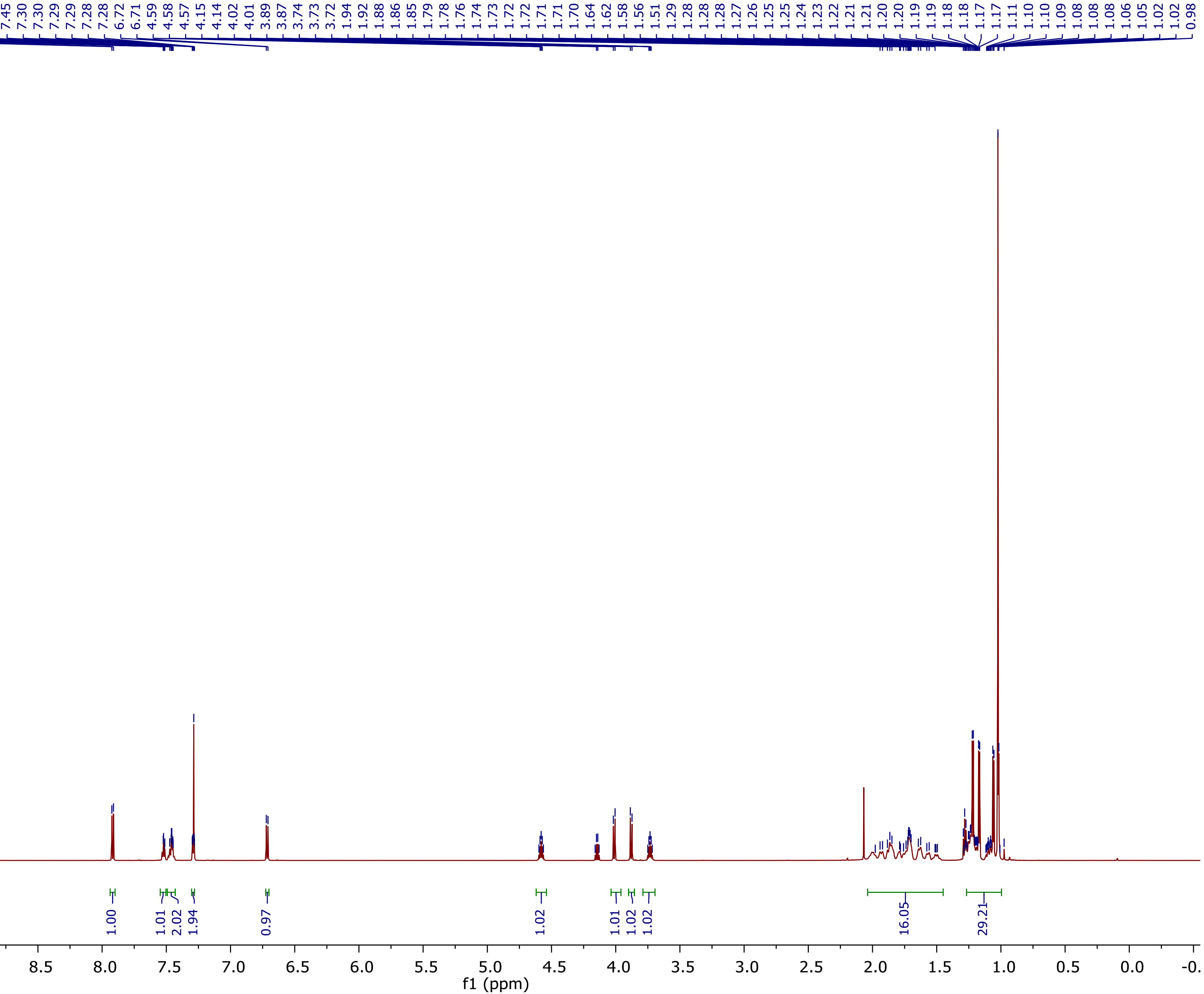

^{31}P-NMR ($CDCl_3$): Neopentyl (S)-2'-(dicyclohexylphosphoryl)-2,6-diisopropoxy-[1,1'-biphenyl]-3-sulfonate

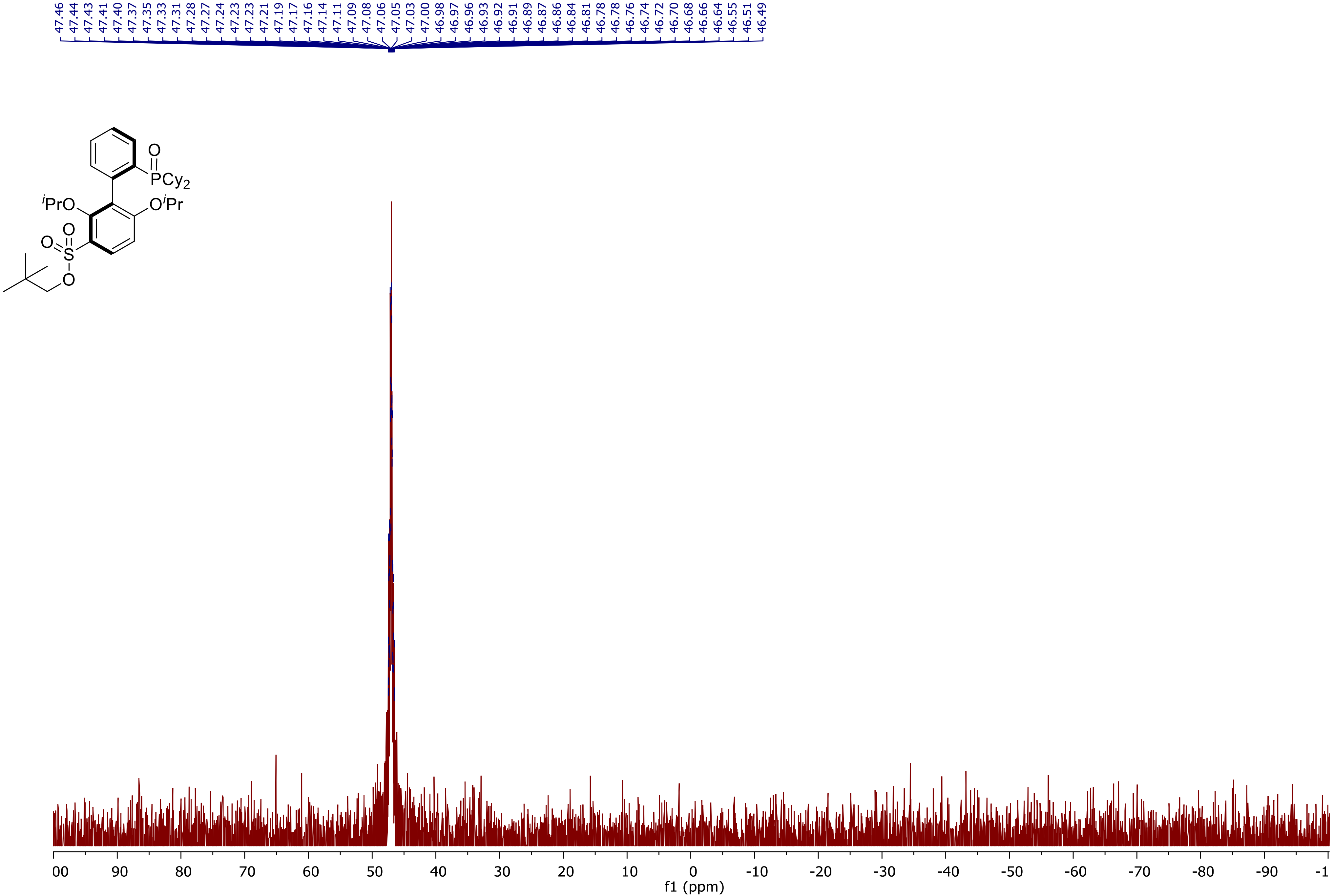

^{13}C-NMR ($CDCl_3$): Neopentyl (S)-2'-(dicyclohexylphosphoryl)-2,6-diisopropoxy-[1,1'-biphenyl]-3-sulfonate

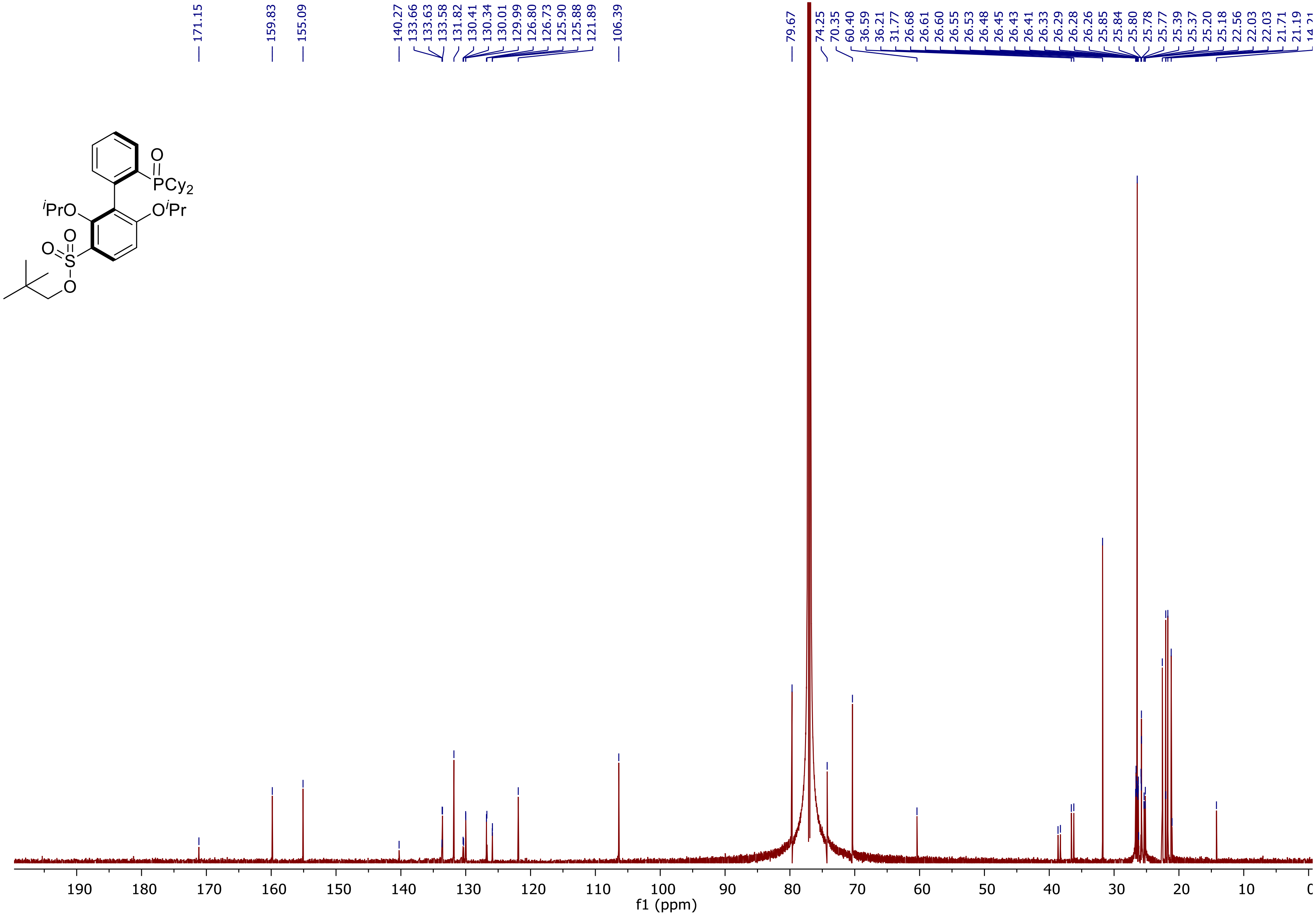

¹H-NMR ($CDCl_3$): Neopentyl (S)-2'-(dicyclohexylphosphaneyl)-2,6-diisopropoxy-[1,1'-biphenyl]-3-sulfonate (S)-sRuPhos-Np

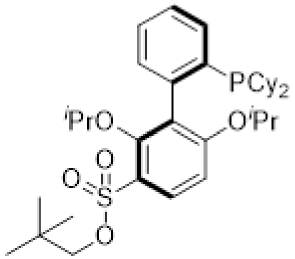

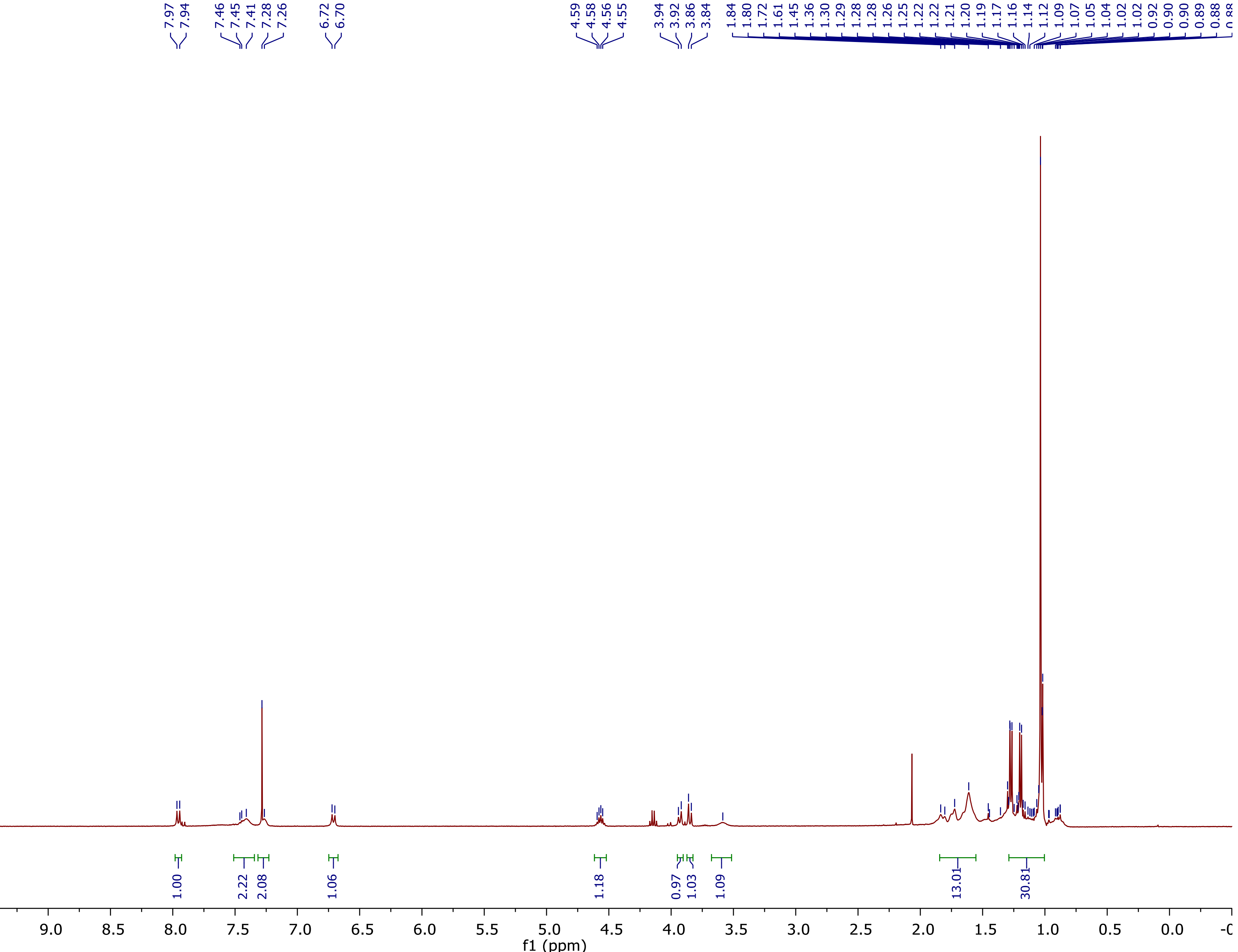

^{31}P-NMR ($CDCl_3$): Neopentyl (S)-2'-(dicyclohexylphosphaneyl)-2,6-diisopropoxy-[1,1'-biphenyl]-3-sulfonate (S)-sRuPhos-Np

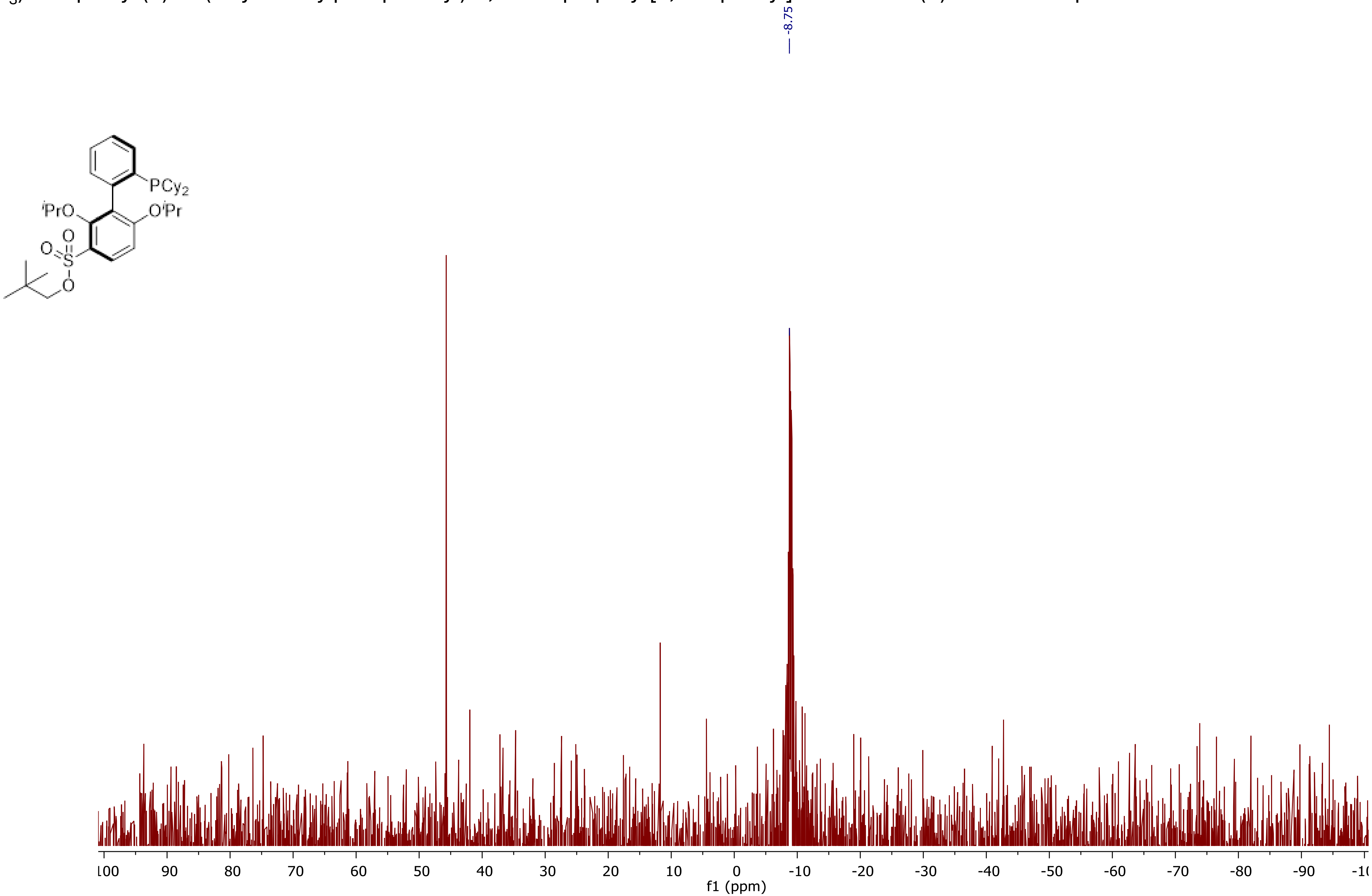

^{13}C-NMR (C_6D_6): Neopentyl (S)-2'-(dicyclohexylphosphaneyl)-2,6-diisopropoxy-[1,1'-biphenyl]-3-sulfonate (S)-sRuPhos-Np

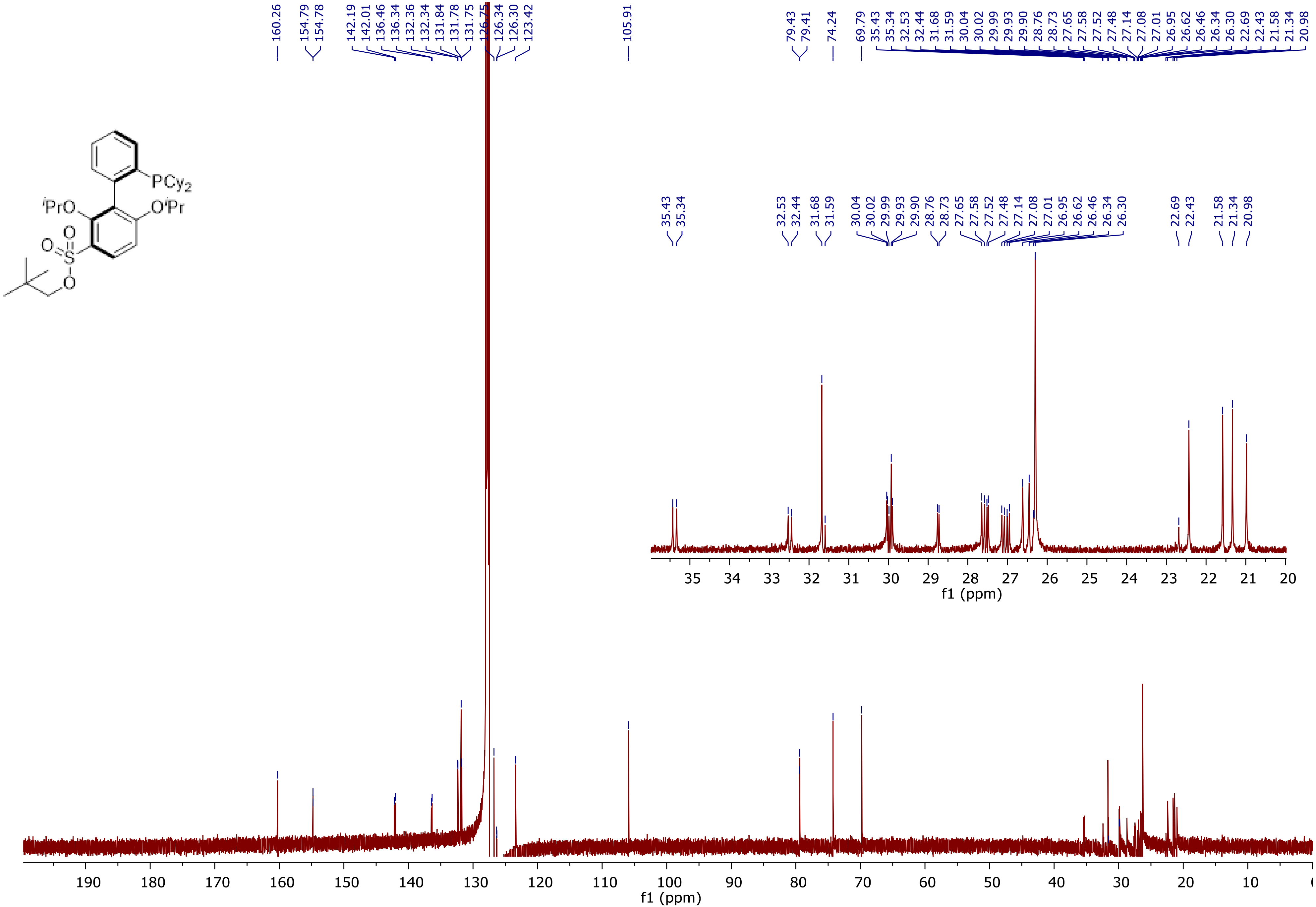

^{1}H-NMR ($CDCl_3$): 3-fluoro-2-(4,4,5,5-tetramethyl-1,3,2-dioxaborolan-2-yl)phenol

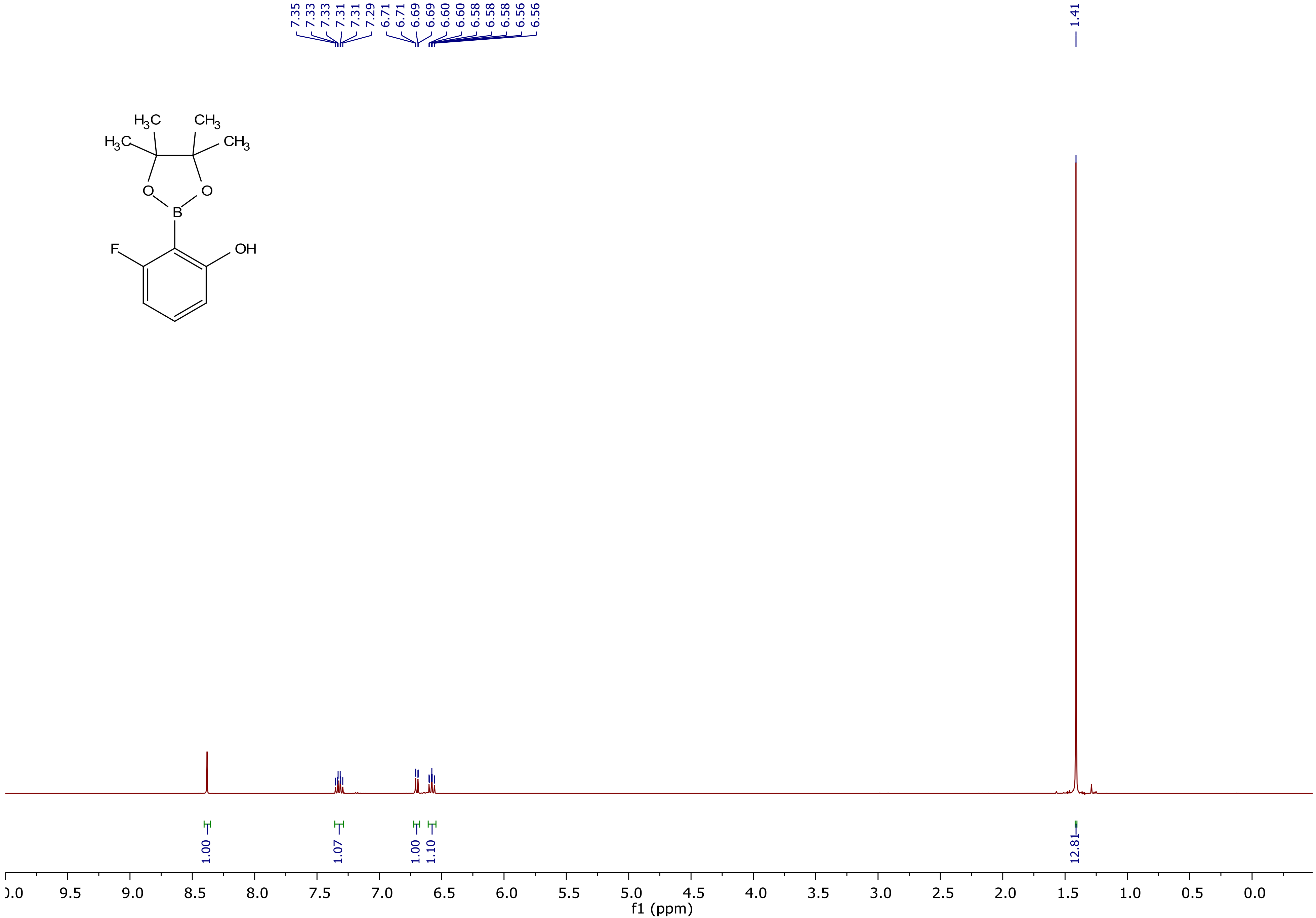

^{19}F-NMR ($CDCl_3$): 3-fluoro-2-(4,4,5,5-tetramethyl-1,3,2-dioxaborolan-2-yl)phenol

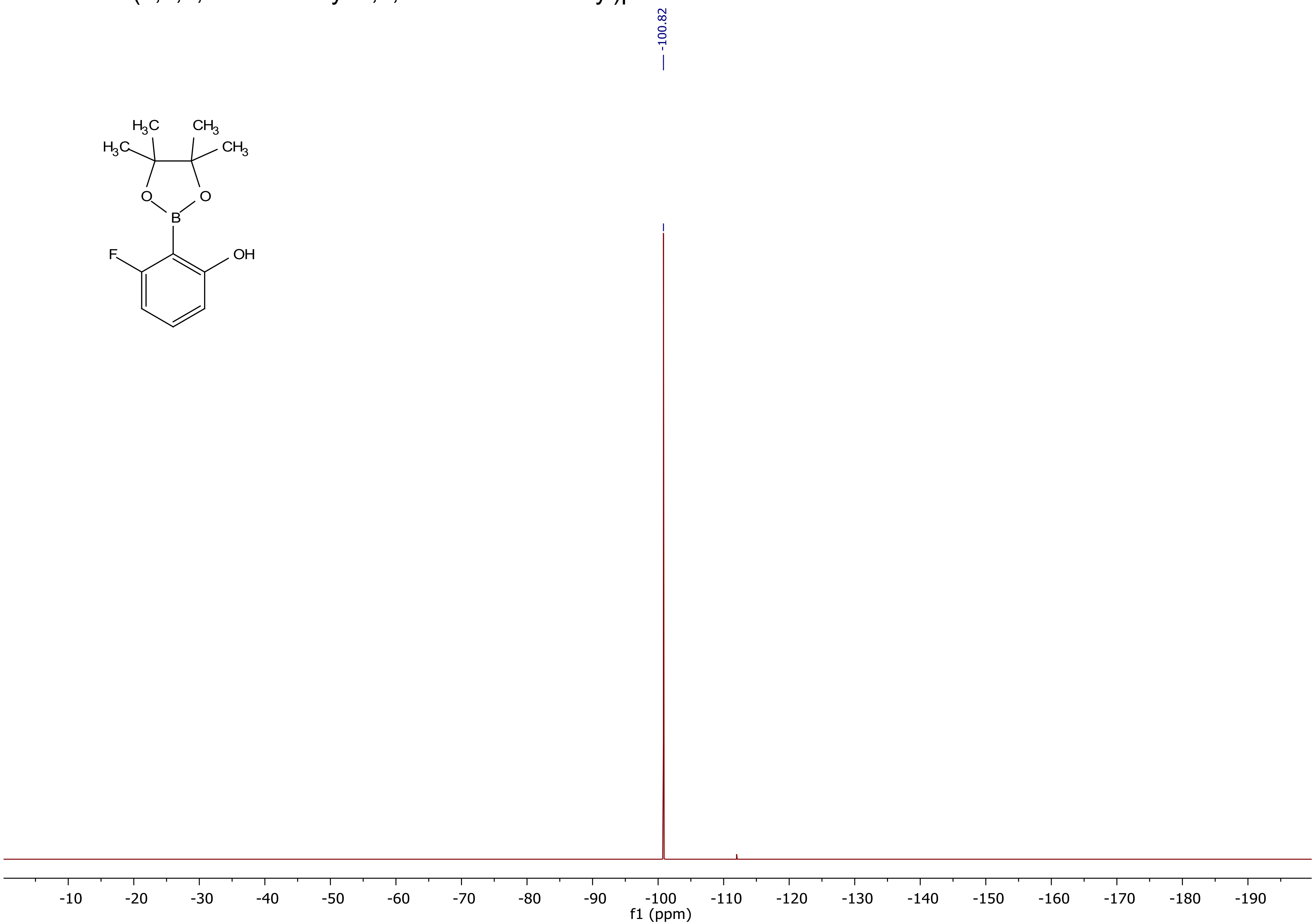

^{13}C-NMR ($CDCl_3$): 3-fluoro-2-(4,4,5,5-tetramethyl-1,3,2-dioxaborolan-2-yl)phenol

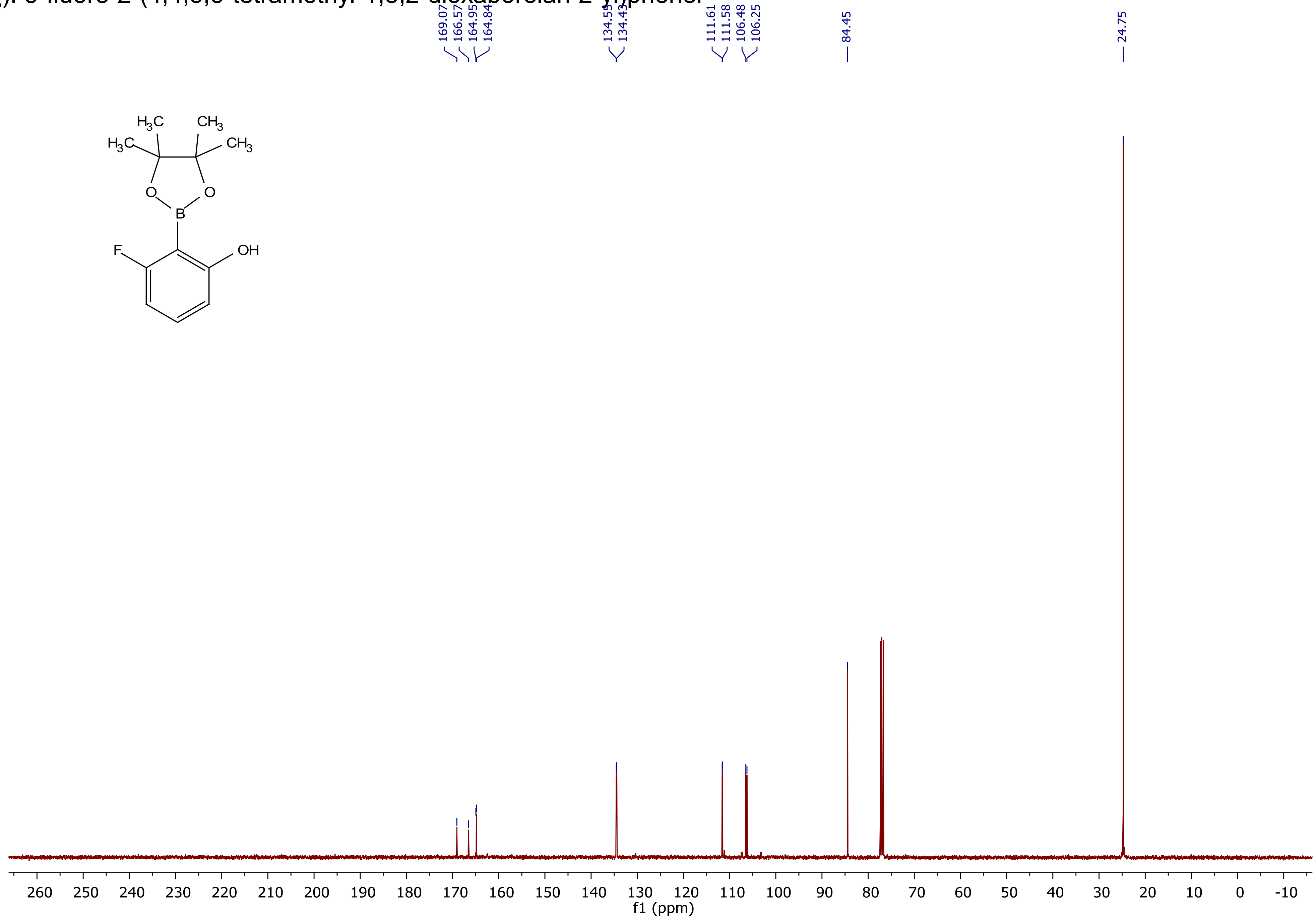

^{1}H-NMR ($CDCl_3$):3-chloro-2-(4,4,5,5-tetramethyl-1,3,2-dioxaborolan-2-yl)phenol

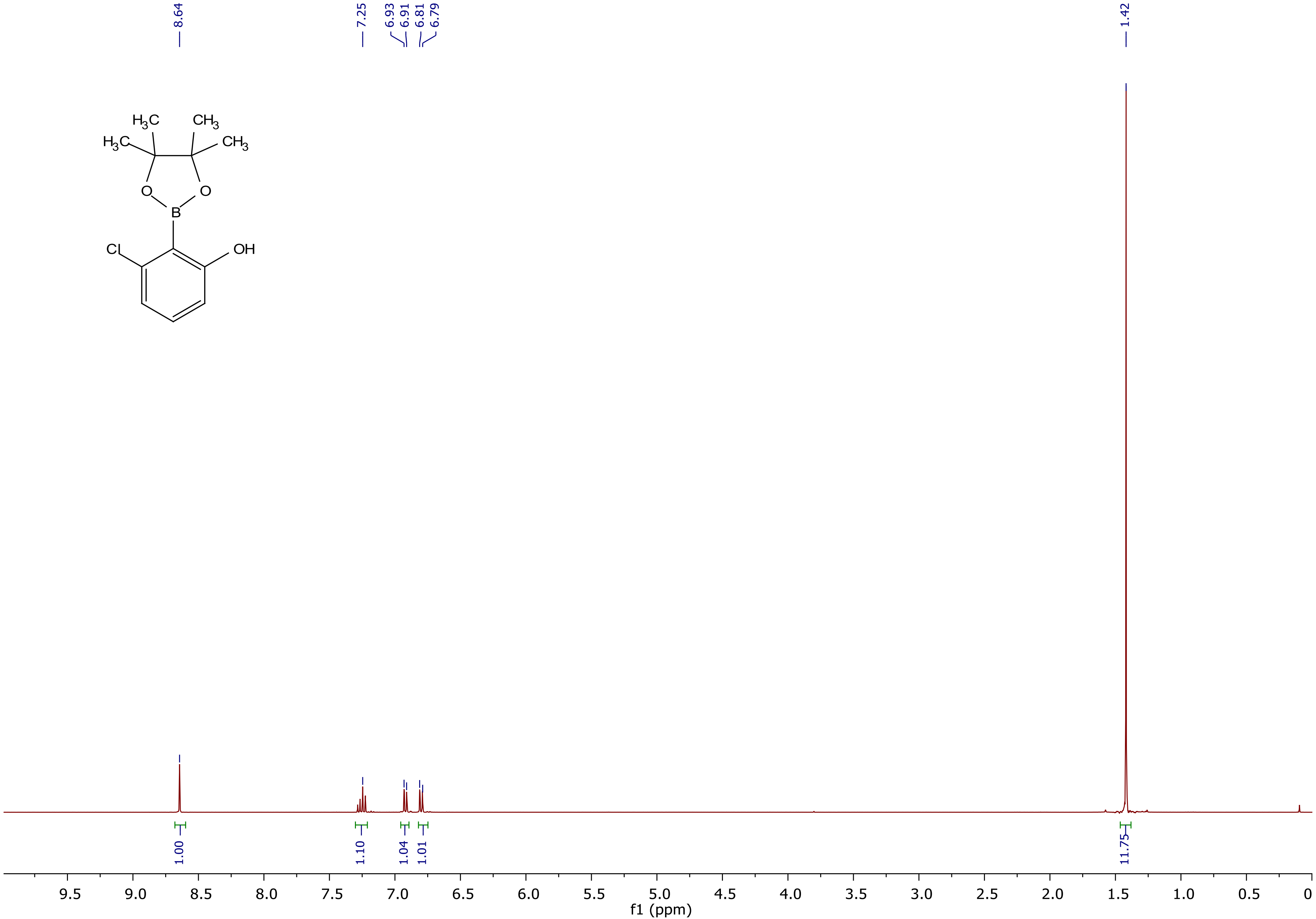

^{13}C-NMR ($CDCl_3$):3-chloro-2-(4,4,5,5-tetramethyl-1,3,2-dioxaborolan-2-yl)phenol

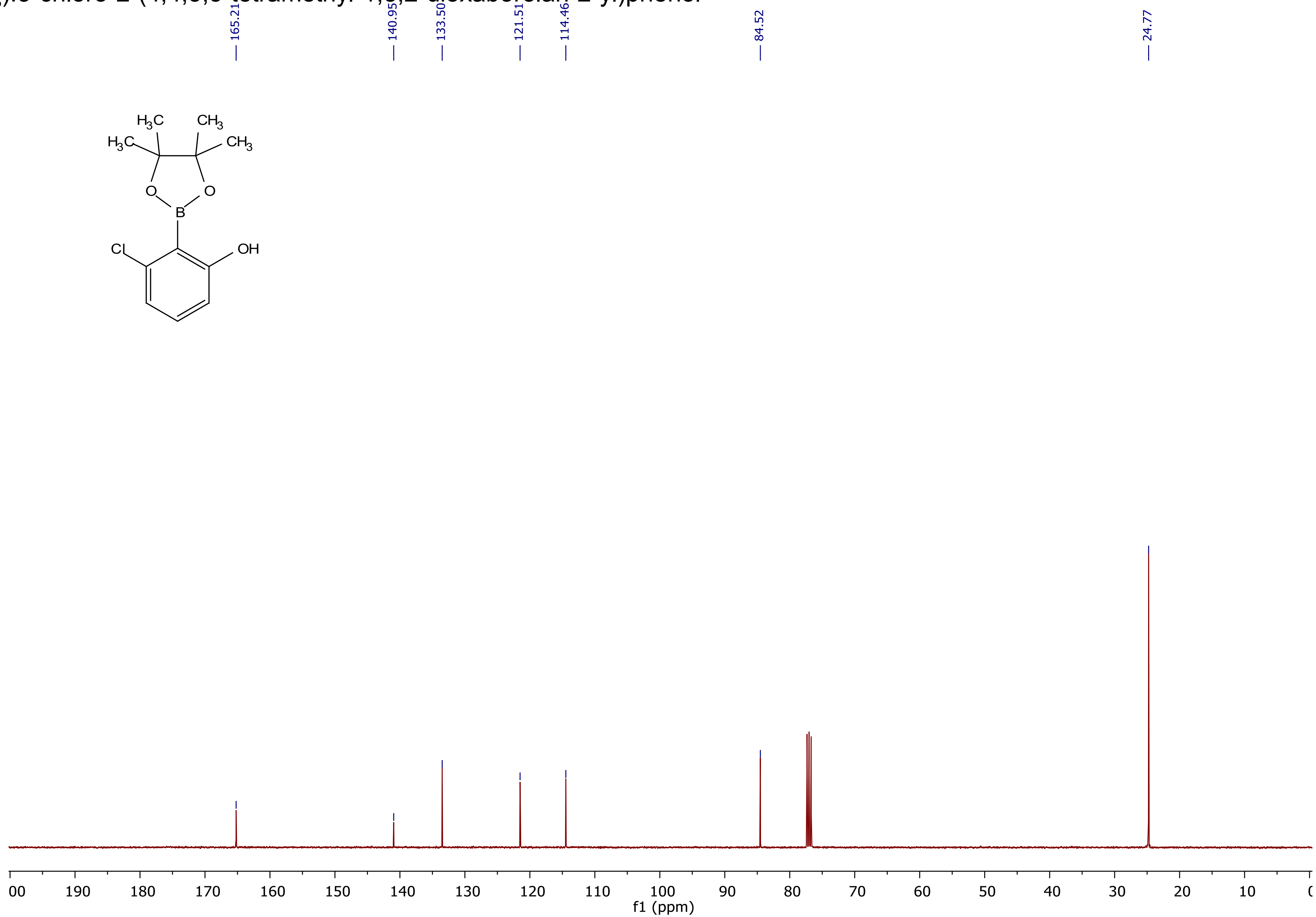

^{1}H-NMR ($CDCl_3$): 3,4-difluoro-2-(4,4,5,5-tetramethyl-1,3,2-dioxaborolan-2-yl)phenol

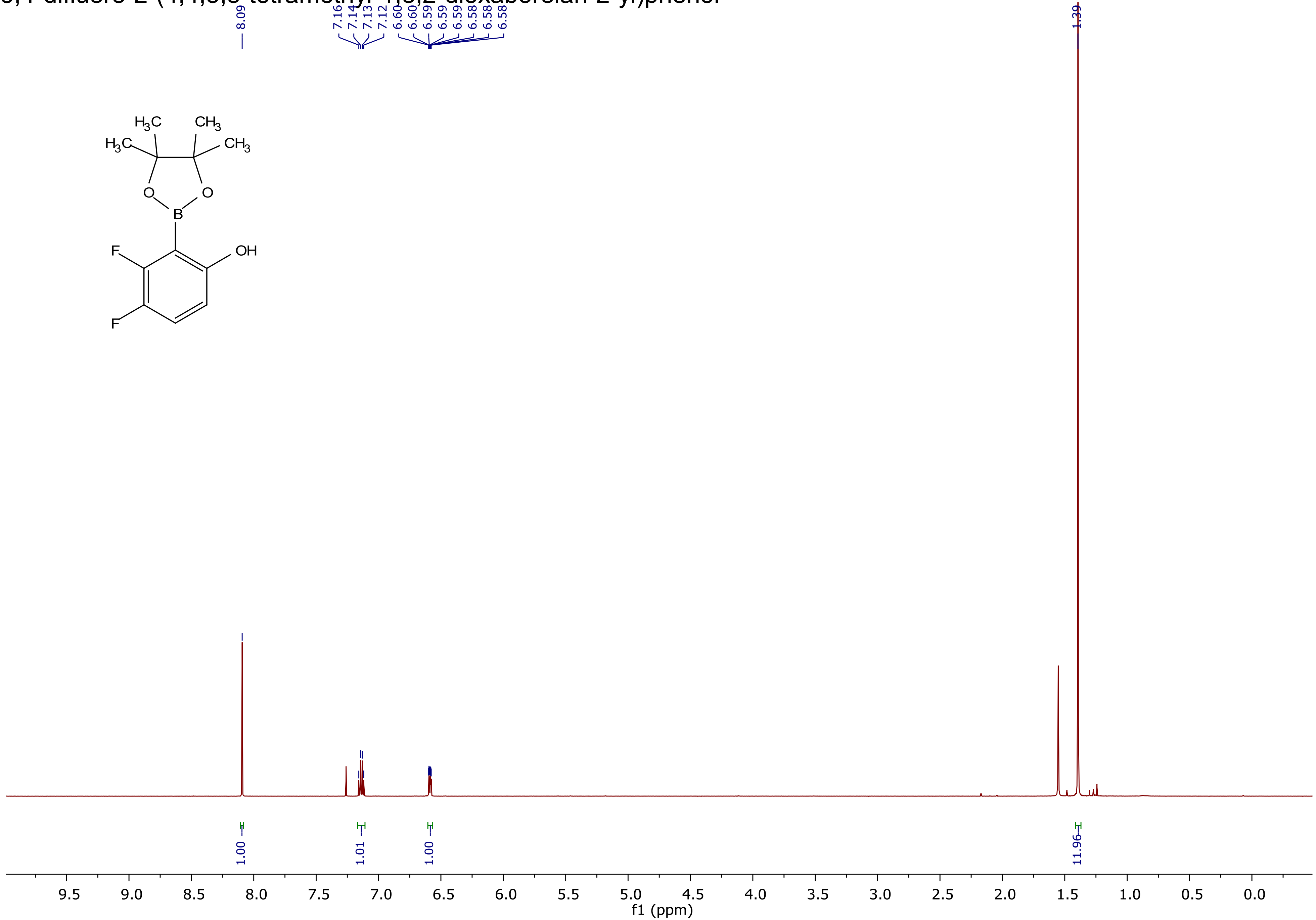

^{19}F-NMR ($CDCl_3$): 3,4-difluoro-2-(4,4,5,5-tetramethyl-1,3,2-dioxaborolan-2-yl)phenol

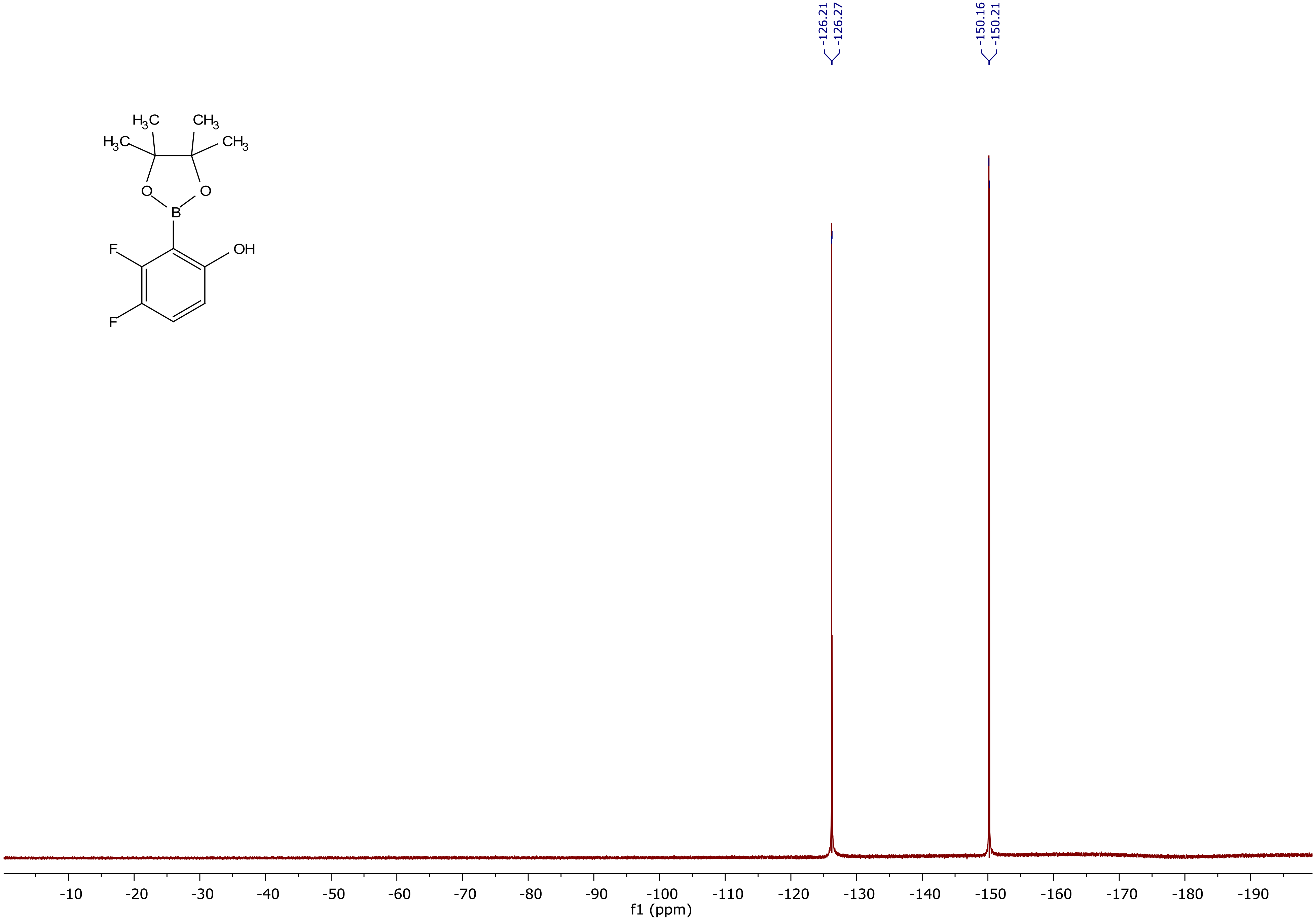

^{13}C-NMR ($CDCl_3$): 3,4-difluoro-2-(4,4,5,5-tetramethyl-1,3,2-dioxaborolan-2-yl)phenol

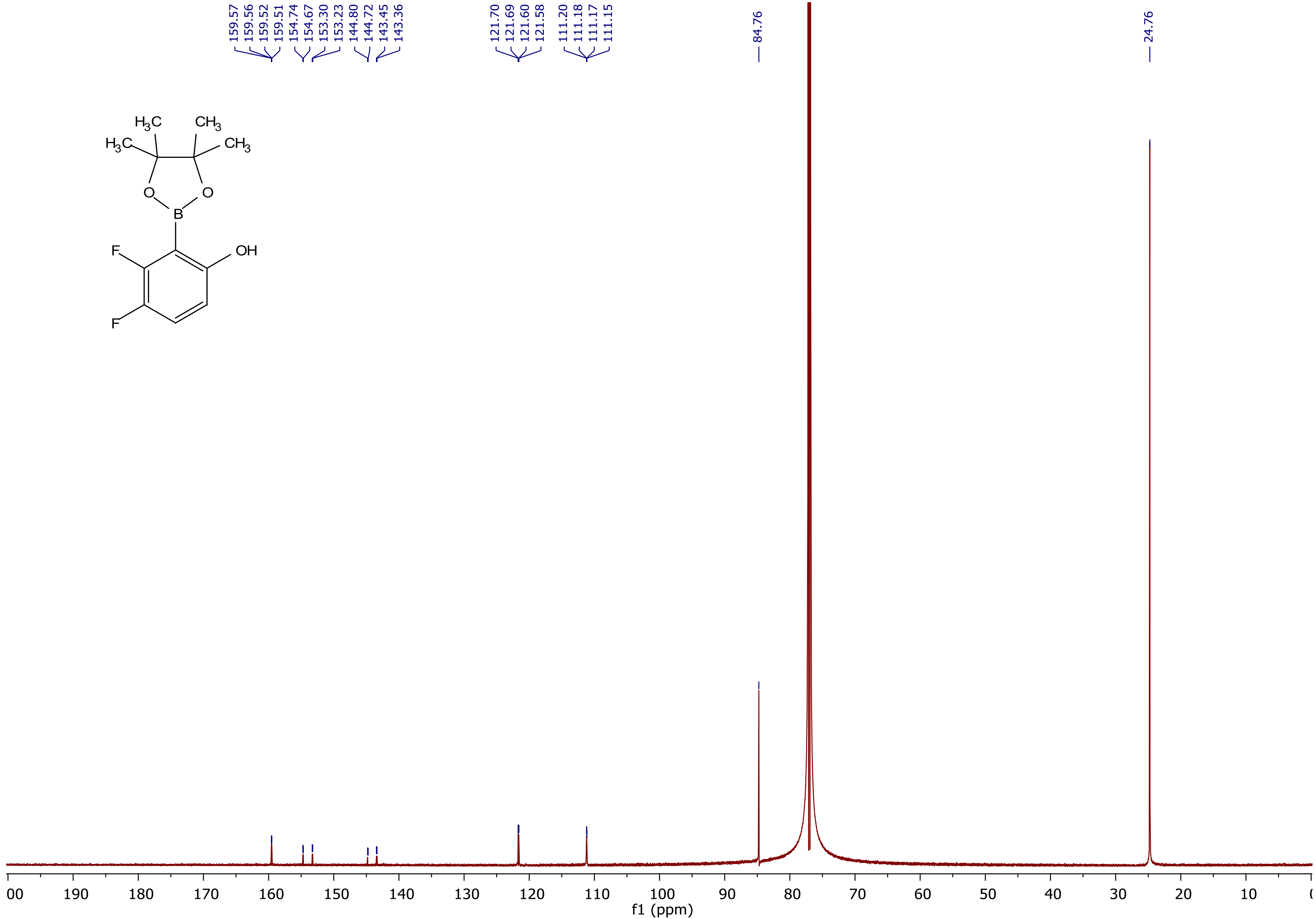

^{1}H-NMR ($CDCl_3$): 3-methyl-2-(4,4,5,5-tetramethyl-1,3,2-dioxaborolan-2-yl)phenol

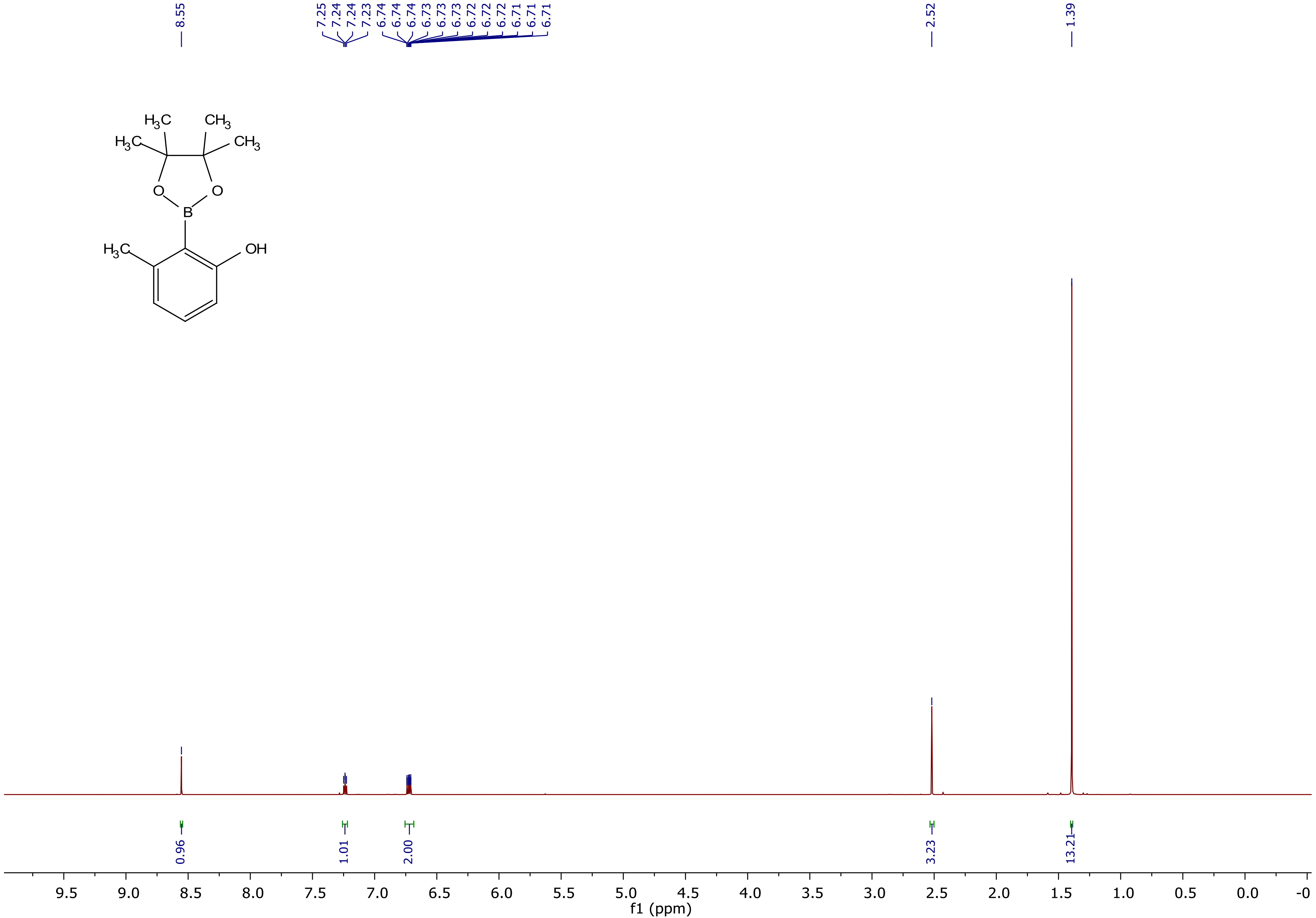

^{13}C-NMR ($CDCl_3$): 3-methyl-2-(4,4,5,5-tetramethyl-1,3,2-dioxaborolan-2-yl)phenol

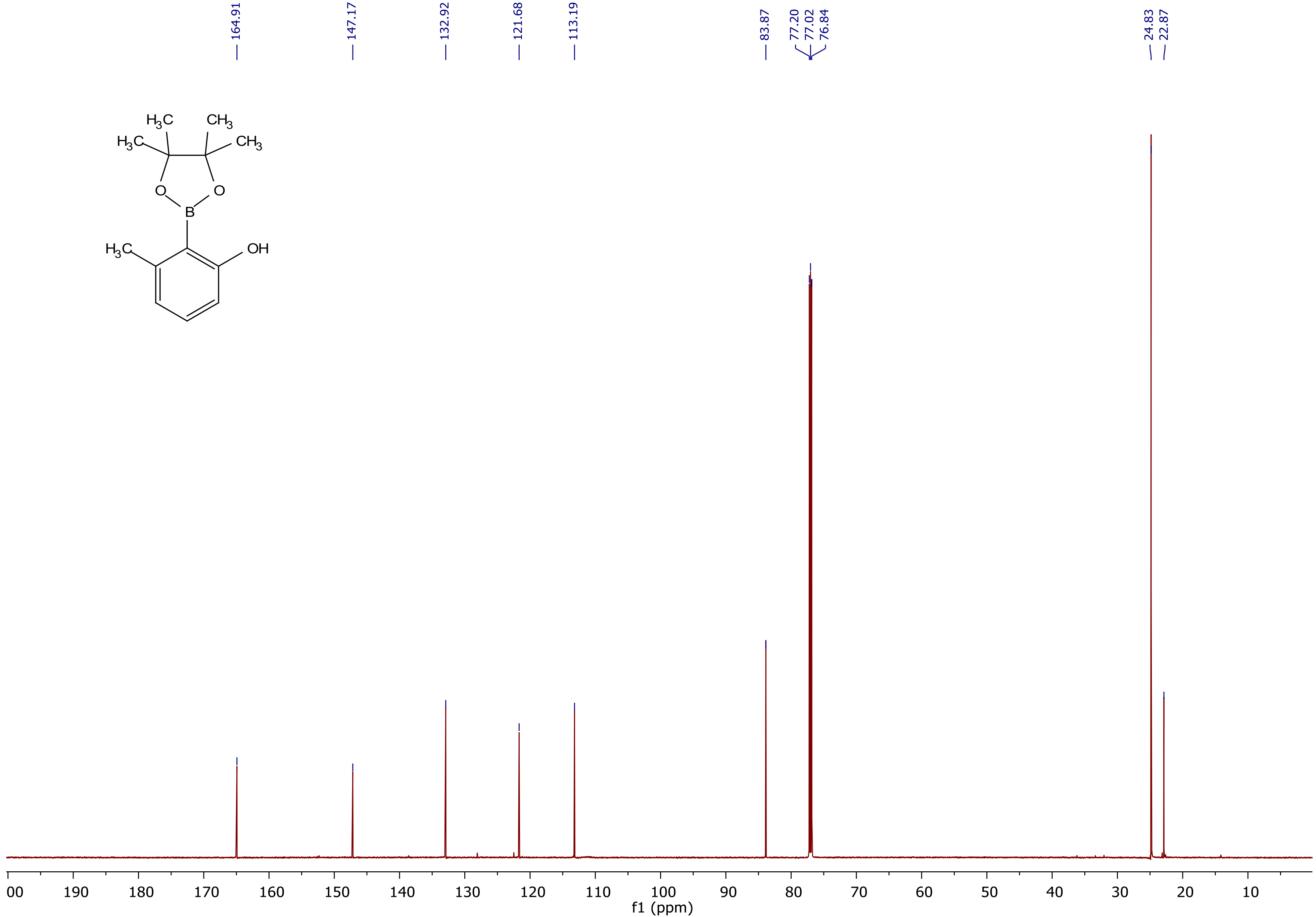

^{1}H-NMR ($CDCl_3$): 1-(4,4,5,5-tetramethyl-1,3,2-dioxaborolan-2-yl)-5,6,7,8-tetrahydronaphthalen-2-ol

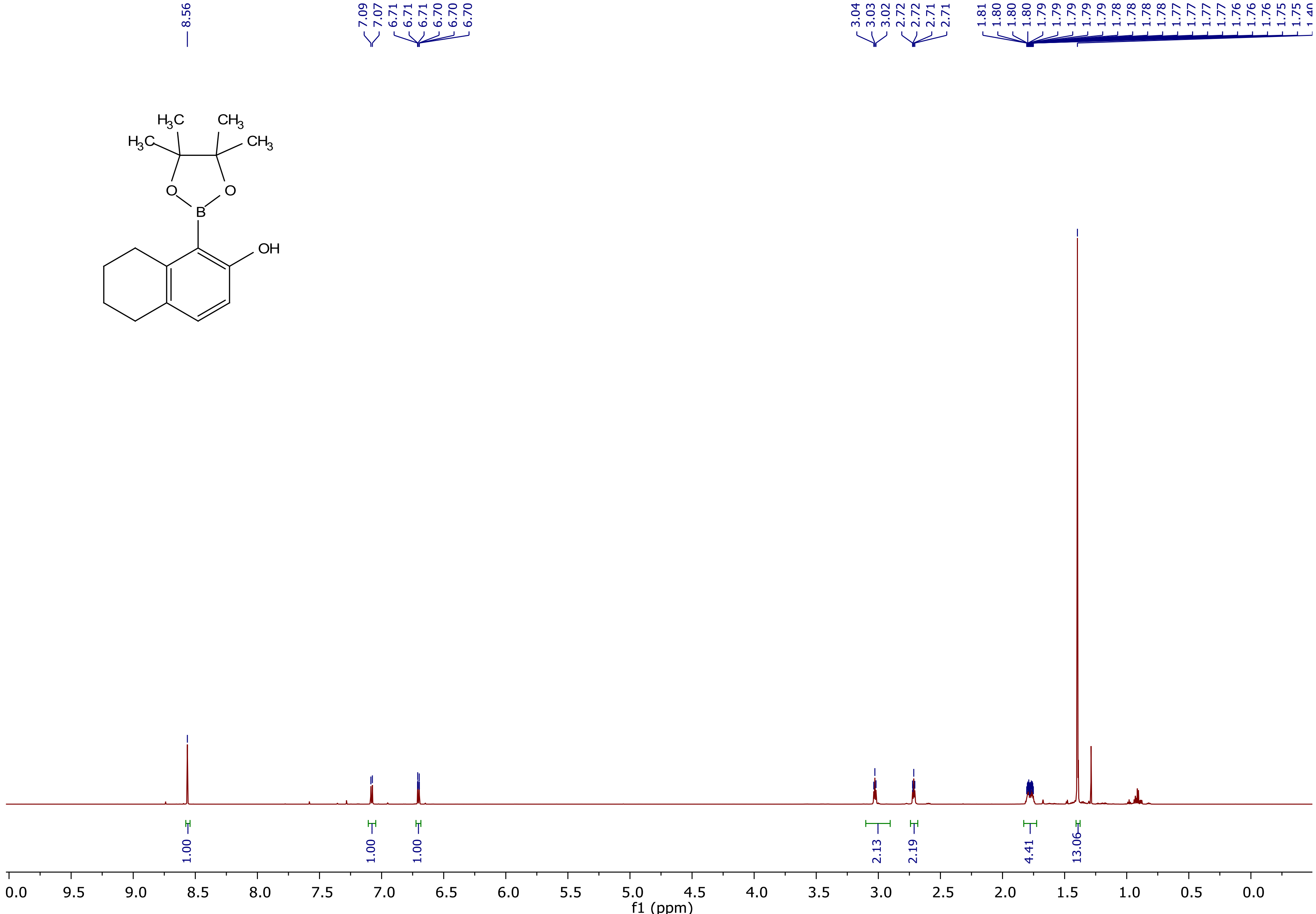

^{13}C-NMR ($CDCl_3$): 1-(4,4,5,5-tetramethyl-1,3,2-dioxaborolan-2-yl)-5,6,7,8-tetrahydronaphthalen-2-ol

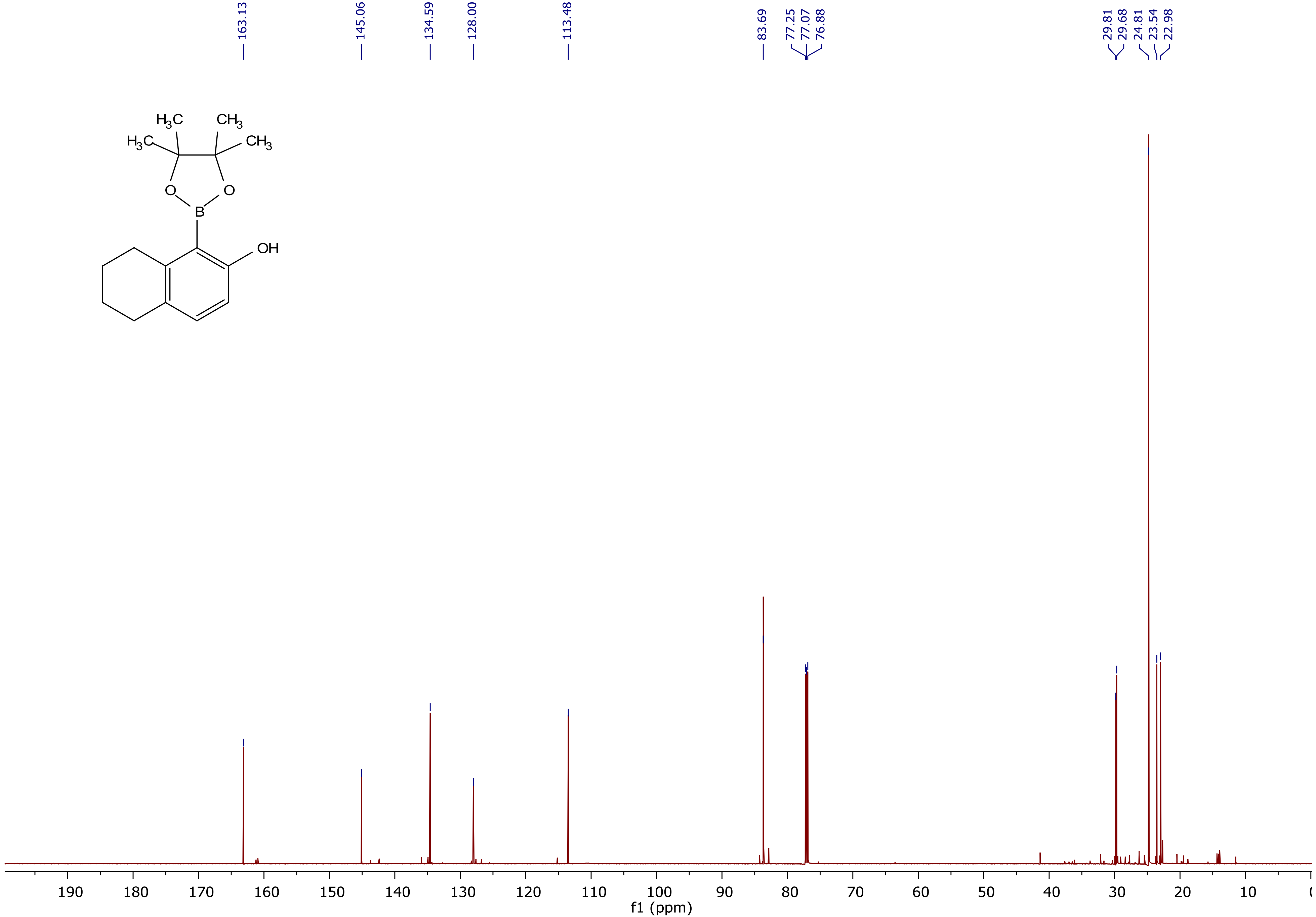

¹H-NMR ($CDCl_3$): 2-(2-fluoro-6-methoxyphenyl)-4,4,5,5-tetramethyl-1,3,2-dioxaborolane

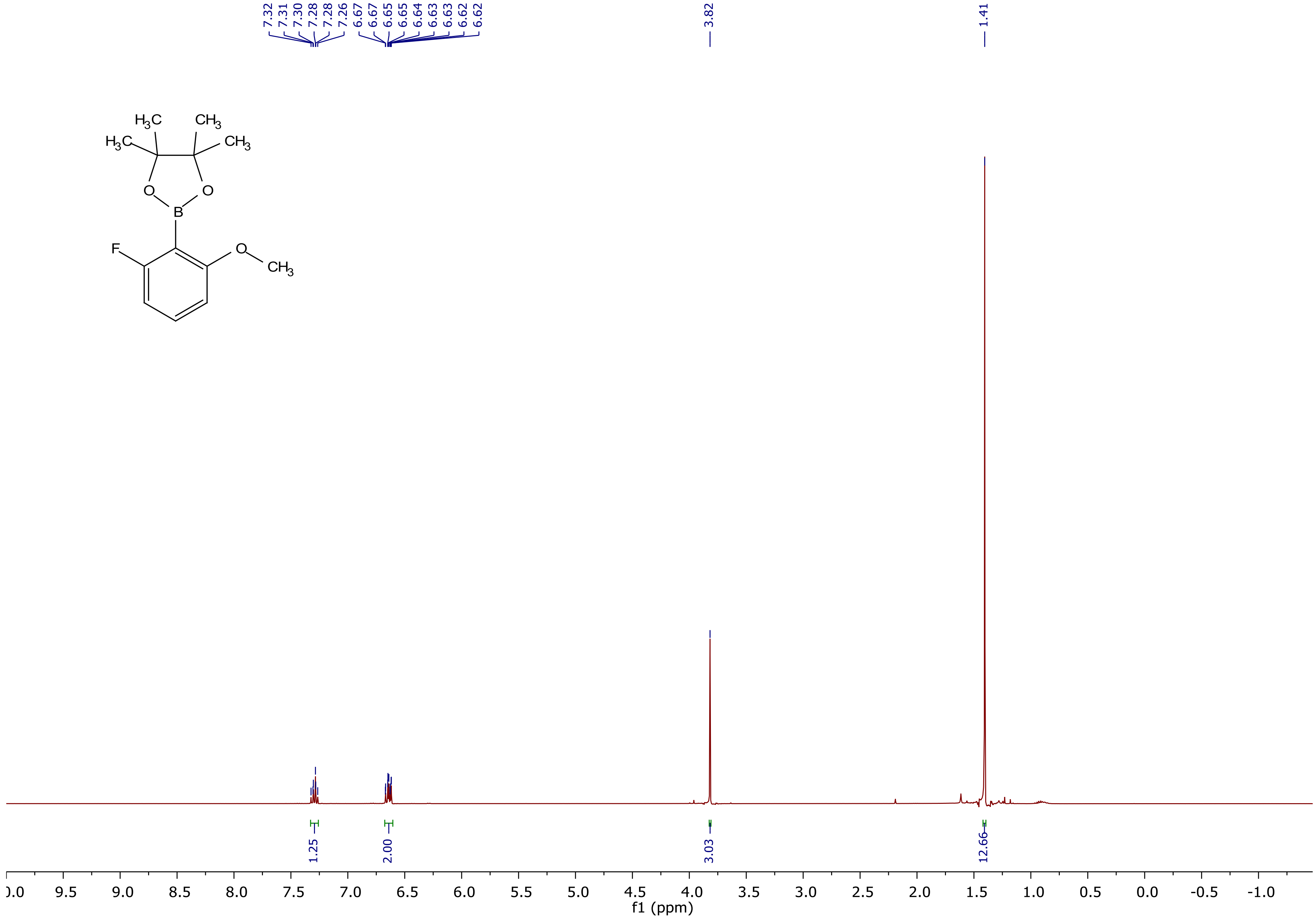

^{19}F-NMR ($CDCl_3$): 2-(2-fluoro-6-methoxyphenyl)-4,4,5,5-tetramethyl-1,3,2-dioxaborolane

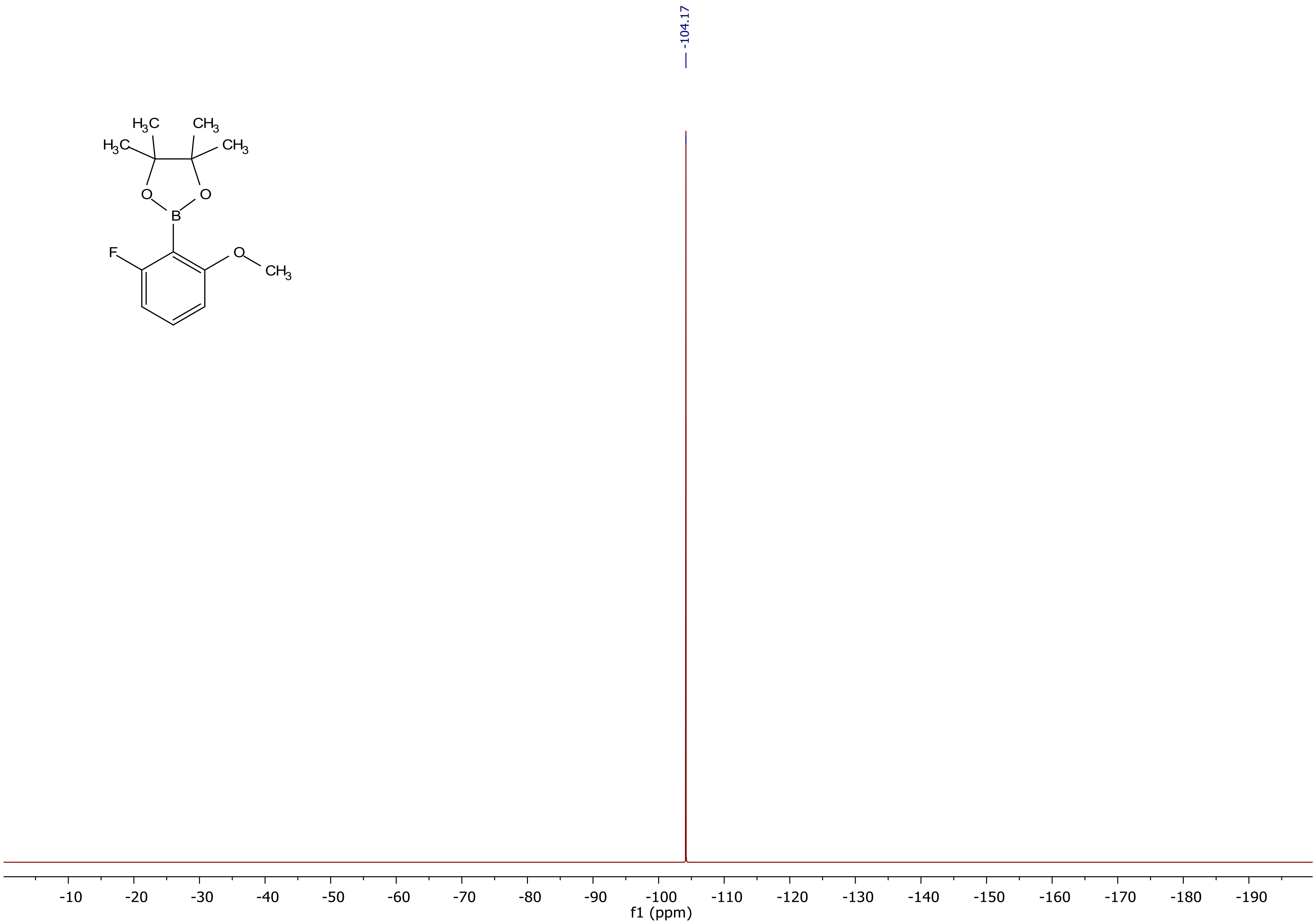

^{13}C-NMR ($CDCl_3$): 2-(2-fluoro-6-methoxyphenyl)-4,4,5,5-tetramethyl-1,3,2-dioxaborolane

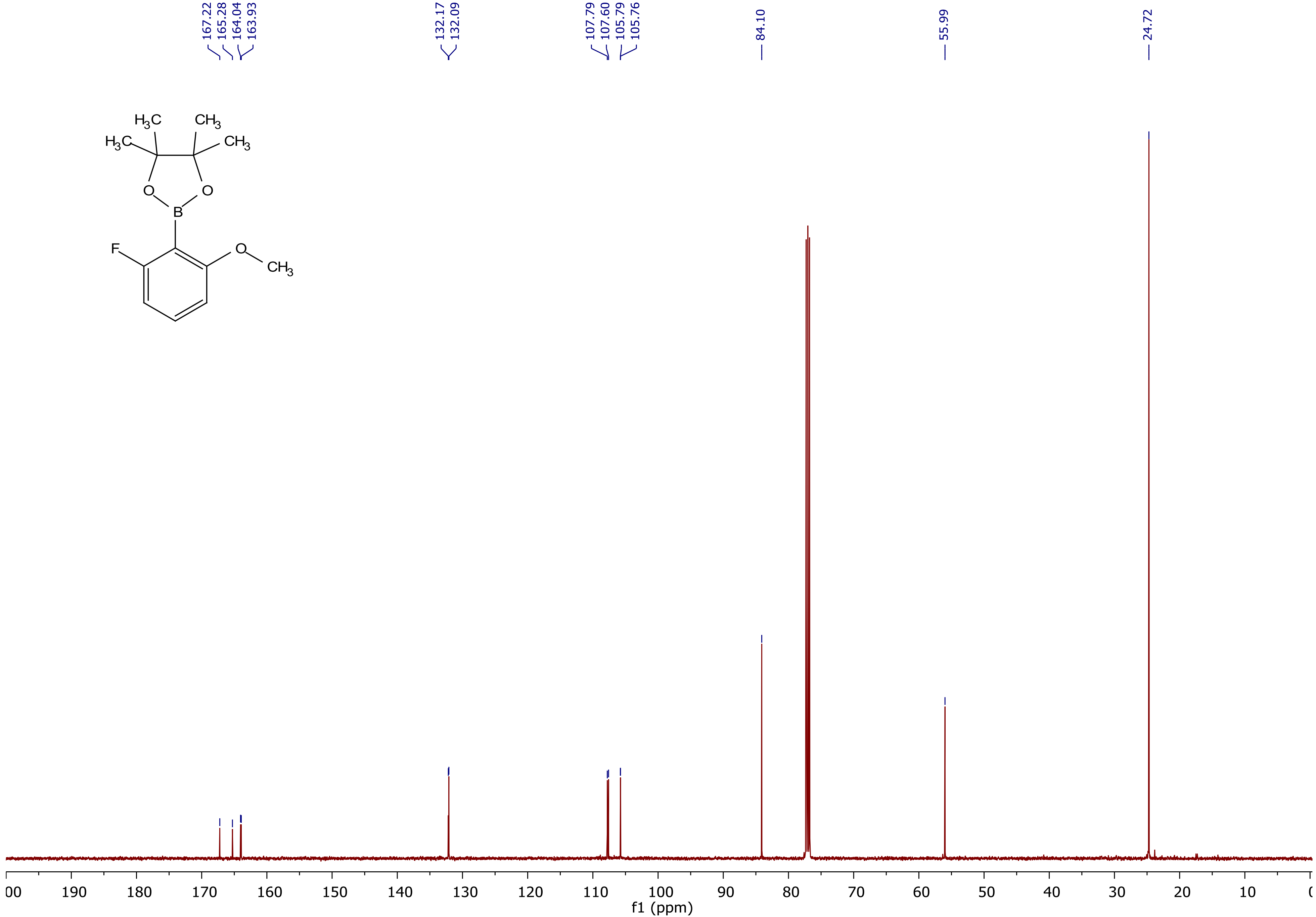

^{1}H-NMR ($CDCl_3$): 2-bromo-3,4-dichloroaniline

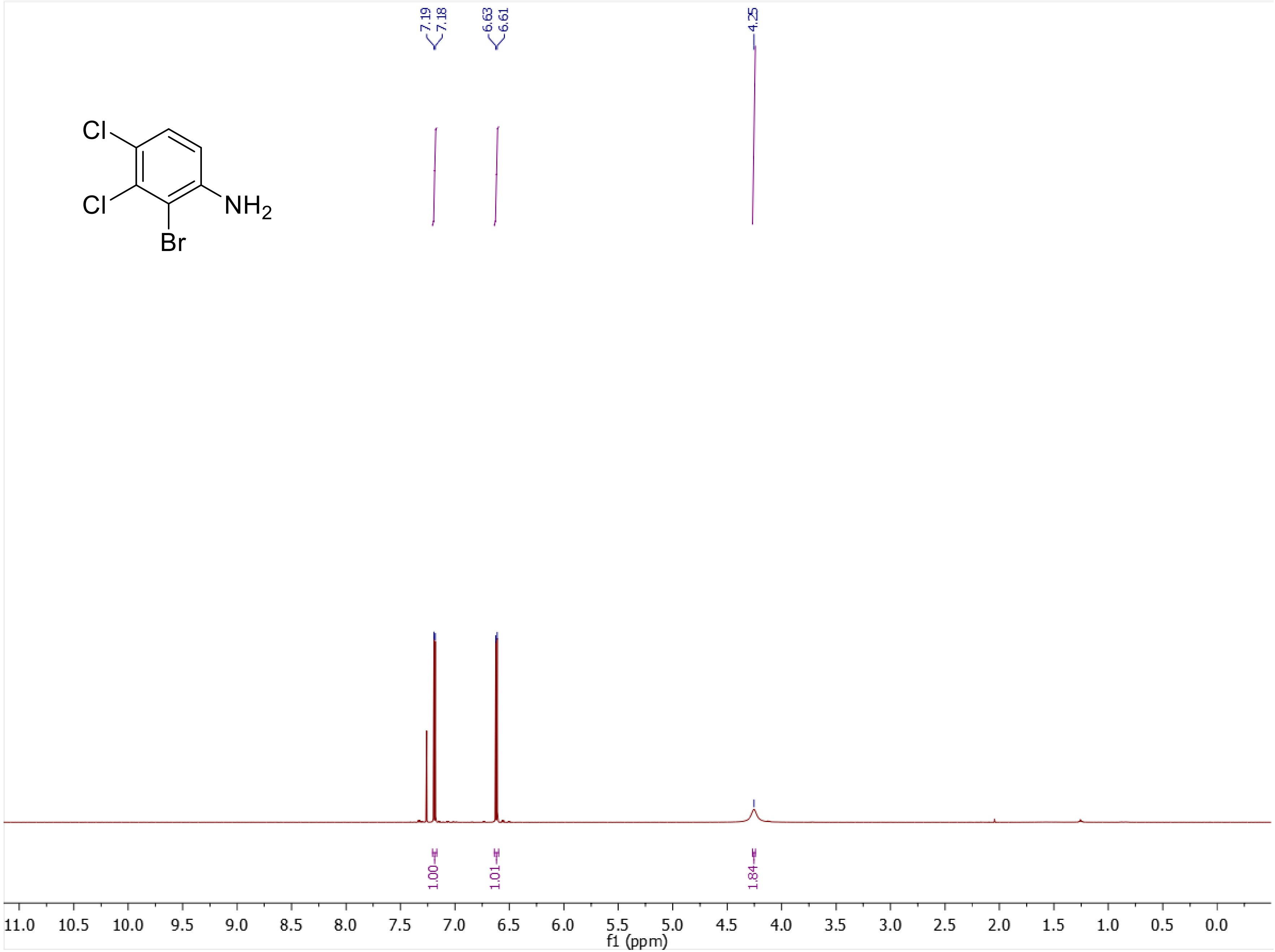

^{13}C-NMR ($CDCl_3$) 2-bromo-3,4-dichloroaniline

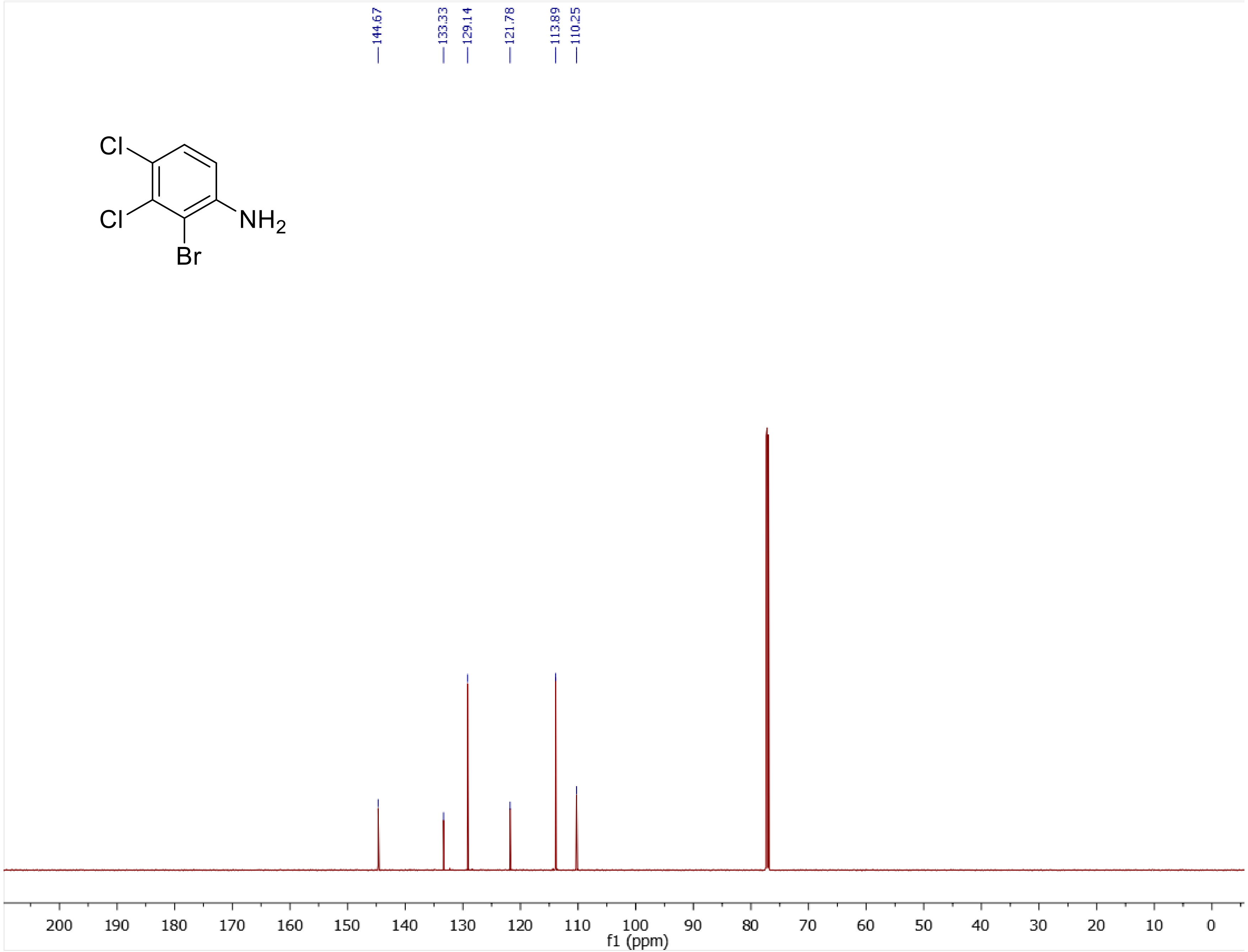

^{1}H-NMR ($CDCl_3$): 2-bromo-4-chloro-3-fluoroaniline

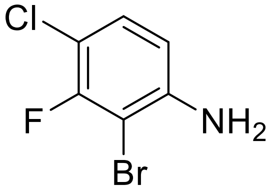

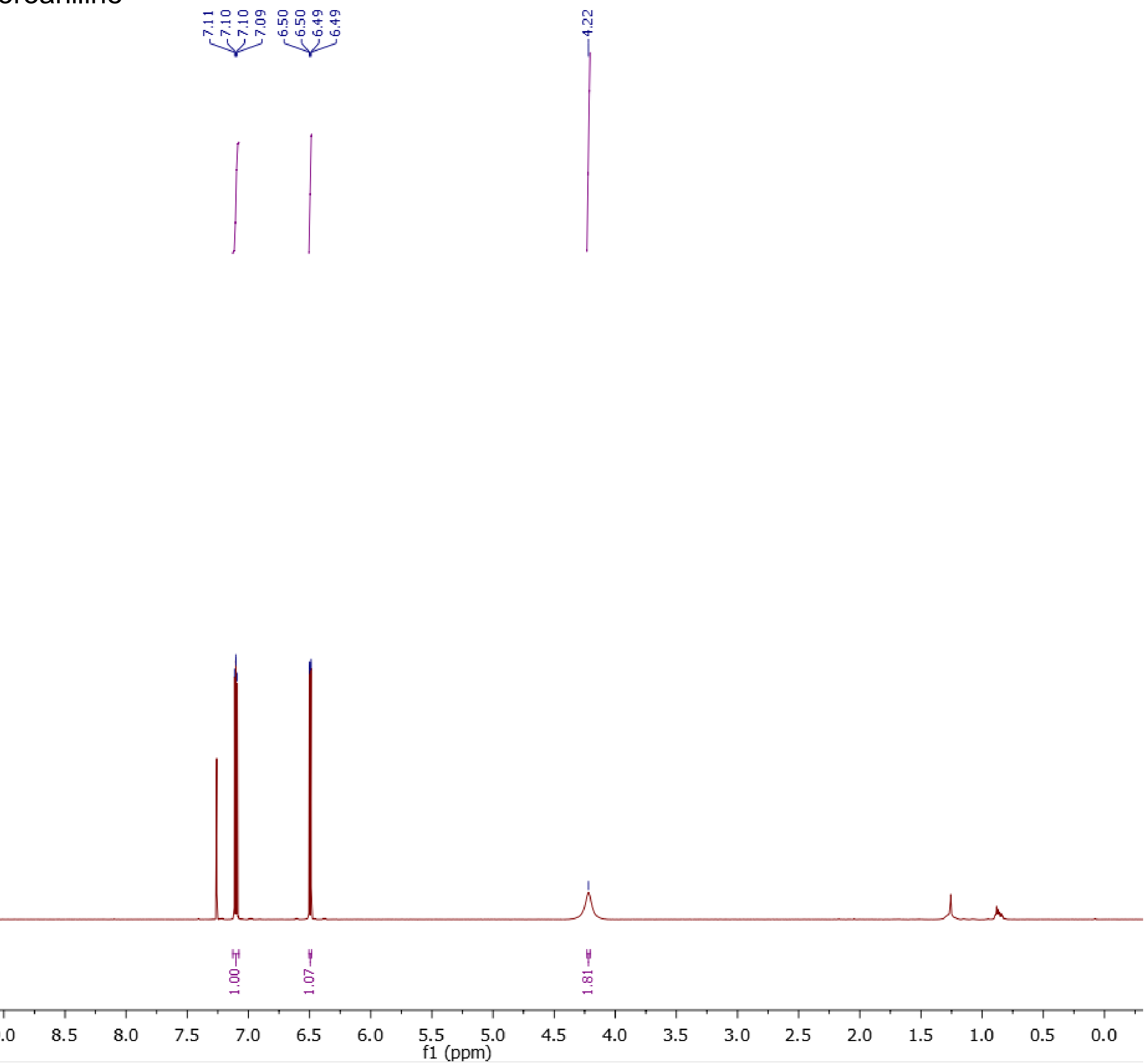

^{19}F-NMR ($CDCl_3$): 2-bromo-4-chloro-3-fluoroaniline

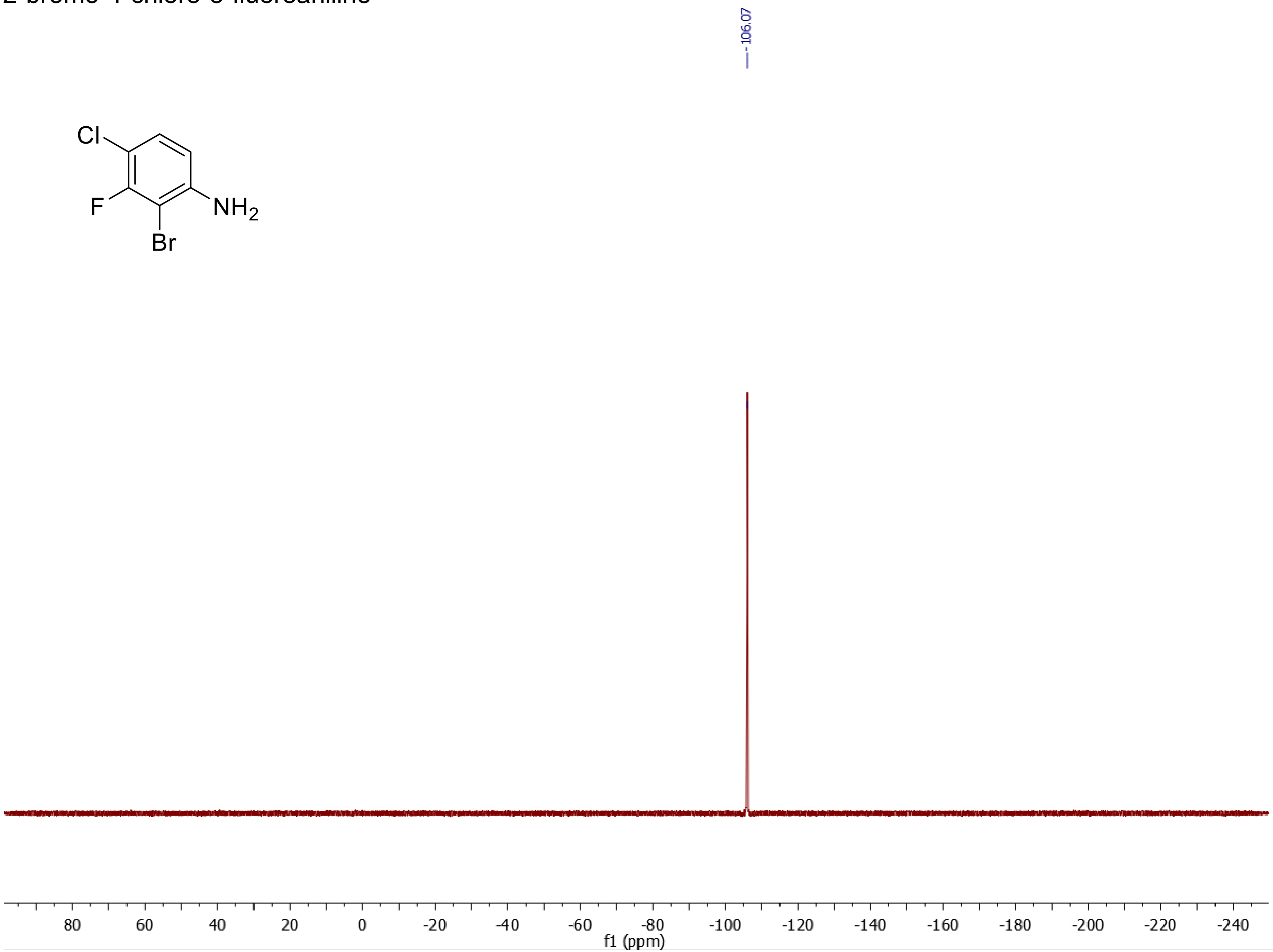

^{13}C-NMR ($CDCl_3$): 2-bromo-4-chloro-3-fluoroaniline

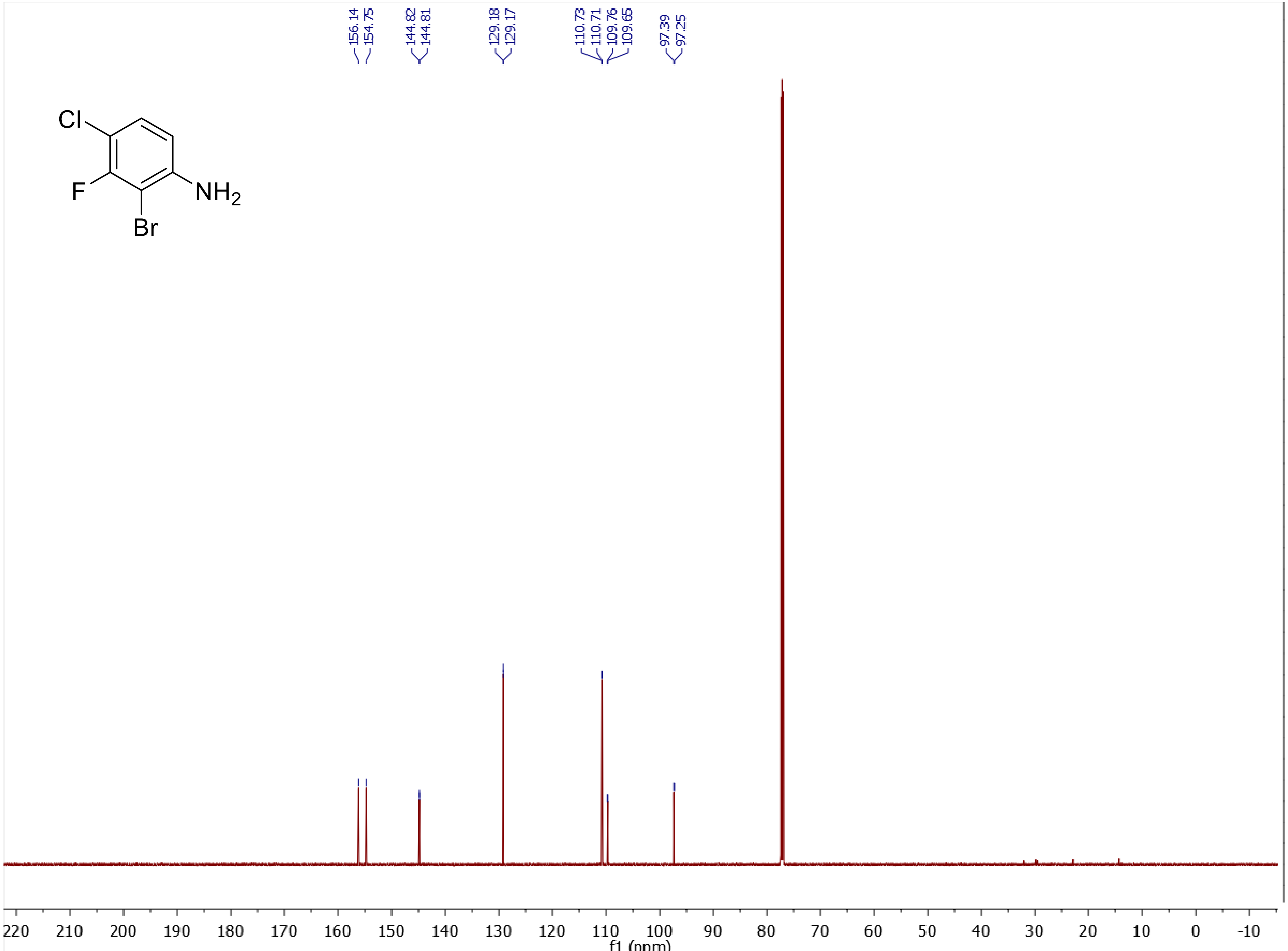

^{1}H-NMR ($CDCl_3$): 2-bromo-4-chloro-3-fluoroaniline

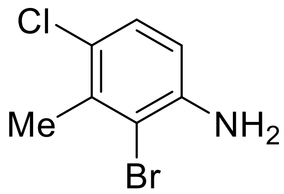

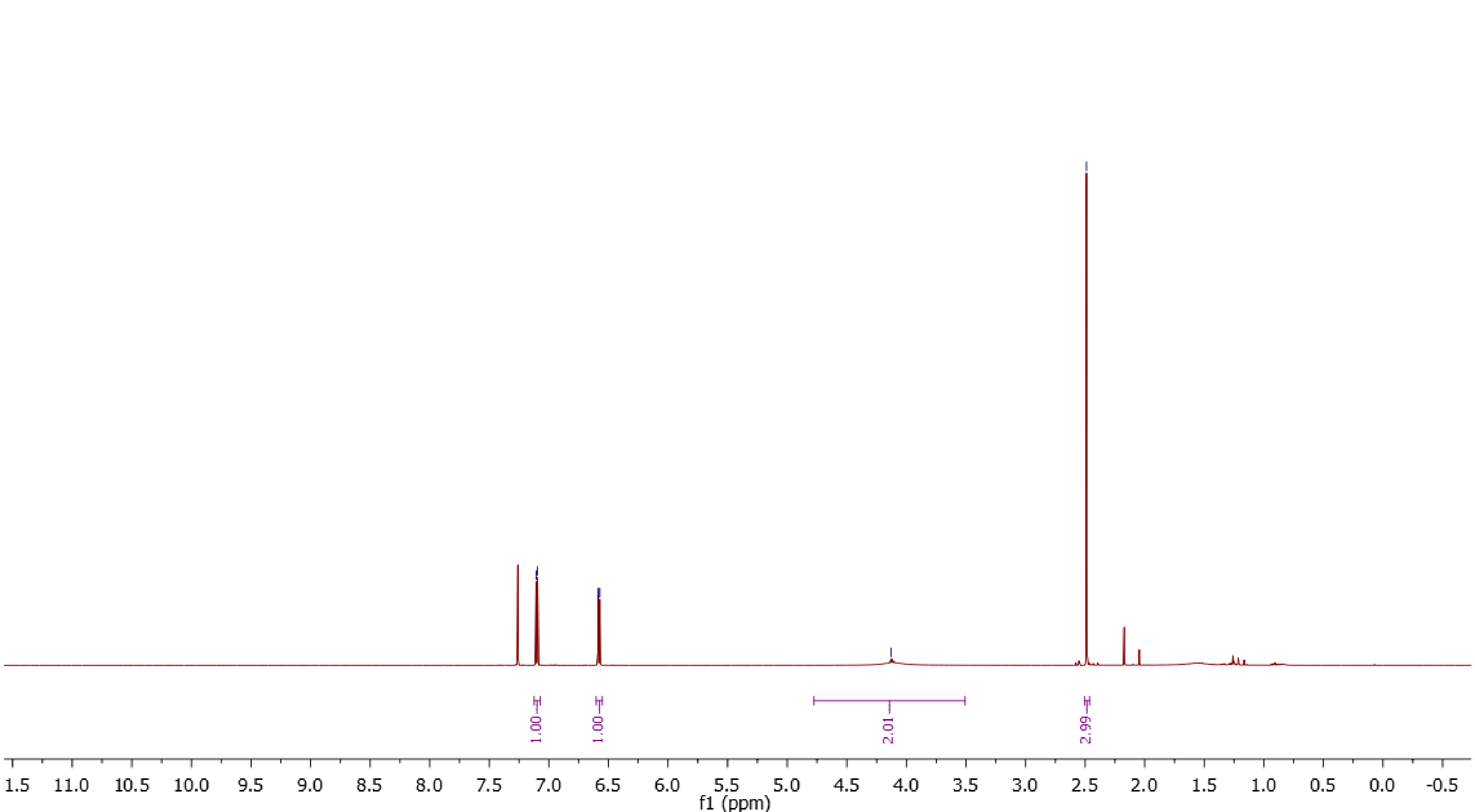

^{13}C-NMR ($CDCl_3$): 2-bromo-4-chloro-3-fluoroaniline

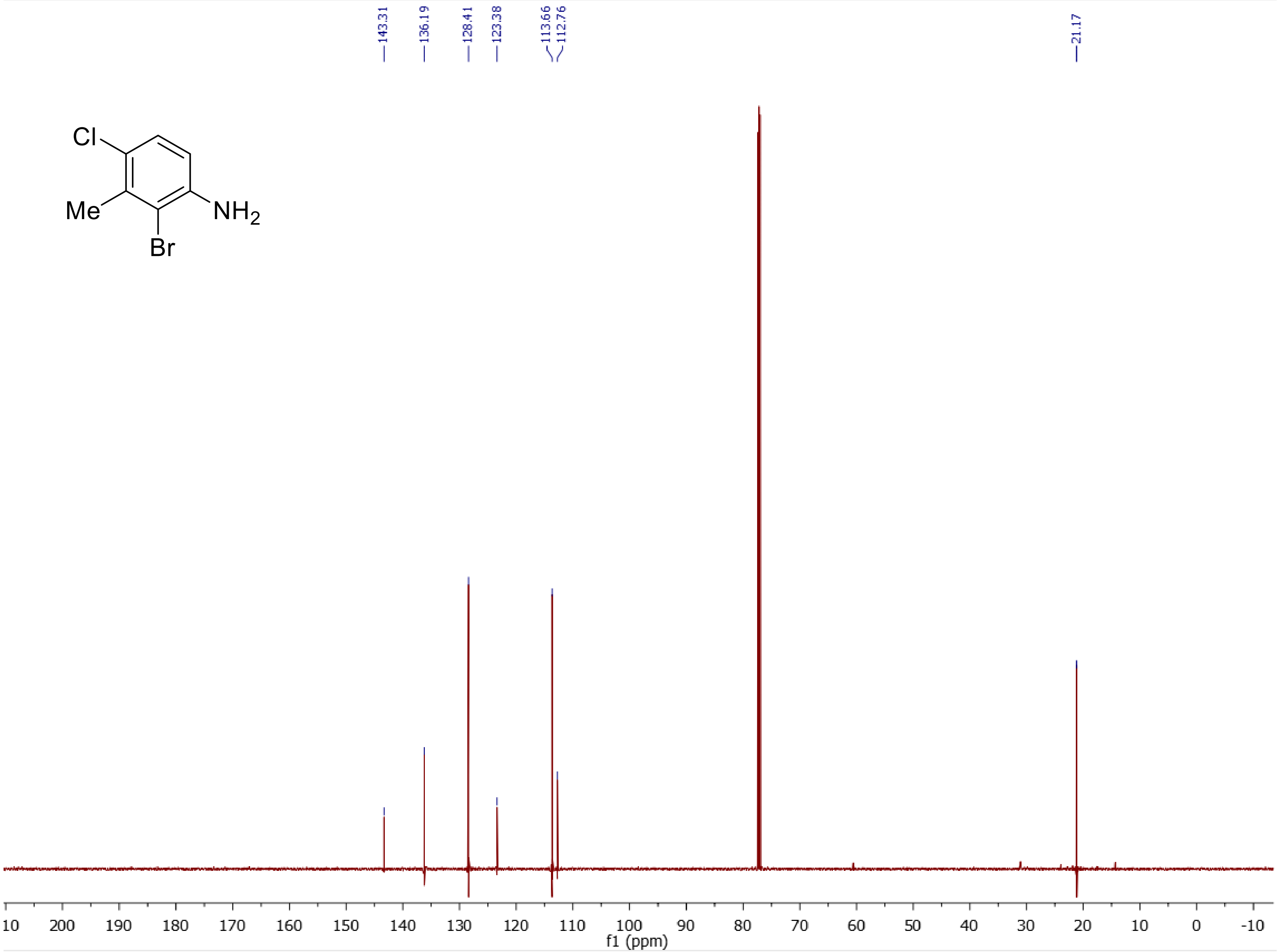

^{1}H-NMR ($CDCl_3$): N-(1-bromo-5,6,7,8-tetrahydronaphthalen-2-yl)pivalamide

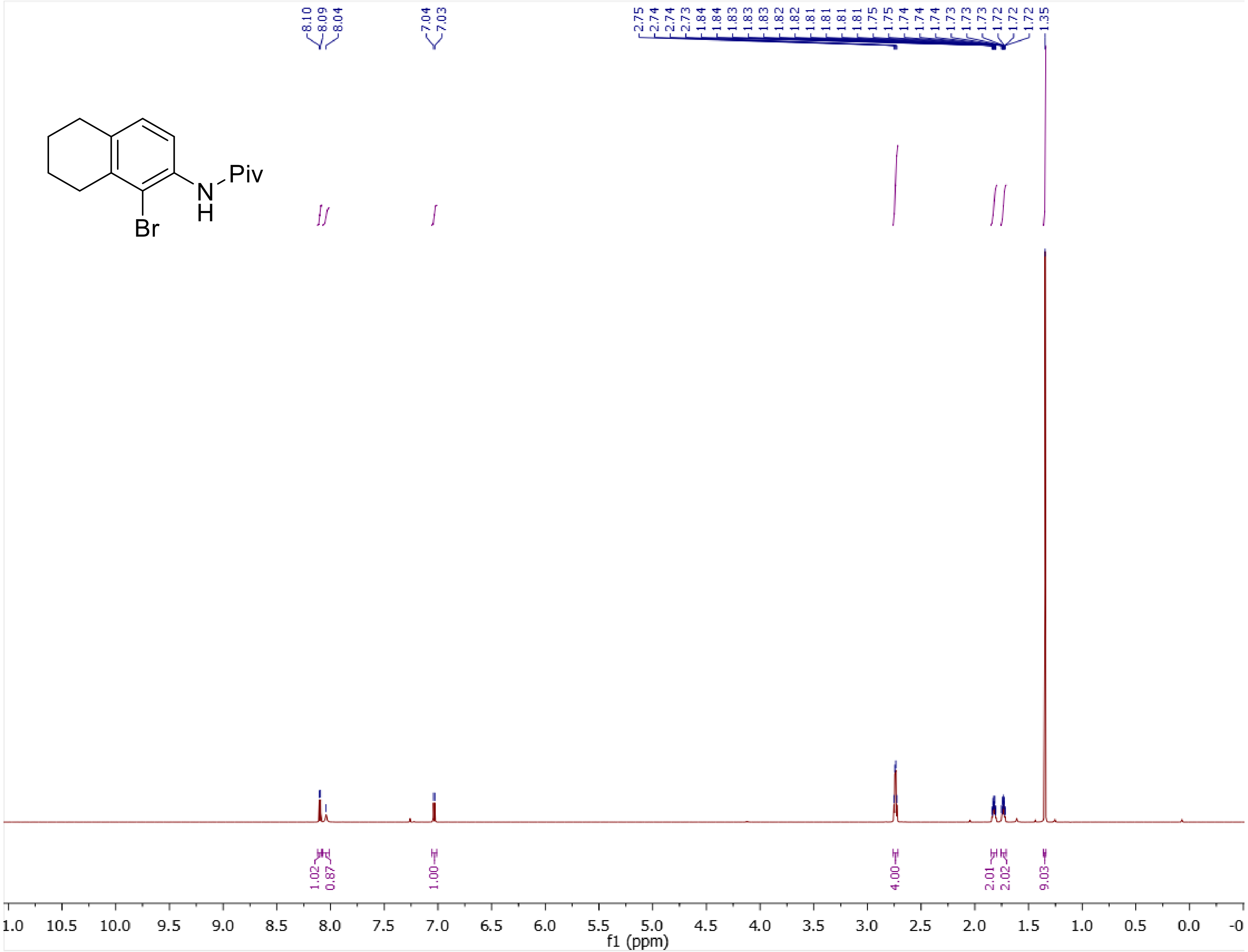

^{13}C-NMR ($CDCl_3$): N-(1-bromo-5,6,7,8-tetrahydronaphthalen-2-yl)pivalamide

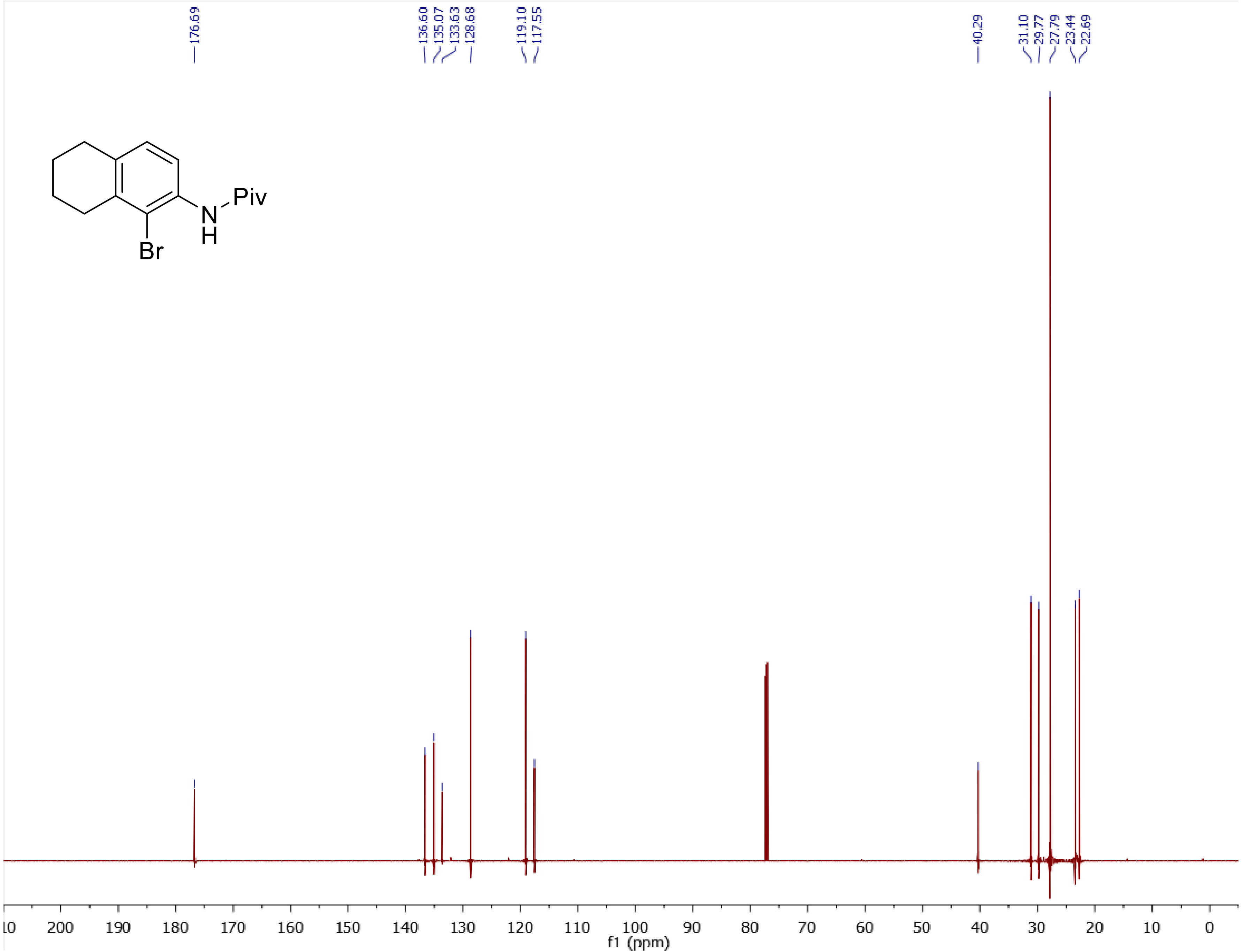

^{1}H-NMR ($CDCl_3$): 1-bromo-5,6,7,8-tetrahydronaphthalen-2-amine

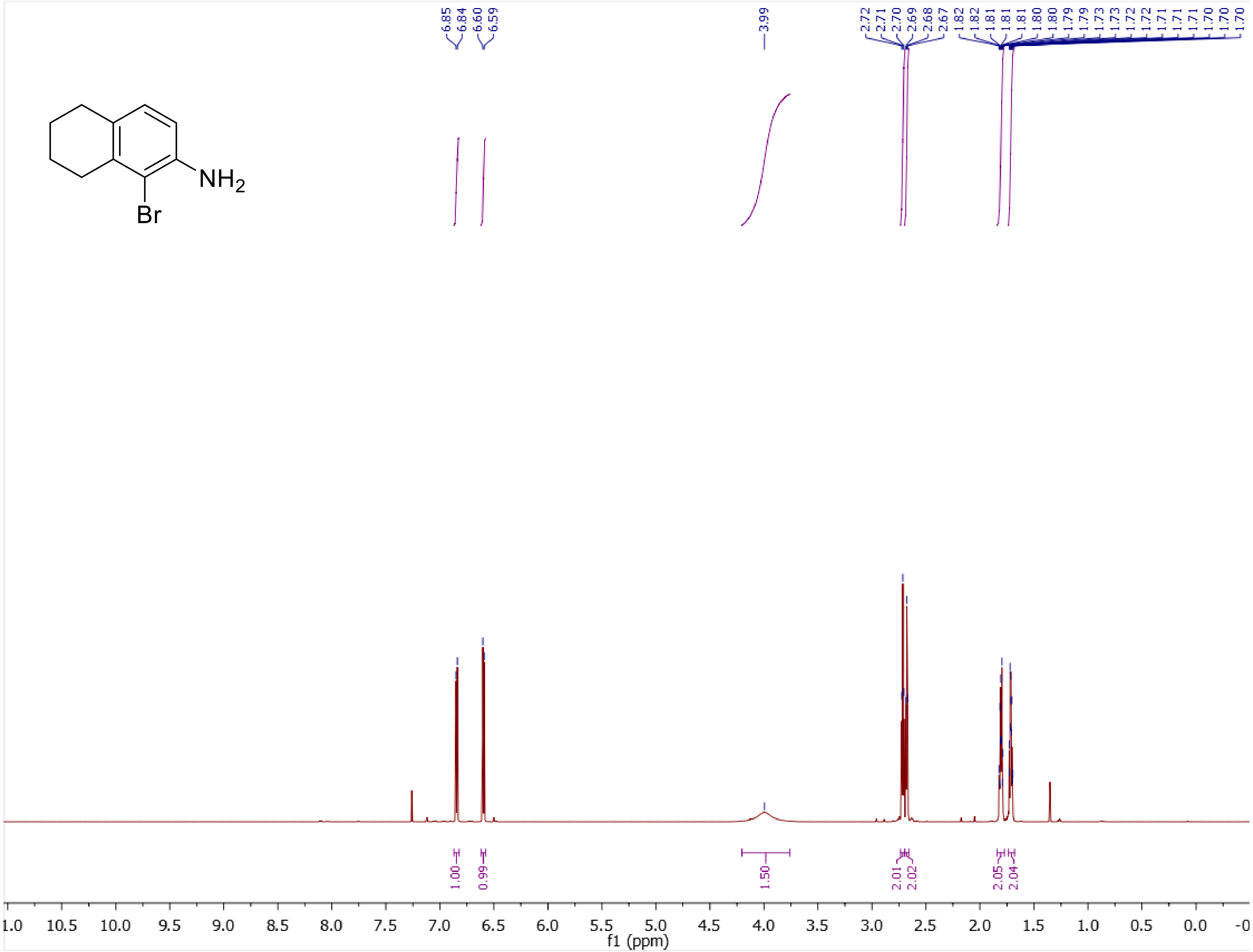

^{13}C-NMR ($CDCl_3$): 1-bromo-5,6,7,8-tetrahydronaphthalen-2-amine

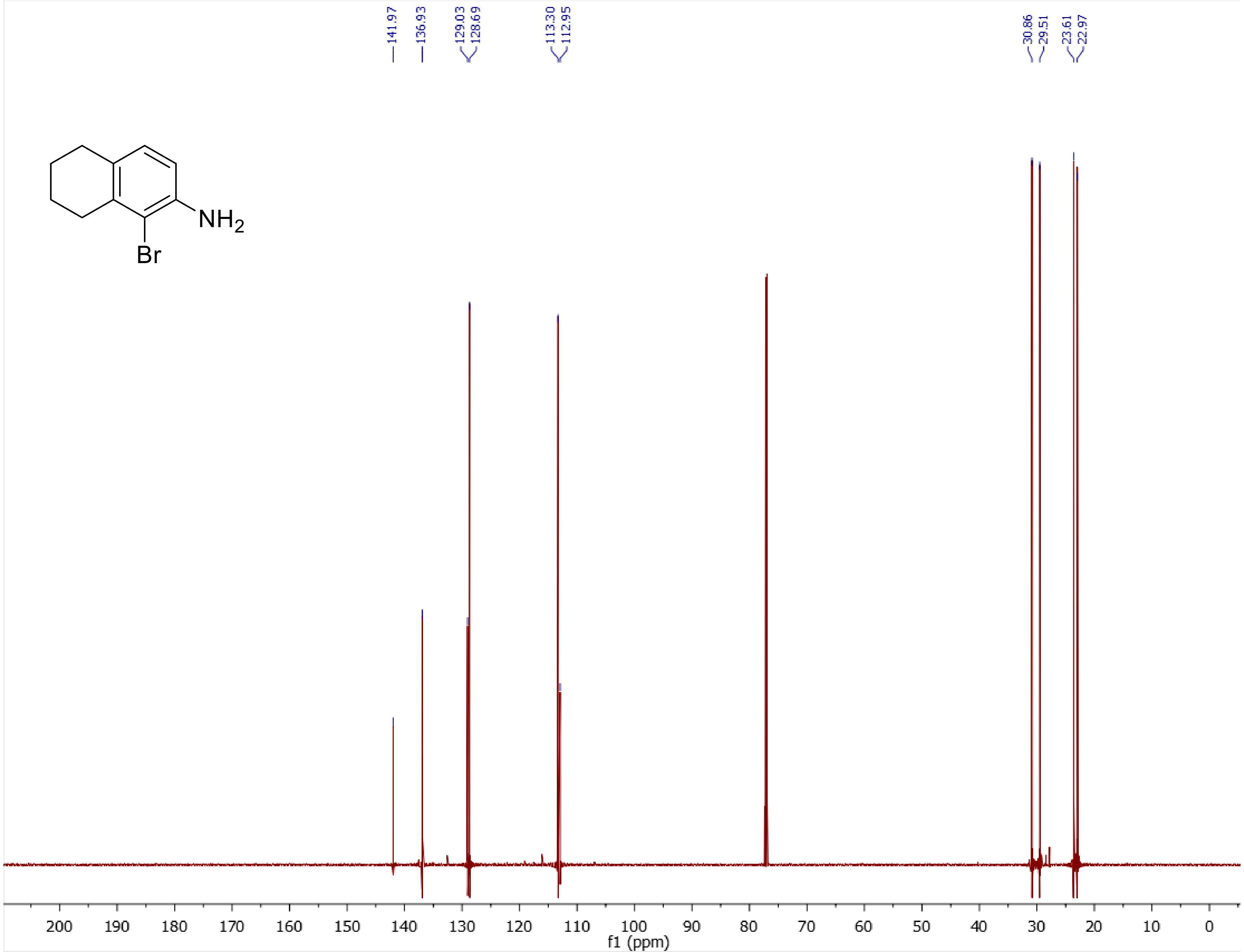

^{1}H-NMR ($CDCl_3$): 2-bromo-3-fluoro-N-methylaniline

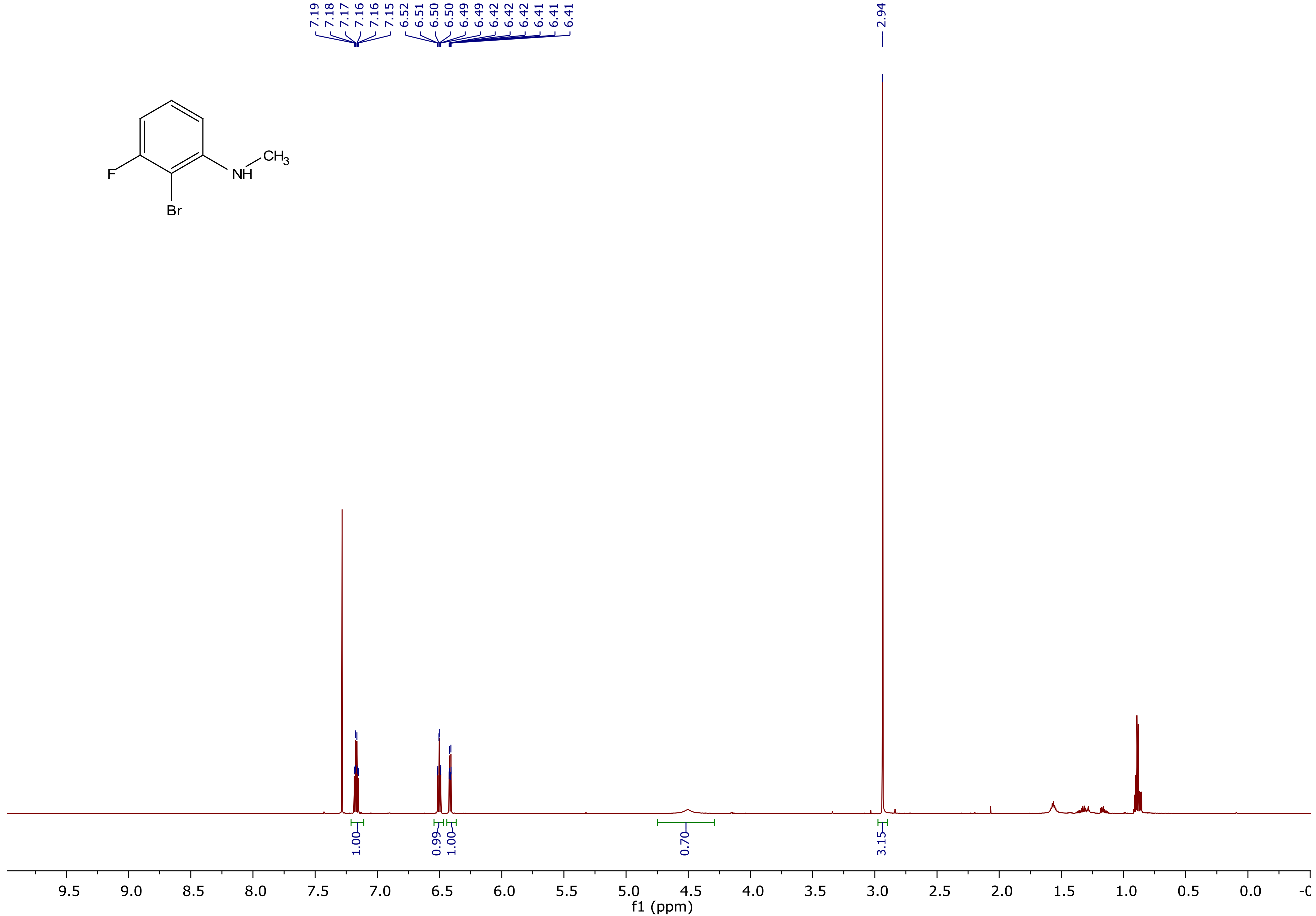

^{19}F-NMR ($CDCl_3$): 2-bromo-3-fluoro-N-methylaniline

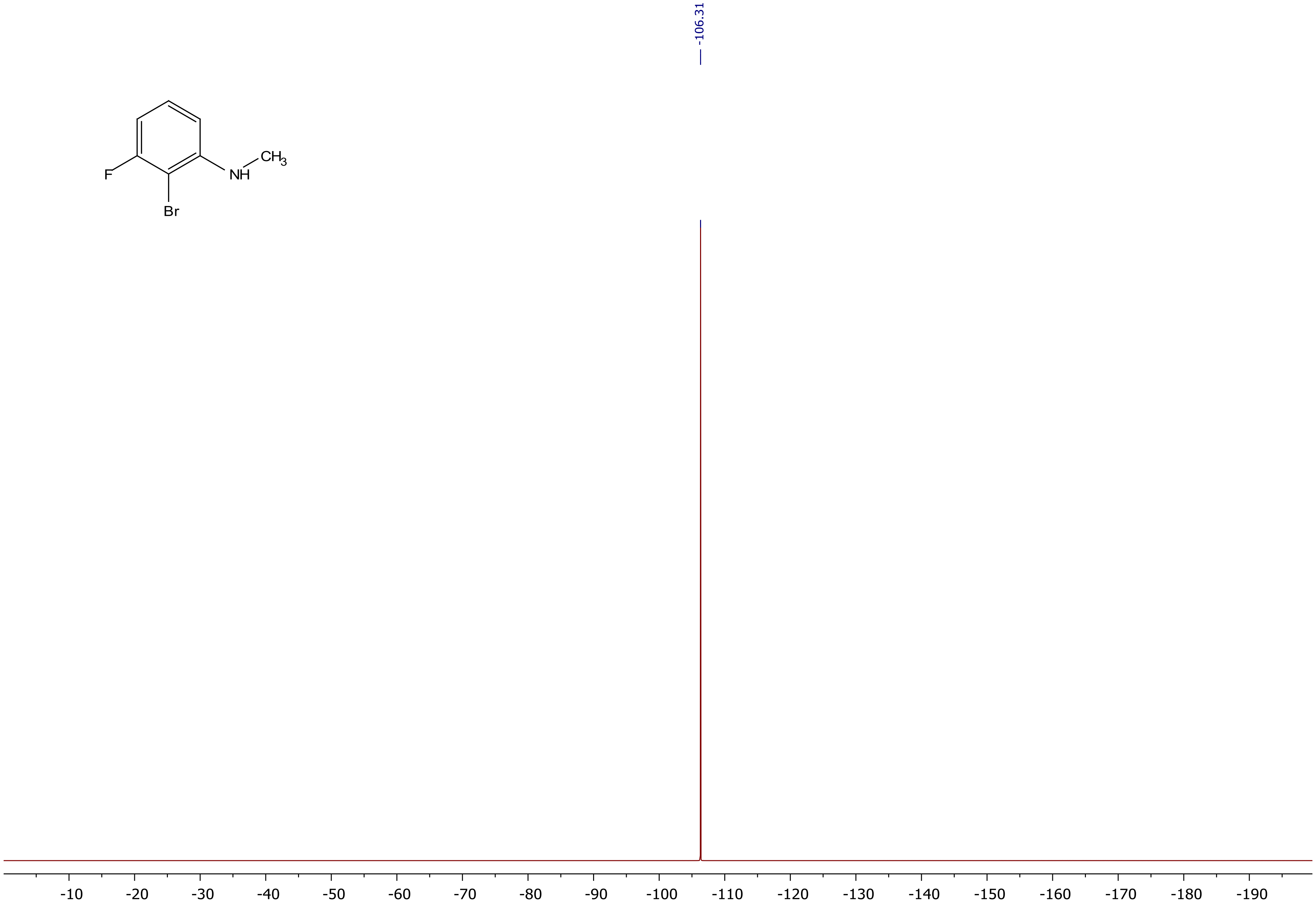

^{13}C-NMR ($CDCl_3$): 2-bromo-3-fluoro-N-methylaniline

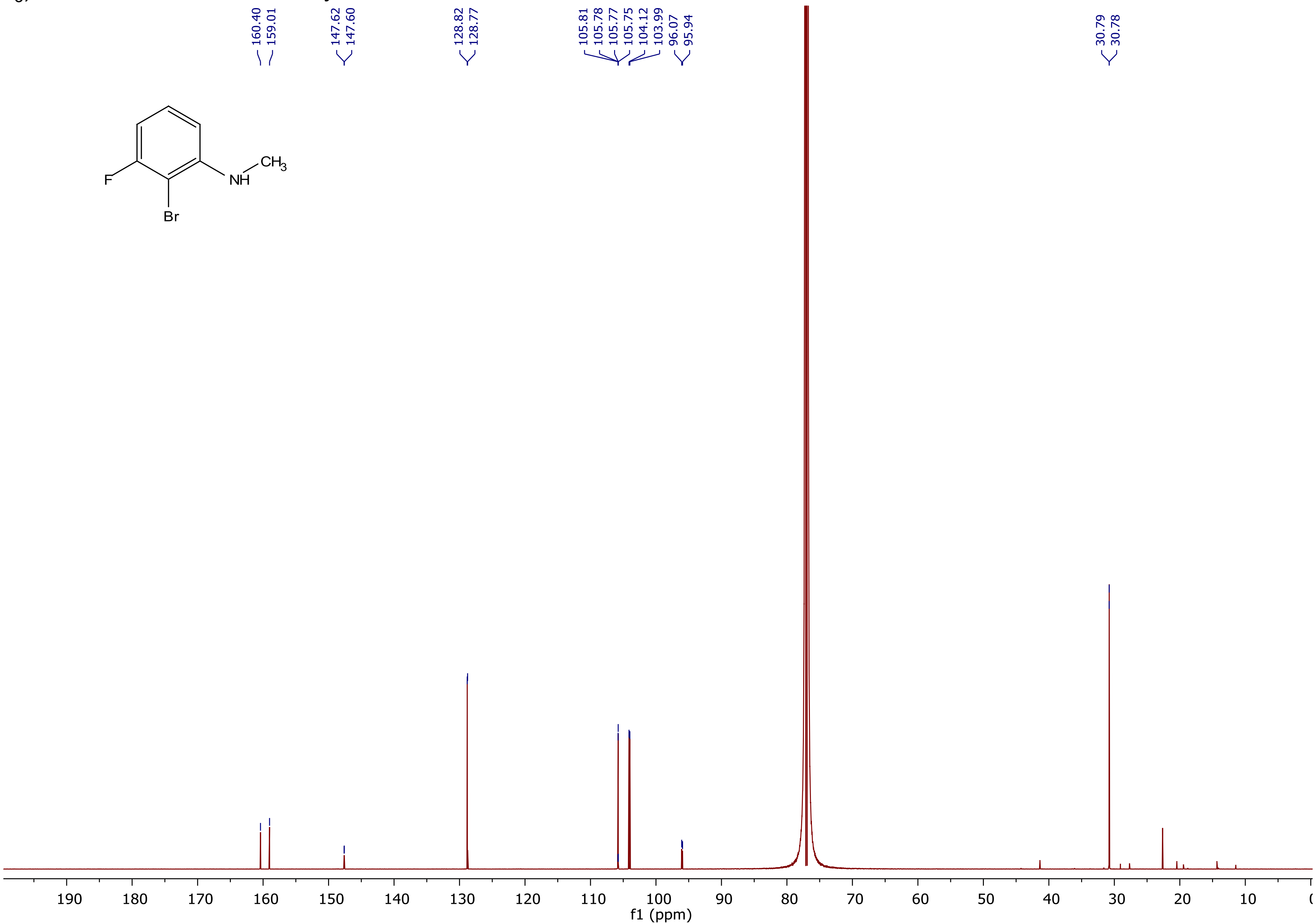

^{1}H-NMR ($CDCl_3$): 2-bromo-3-methyl-N-methylaniline

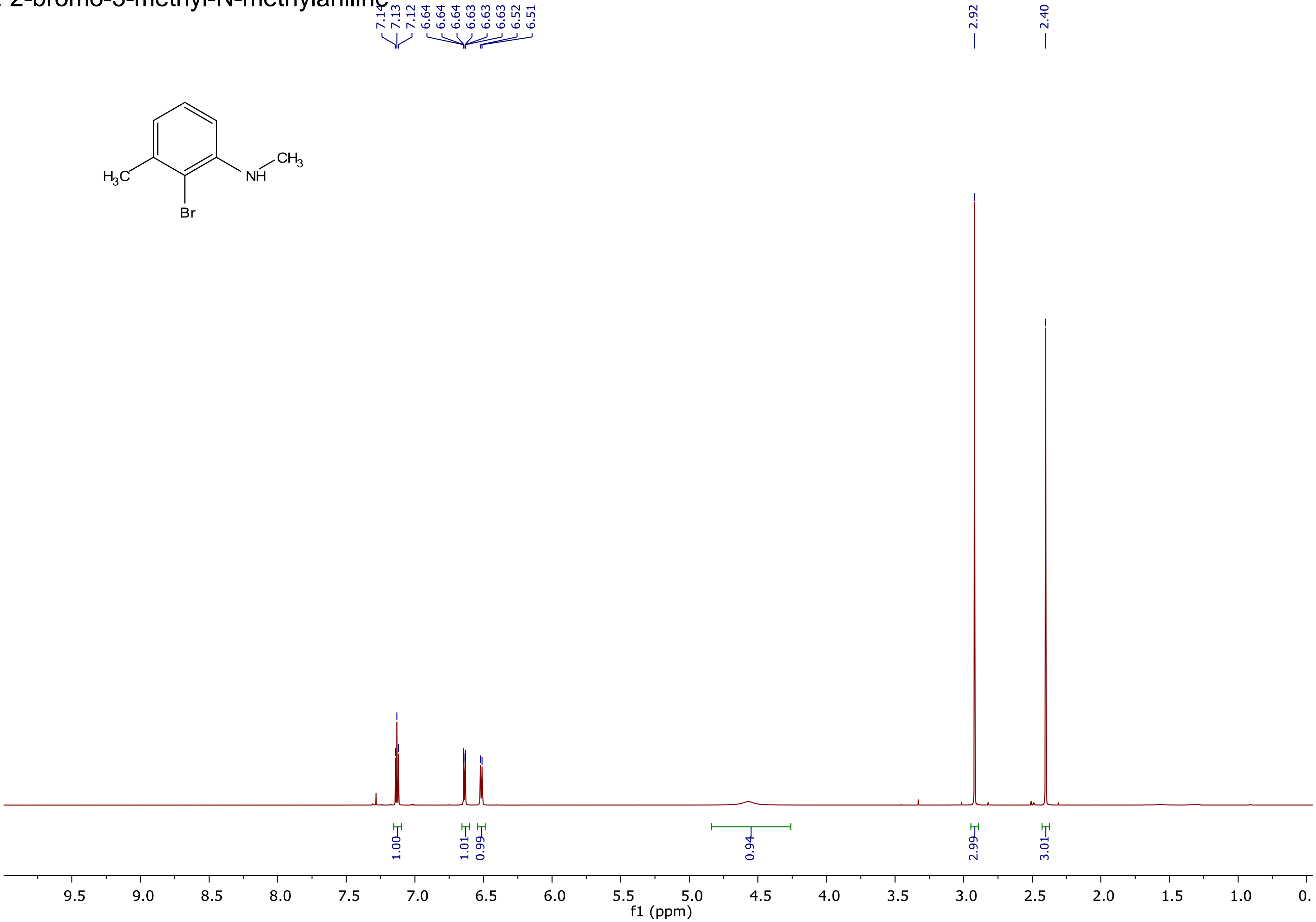

^{13}C-NMR ($CDCl_3$): 2-bromo-3-methyl-N-methylaniline

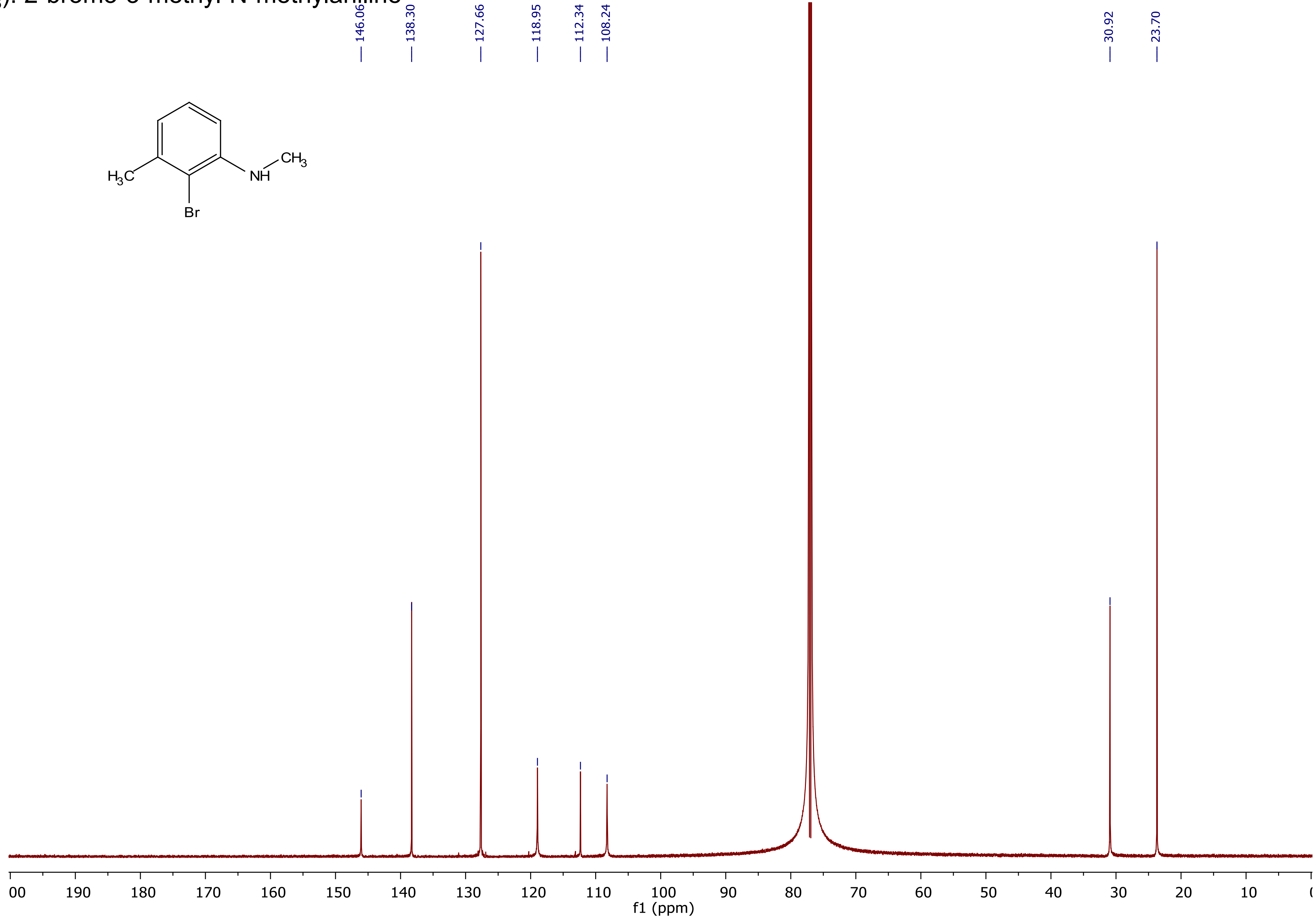

^{1}H-NMR ($CDCl_3$): 2-bromo-N-ethyl-3-fluoroaniline

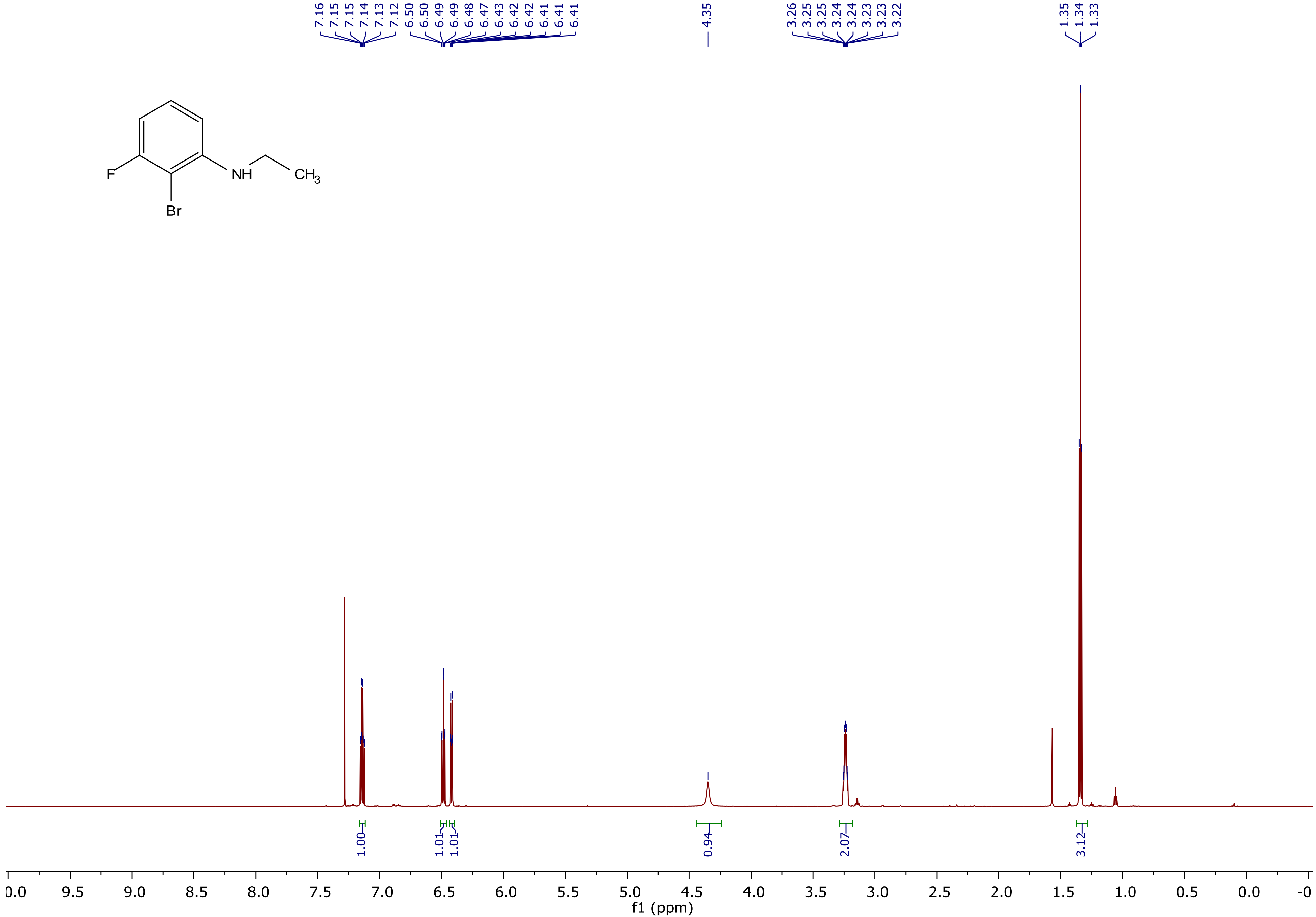

^{19}F-NMR ($CDCl_3$): 2-bromo-N-ethyl-3-fluoroaniline

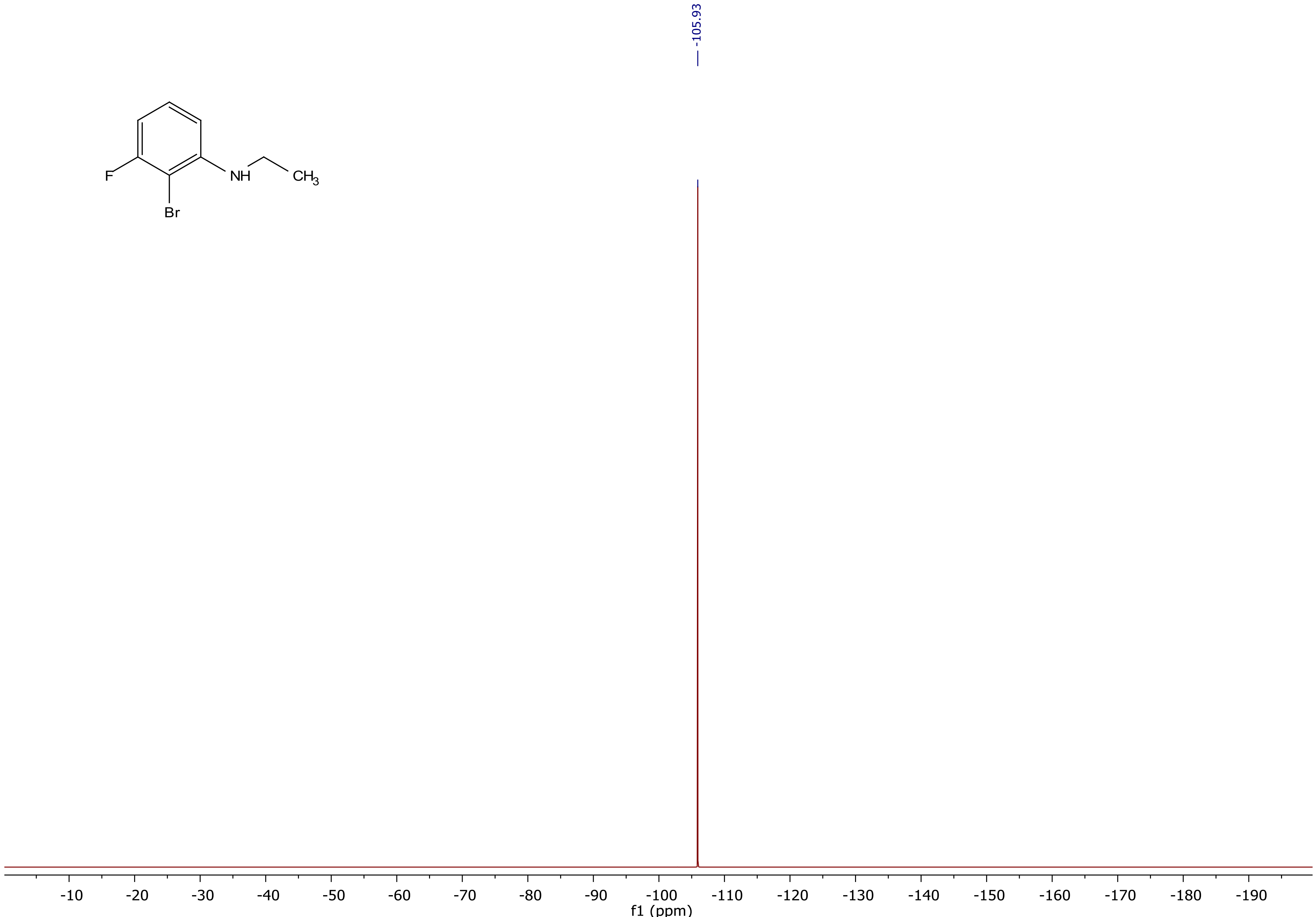

^{13}C-NMR ($CDCl_3$): 2-bromo-N-ethyl-3-fluoroaniline

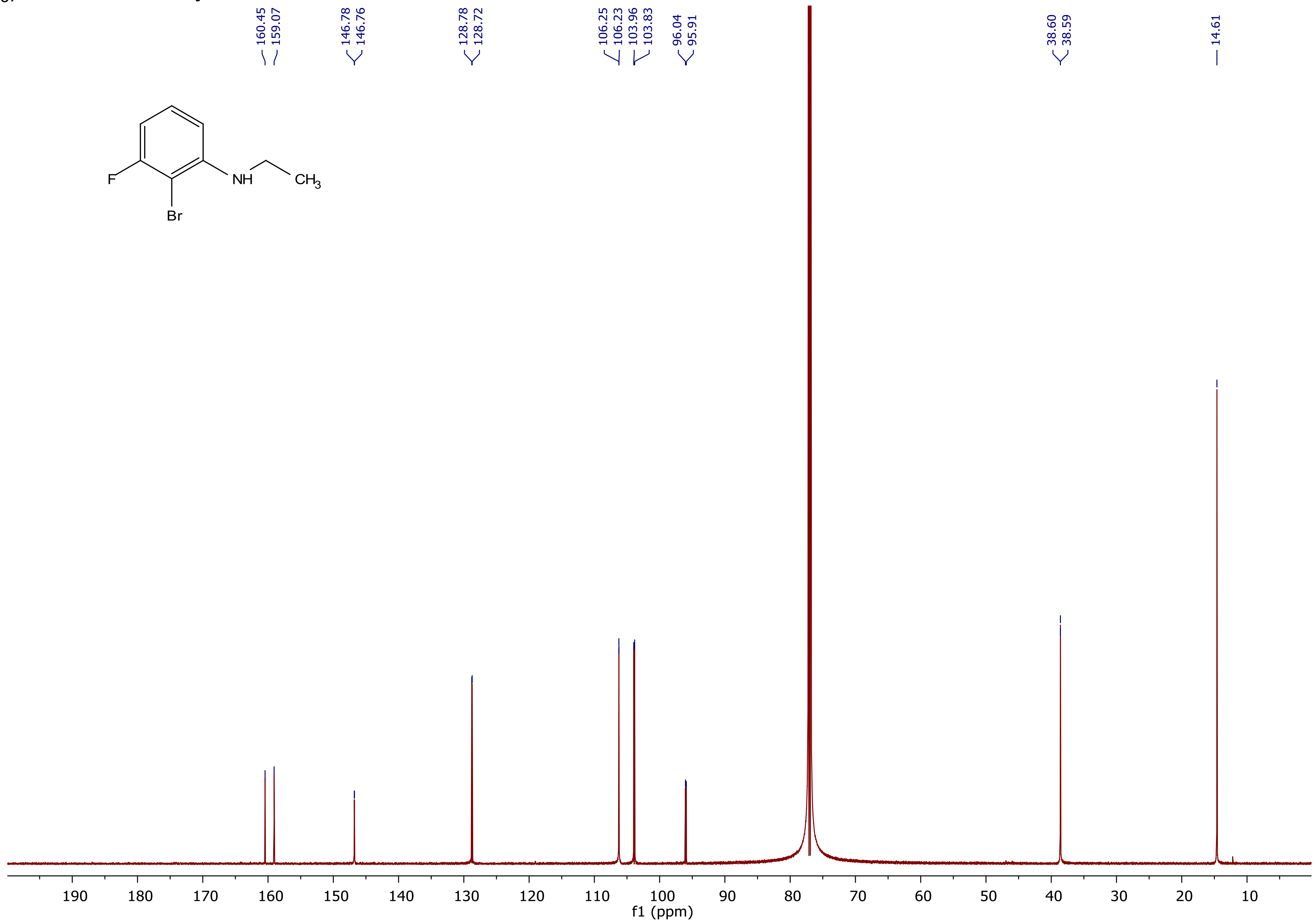

^{1}H-NMR ($CDCl_3$): 2-bromo-N-ethyl-3-methylaniline

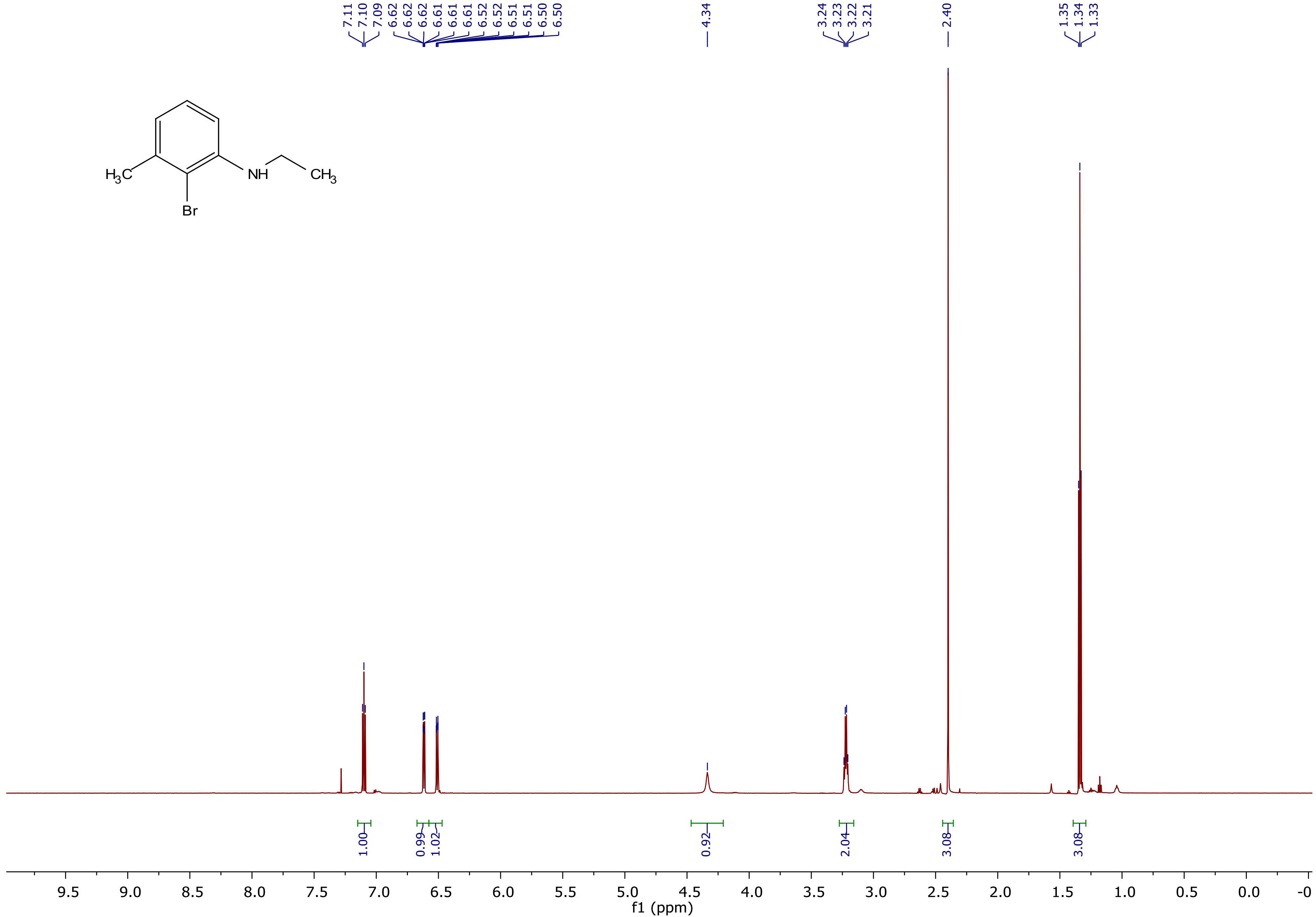

^{13}C-NMR ($CDCl_3$): 2-bromo-N-ethyl-3-methylaniline

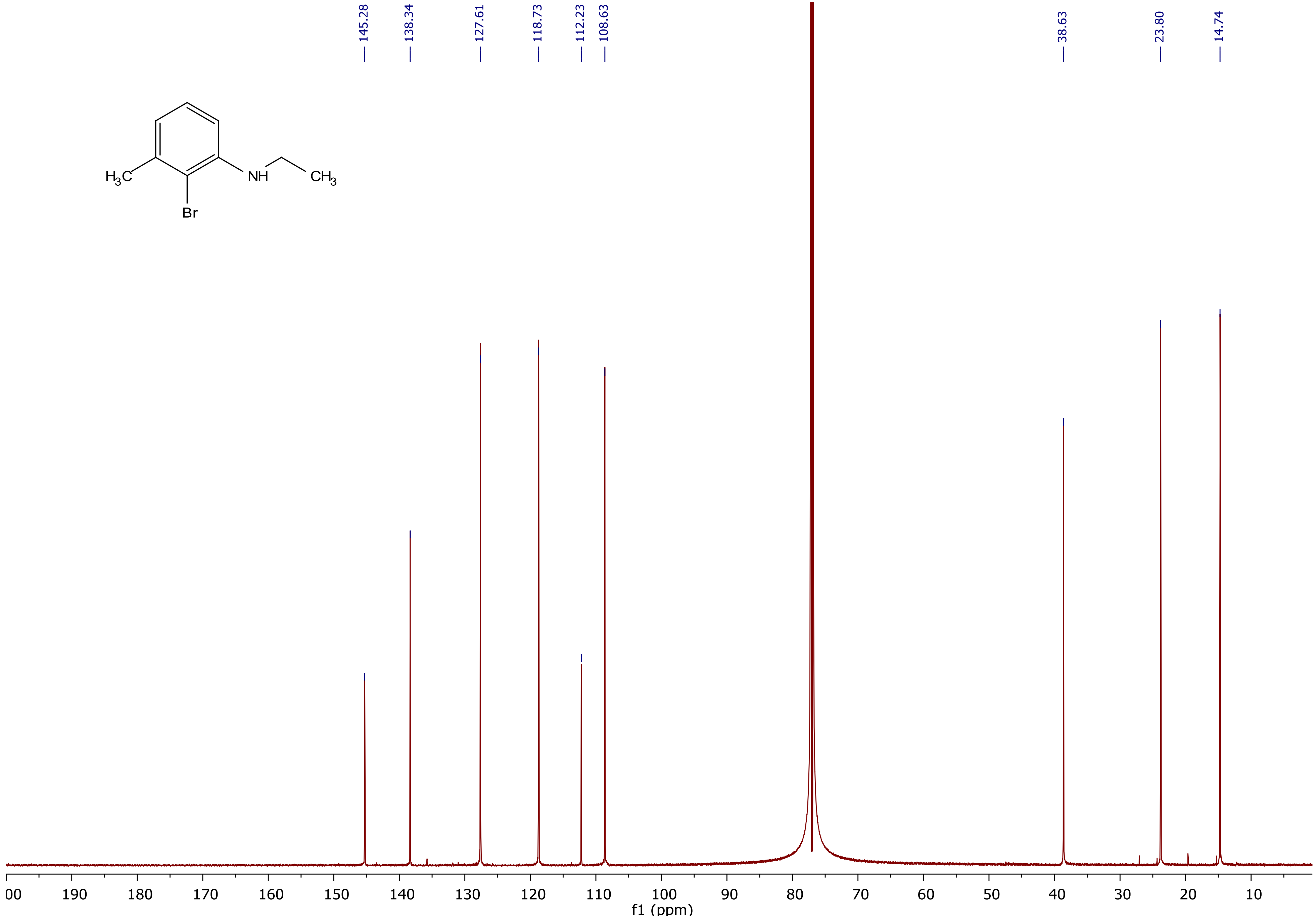

¹H-NMR ($CDCl_3$): N-benzyl-2-bromo-3-fluoroaniline

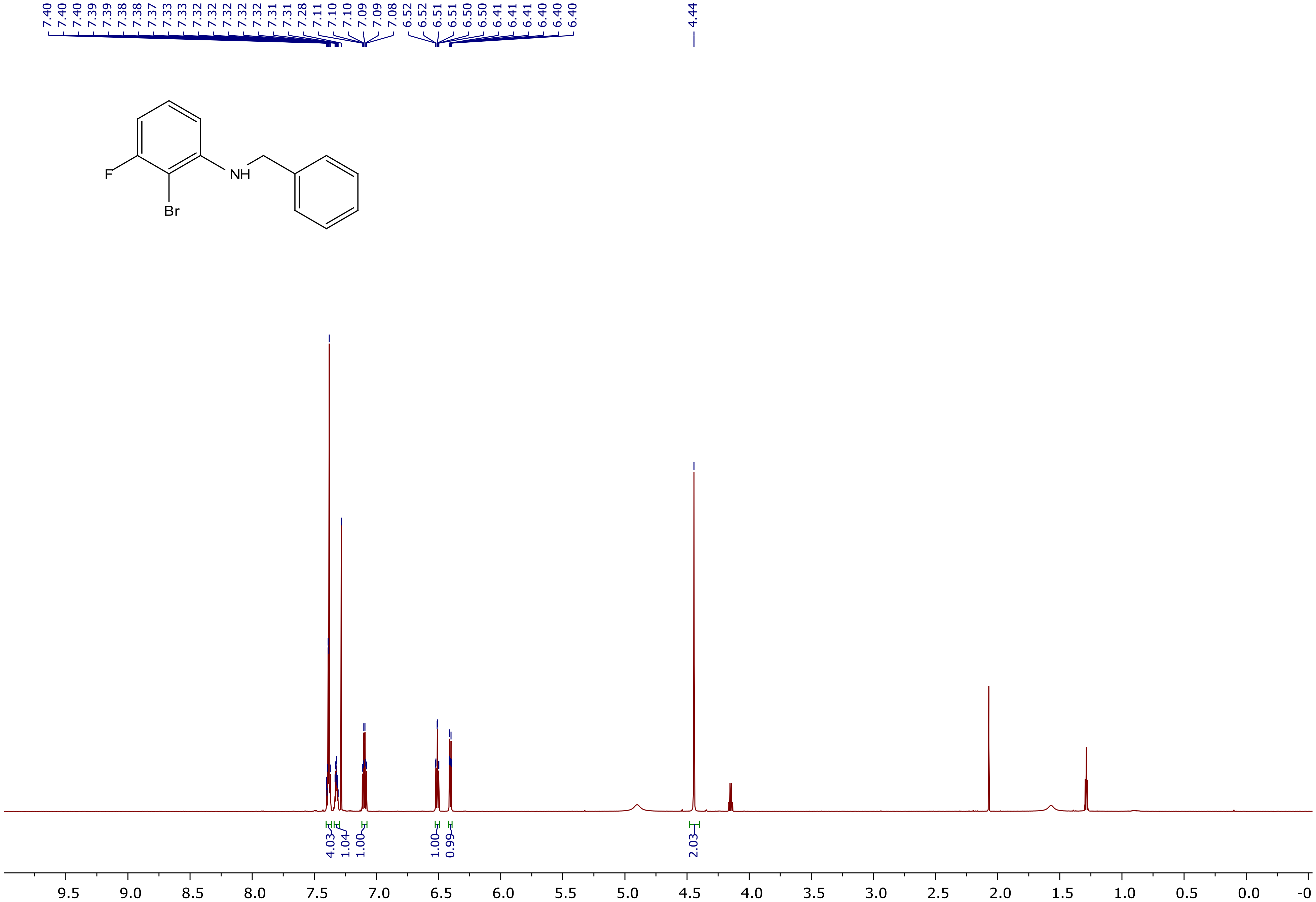

^{19}F-NMR ($CDCl_3$): N-benzyl-2-bromo-3-fluoroaniline

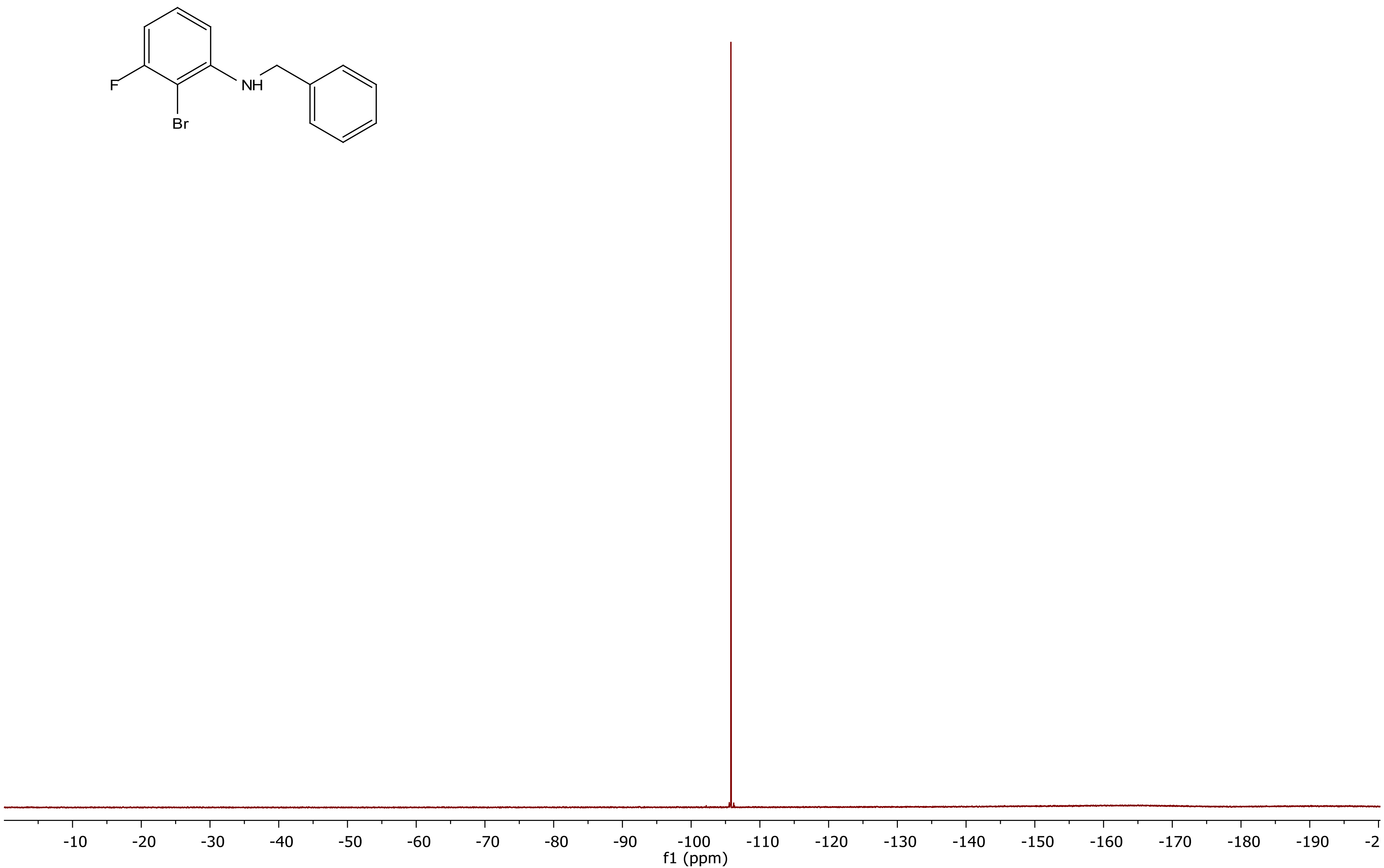

^{13}C-NMR ($CDCl_3$): N-benzyl-2-bromo-3-fluoroaniline

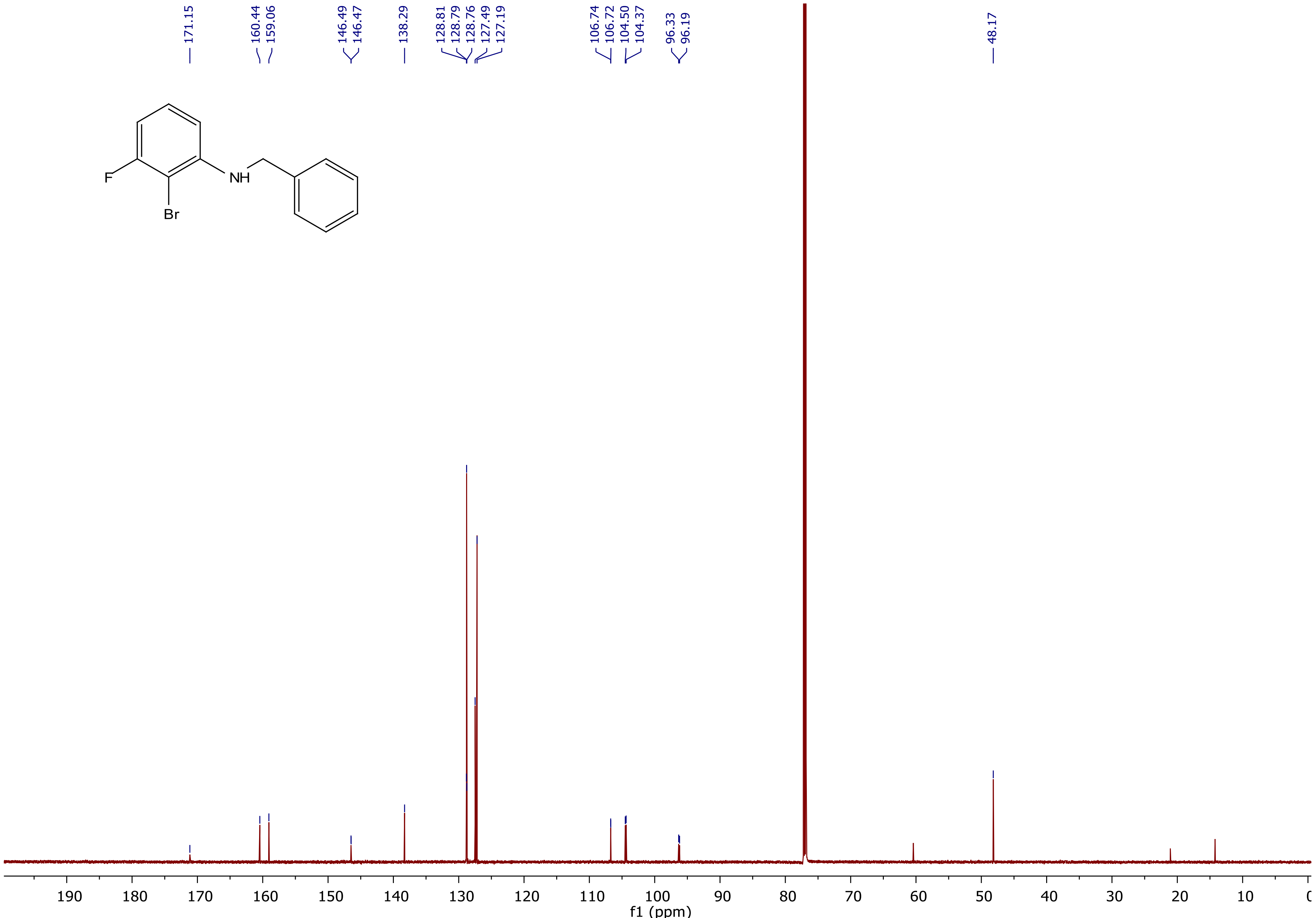

^{1}H-NMR ($CDCl_3$): N-(2-bromo-3-methylphenyl)-4-methylbenzenesulfonamide

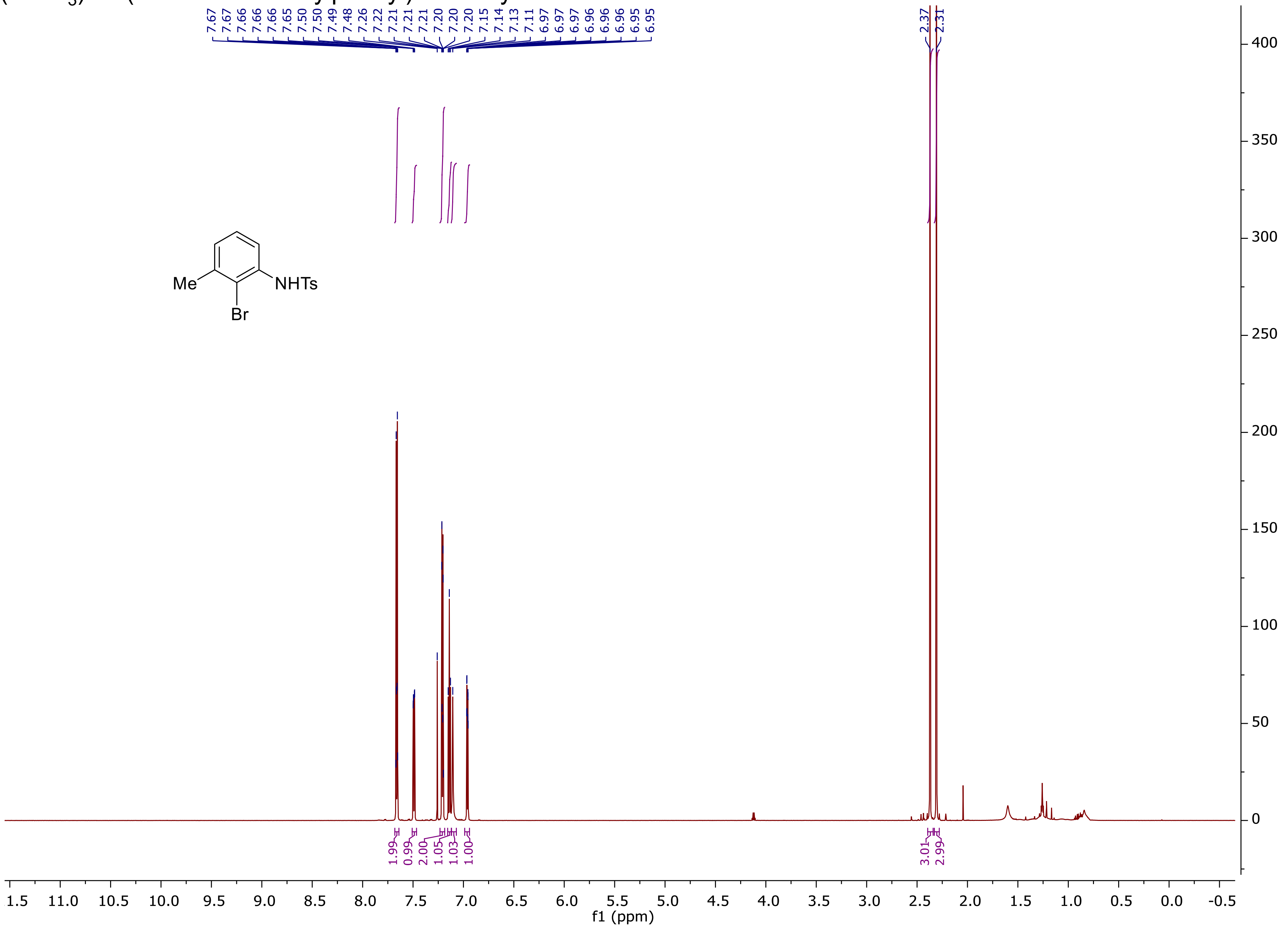

^{13}C-NMR ($CDCl_3$): N-(2-bromo-3-methylphenyl)-4-methylbenzenesulfonamide

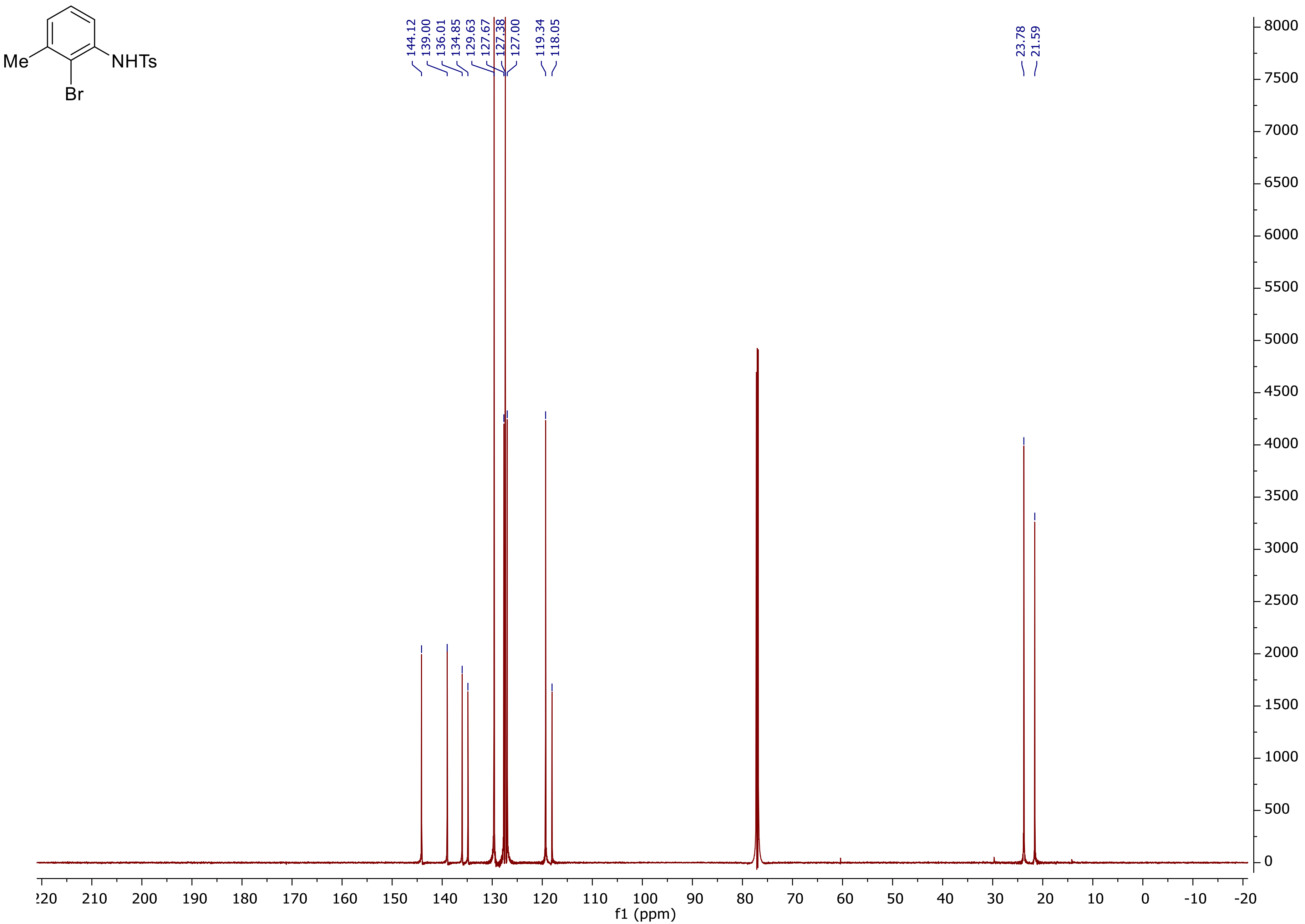

¹H-NMR ($CDCl_3$): N-(2-bromo-3-methylphenyl)-2,2,2-trifluoroacetamide

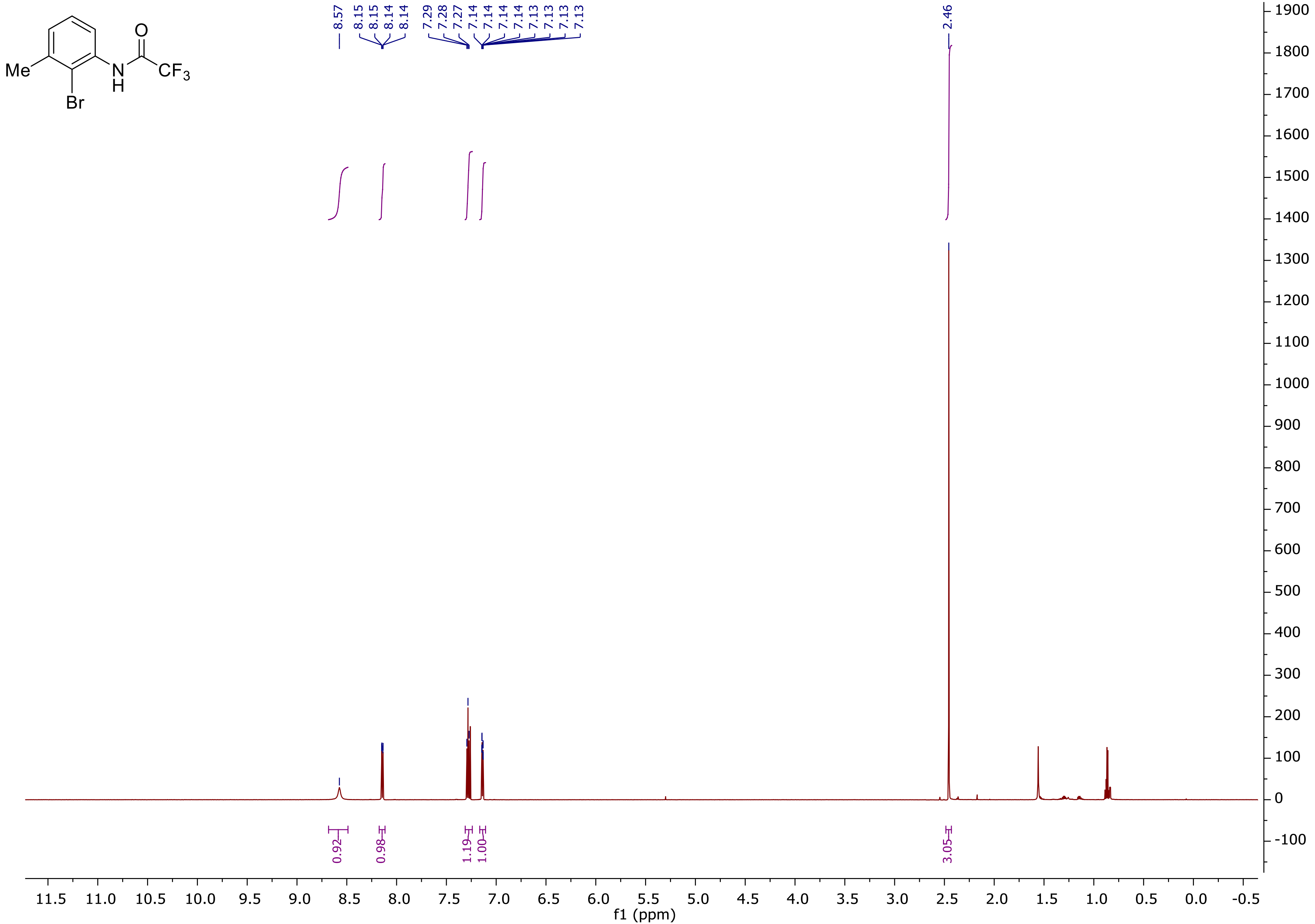

^{19}F-NMR ($CDCl_3$): N-(2-bromo-3-methylphenyl)-2,2,2-trifluoroacetamide

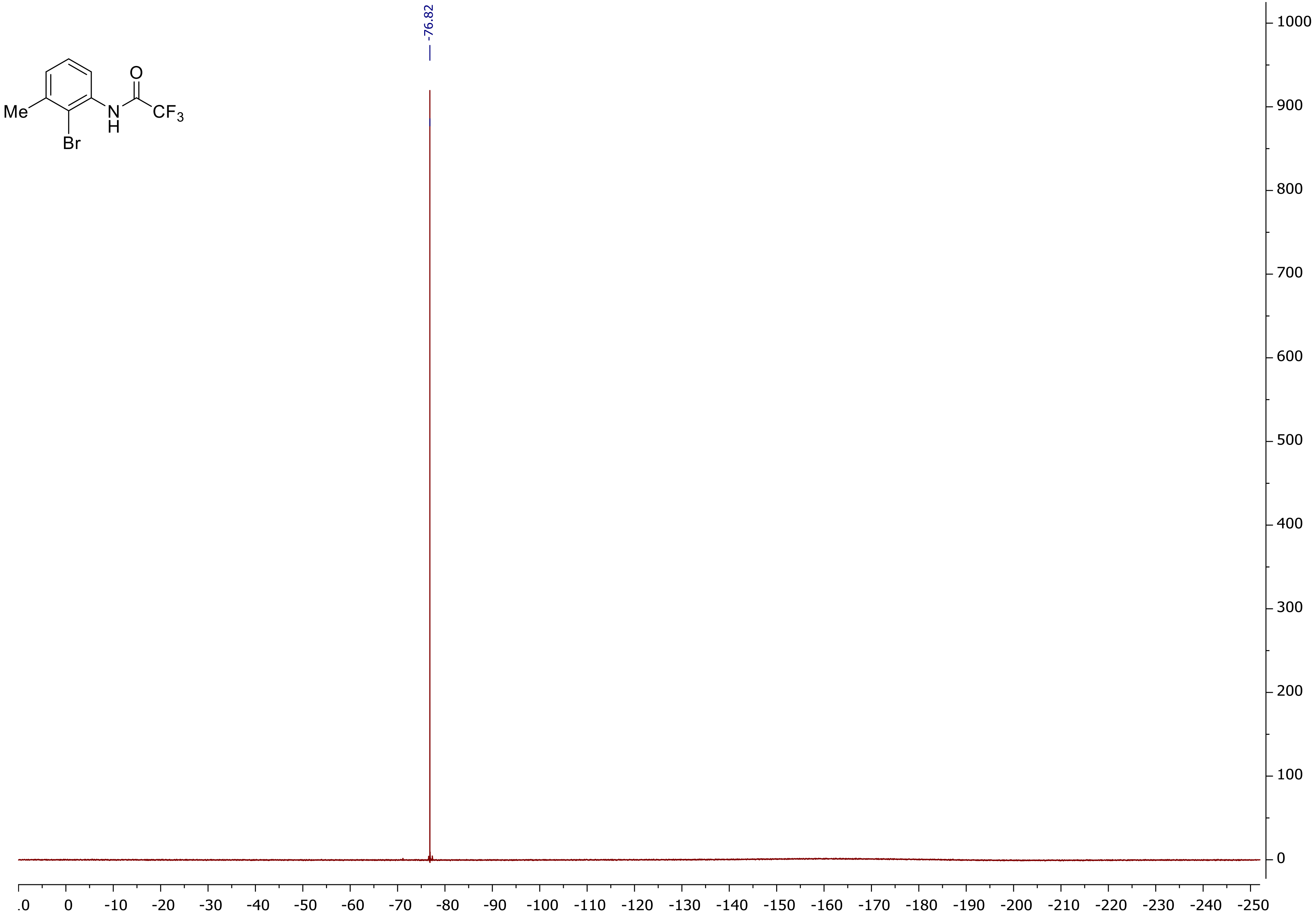

^{13}C-NMR ($CDCl_3$): N-(2-bromo-3-methylphenyl)-2,2,2-trifluoroacetamide

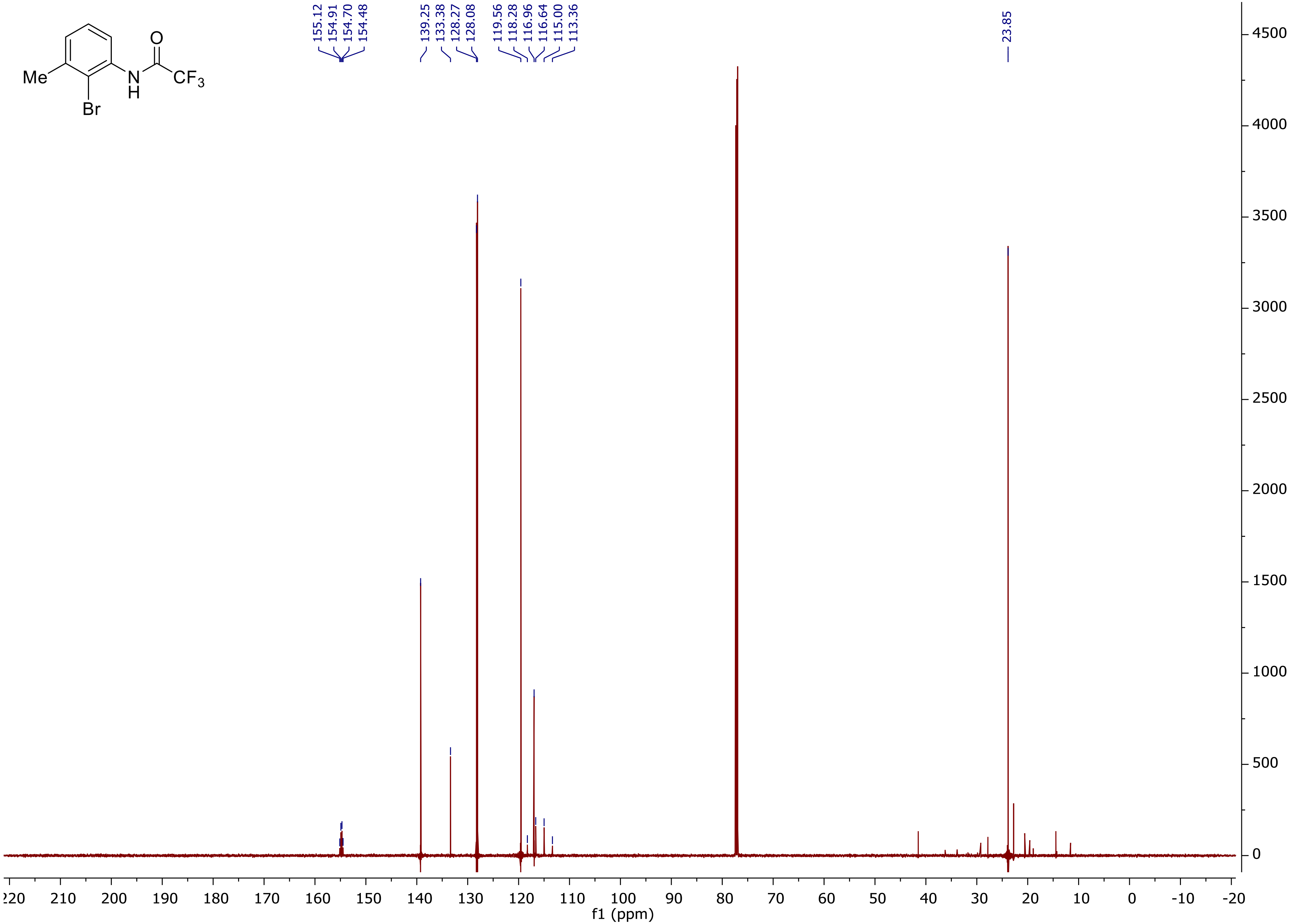

^{1}H-NMR ($CDCl_3$): 1-bromo-5,6,7,8-tetrahydronaphthalen-2-ol

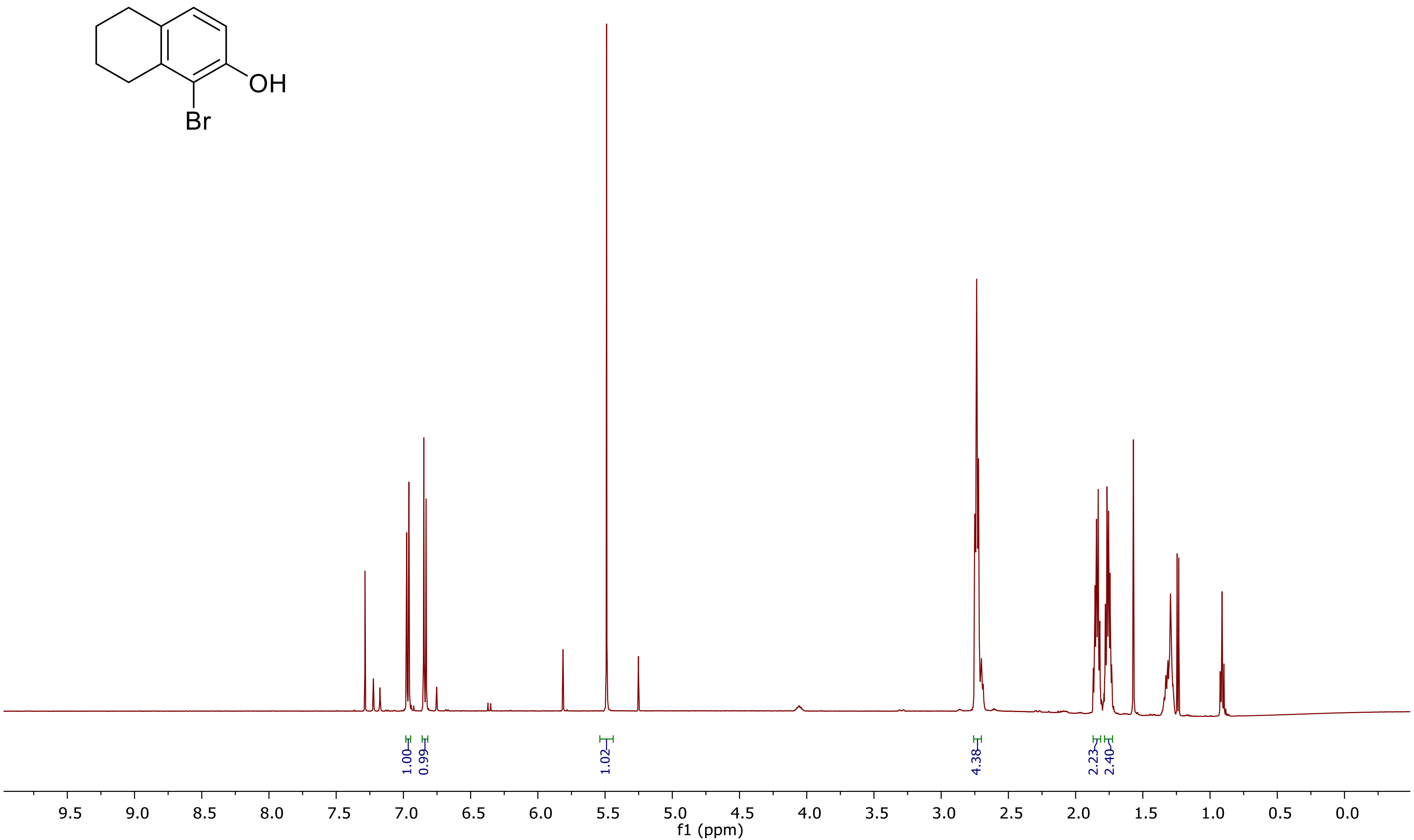

^{13}C-NMR ($CDCl_3$): 1-bromo-5,6,7,8-tetrahydronaphthalen-2-ol

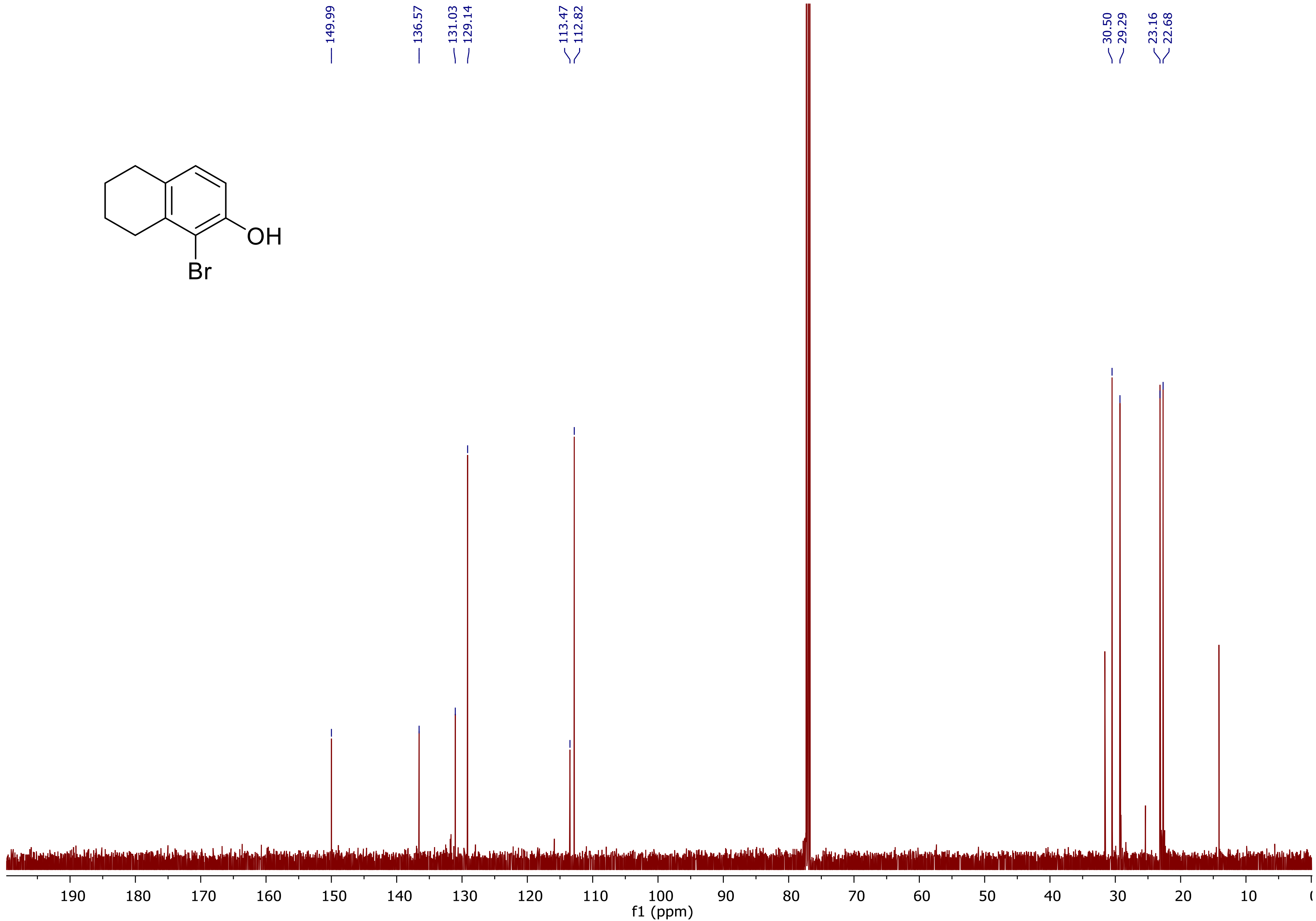

^{1}H-NMR ($CDCl_3$): 5-bromo-6-methoxy-1,2,3,4-tetrahydronaphthalene

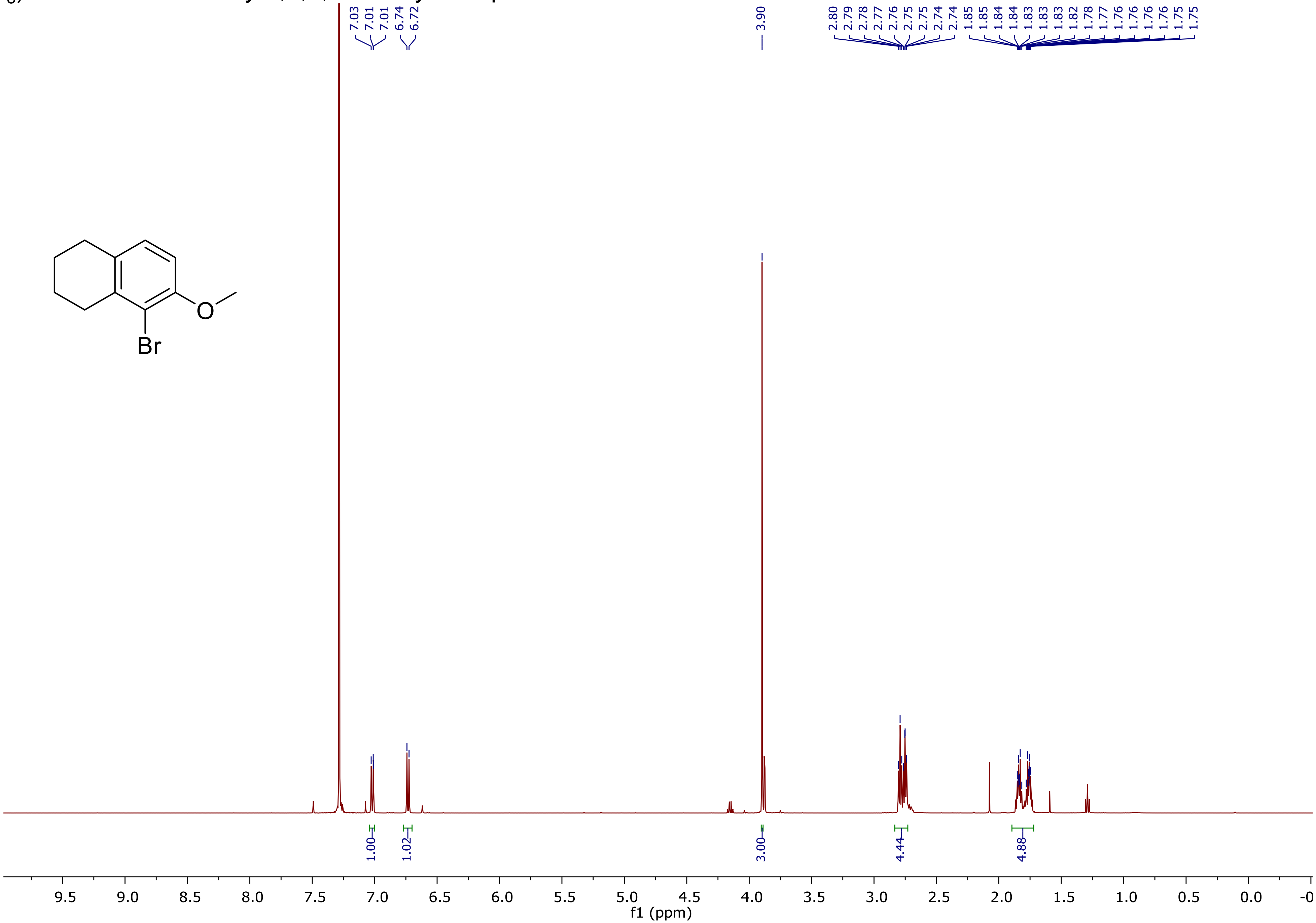

^{13}C-NMR ($CDCl_3$): 5-bromo-6-methoxy-1,2,3,4-tetrahydronaphthalene

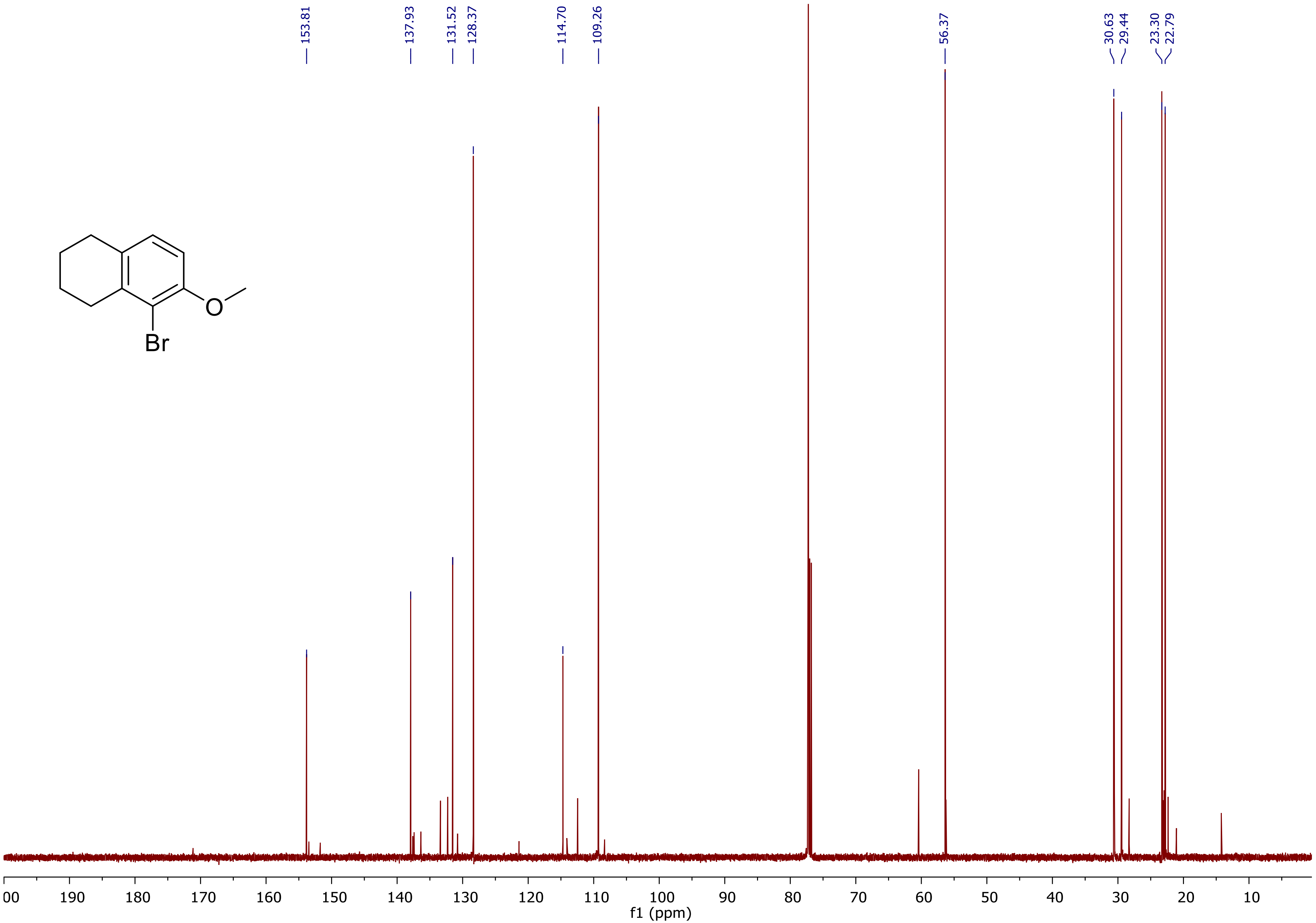

¹H-NMR ($CDCl_3$): 2-bromo-4-chloro-3-methylphenol

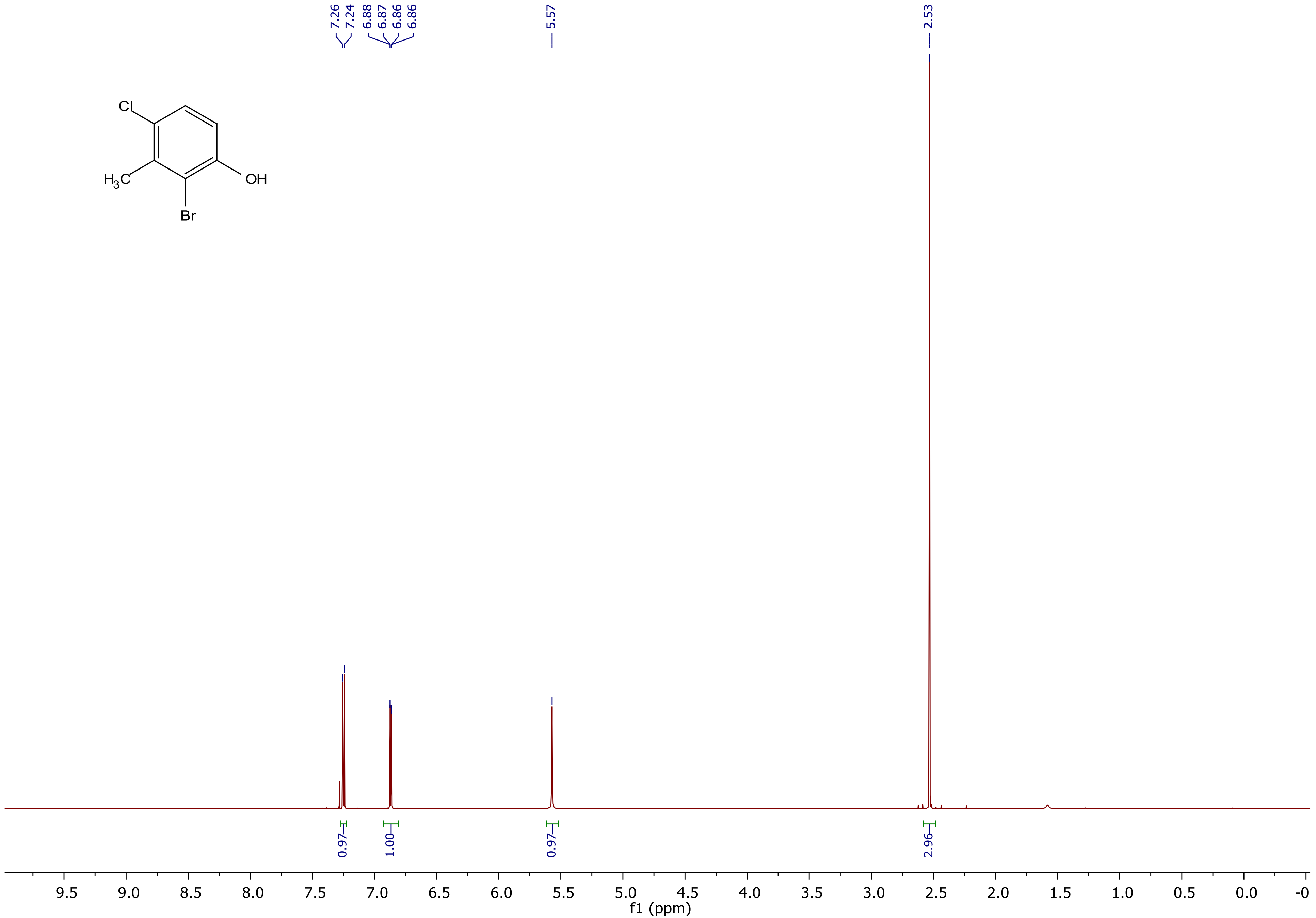

^{13}C-NMR ($CDCl_3$): 2-bromo-4-chloro-3-methylphenol

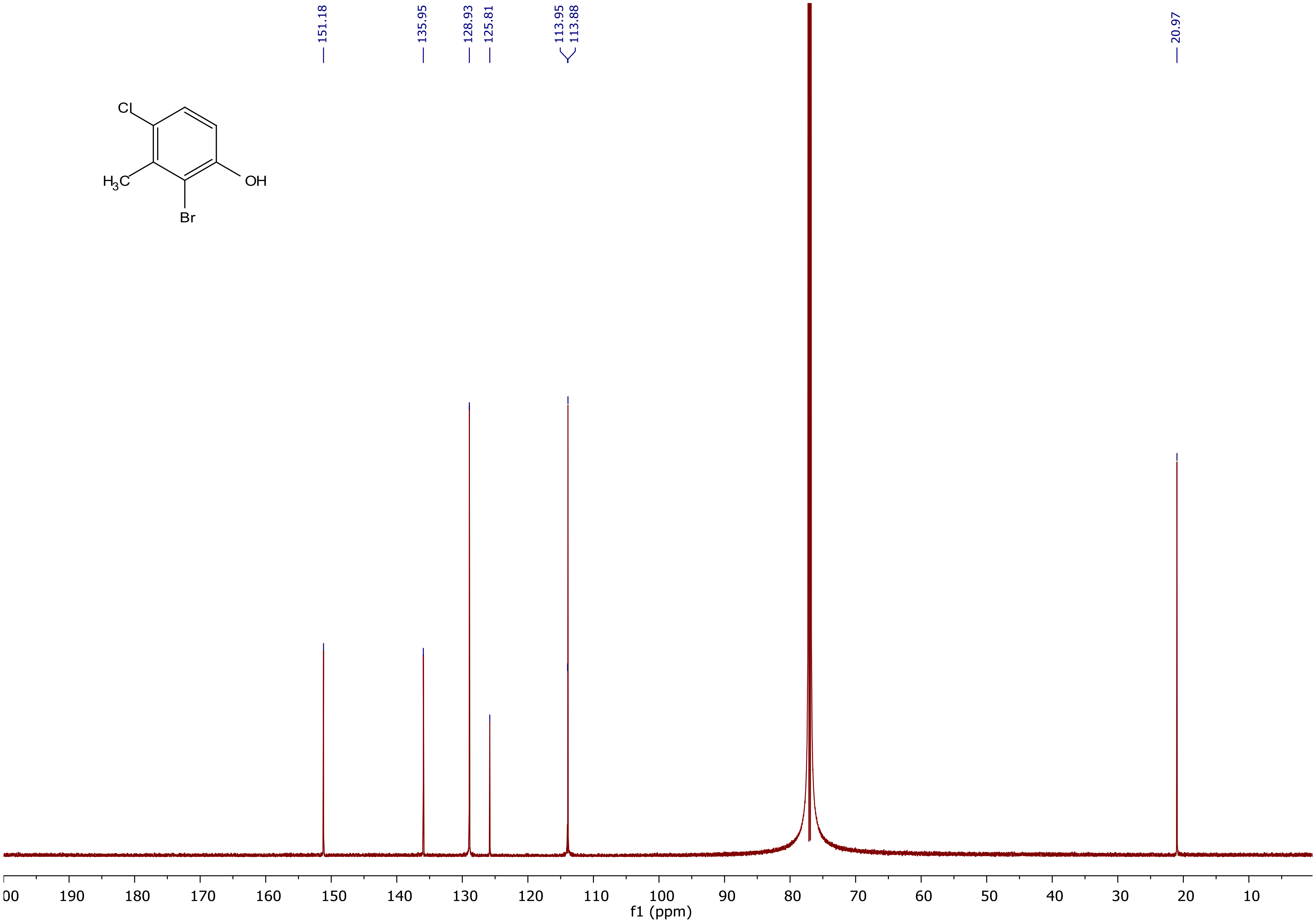

^{1}H-NMR ($CDCl_3$): 7-bromo-6-methylindole

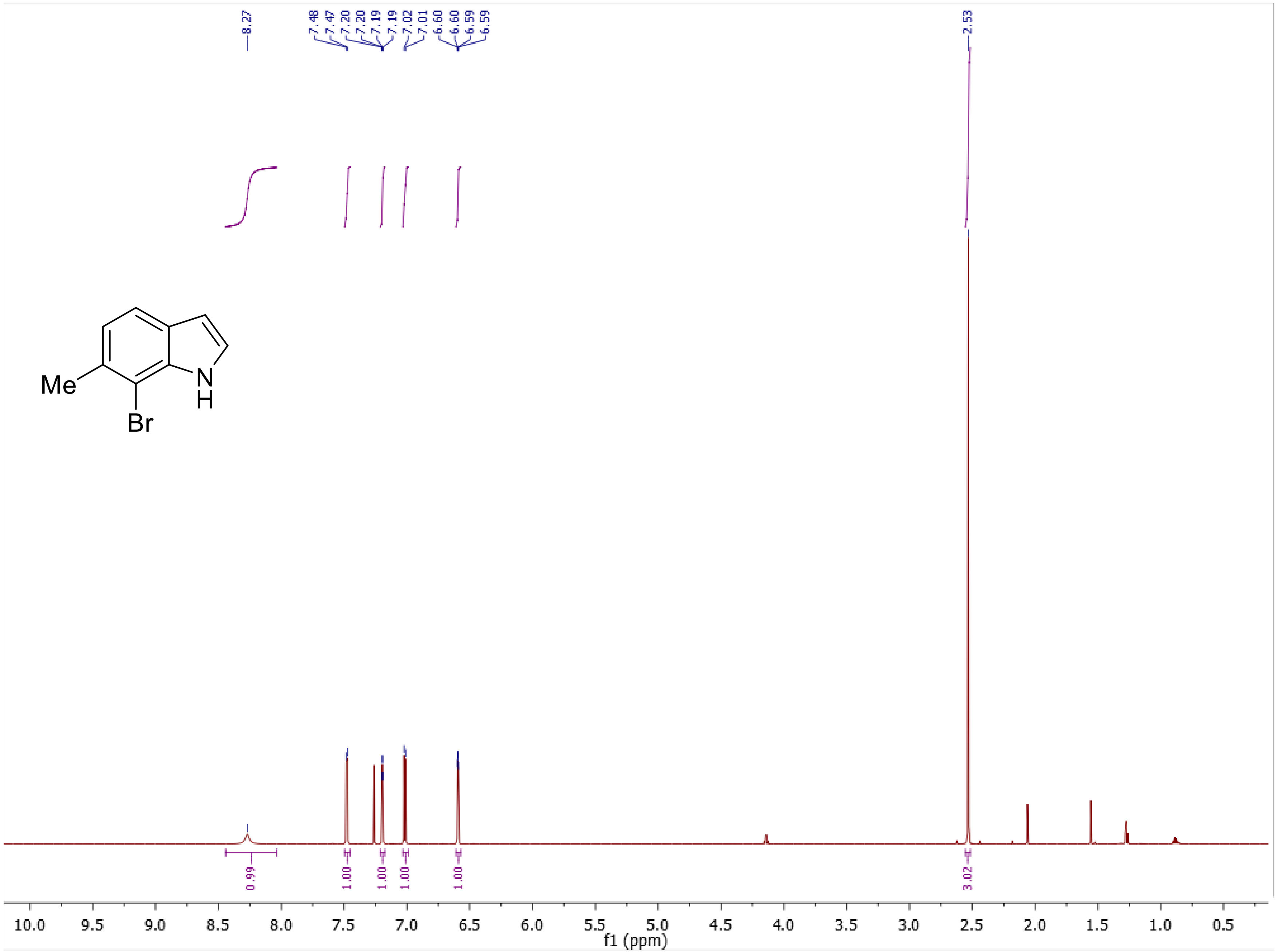

^{13}C-NMR ($CDCl_3$): 7-bromo-6-methylindole

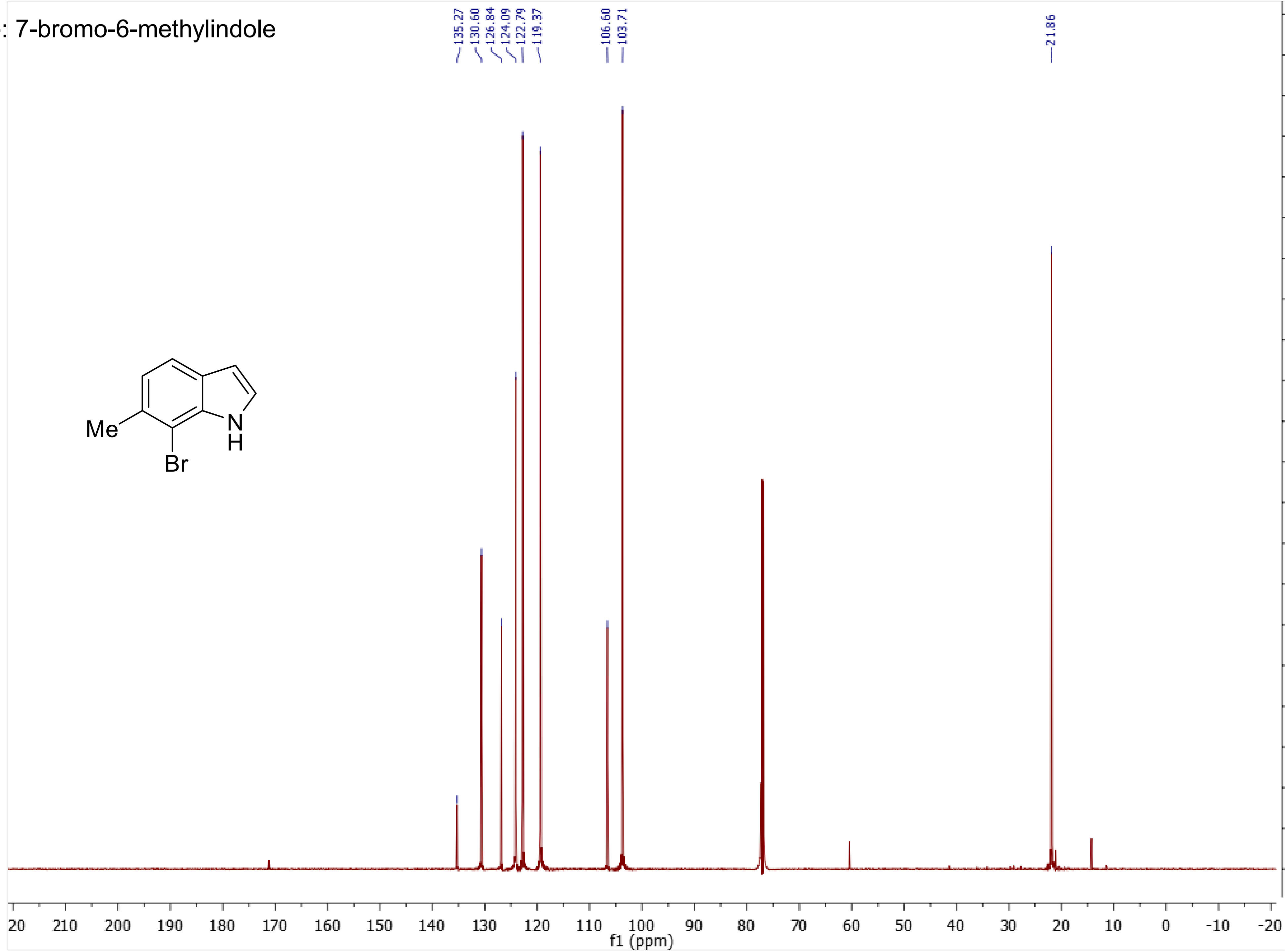

^{1}H-NMR ($CDCl_3$): 7-bromo-6-methylindoline

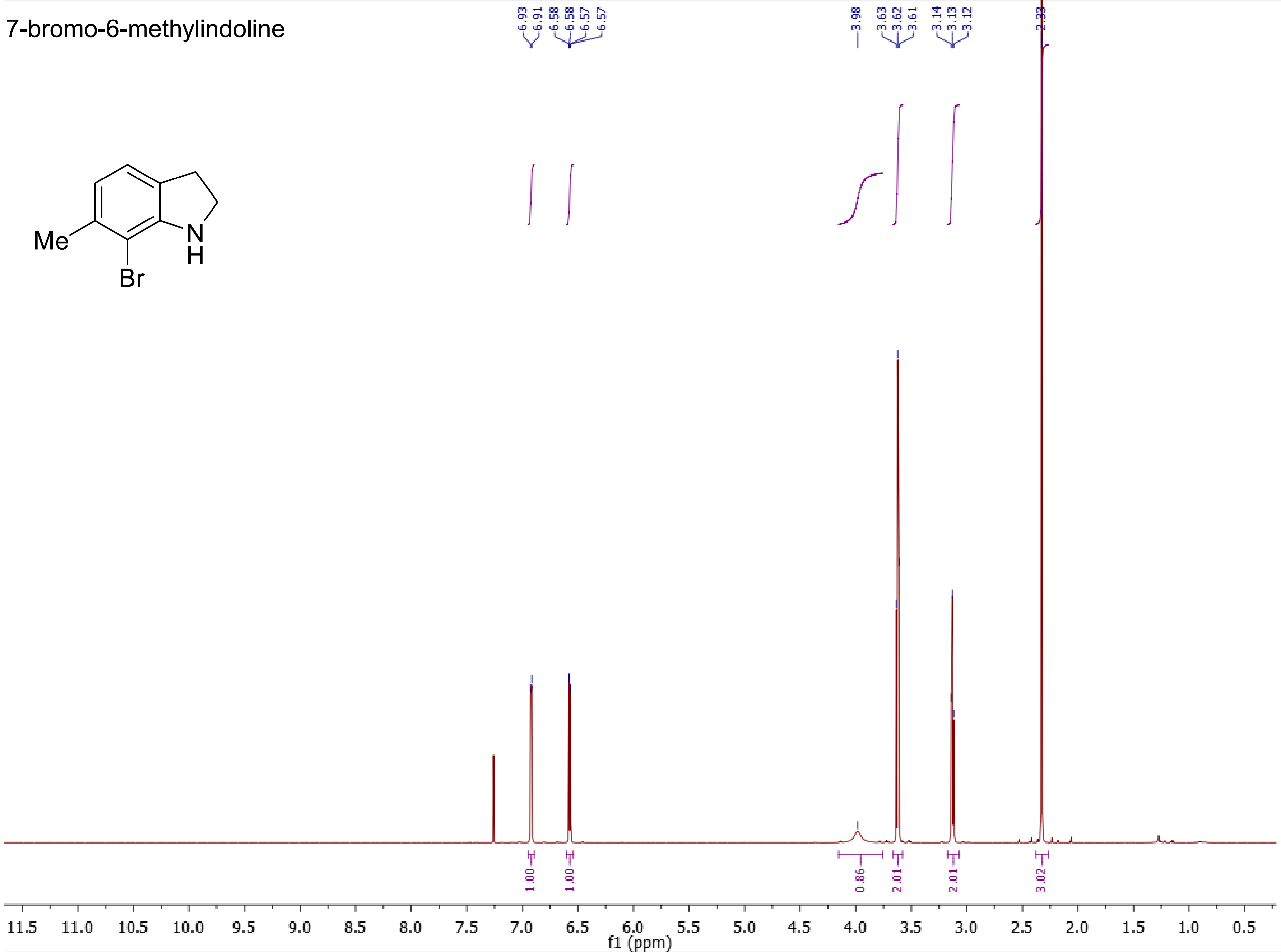

^{13}C-NMR ($CDCl_3$): 7-bromo-6-methylindoline

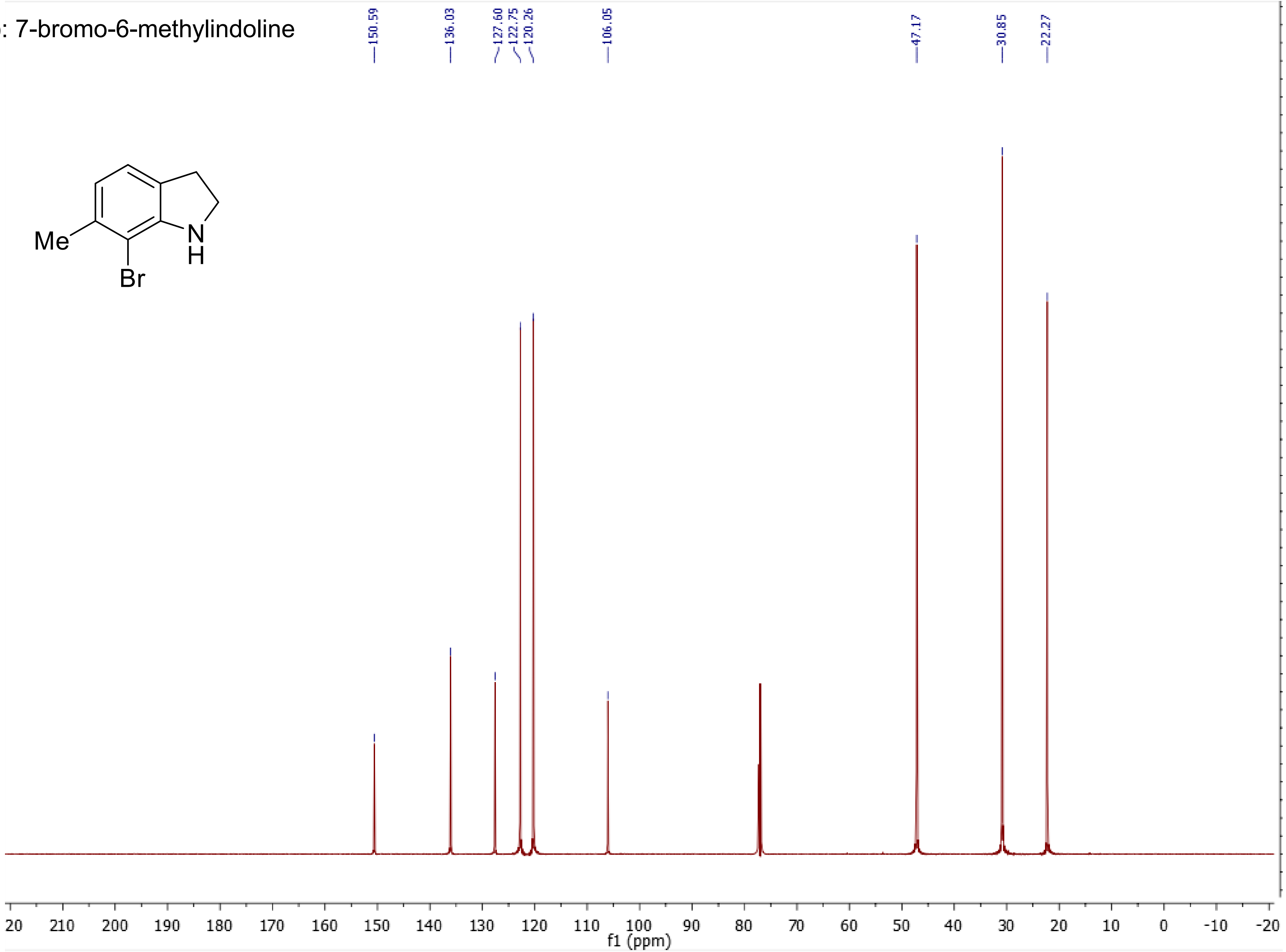

^{1}H-NMR (MeOD): *(S)*-2'-amino-6'-chloro-6-fluoro-[1,1'-biphenyl]-2-ol (**3a**)

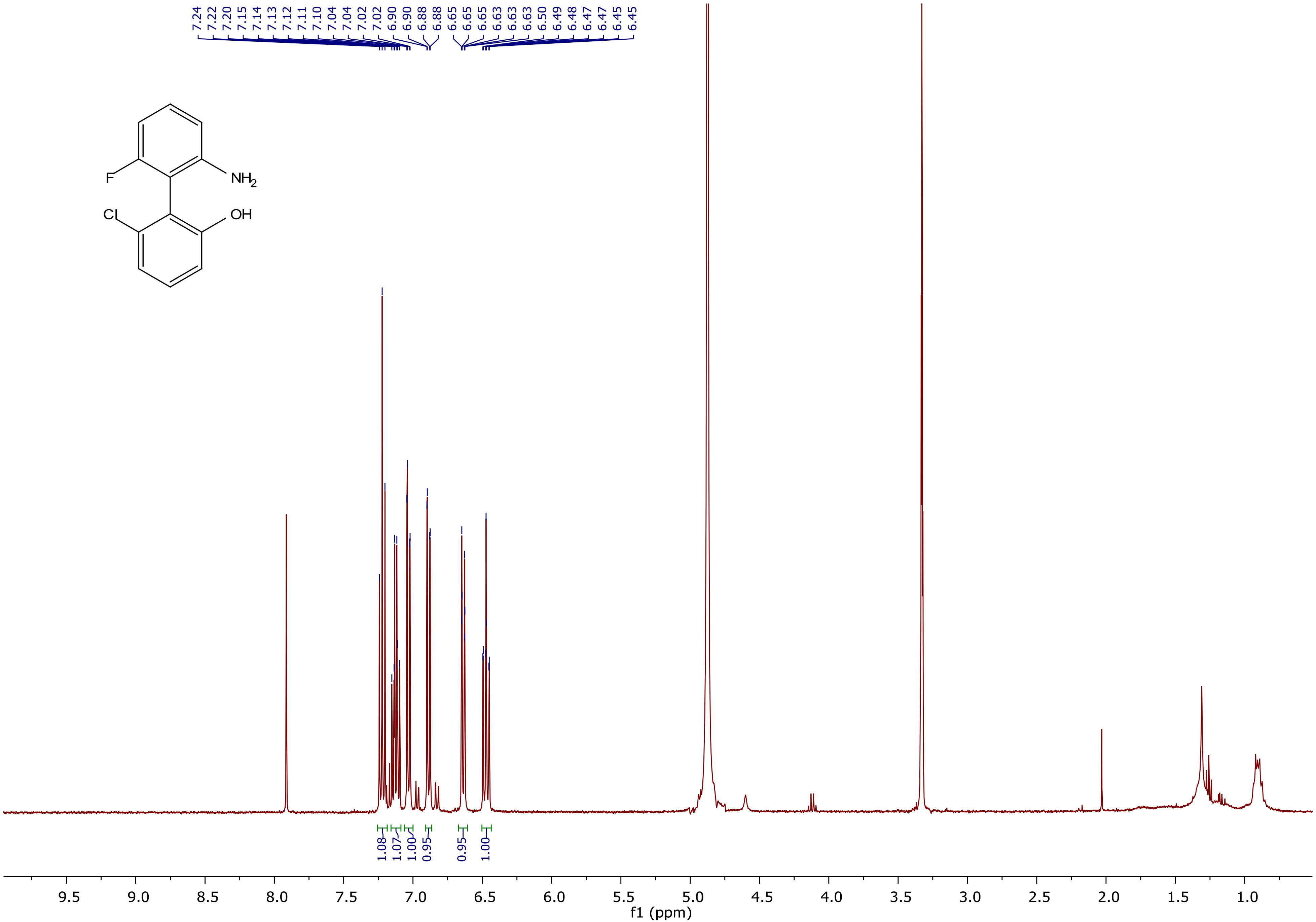

^{19}F-NMR (MeOD): *(S)*-2'-amino-6'-chloro-6-fluoro-[1,1'-biphenyl]-2-ol (**3a)**

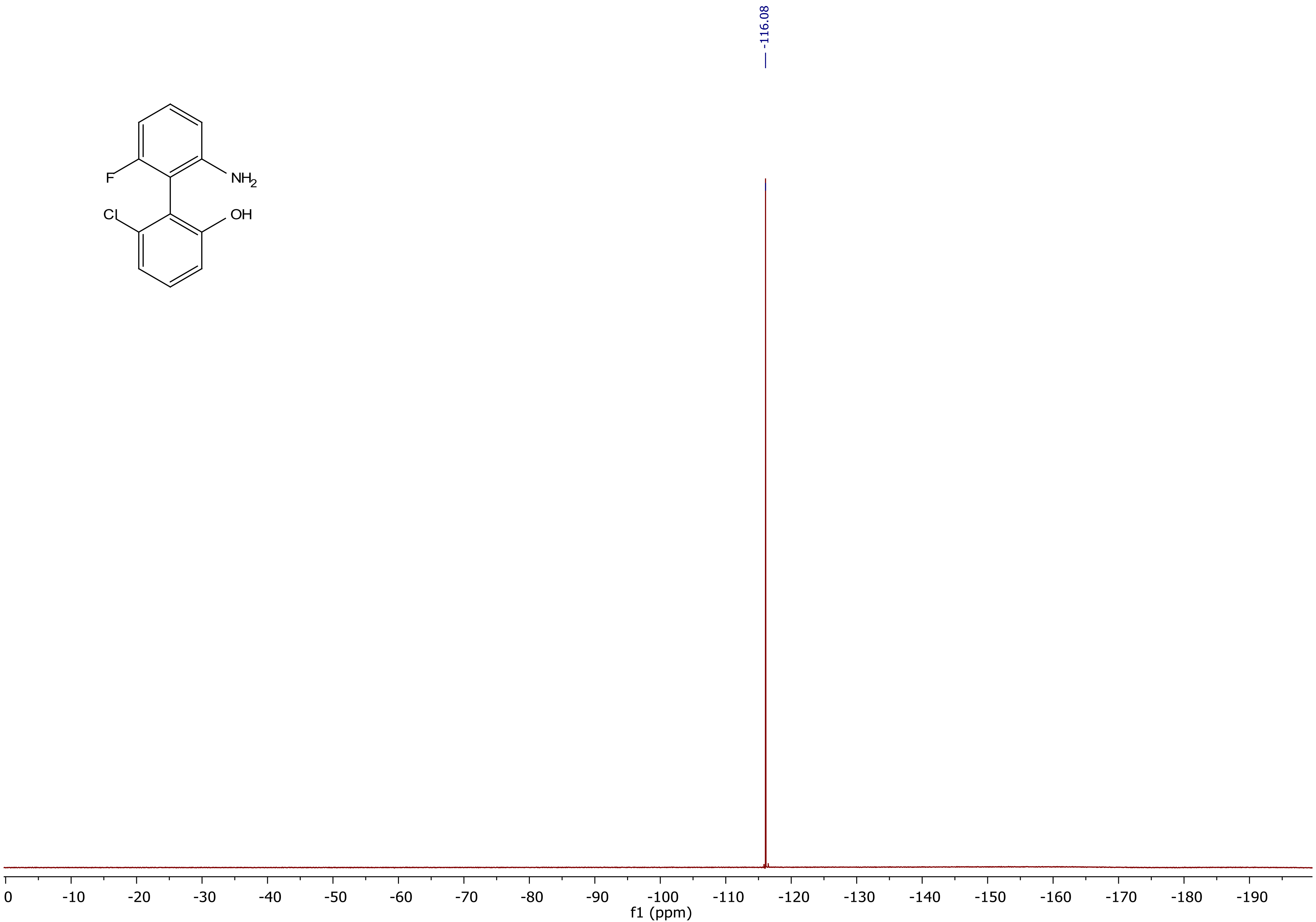

^{13}C-NMR (MeOD): *(S)*-2'-amino-6'-chloro-6-fluoro-[1,1'-biphenyl]-2-ol (**3a**)

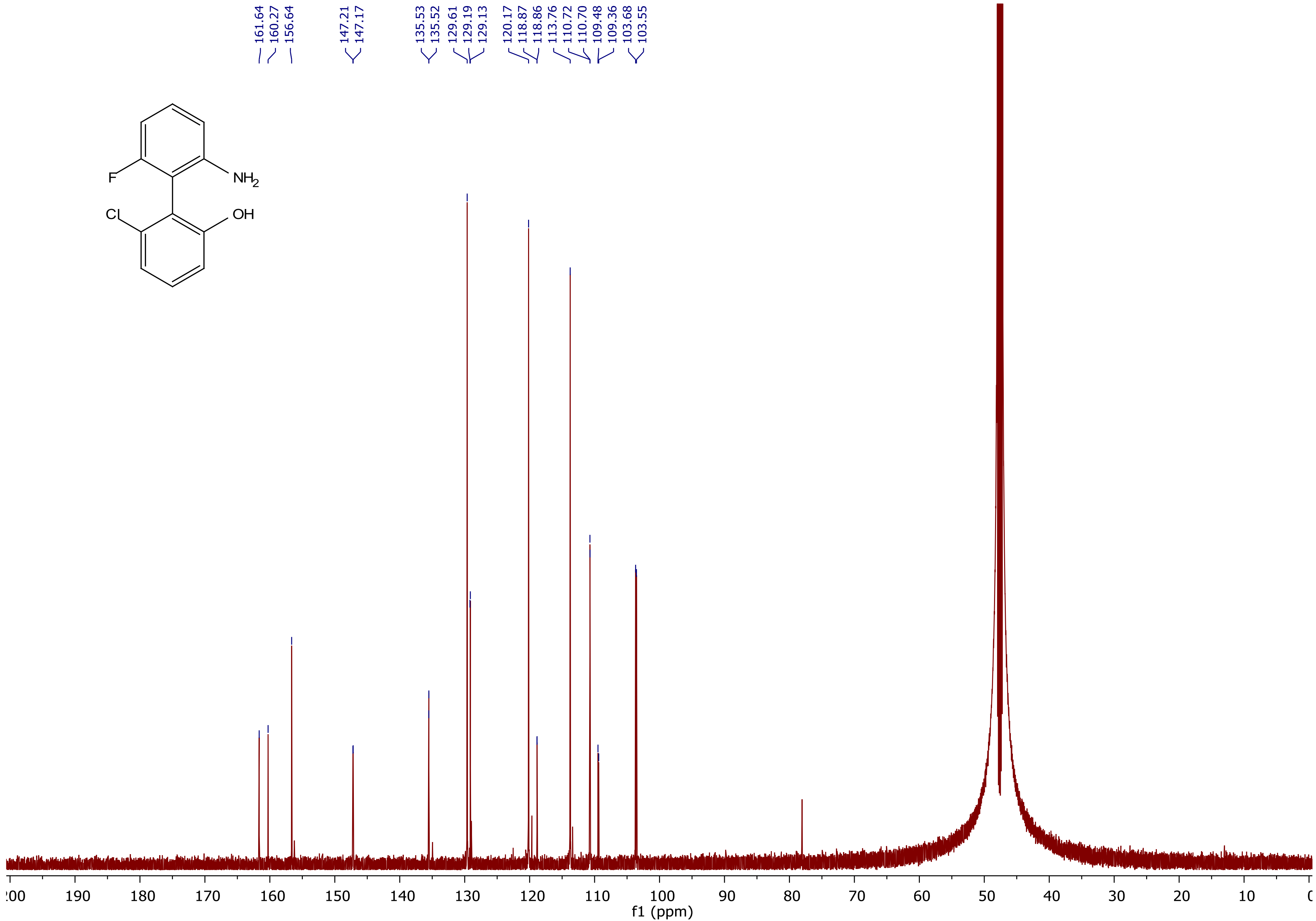

¹H-NMR ($CDCl_3$): *(S)*-2'-amino-6-chloro-4',6'-difluoro-[1,1'-biphenyl]-2-ol (**3b**)

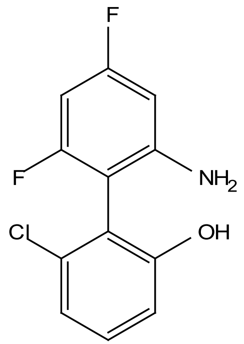

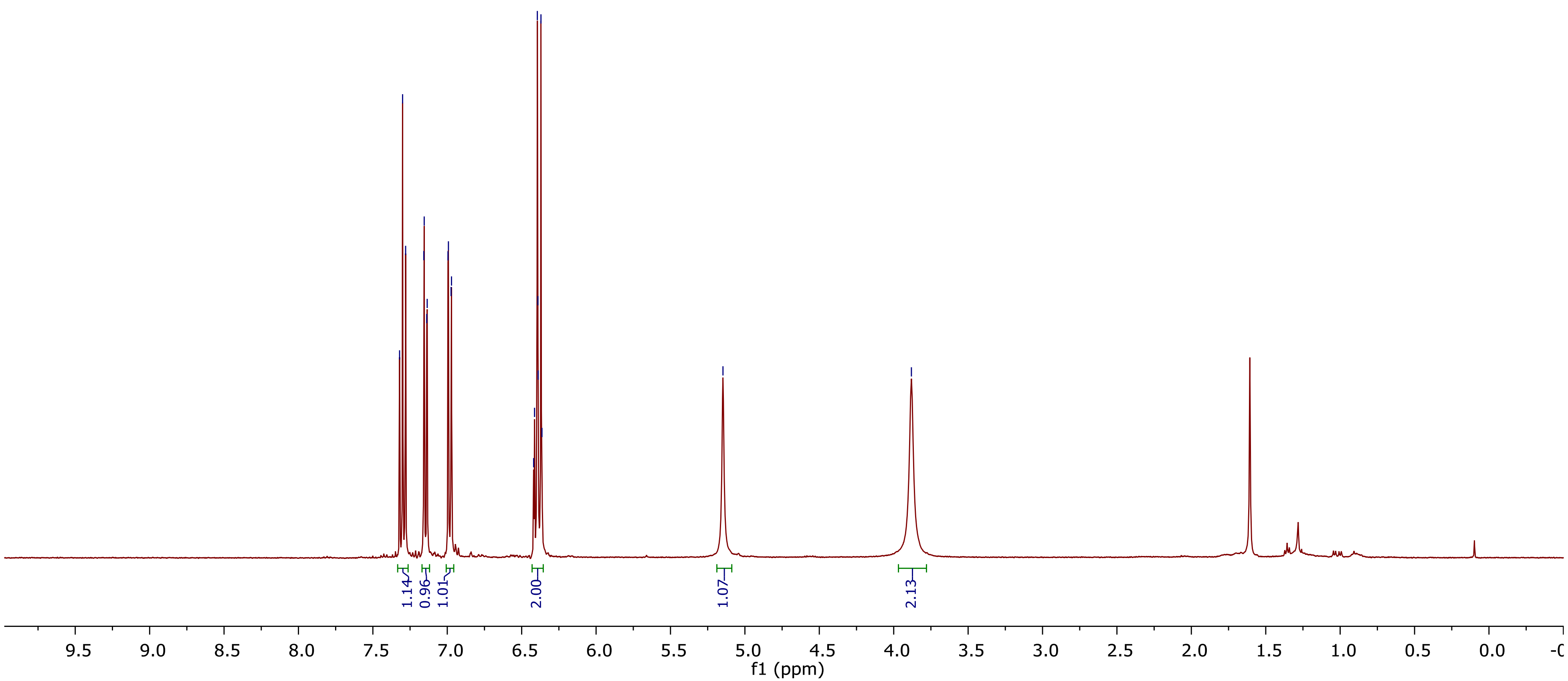

^{19}F-NMR ($CDCl_3$): *(S)*-2'-amino-6-chloro-4',6'-difluoro-[1,1'-biphenyl]-2-ol (**3b**)

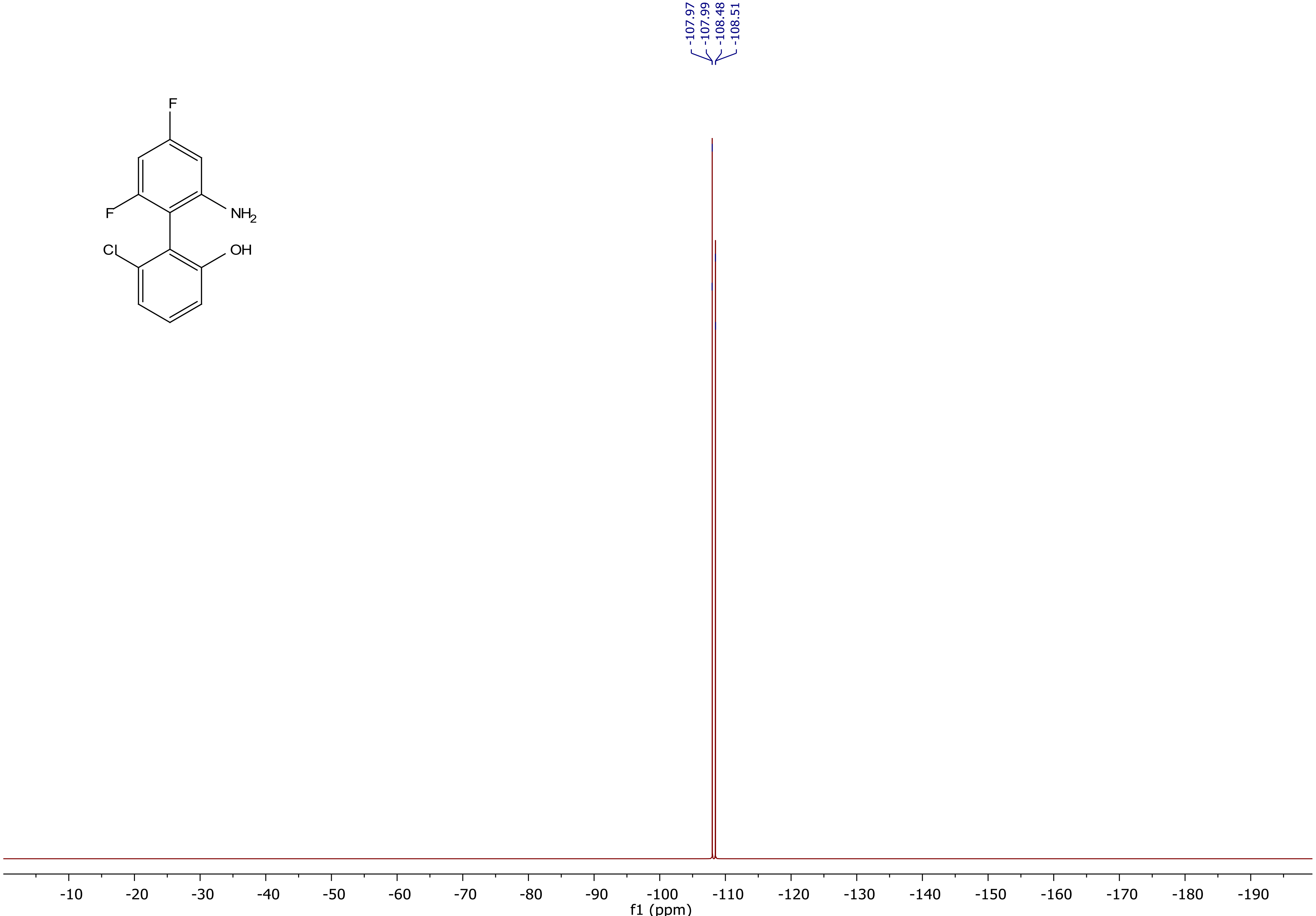

^{13}C-NMR (CDCl$_3$): *(S)*-2'-amino-6-chloro-4',6'-difluoro-[1,1'-biphenyl]-2-ol (**3b**)

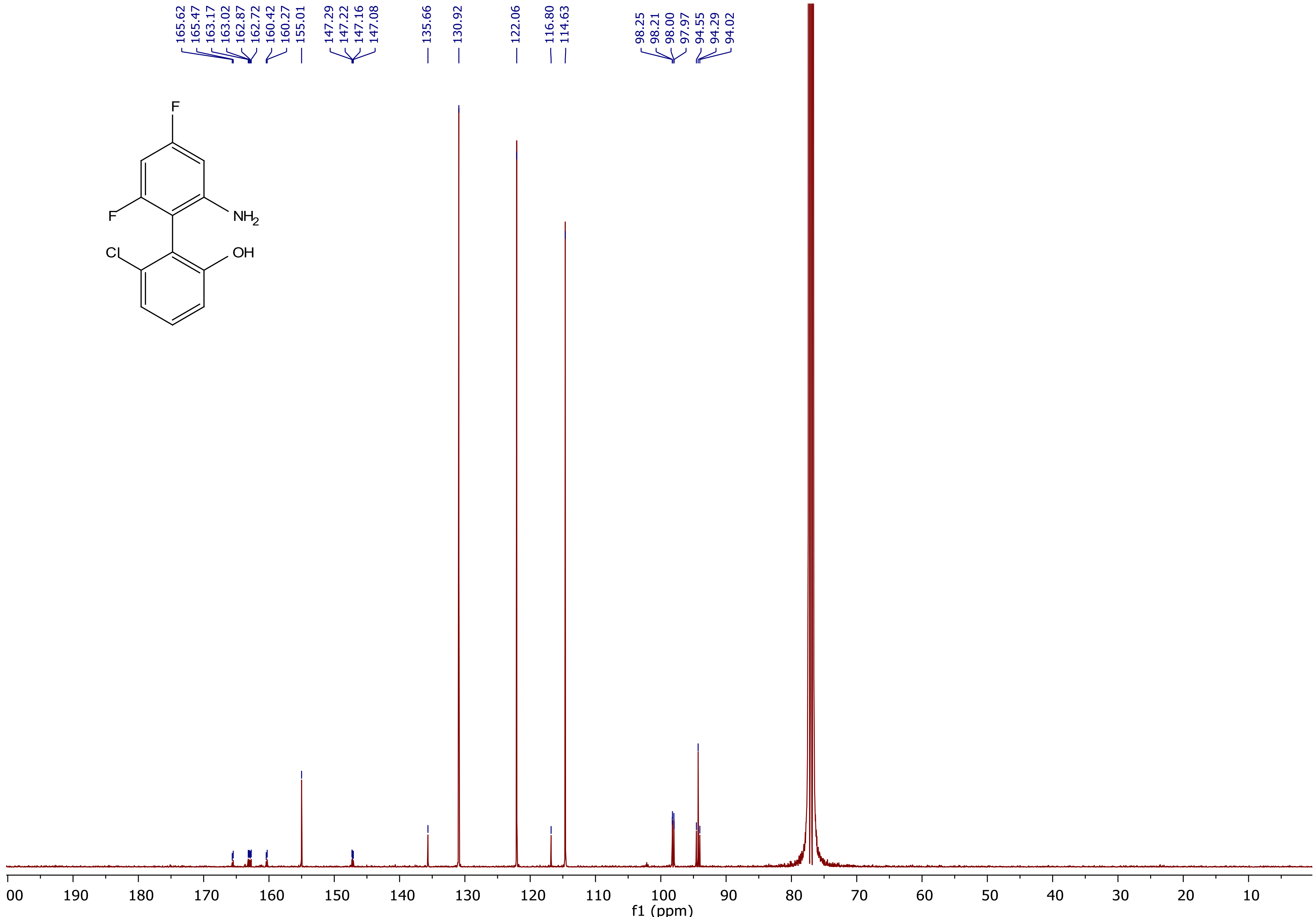

^{1}H-NMR ($CDCl_3$): *(S)*-6'-amino-6-chloro-2',3'-difluoro-[1,1'-biphenyl]-2-ol (**3c**)

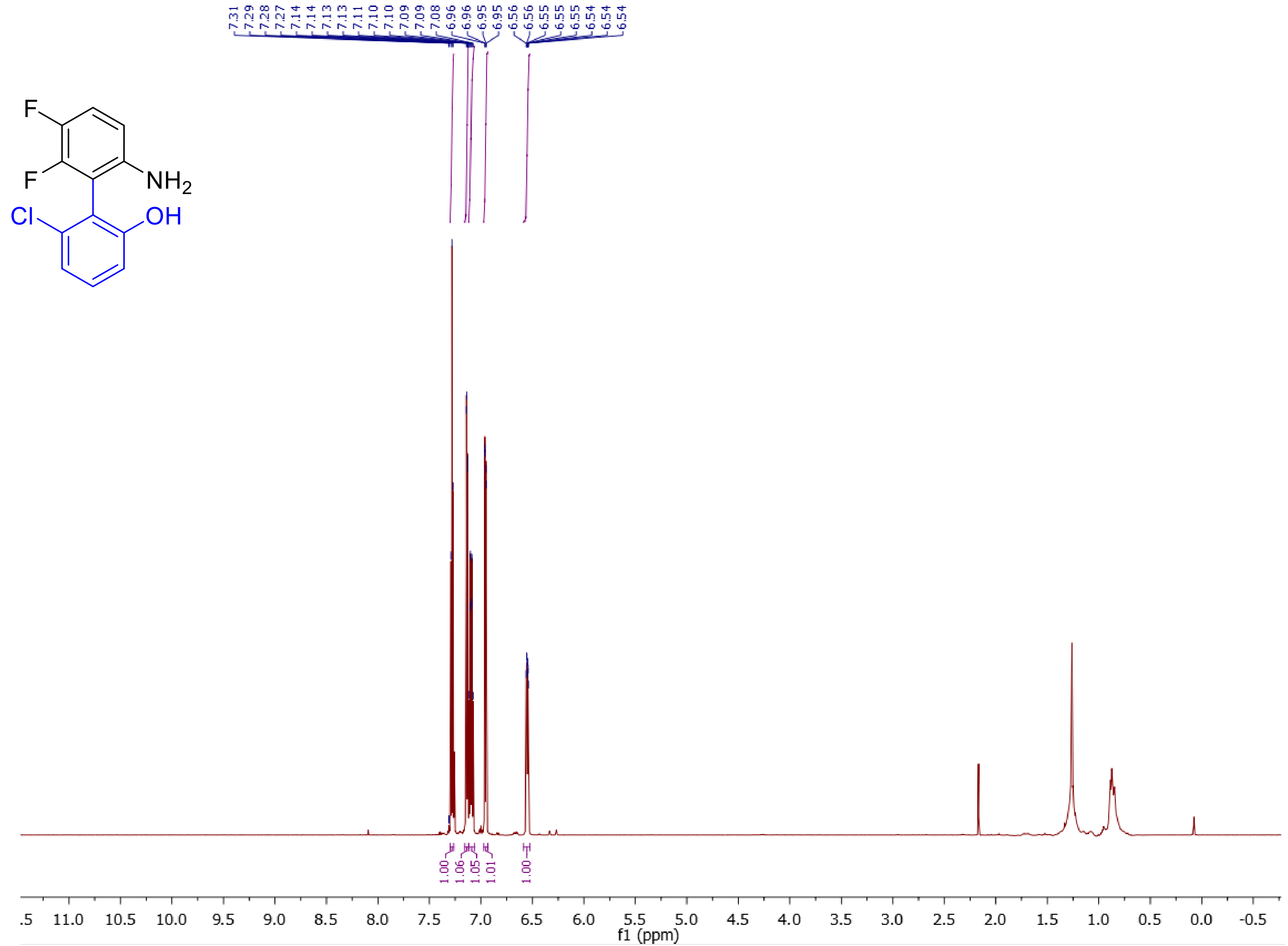

^{19}F-NMR ($CDCl_3$): *(S)*-6'-amino-6-chloro-2',3'-difluoro-[1,1'-biphenyl]-2-ol (**3c**)

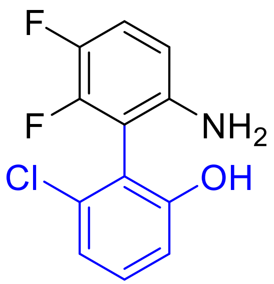

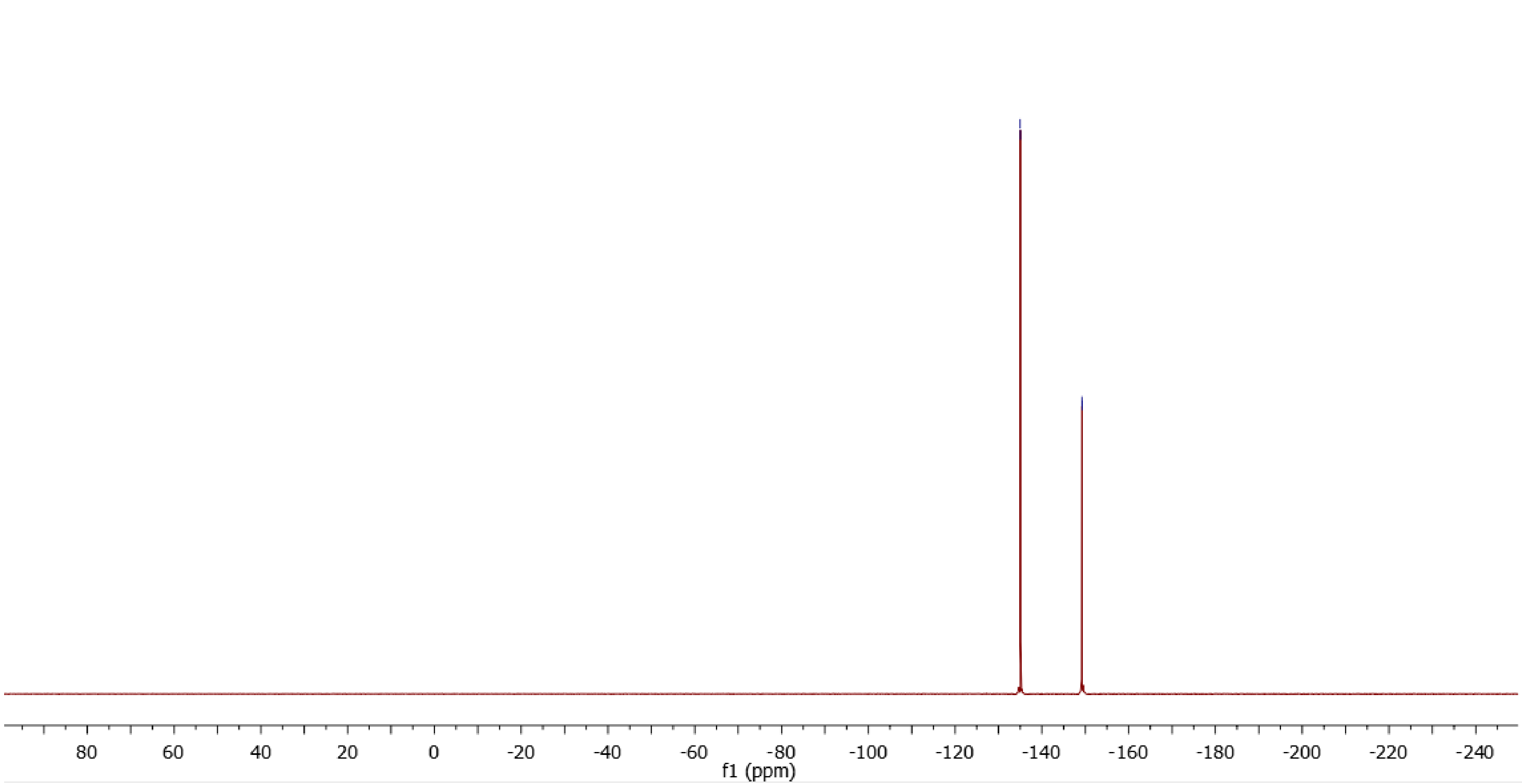

^{13}C-NMR ($CDCl_3$): *(S)*-6'-amino-6-chloro-2',3'-difluoro-[1,1'-biphenyl]-2-ol (**3c**)

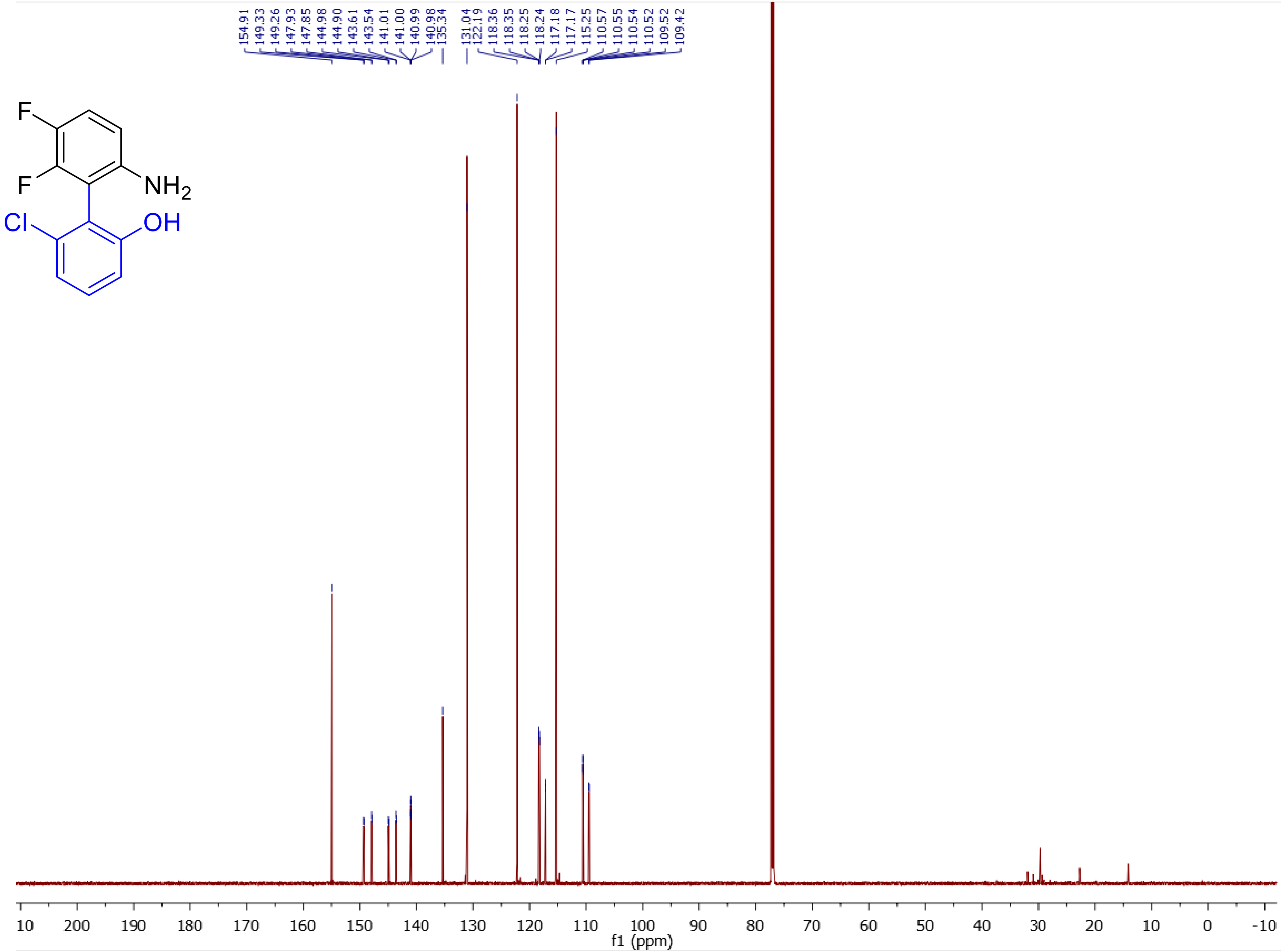

^{1}H-NMR (MeOD): *(S)*- 6'-amino-3',6-dichloro-2'-fluoro-[1,1'-biphenyl]-2-ol (**3d**)

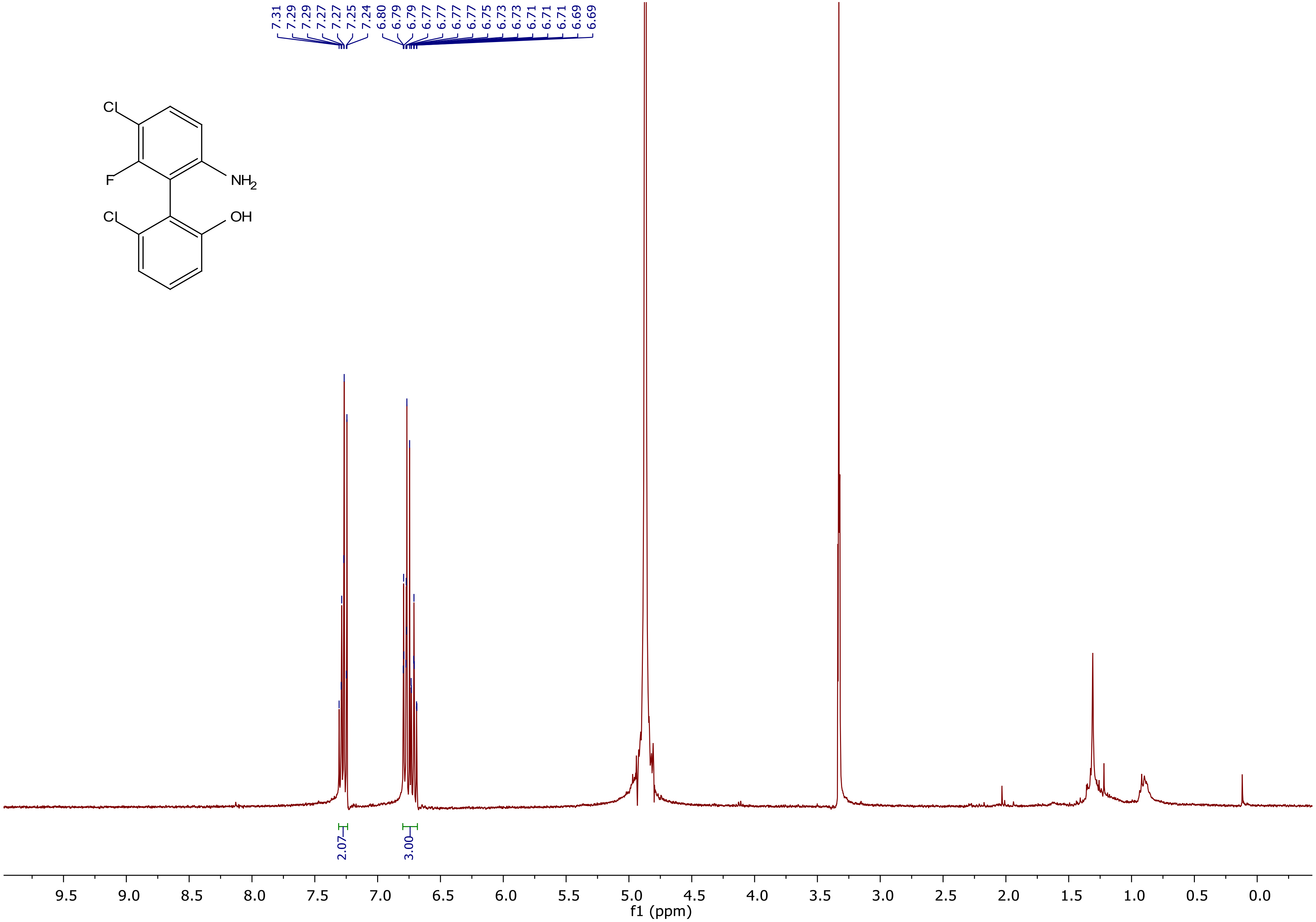

^{19}F-NMR (MeOD): *(S)*- 6'-amino-3',6-dichloro-2'-fluoro-[1,1'-biphenyl]-2-ol (**3d**)

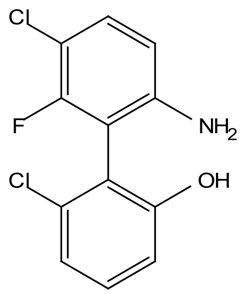

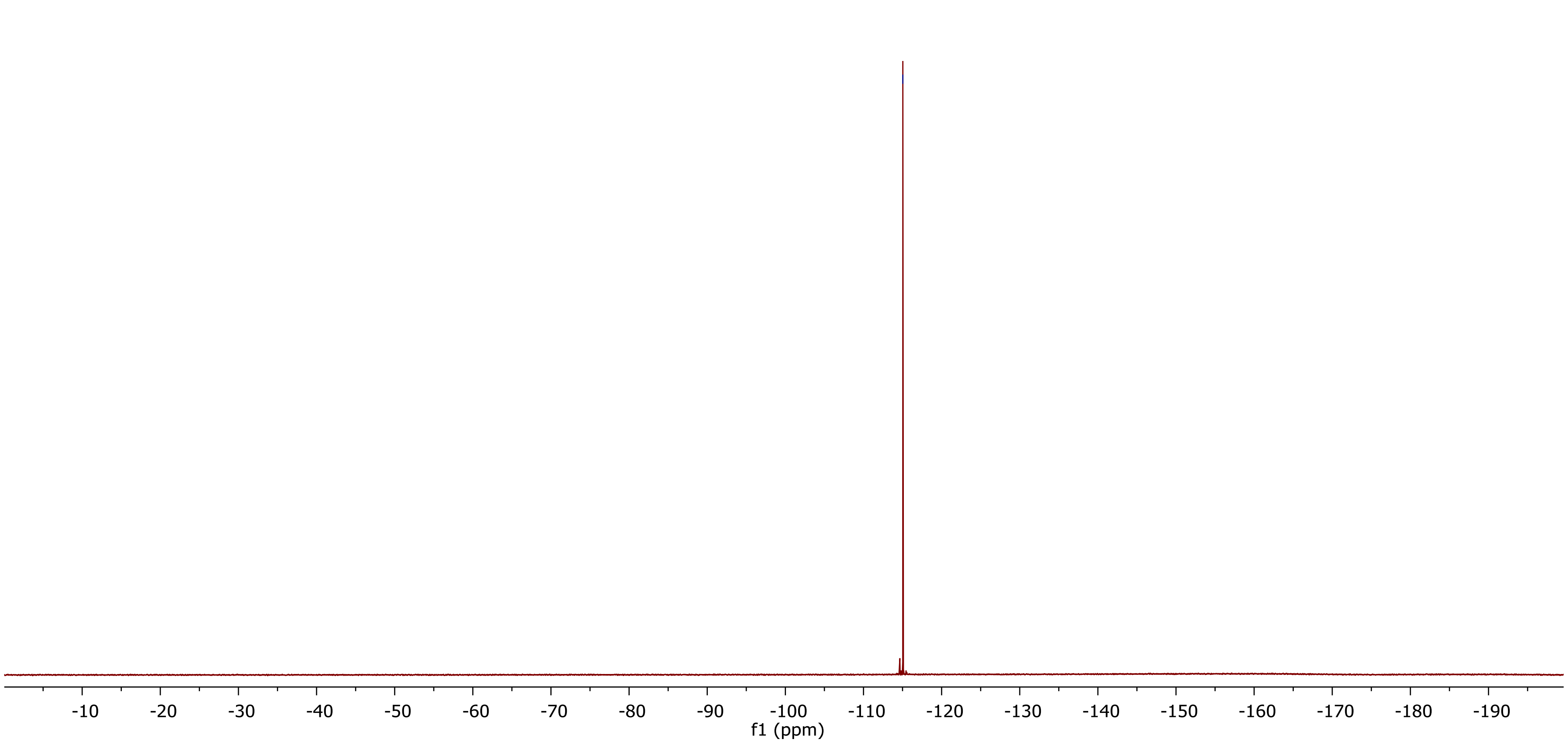

^{13}C-NMR (MeOD): *(S)*- 6'-amino-3',6-dichloro-2'-fluoro-[1,1'-biphenyl]-2-ol (**3d**)

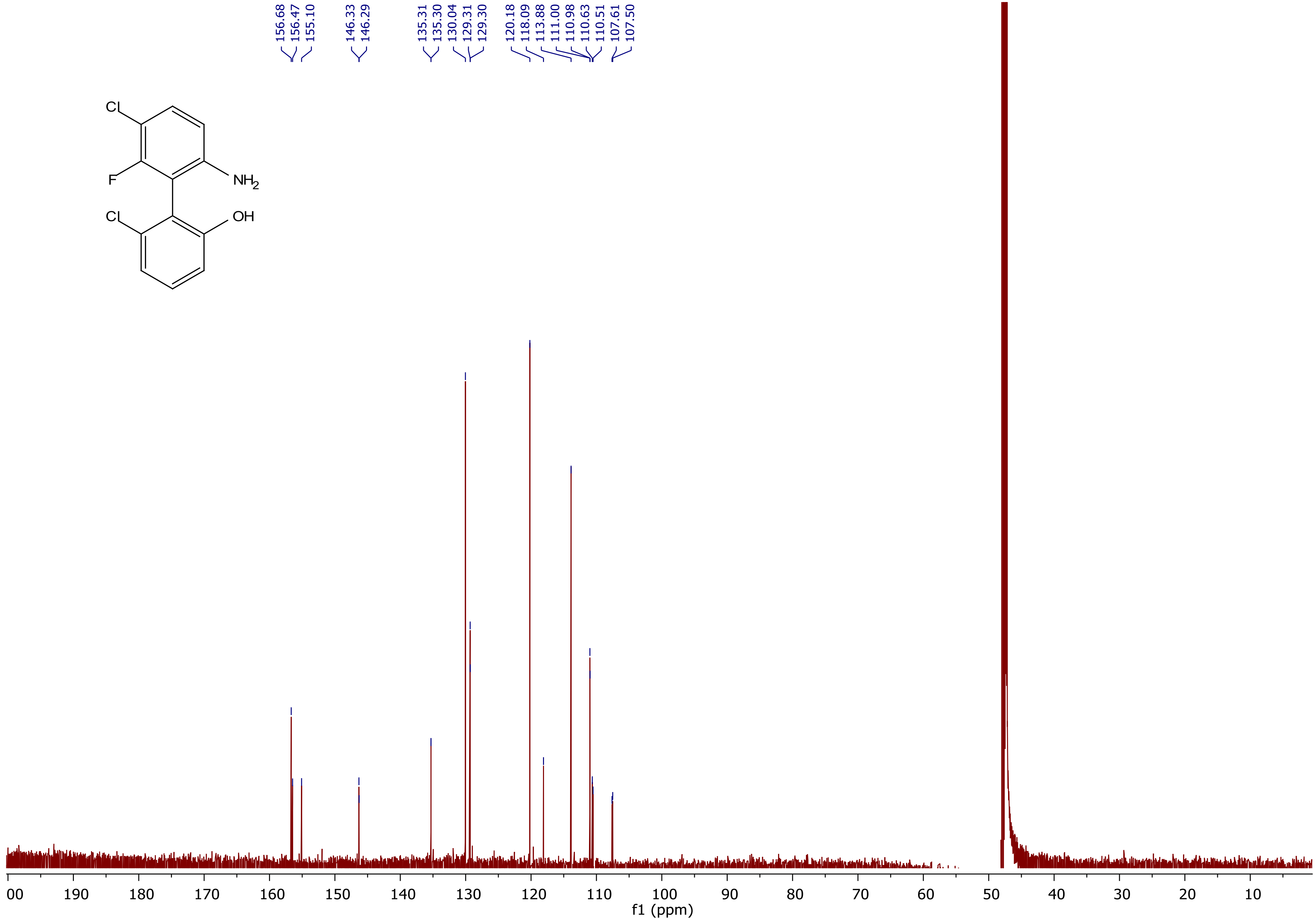

^{1}H-NMR ($CDCl_3$): *(S)*-2'-amino-6-chloro-6'-fluoro-3'-nitro-[1,1'-biphenyl]-2-ol (**3e**)

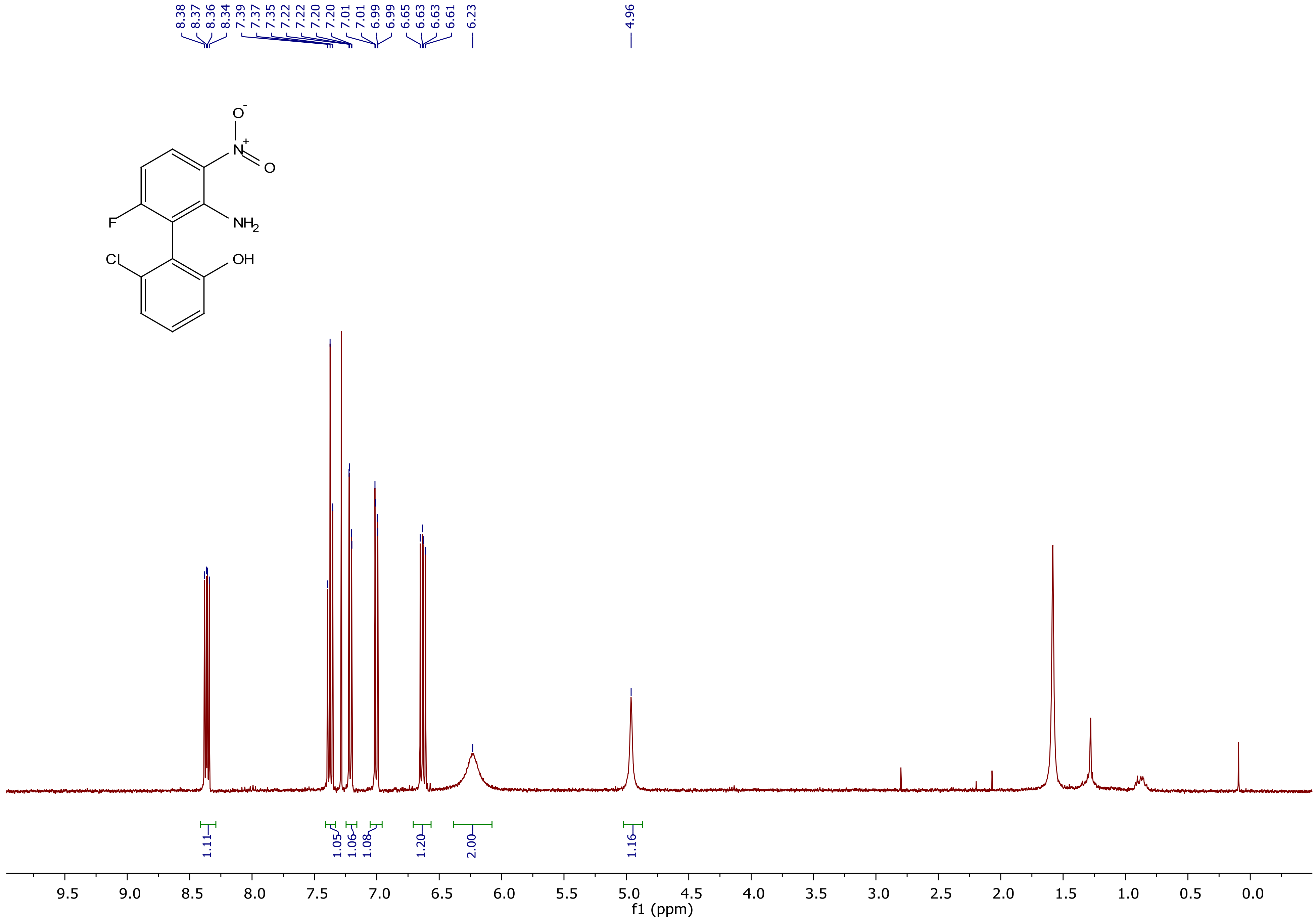

^{19}F-NMR ($CDCl_3$): *(S)*-2'-amino-6-chloro-6'-fluoro-3'-nitro-[1,1'-biphenyl]-2-ol (**3e**)

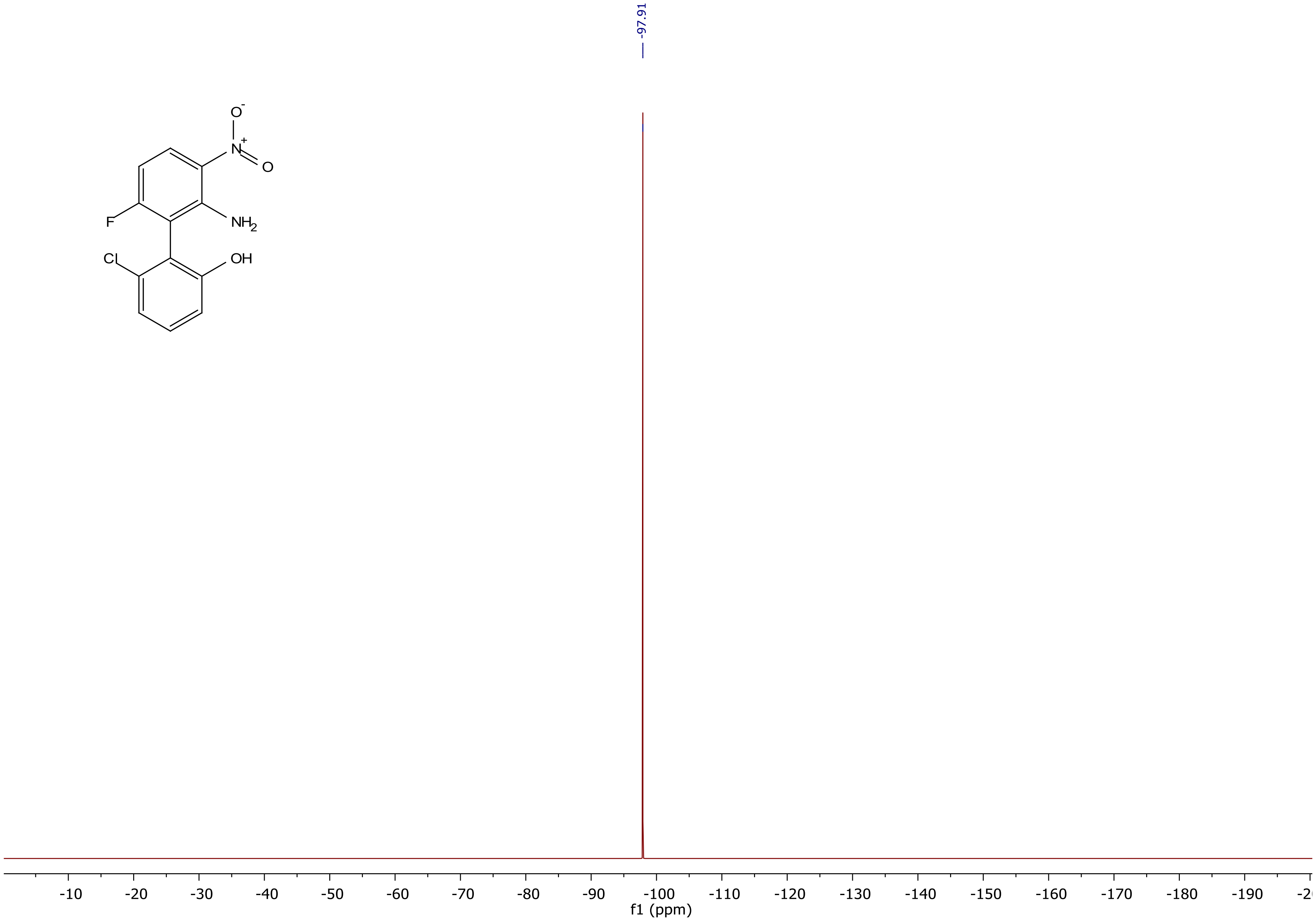

^{13}C-NMR (CDCl$_3$): *(S)*-2'-amino-6-chloro-6'-fluoro-3'-nitro-[1,1'-biphenyl]-2-ol (**3e**)

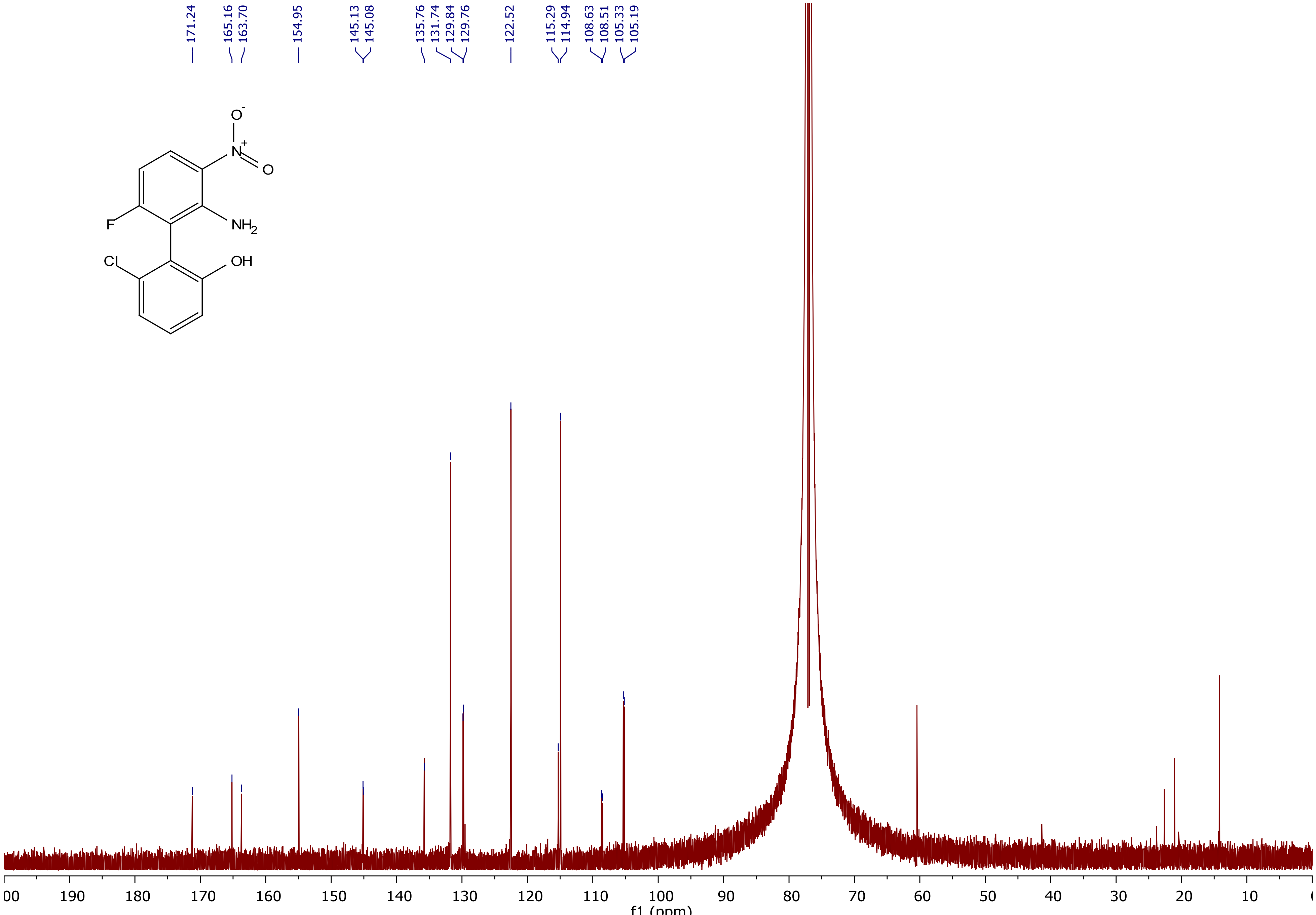

^{1}H-NMR (MeOD): (S)-2'-amino-6'-chloro-6-fluoro-[1,1'-biphenyl]-2-ol (**3f**)

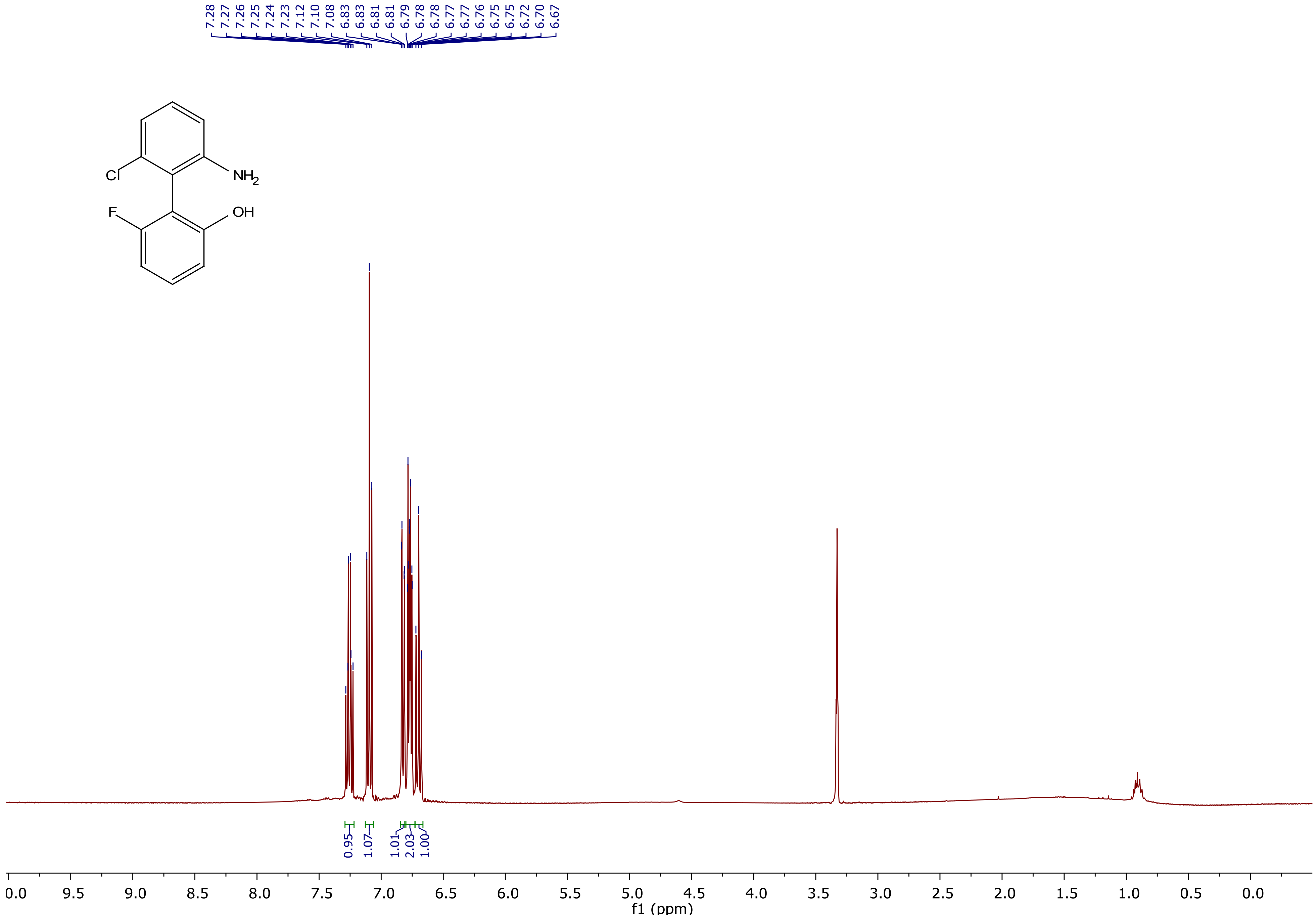

^{19}F-NMR (MeOD): (S)-2'-amino-6'-chloro-6-fluoro-[1,1'-biphenyl]-2-ol (**3f**)

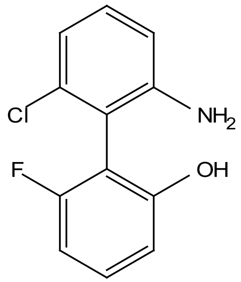

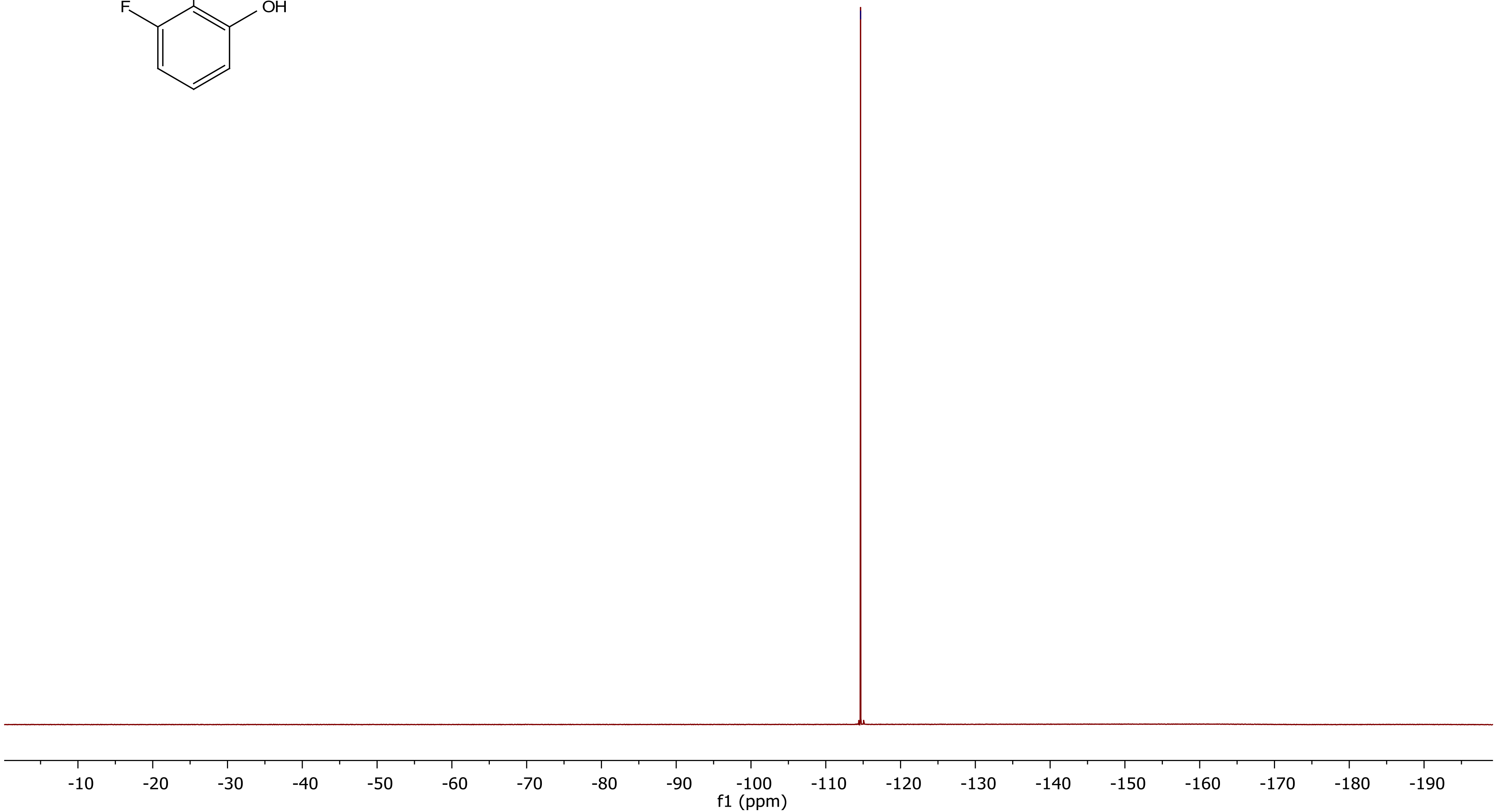

^{13}C-NMR (MeOD): (S)-2'-amino-6'-chloro-6-fluoro-[1,1'-biphenyl]-2-ol (**3f**)

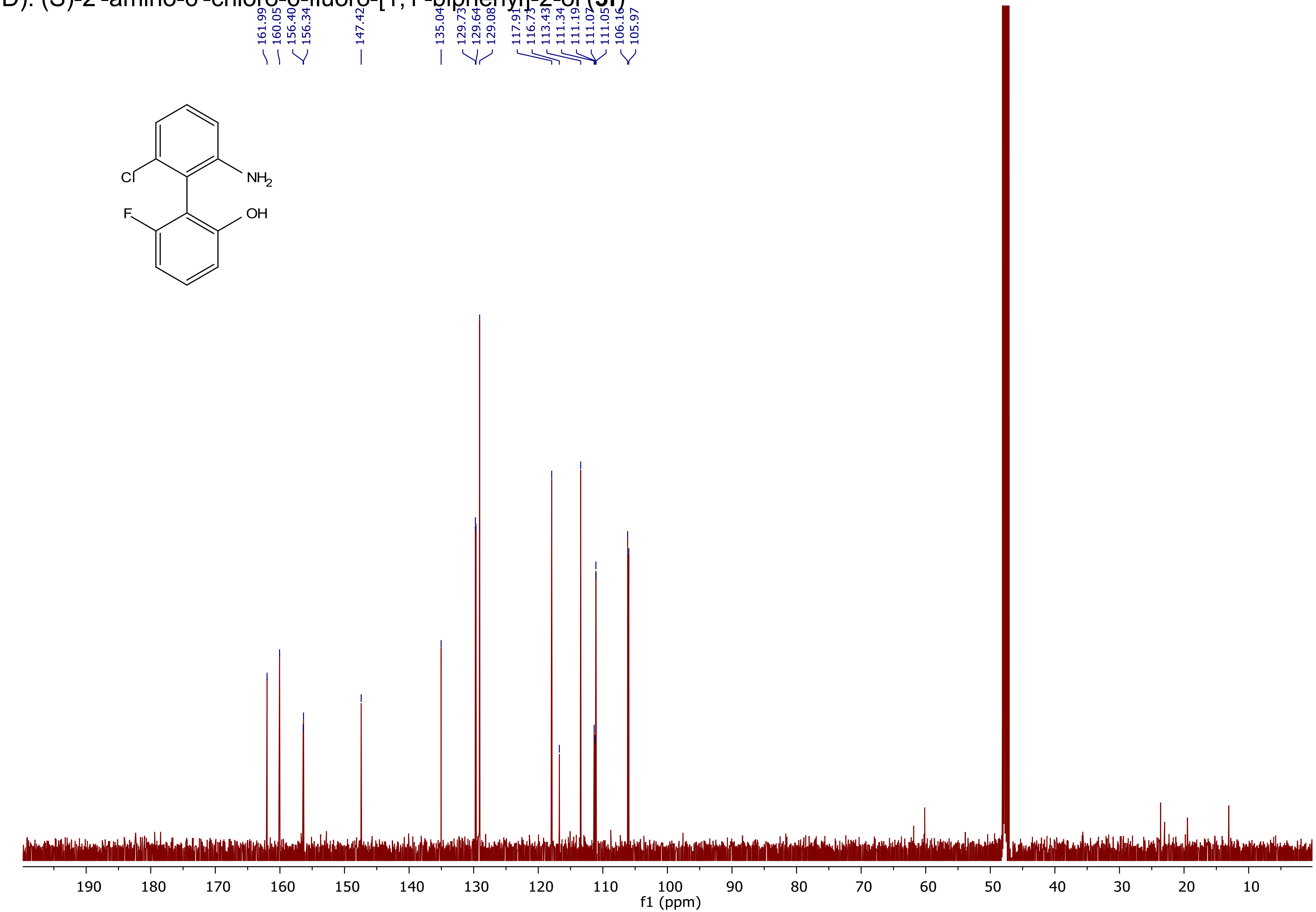

^{1}H-NMR (MeOD): *(S)*-6'-amino-2',3'-dichloro-6-fluoro-[1,1'-biphenyl]-2-ol (**3g**)

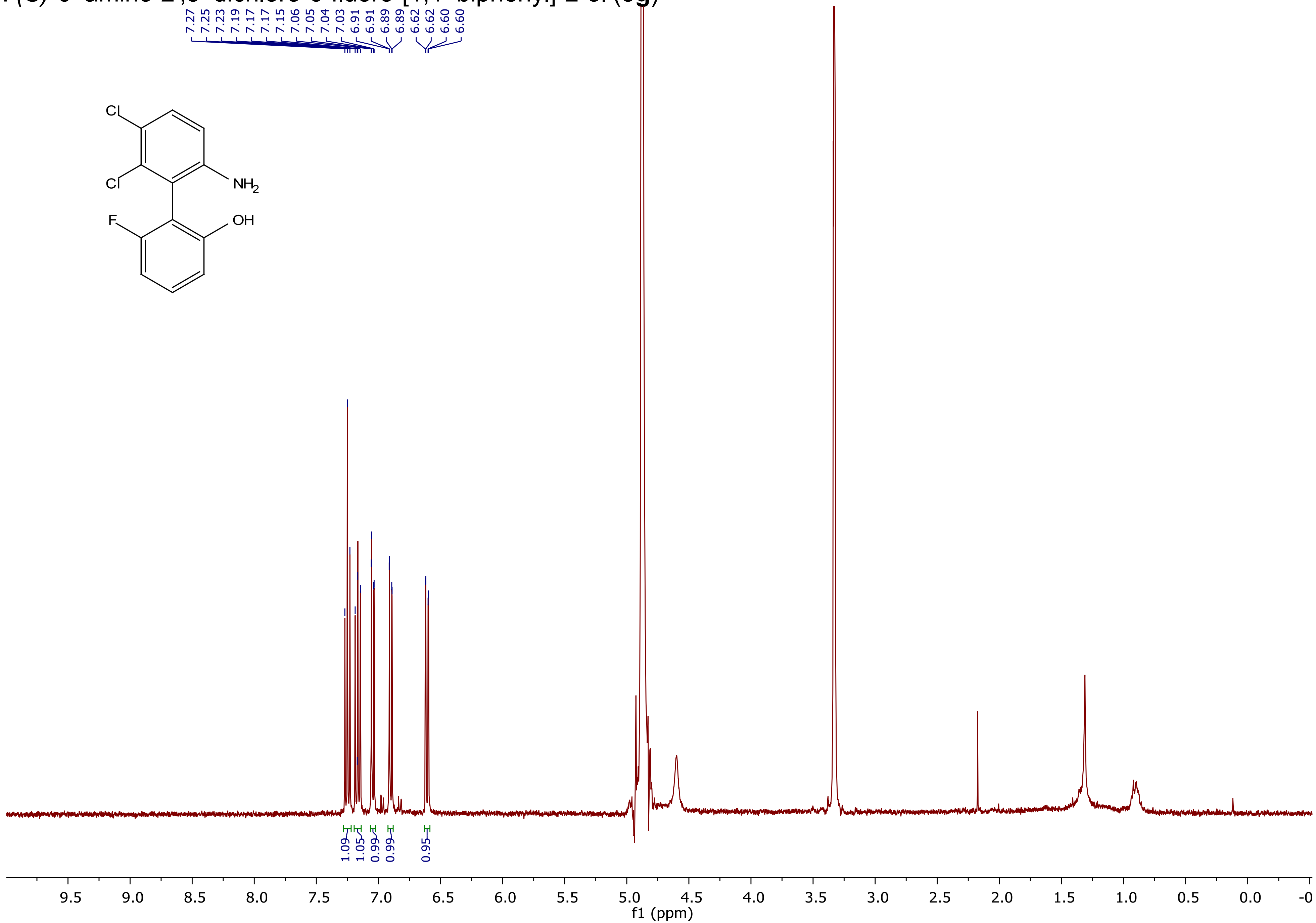

^{19}F-NMR (MeOD): *(S)*-6'-amino-2',3'-dichloro-6-fluoro-[1,1'-biphenyl]-2-ol (**3g**)

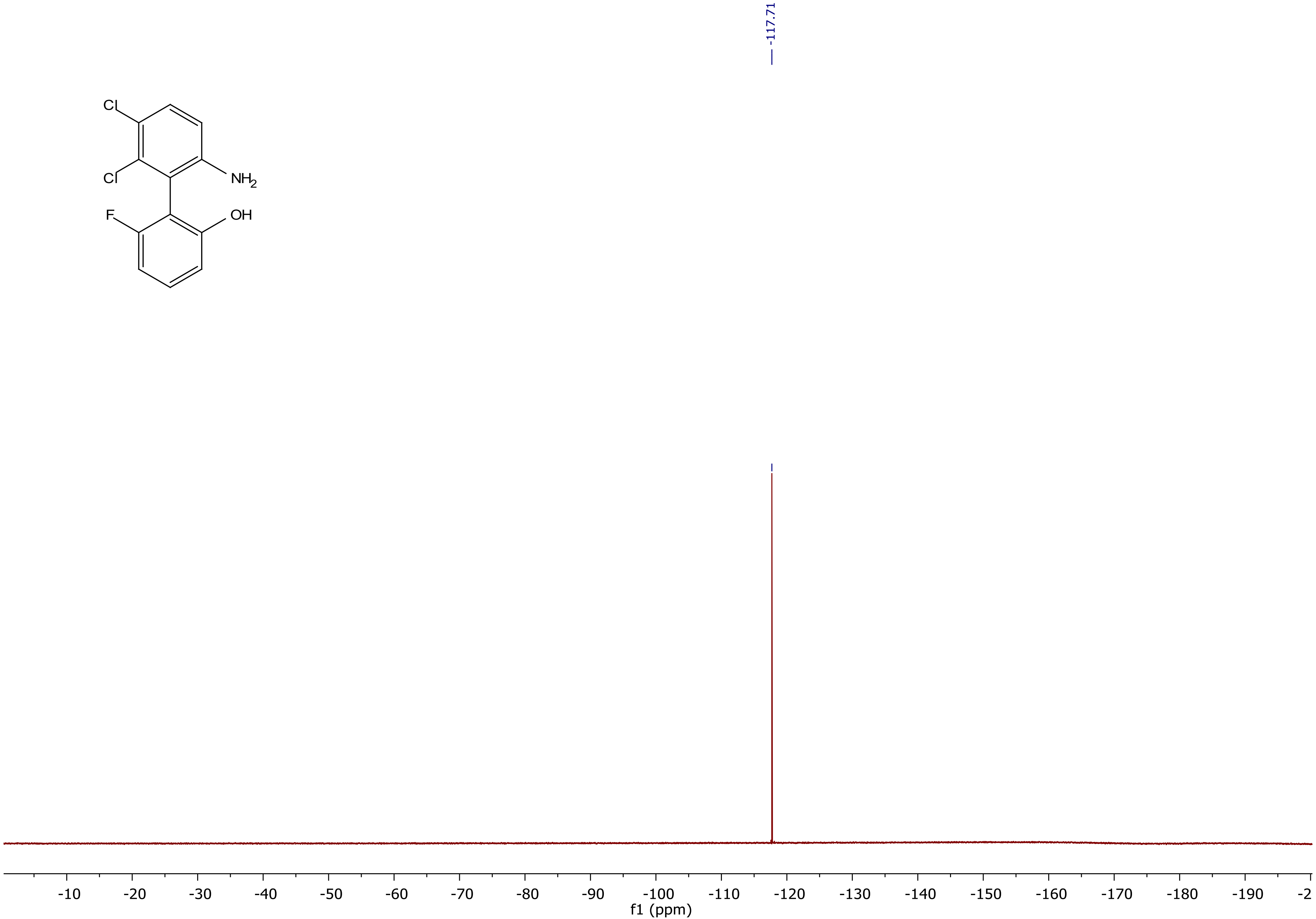

^{13}C-NMR (MeOD): *(S)*-6'-amino-2',3'-dichloro-6-fluoro-[1,1'-biphenyl]-2-ol (**3g**)

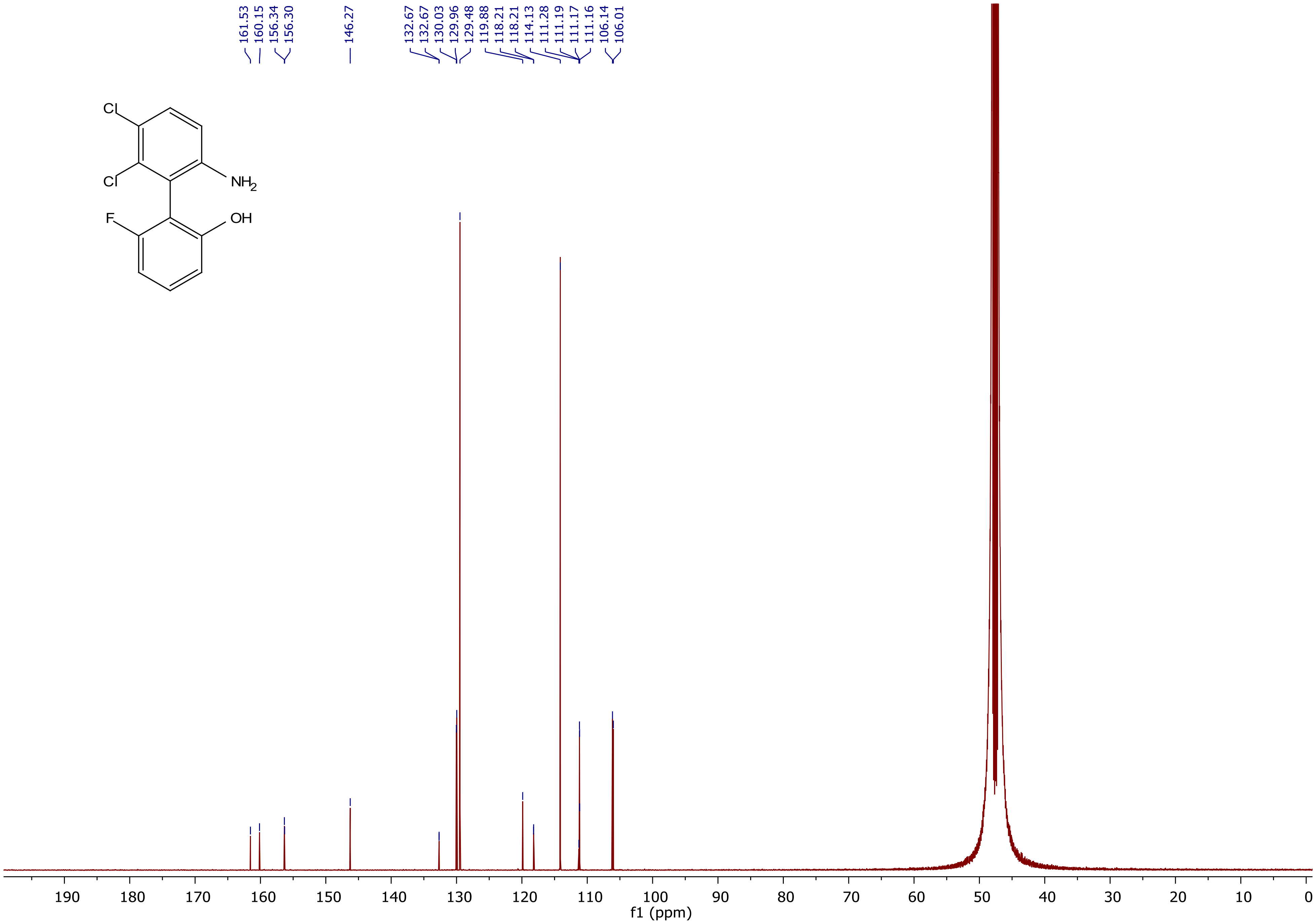

¹H-NMR ($CDCl_3$): *(S)*-2'-amino-4',6'-dichloro-6-fluoro-[1,1'-biphenyl]-2-ol (**3h**)

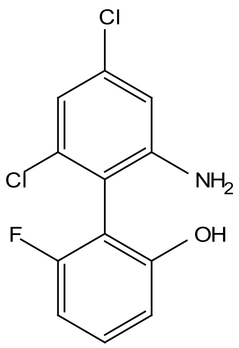

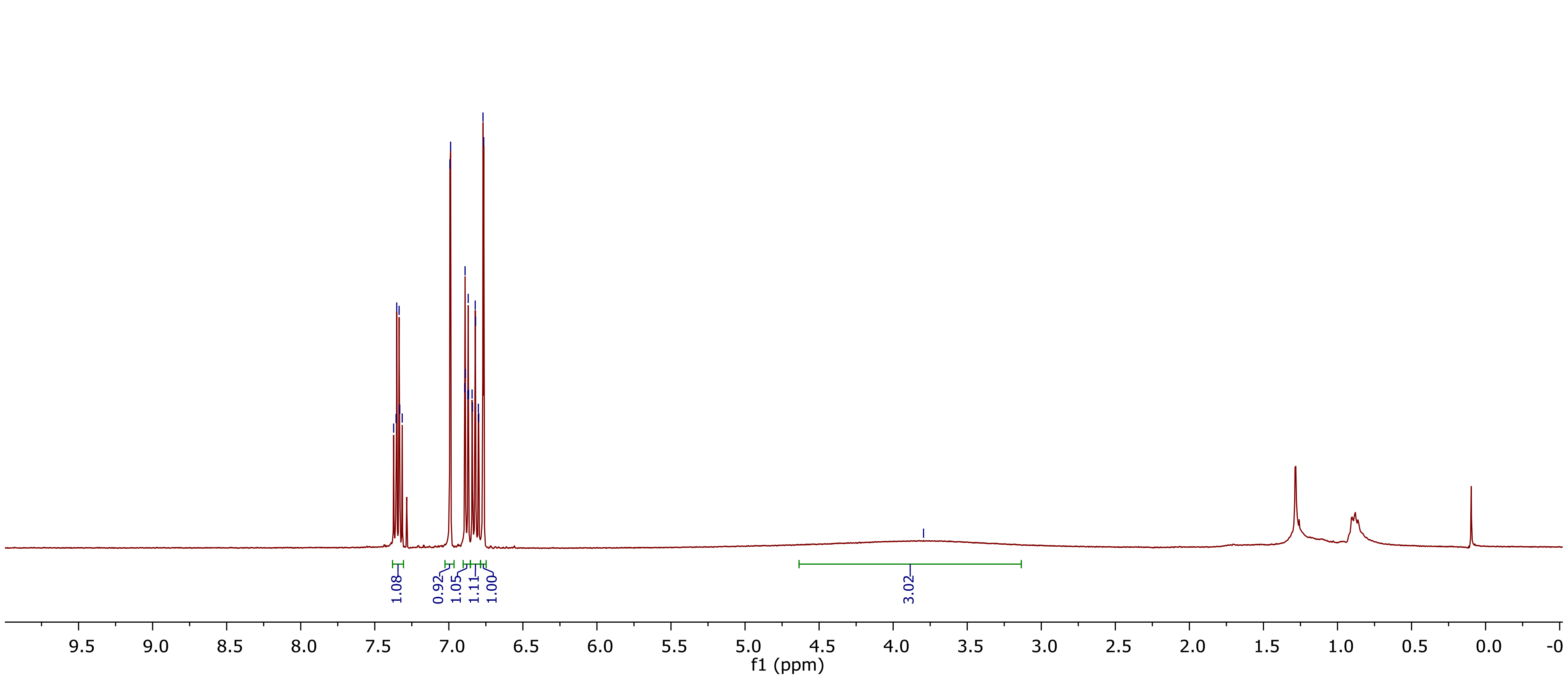

^{19}F-NMR ($CDCl_3$): *(S)*-2'-amino-4',6'-dichloro-6-fluoro-[1,1'-biphenyl]-2-ol (**3h**)

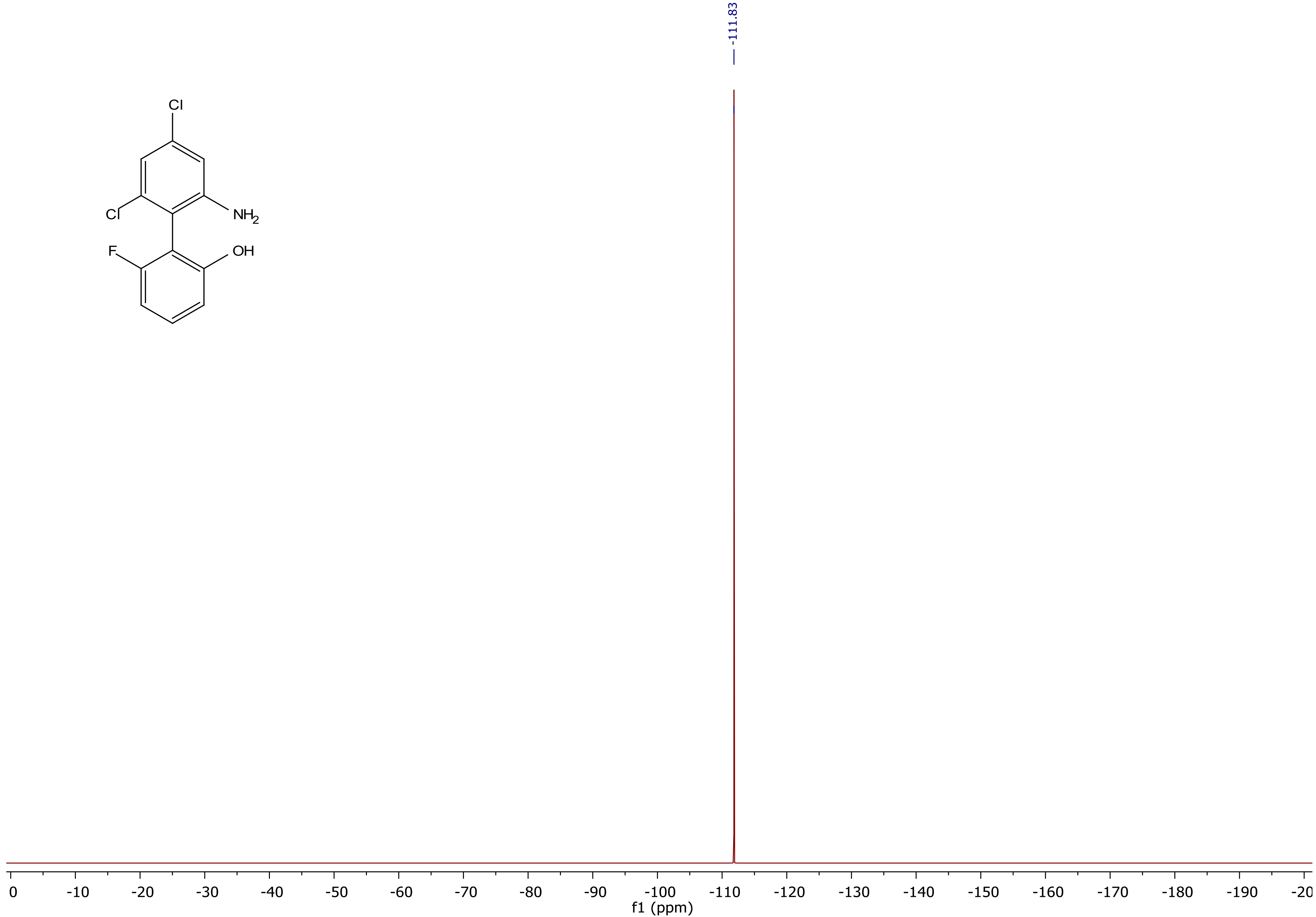

^{13}C-NMR ($CDCl_3$): *(S)*-2'-amino-4',6'-dichloro-6-fluoro-[1,1'-biphenyl]-2-ol (**3h**)

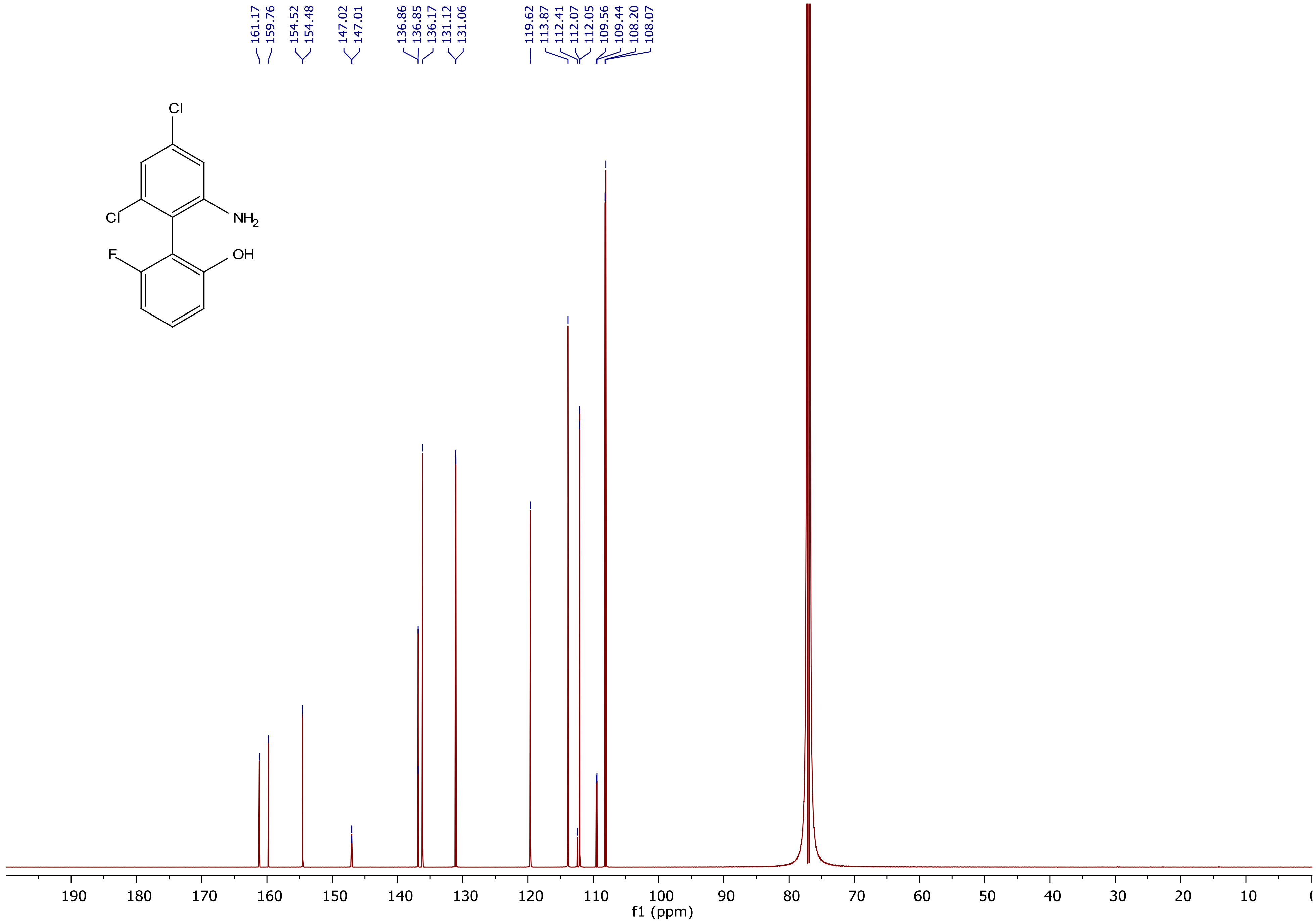

^{1}H-NMR ($CDCl_3$): *(R)*-2'-amino-6-fluoro-6'-methyl-[1,1'-biphenyl]-2-ol (**3i**)

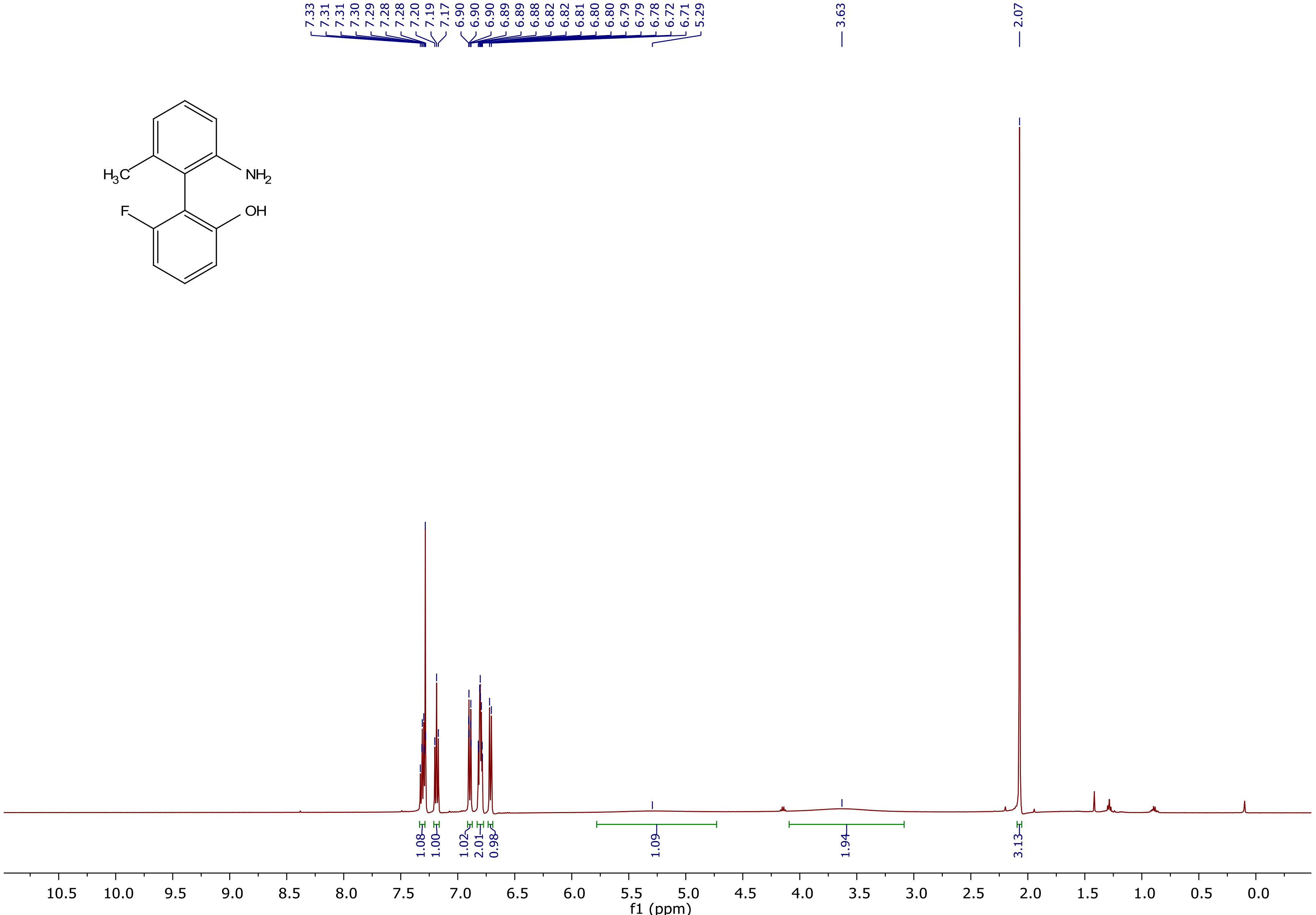

^{19}F-NMR ($CDCl_3$): *(R)*-2'-amino-6-fluoro-6'-methyl-[1,1'-biphenyl]-2-ol (**3i**)

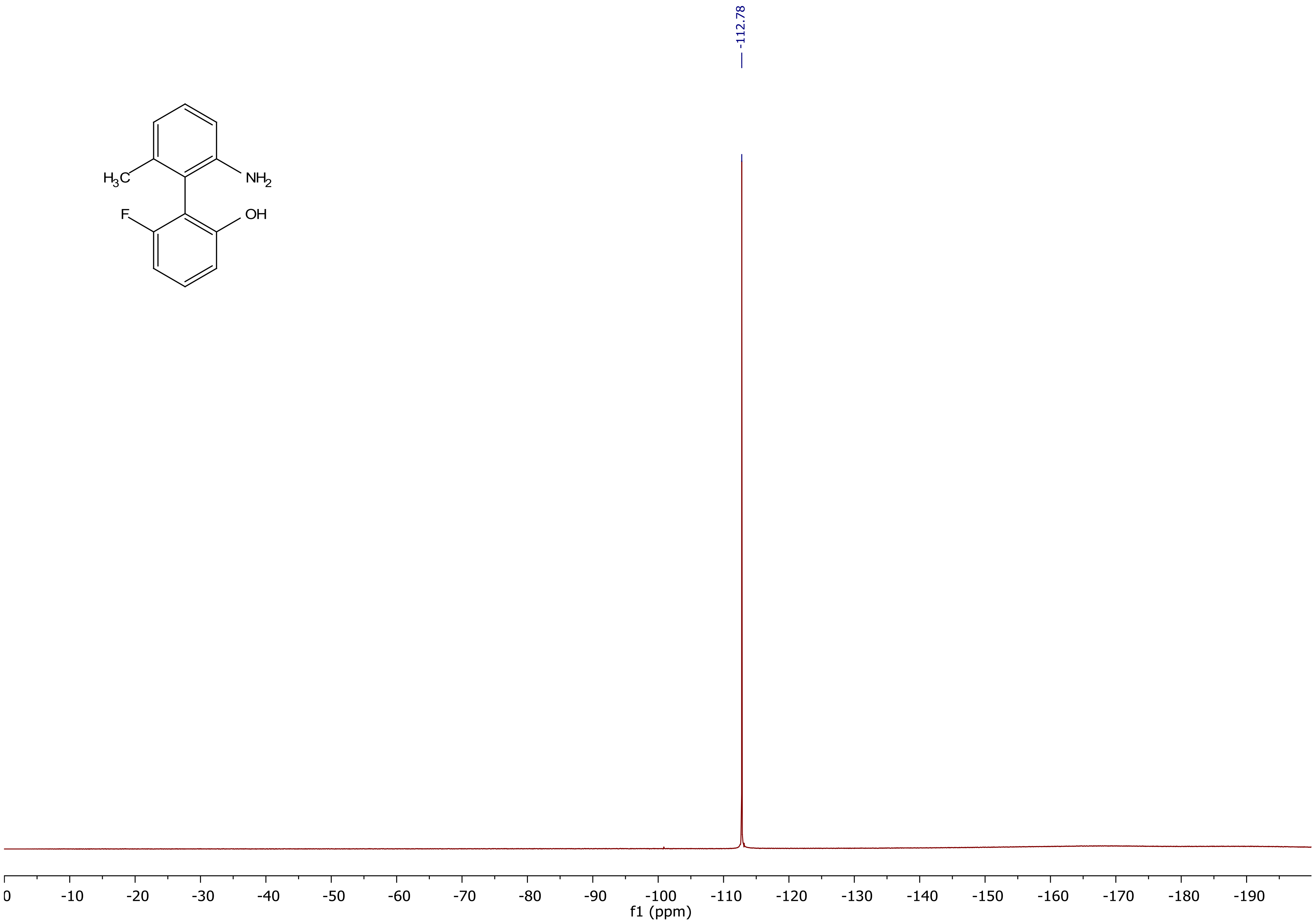

^{13}C-NMR (CDCl$_3$): *(R)*-2'-amino-6-fluoro-6'-methyl-[1,1'-biphenyl]-2-ol (**3i**)

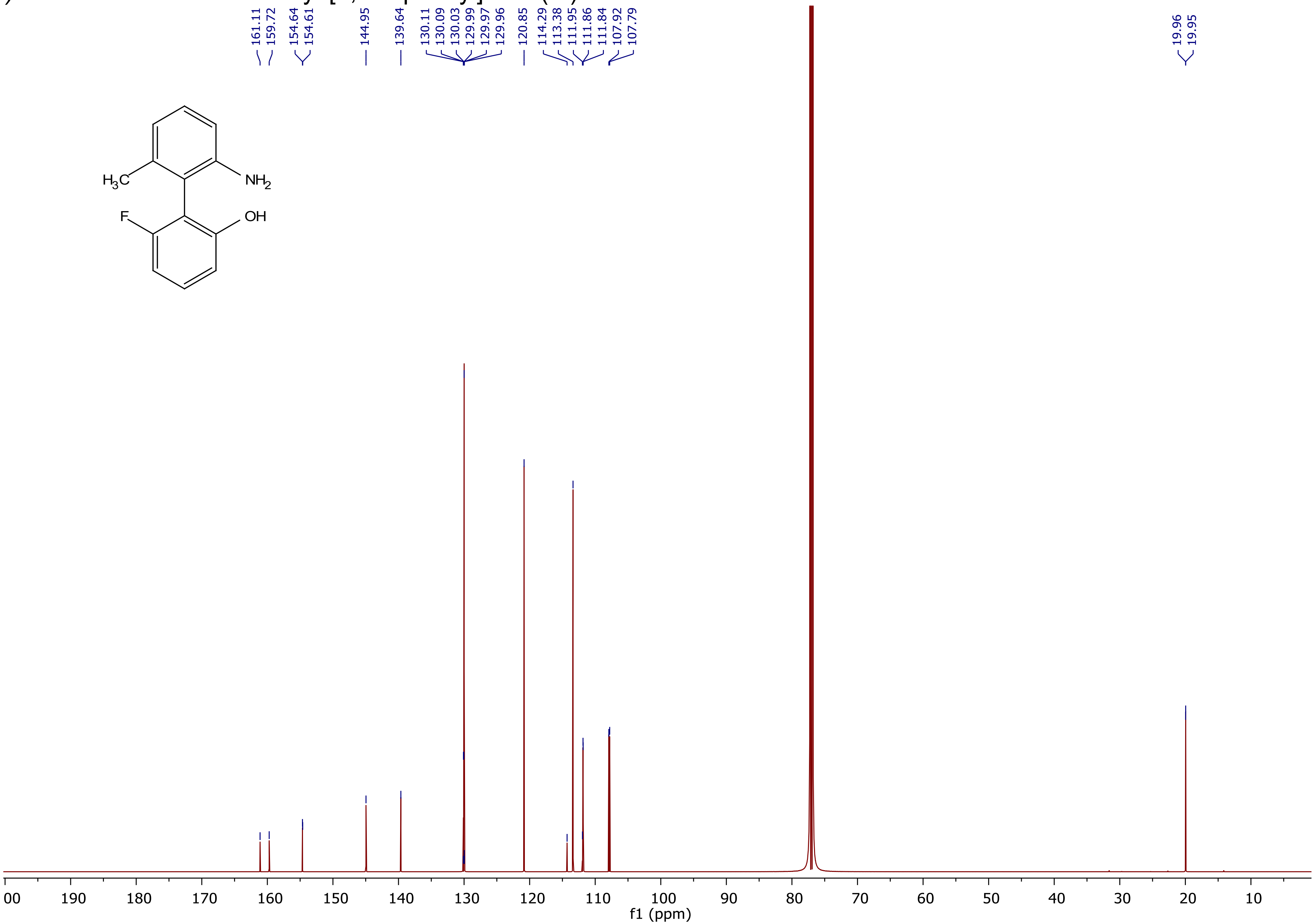

¹H-NMR (MeOD): *(R)*-2-(2-amino-5,6,7,8-tetrahydronaphthalen-1-yl)-3-fluorophenol (**3j**)

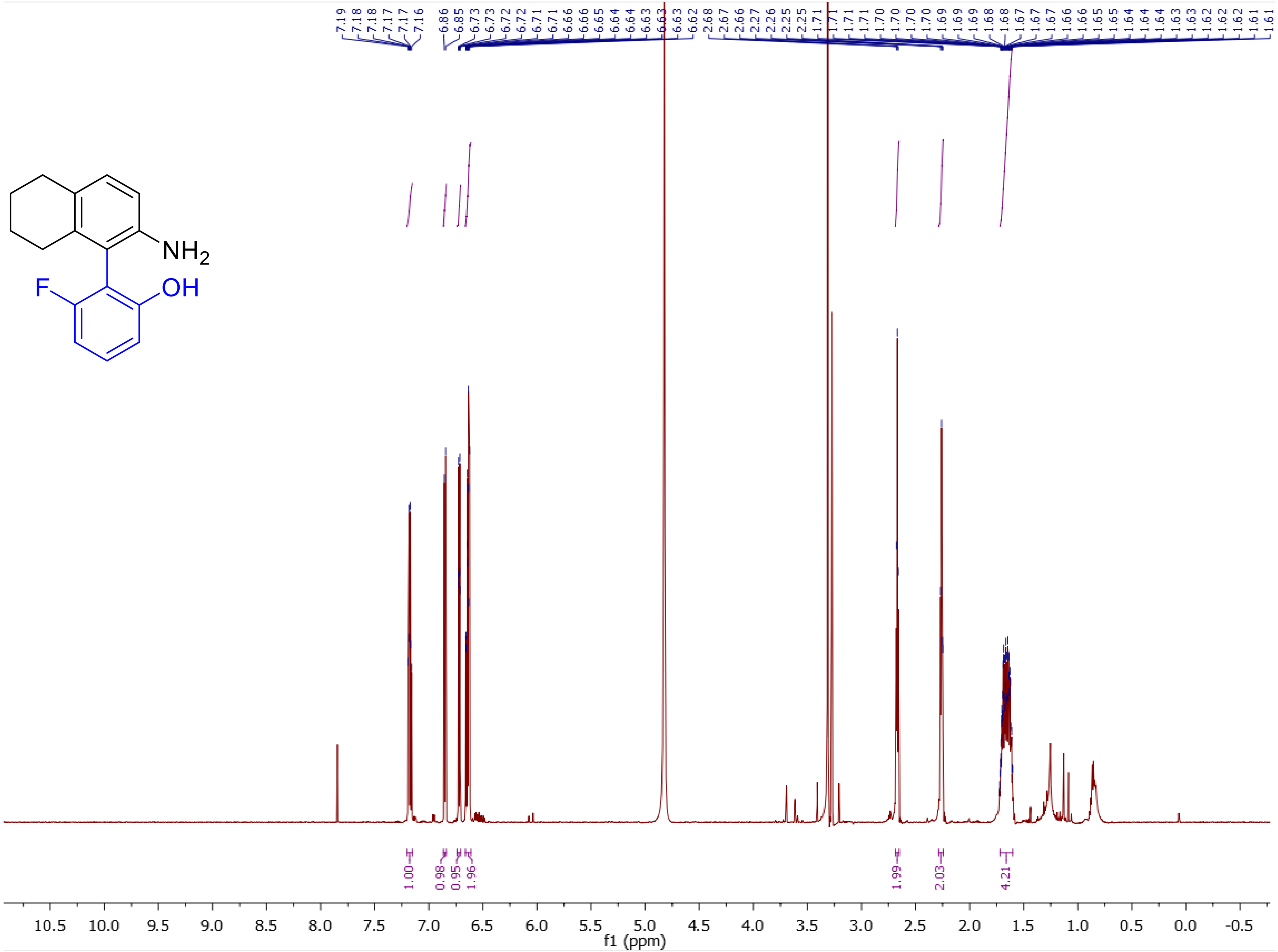

^{19}F-NMR (CDCl$_3$): *(R)*-2-(2-amino-5,6,7,8-tetrahydronaphthalen-1-yl)-3-fluorophenol (**3j**)

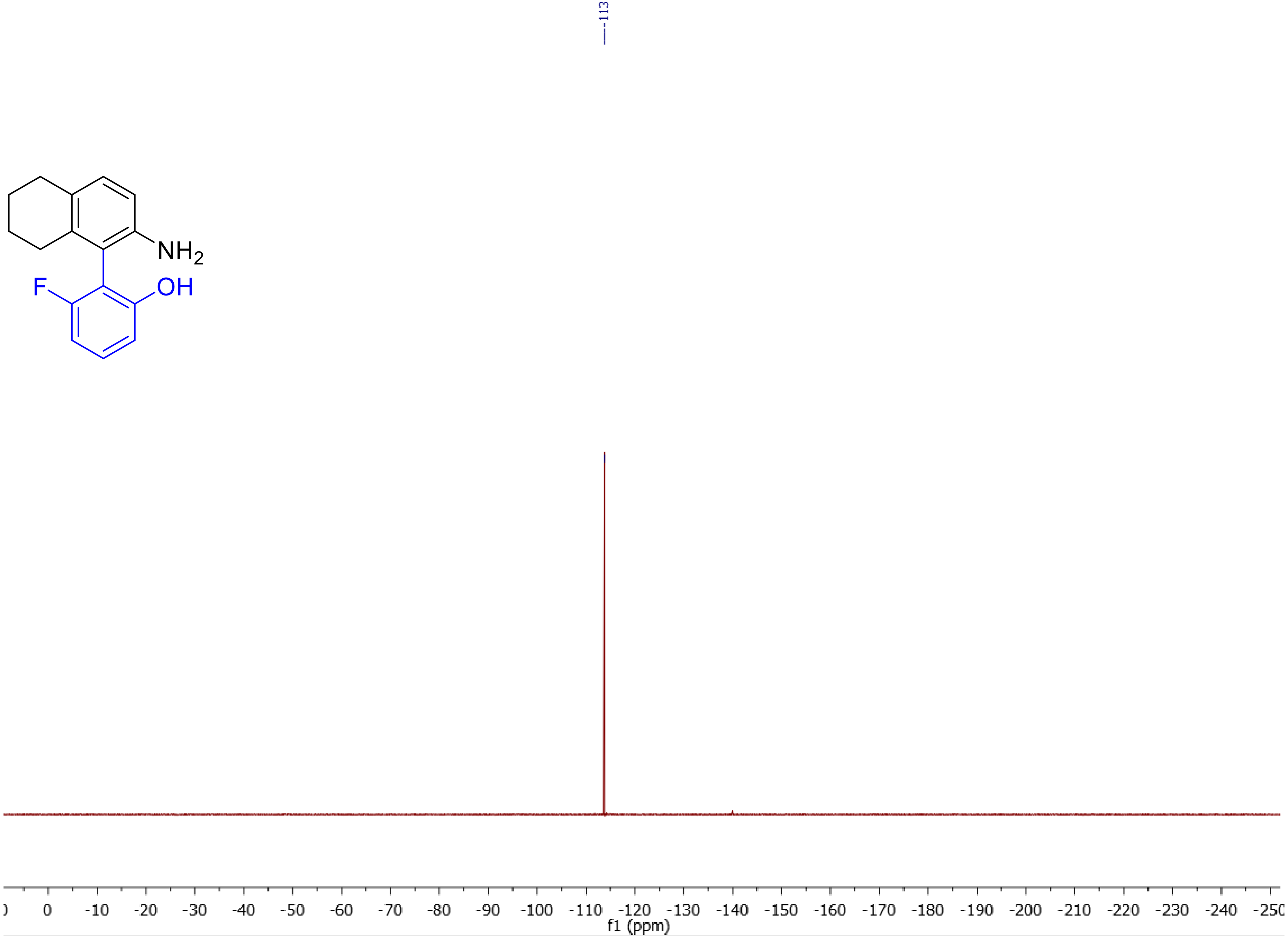

^{13}C-NMR (MeOD): *(R)*-2-(2-amino-5,6,7,8-tetrahydronaphthalen-1-yl)-3-fluorophenol (**3j**)

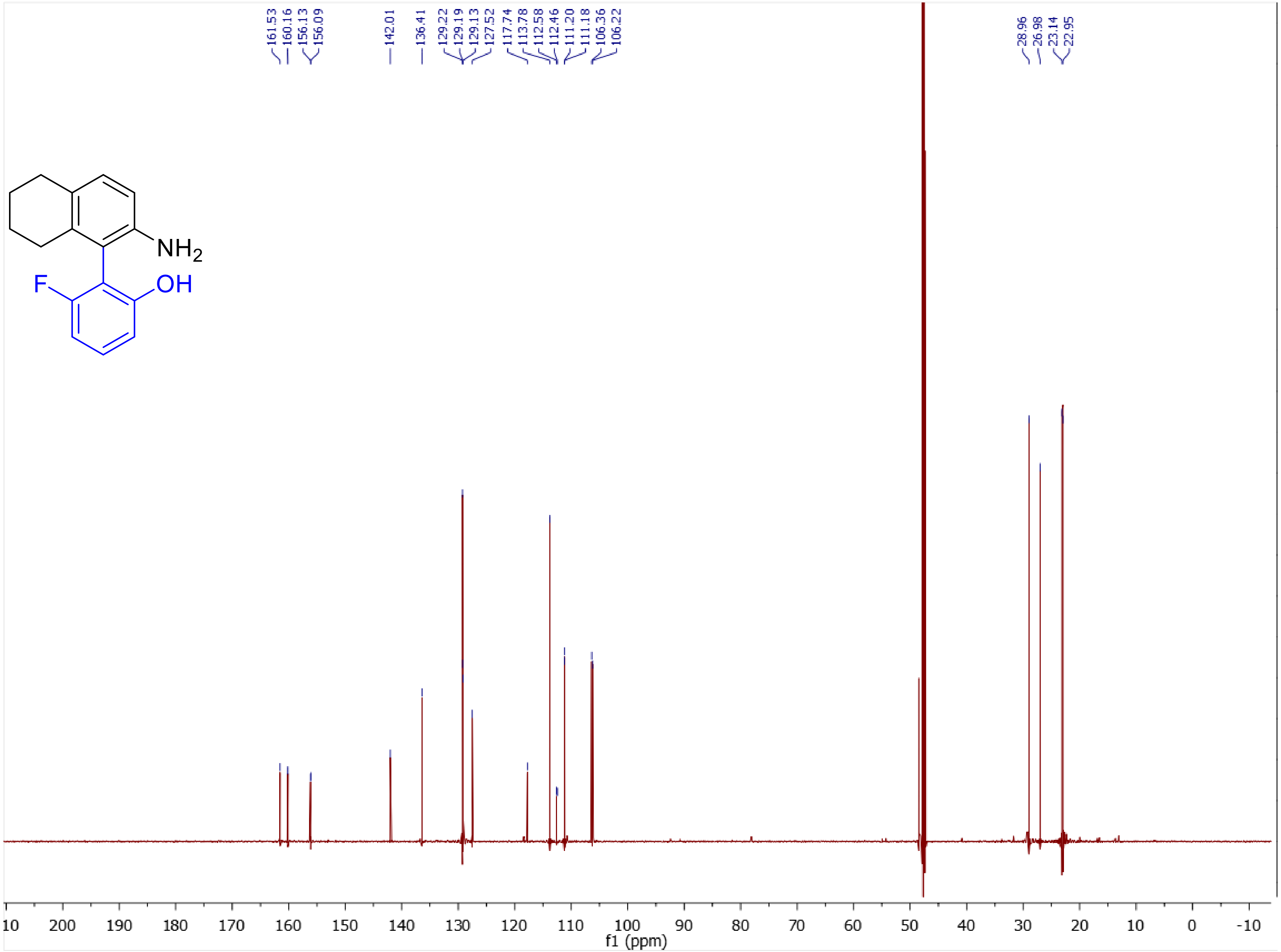

^{1}H-NMR (MeOD): *(R)-6'-amino-3'-chloro-6-fluoro-2'-methyl-[1,1'-biphenyl]-2-ol* (**3k**)

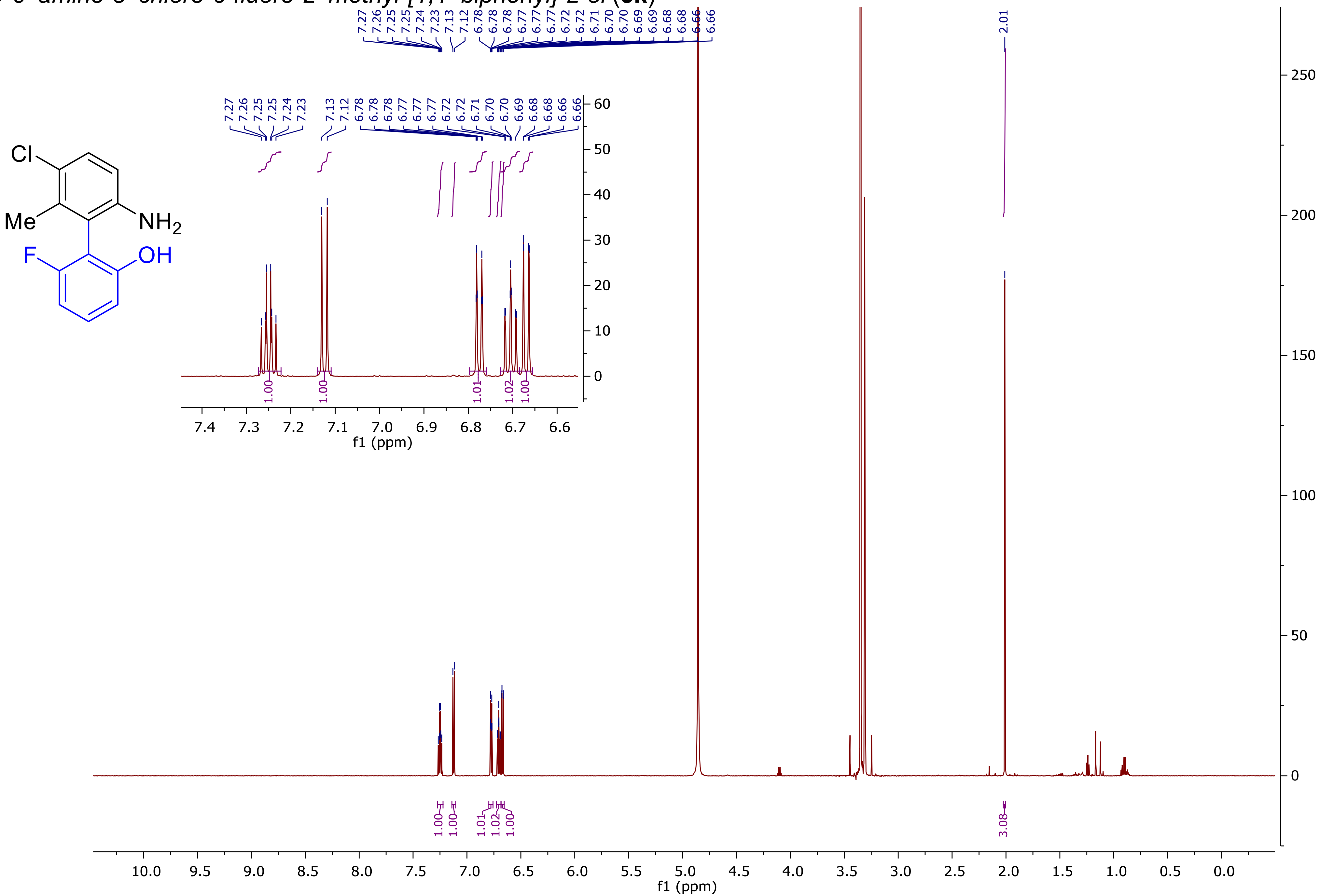

^{19}F-NMR ($CDCl_3$): *(R)-6'-amino-3'-chloro-6-fluoro-2'-methyl-[1,1'-biphenyl]-2-ol* (**3k**)

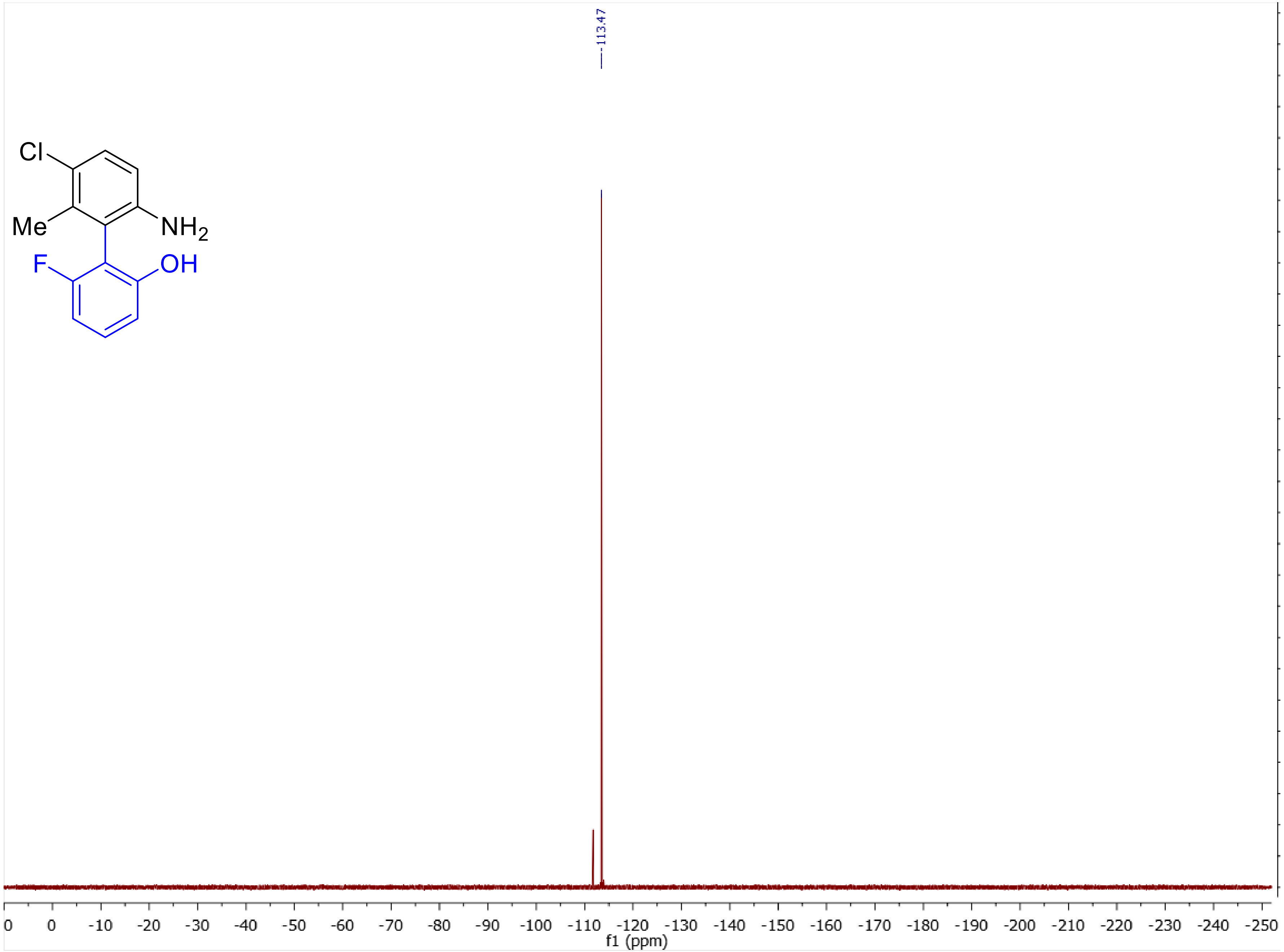

^{13}**C-NMR** (MeOD): *(R)-6'-amino-3'-chloro-6-fluoro-2'-methyl-[1,1'-biphenyl]-2-ol* (**3k**)

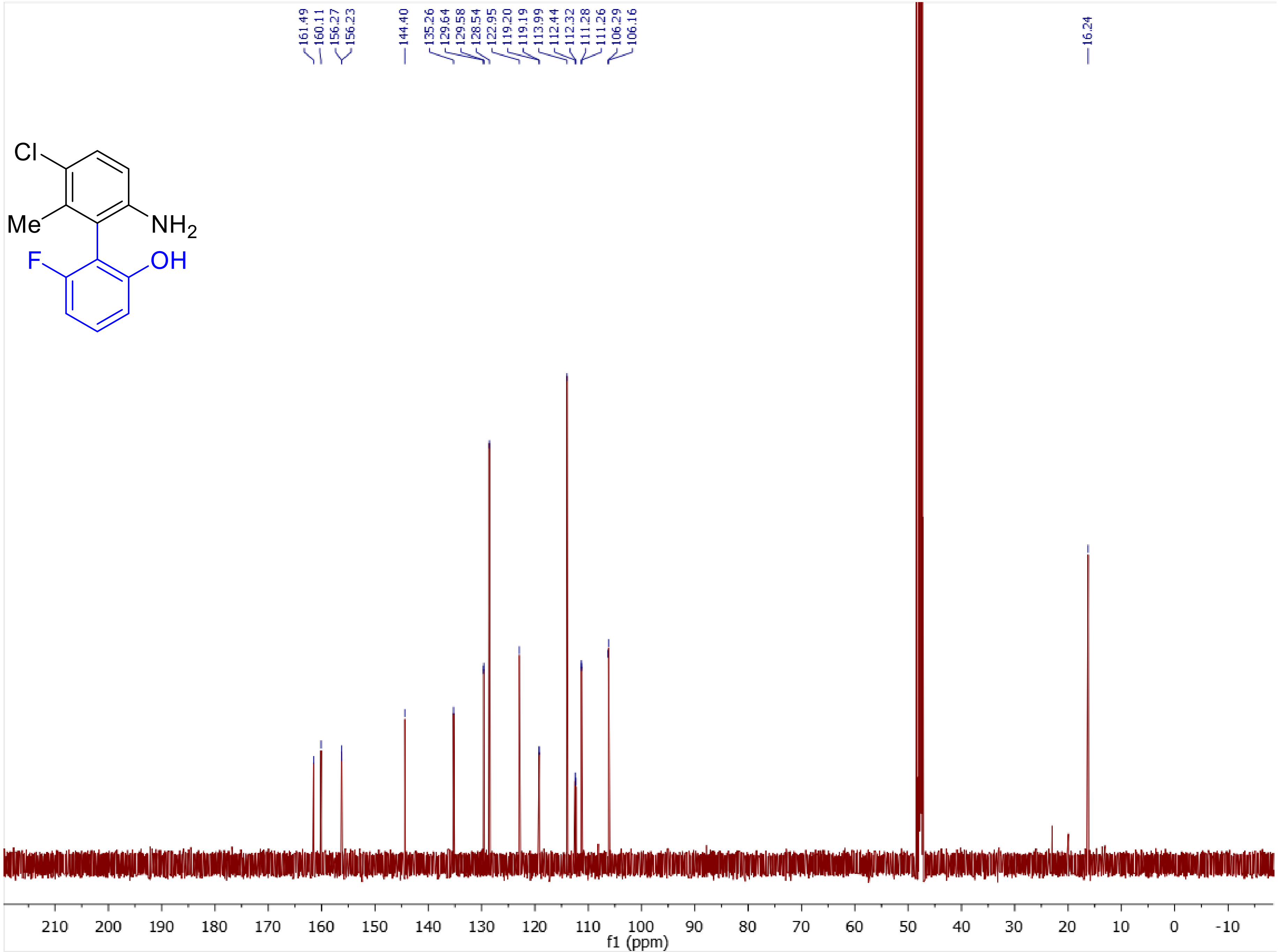

^{1}H-NMR ($CDCl_3$): *(S)*-2'-amino-4',6'-dichloro-5,6-difluoro-[1,1'-biphenyl]-2-ol (**3l**)

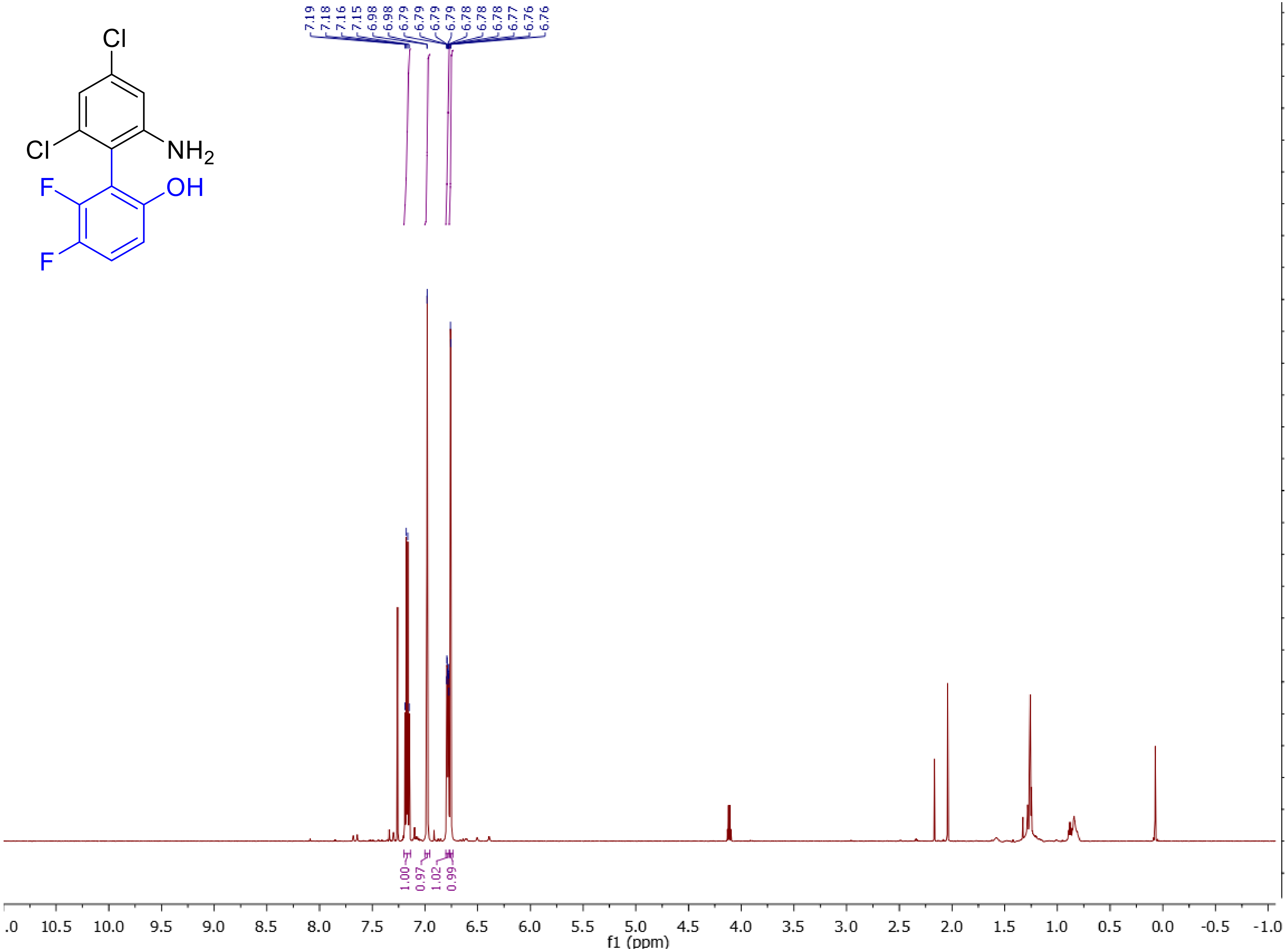

^{19}F-NMR ($CDCl_3$): *(S)*-2'-amino-4',6'-dichloro-5,6-difluoro-[1,1'-biphenyl]-2-ol (**3l**)

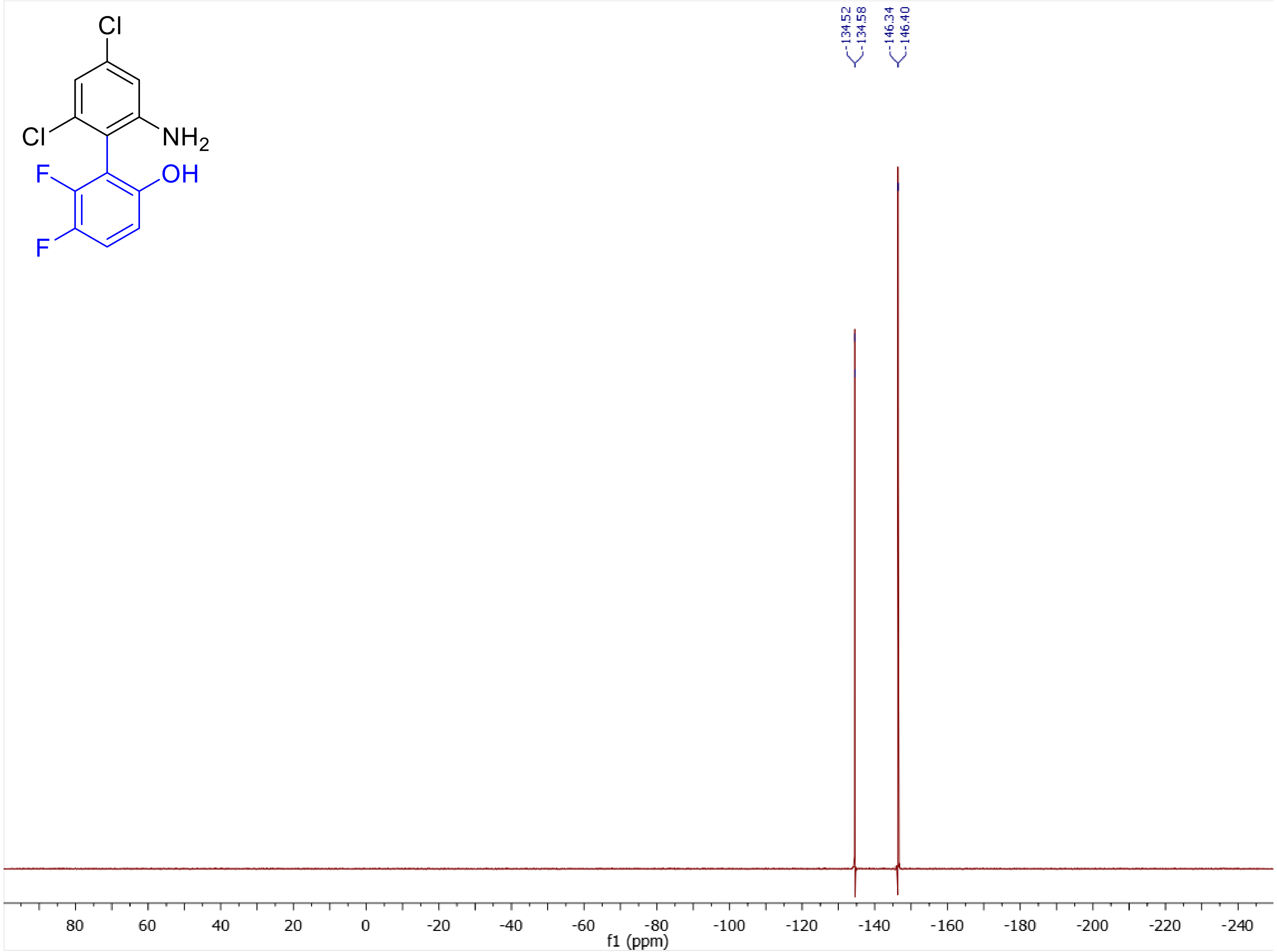

^{13}C-NMR ($CDCl_3$): *(S)*-2'-amino-4',6'-dichloro-5,6-difluoro-[1,1'-biphenyl]-2-ol (**3l**)

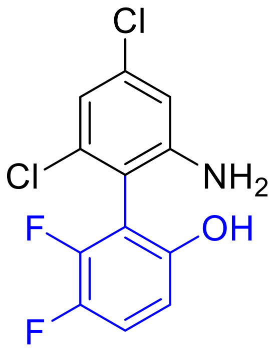

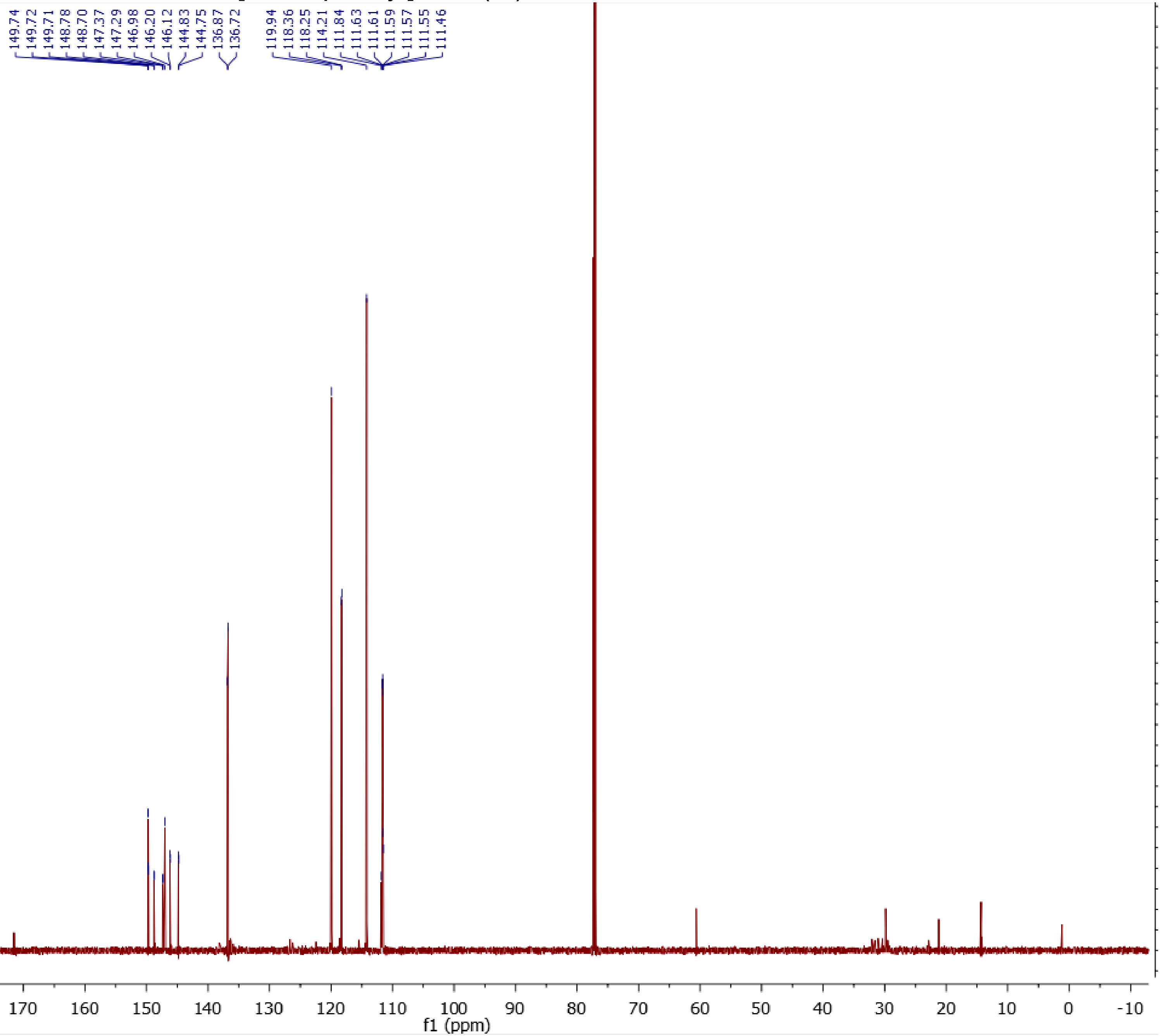

¹H-NMR (Acetone-d6): *(S)*-2'-amino-6'-chloro-5,6-difluoro-[1,1'-biphenyl]-2-ol (**3m**)

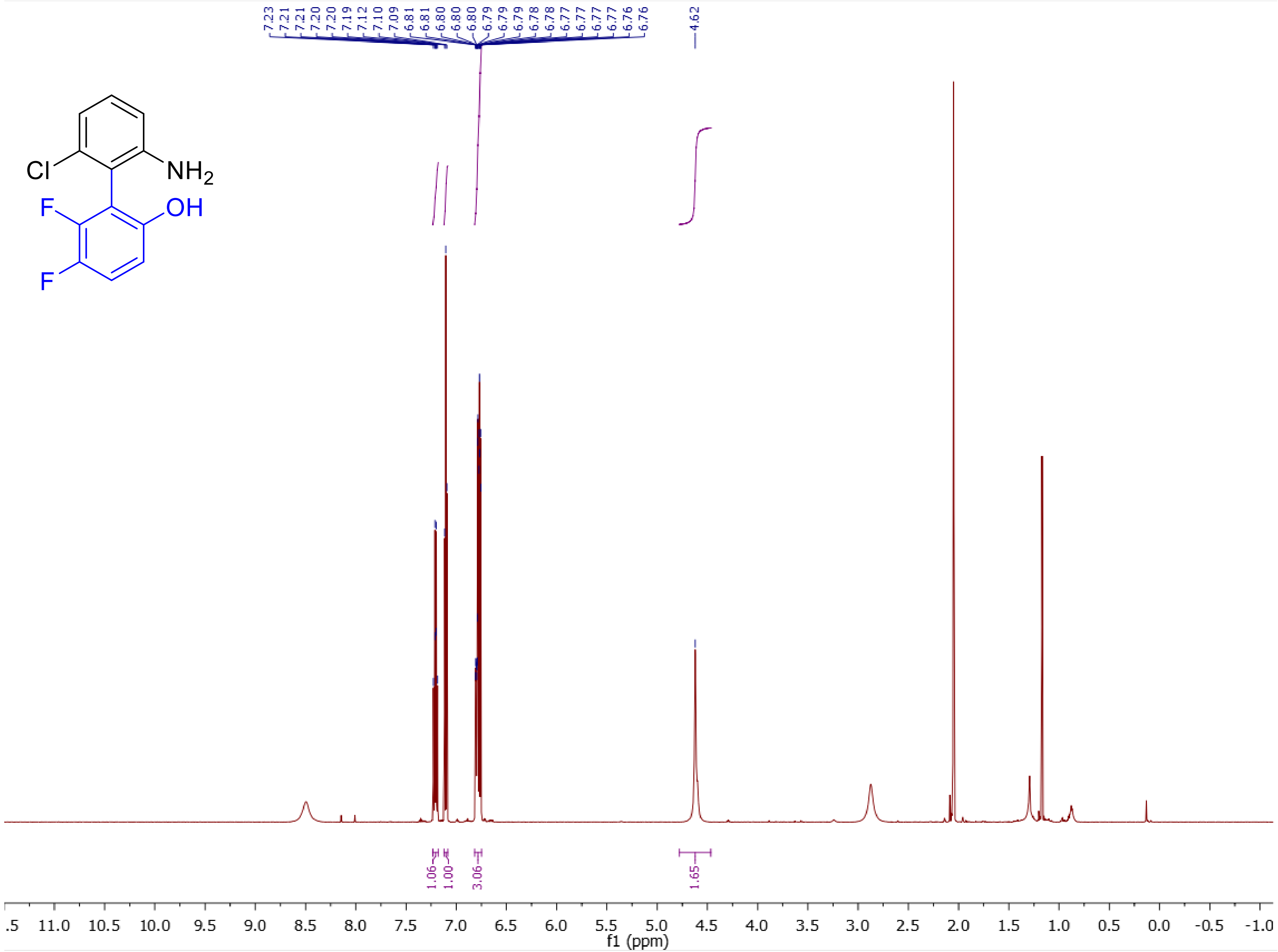

^{19}F-NMR (Acetone-d6): *(S)*-2'-amino-6'-chloro-5,6-difluoro-[1,1'-biphenyl]-2-ol (**3m**)

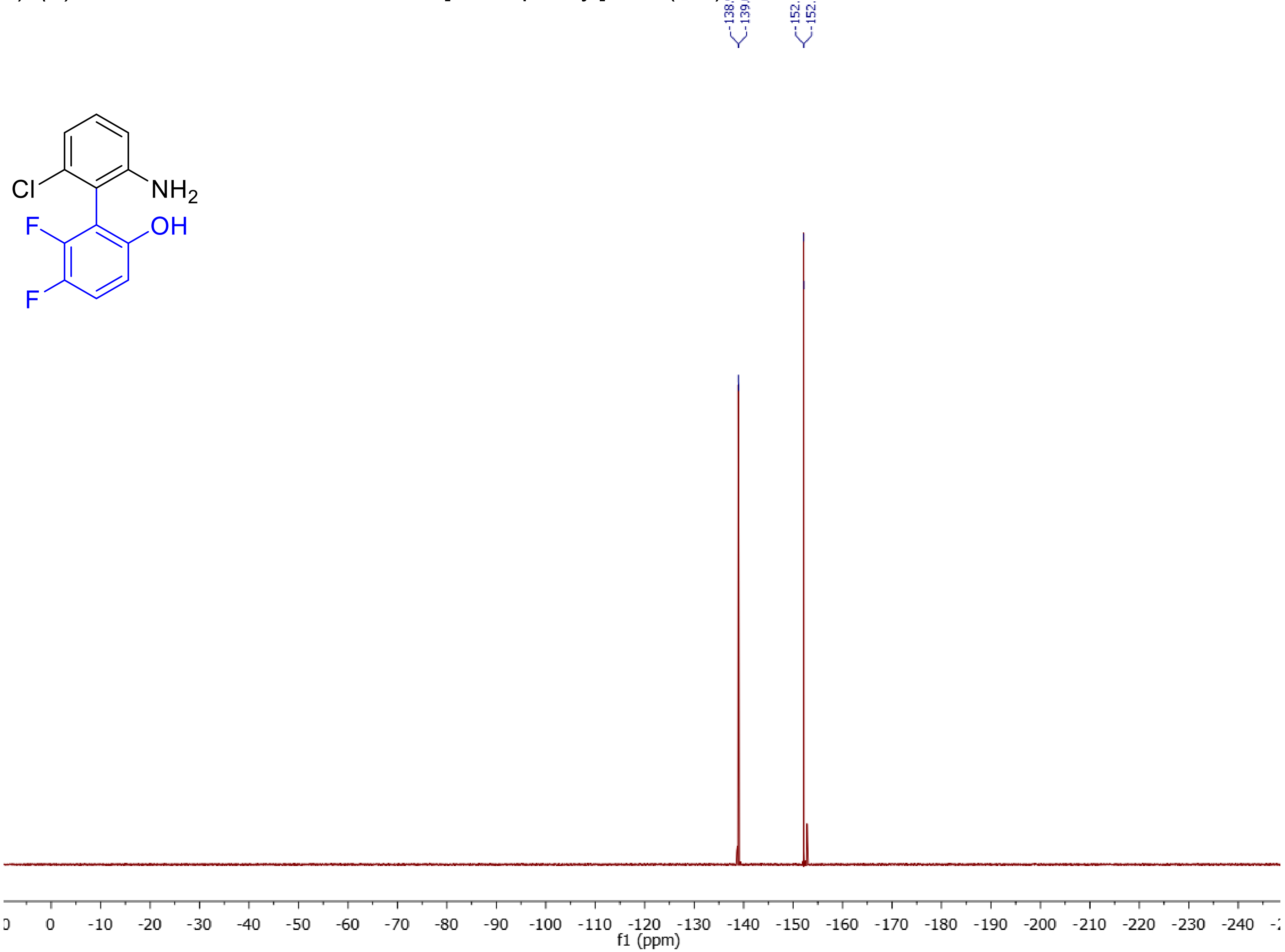

^{13}C-NMR (Acetone-d6): *(S)*-2'-amino-6'-chloro-5,6-difluoro-[1,1'-biphenyl]-2-ol (**3m**)

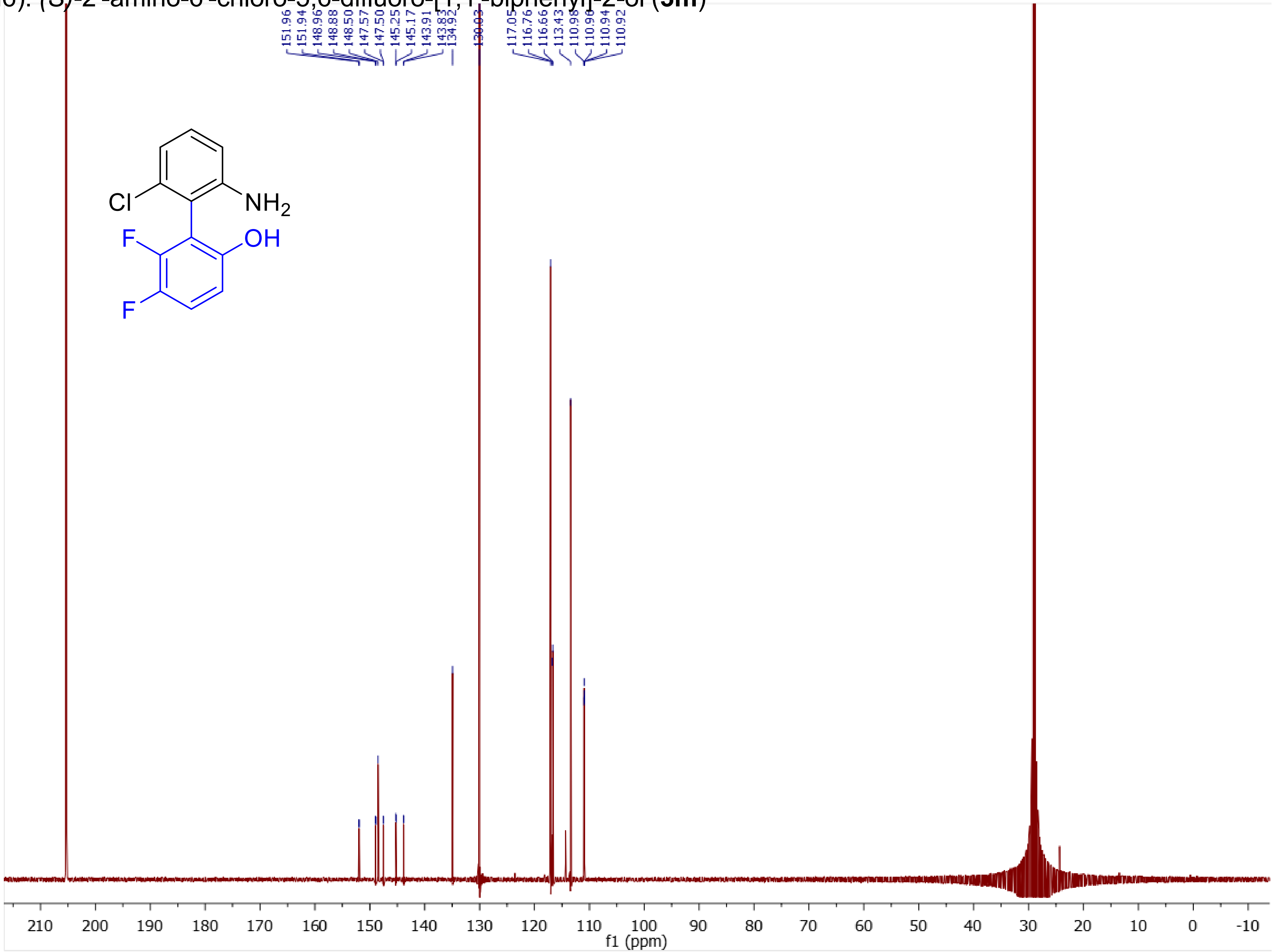

^{1}H-NMR ($CDCl_3$): (R)-2'-amino-5,6-difluoro-6'-methyl-[1,1'-biphenyl]-2-ol (**3n**)

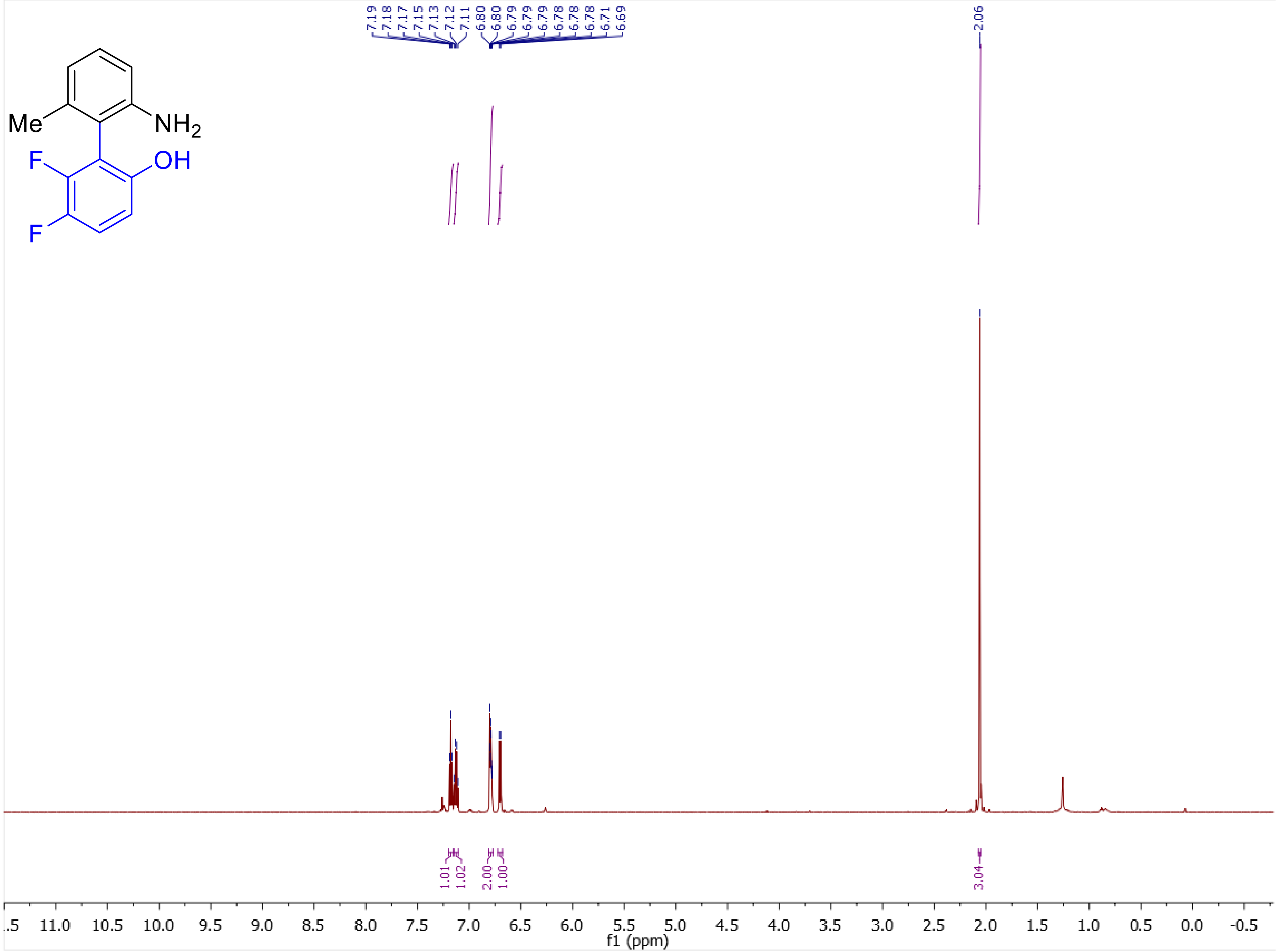

^{19}F-NMR ($CDCl_3$): (R)-2'-amino-5,6-difluoro-6'-methyl-[1,1'-biphenyl]-2-ol (**3n**)

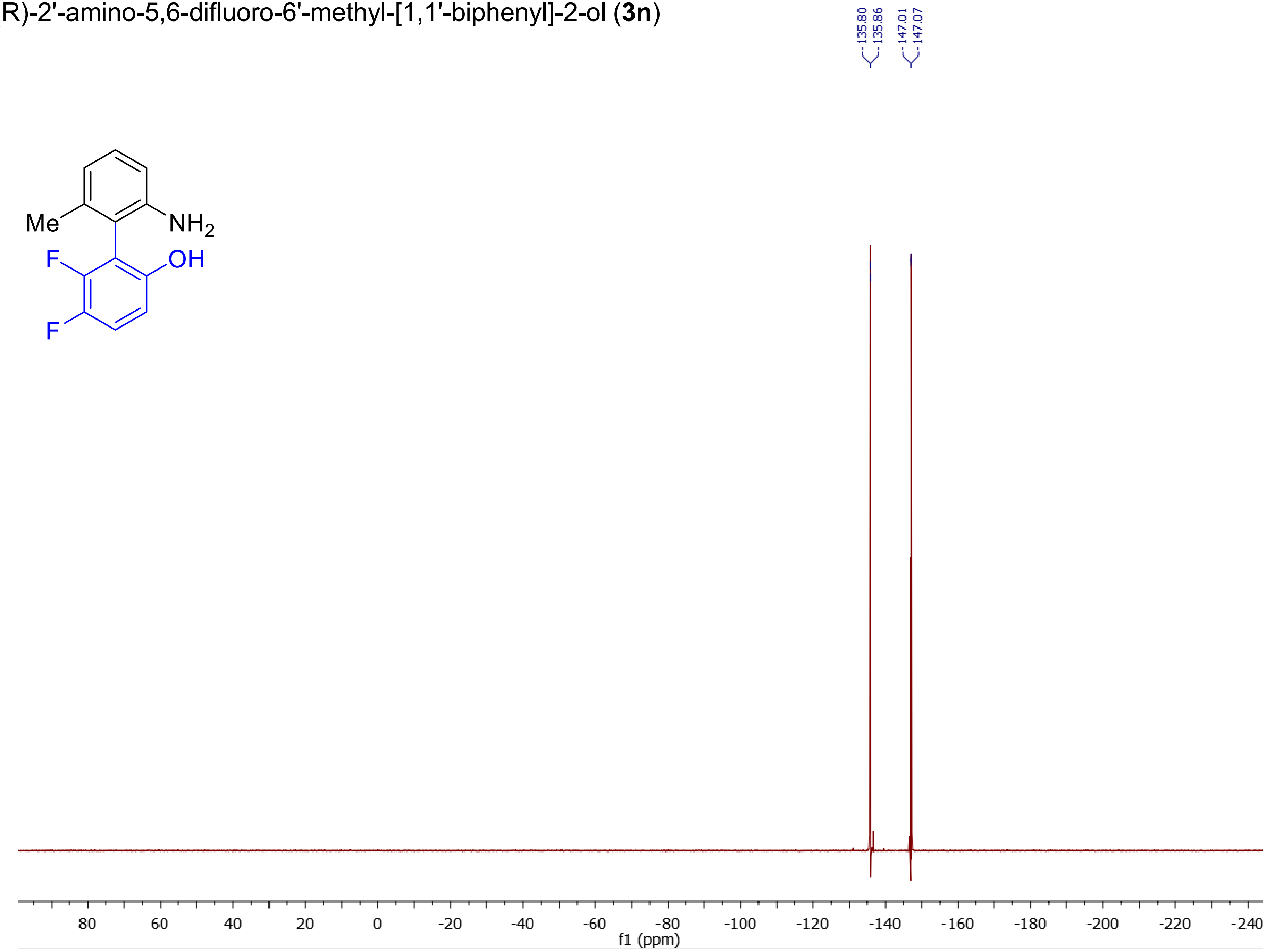

^{13}C-NMR ($CDCl_3$): (R)-2'-amino-5,6-difluoro-6'-methyl-[1,1'-biphenyl]-2-ol (**3n**)

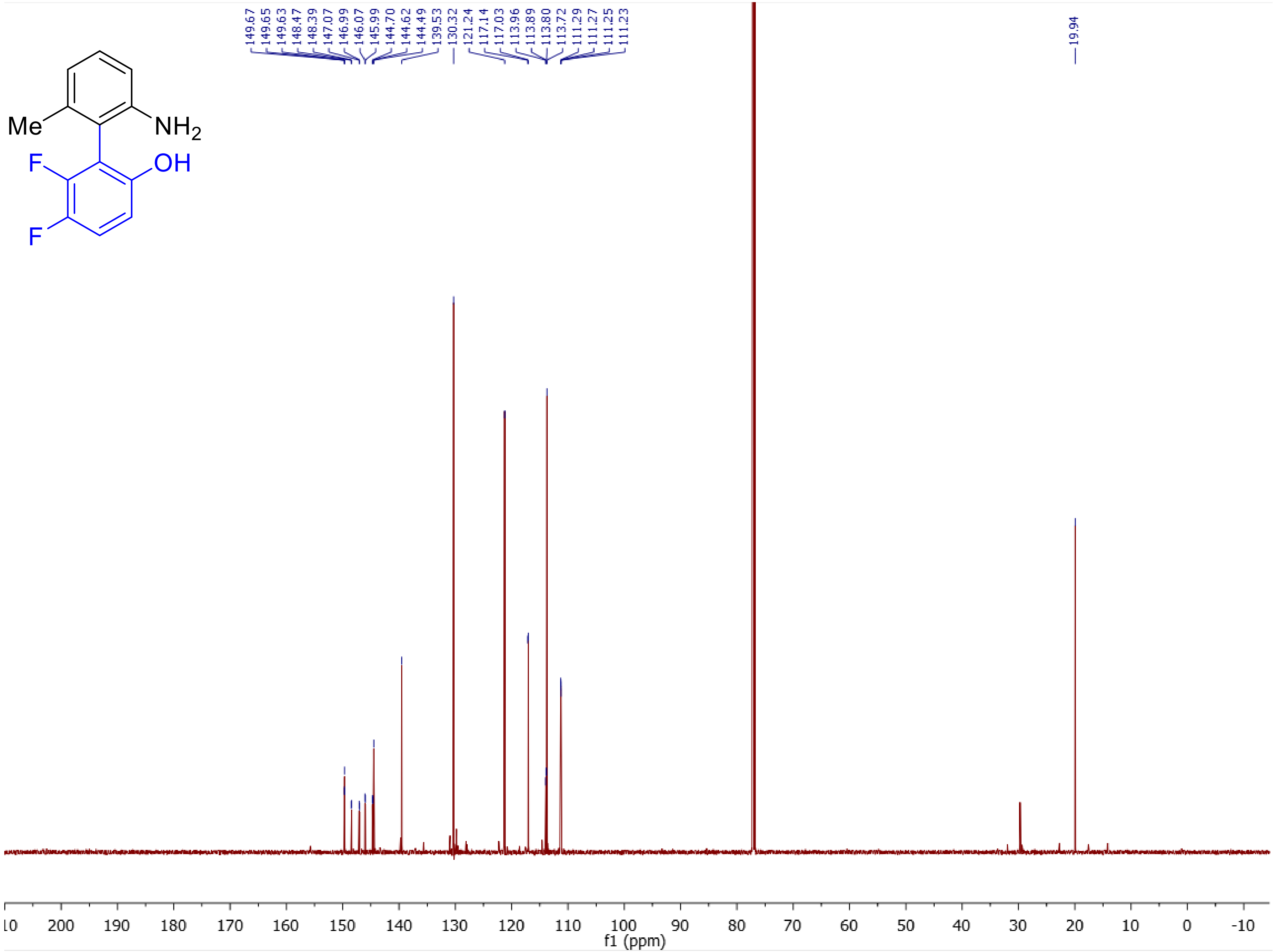

^{1}H-NMR (MeOD): *(R)*-2-(2-amino-5,6,7,8-tetrahydronaphthalen-1-yl)-3,4-difluorophenol (**3o**)

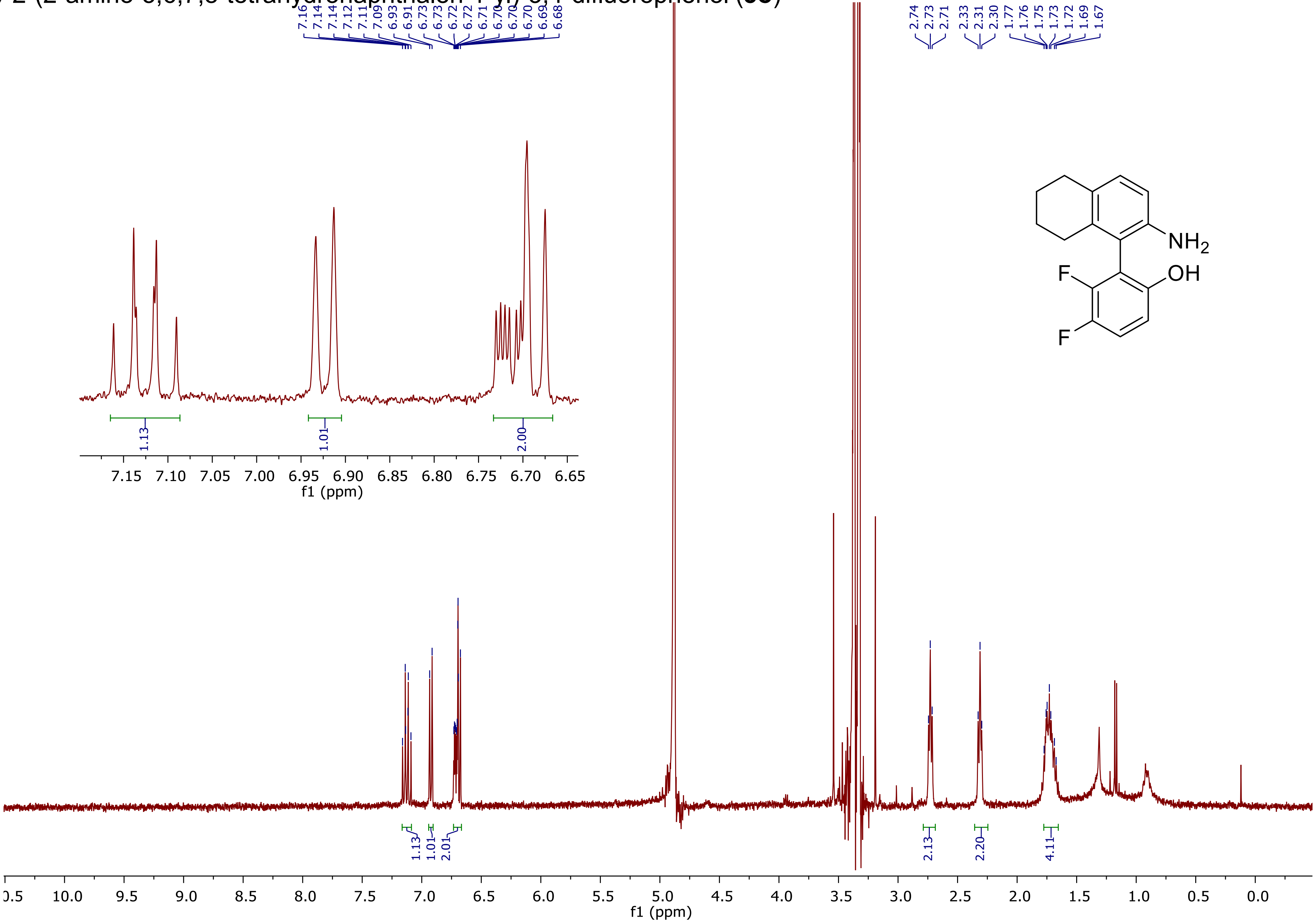

^{19}F-NMR (CDCl$_3$): *(R)*-2-(2-amino-5,6,7,8-tetrahydronaphthalen-1-yl)-3,4-difluorophenol (**3o**)

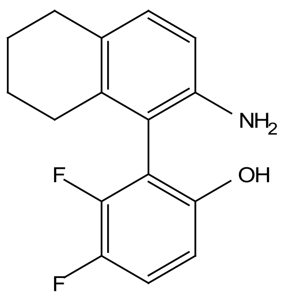

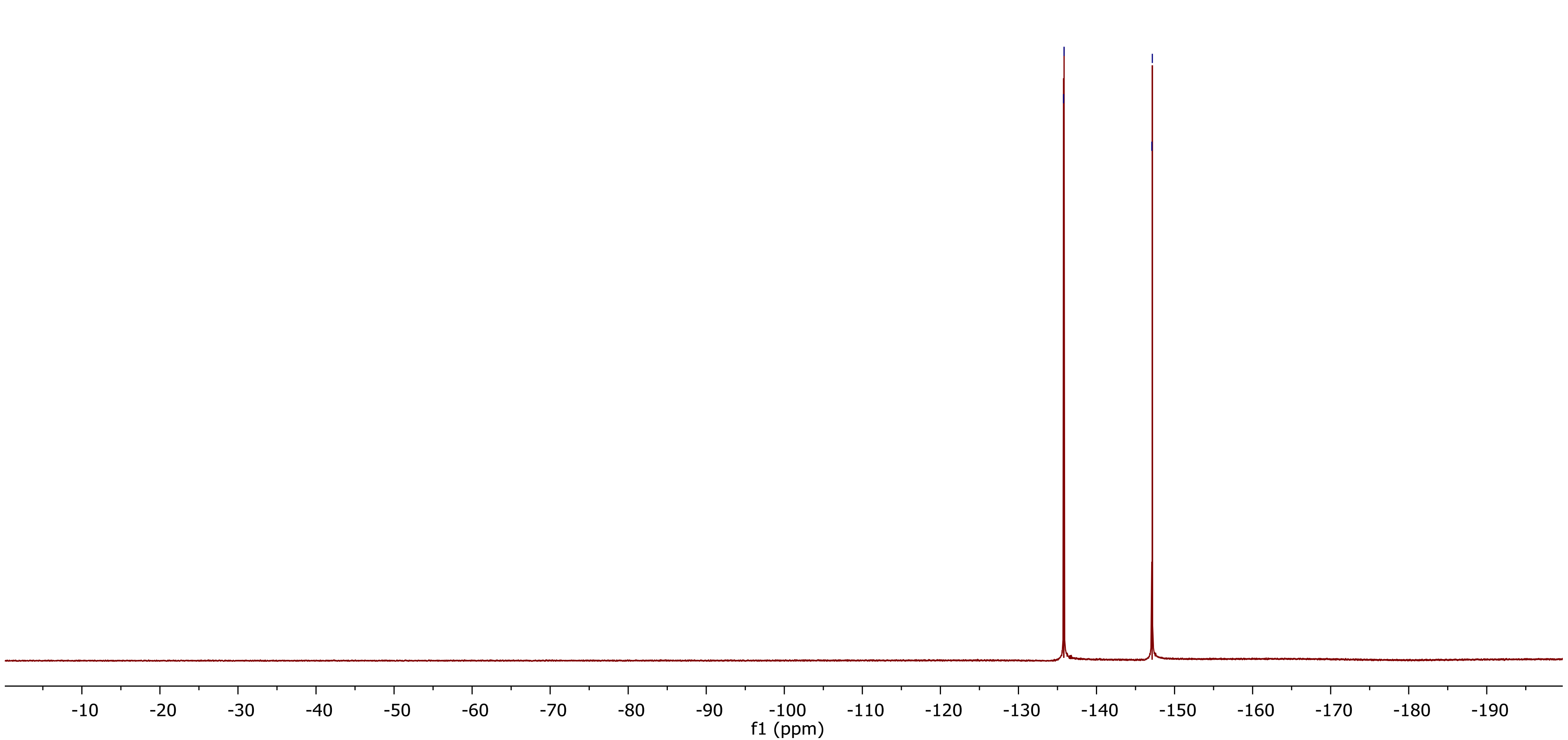

^{13}C-NMR (CDCl$_3$): *(R)*-2'-amino-6-chloro-6'-fluoro-[1,1'-biphenyl]-2-ol (**3o**)

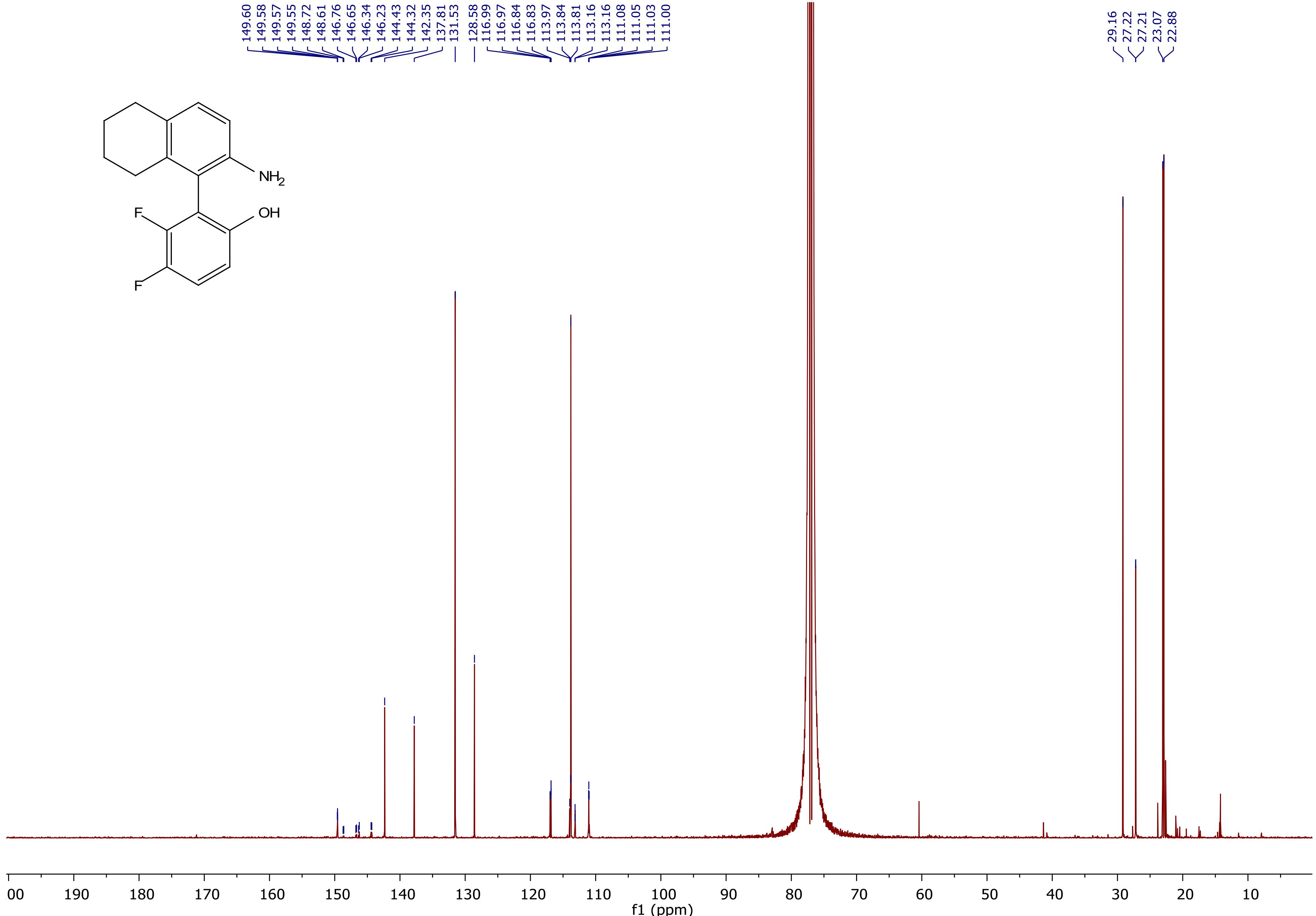

^{1}H-NMR ($CDCl_3$): *(R)*-2'-amino-6'-fluoro-6-methyl-[1,1'-biphenyl]-2-ol (**3p**)

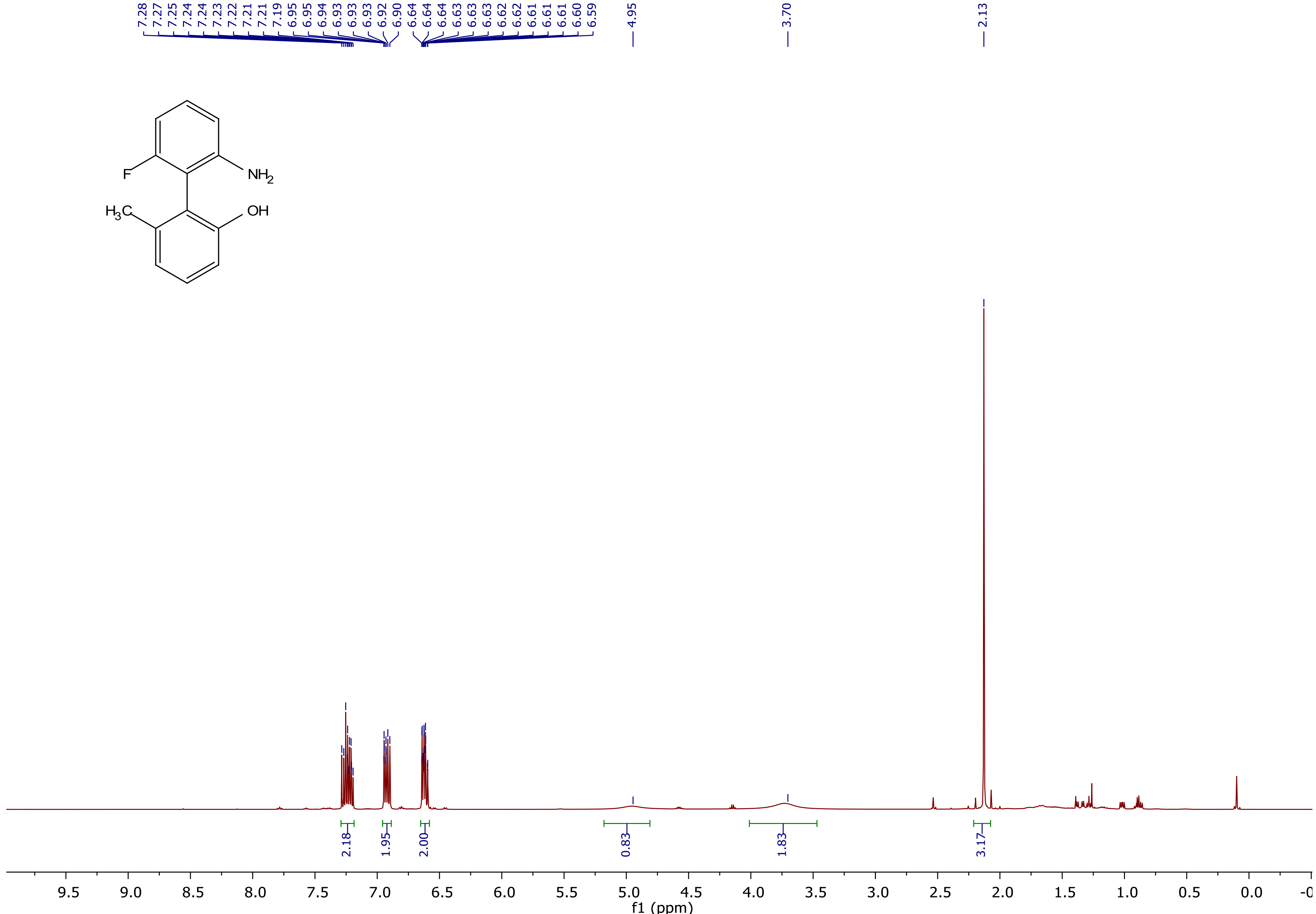

^{19}F-NMR ($CDCl_3$): *(R)*-2'-amino-6'-fluoro-6-methyl-[1,1'-biphenyl]-2-ol (**3p**)

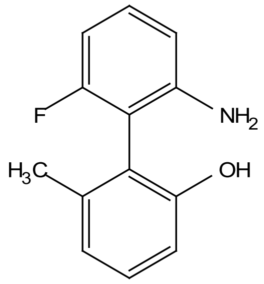

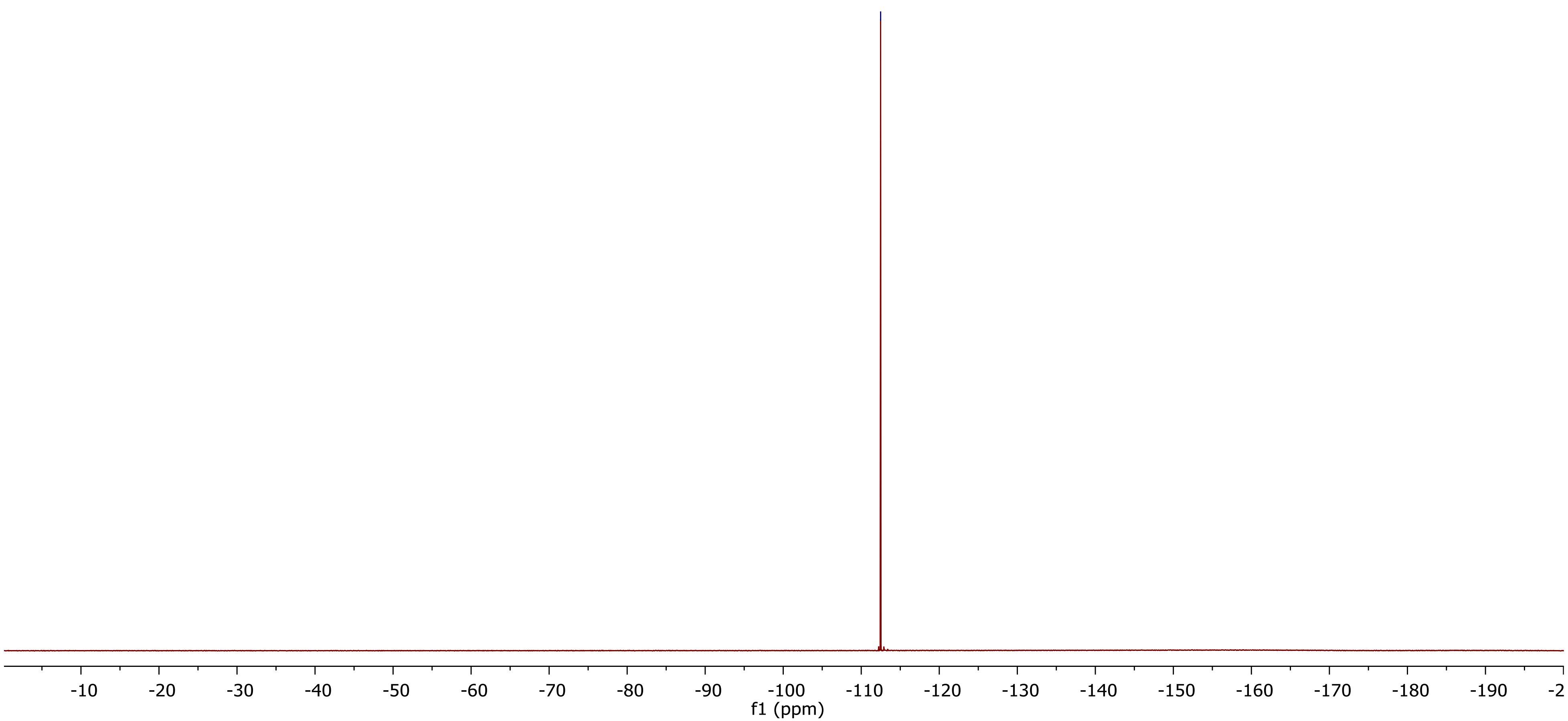

^{13}C-NMR ($CDCl_3$): *(R)*-2'-amino-6'-fluoro-6-methyl-[1,1'-biphenyl]-2-ol (**3p**)

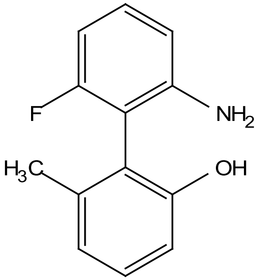

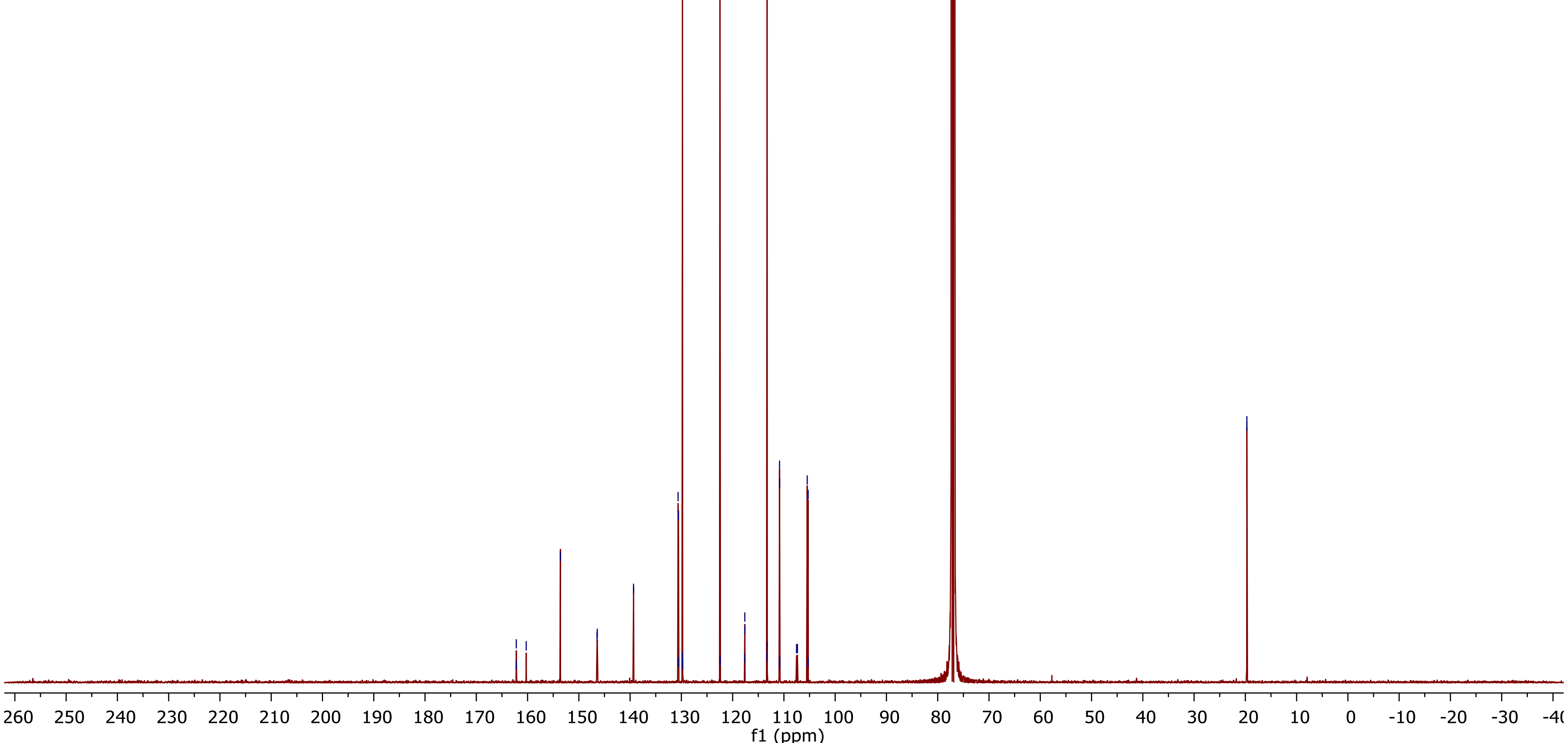

^{1}H-NMR ($CDCl_3$): *(R)*-1-(2-amino-6-fluorophenyl)-5,6,7,8-tetrahydronaphthalen-2-ol (**3q**)

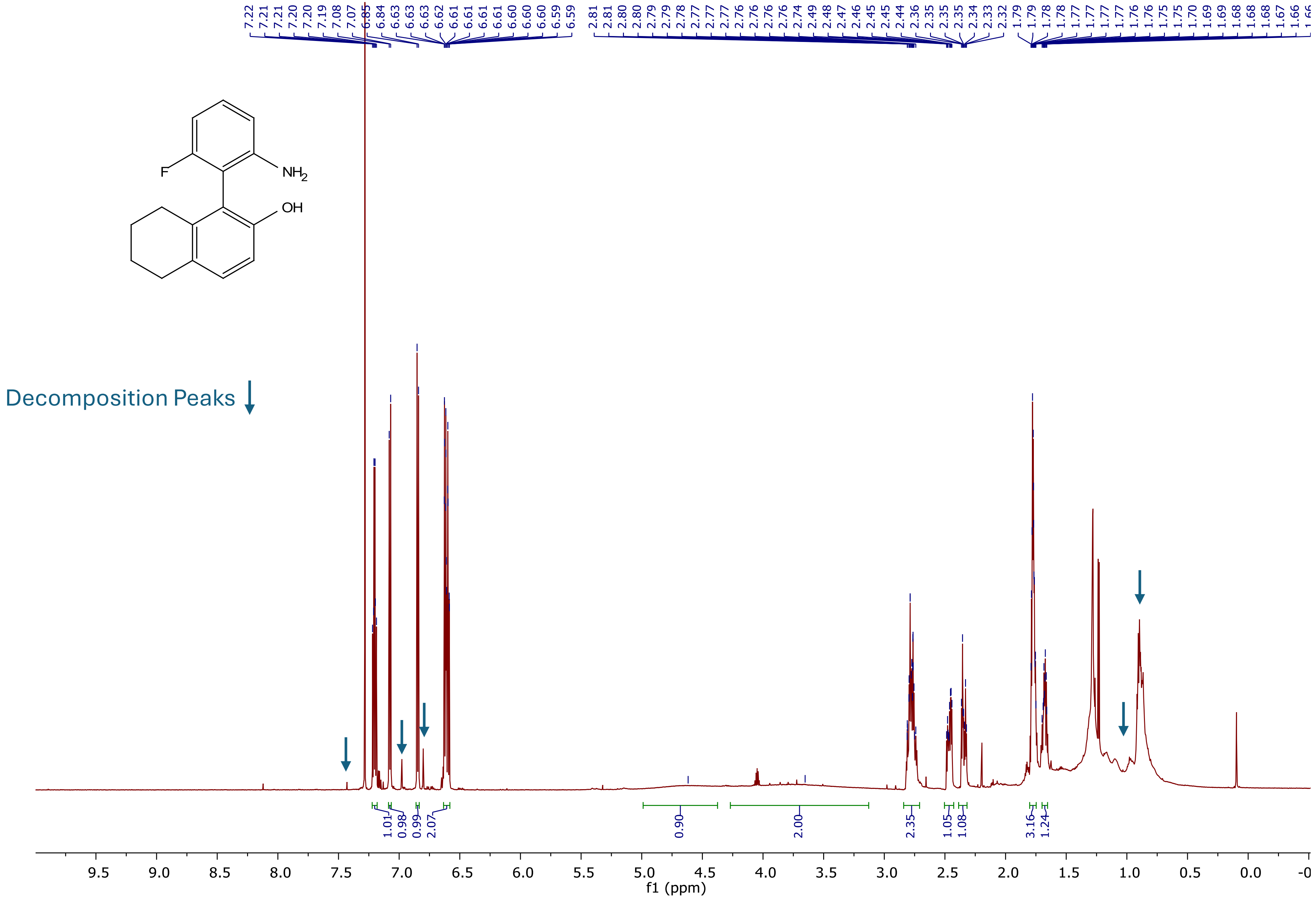

^{19}F-NMR (CDCl$_3$): *(R)*-1-(2-amino-6-fluorophenyl)-5,6,7,8-tetrahydronaphthalen-2-ol (**3q**)

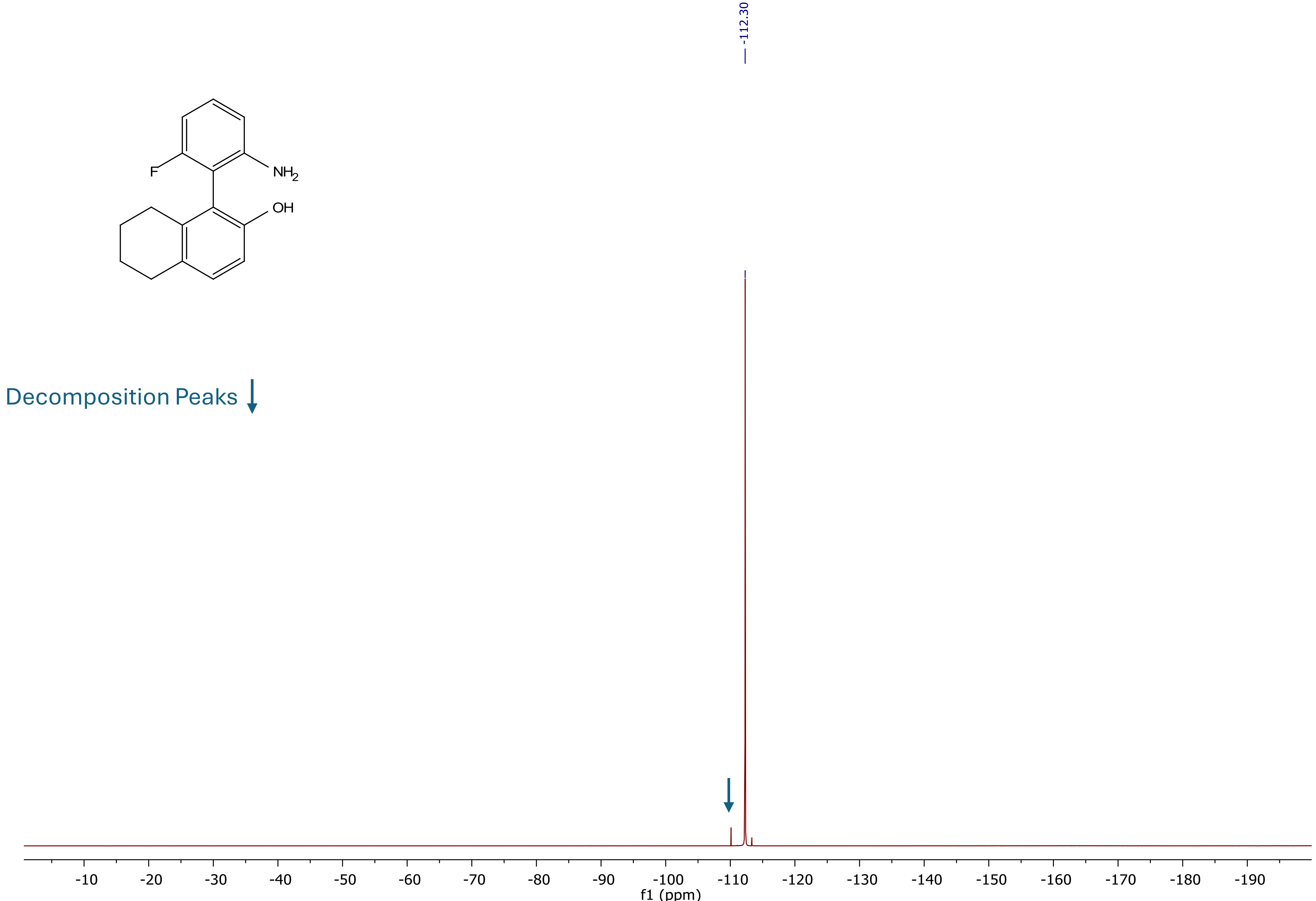

^{13}C-NMR (CDCl$_3$): *(R)*-1-(2-amino-6-fluorophenyl)-5,6,7,8-tetrahydronaphthalen-2-ol (**3q**)

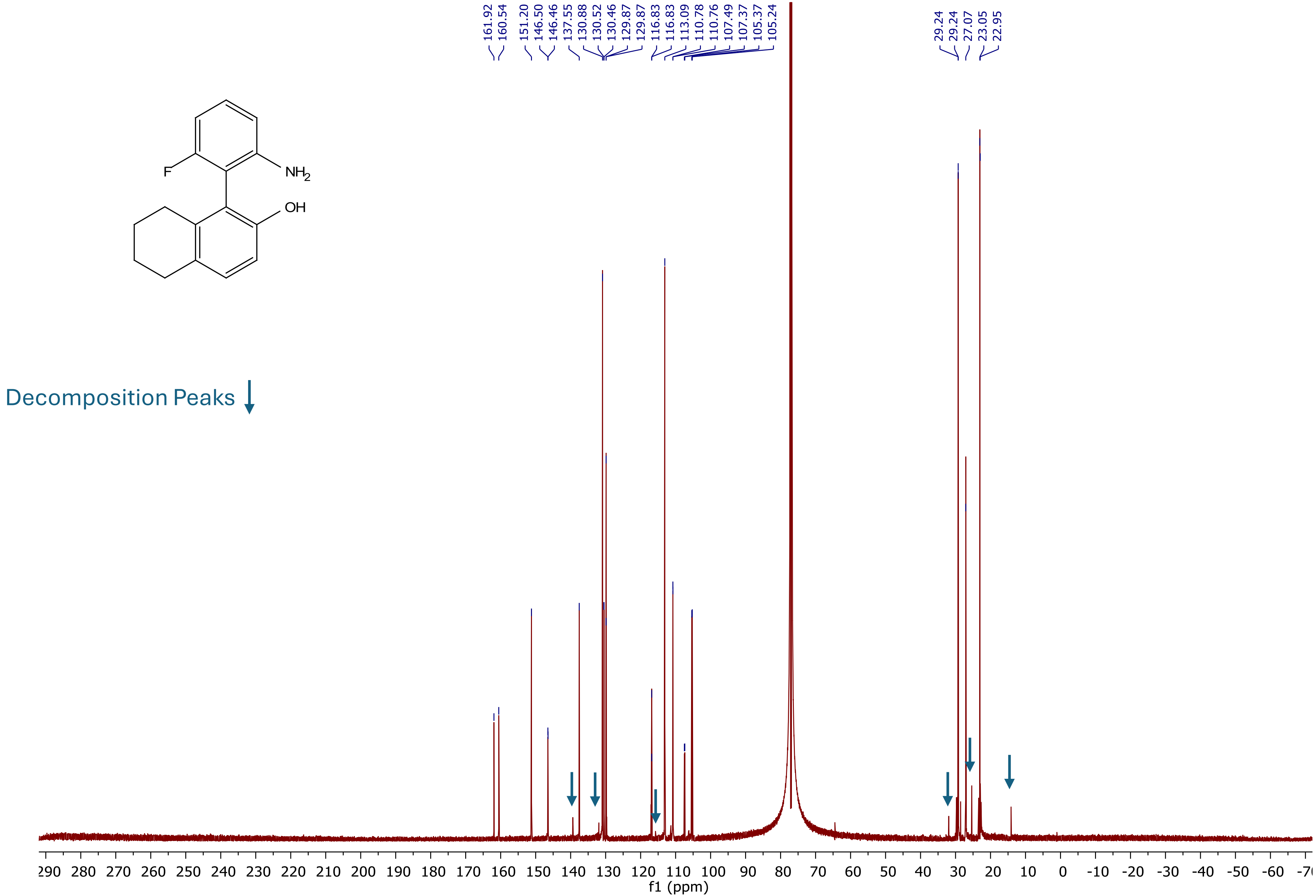

^{1}H-NMR ($CDCl_3$): *(S)*-2'-amino-3',5'-dichloro-6-methyl-[1,1'-biphenyl]-2-ol (**3r**)

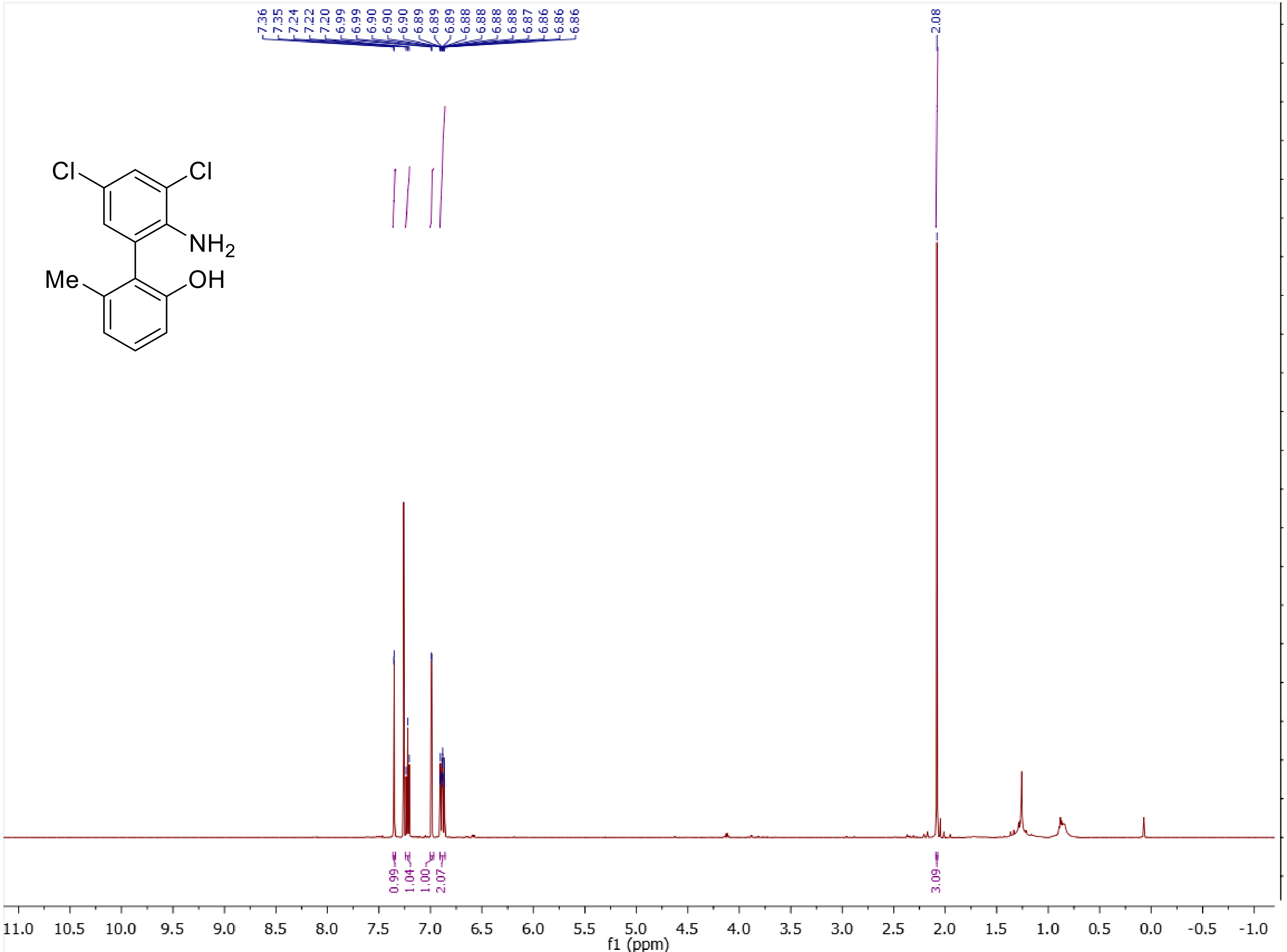

^{13}C-NMR (CDCl$_3$): *(S)*-2'-amino-3',5'-dichloro-6-methyl-[1,1'-biphenyl]-2-ol (**3r**)

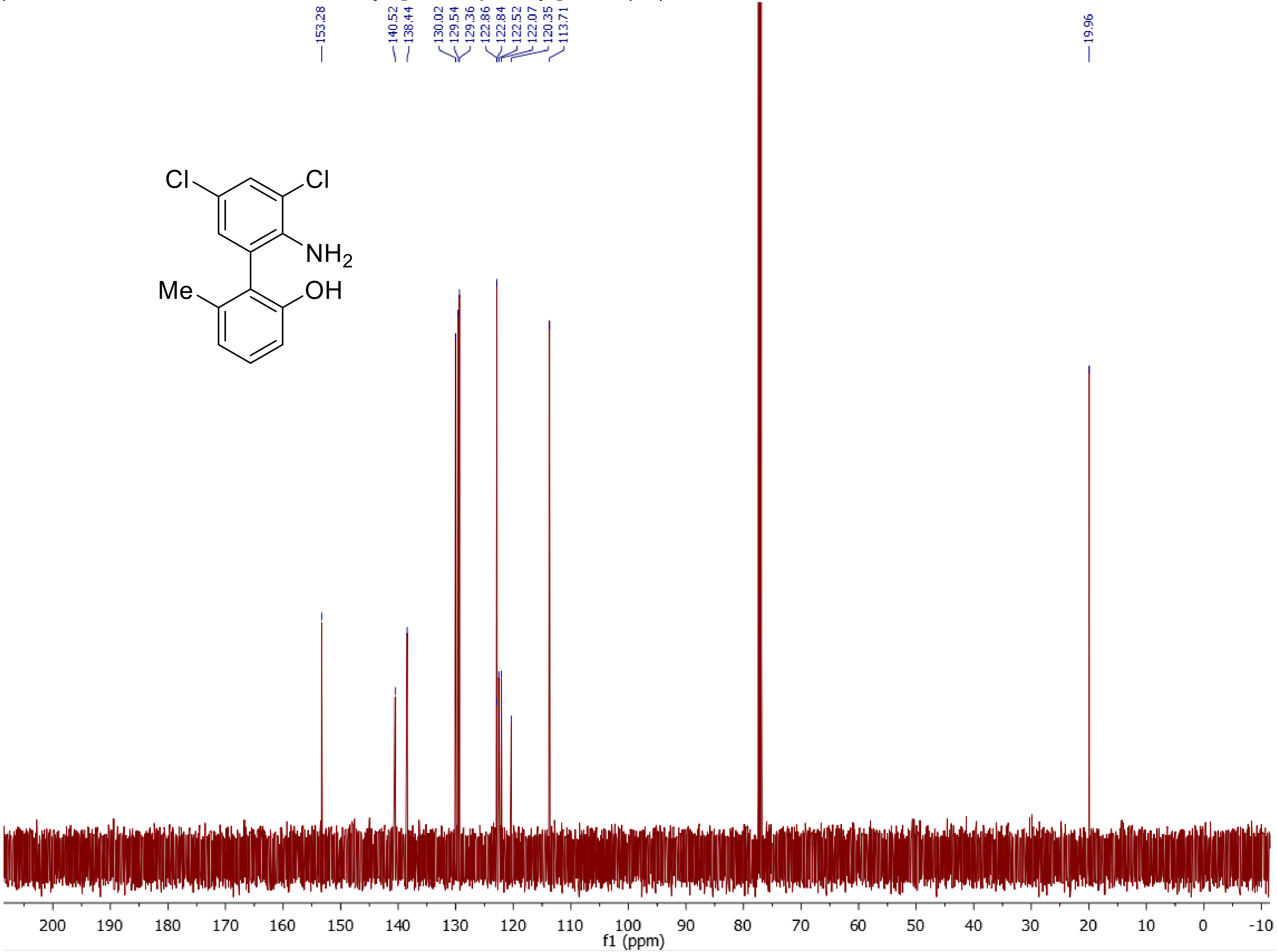

¹H-NMR (MeOD): *(S)*-6-chloro-2'-fluoro-6'-(methylamino)-[1,1'-biphenyl]-2-ol (**3s**)

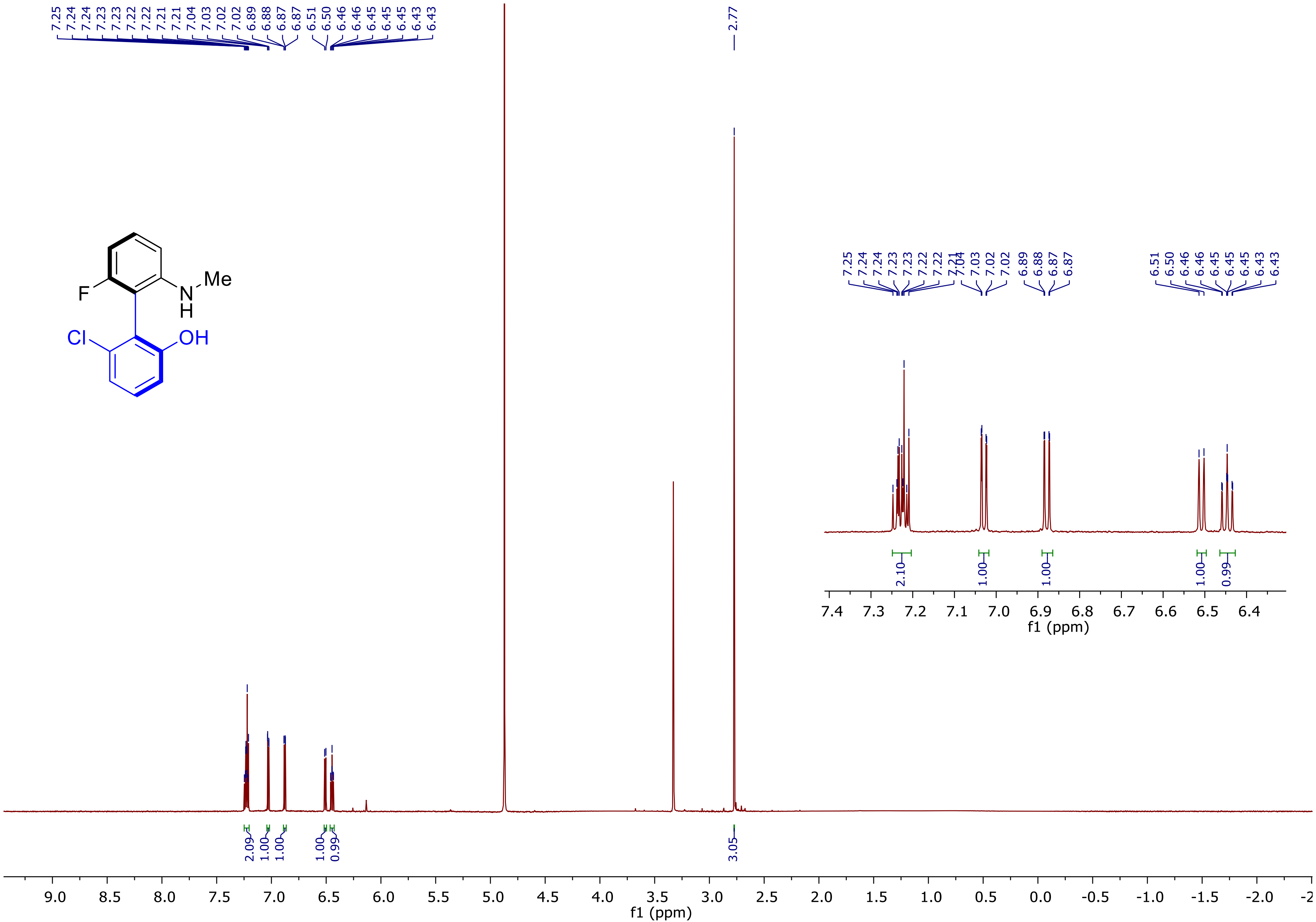

^{19}F-NMR ($CDCl_3$): *(S)*-6-chloro-2'-fluoro-6'-(methylamino)-[1,1'-biphenyl]-2-ol (**3s**)

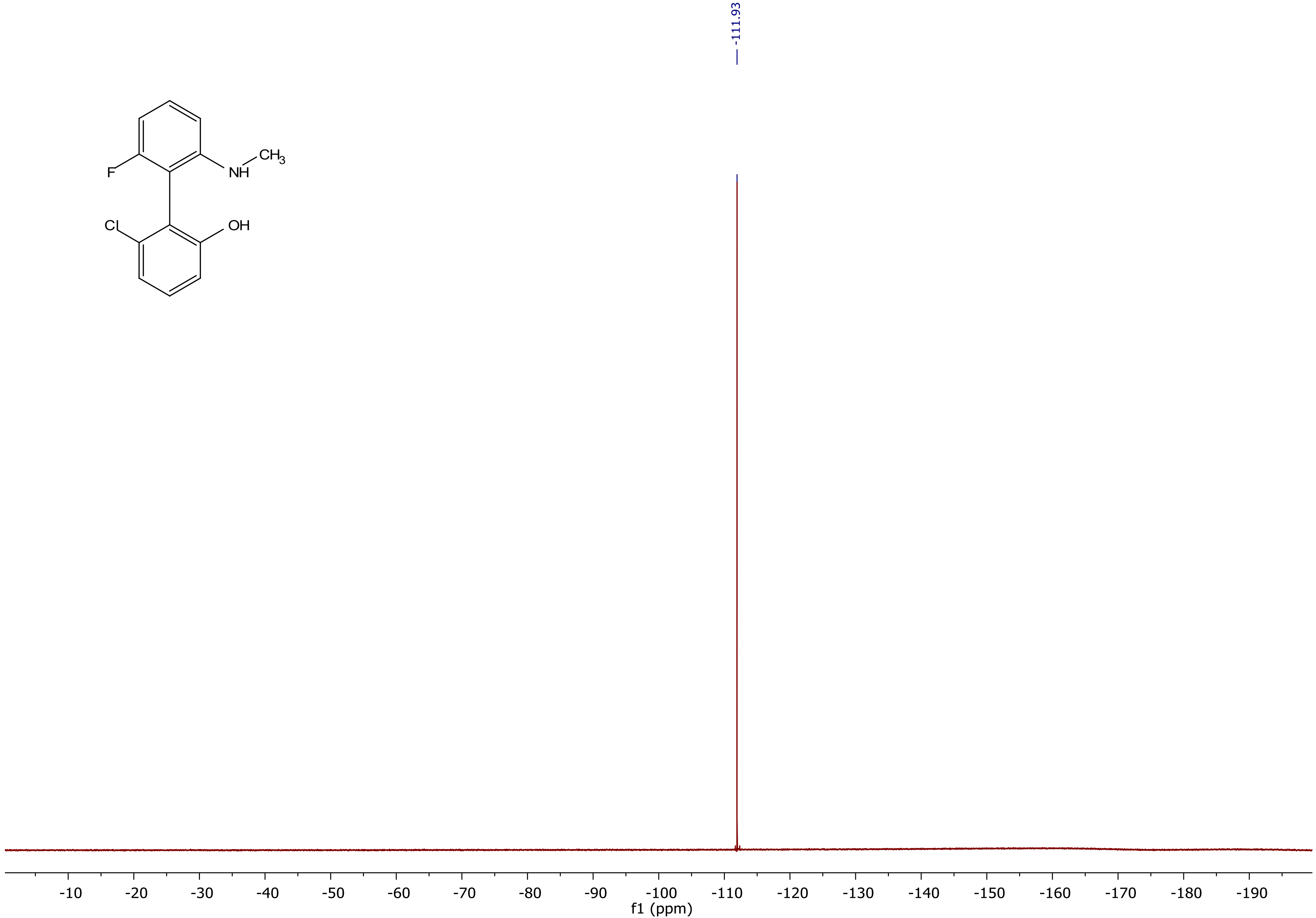

^{13}C-NMR (MeOD): *(S)*-6-chloro-2'-fluoro-6'-(methylamino)-[1,1'-biphenyl]-2-ol (**3s**)

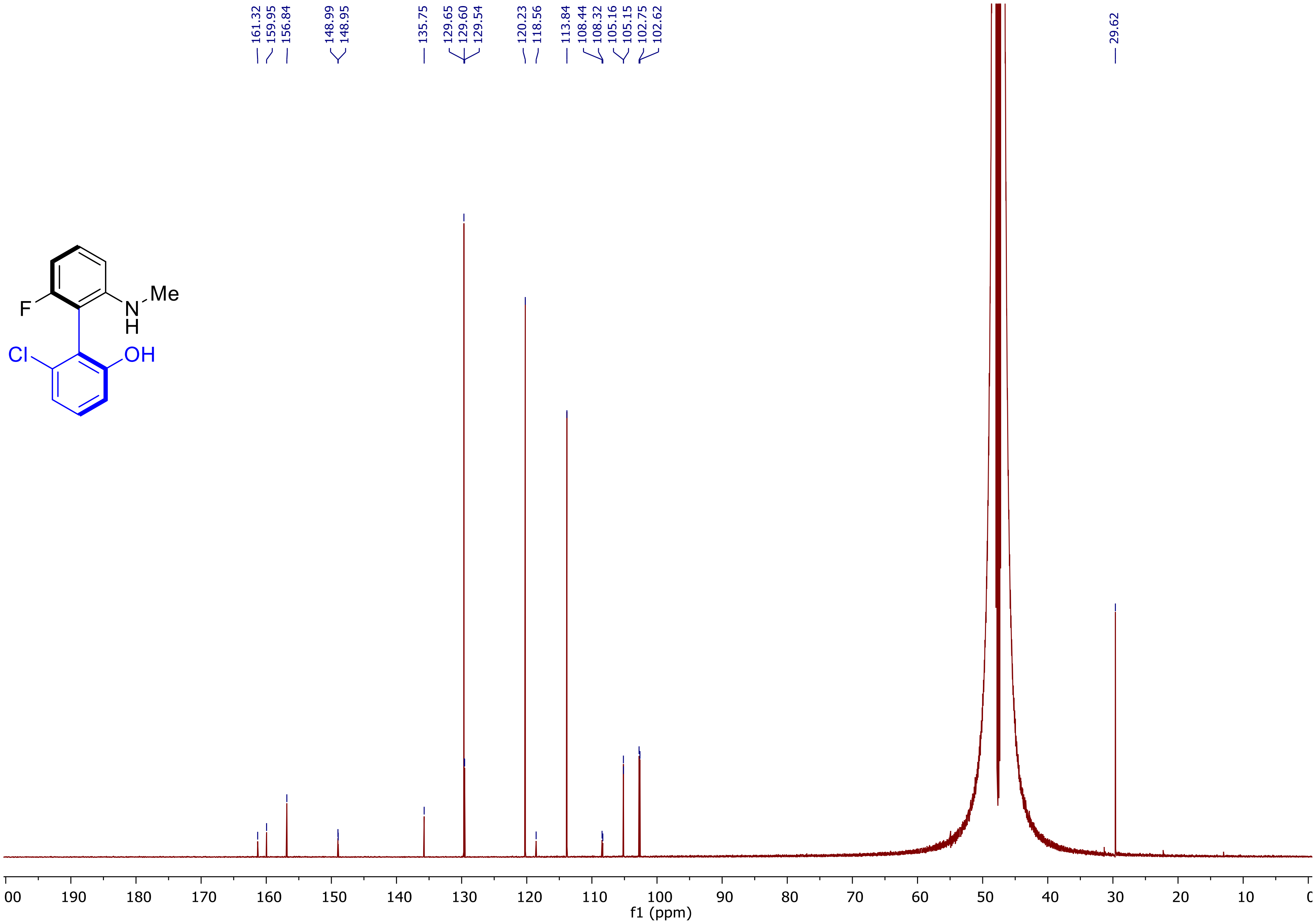

^{1}H-NMR ($CDCl_3$): (R)-6-fluoro-2'-methyl-6'-(methylamino)-[1,1'-biphenyl]-2-ol (**3t**)

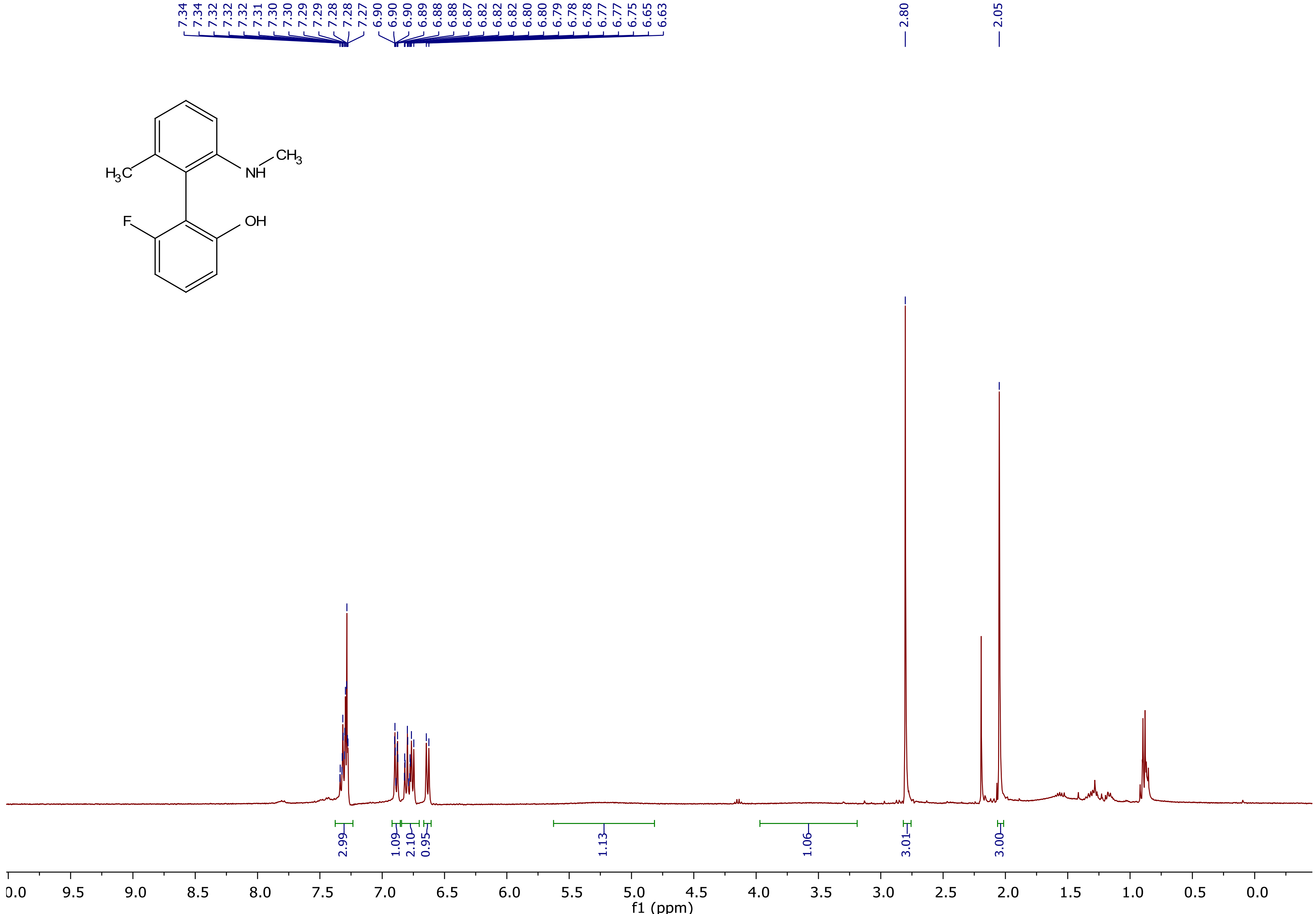

^{19}F-NMR (CDCl$_3$): *(R)-6-fluoro-2'-methyl-6'-(methylamino)-[1,1'-biphenyl]-2-ol* (**3t**)

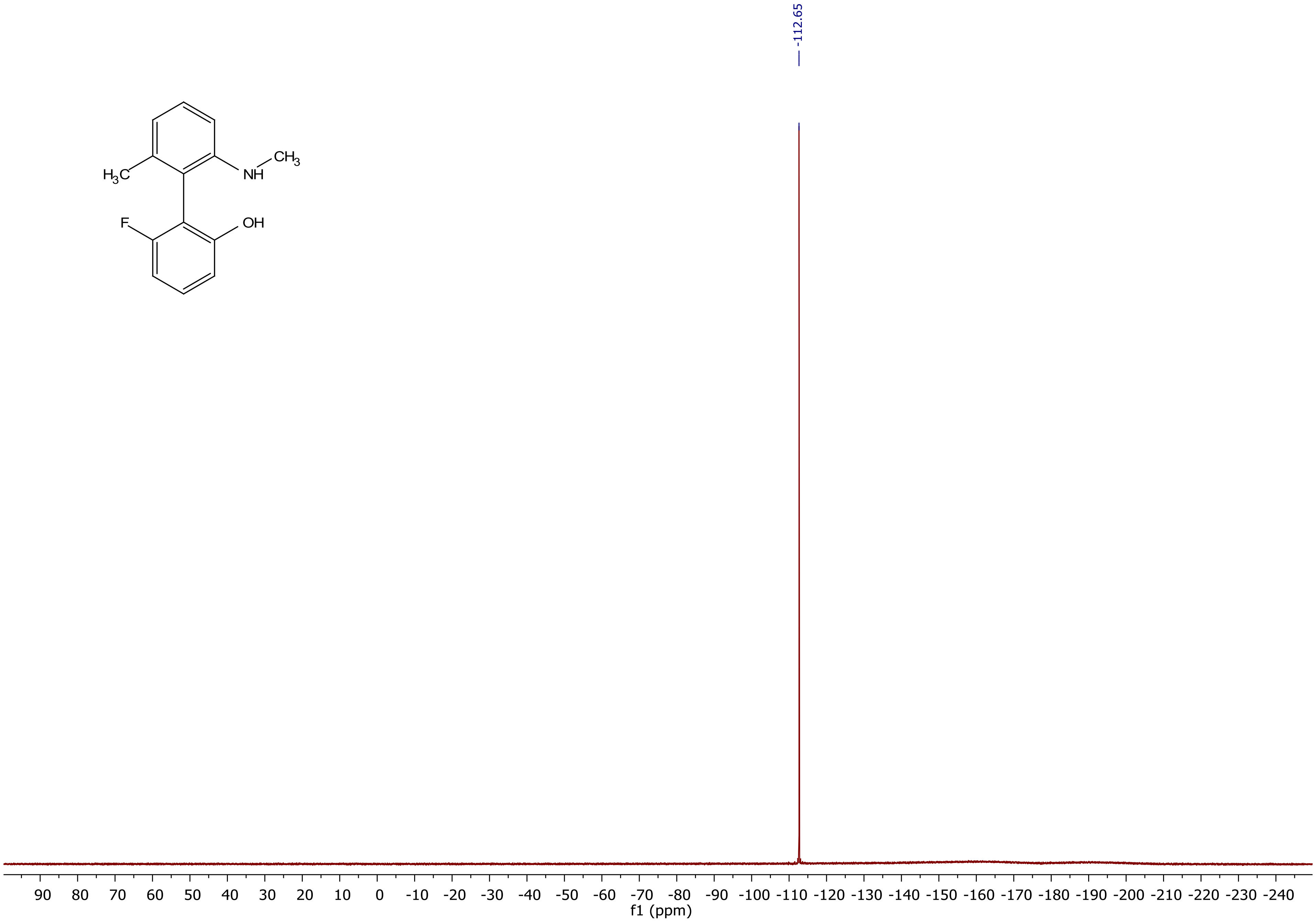

^{13}C-NMR (CDCl$_3$): *(R)-6-fluoro-2'-methyl-6'-(methylamino)-[1,1'-biphenyl]-2-ol* (**3t**)

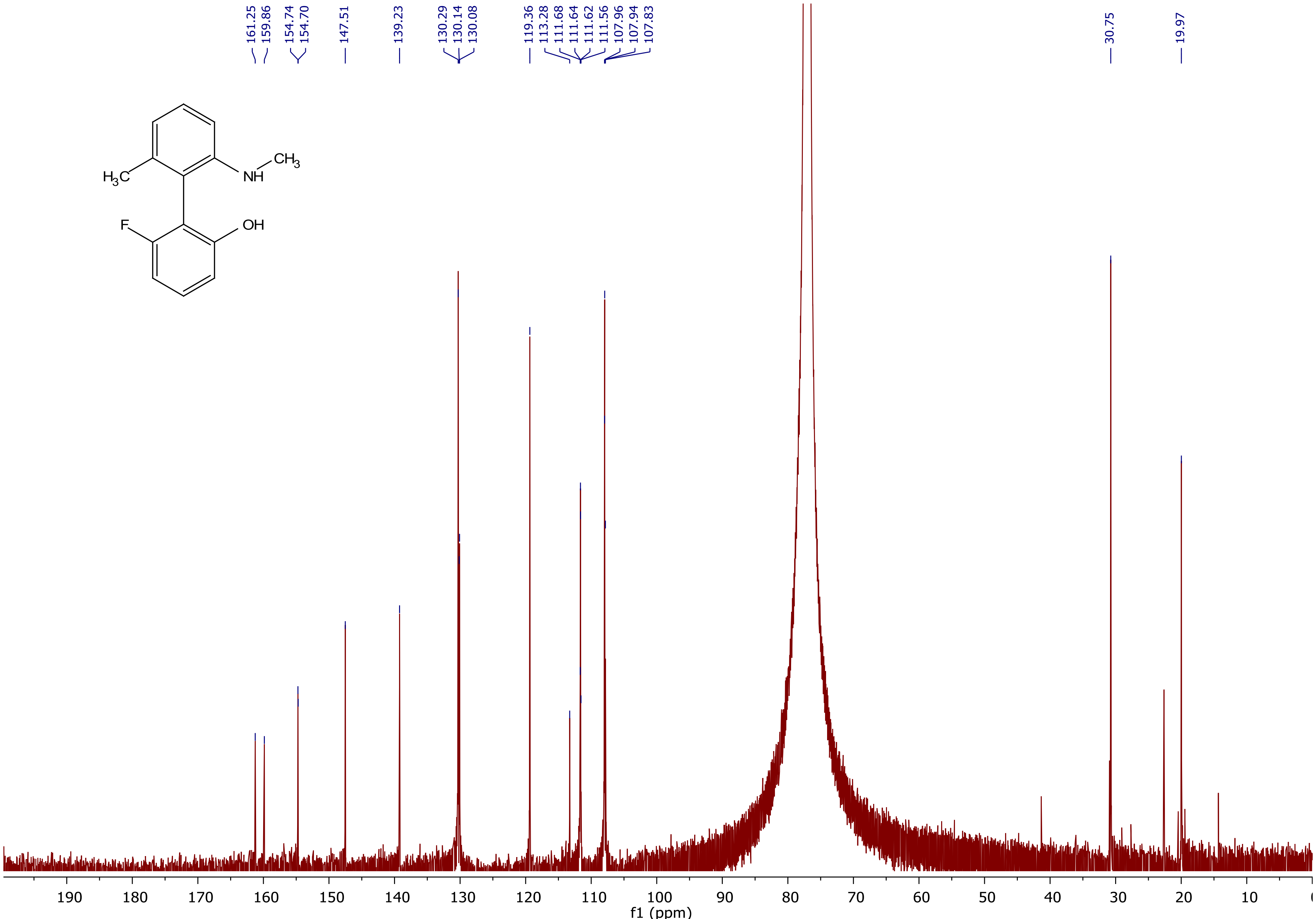

^{1}H-NMR ($CDCl_3$): *(R)*-6-chloro-2'-(ethylamino)-6'-fluoro-[1,1'-biphenyl]-2-ol (**3u**)

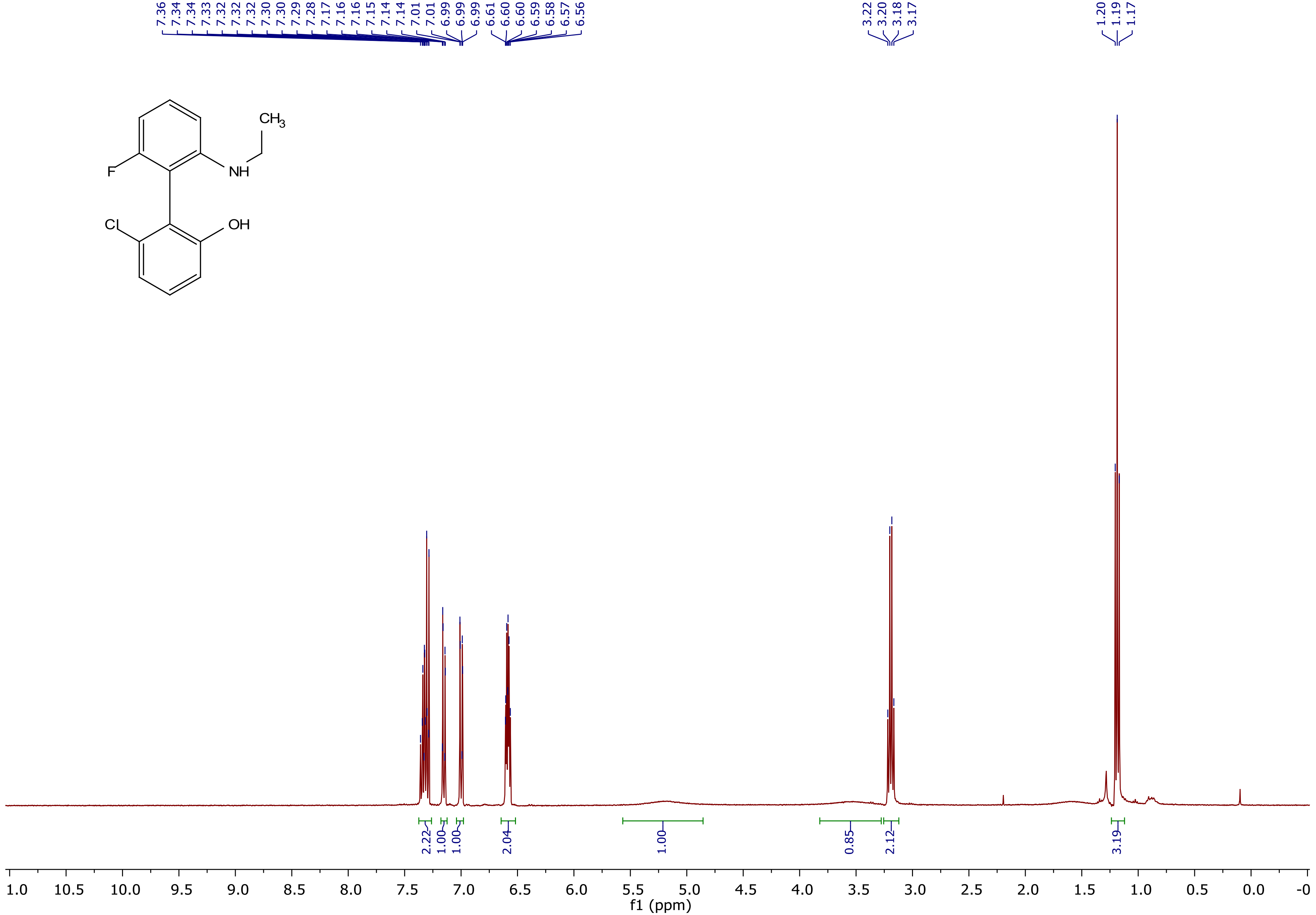

^{19}F-NMR (CDCl$_3$): *(S)*-6-chloro-2'-(ethylamino)-6'-fluoro-[1,1'-biphenyl]-2-ol (**3u**)

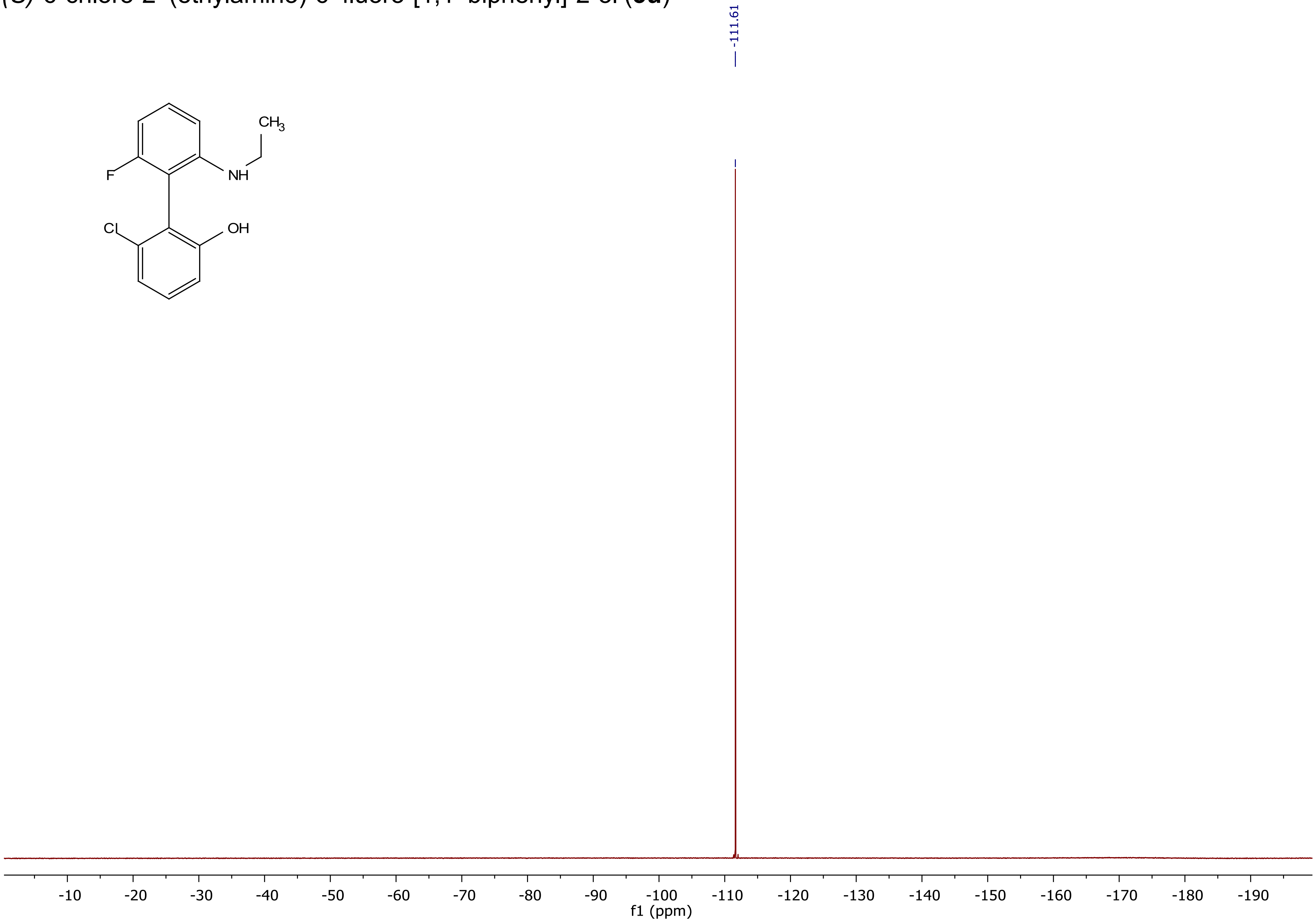

^{13}C-NMR ($CDCl_3$): *(S)*-6-chloro-2'-(ethylamino)-6'-fluoro-[1,1'-biphenyl]-2-ol (**3u**)

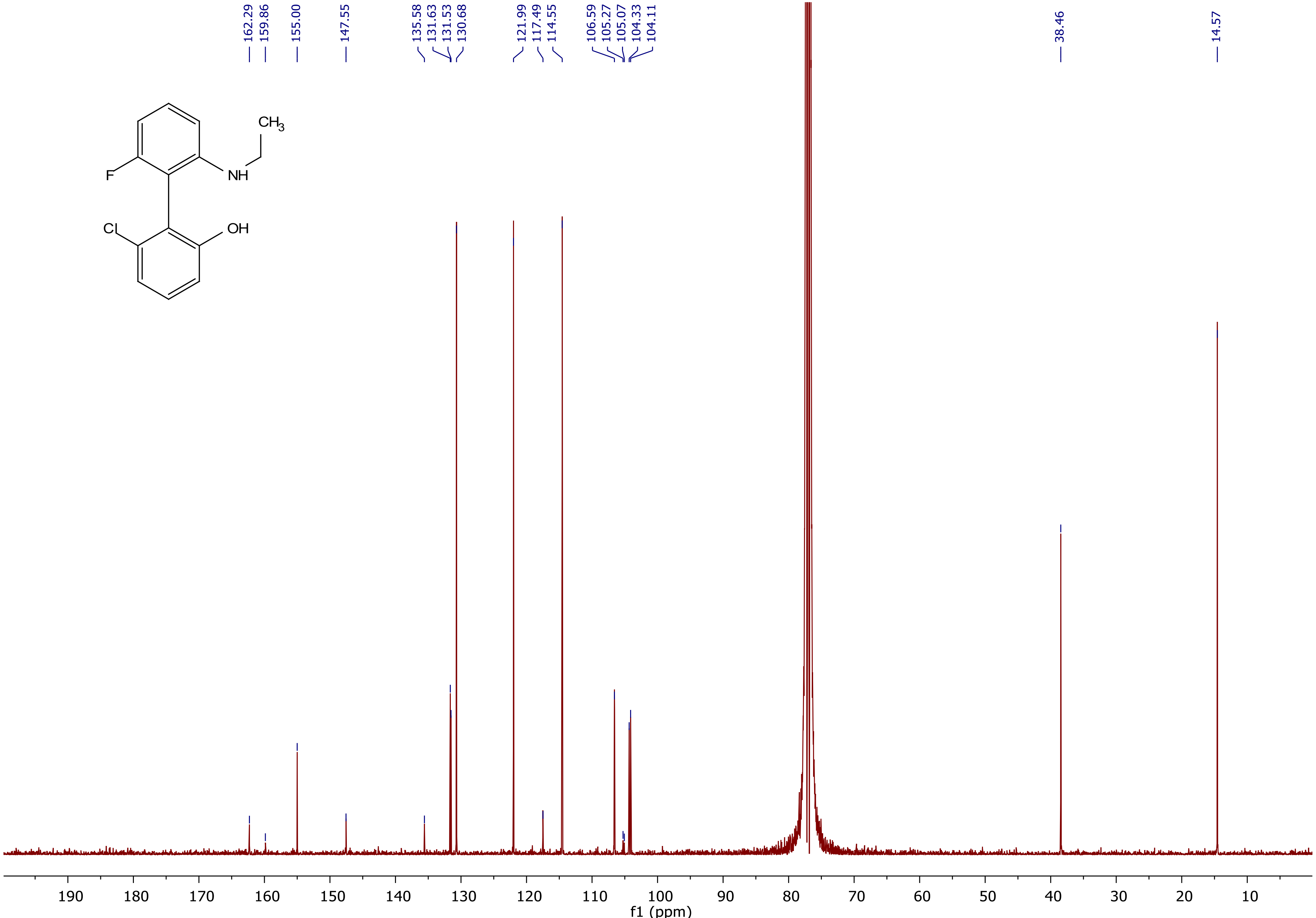

^{1}H-NMR ($CDCl_3$): (R)-2'-(ethylamino)-6-fluoro-6'-methyl-[1,1'-biphenyl]-2-ol (**3v**)

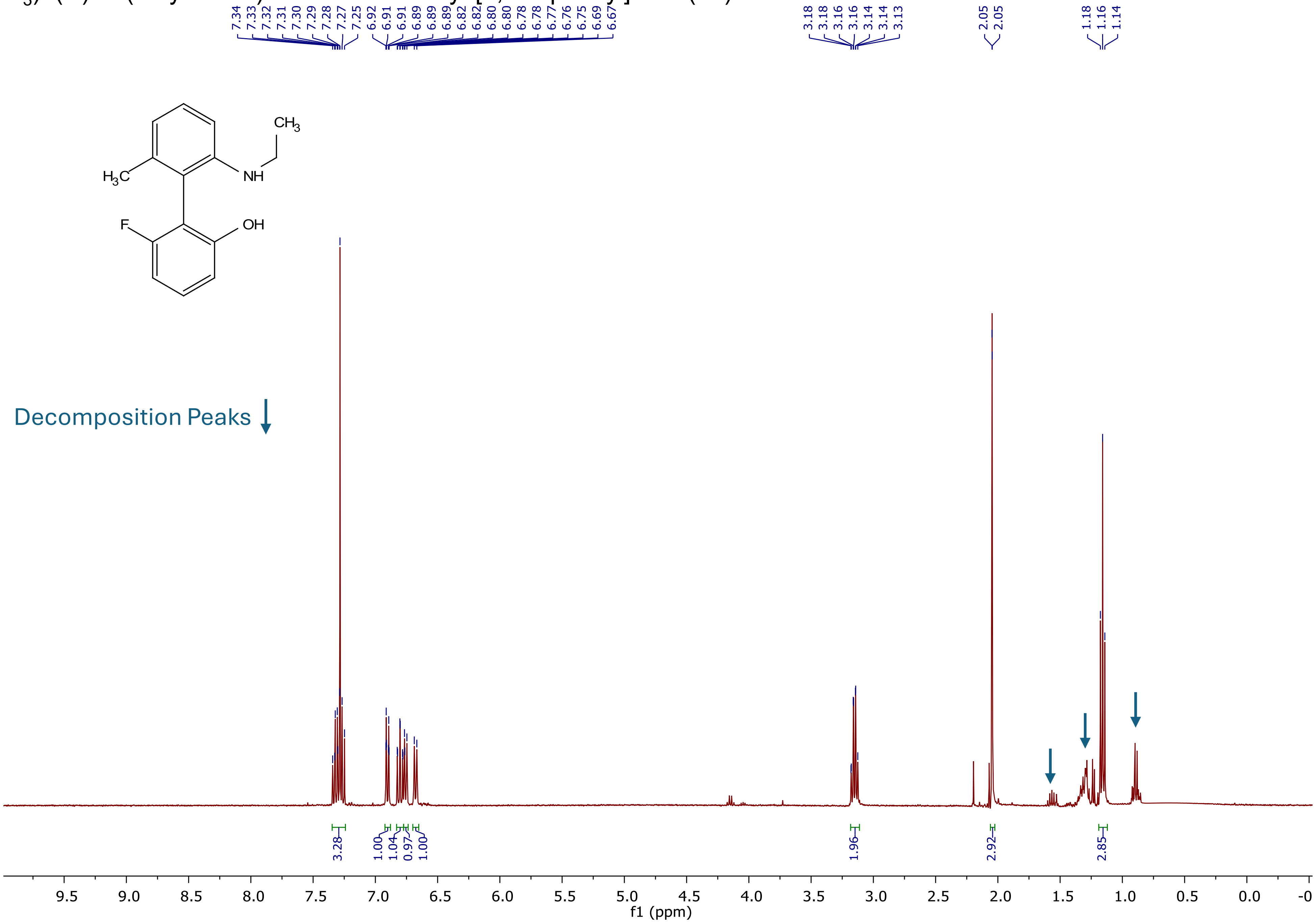

^{19}F-NMR (CDCl$_3$): (R)-2'-(ethylamino)-6-fluoro-6'-methyl-[1,1'-biphenyl]-2-ol (**3v**)

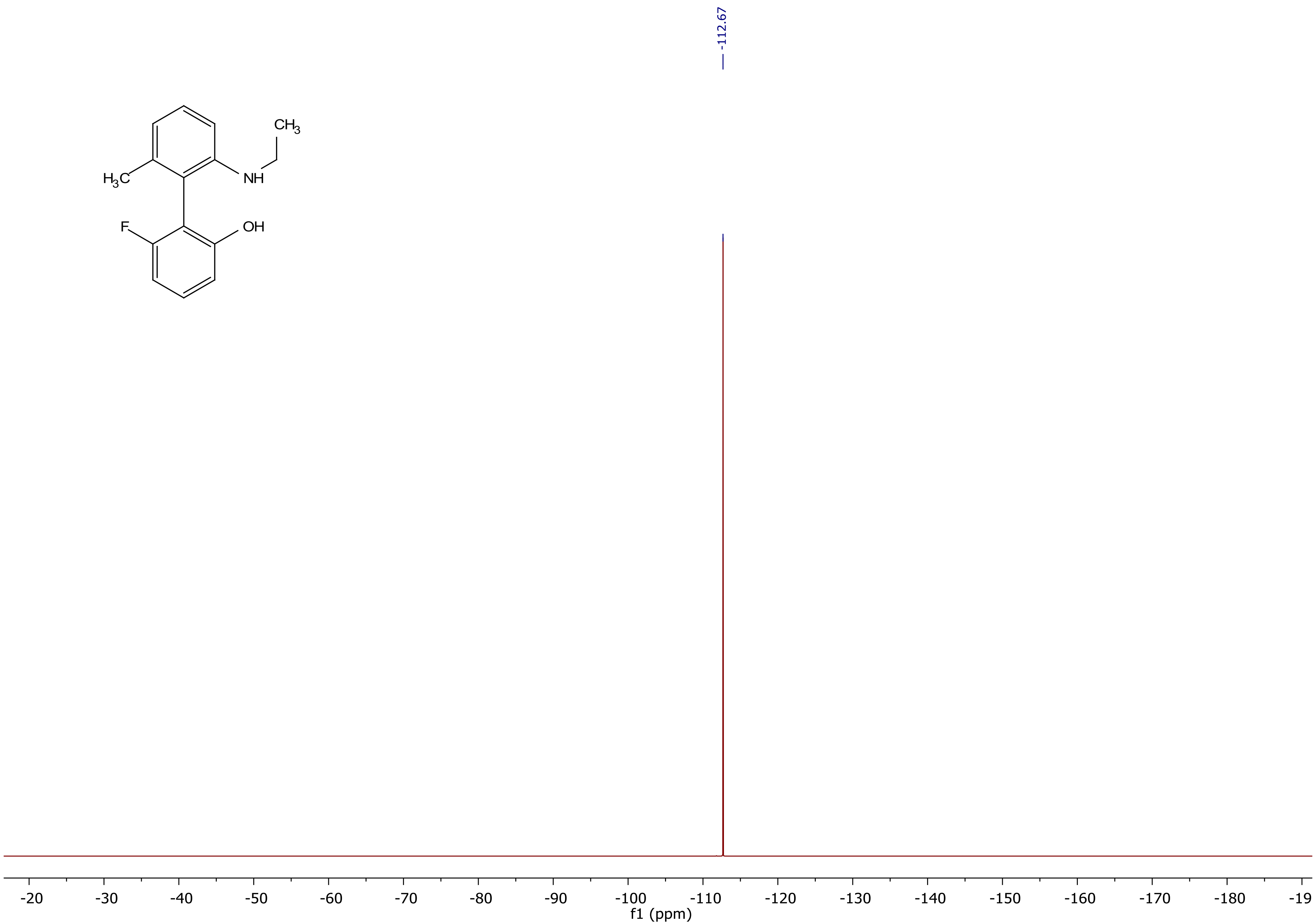

^{13}C-NMR ($CDCl_3$): *(R)-2'-(ethylamino)-6-fluoro-6'-methyl-[1,1'-biphenyl]-2-ol* (**3v**)

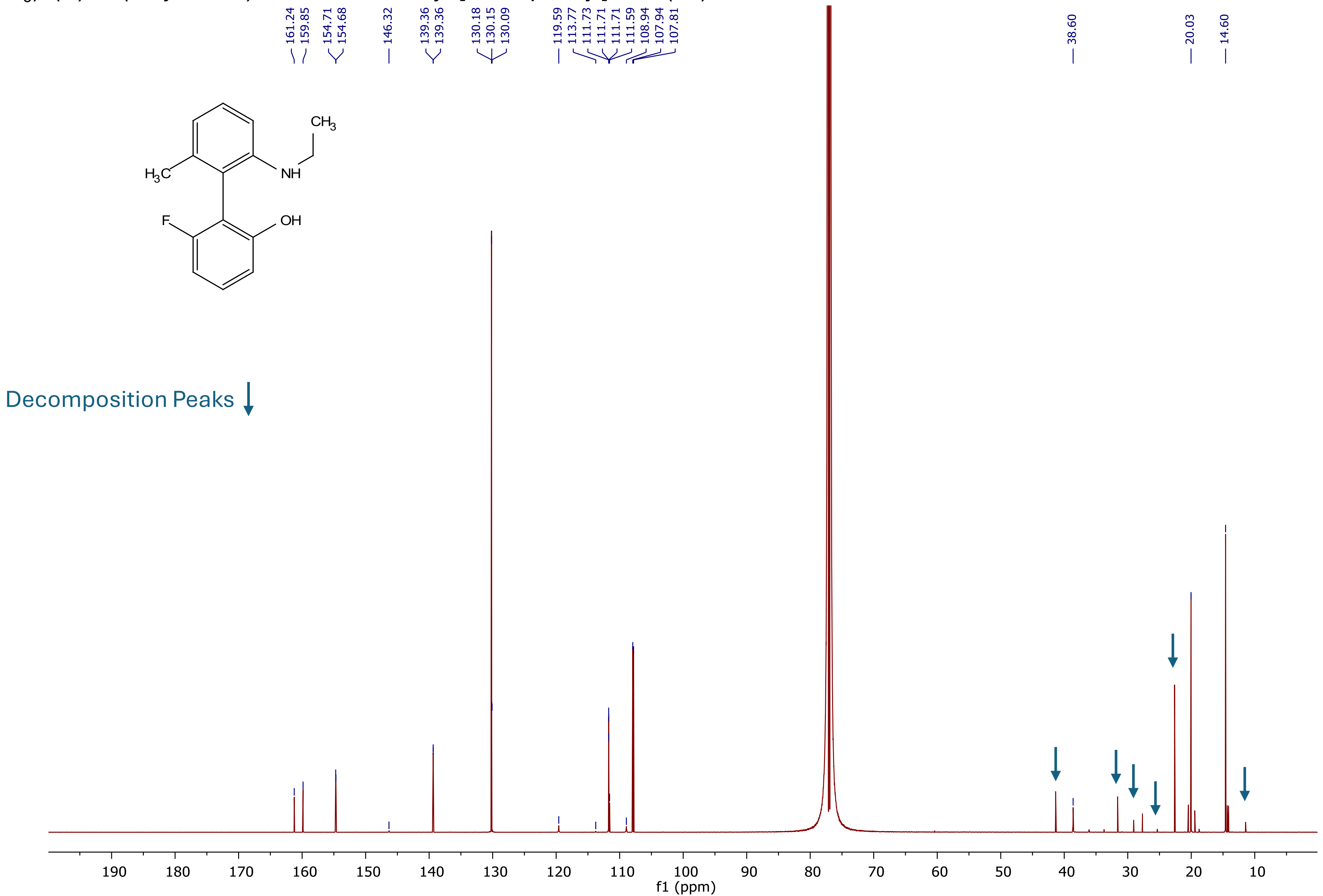

^{1}H-NMR ($CDCl_3$): *(S)*-2'-(benzylamino)-6-chloro-6'-fluoro-[1,1'-biphenyl]-2-ol (**3w**)

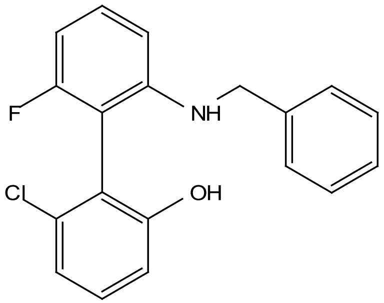

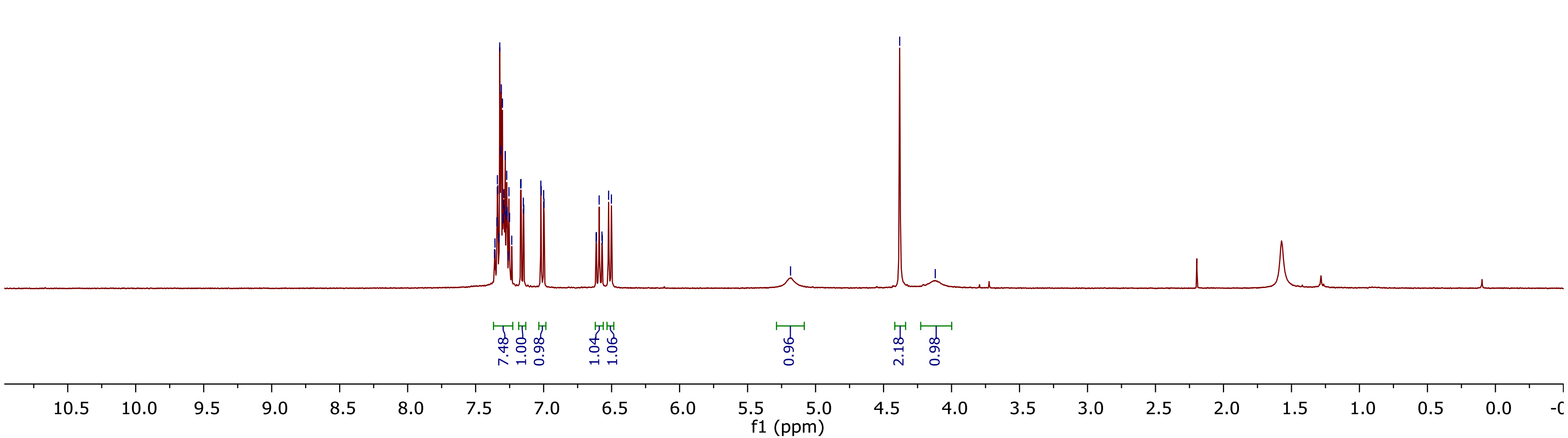

^{19}F-NMR ($CDCl_3$): *(S)*-2'-(benzylamino)-6-chloro-6'-fluoro-[1,1'-biphenyl]-2-ol (**3w**)

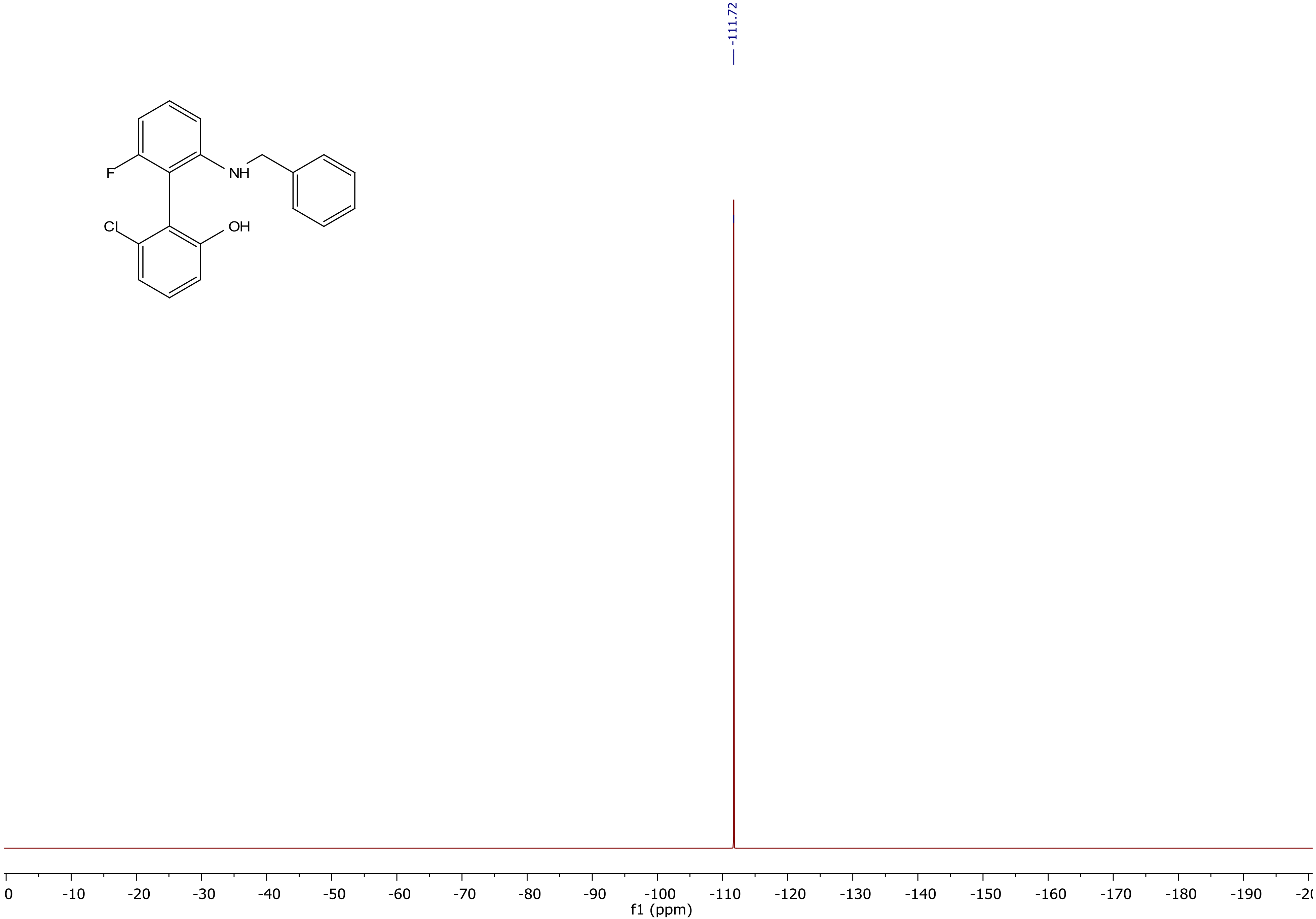

^{13}C-NMR ($CDCl_3$): *(S)*-2'-(benzylamino)-6-chloro-6'-fluoro-[1,1'-biphenyl]-2-ol (**3w**)

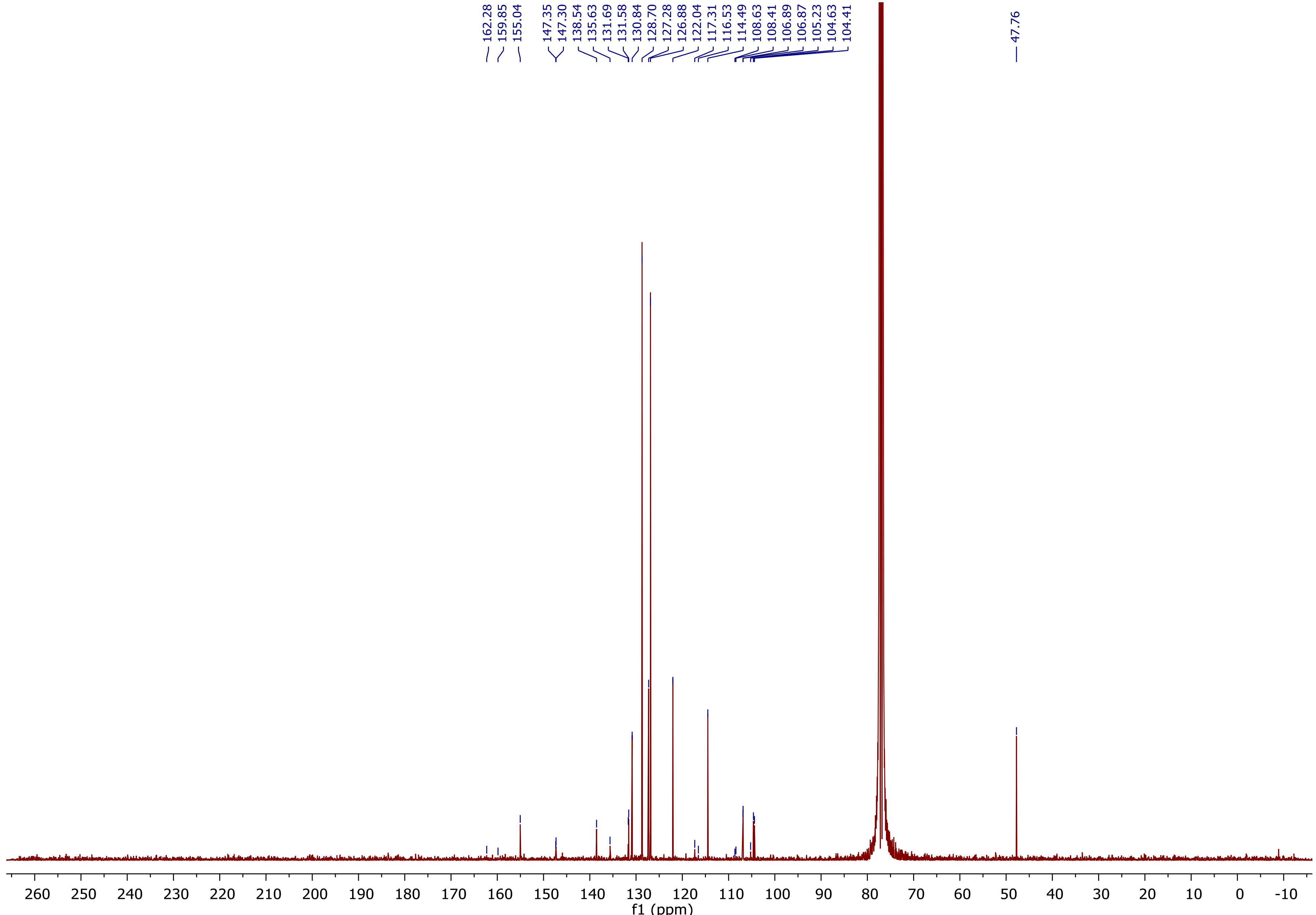

^{1}H-NMR ($CDCl_3$): *(R)-6-fluoro-2'-methyl-6'-nitro-[1,1'-biphenyl]-2-ol* (***3y***)

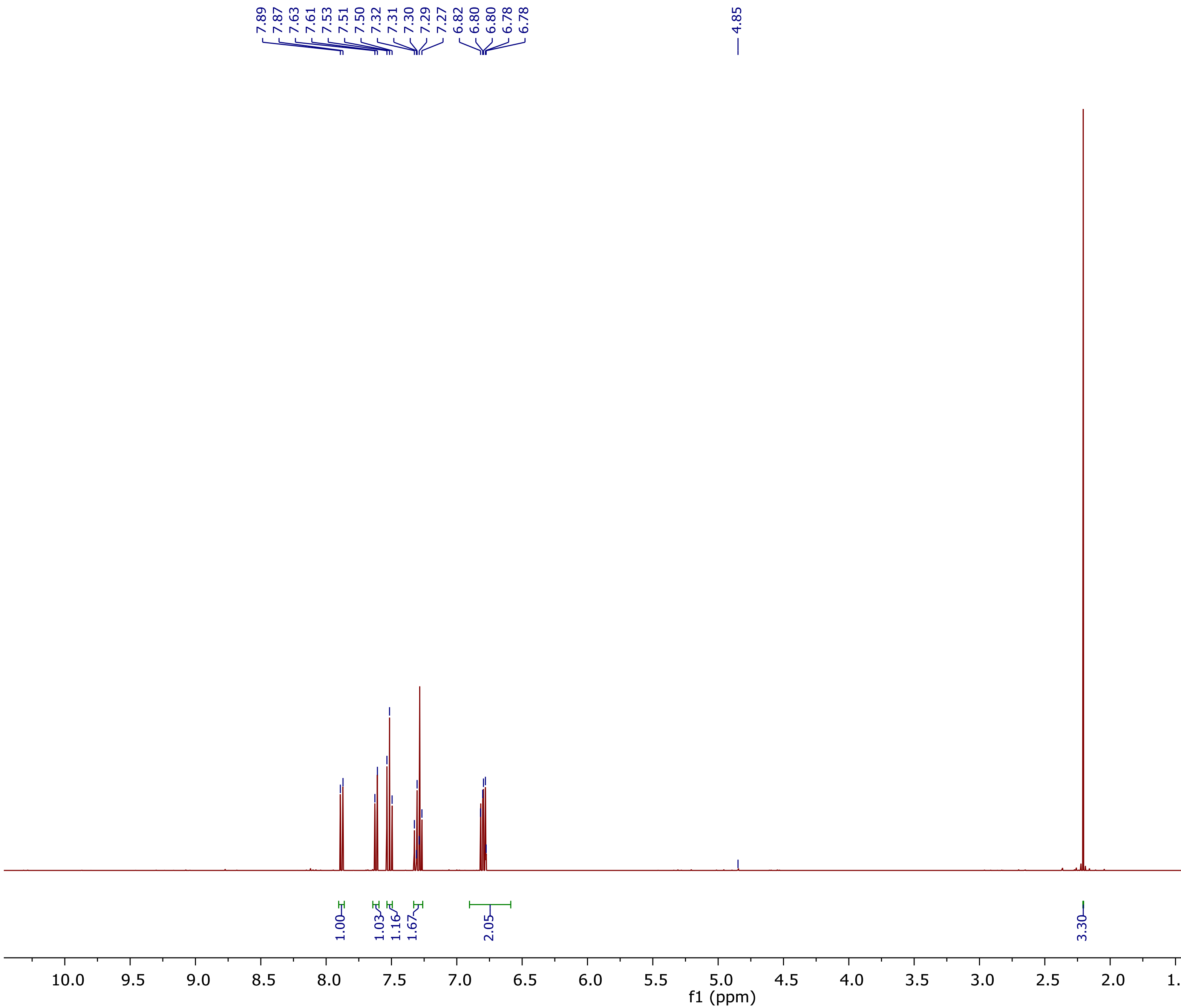

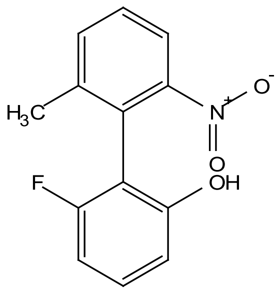

^{19}F-NMR (CDCl$_3$): *(R)-6-fluoro-2'-methyl-6'-nitro-[1,1'-biphenyl]-2-ol* (***3y***)

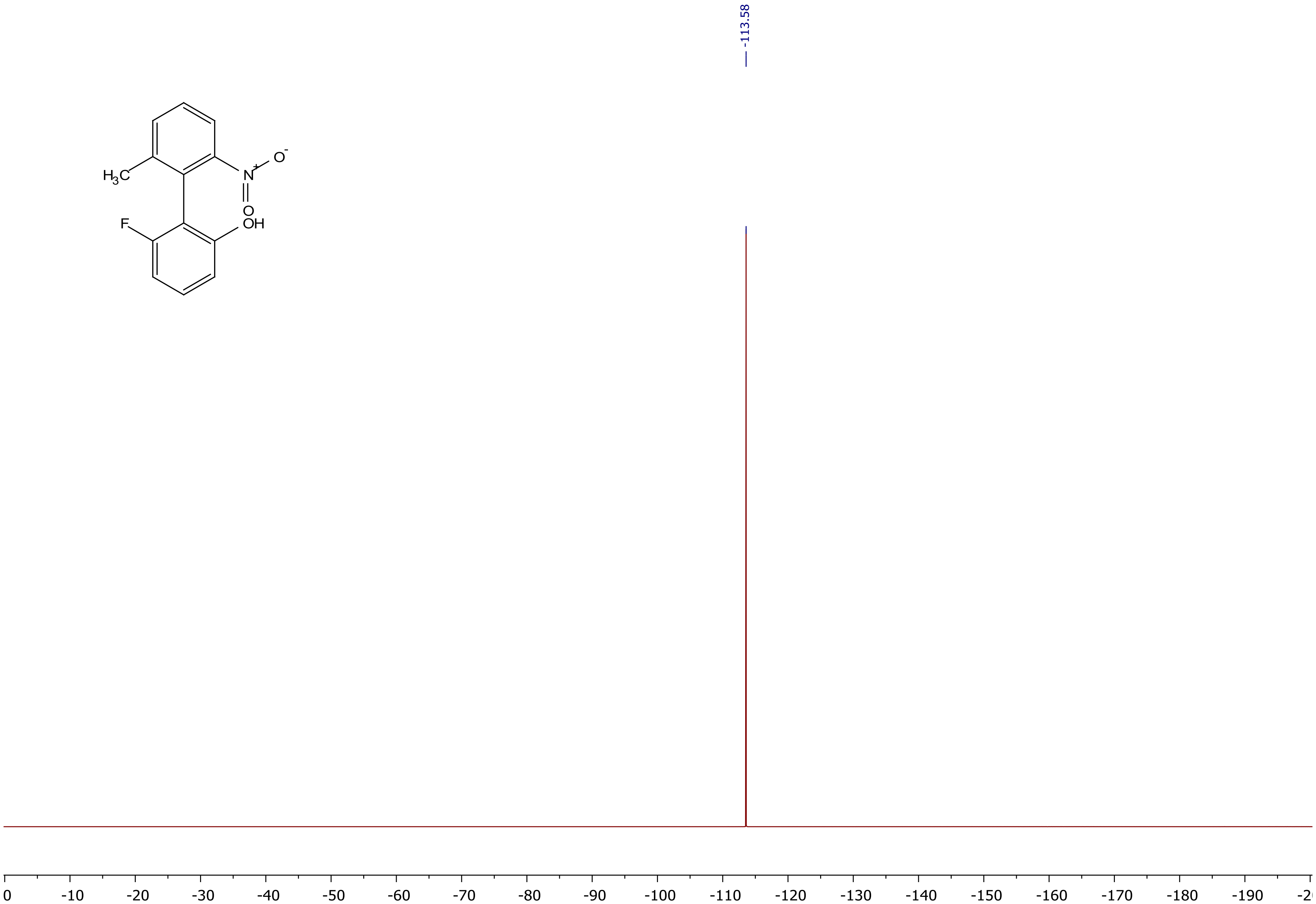

^{13}C-NMR ($CDCl_3$): *(R)-6-fluoro-2'-methyl-6'-nitro-[1,1'-biphenyl]-2-ol* (***3y***)

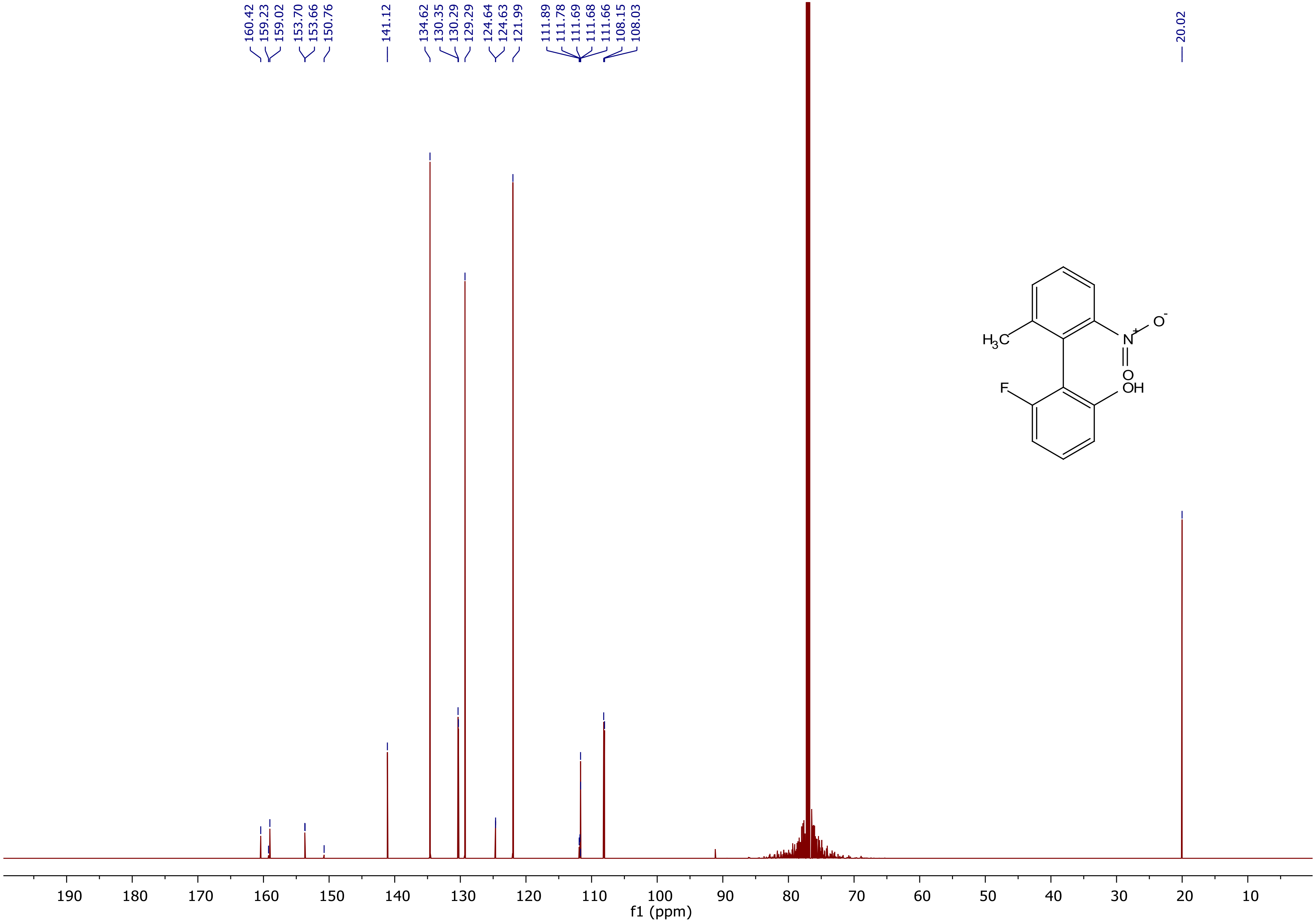

¹H-NMR ($CDCl_3$): *(R)-2-(2-aminonaphthalen-1-yl)-3-fluorophenol* (***3z***)

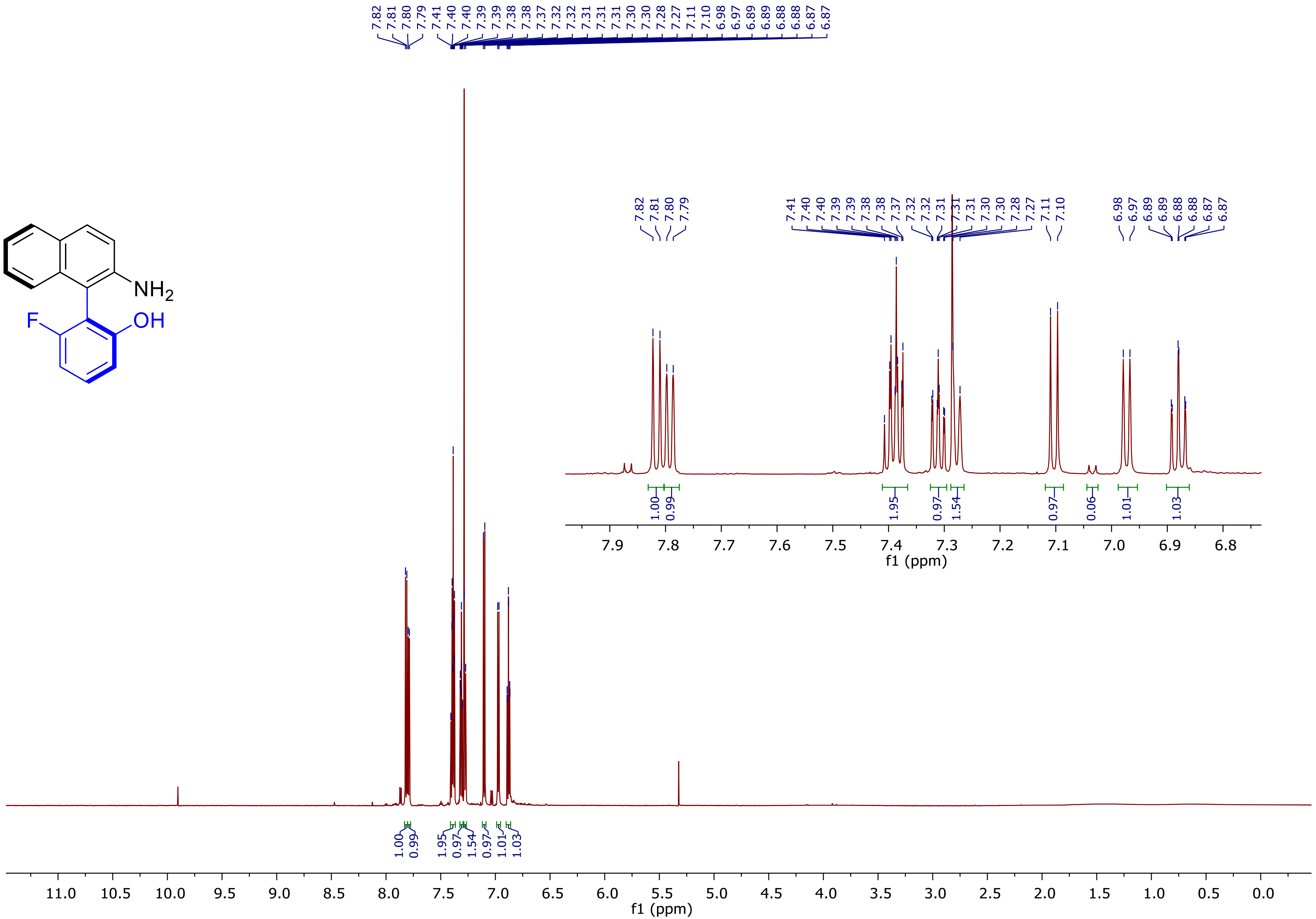

^{19}F-NMR ($CDCl_3$): *(R)-2-(2-aminonaphthalen-1-yl)-3-fluorophenol (**3z**)*

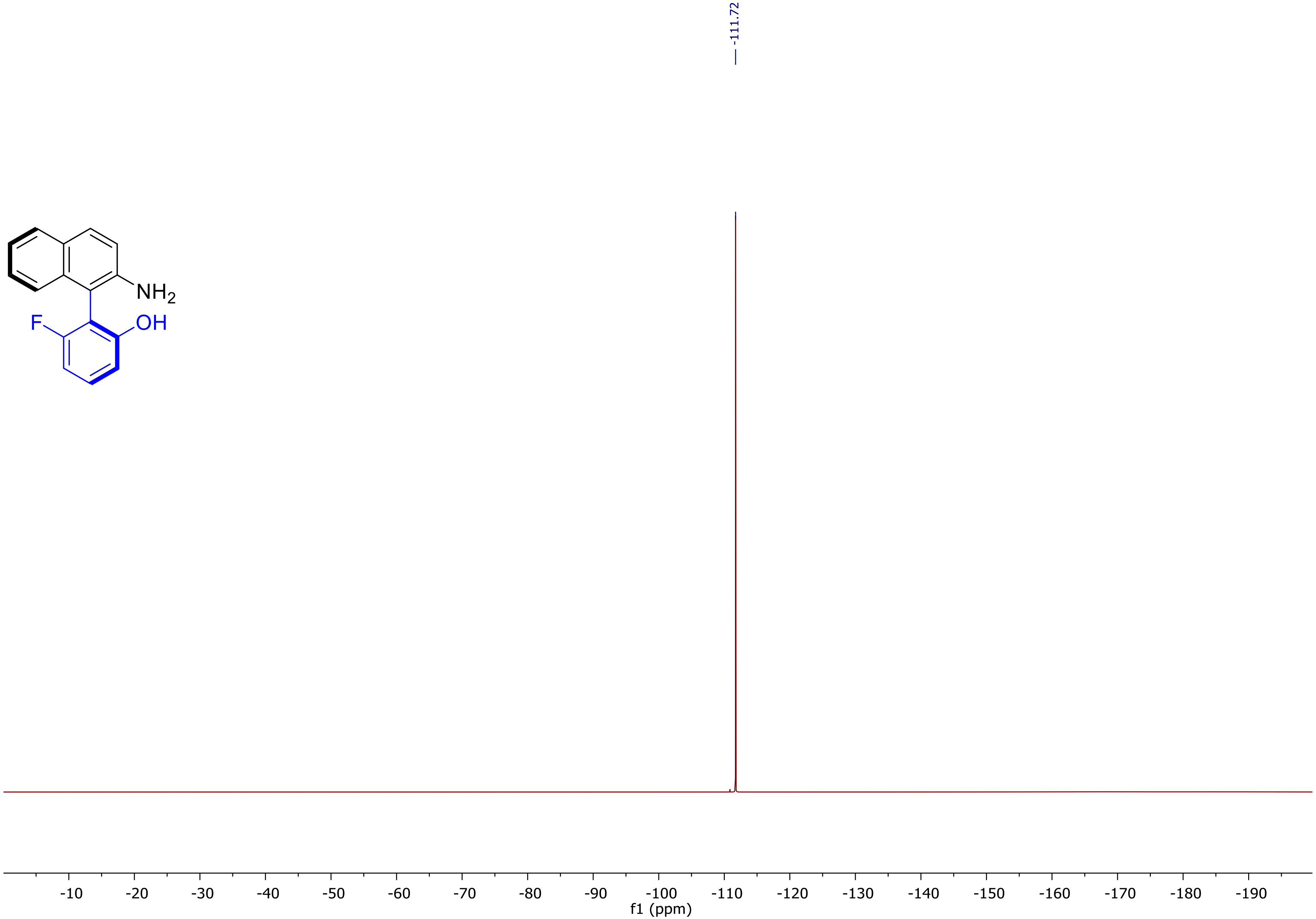

^{13}C-NMR ($CDCl_3$): *(R)-2-(2-aminonaphthalen-1-yl)-3-fluorophenol* (***3z***)

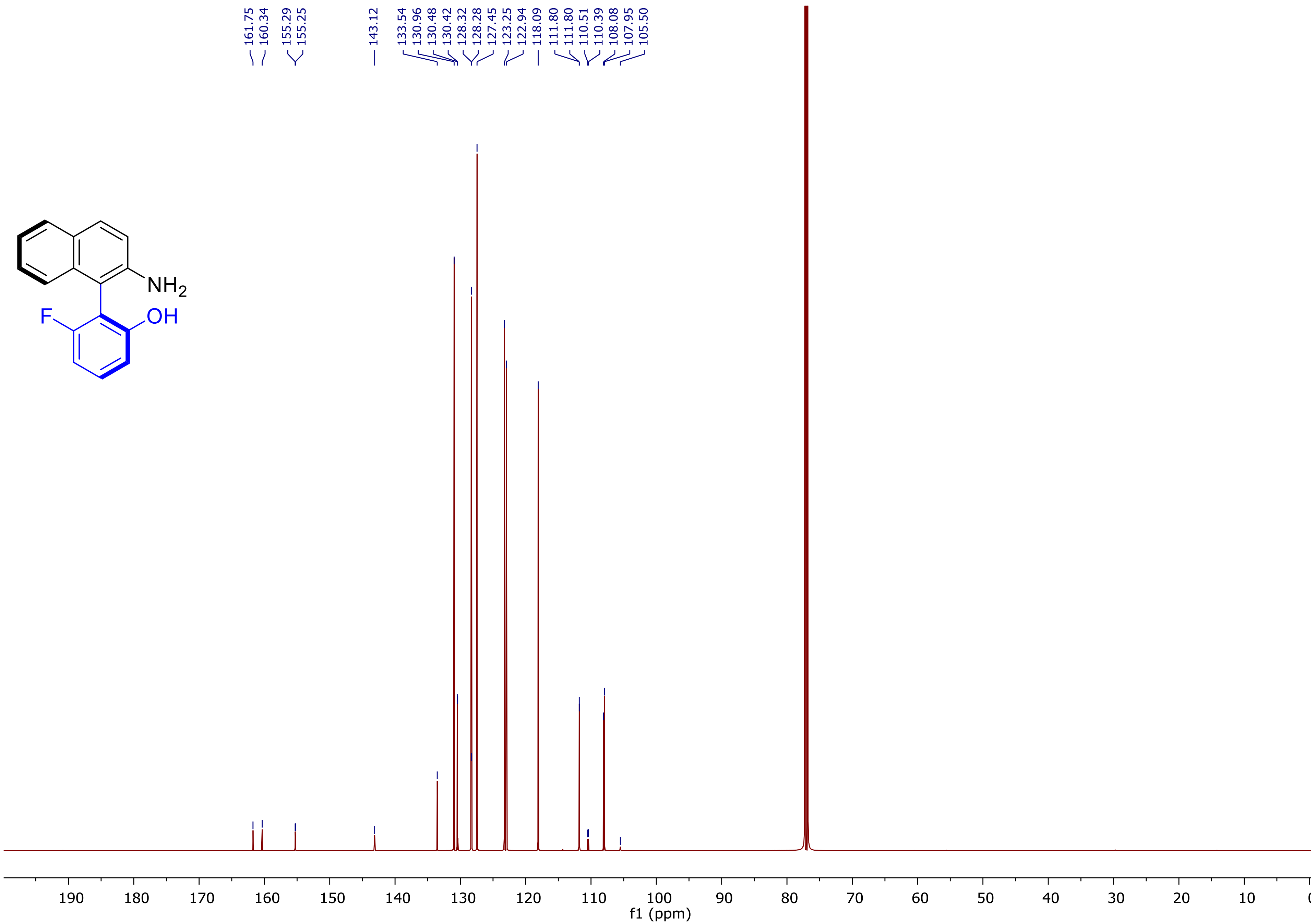

¹H-NMR ($CDCl_3$): *(R)-3-chloro-2-(6-methylindolin-7-yl)phenol (**3za**)*

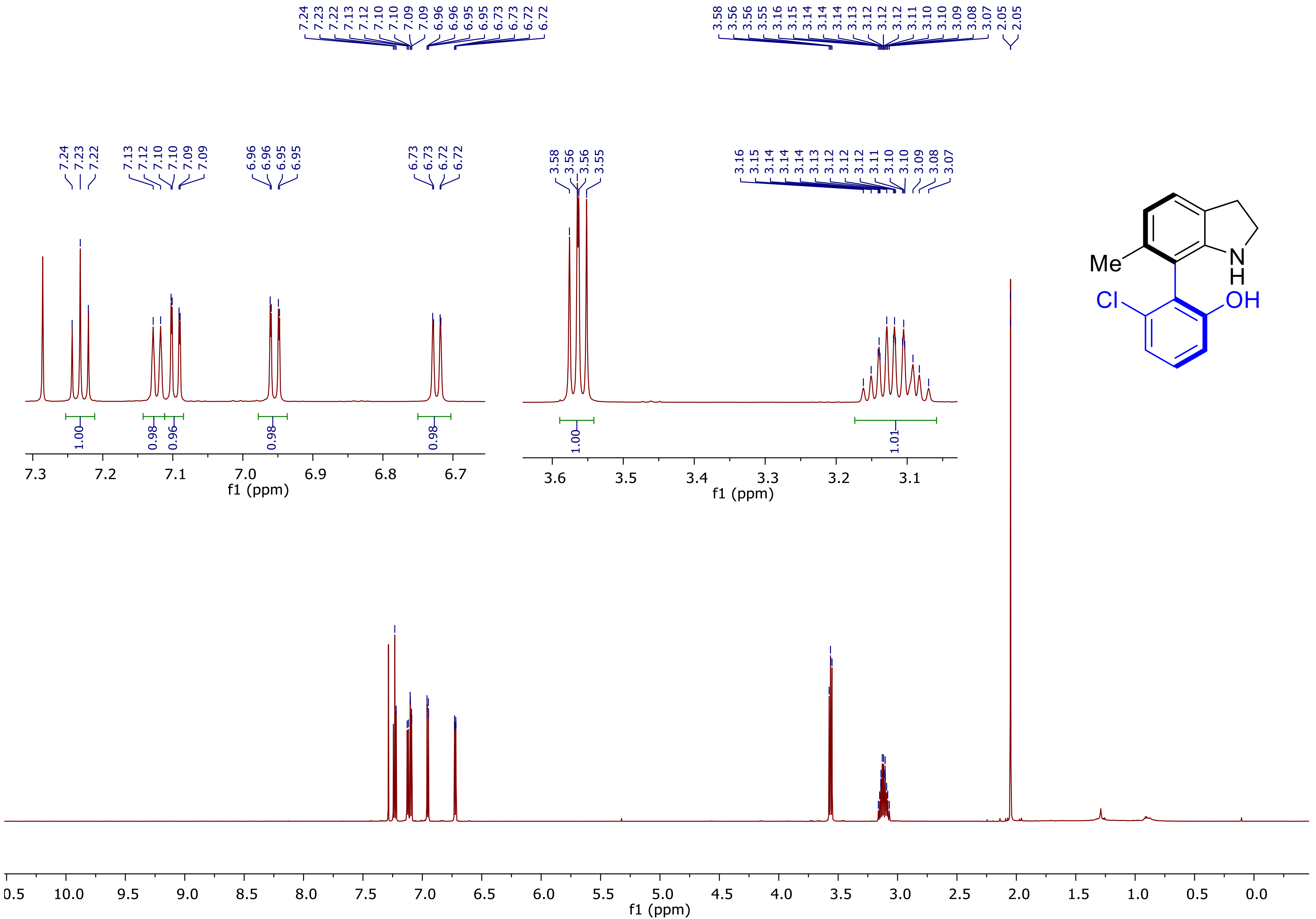

^{13}C-NMR ($CDCl_3$): *(R)-3-chloro-2-(6-methylindolin-7-yl)phenol* (***3za***)

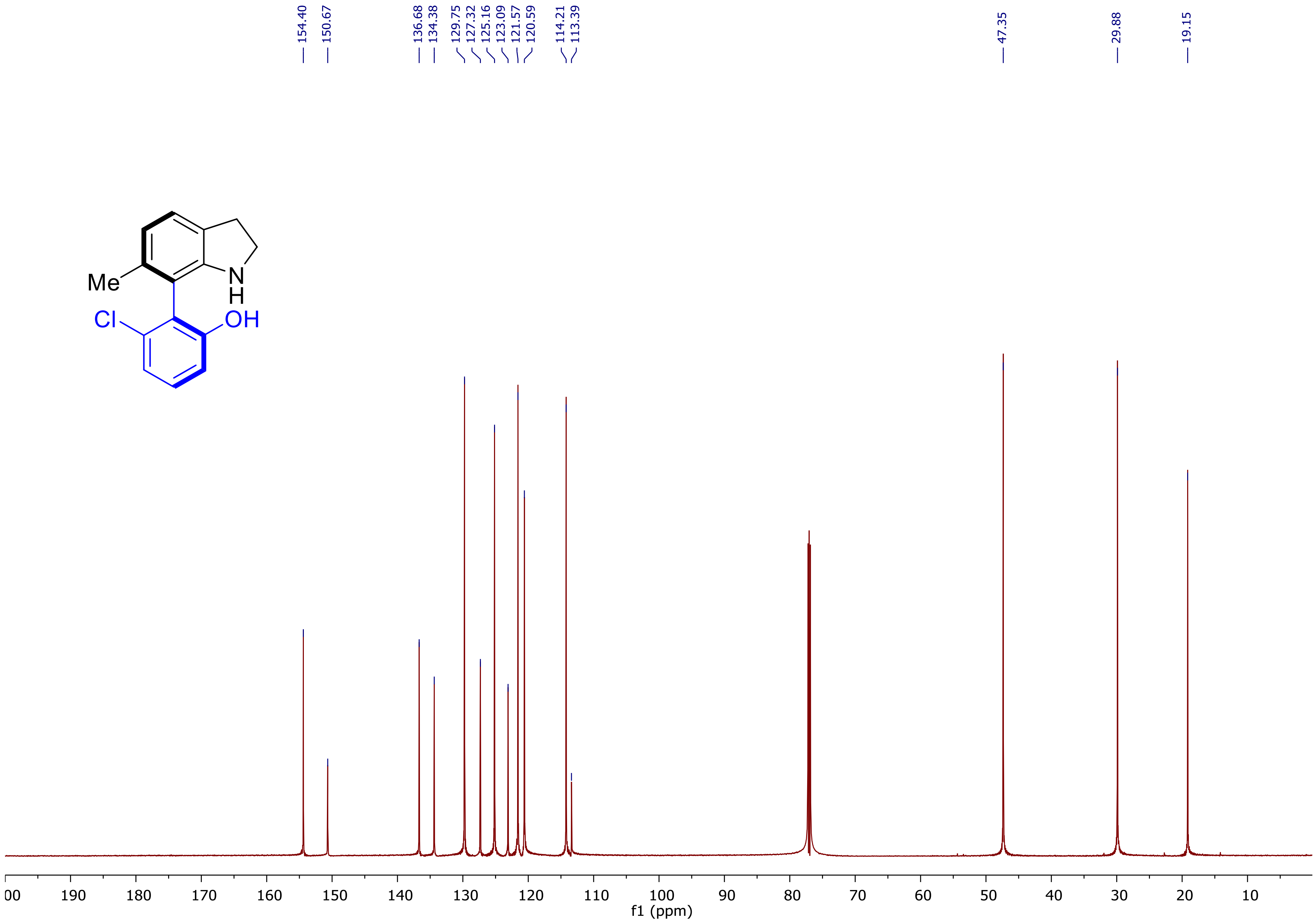

^{1}H-NMR (MeOD): *(R)-3-chloro-2-(6-methyl-1H-indol-7-yl)phenol* (***3zb***)

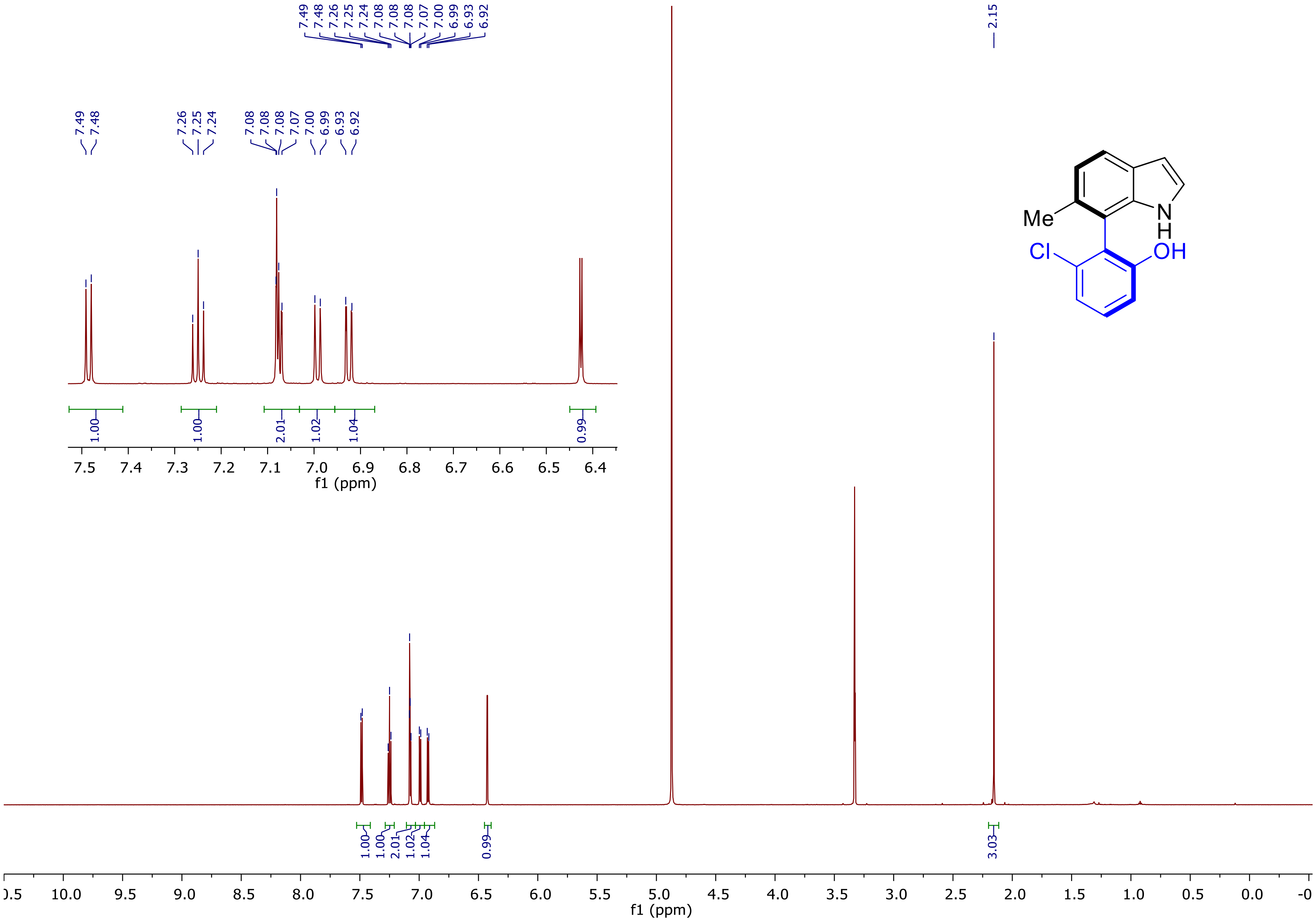

^{13}C-NMR (MeOD): *(R)-3-chloro-2-(6-methyl-1H-indol-7-yl)phenol* (***3zb***)

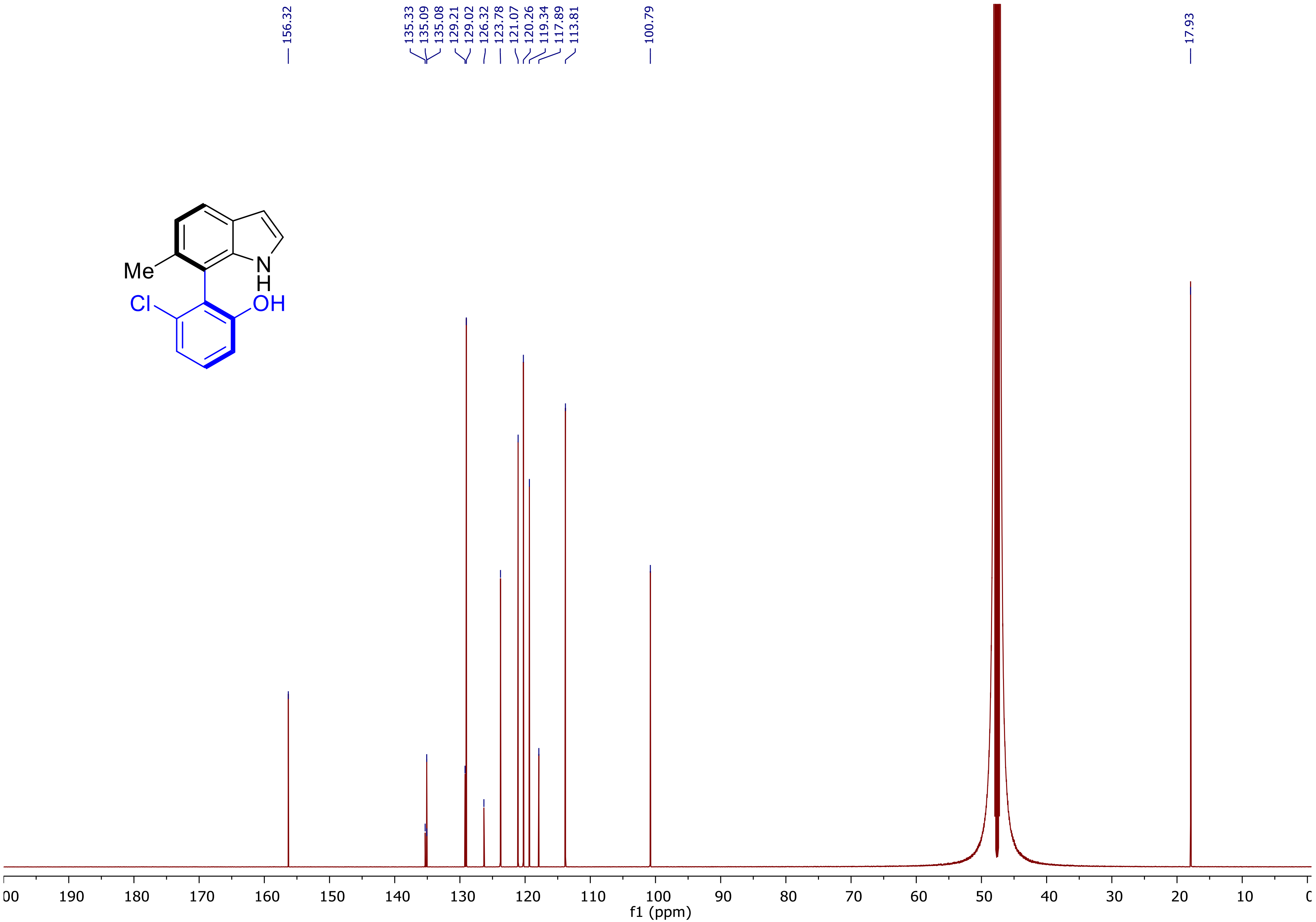

¹H-NMR (MeOD): *(S)-2-(2-amino-4-chloropyridin-3-yl)-3-fluorophenol* (***3zc***)

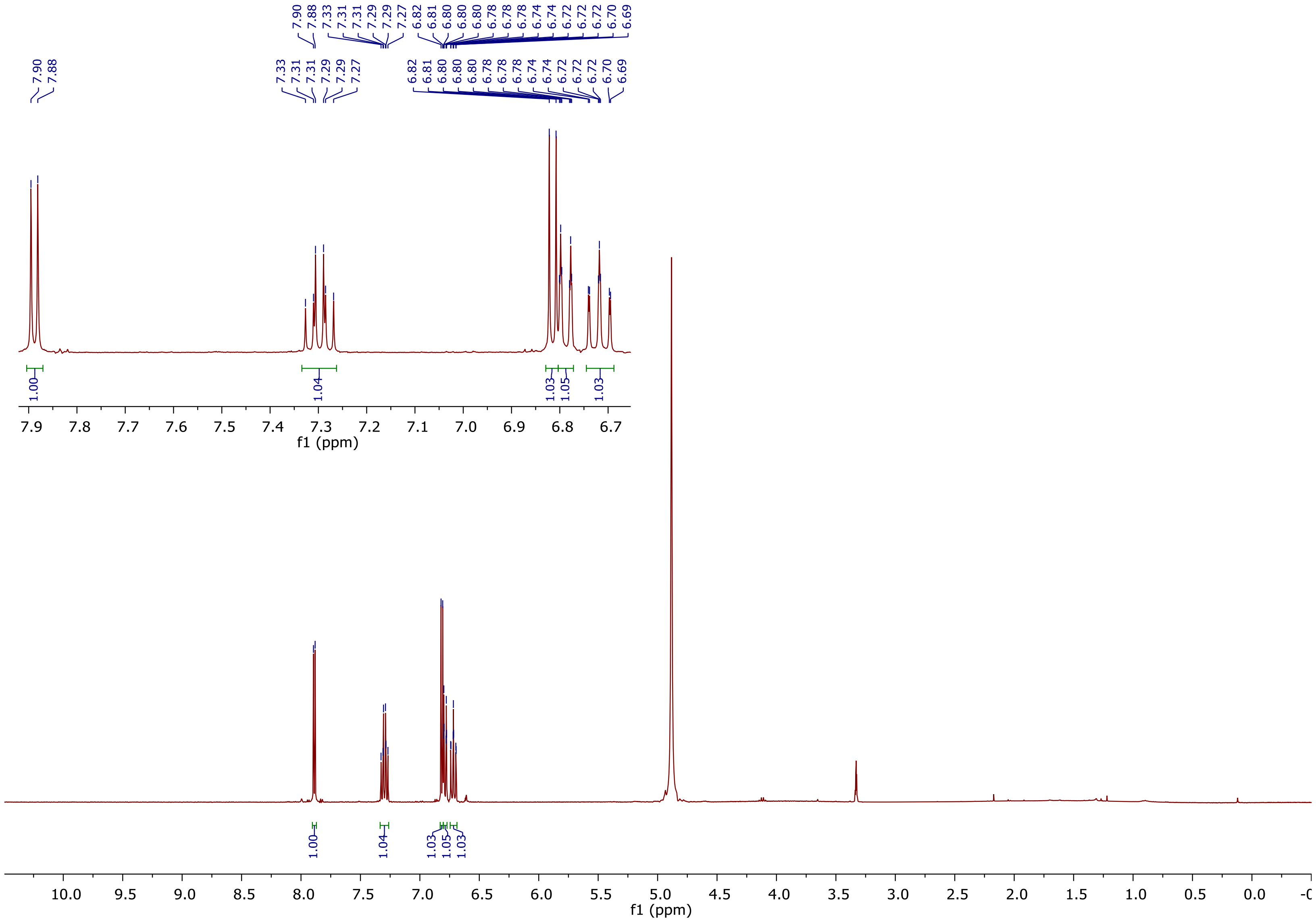

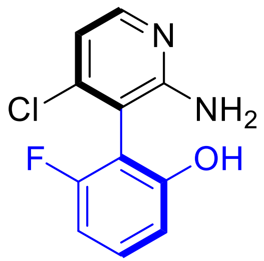

^{19}F-NMR (MeOD): *(S)-2-(2-amino-4-chloropyridin-3-yl)-3-fluorophenol (**3zc**)*

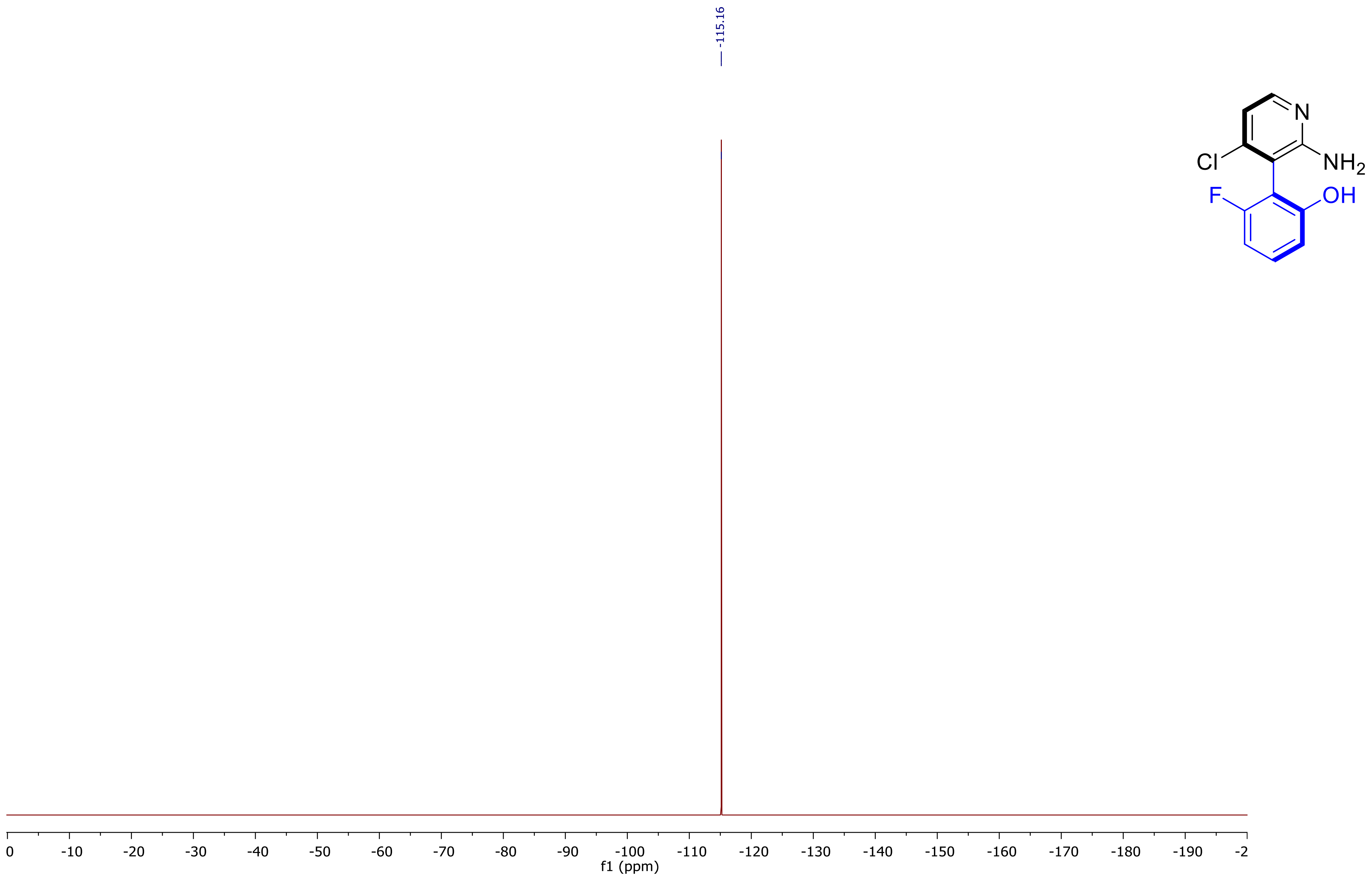

^{13}C-NMR (MeOD): *(S)-2-(2-amino-4-chloropyridin-3-yl)-3-fluorophenol (**3zc**)*

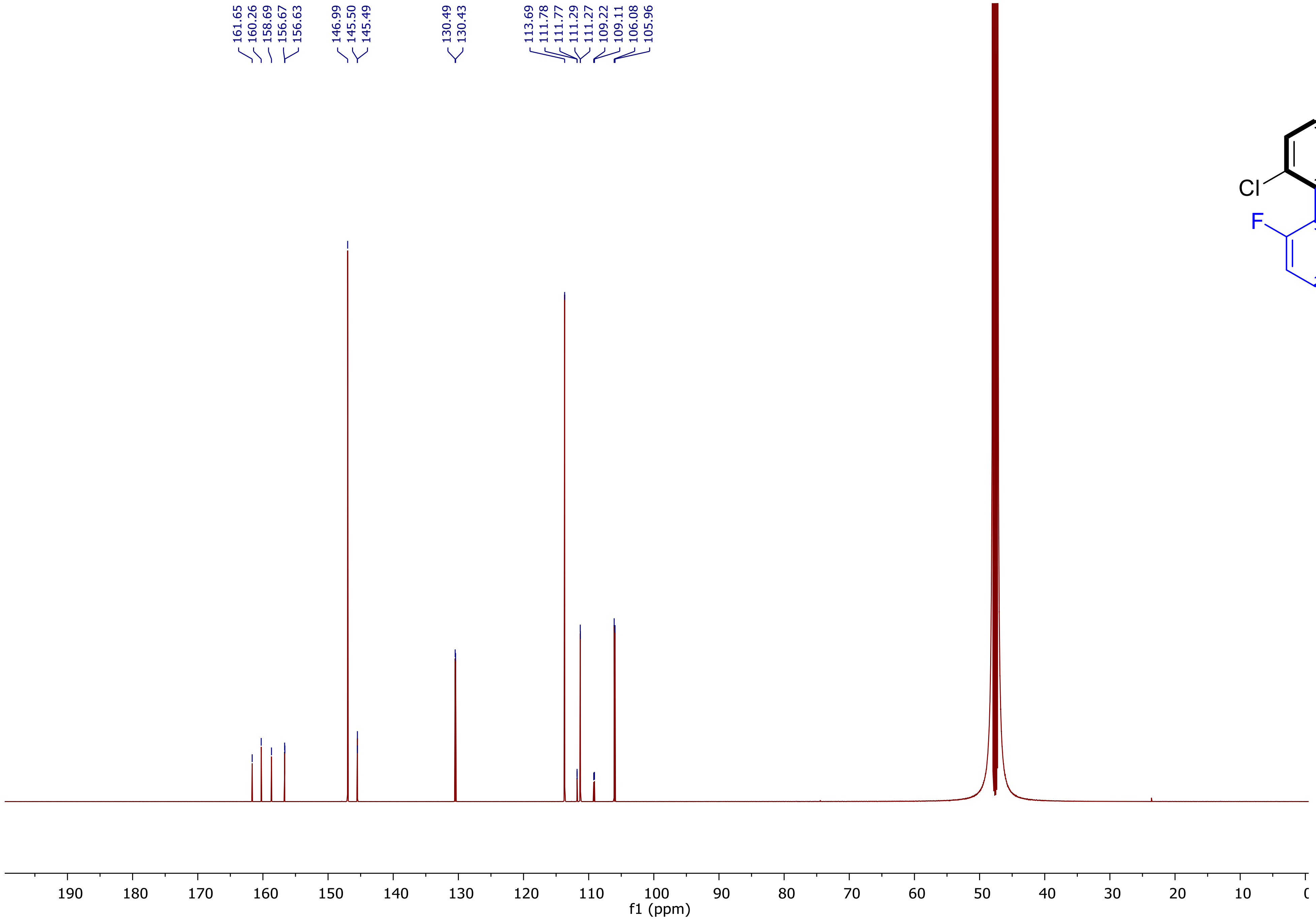

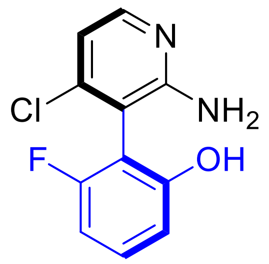

^{1}**H-NMR** ($CDCl_3$): (S)-6,6'-dichloro-[1,1'-biphenyl]-2,2'-diol (**4a**)

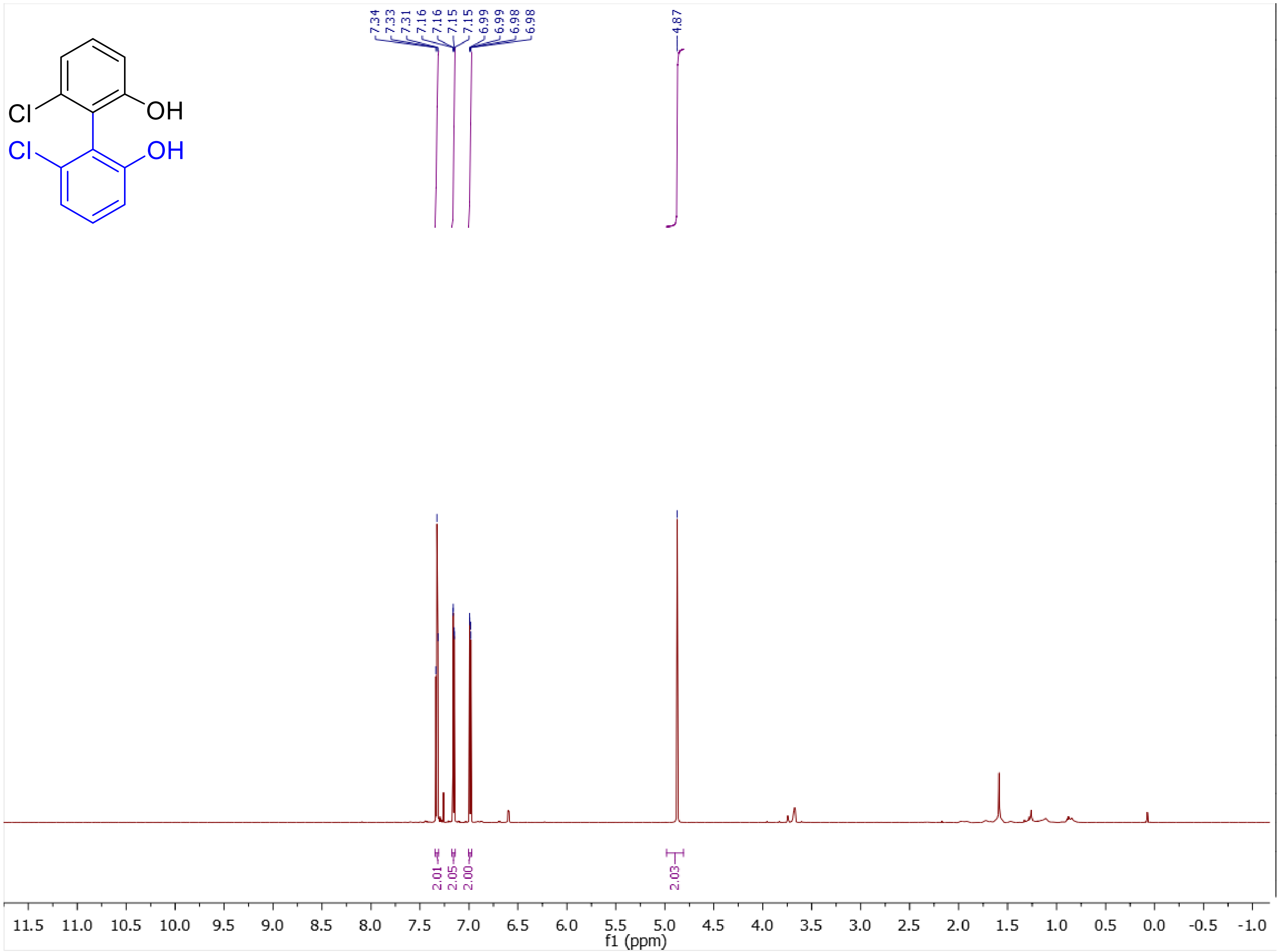

^{13}C-NMR (CDCl$_3$): (S)-6,6'-dichloro-[1,1'-biphenyl]-2,2'-diol (**4a**)

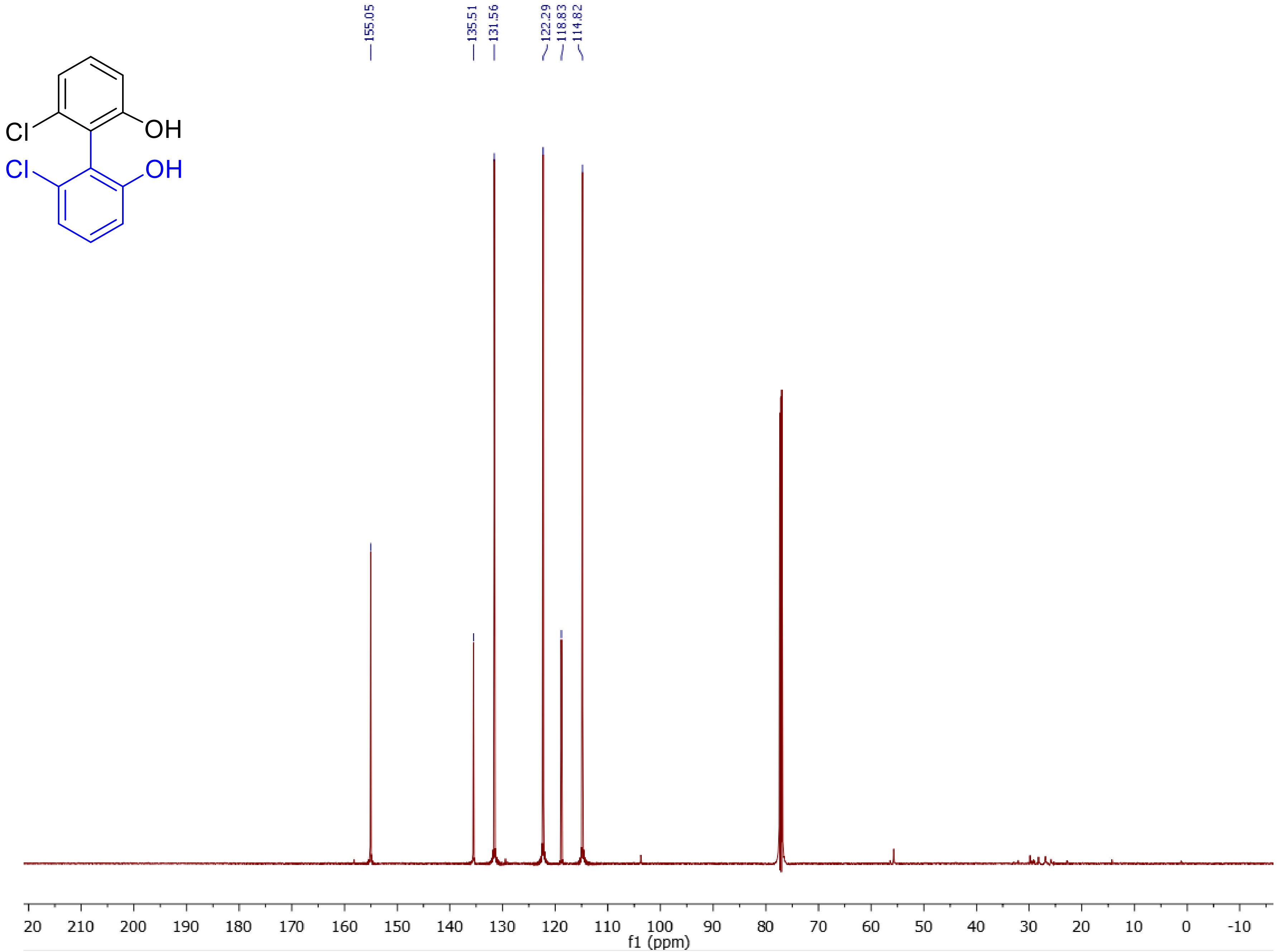

¹H-NMR ($CDCl_3$): (S)-6,6'-dimethyl-[1,1'-biphenyl]-2,2'-diol (**4b**)

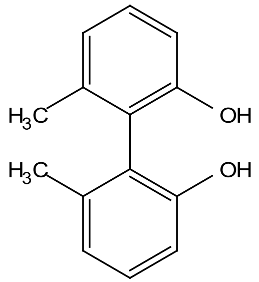

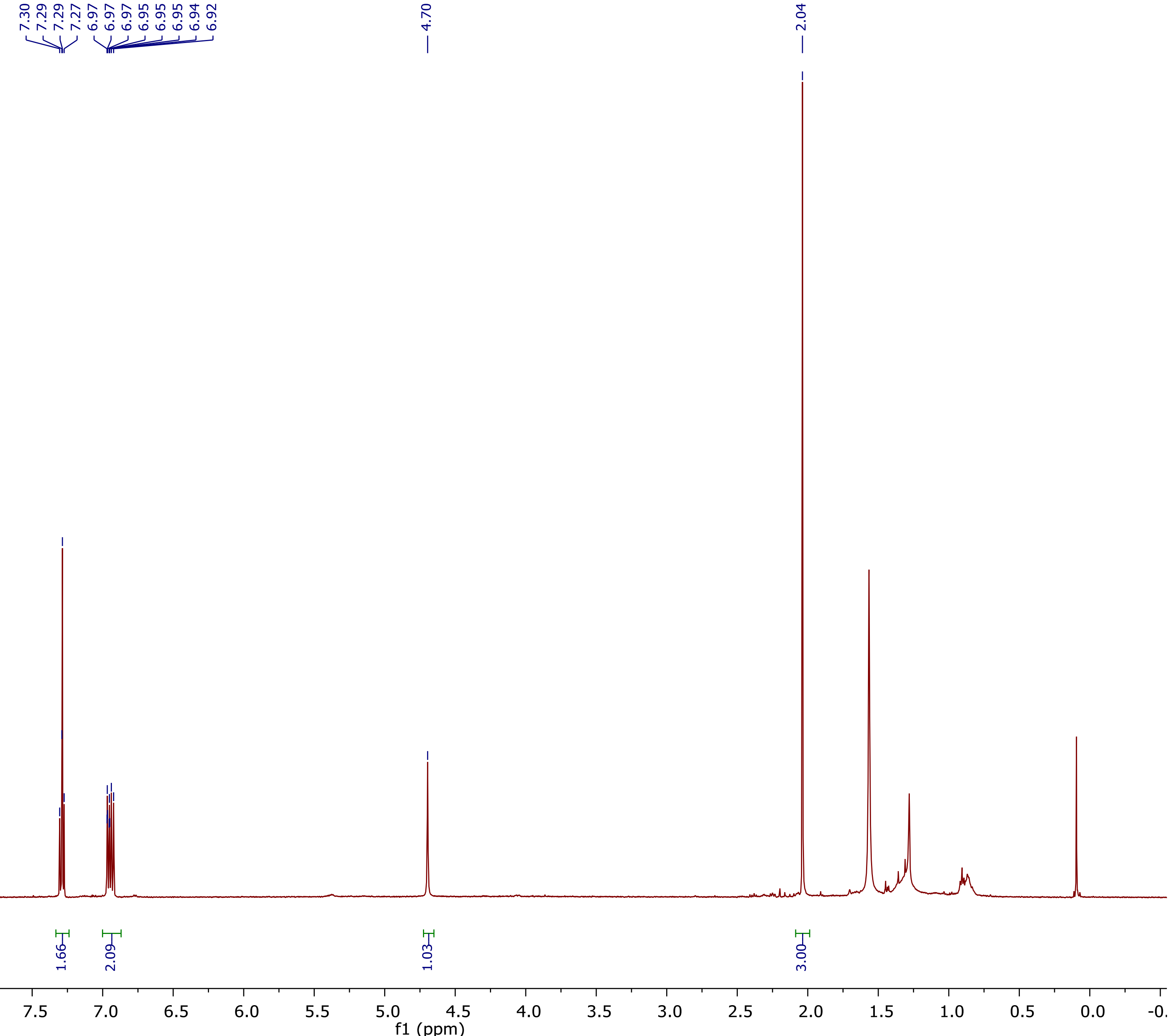

^{13}C-NMR ($CDCl_3$): (S)-6,6'-dimethyl-[1,1'-biphenyl]-2,2'-diol (**4b**)

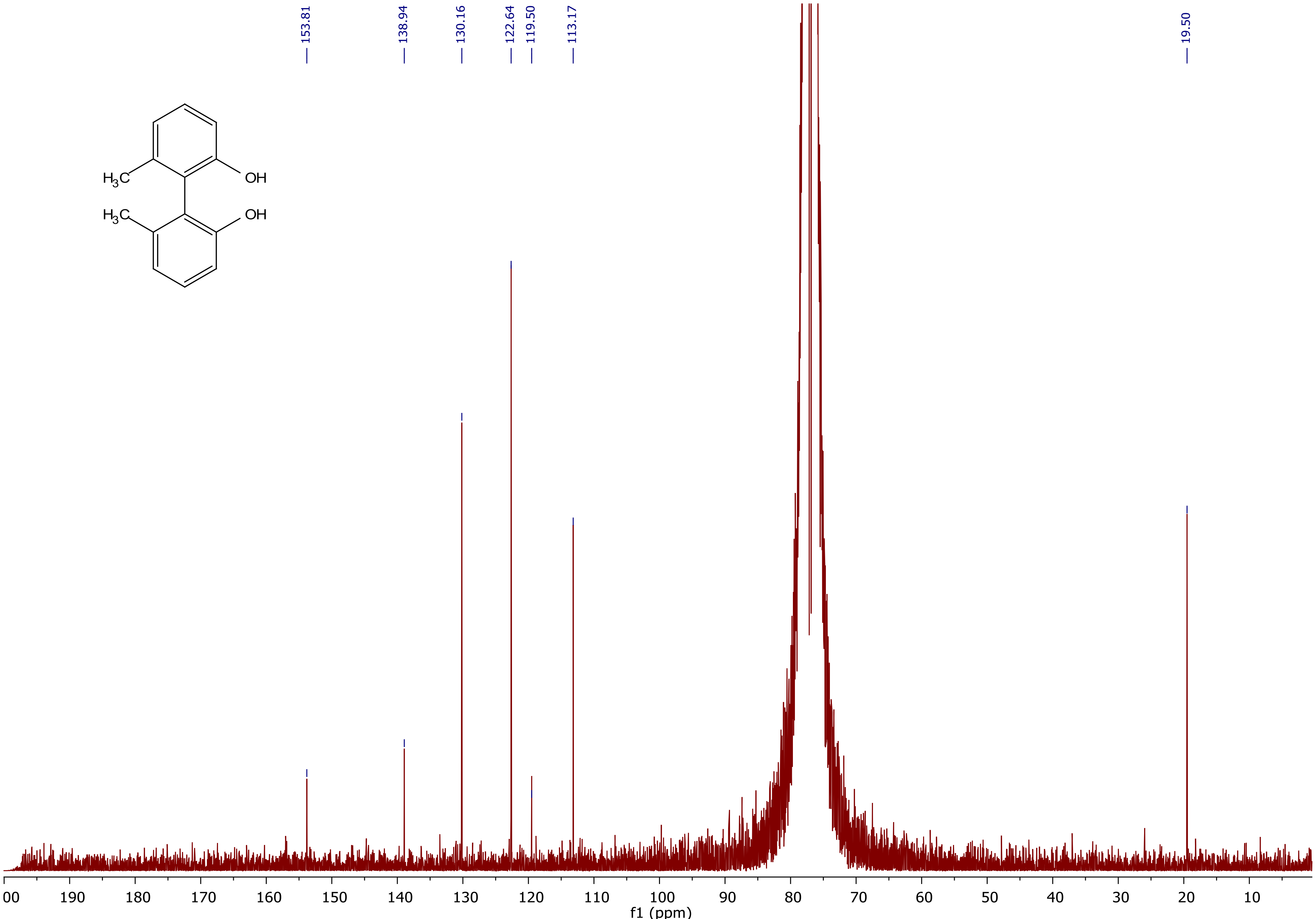

^{1}H-NMR (MeOD): (R)-1-(2,3-difluoro-6-hydroxyphenyl)-5,6,7,8-tetrahydronaphthalen-2-ol (**4c**)

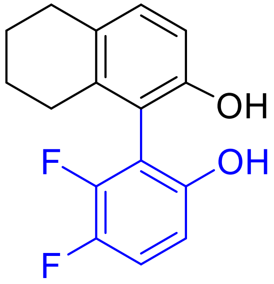

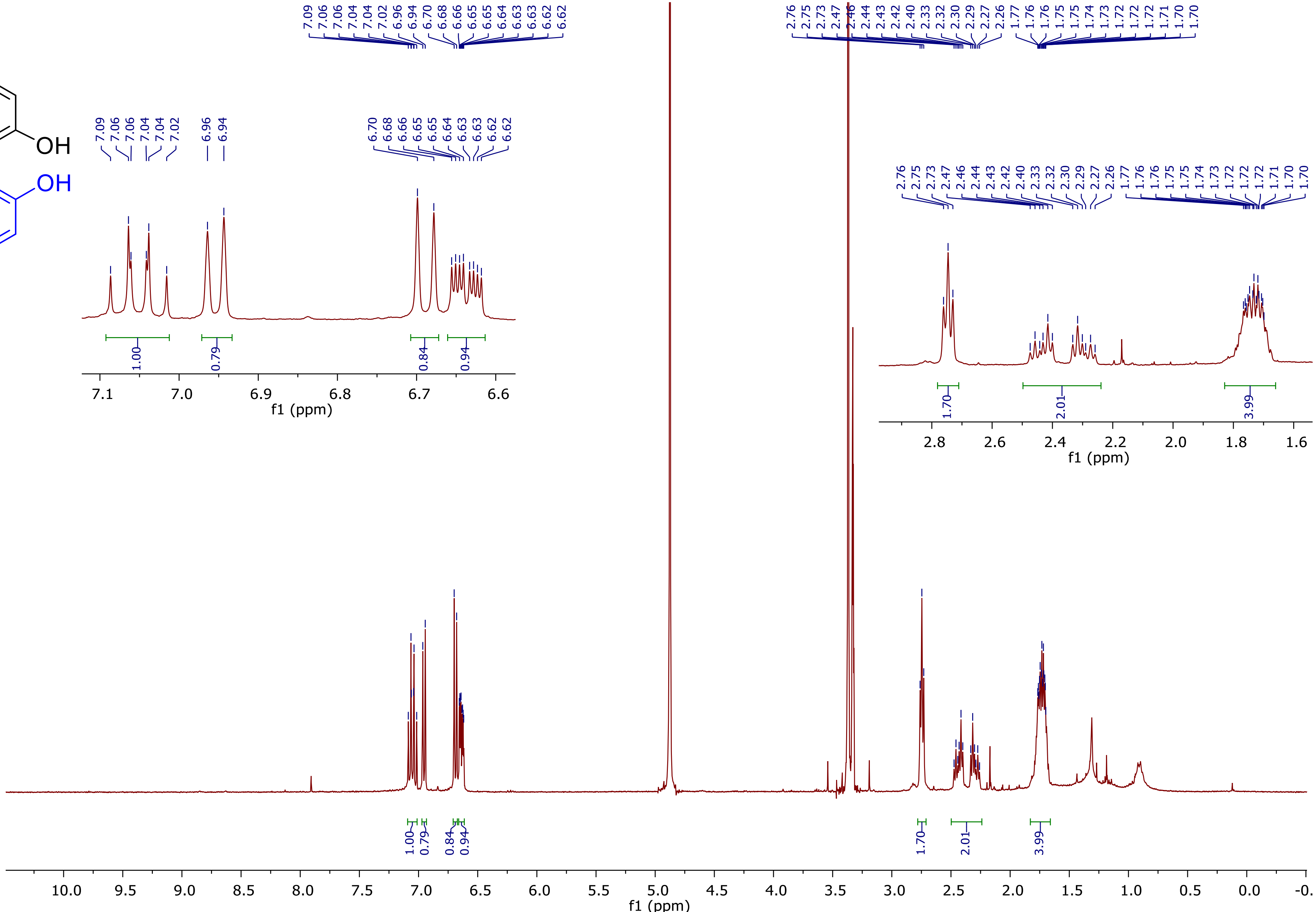

^{19}F-NMR ($CDCl_3$): (R)-1-(2,3-difluoro-6-hydroxyphenyl)-5,6,7,8-tetrahydronaphthalen-2-ol (**4c**)

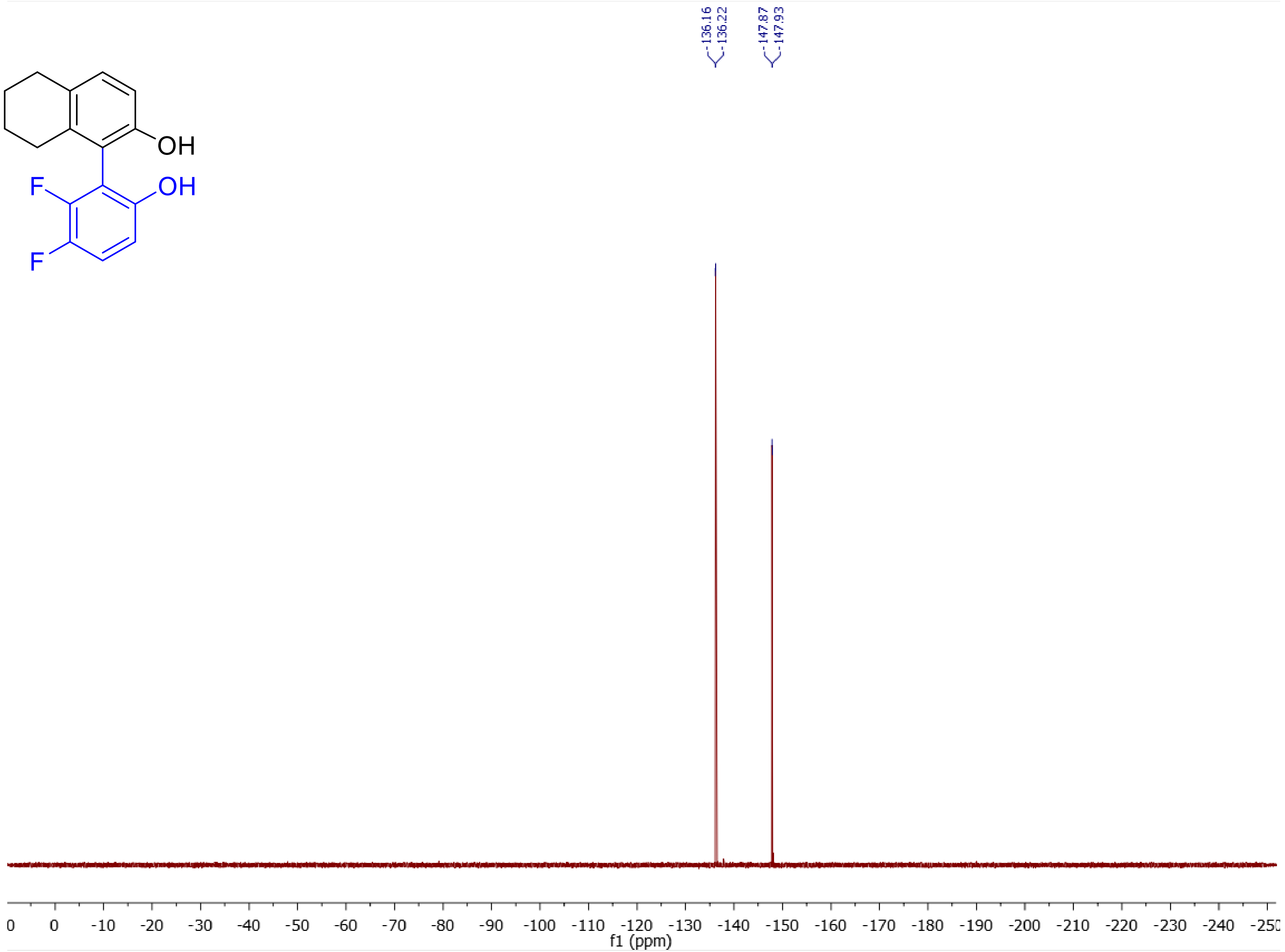

^{13}C-NMR ($CDCl_3$): (R)-1-(2,3-difluoro-6-hydroxyphenyl)-5,6,7,8-tetrahydronaphthalen-2-ol (**4c**)

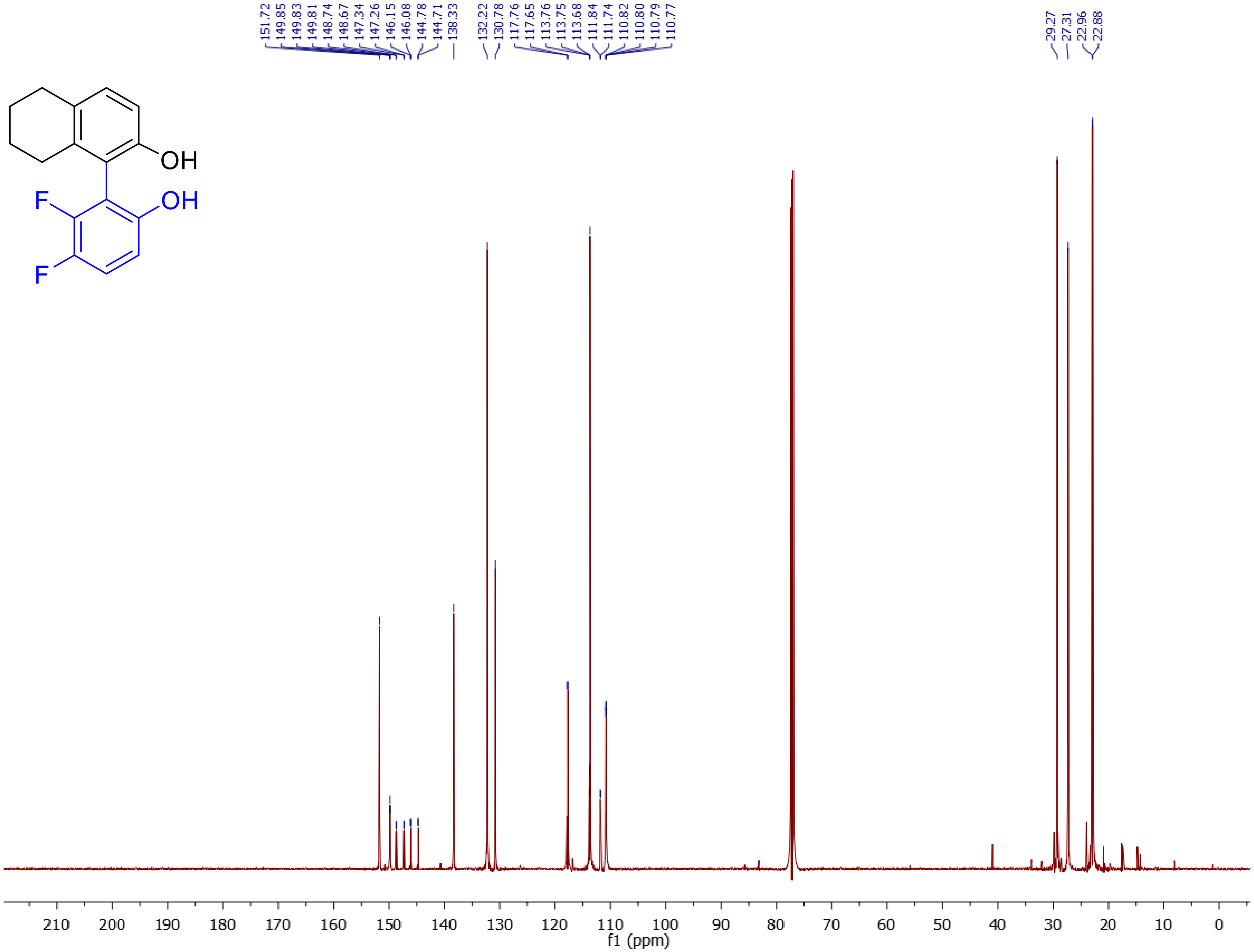

¹H-NMR ($CDCl_3$): (R)-5,6'-dichloro-6-methyl-[1,1'-biphenyl]-2,2'-diol (**4d**)

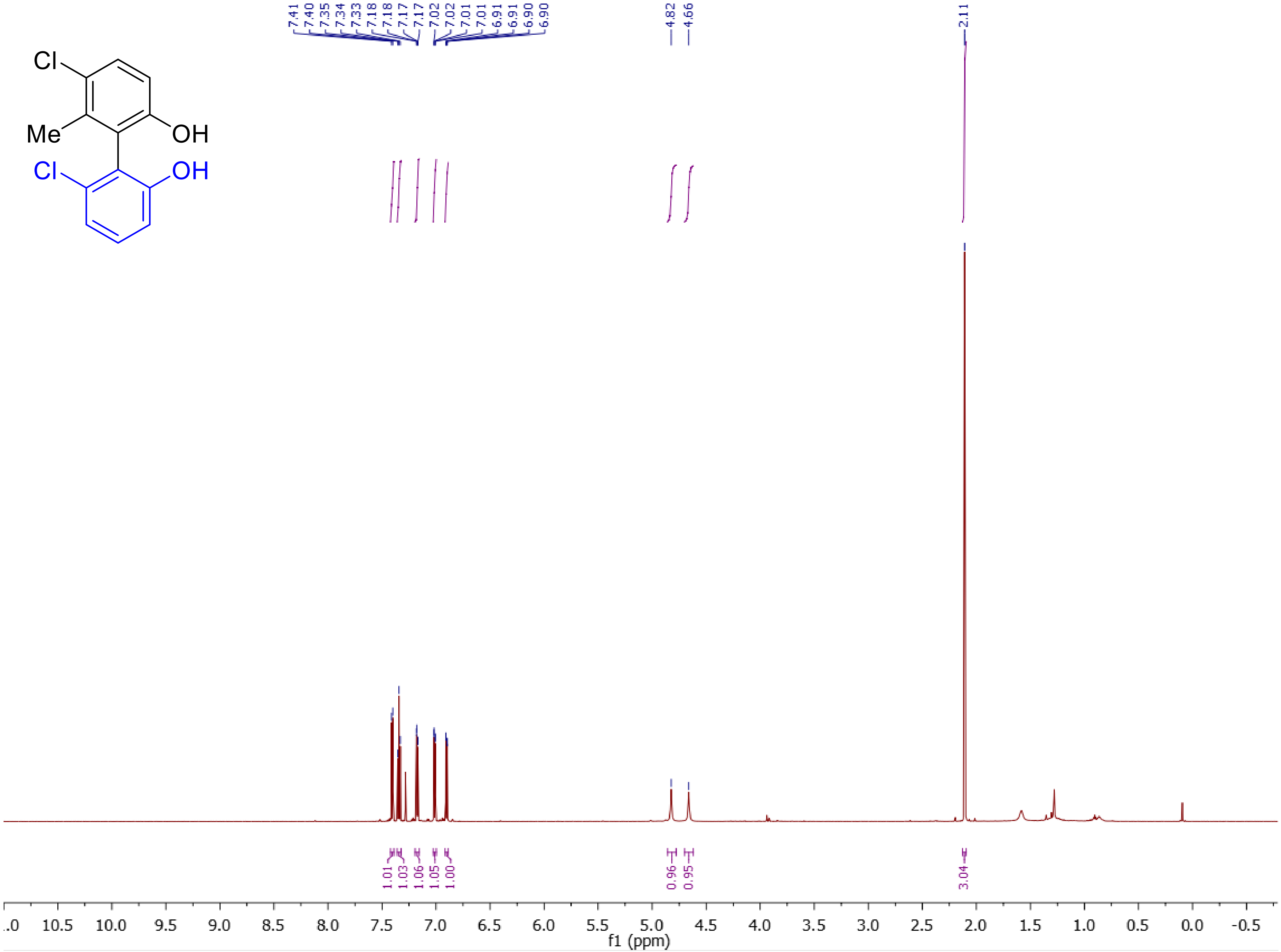

^{13}**C-NMR** ($CDCl_3$): (R)-5,6'-dichloro-6-methyl-[1,1'-biphenyl]-2,2'-diol (**4d**)

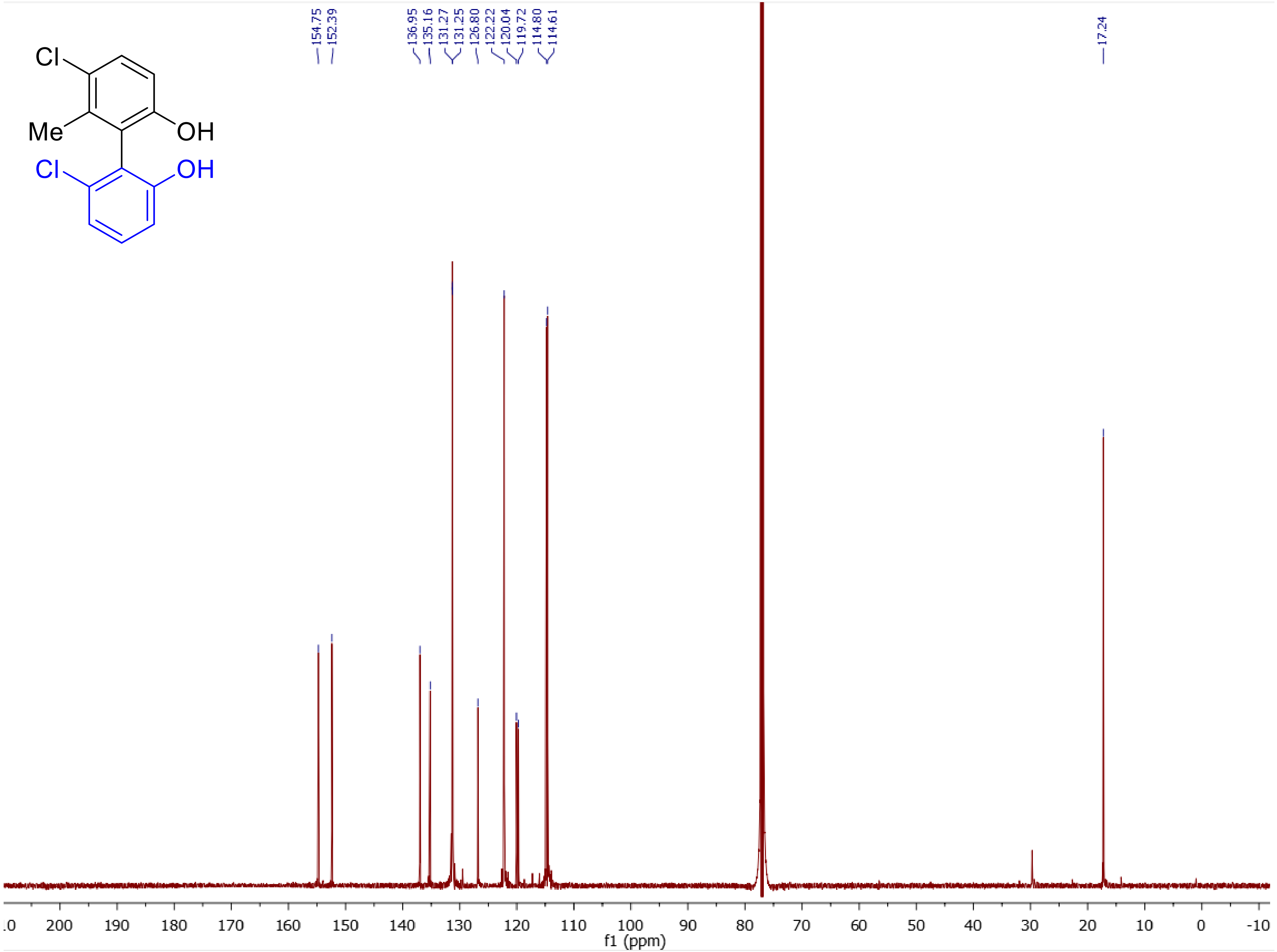

^{1}H-NMR ($CDCl_3$): 6-fluoro-2'-methoxy-6'-methyl-[1,1'-biphenyl]-2-ol (**4e**)

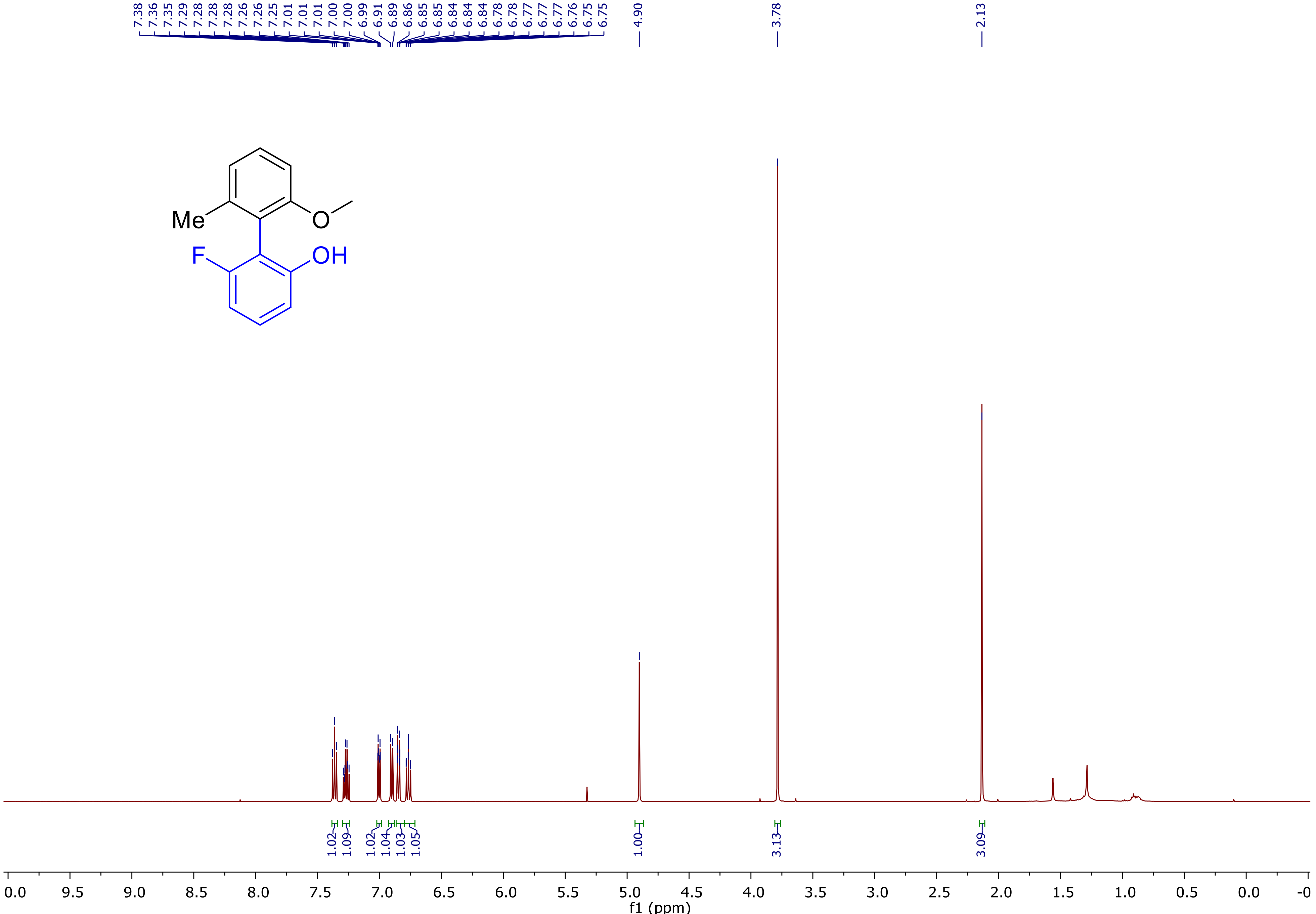

^{13}C-NMR ($CDCl_3$): 6-fluoro-2'-methoxy-6'-methyl-[1,1'-biphenyl]-2-ol (**4e**)

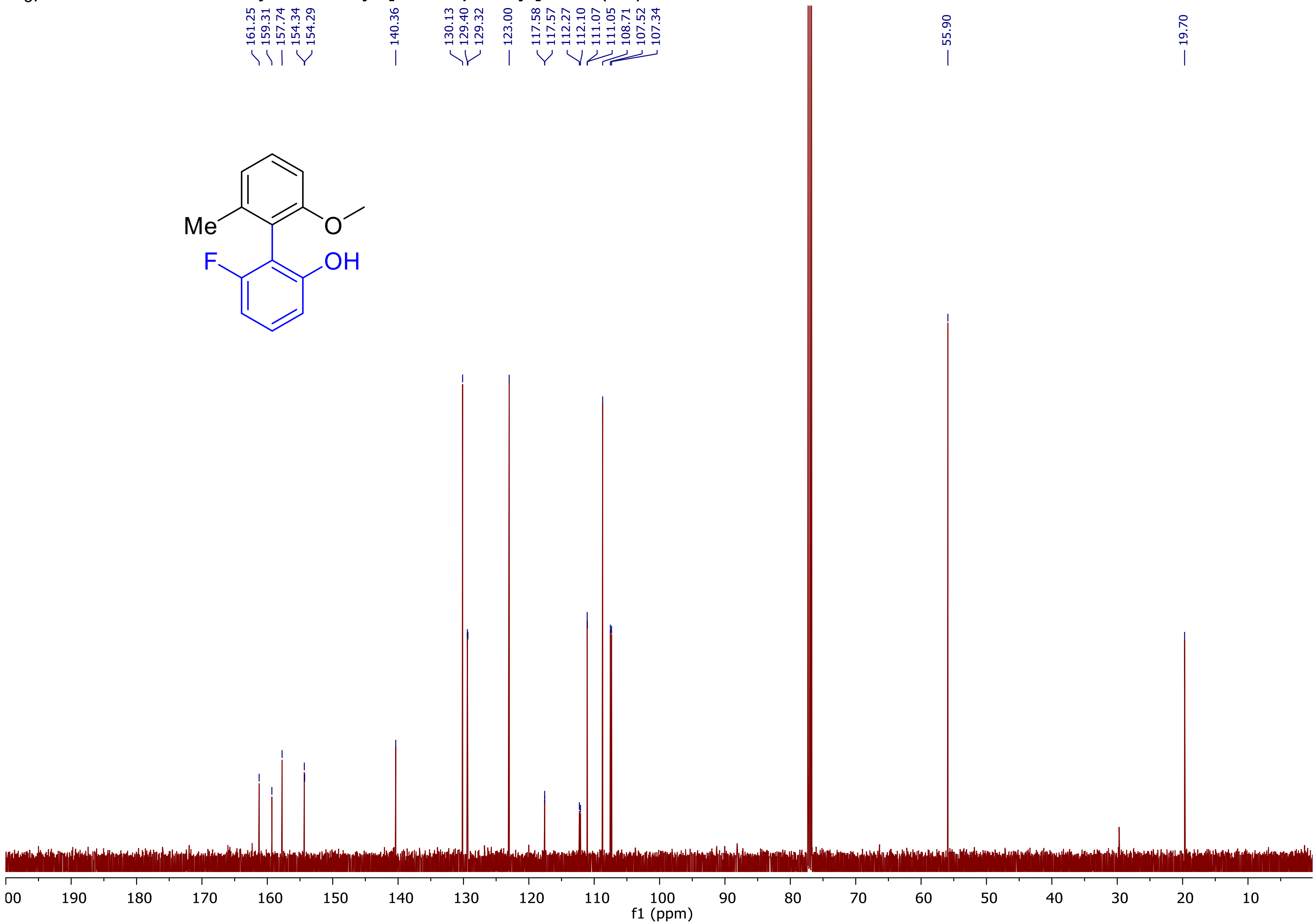

^{1}H-NMR ($CDCl_3$): (S)-2'-chloro-6-fluoro-6'-methoxy-[1,1'-biphenyl]-2-ol (**4f**)

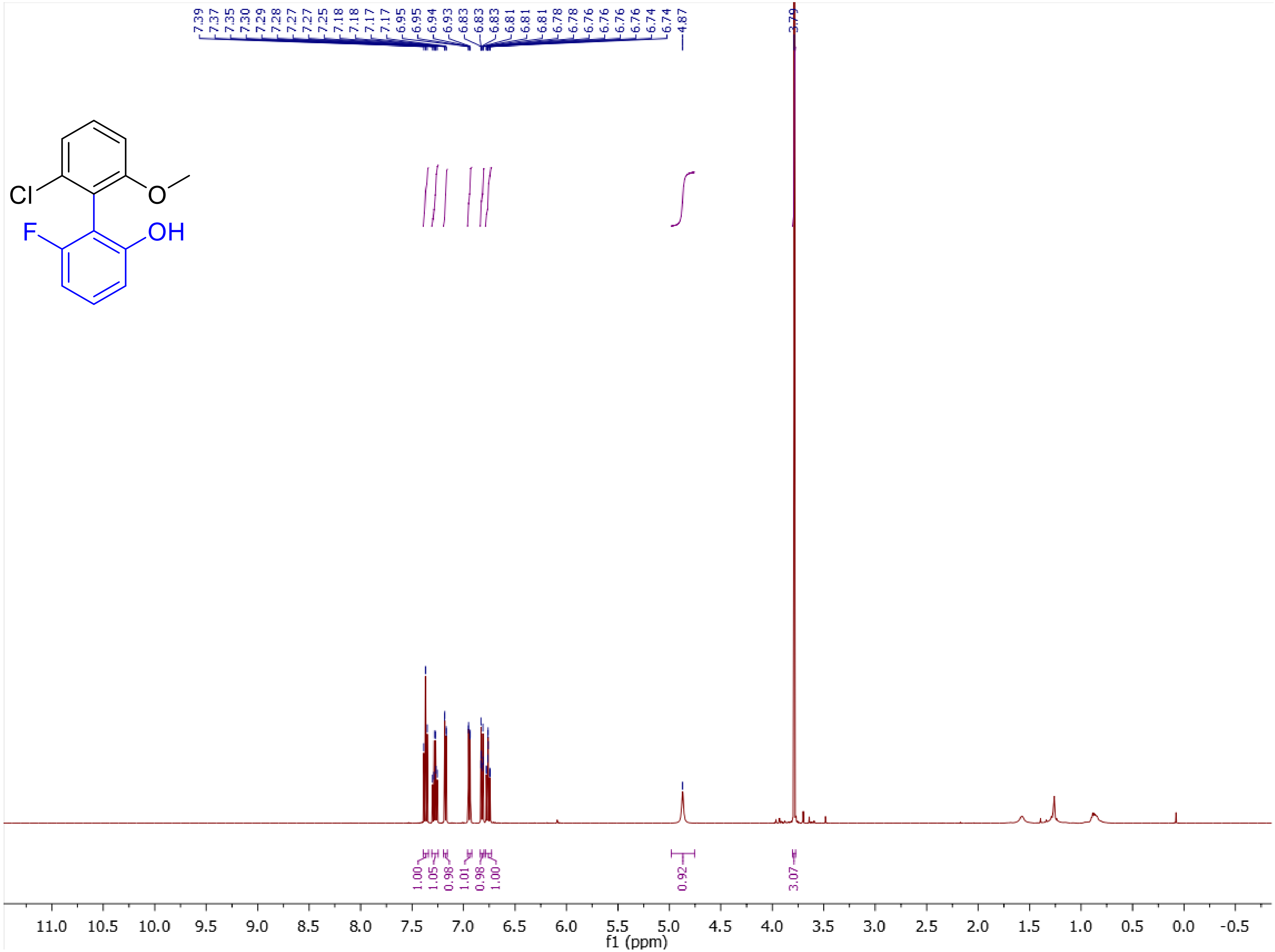

^{19}F-NMR ($CDCl_3$): (S)-2'-chloro-6-fluoro-6'-methoxy-[1,1'-biphenyl]-2-ol (**4f**)

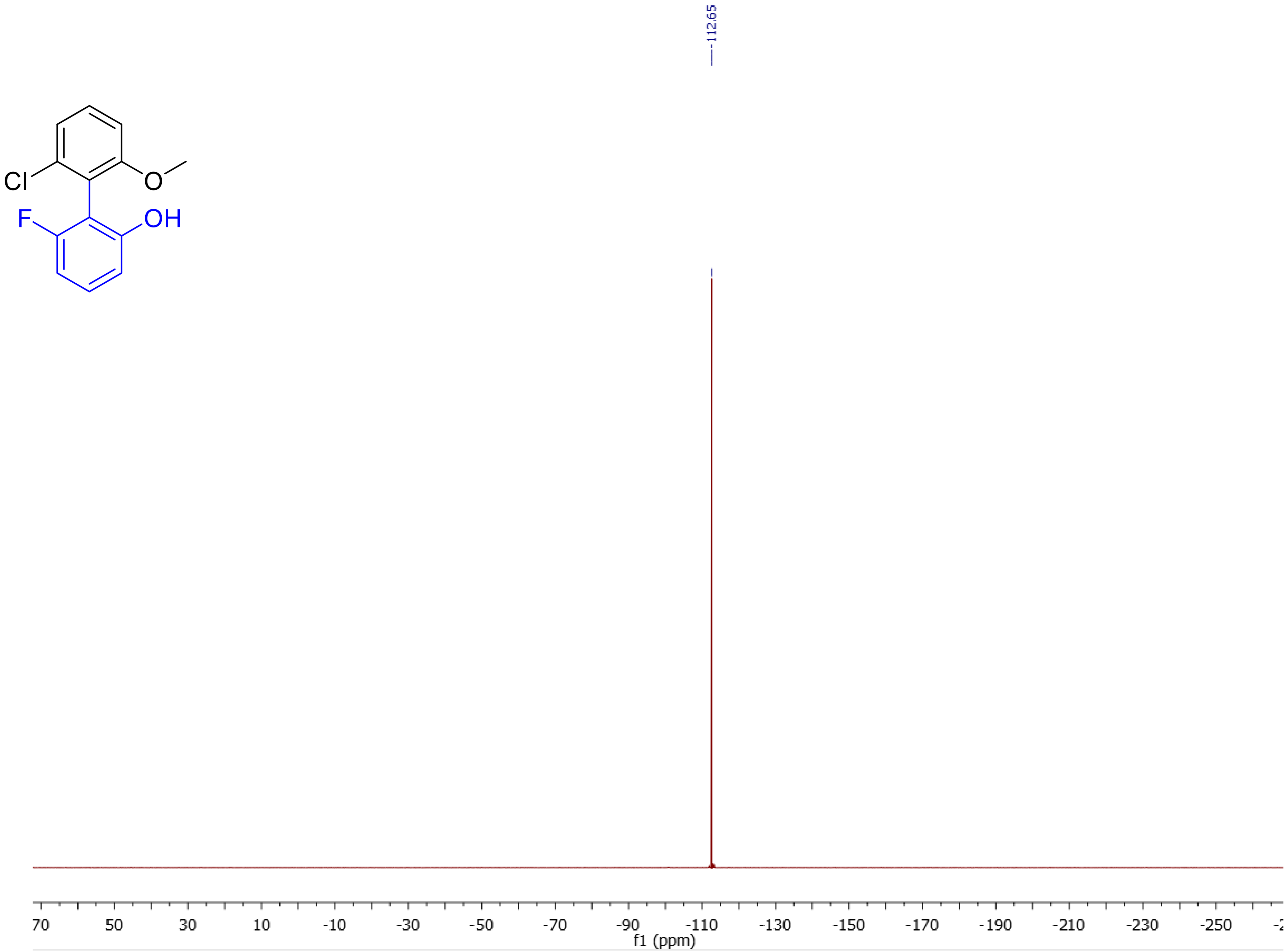

^{13}C-NMR ($CDCl_3$): (S)-2'-chloro-6-fluoro-6'-methoxy-[1,1'-biphenyl]-2-ol (**4f**)

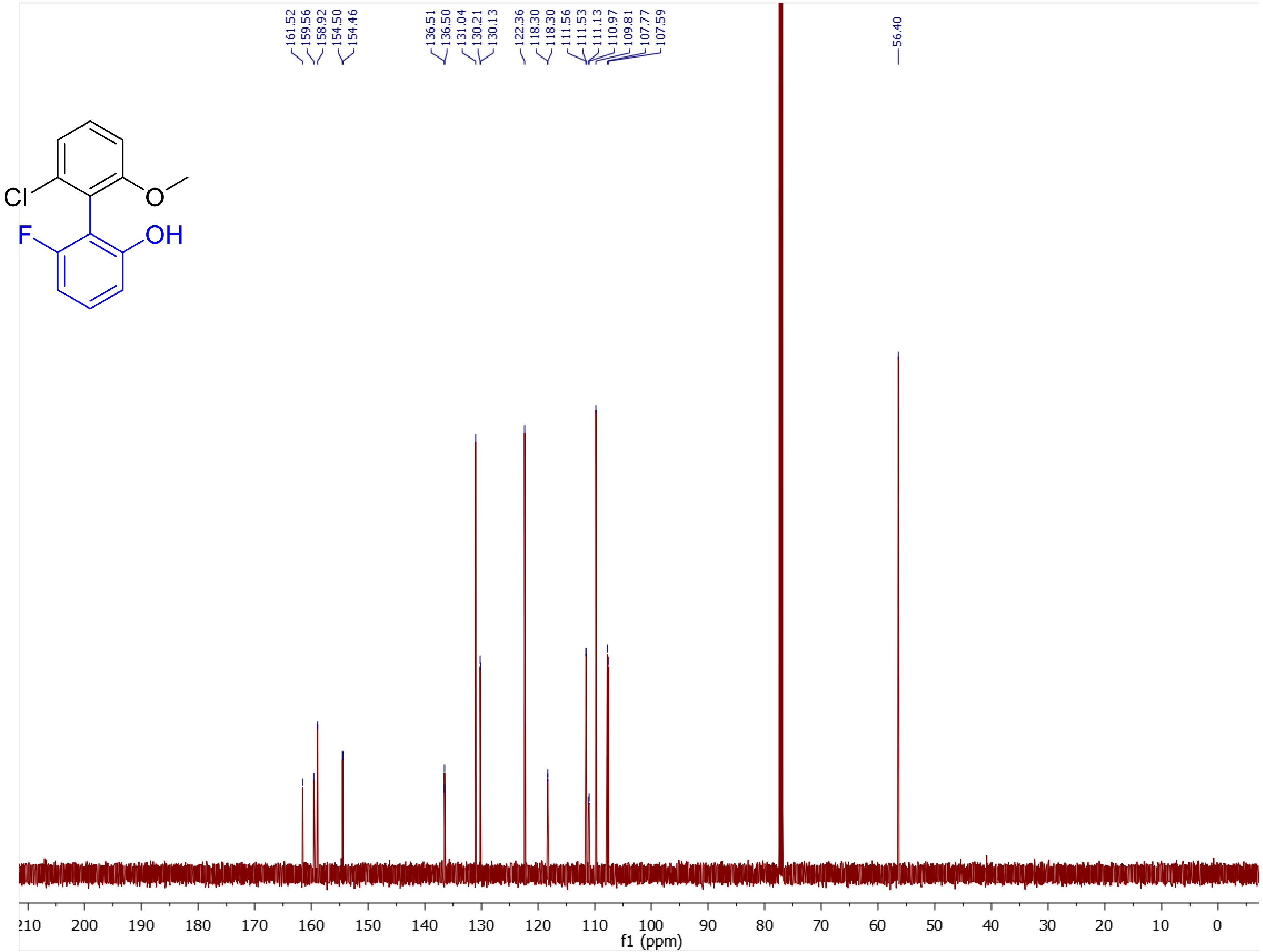

^{1}H-NMR ($CDCl_3$): (R)-3-fluoro-2-(2-methoxy-5,6,7,8-tetrahydronaphthalen-1-yl)phenol (**4g**)

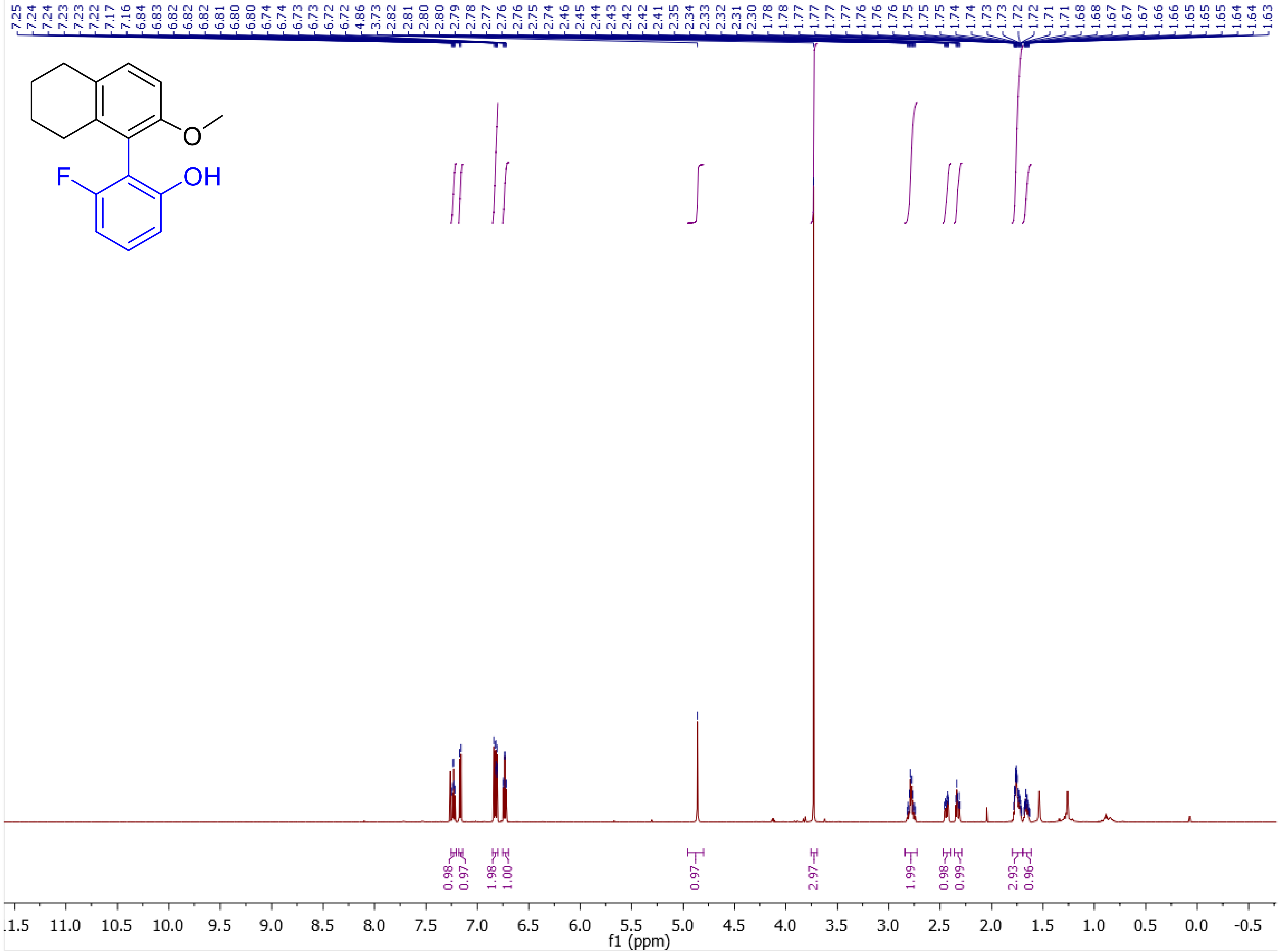

^{19}F-NMR ($CDCl_3$): (R)-3-fluoro-2-(2-methoxy-5,6,7,8-tetrahydronaphthalen-1-yl)phenol (**4g**)

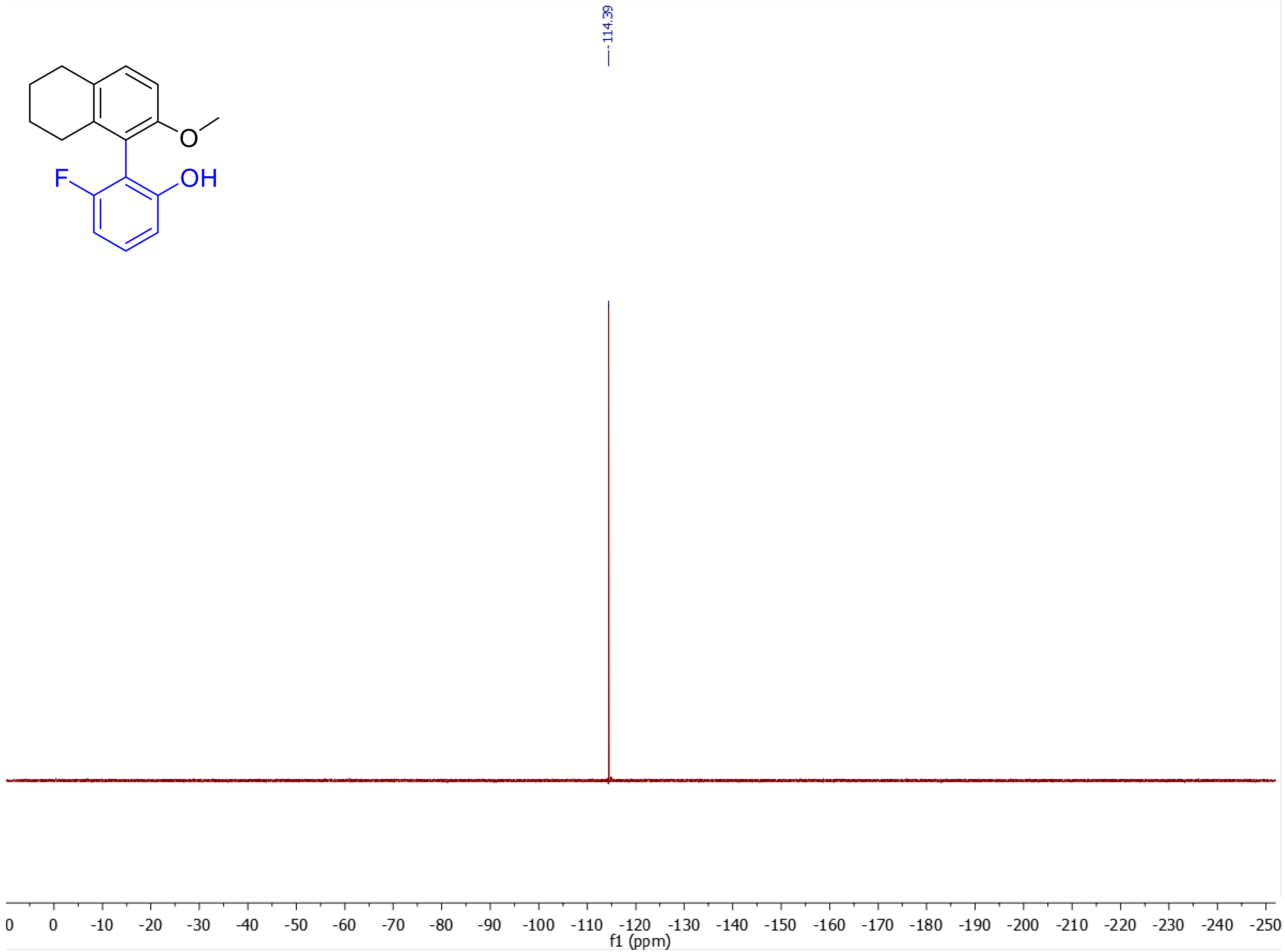

^{13}C-NMR (CDCl$_3$): (R)-3-fluoro-2-(2-methoxy-5,6,7,8-tetrahydronaphthalen-1-yl)phenol (**4g**)

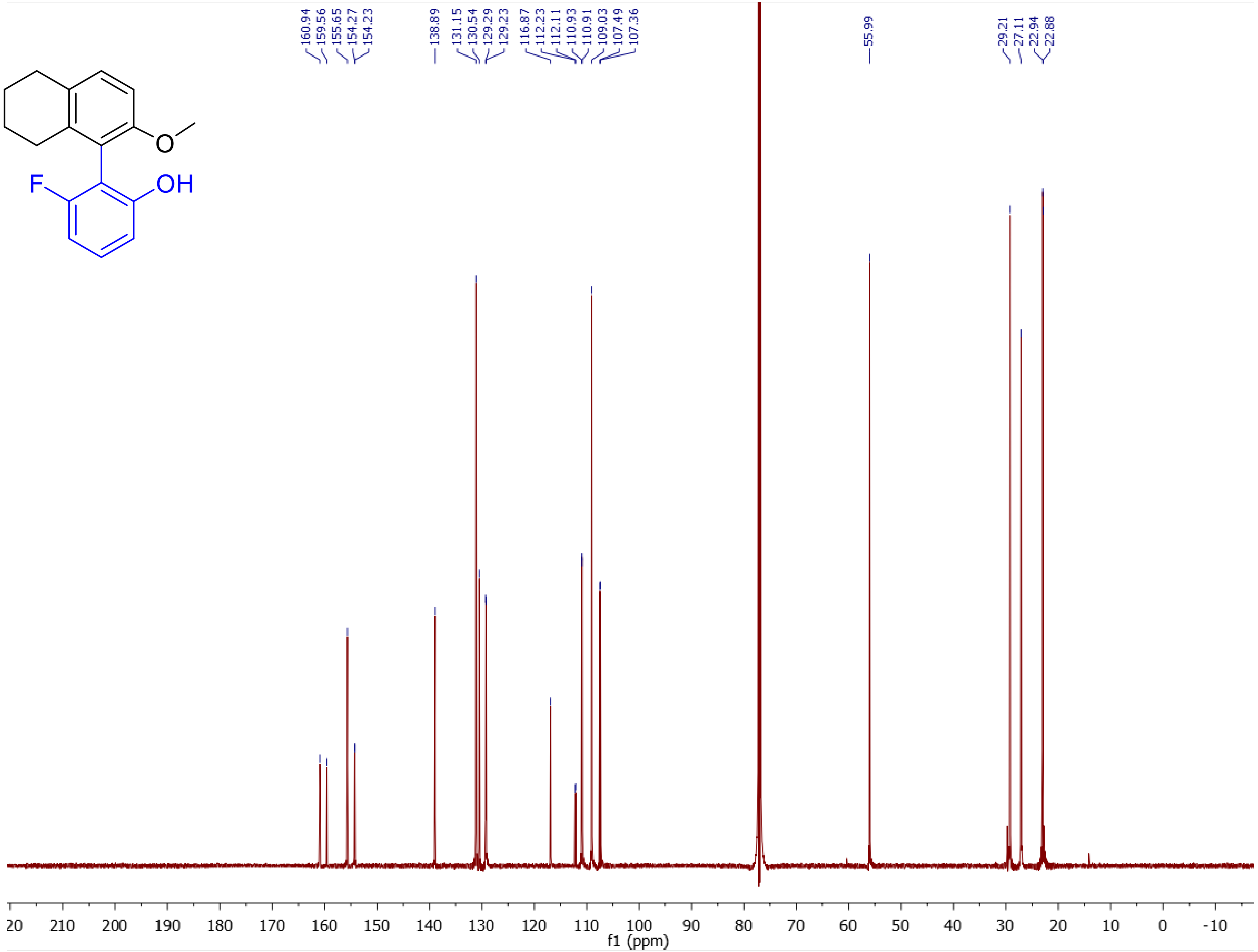

^{1}H-NMR ($CDCl_3$): (R)-5,6-difluoro-2'-methoxy-6'-methyl-[1,1'-biphenyl]-2-ol (**4h**)

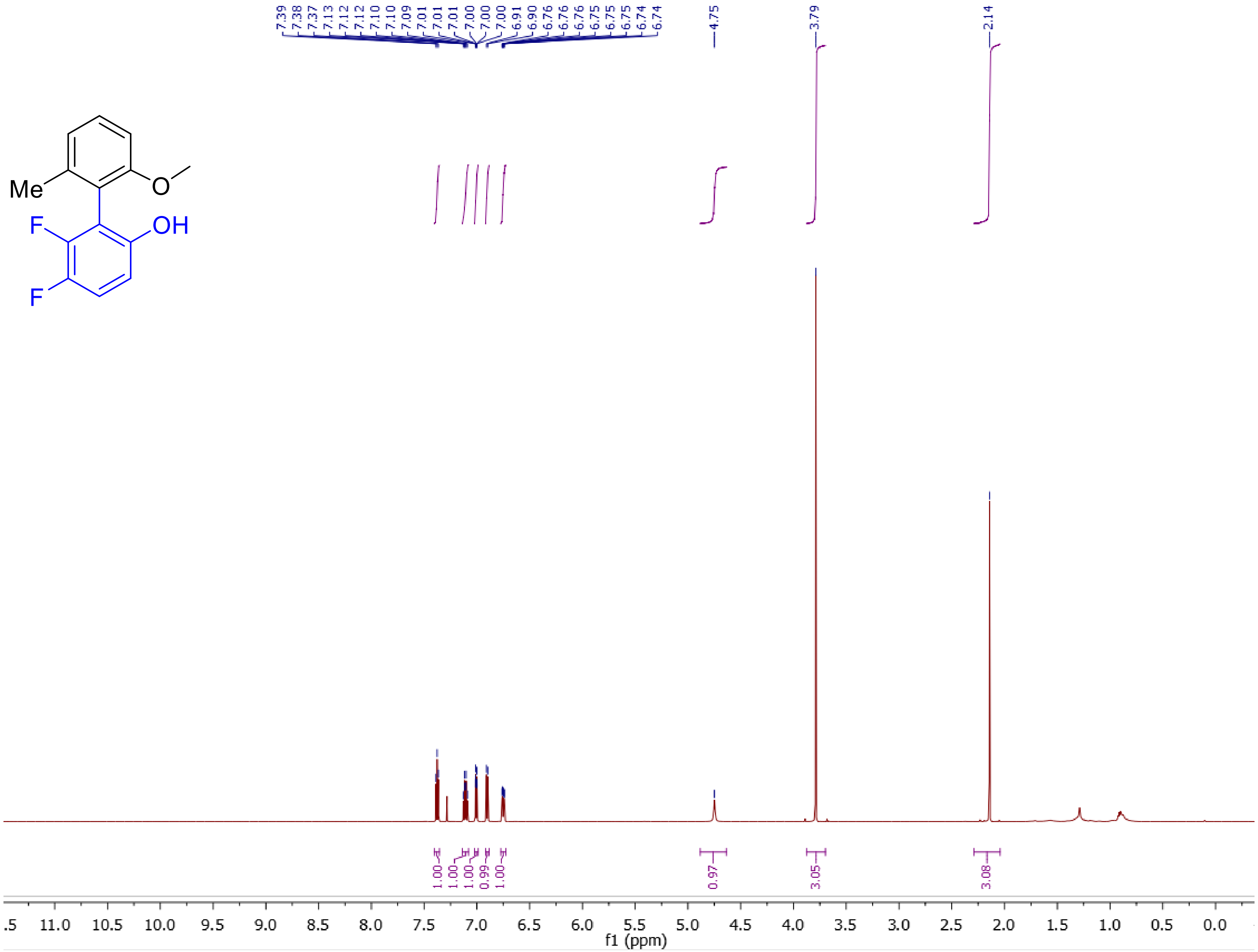

^{19}F-NMR ($CDCl_3$): (R)-5,6-difluoro-2'-methoxy-6'-methyl-[1,1'-biphenyl]-2-ol (**4h**)

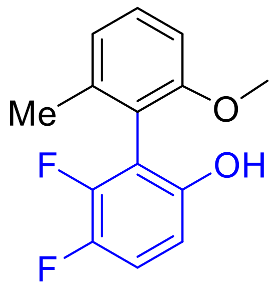

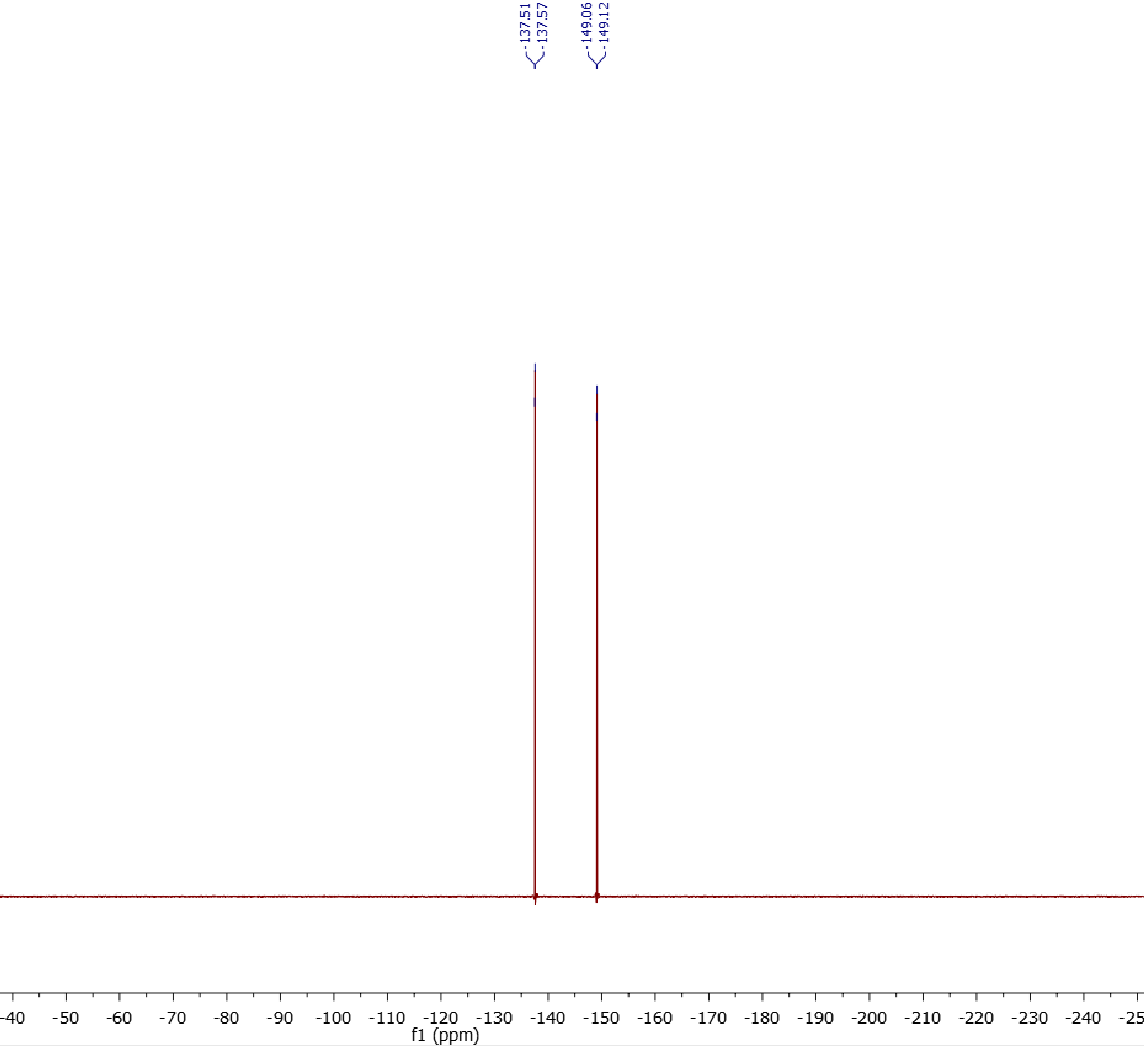

^{13}C-NMR ($CDCl_3$): (R)-5,6-difluoro-2'-methoxy-6'-methyl-[1,1'-biphenyl]-2-ol (**4h**)

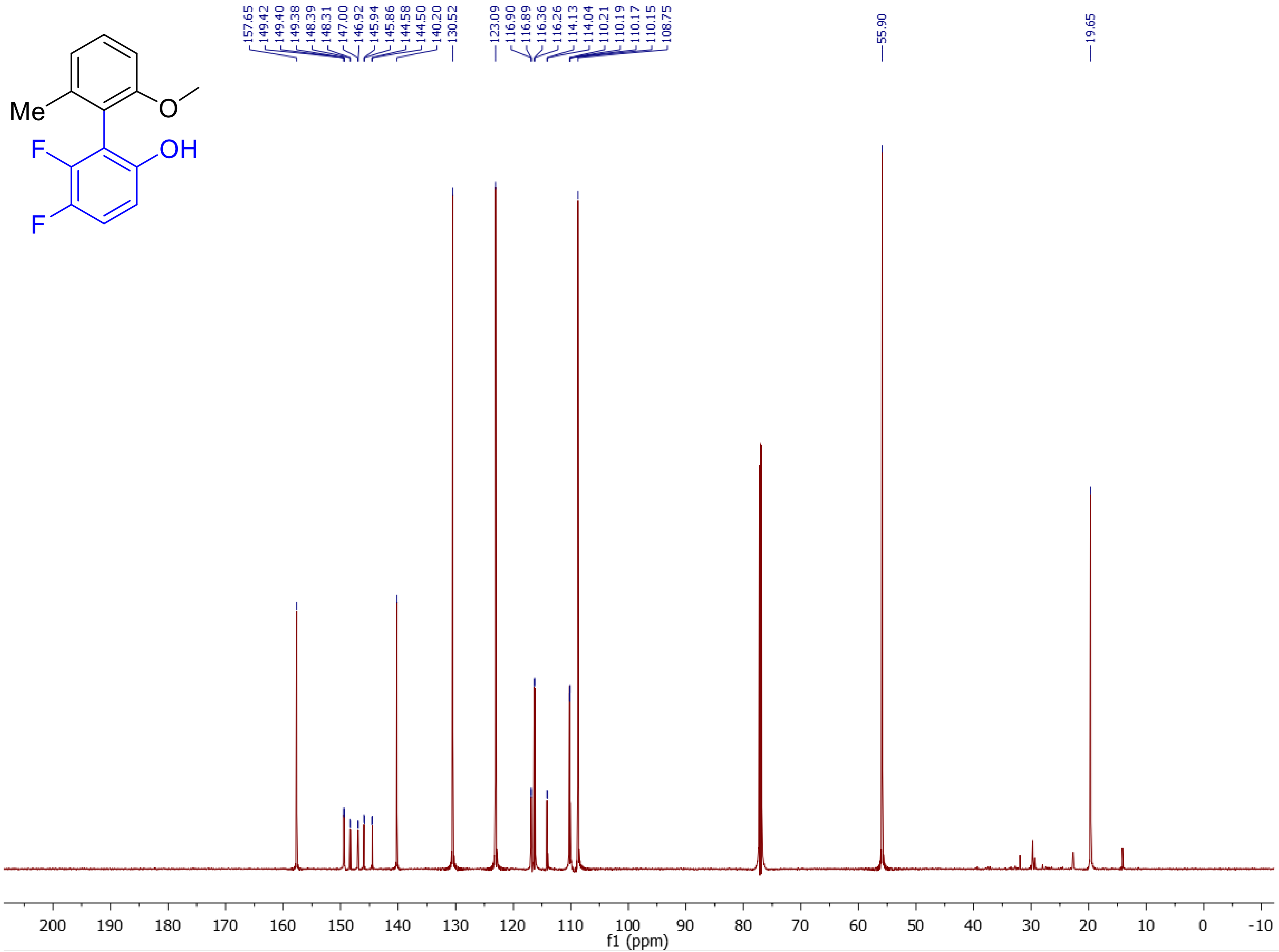

^{1}H-NMR ($CDCl_3$): (R)-6-fluoro-6'-methyl-[1,1'-biphenyl]-2,2'-diol (4i)

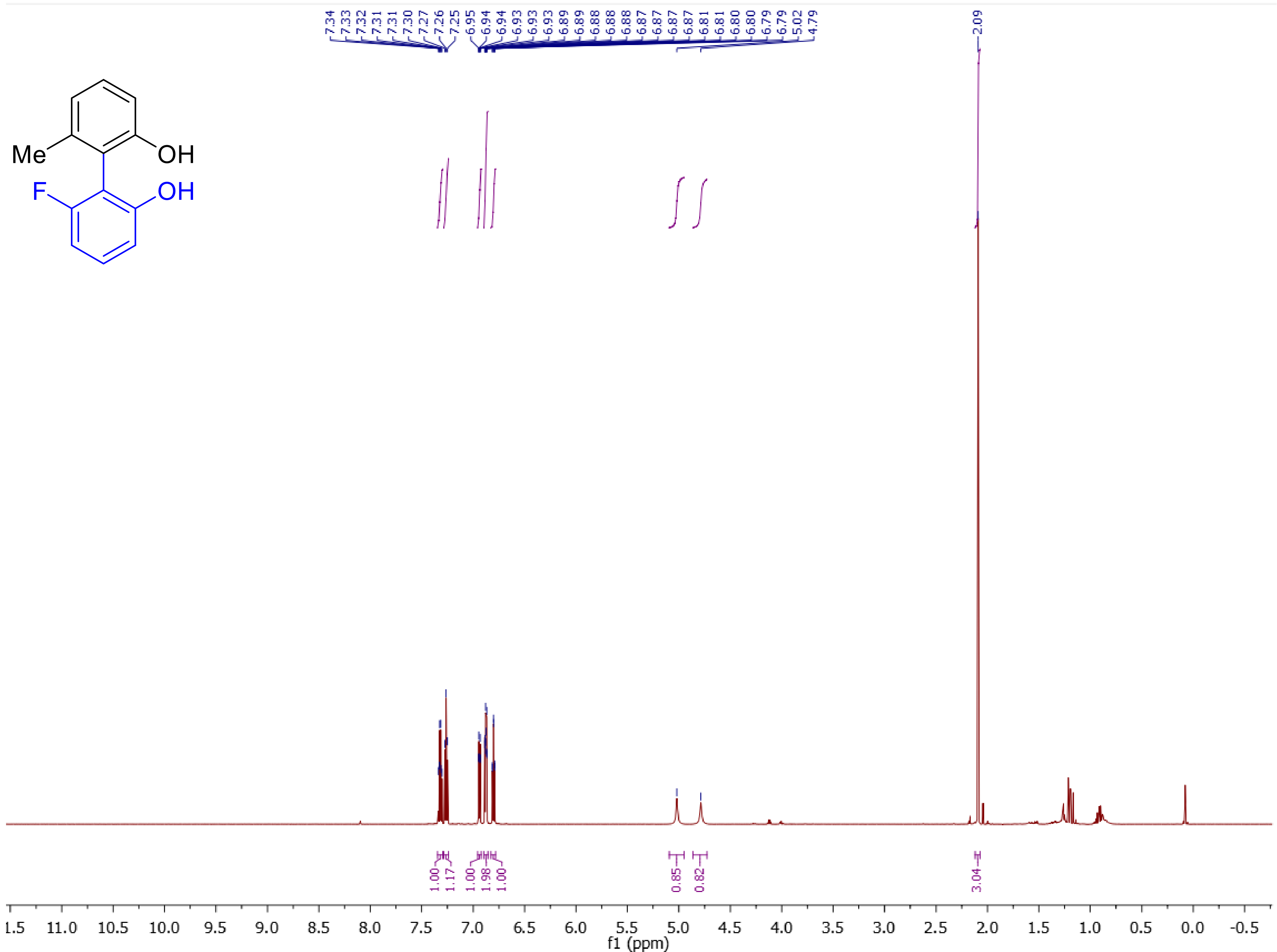

^{19}F-NMR ($CDCl_3$): (R)-6-fluoro-6'-methyl-[1,1'-biphenyl]-2,2'-diol (4i)

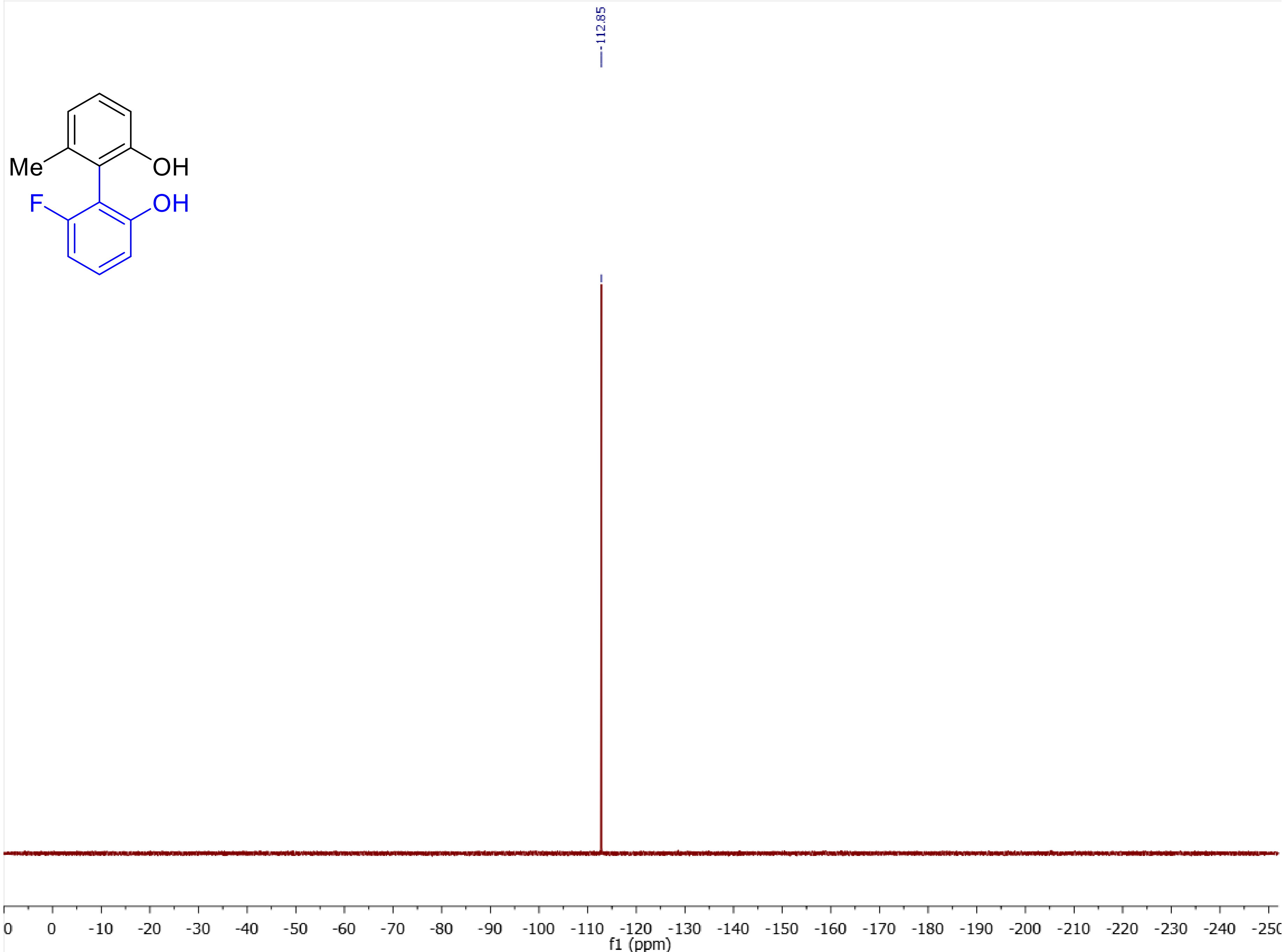

^{13}C-NMR ($CDCl_3$): (R)-6-fluoro-6'-methyl-[1,1'-biphenyl]-2,2'-diol (4i)

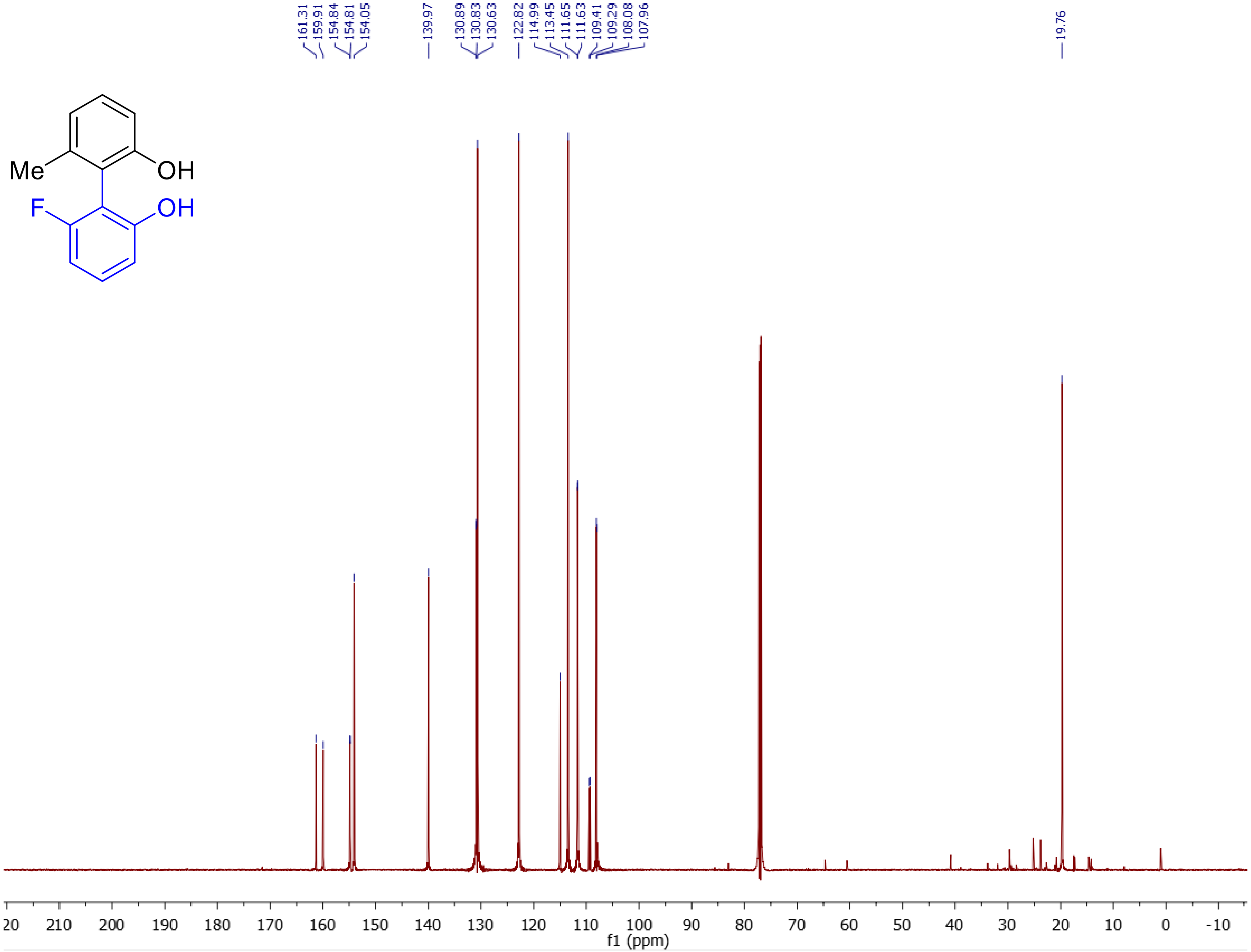

^{1}H-NMR ($CDCl_3$): (R)-2'-fluoro-6'-methoxy-6-methyl-[1,1'-biphenyl]-2-ol (4j)

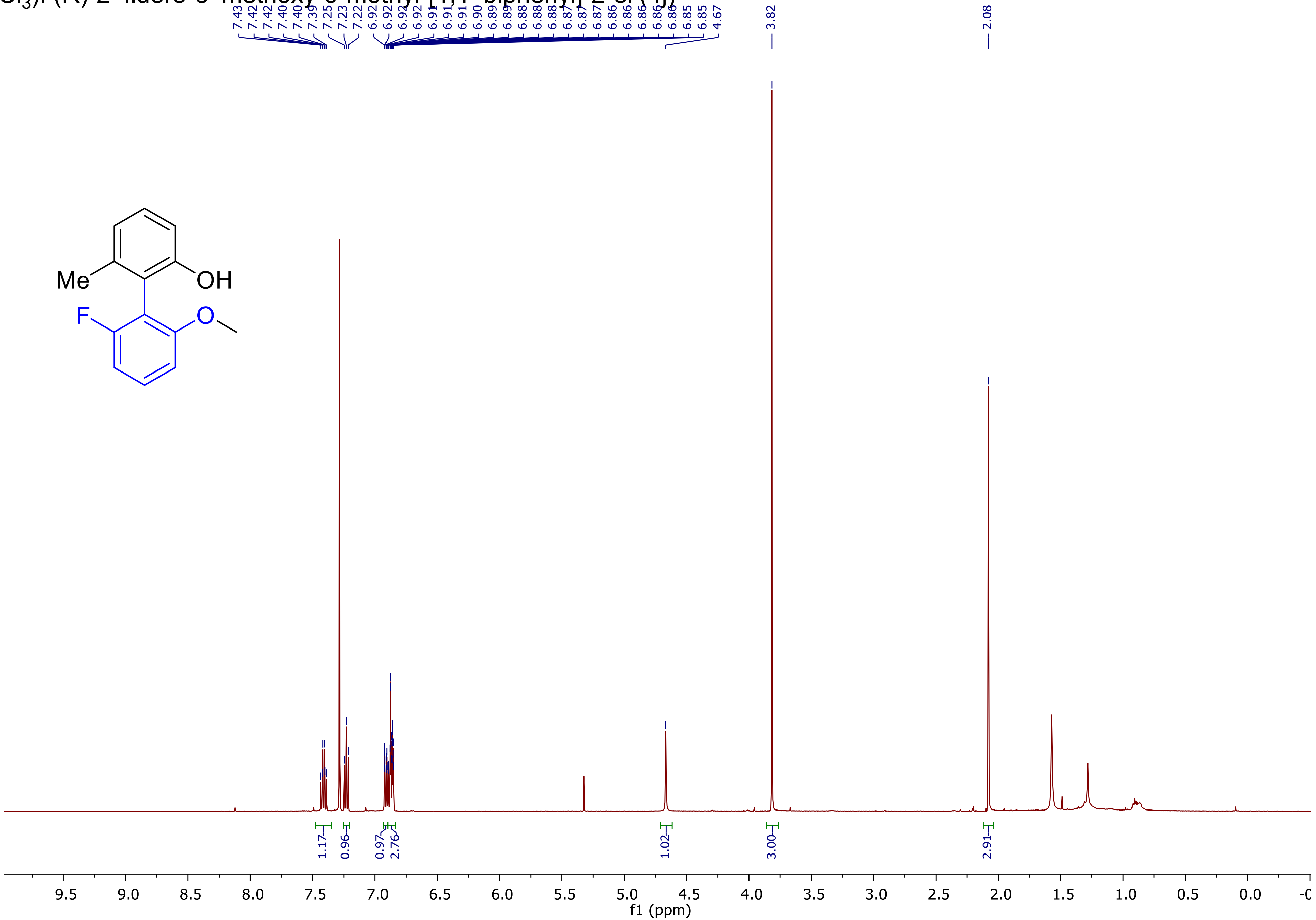

^{13}C-NMR ($CDCl_3$): (R)-2'-fluoro-6'-methoxy-6-methyl-[1,1'-biphenyl]-2-ol (4j)

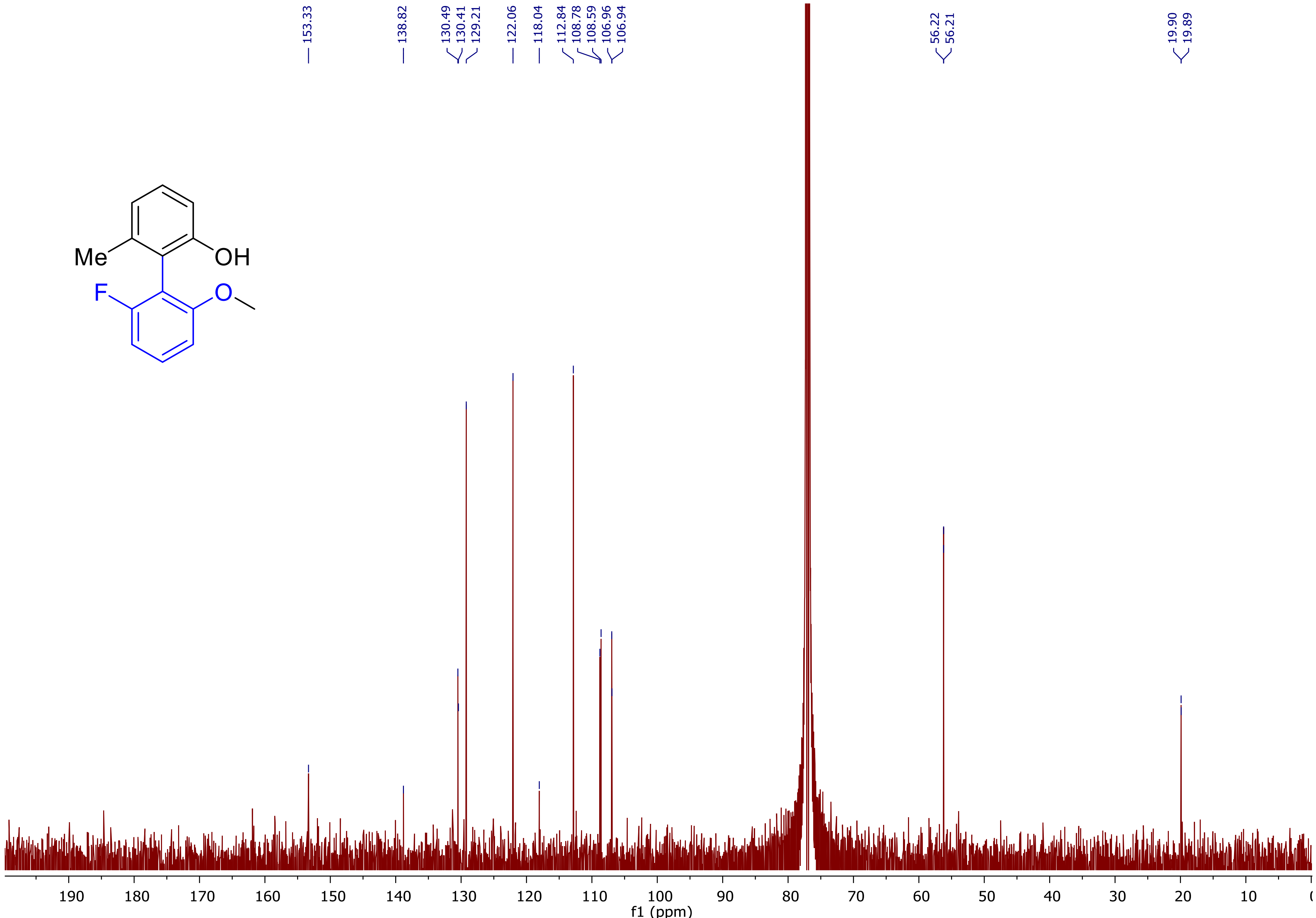

^{1}H-NMR ($CDCl_3$): 6-chloro-2'-(trifluoromethyl)-[1,1'-biphenyl]-2-ol (5a)

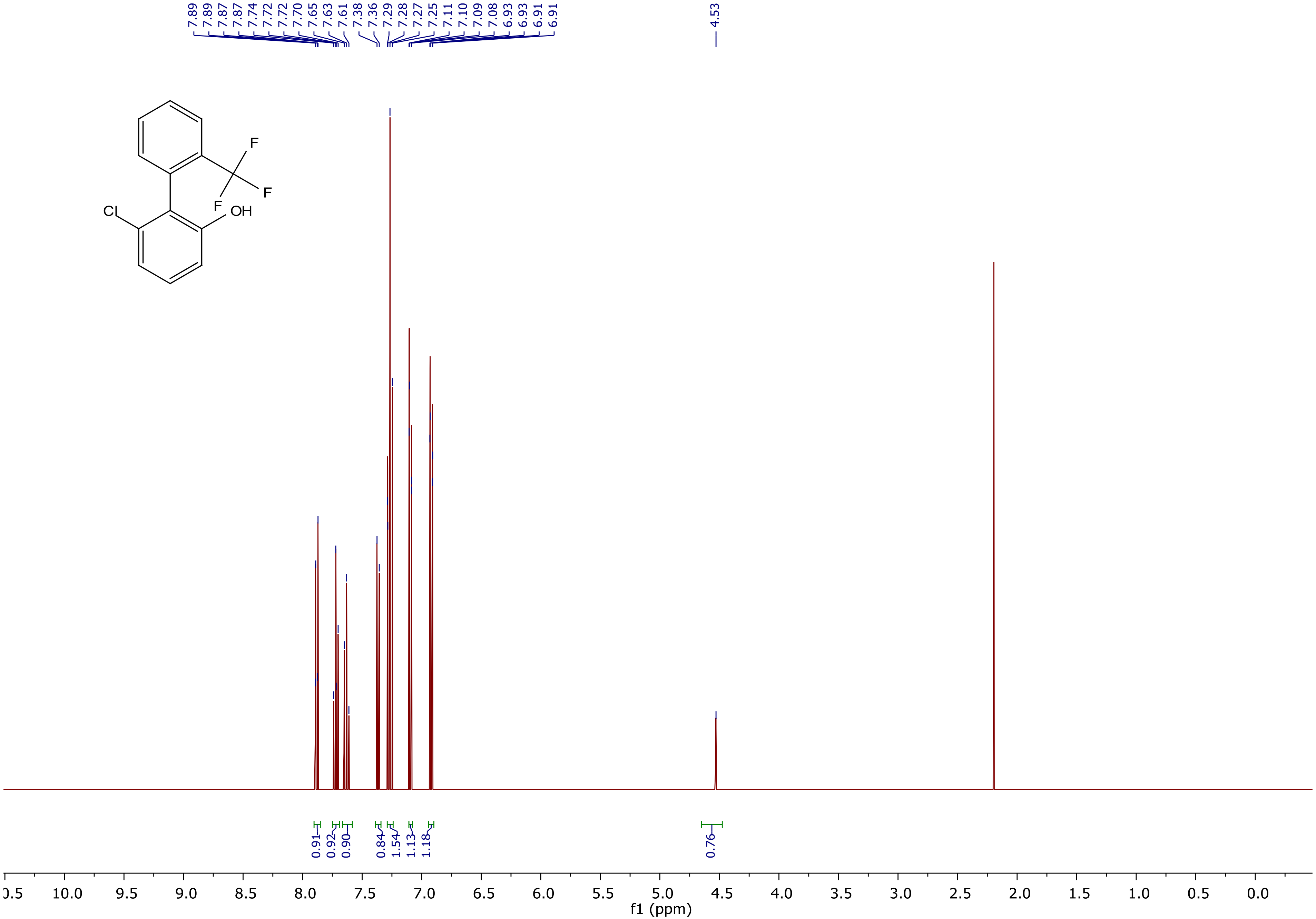

^{19}F-NMR ($CDCl_3$): 6-chloro-2'-(trifluoromethyl)-[1,1'-biphenyl]-2-ol (5a)

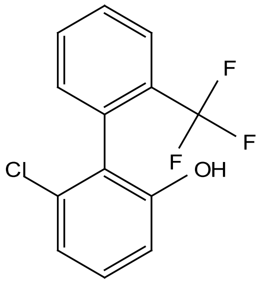

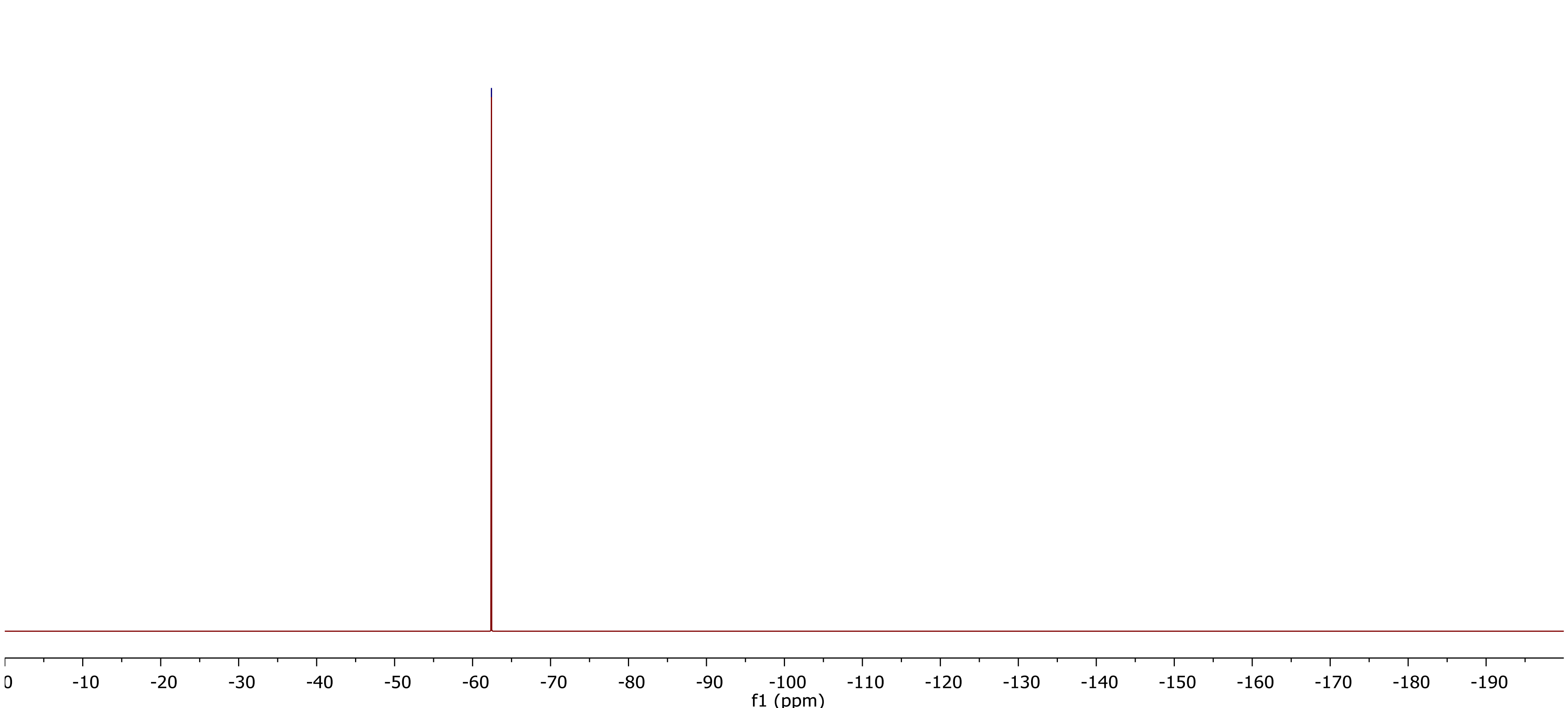

^{13}C-NMR ($CDCl_3$): *6-chloro-2'-(trifluoromethyl)-[1,1'-biphenyl]-2-ol (5a)*

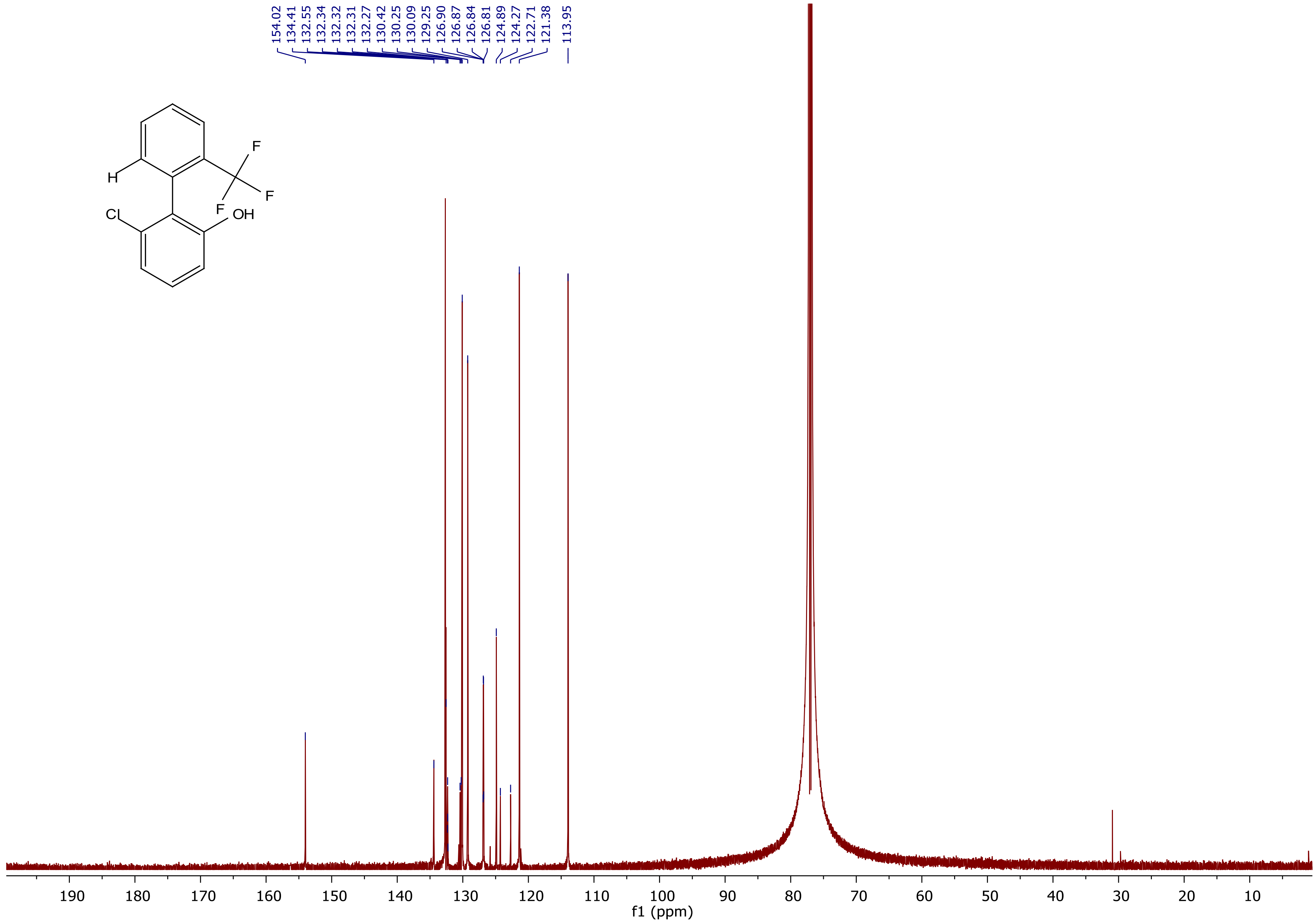

¹H-NMR ($CDCl_3$): *(S)*-2'-chloro-6-fluoro-6'-methyl-[1,1'-biphenyl]-2-ol (5b)

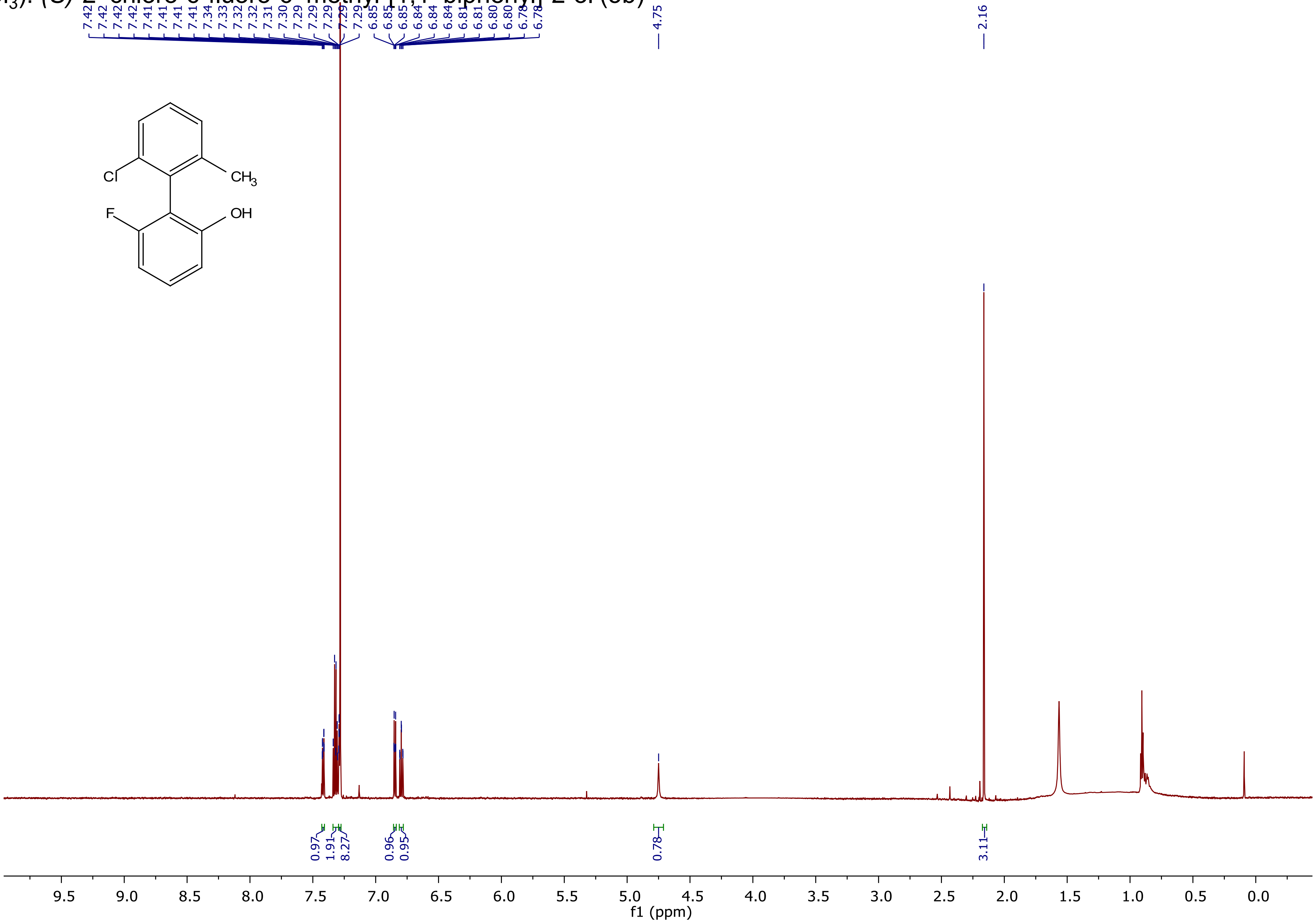

^{13}C-NMR ($CDCl_3$): *(S)*-2'-chloro-6-fluoro-6'-methyl-[1,1'-biphenyl]-2-ol (5b)

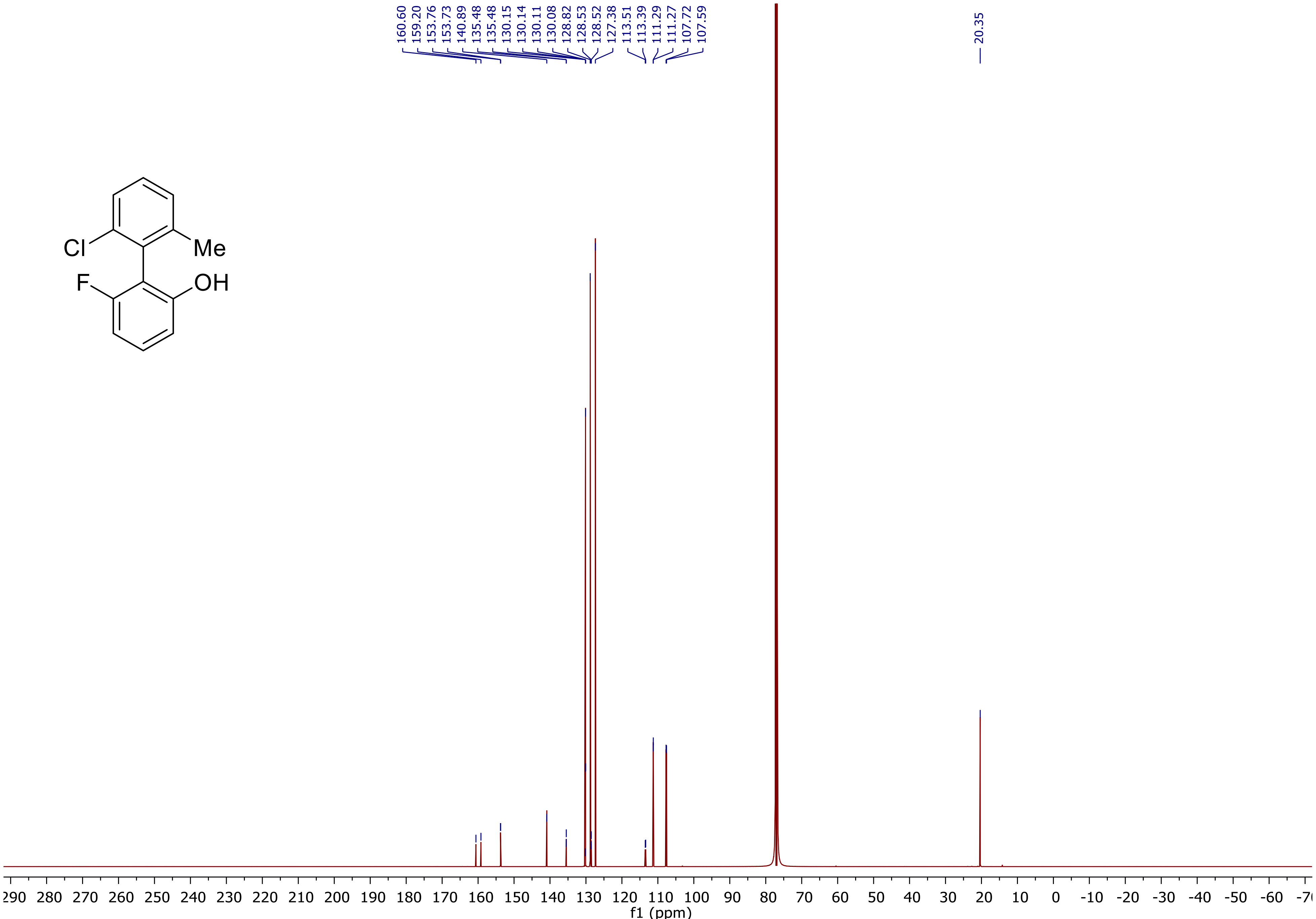

^{1}H-NMR ($CDCl_3$): (S)-2'-amino-6-fluoro-6'-methoxy-[1,1'-biphenyl]-2-ol (5c)

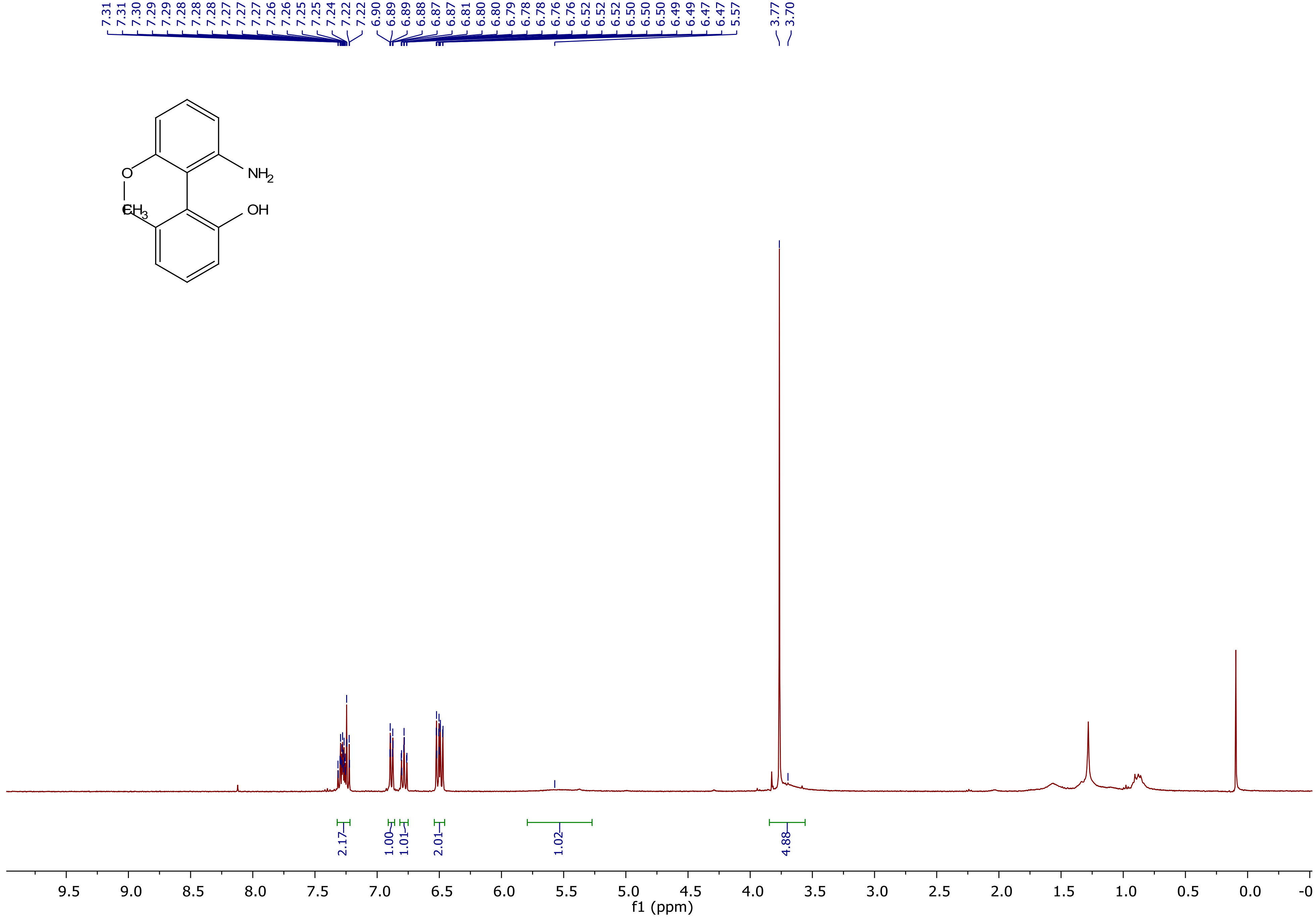

^{19}F-NMR ($CDCl_3$): (S)-2'-amino-6-fluoro-6'-methoxy-[1,1'-biphenyl]-2-ol (5c)

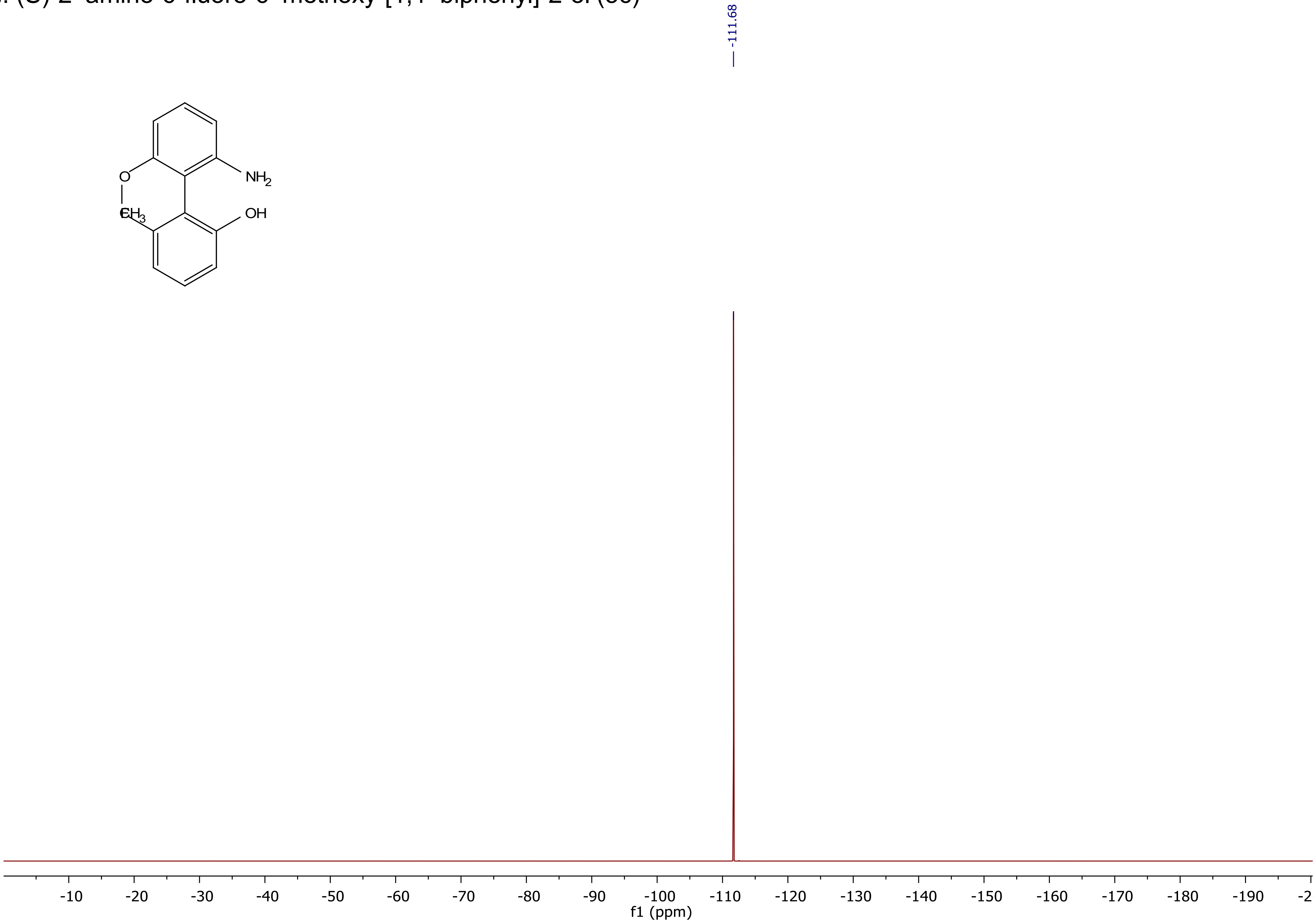

^{13}C-NMR ($CDCl_3$): (S)-2'-amino-6-fluoro-6'-methoxy-[1,1'-biphenyl]-2-ol (5c)

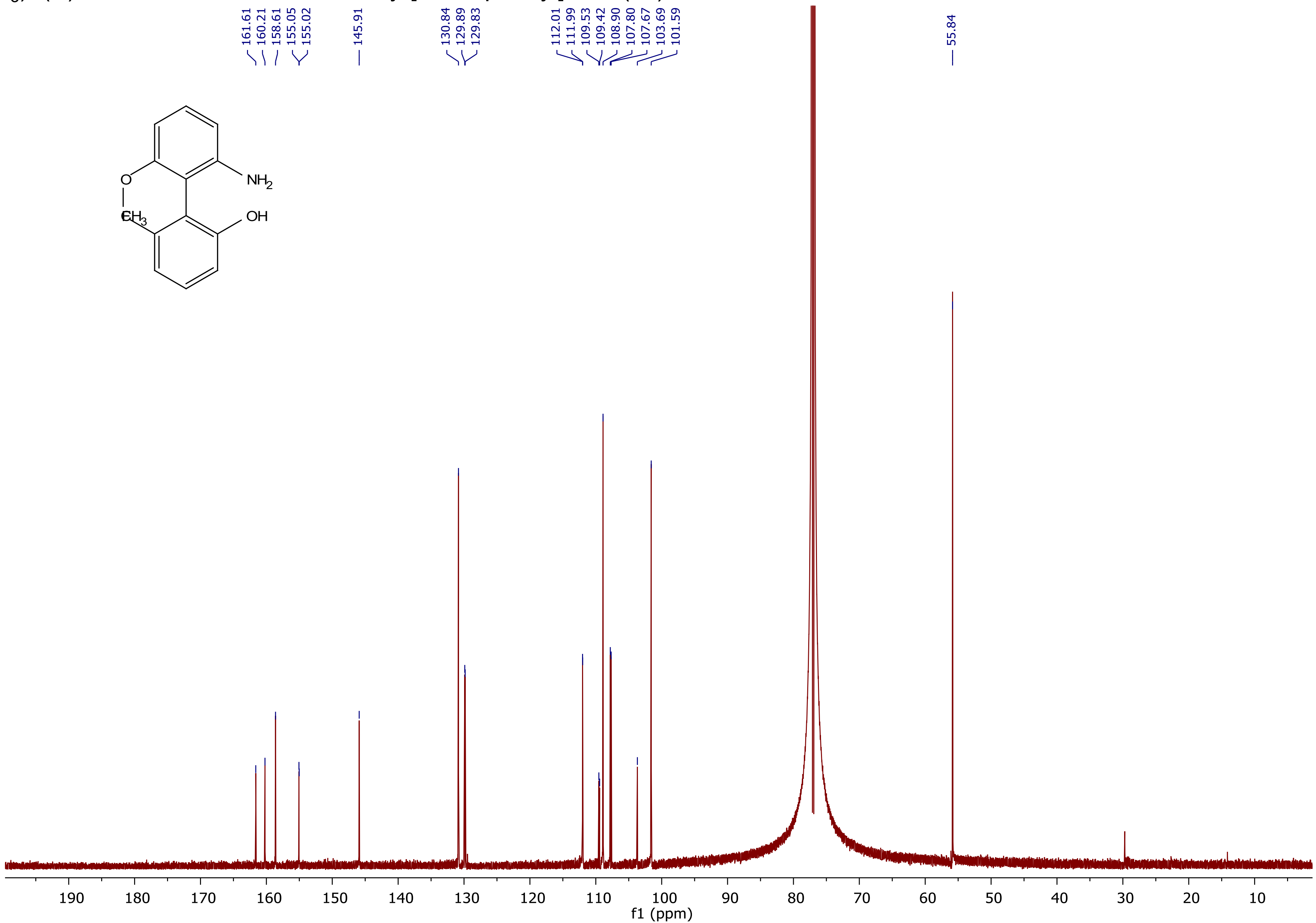

^{1}H-NMR ($CDCl_3$): *(R)*-6-amino-6'-fluoro-[1,1'-biphenyl]-2,2'-diol (5d)

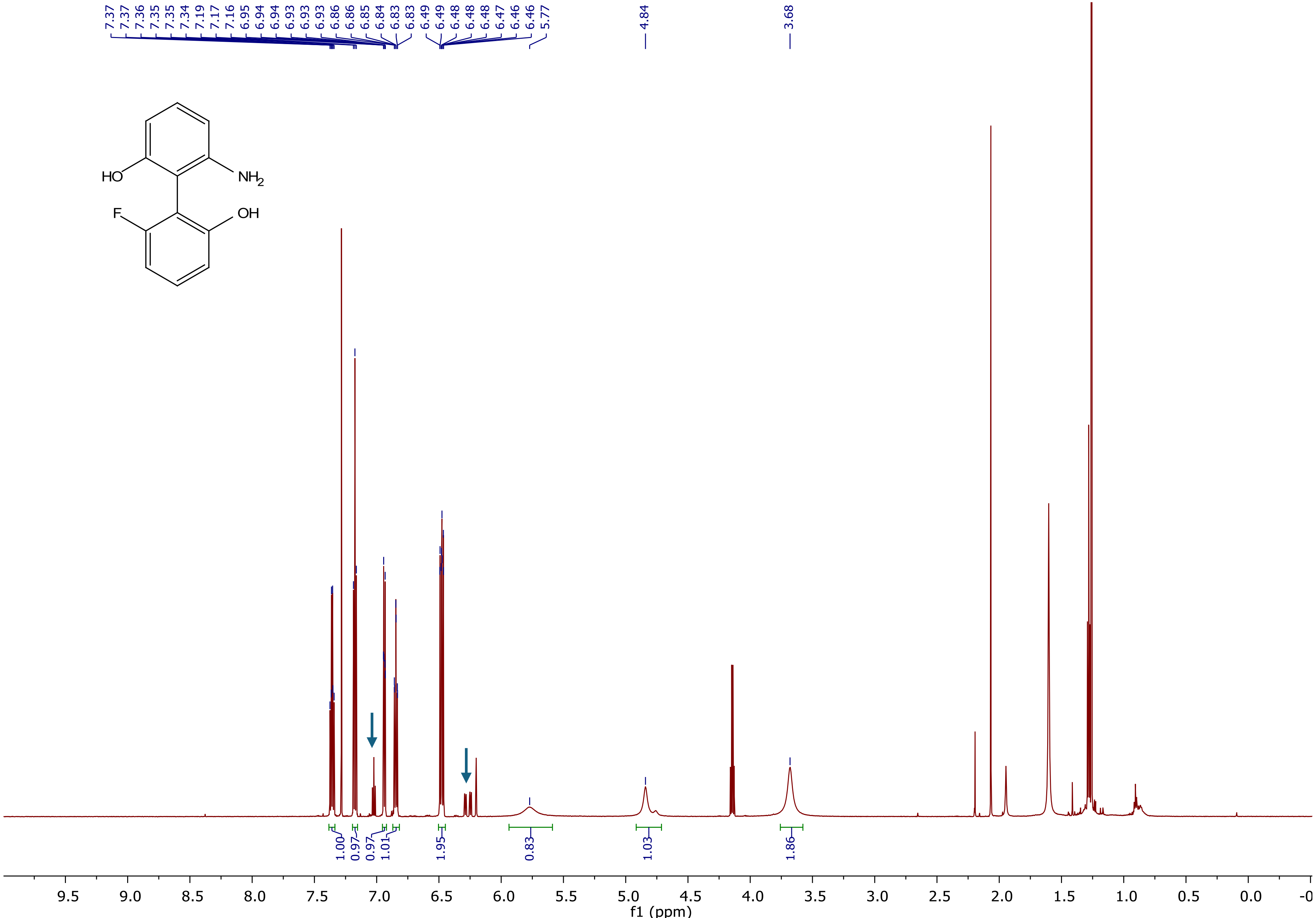

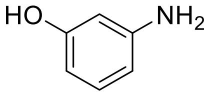

Impurity peaks ↓

^{19}F-NMR ($CDCl_3$): *(R)*-6-amino-6'-fluoro-[1,1'-biphenyl]-2,2'-diol (5d)

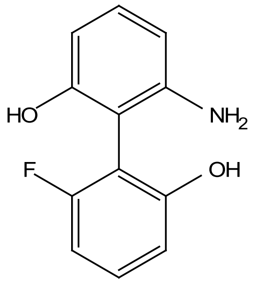

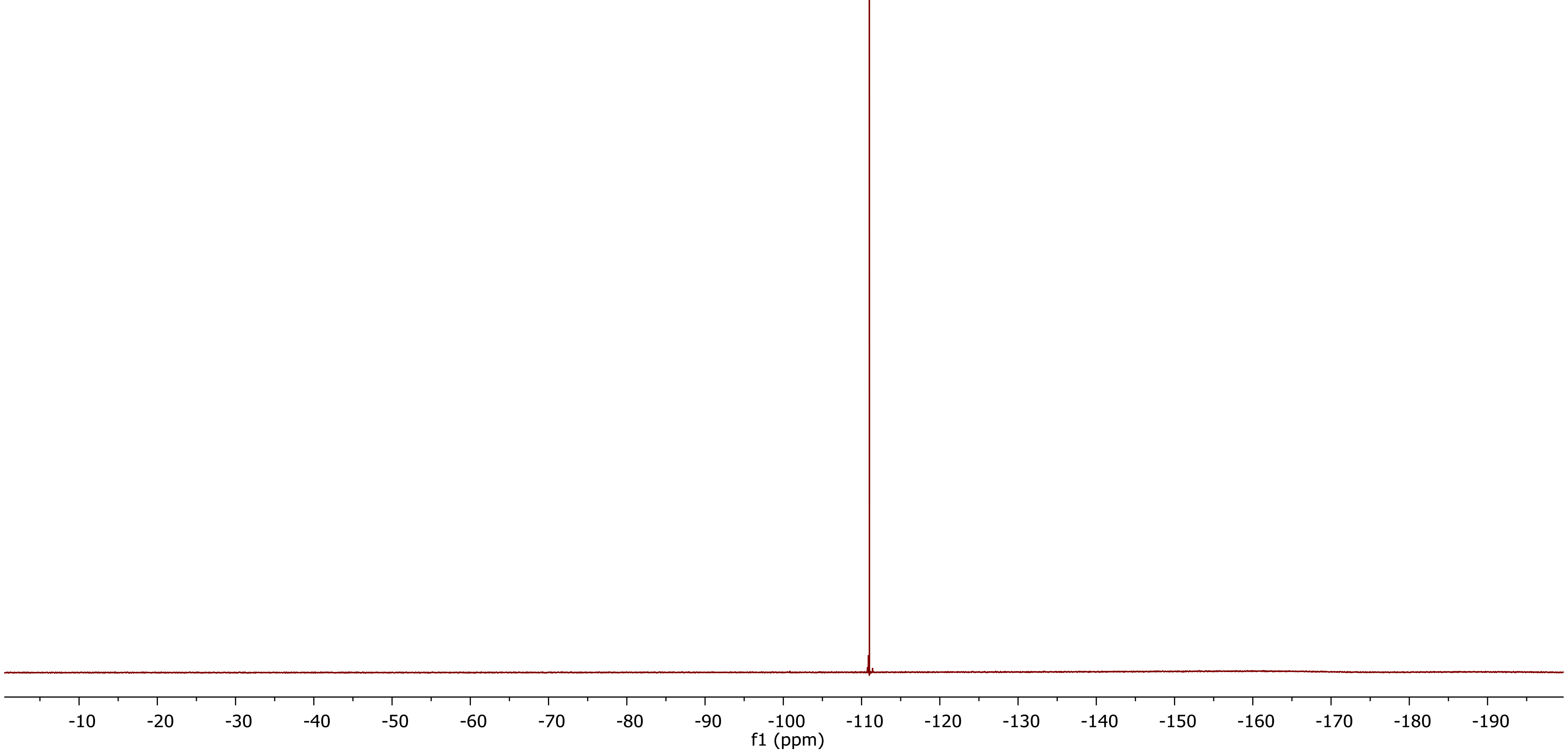

^{13}C-NMR ($CDCl_3$): *(R)*-6-amino-6'-fluoro-[1,1'-biphenyl]-2,2'-diol (5d)

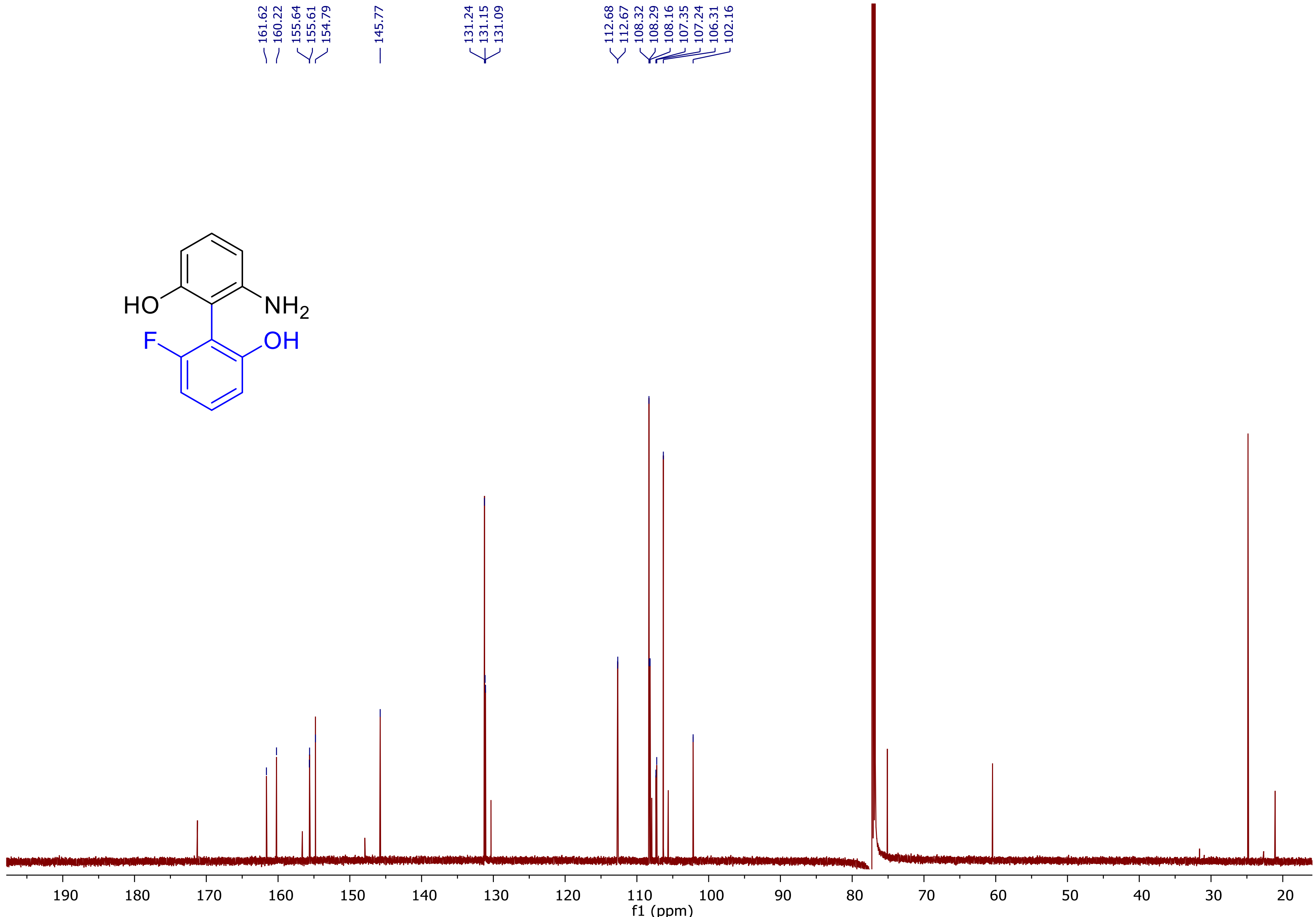

^{1}H-NMR ($CDCl_3$): (R)-N-(2'-fluoro-6'-hydroxy-6-methyl-[1,1'-biphenyl]-2-yl)acetamide (5f)

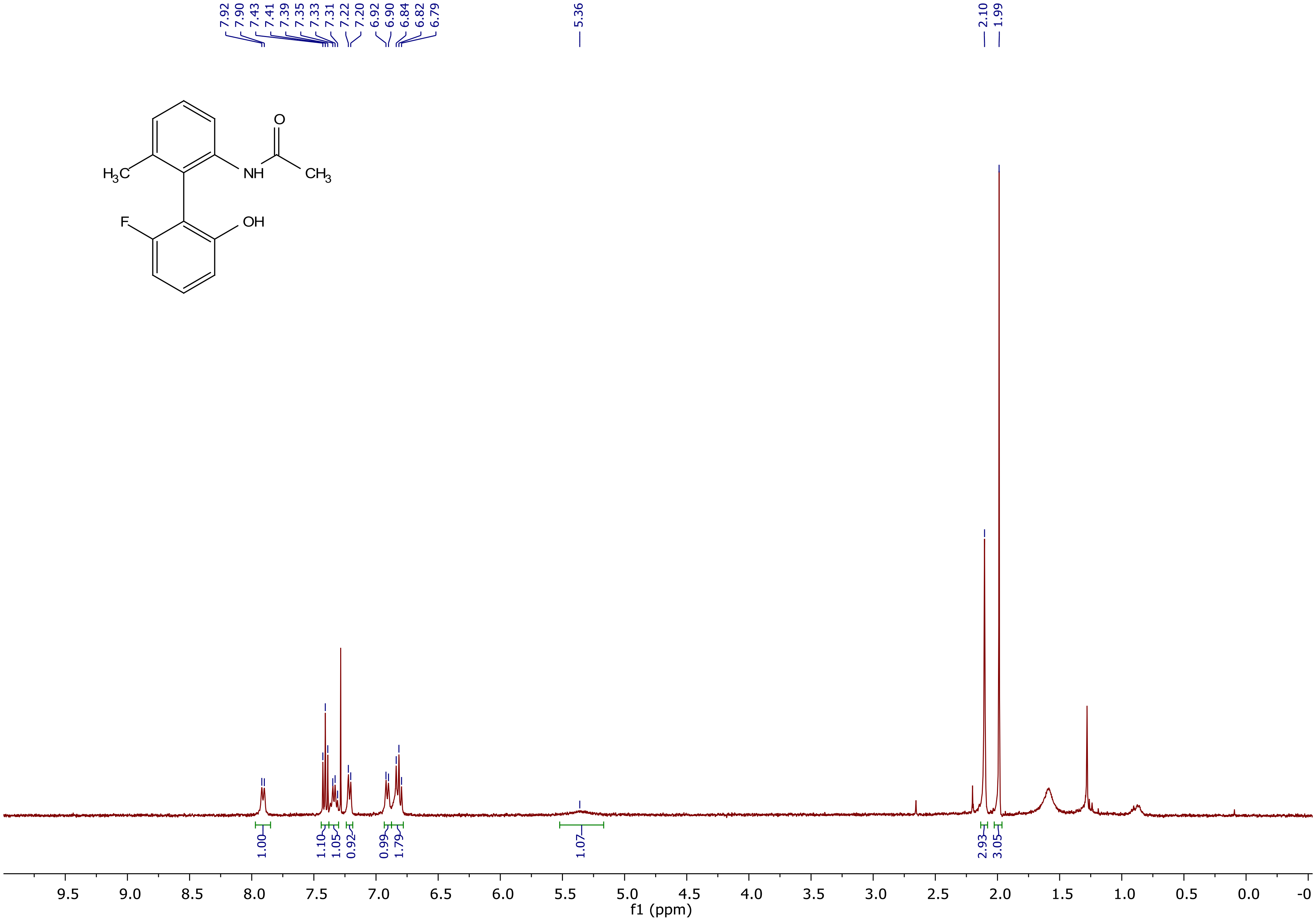

^{19}F-NMR ($CDCl_3$): (R)-N-(2'-fluoro-6'-hydroxy-6-methyl-[1,1'-biphenyl]-2-yl)acetamide (5f)

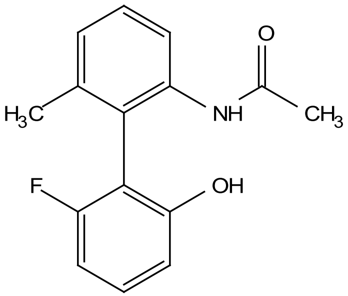

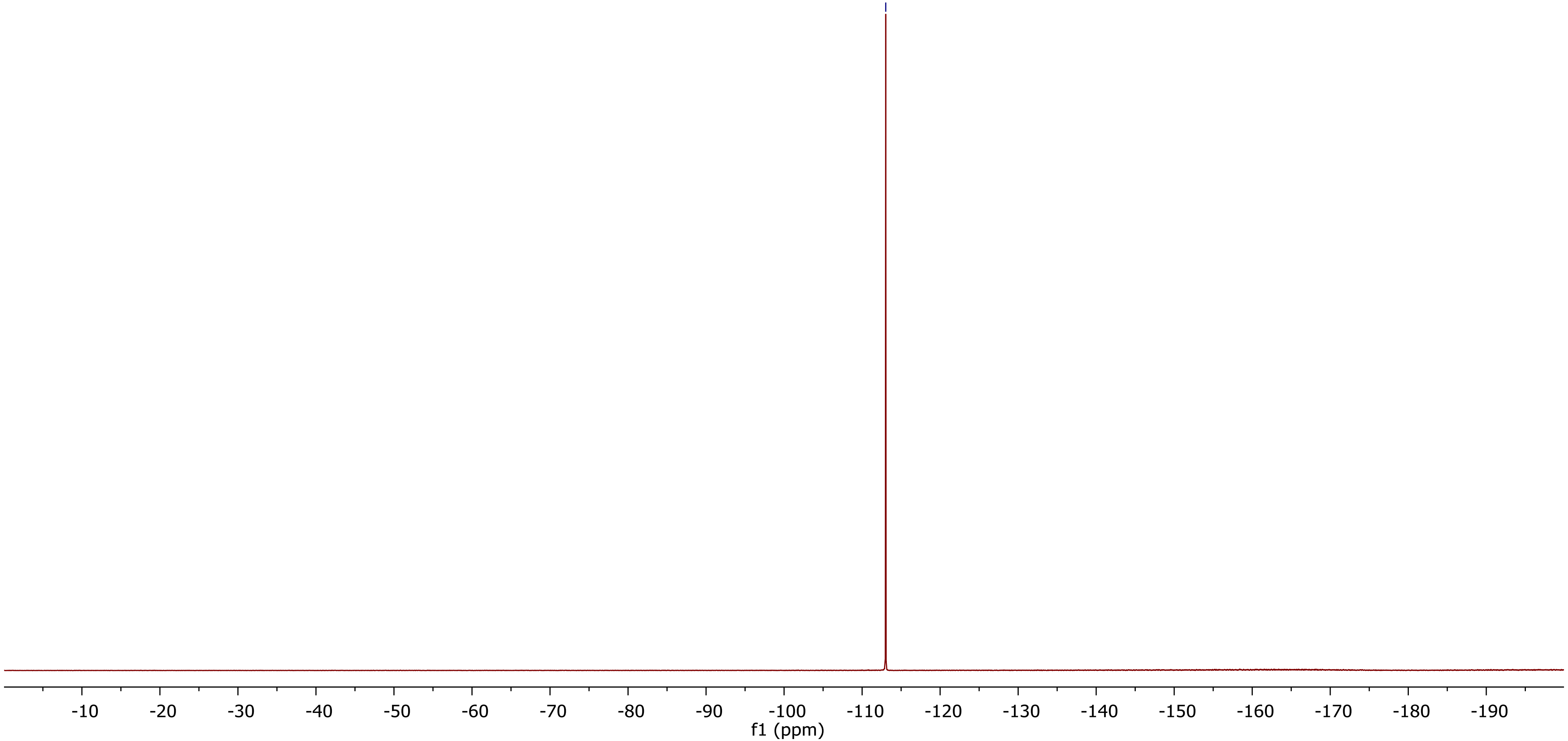

^{13}C-NMR ($CDCl_3$): (R)-N-(2'-fluoro-6'-hydroxy-6-methyl-[1,1'-biphenyl]-2-yl)acetamide (5f)

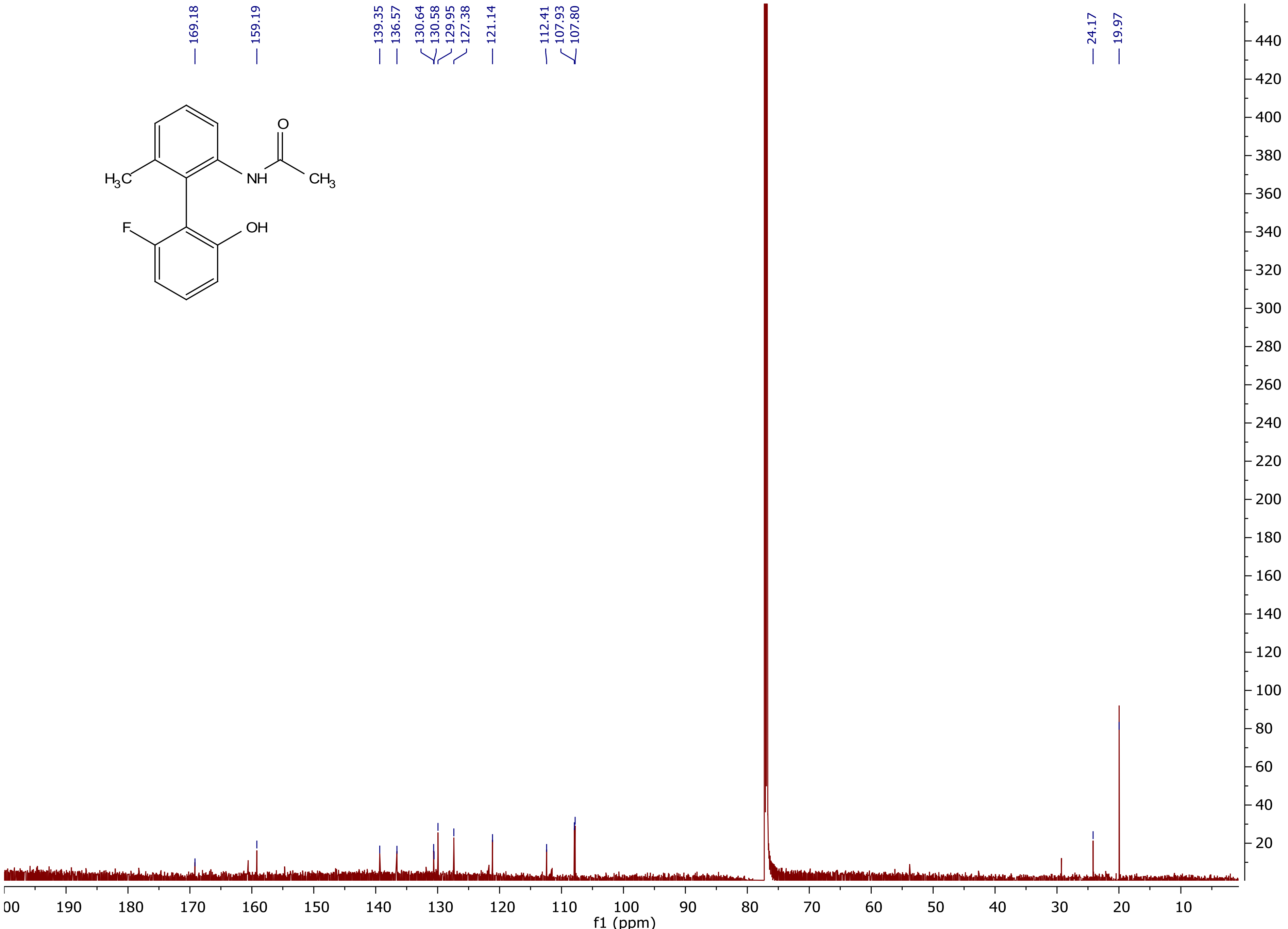

^{1}H-NMR ($CDCl_3$): (R)-2,2,2-trifluoro-N-(2'-fluoro-6'-hydroxy-6-methyl-[1,1'-biphenyl]-2-yl)acetamide (5g)

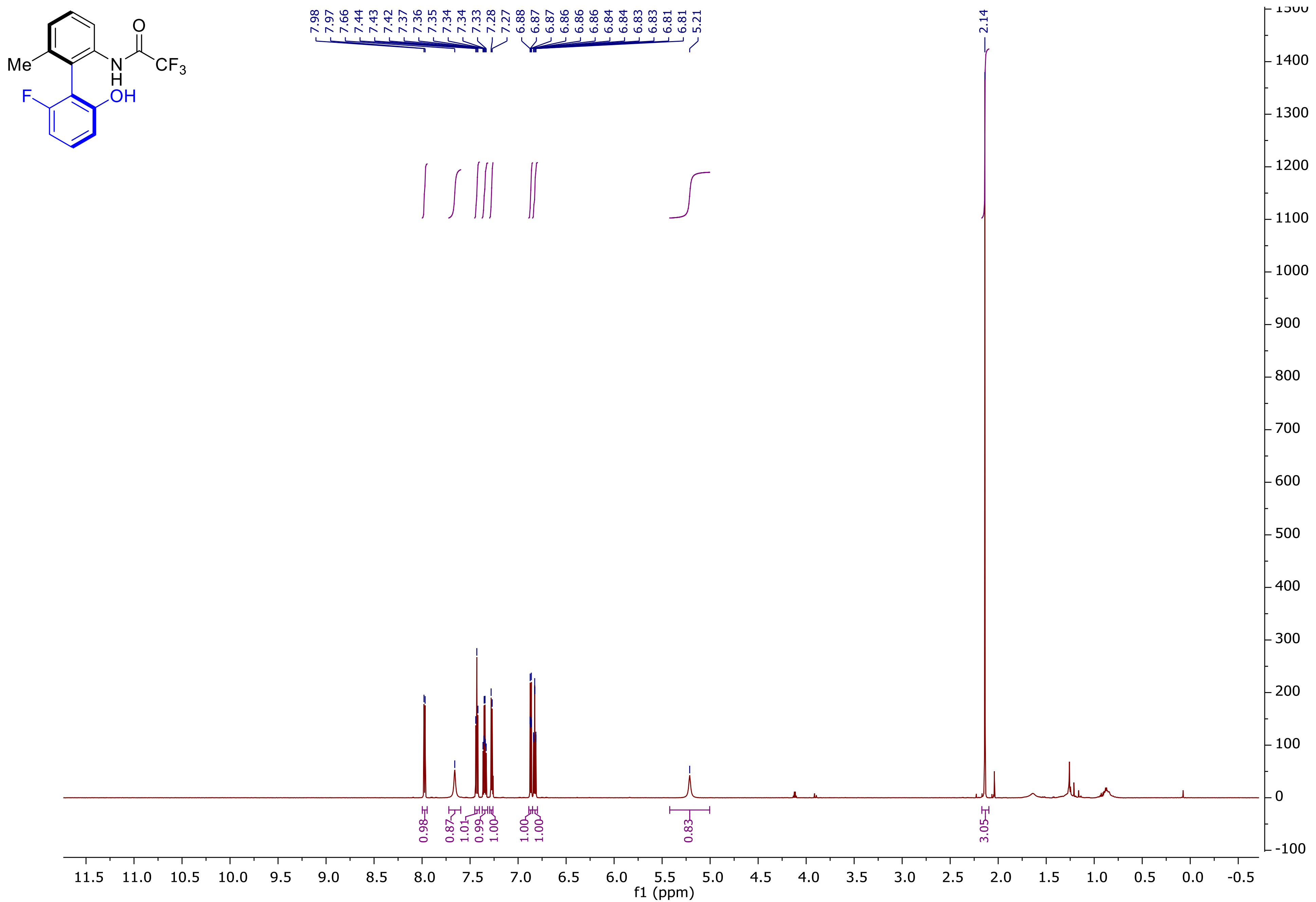

^{19}F-NMR ($CDCl_3$): (R)-2,2,2-trifluoro-N-(2'-fluoro-6'-hydroxy-6-methyl-[1,1'-biphenyl]-2-yl)acetamide (5g)

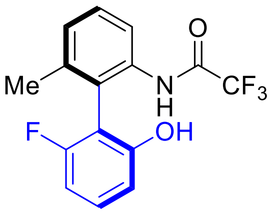

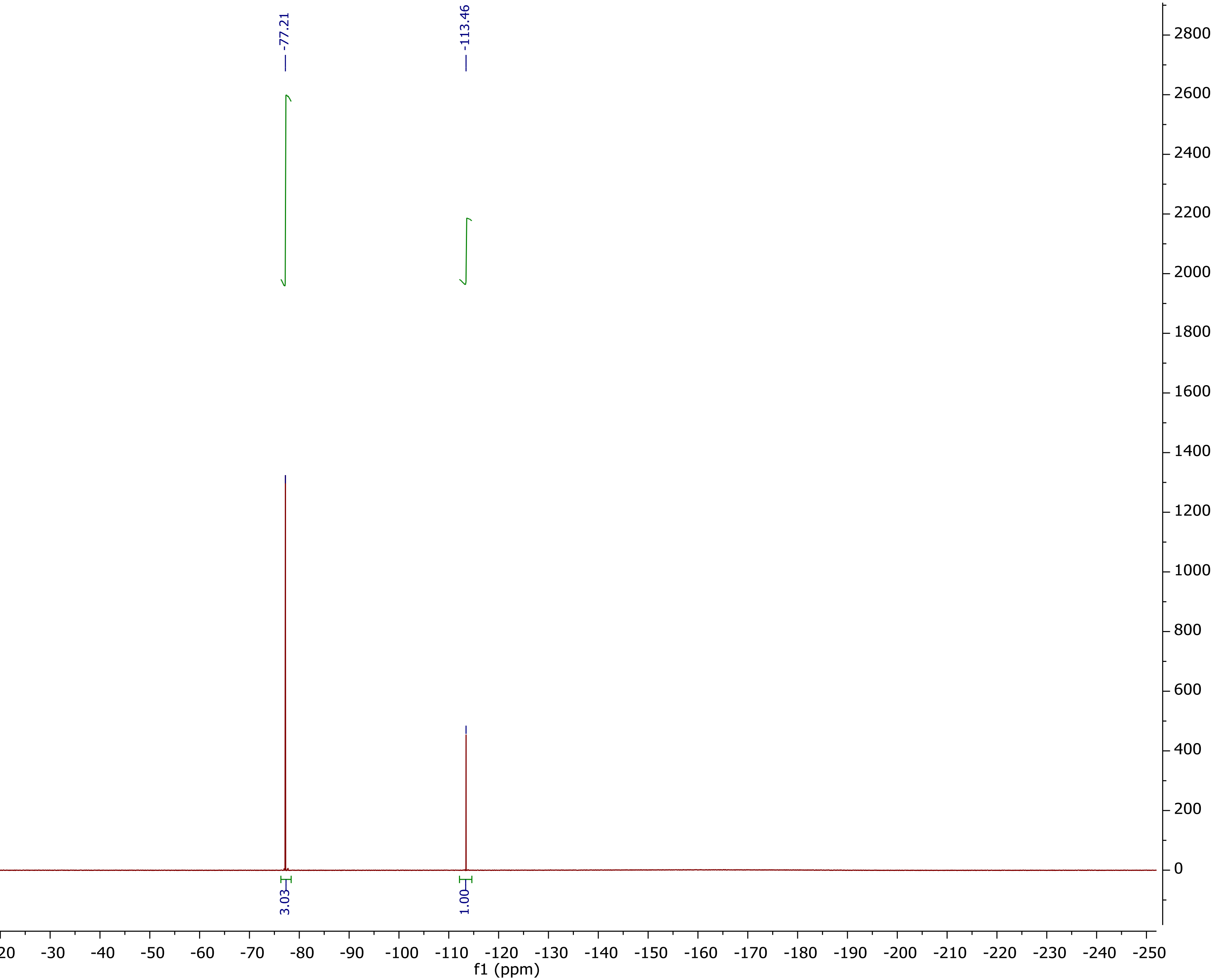

^{13}C-NMR ($CDCl_3$): (R)-2,2,2-trifluoro-N-(2'-fluoro-6'-hydroxy-6-methyl-[1,1'-biphenyl]-2-yl)acetamide (5g)

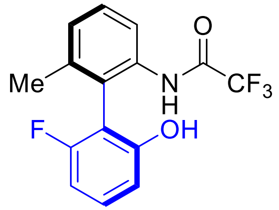

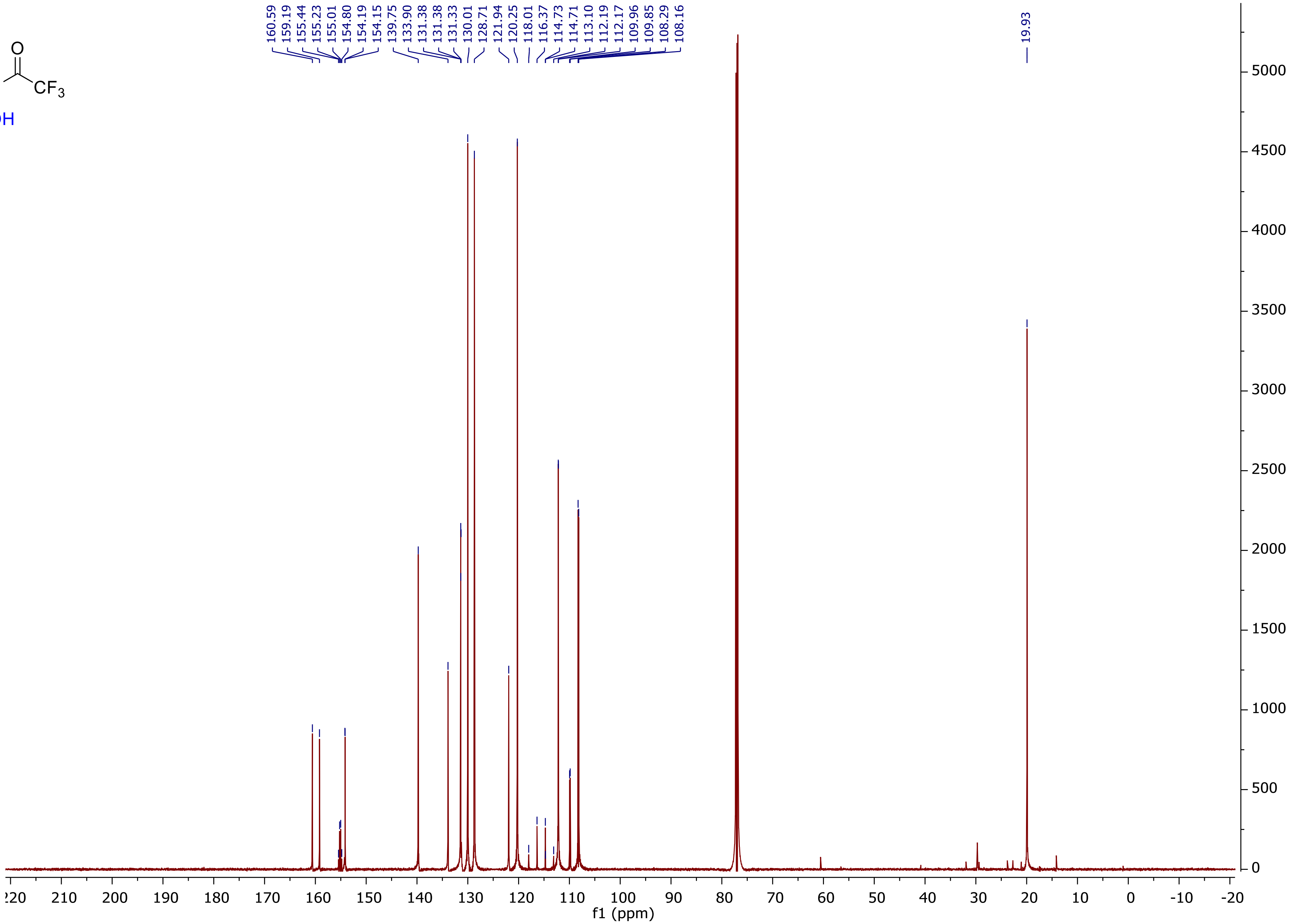

^{1}H-NMR ($CDCl_3$): (R)-N-(2'-fluoro-6'-hydroxy-6-methyl-[1,1'-biphenyl]-2-yl)-4-methylbenzenesulfonamide (5h)

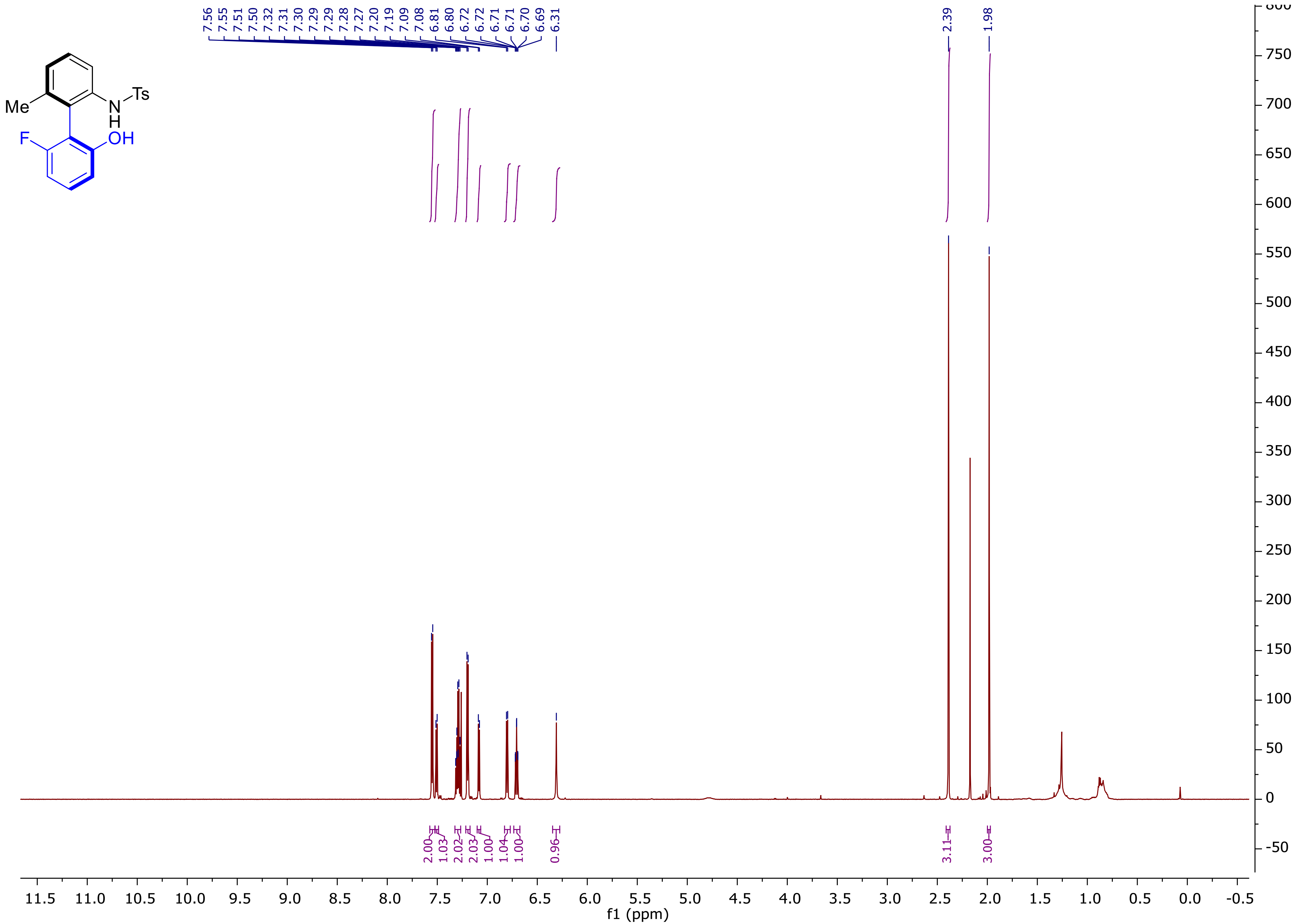

^{19}F-NMR ($CDCl_3$): (R)-N-(2'-fluoro-6'-hydroxy-6-methyl-[1,1'-biphenyl]-2-yl)-4-methylbenzenesulfonamide (5h)

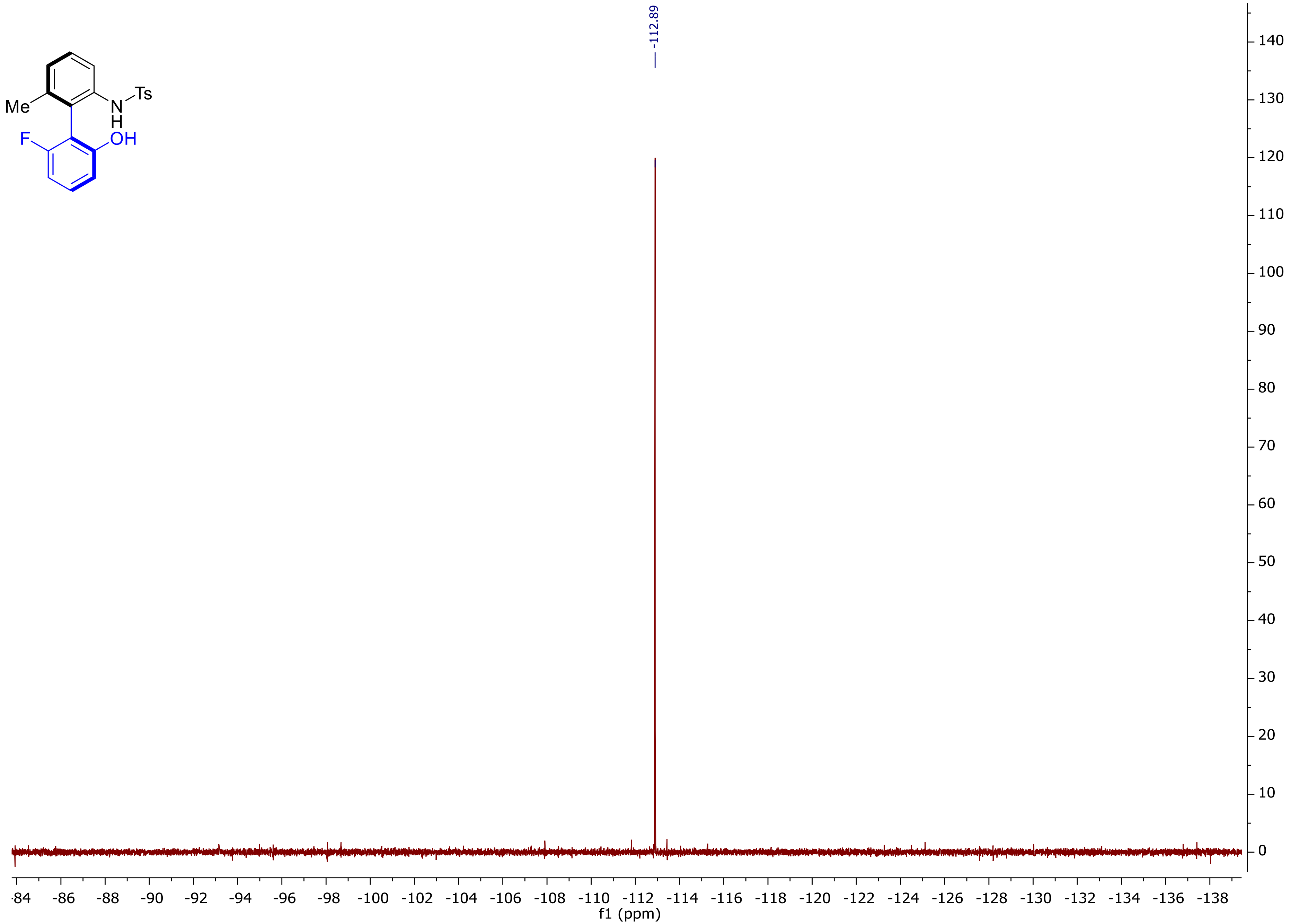

13C-NMR ($CDCl_3$): (R)-N-(2'-fluoro-6'-hydroxy-6-methyl-[1,1'-biphenyl]-2-yl)-4-methylbenzenesulfonamide (5h)

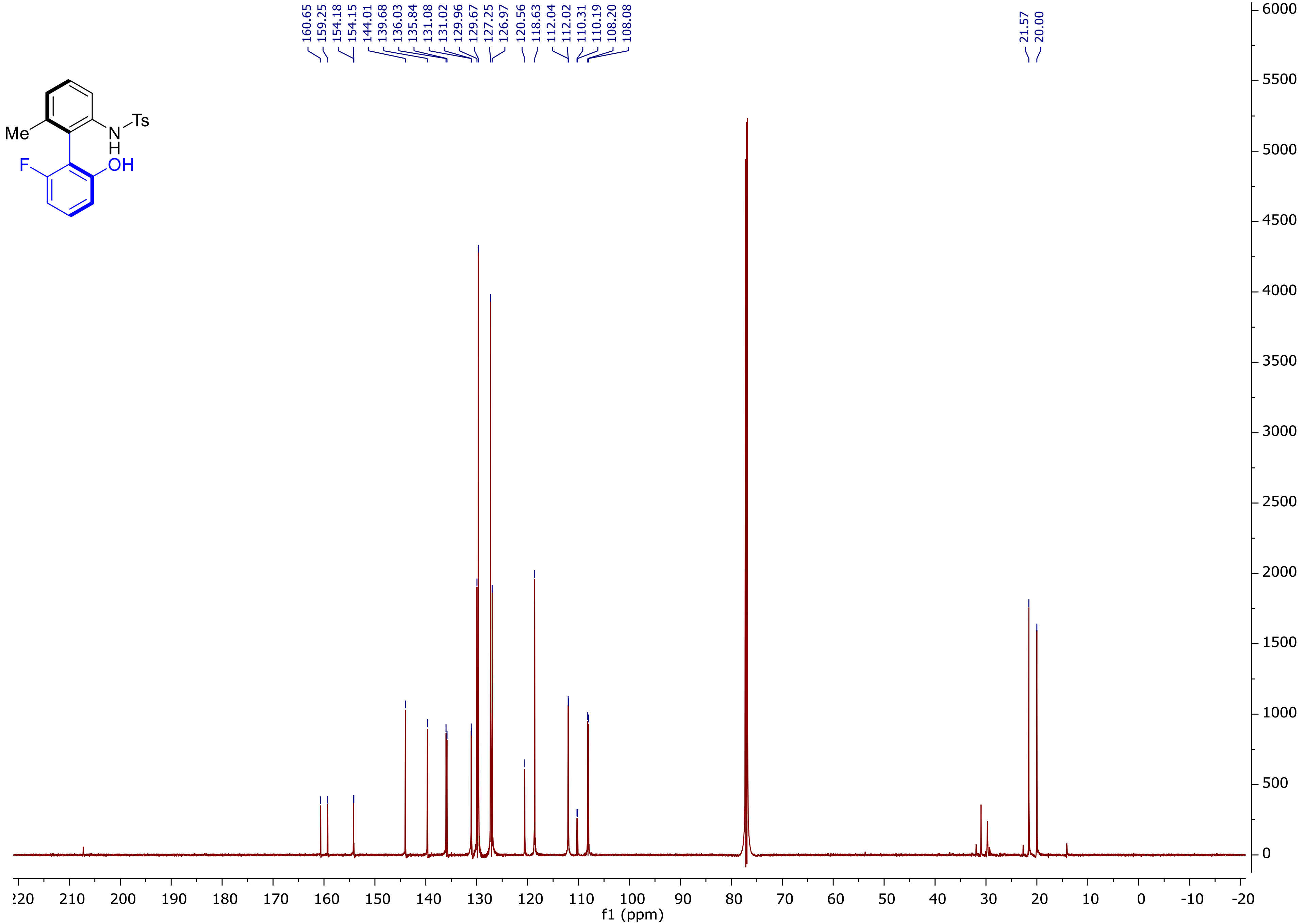

Supplement: Supplementary file 1 — Supporting Information [file ANIE-65-e20698-s001.pdf]
